# Supplementary figures and images for: PQBP3 prevents senescence by suppressing PSME3-mediated proteasomal Lamin B1 degradation (part 1 of 4)
Source: EMBO J. 2024 Aug 5;43(18):3968–99. doi: 10.1038/s44318-024-00192-4 (PMC11405525; doi:10.1038/s44318-024-00192-4)

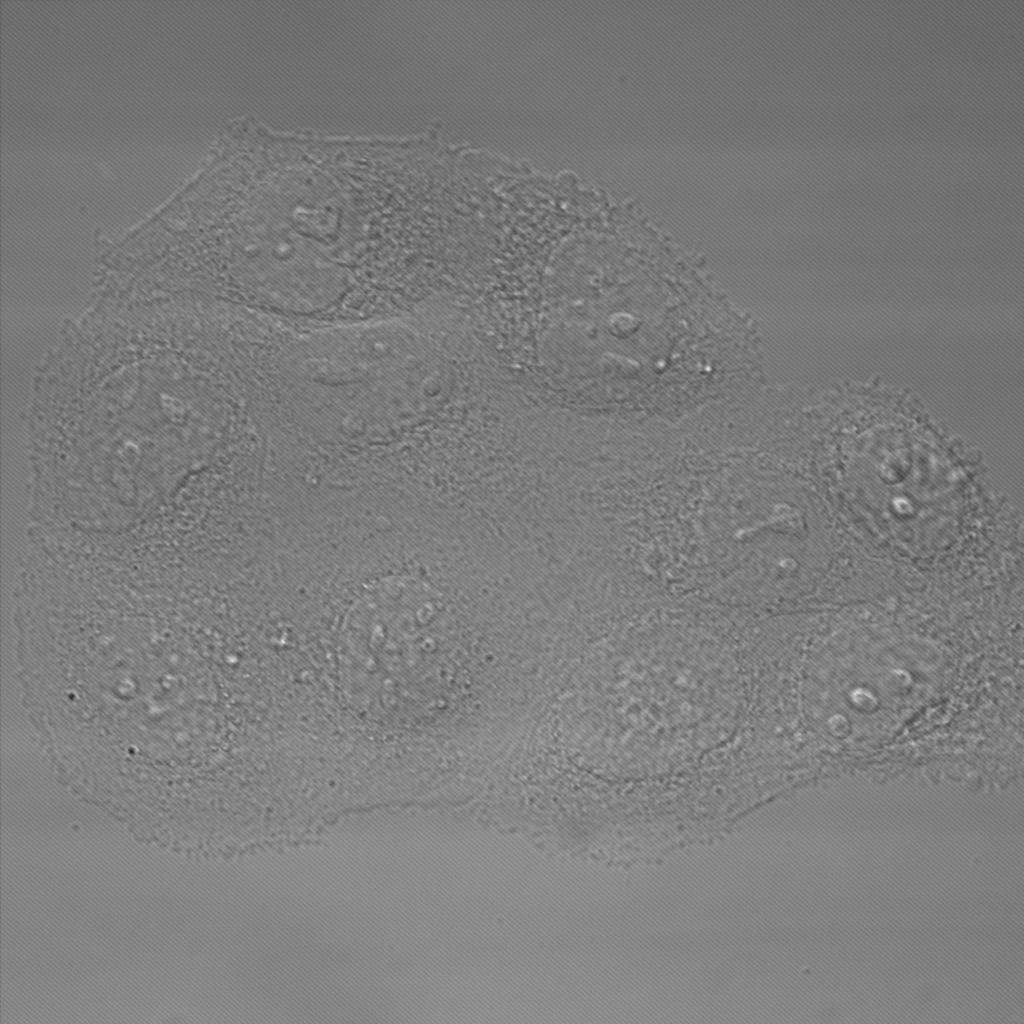

Supplement: Supplementary file 2 — Source data Fig. 1 [file 44318_2024_192_MOESM2_ESM.zip › Figure1/Figure1a/Bright field.tif]

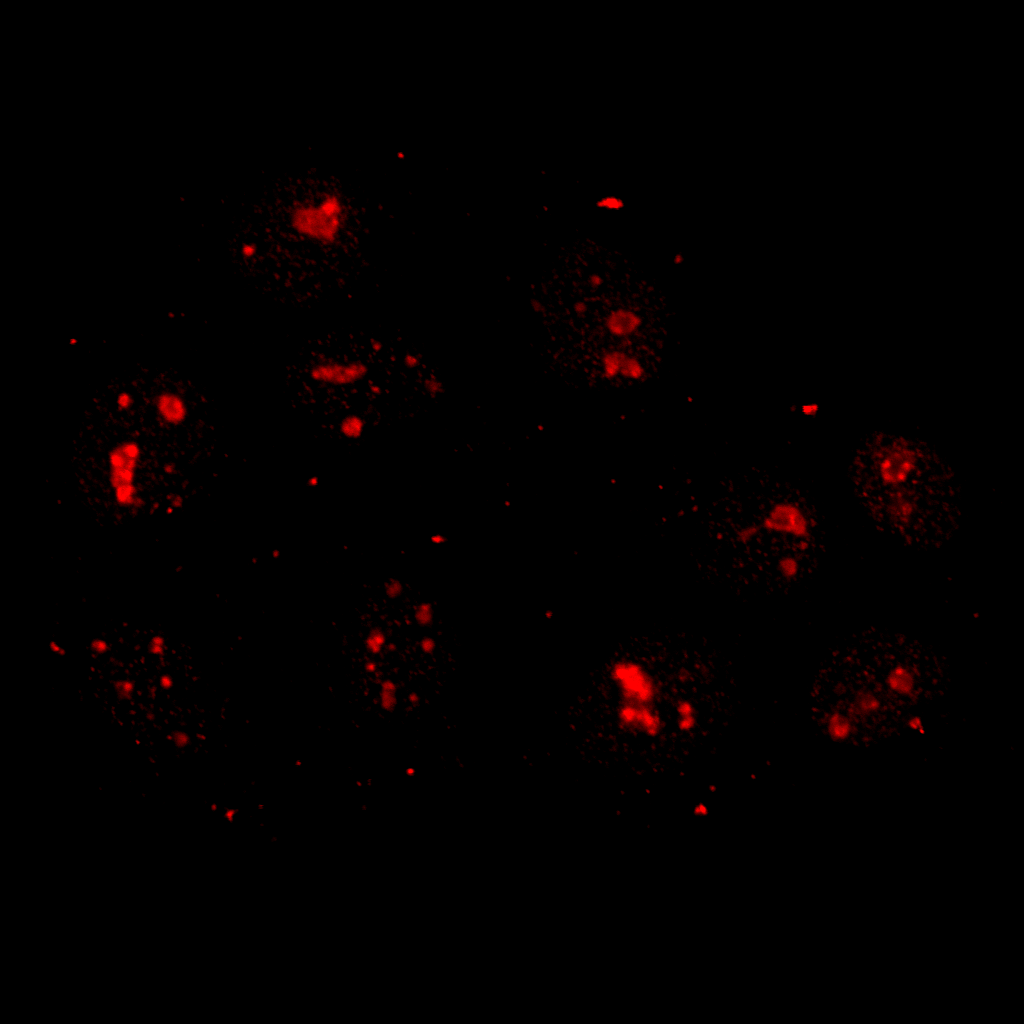

Supplement: Supplementary file 2 — Source data Fig. 1 [file 44318_2024_192_MOESM2_ESM.zip › Figure1/Figure1a/Fibrillarin.tif]

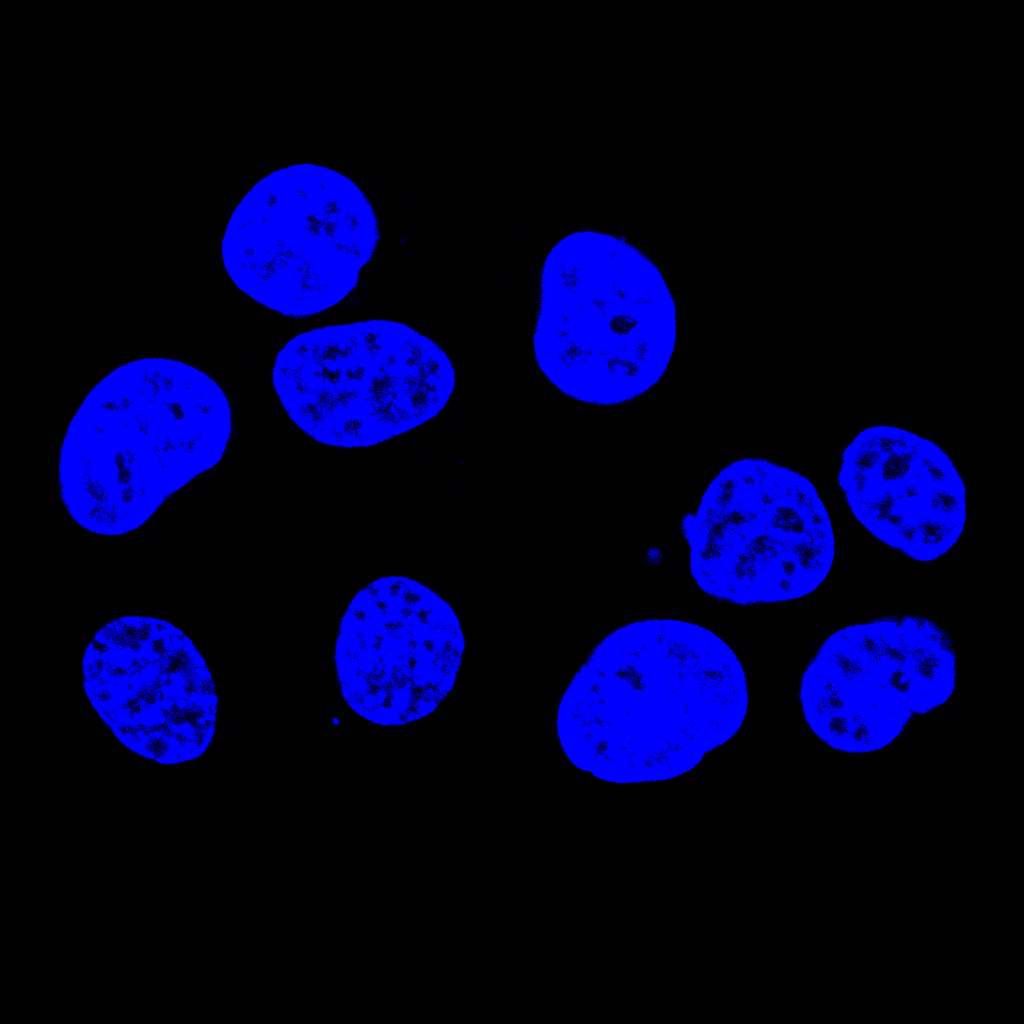

Supplement: Supplementary file 2 — Source data Fig. 1 [file 44318_2024_192_MOESM2_ESM.zip › Figure1/Figure1a/Hoechst 33342.tif]

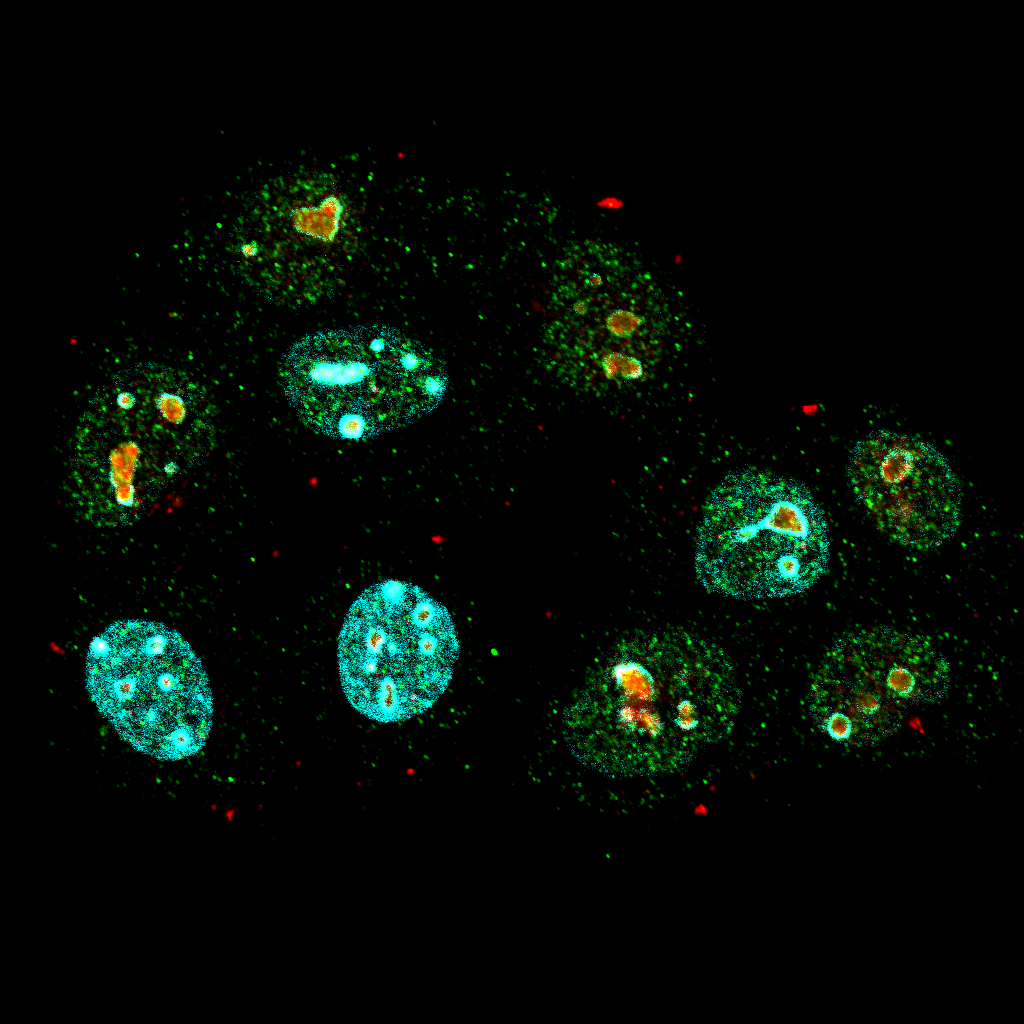

Supplement: Supplementary file 2 — Source data Fig. 1 [file 44318_2024_192_MOESM2_ESM.zip › Figure1/Figure1a/Merge.tif]

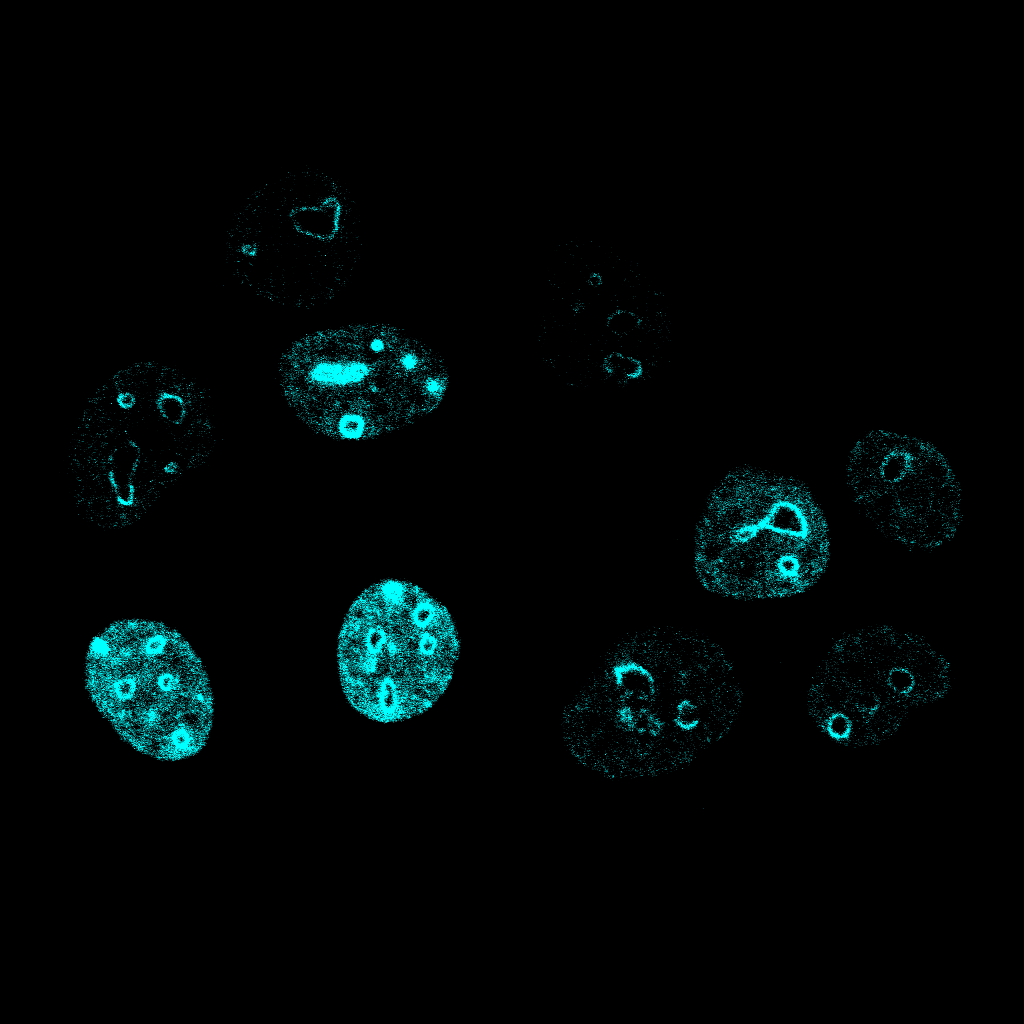

Supplement: Supplementary file 2 — Source data Fig. 1 [file 44318_2024_192_MOESM2_ESM.zip › Figure1/Figure1a/Nucleolin.tif]

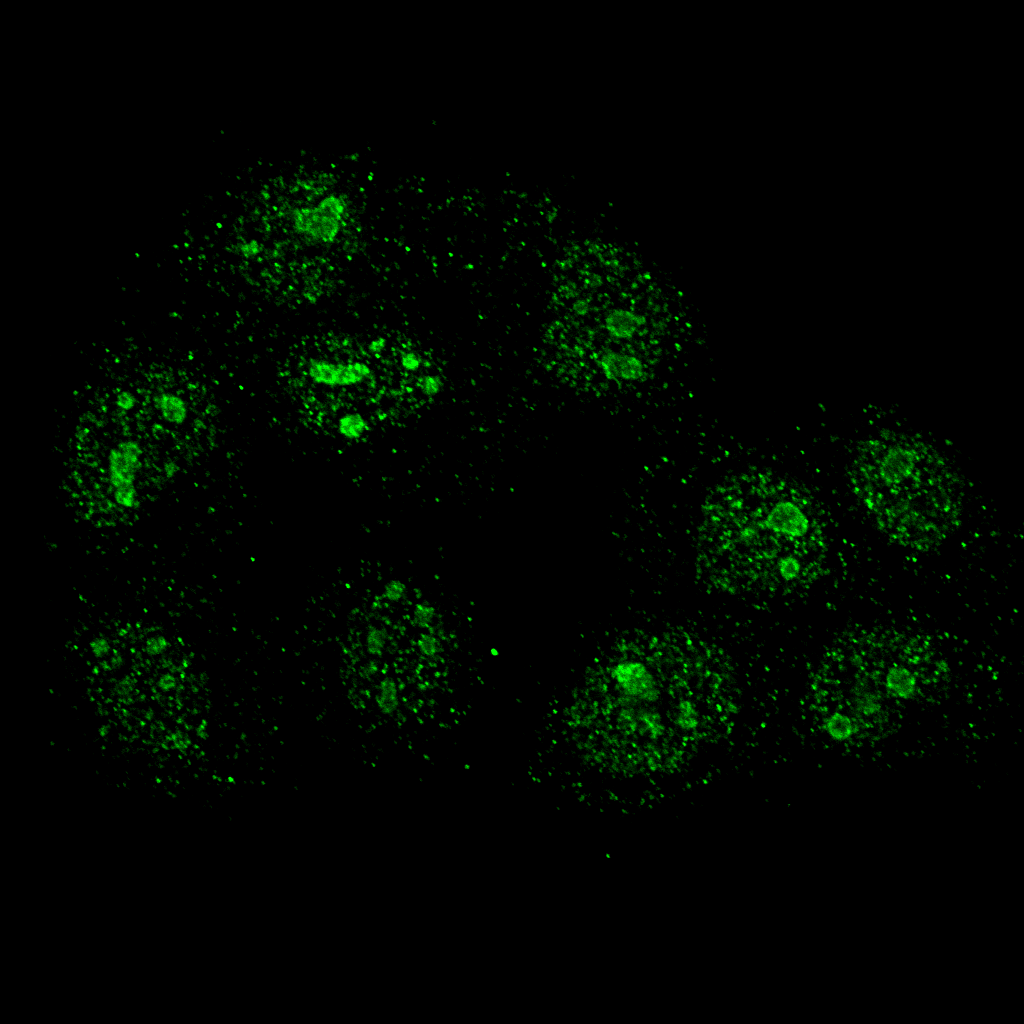

Supplement: Supplementary file 2 — Source data Fig. 1 [file 44318_2024_192_MOESM2_ESM.zip › Figure1/Figure1a/PQBP3.tif]

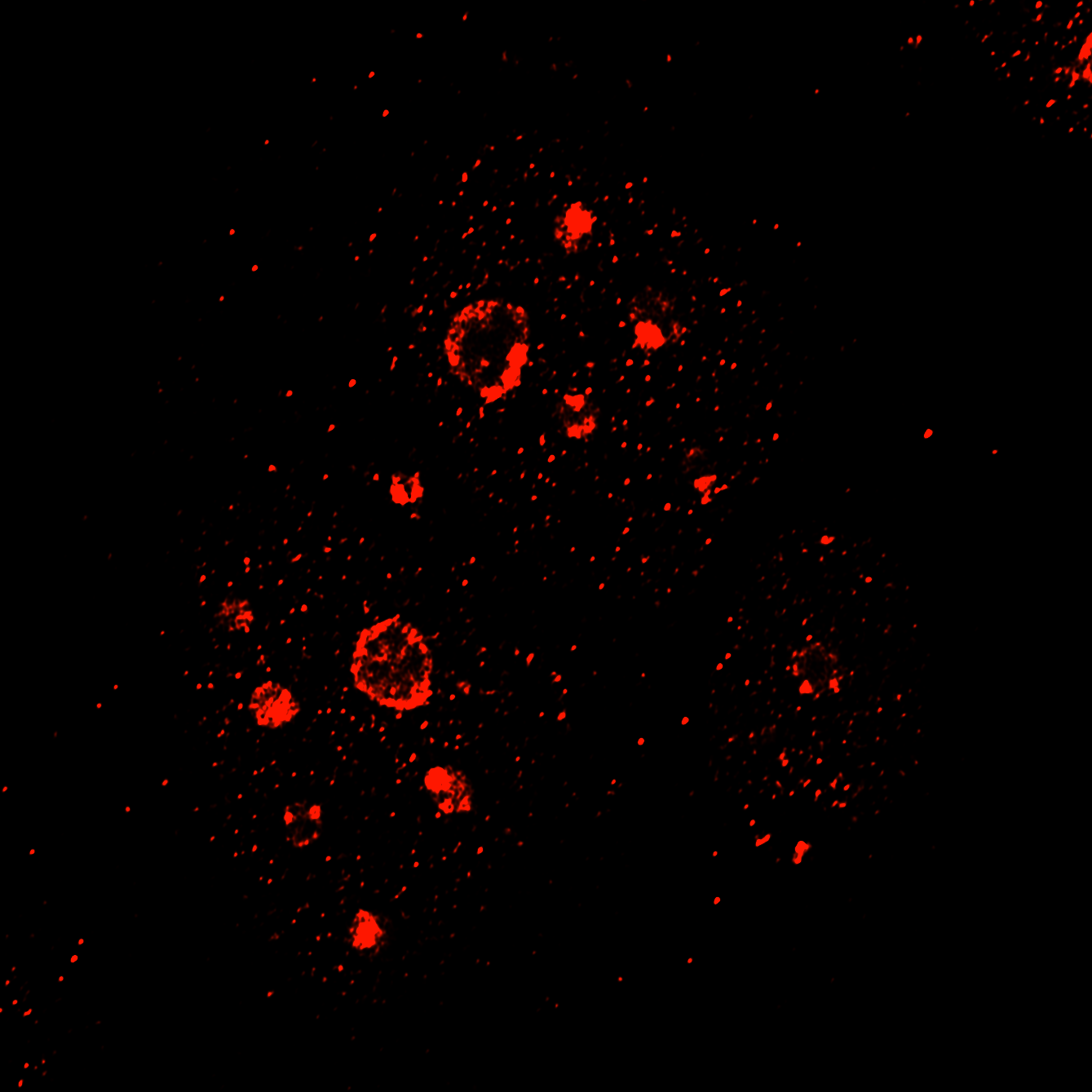

Supplement: Supplementary file 2 — Source data Fig. 1 [file 44318_2024_192_MOESM2_ESM.zip › Figure1/Figure1b/Fibrillarin.tif]

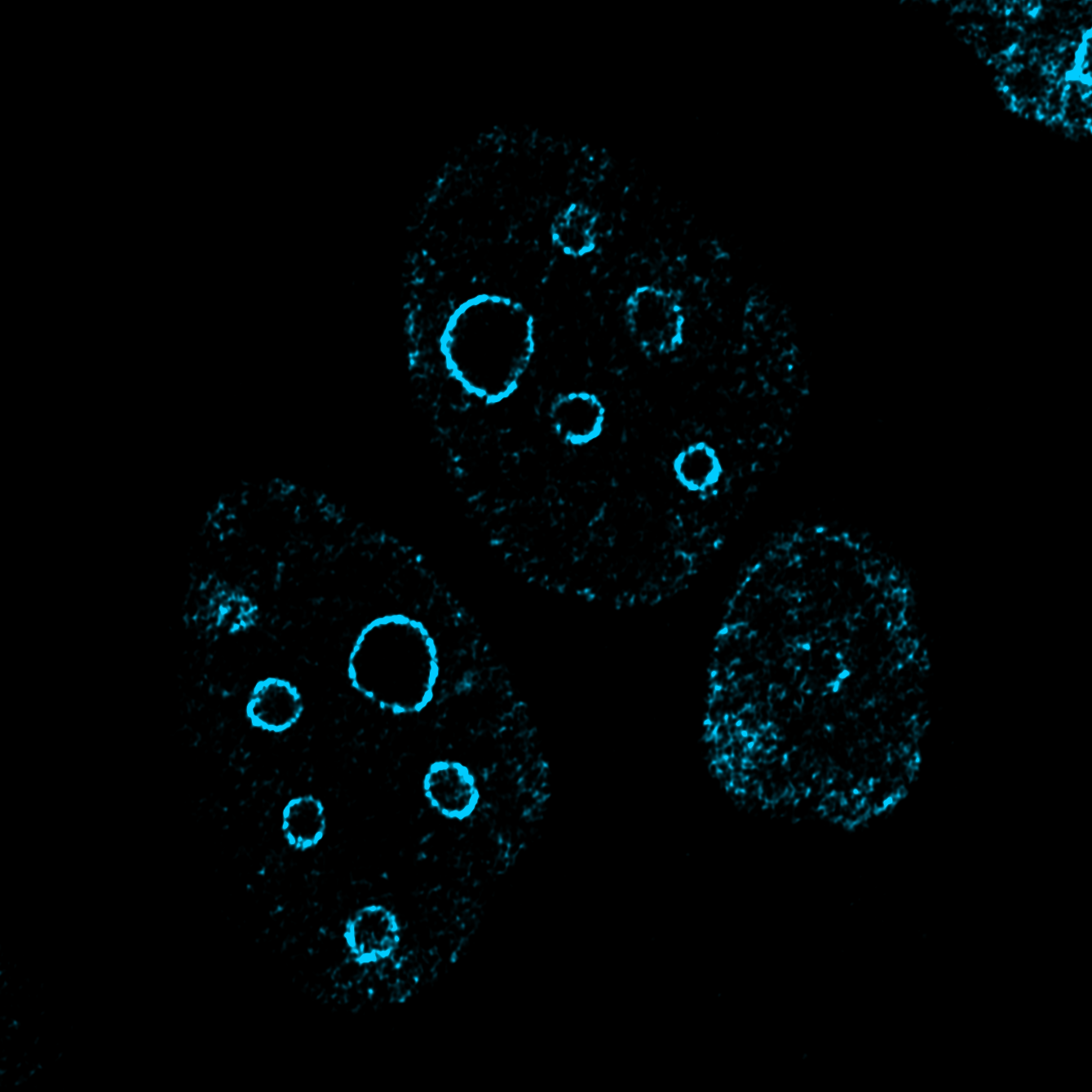

Supplement: Supplementary file 2 — Source data Fig. 1 [file 44318_2024_192_MOESM2_ESM.zip › Figure1/Figure1b/Nucleolin.tif]

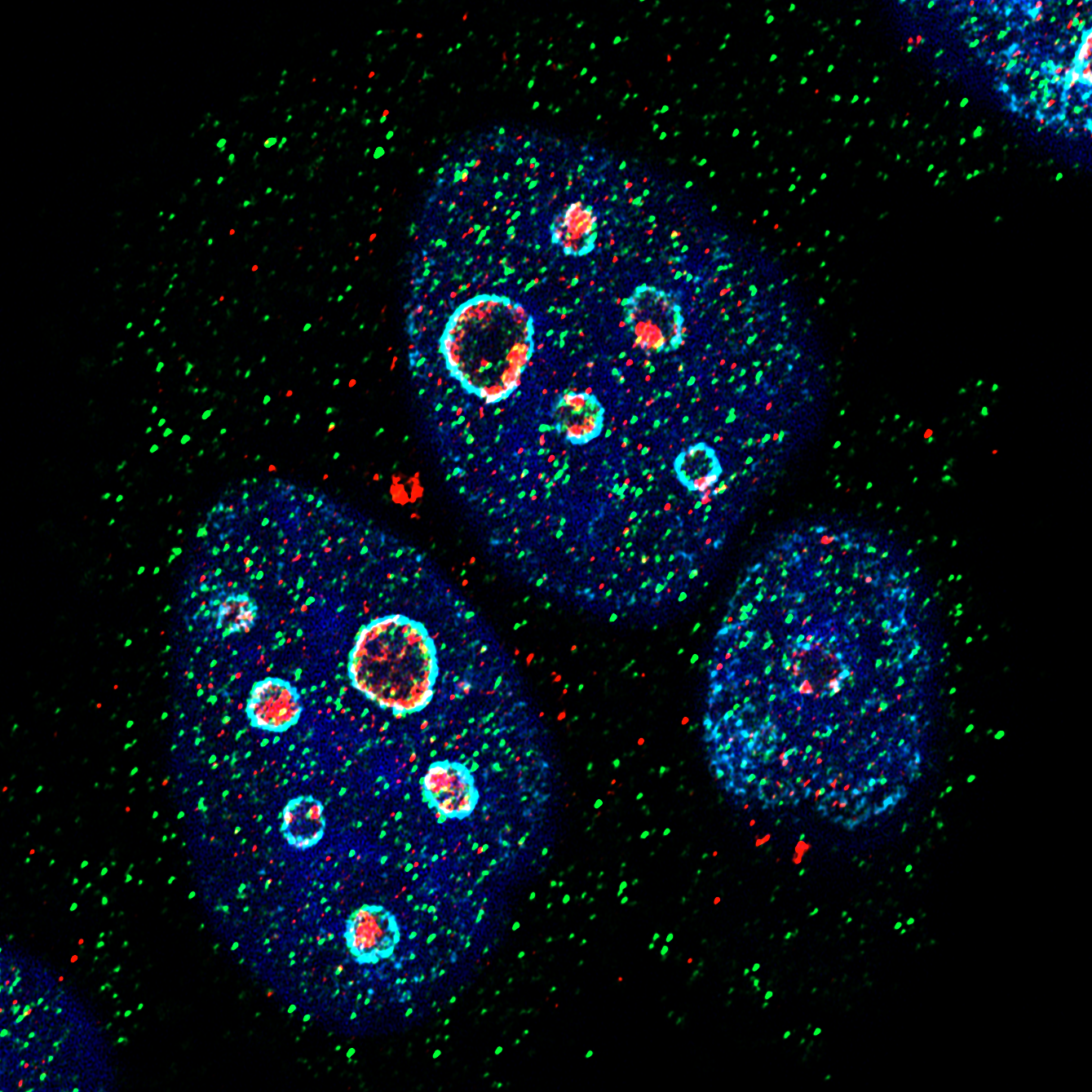

Supplement: Supplementary file 2 — Source data Fig. 1 [file 44318_2024_192_MOESM2_ESM.zip › Figure1/Figure1b/PQBP3+FIbrillarin+Nucleolin+DAPI.tif]

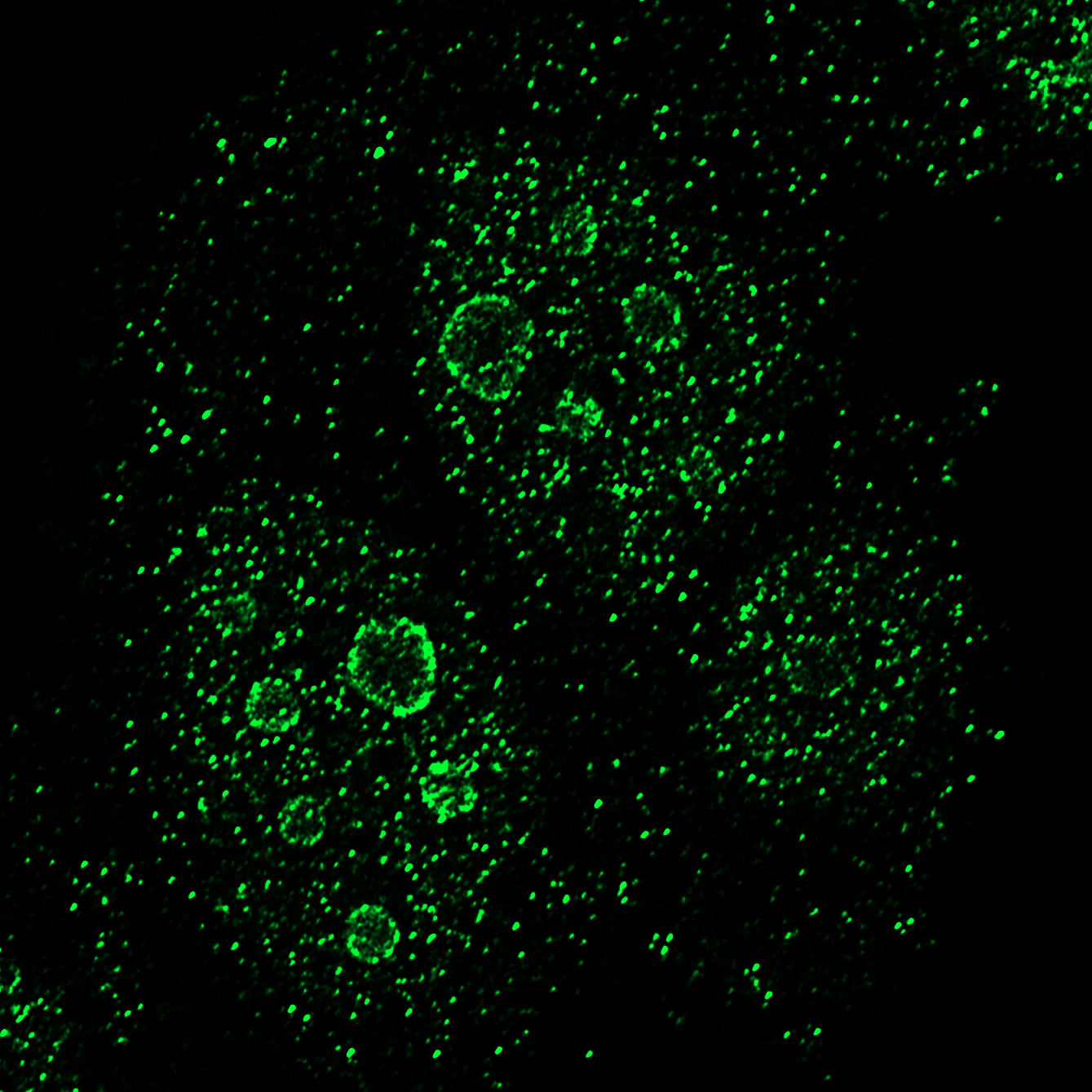

Supplement: Supplementary file 2 — Source data Fig. 1 [file 44318_2024_192_MOESM2_ESM.zip › Figure1/Figure1b/PQBP3.tif]

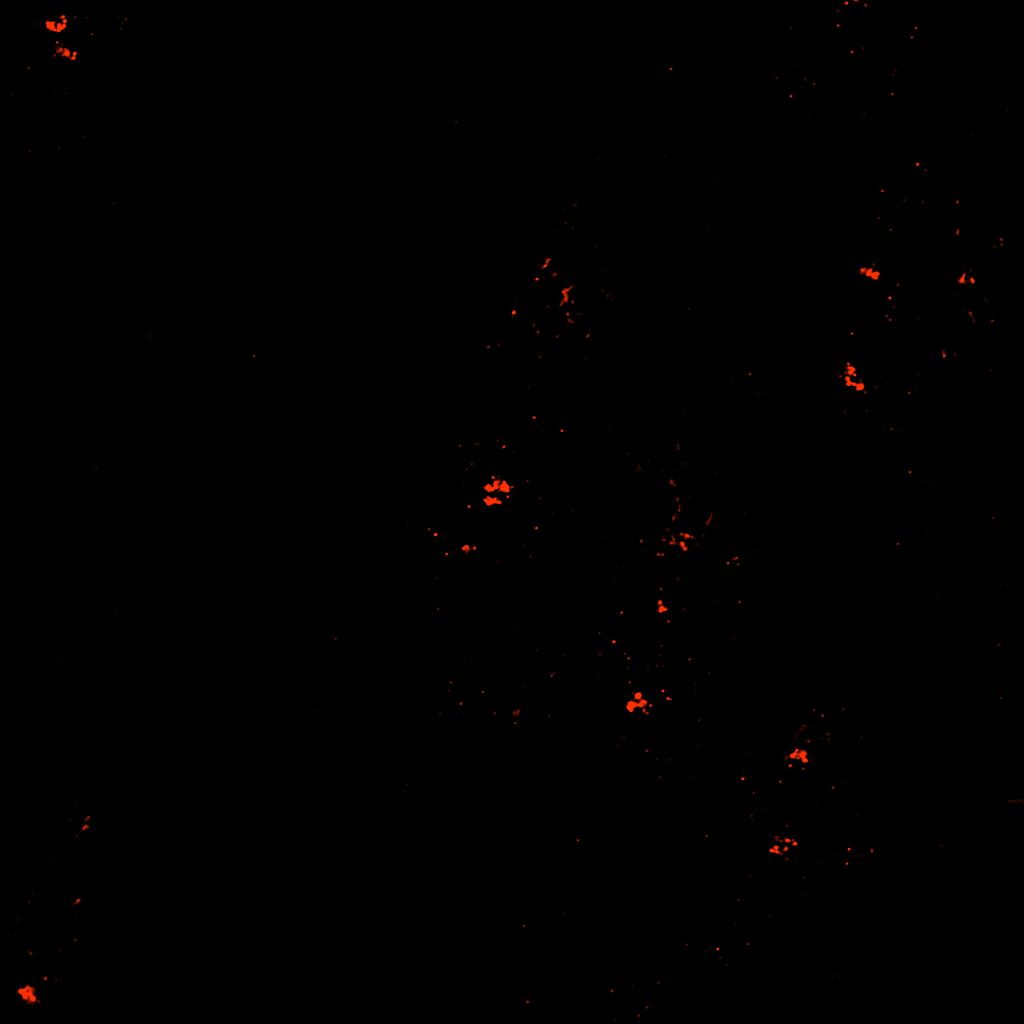

Supplement: Supplementary file 2 — Source data Fig. 1 [file 44318_2024_192_MOESM2_ESM.zip › Figure1/Figure1c/Fibrillarin.tif]

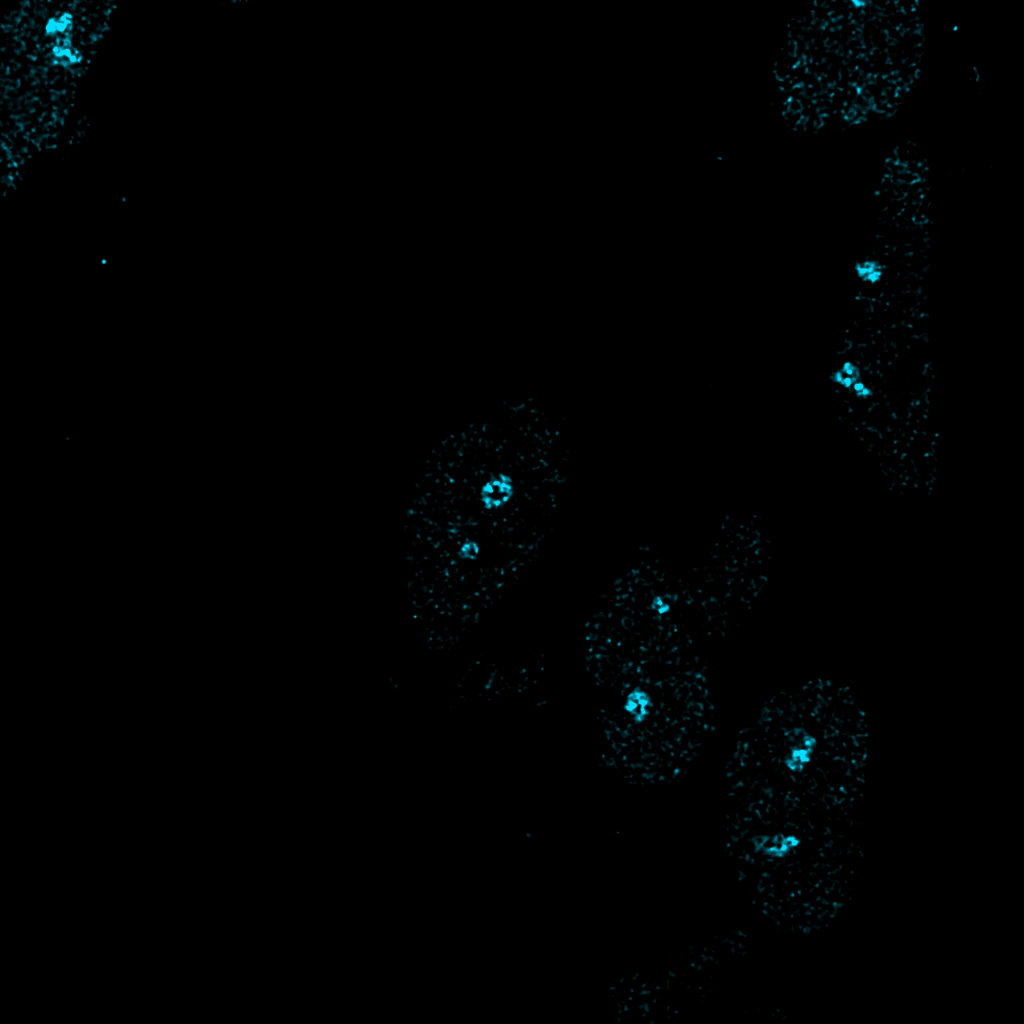

Supplement: Supplementary file 2 — Source data Fig. 1 [file 44318_2024_192_MOESM2_ESM.zip › Figure1/Figure1c/Nucleolin.tif]

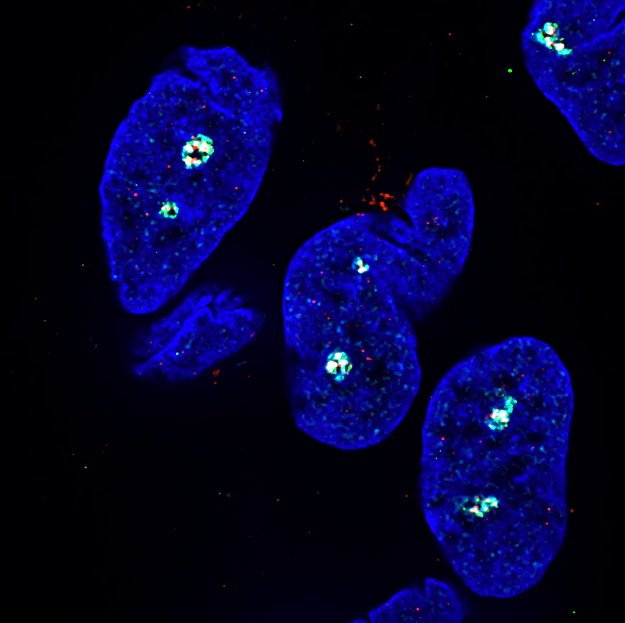

Supplement: Supplementary file 2 — Source data Fig. 1 [file 44318_2024_192_MOESM2_ESM.zip › Figure1/Figure1c/PQBP3+Fibrillarin+Nucleolin+DAPI.tif]

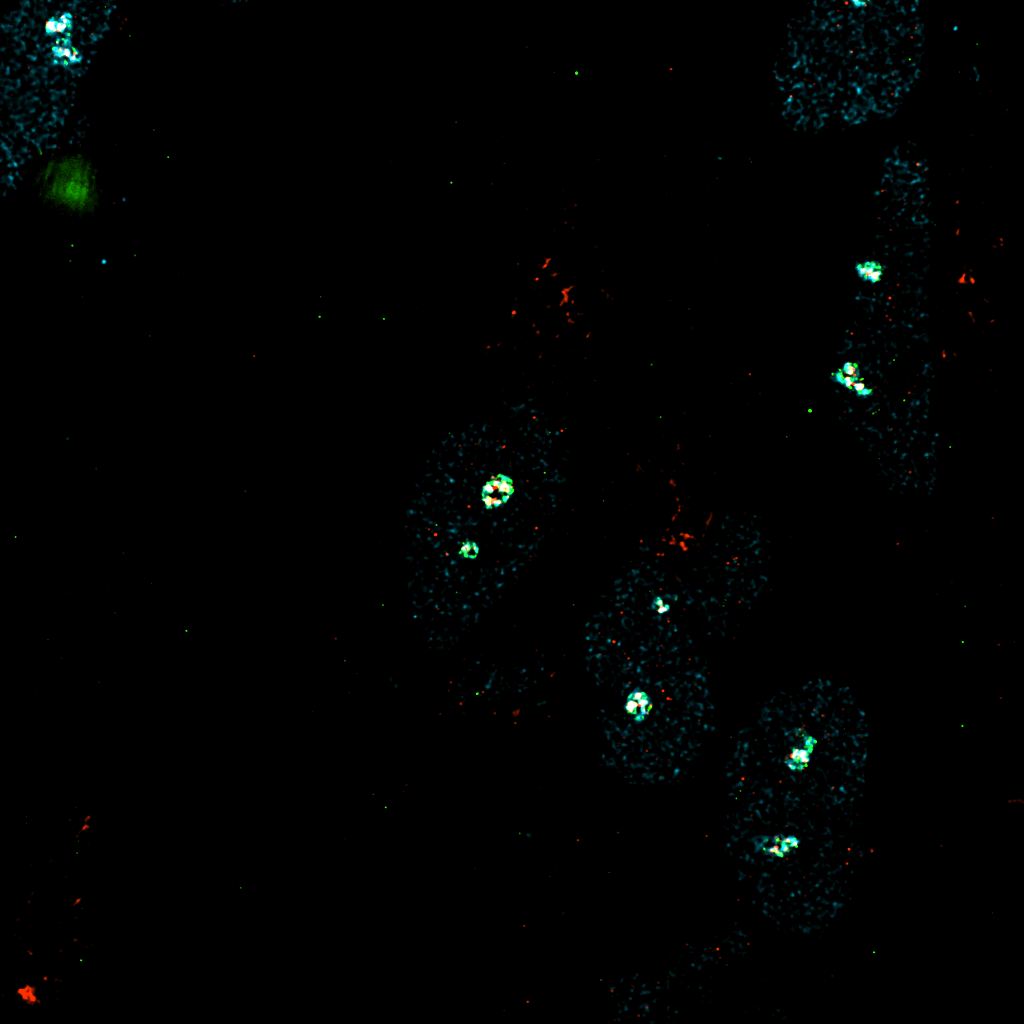

Supplement: Supplementary file 2 — Source data Fig. 1 [file 44318_2024_192_MOESM2_ESM.zip › Figure1/Figure1c/PQBP3+Fibrillarin+Nucleolin.tif]

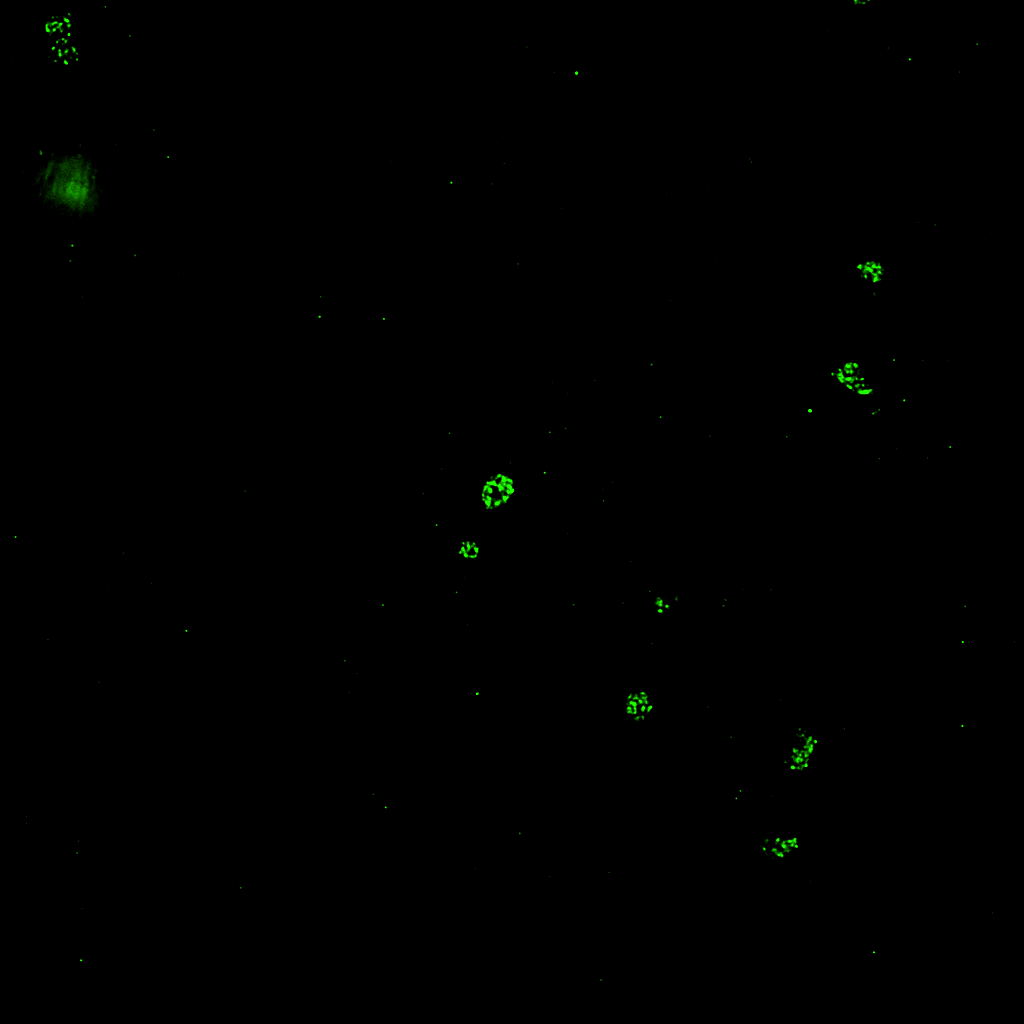

Supplement: Supplementary file 2 — Source data Fig. 1 [file 44318_2024_192_MOESM2_ESM.zip › Figure1/Figure1c/PQBP3.tif]

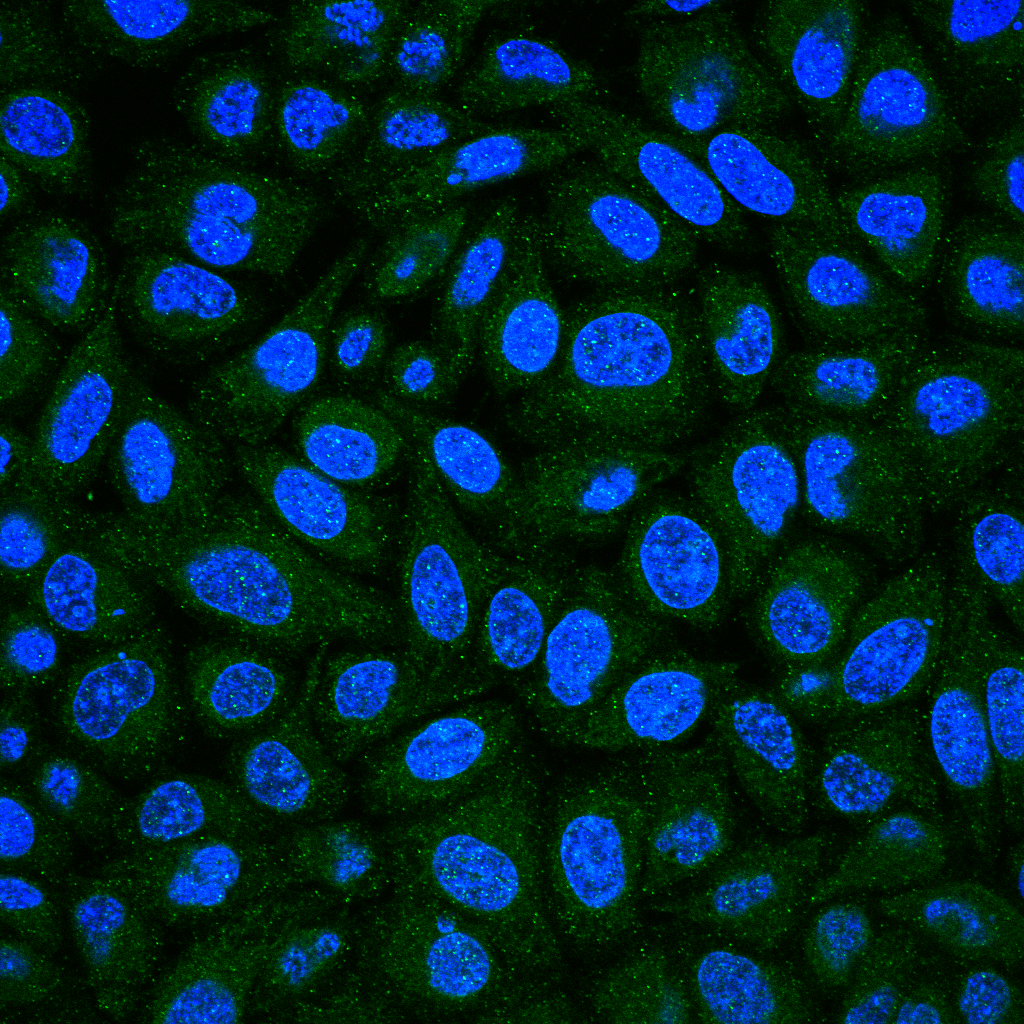

Supplement: Supplementary file 3 — Source data Fig. 2 [file 44318_2024_192_MOESM3_ESM.zip › Figure2/Figure2a/High.tif]

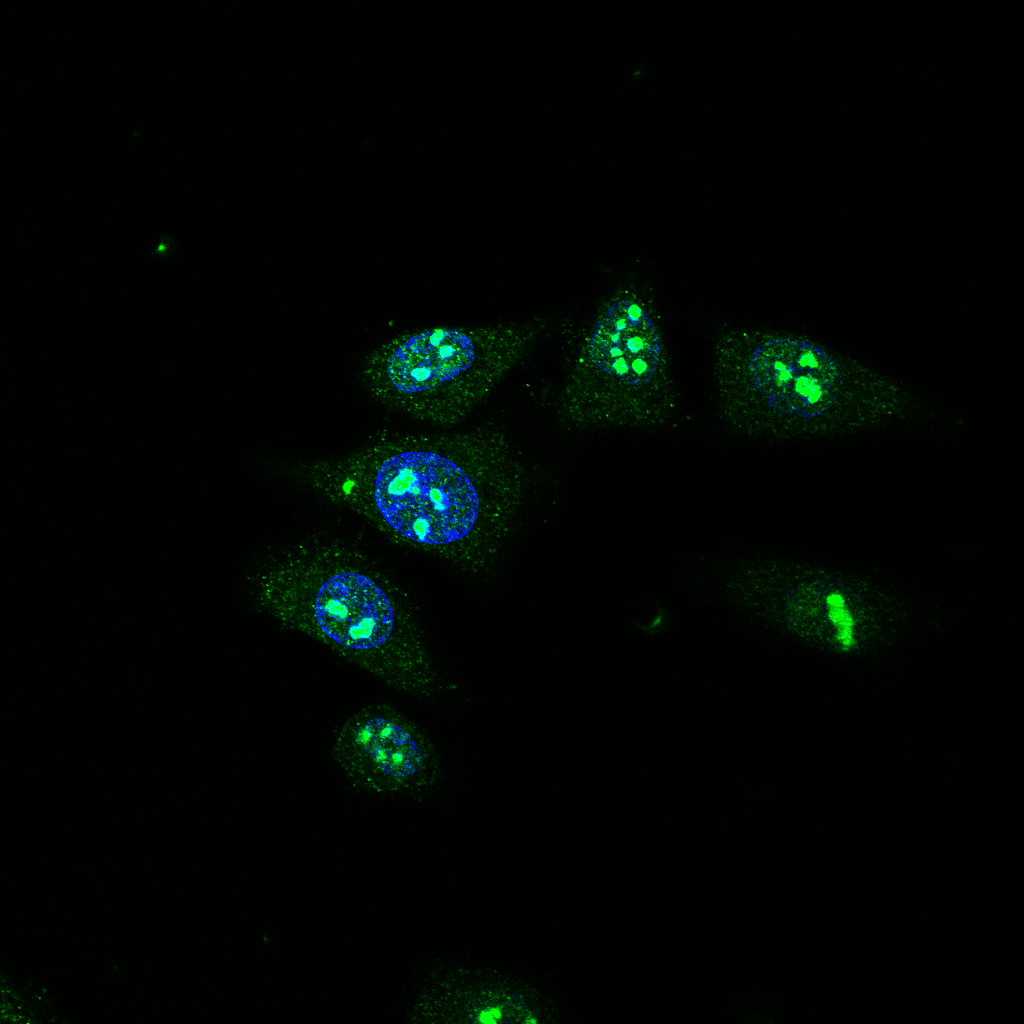

Supplement: Supplementary file 3 — Source data Fig. 2 [file 44318_2024_192_MOESM3_ESM.zip › Figure2/Figure2a/Low.tif]

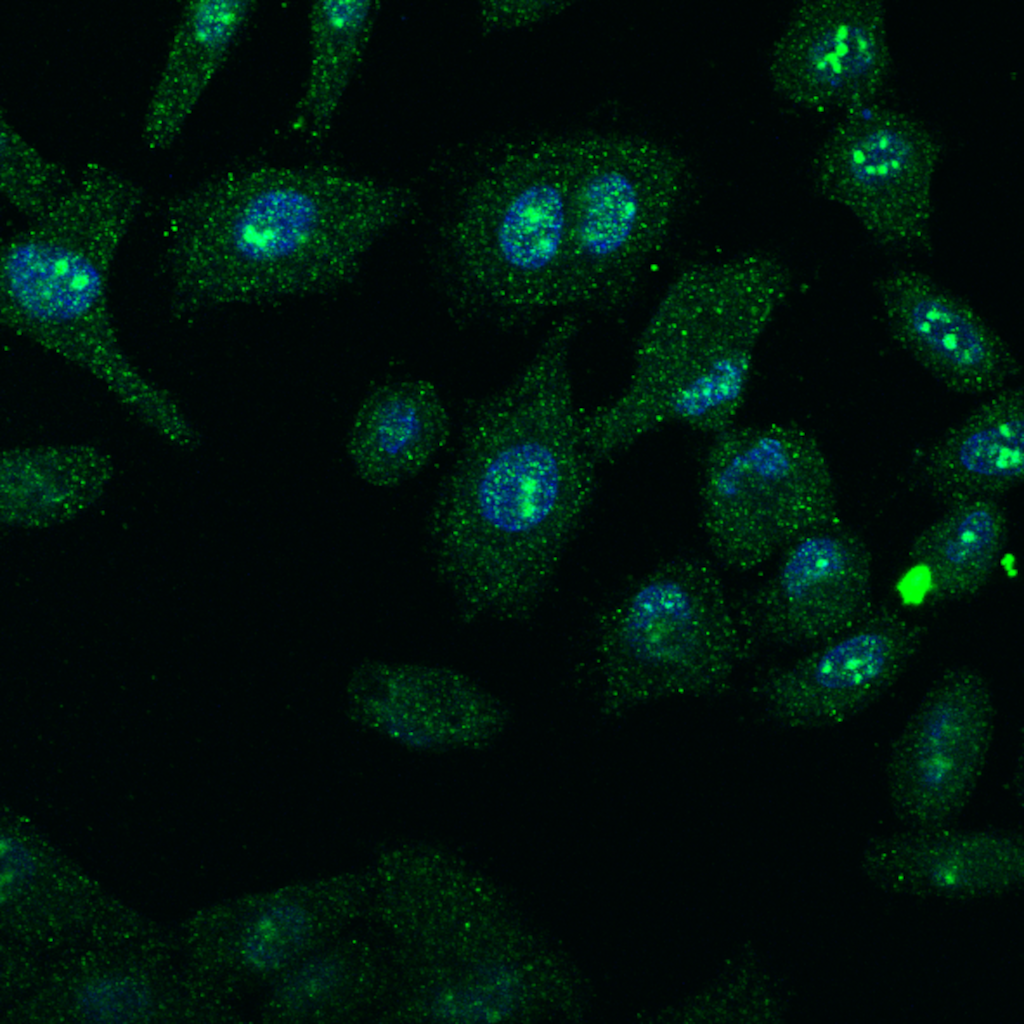

Supplement: Supplementary file 3 — Source data Fig. 2 [file 44318_2024_192_MOESM3_ESM.zip › Figure2/Figure2a/Middle.tif]

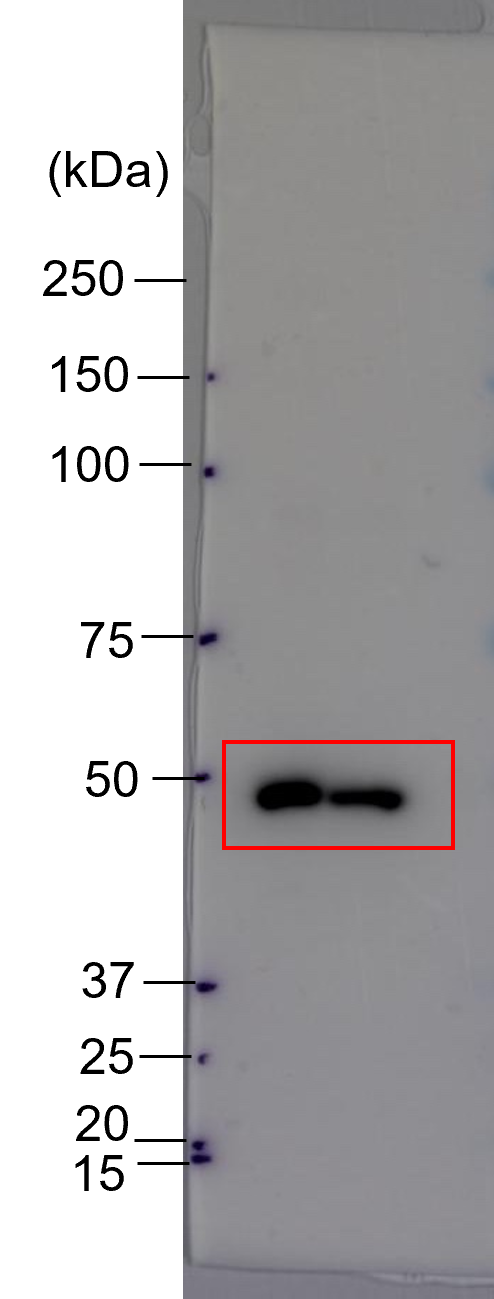

Supplement: Supplementary file 3 — Source data Fig. 2 [file 44318_2024_192_MOESM3_ESM.zip › Figure2/Figure2c/Cytoplasmic fraction b-Tub.tif]

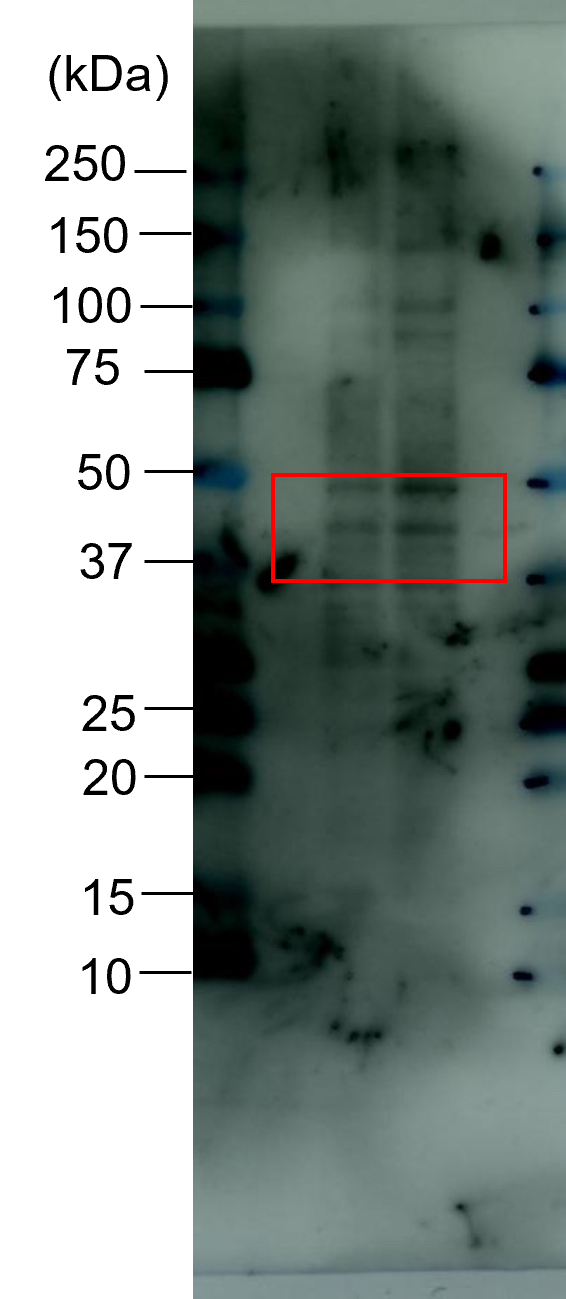

Supplement: Supplementary file 3 — Source data Fig. 2 [file 44318_2024_192_MOESM3_ESM.zip › Figure2/Figure2c/Cytoplasmic fraction PQBP3.tif]

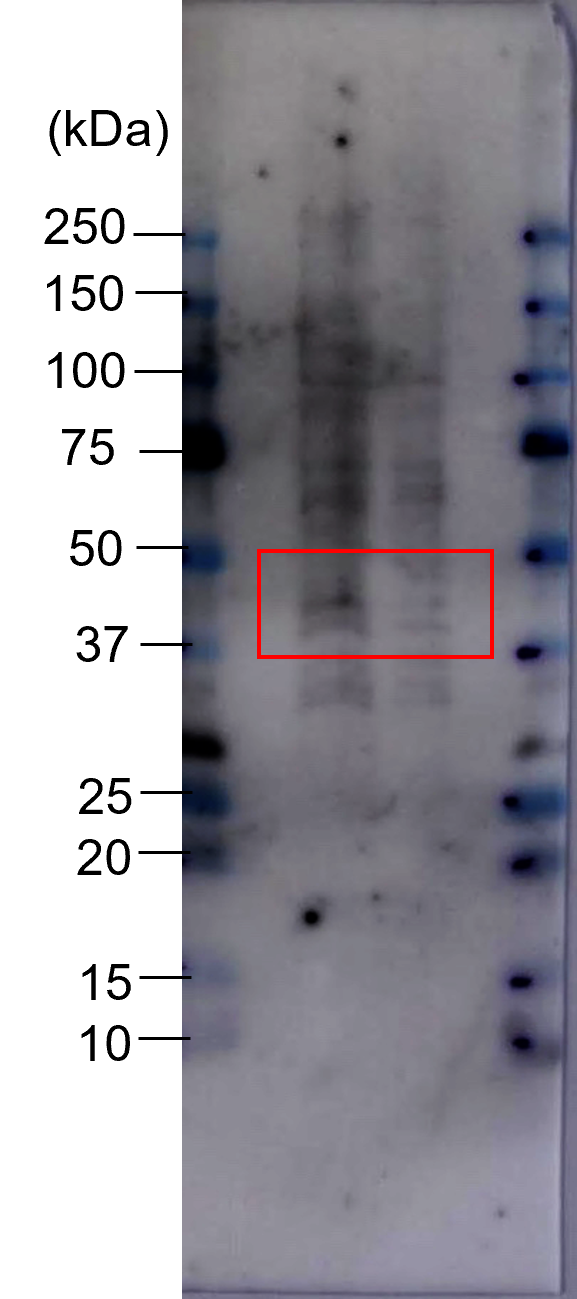

Supplement: Supplementary file 3 — Source data Fig. 2 [file 44318_2024_192_MOESM3_ESM.zip › Figure2/Figure2c/Nuclear fraction PQBP3.tif]

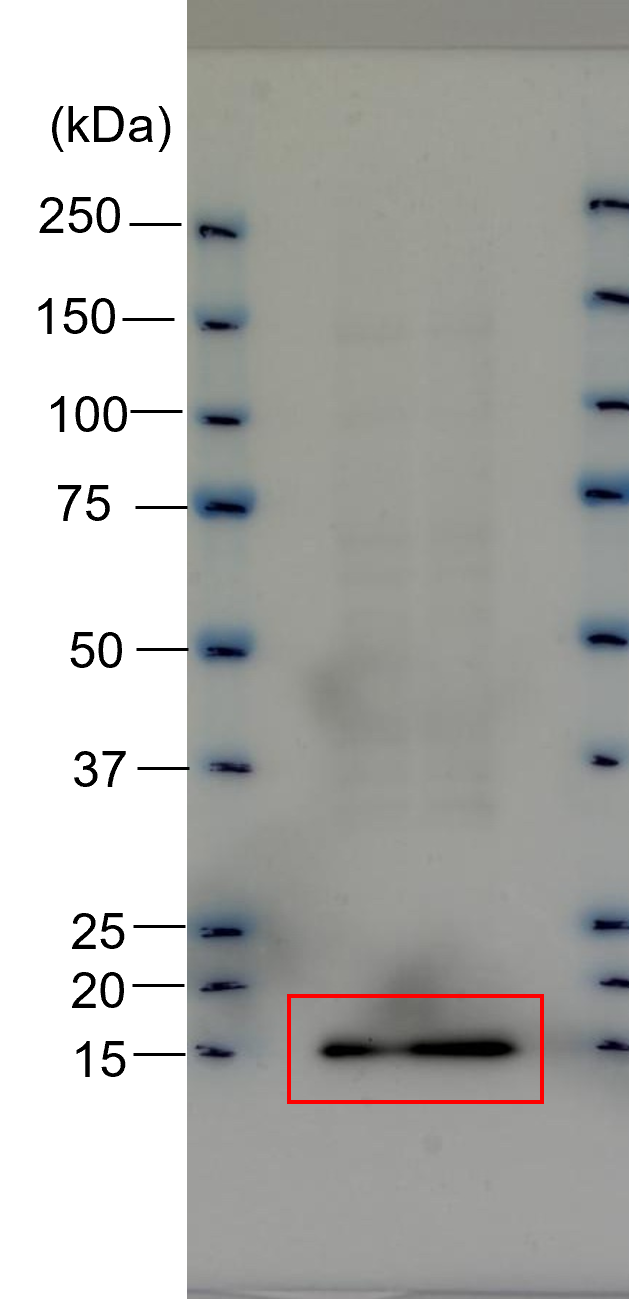

Supplement: Supplementary file 3 — Source data Fig. 2 [file 44318_2024_192_MOESM3_ESM.zip › Figure2/Figure2c/Nuclear fraction histone.tif]

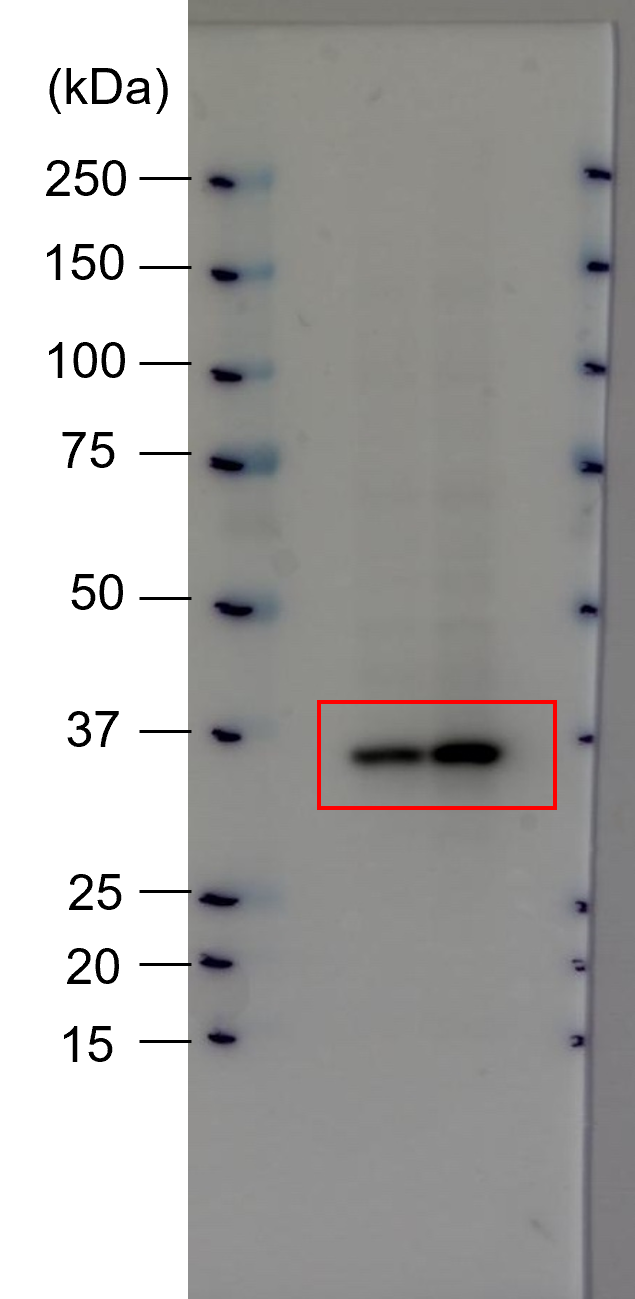

Supplement: Supplementary file 3 — Source data Fig. 2 [file 44318_2024_192_MOESM3_ESM.zip › Figure2/Figure2c/Total cell extract GAPDH.tif]

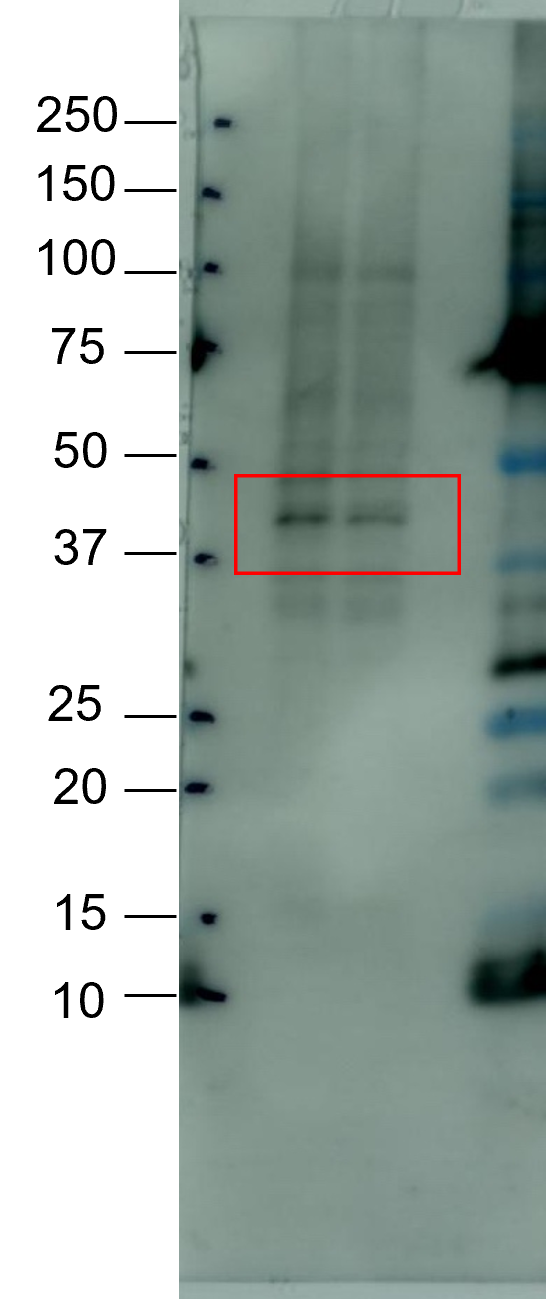

Supplement: Supplementary file 3 — Source data Fig. 2 [file 44318_2024_192_MOESM3_ESM.zip › Figure2/Figure2c/Total cell extract PQBP3.tif]

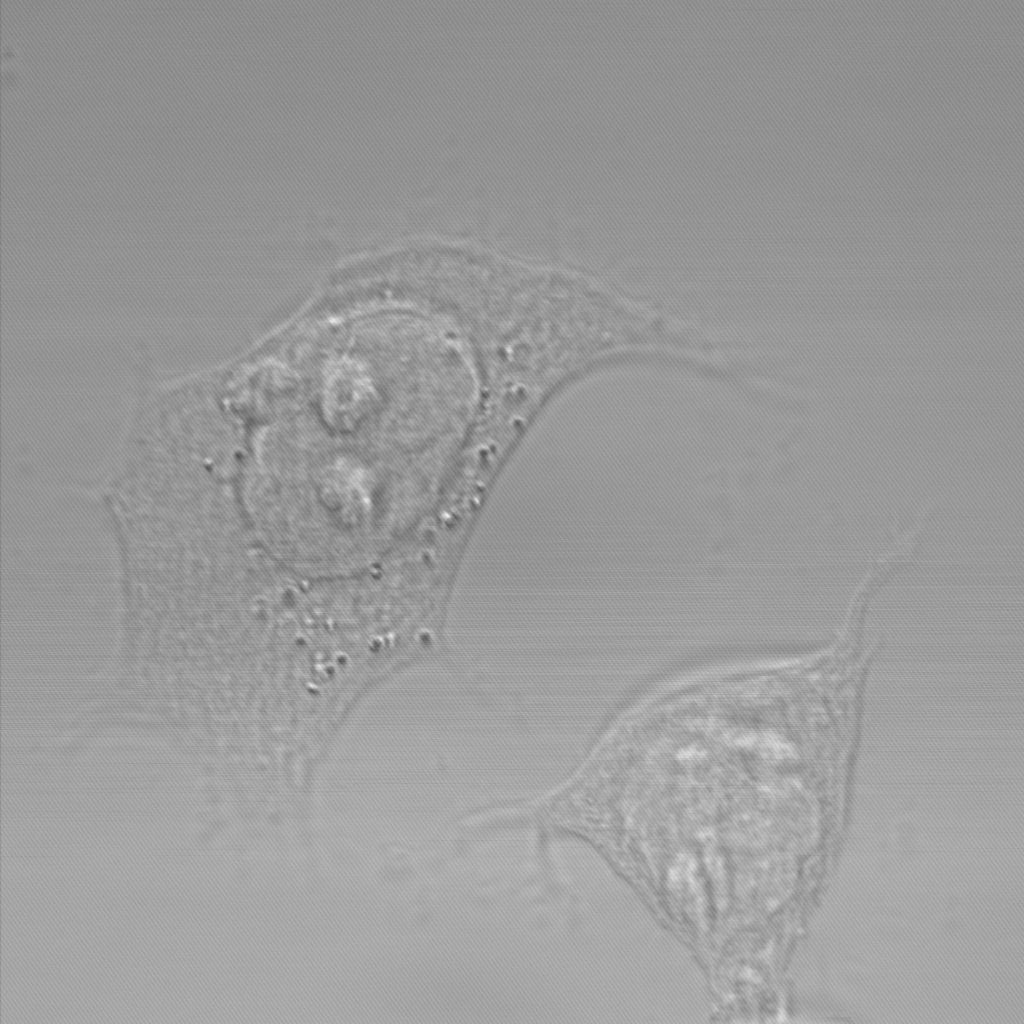

Supplement: Supplementary file 3 — Source data Fig. 2 [file 44318_2024_192_MOESM3_ESM.zip › Figure2/Figure2d/Lower images/bright image.tif]

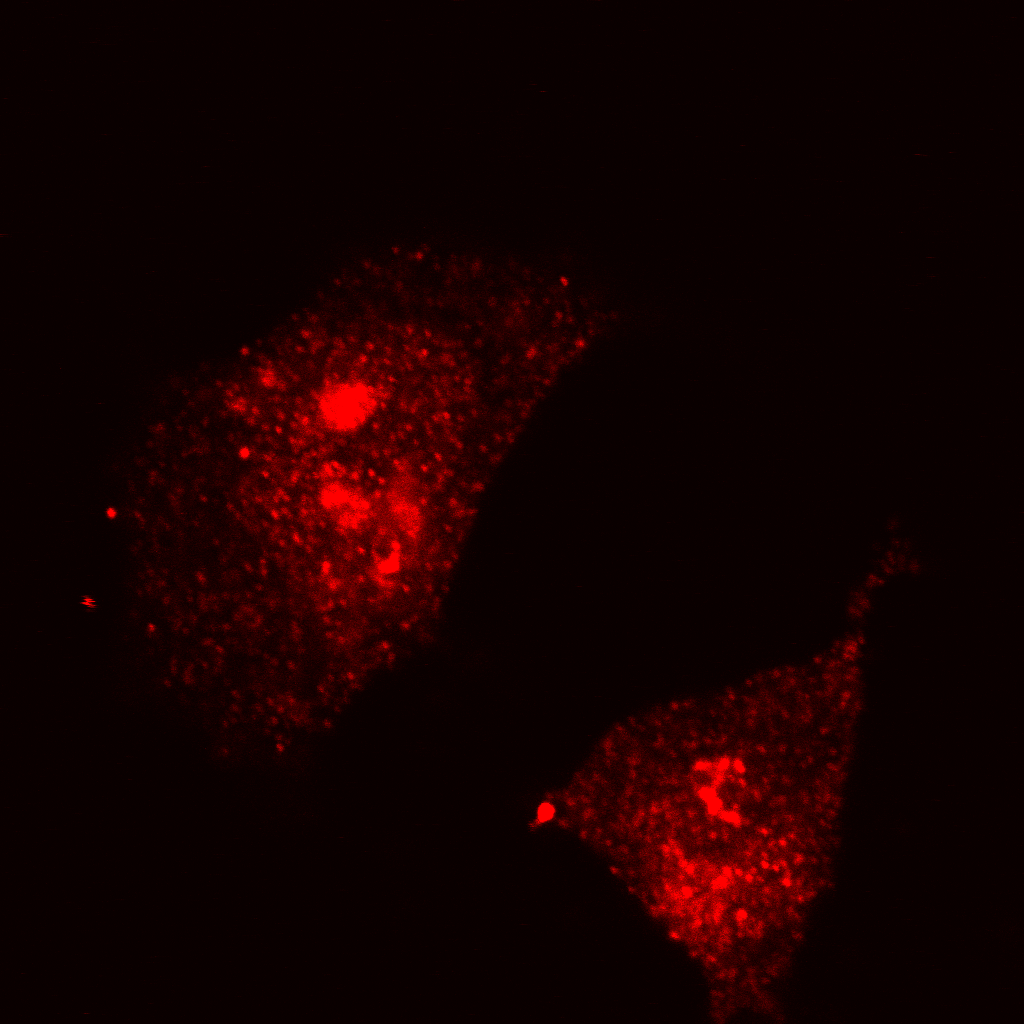

Supplement: Supplementary file 3 — Source data Fig. 2 [file 44318_2024_192_MOESM3_ESM.zip › Figure2/Figure2d/Lower images/Fibrillarin.tif]

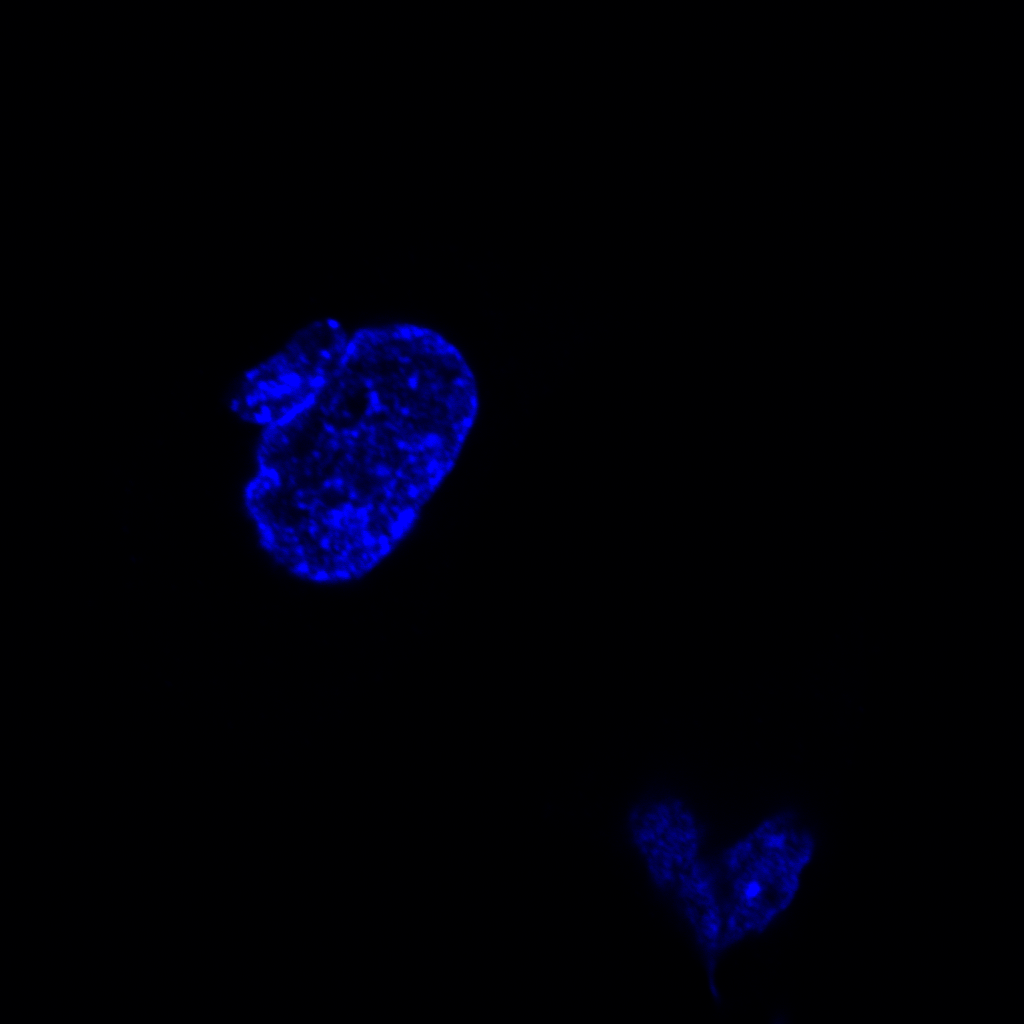

Supplement: Supplementary file 3 — Source data Fig. 2 [file 44318_2024_192_MOESM3_ESM.zip › Figure2/Figure2d/Lower images/Hoechst 33342.tif]

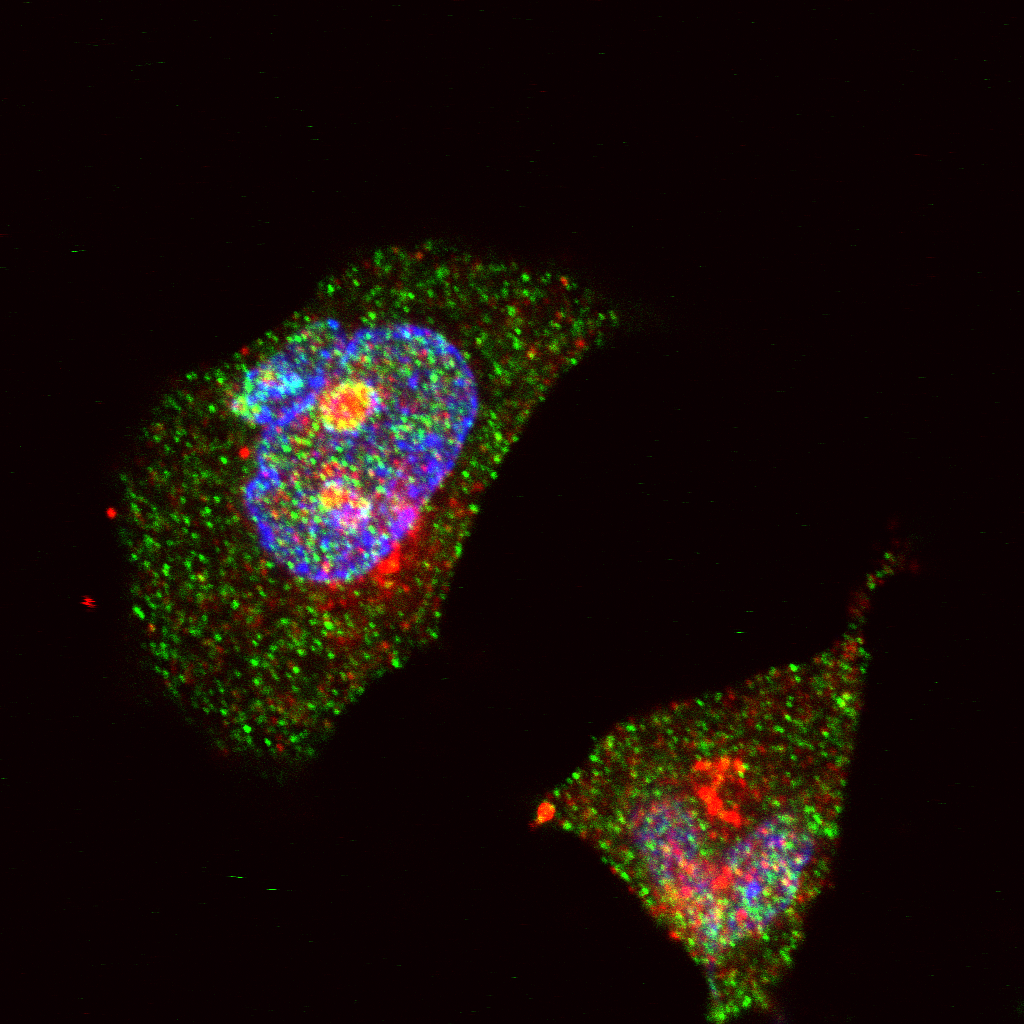

Supplement: Supplementary file 3 — Source data Fig. 2 [file 44318_2024_192_MOESM3_ESM.zip › Figure2/Figure2d/Lower images/merge.tif]

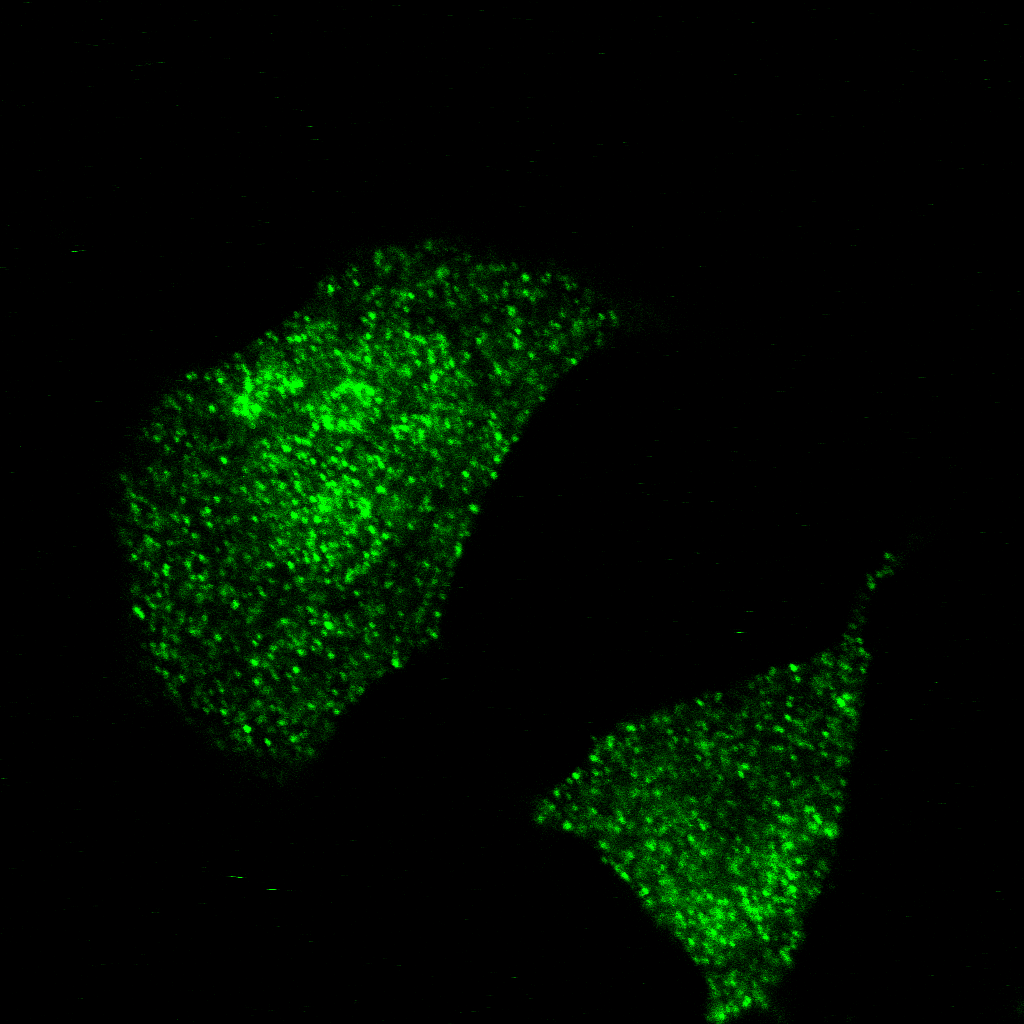

Supplement: Supplementary file 3 — Source data Fig. 2 [file 44318_2024_192_MOESM3_ESM.zip › Figure2/Figure2d/Lower images/PQBP3.tif]

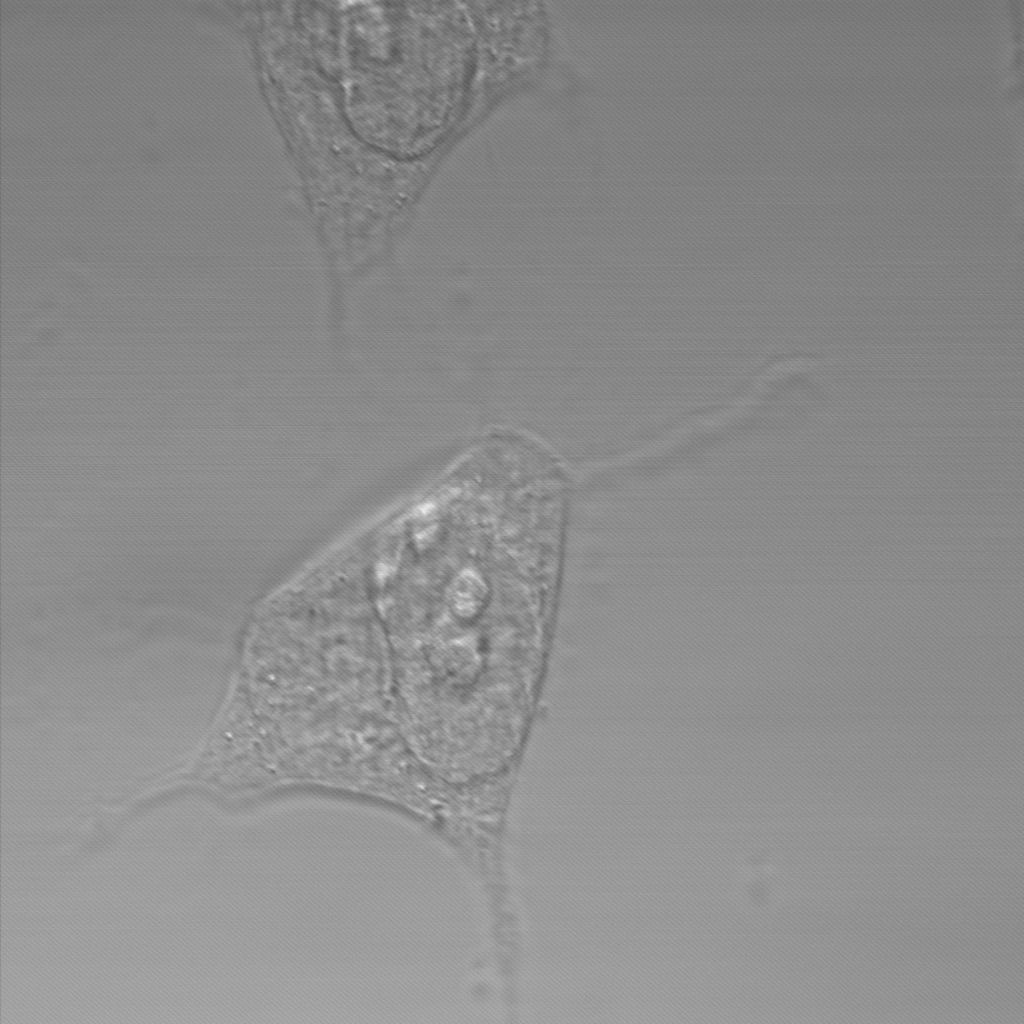

Supplement: Supplementary file 3 — Source data Fig. 2 [file 44318_2024_192_MOESM3_ESM.zip › Figure2/Figure2d/Upper images/bright image.tif]

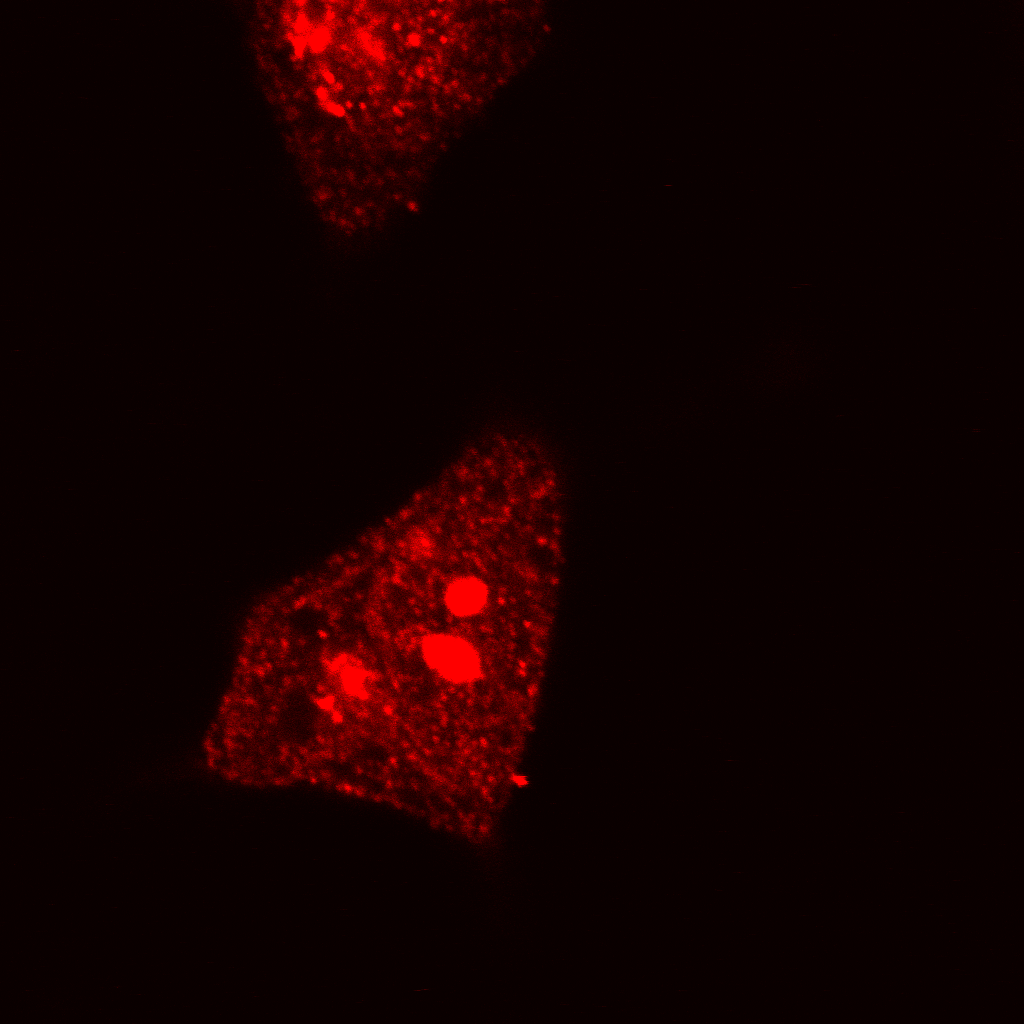

Supplement: Supplementary file 3 — Source data Fig. 2 [file 44318_2024_192_MOESM3_ESM.zip › Figure2/Figure2d/Upper images/Fibrillarin.tif]

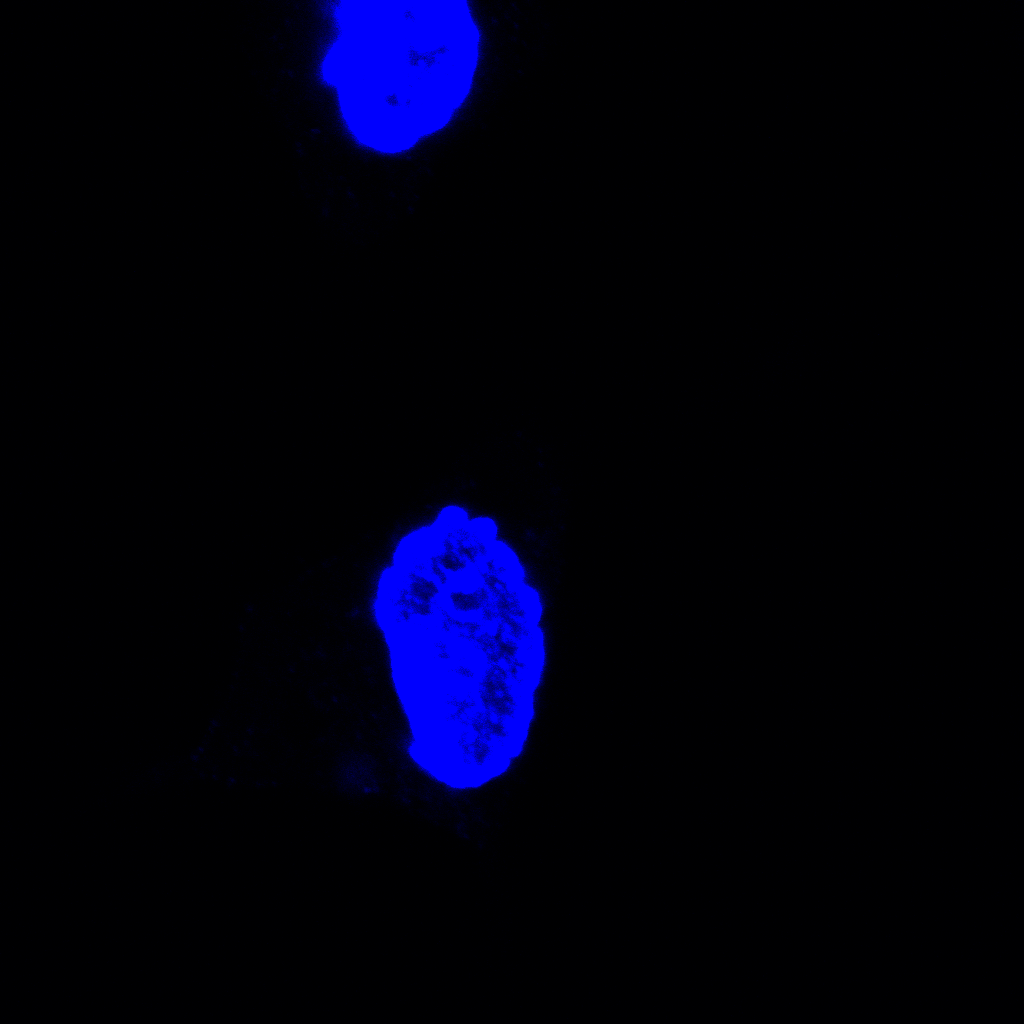

Supplement: Supplementary file 3 — Source data Fig. 2 [file 44318_2024_192_MOESM3_ESM.zip › Figure2/Figure2d/Upper images/Hoechst 33342.tif]

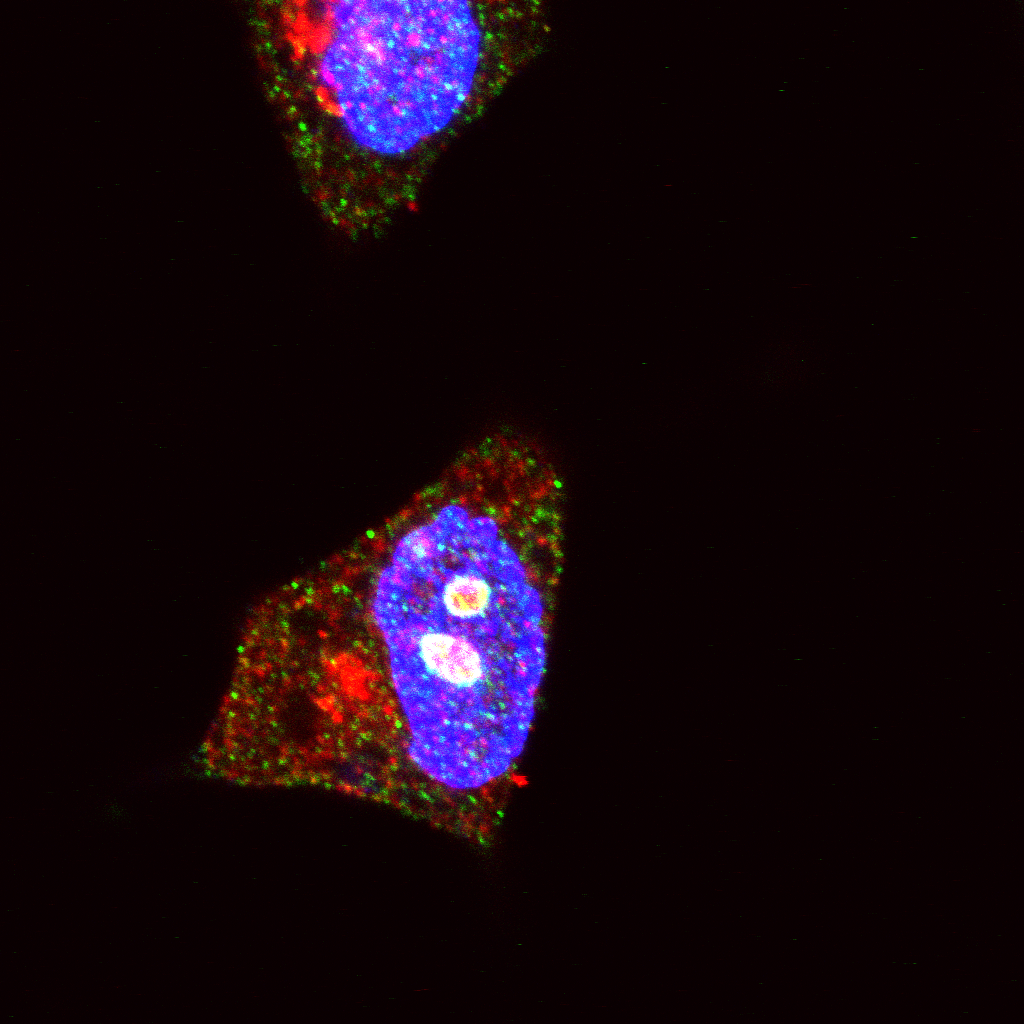

Supplement: Supplementary file 3 — Source data Fig. 2 [file 44318_2024_192_MOESM3_ESM.zip › Figure2/Figure2d/Upper images/merge.tif]

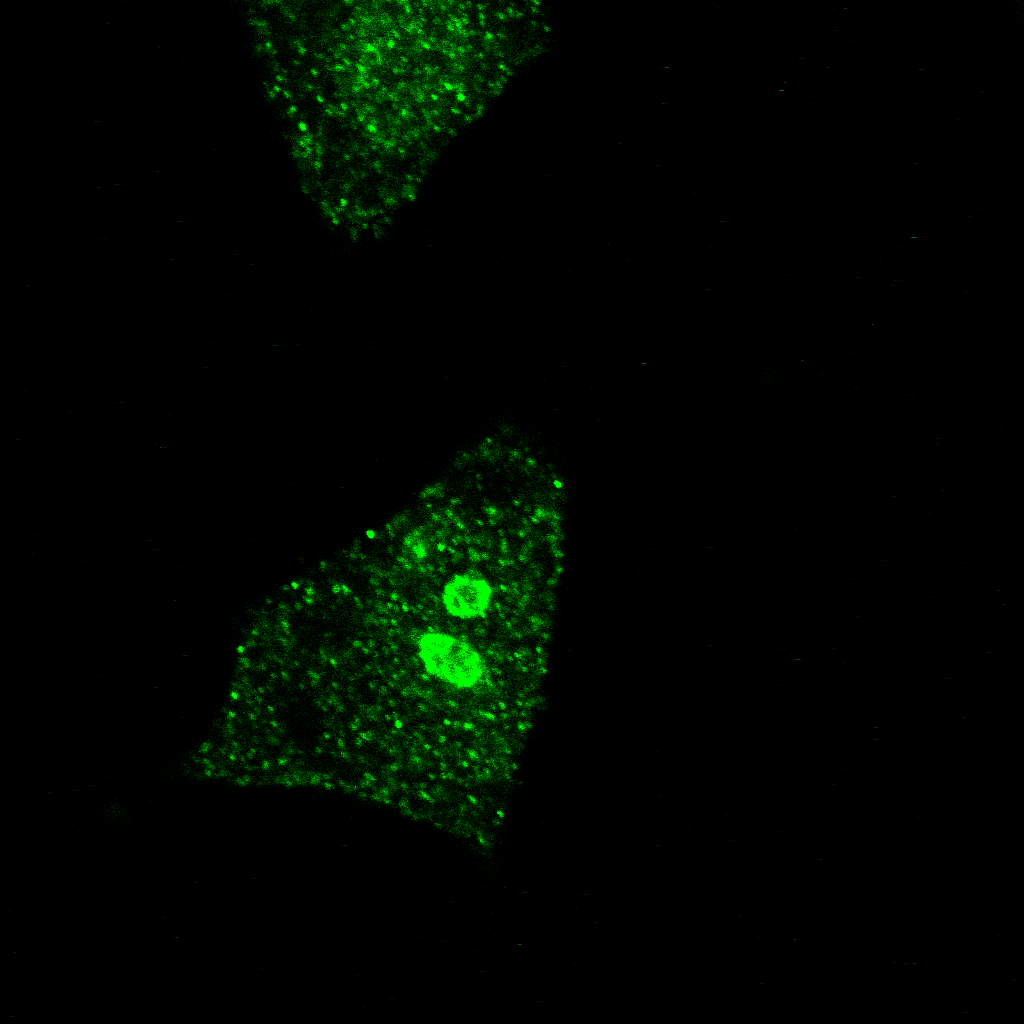

Supplement: Supplementary file 3 — Source data Fig. 2 [file 44318_2024_192_MOESM3_ESM.zip › Figure2/Figure2d/Upper images/PQBP3.tif]

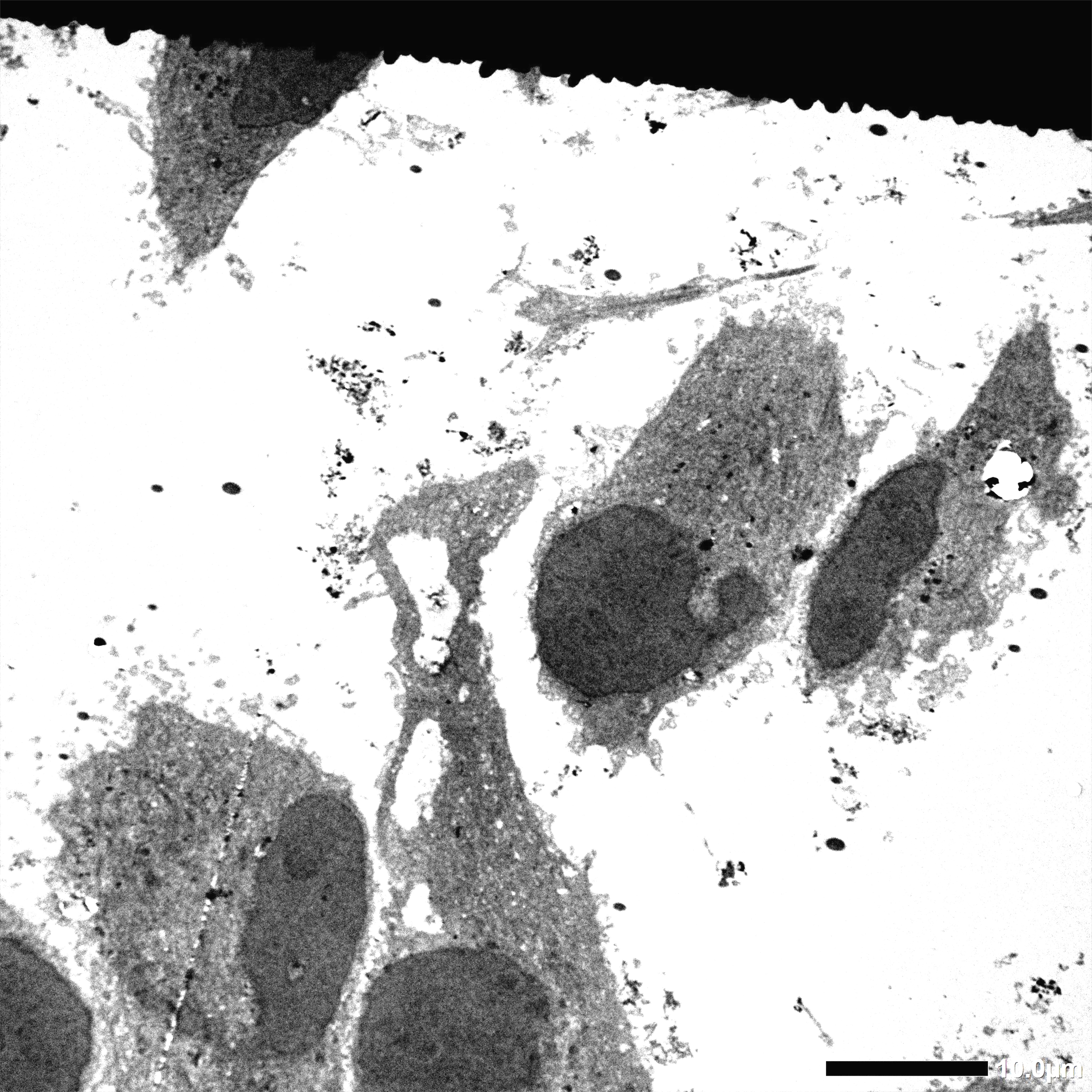

Supplement: Supplementary file 3 — Source data Fig. 2 [file 44318_2024_192_MOESM3_ESM.zip › Figure2/Figure2e/#1_left.jpeg]

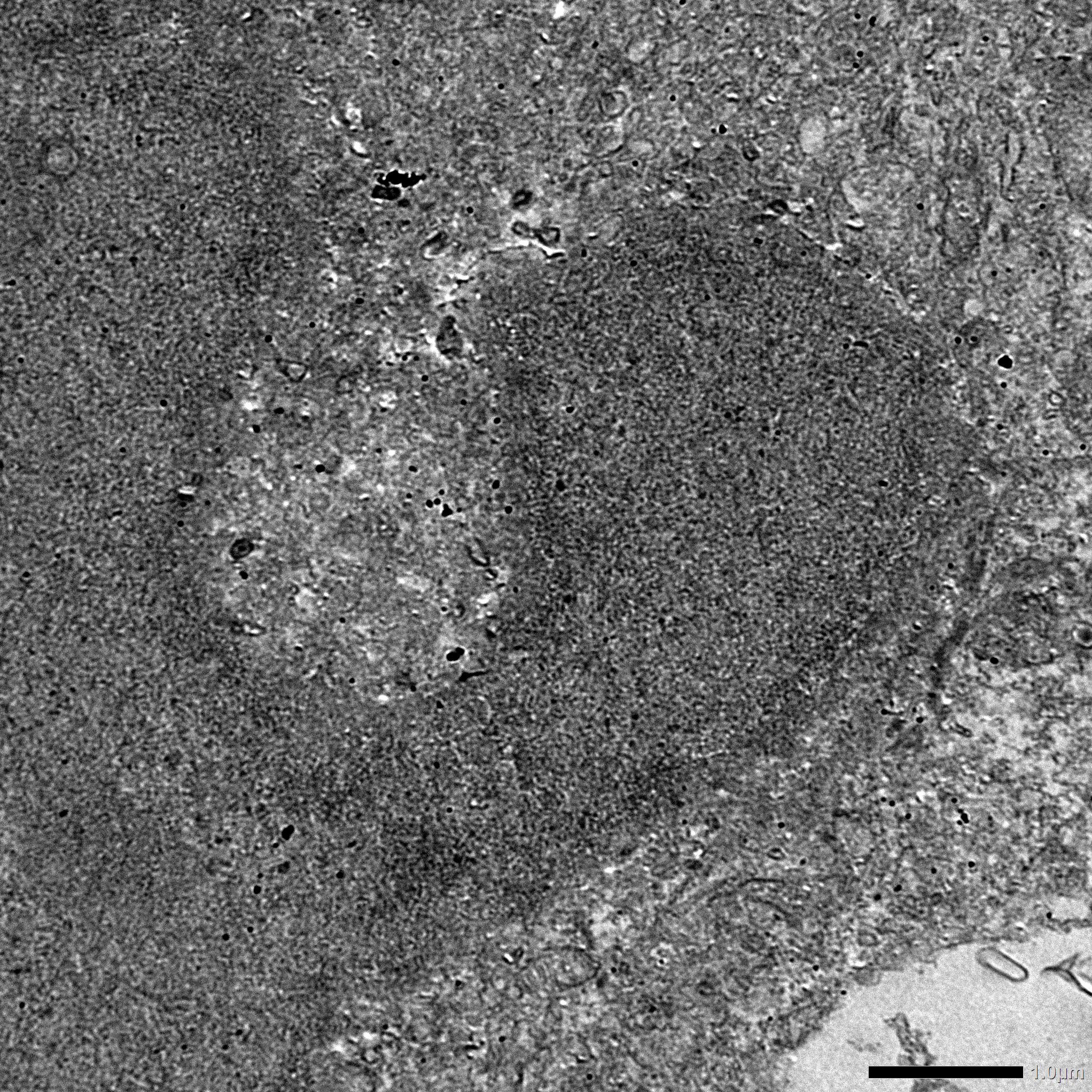

Supplement: Supplementary file 3 — Source data Fig. 2 [file 44318_2024_192_MOESM3_ESM.zip › Figure2/Figure2e/#1_right.jpg]

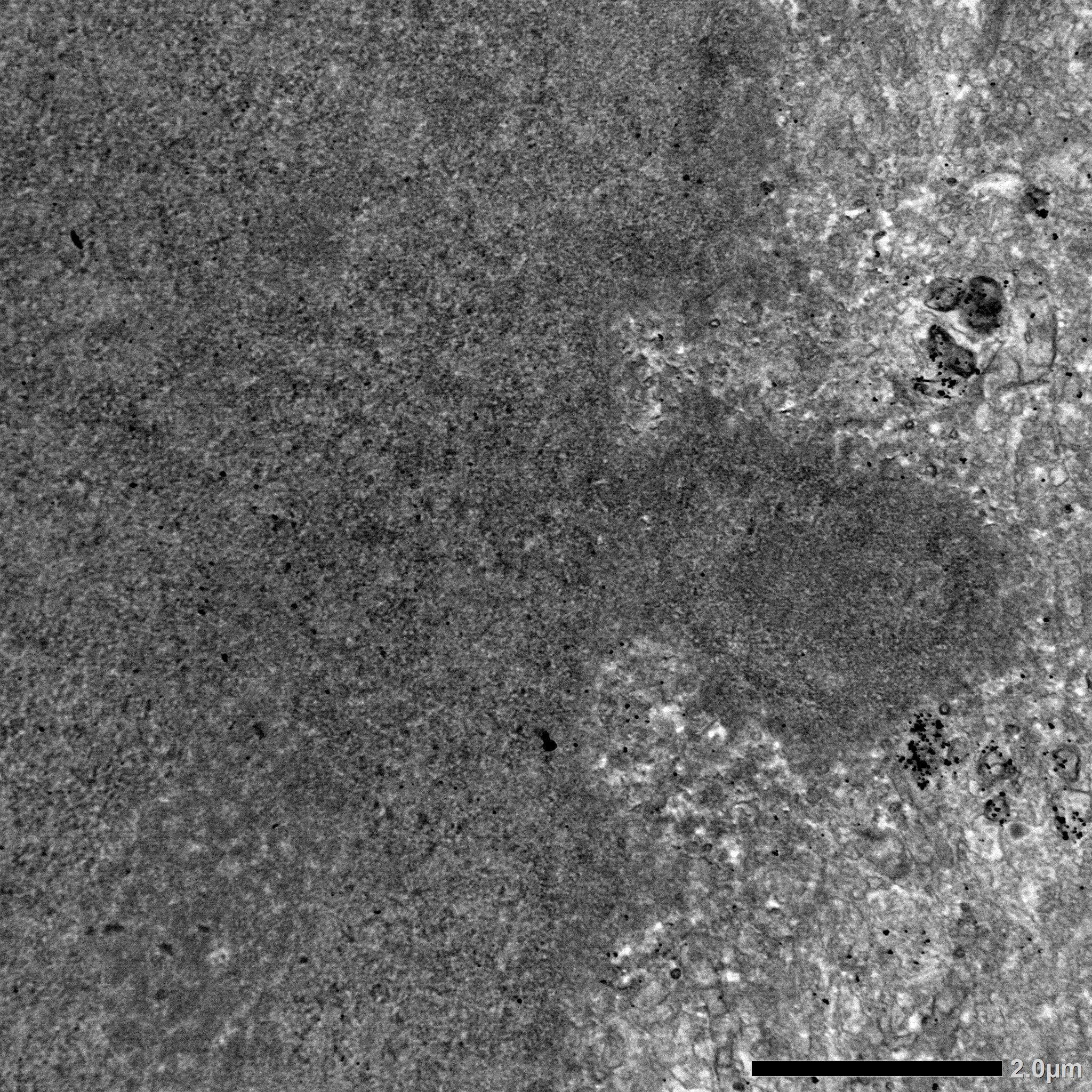

Supplement: Supplementary file 3 — Source data Fig. 2 [file 44318_2024_192_MOESM3_ESM.zip › Figure2/Figure2e/#2_left.jpg]

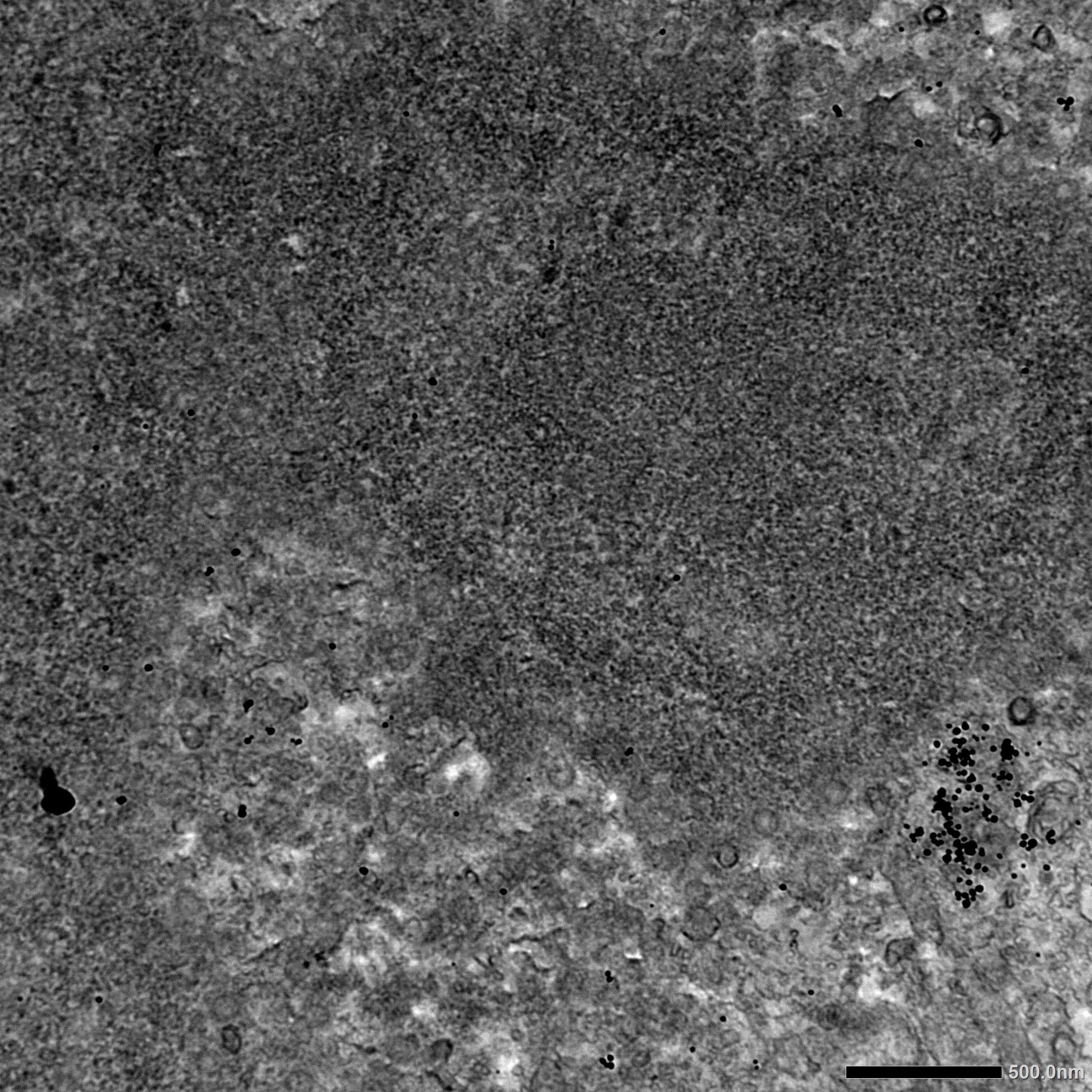

Supplement: Supplementary file 3 — Source data Fig. 2 [file 44318_2024_192_MOESM3_ESM.zip › Figure2/Figure2e/#2_right.jpg]

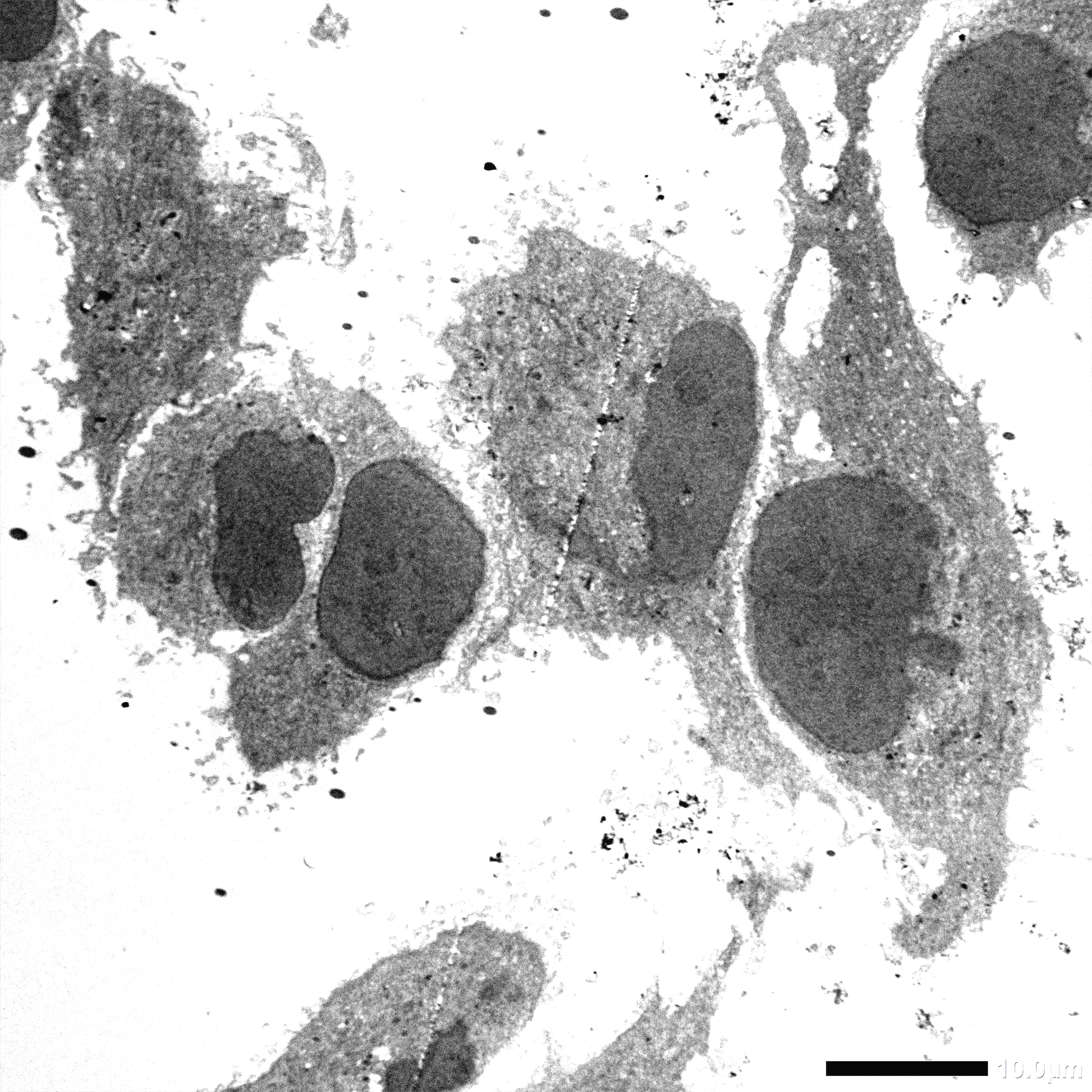

Supplement: Supplementary file 3 — Source data Fig. 2 [file 44318_2024_192_MOESM3_ESM.zip › Figure2/Figure2e/#2_upper.jpeg]

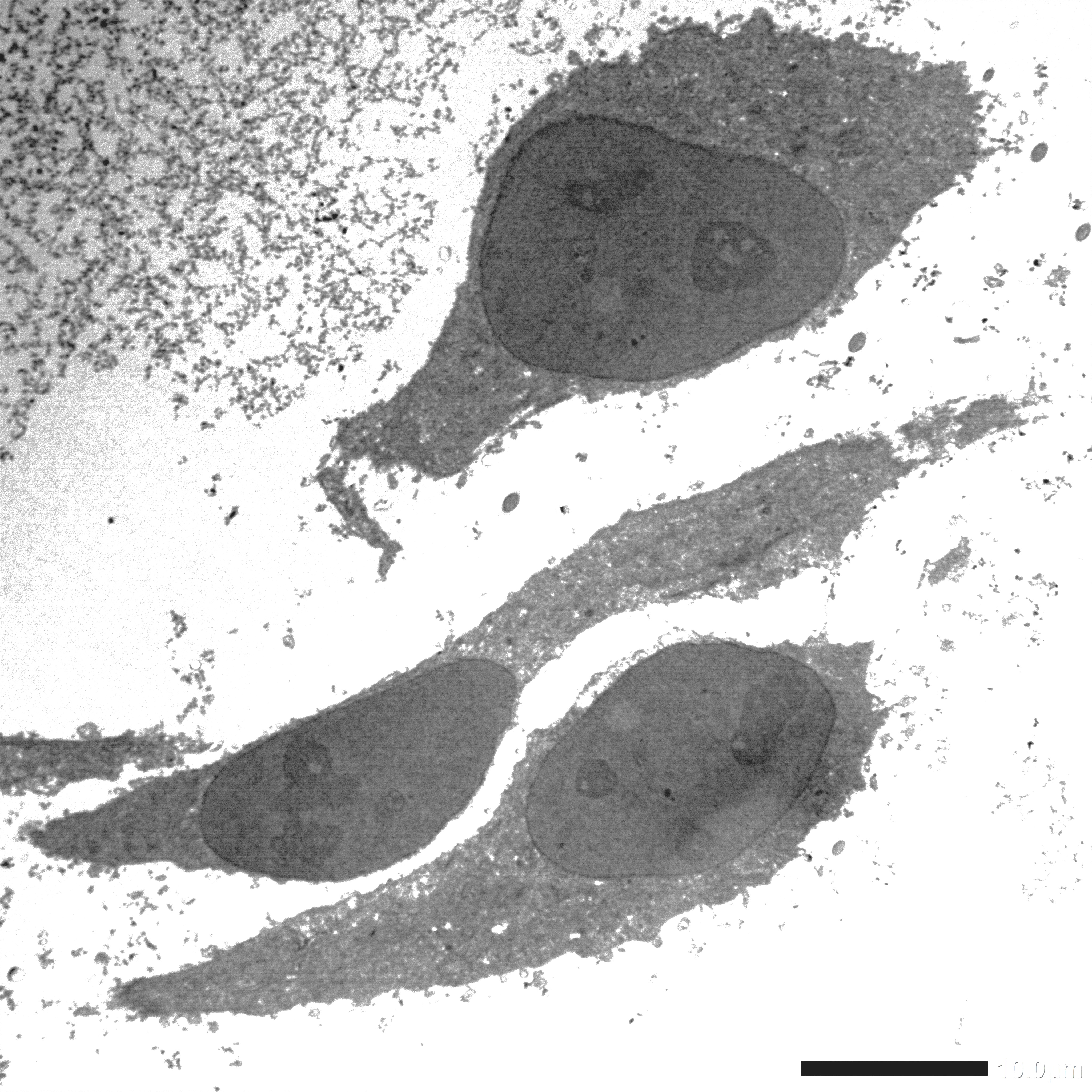

Supplement: Supplementary file 3 — Source data Fig. 2 [file 44318_2024_192_MOESM3_ESM.zip › Figure2/Figure2e/#3_left.jpeg]

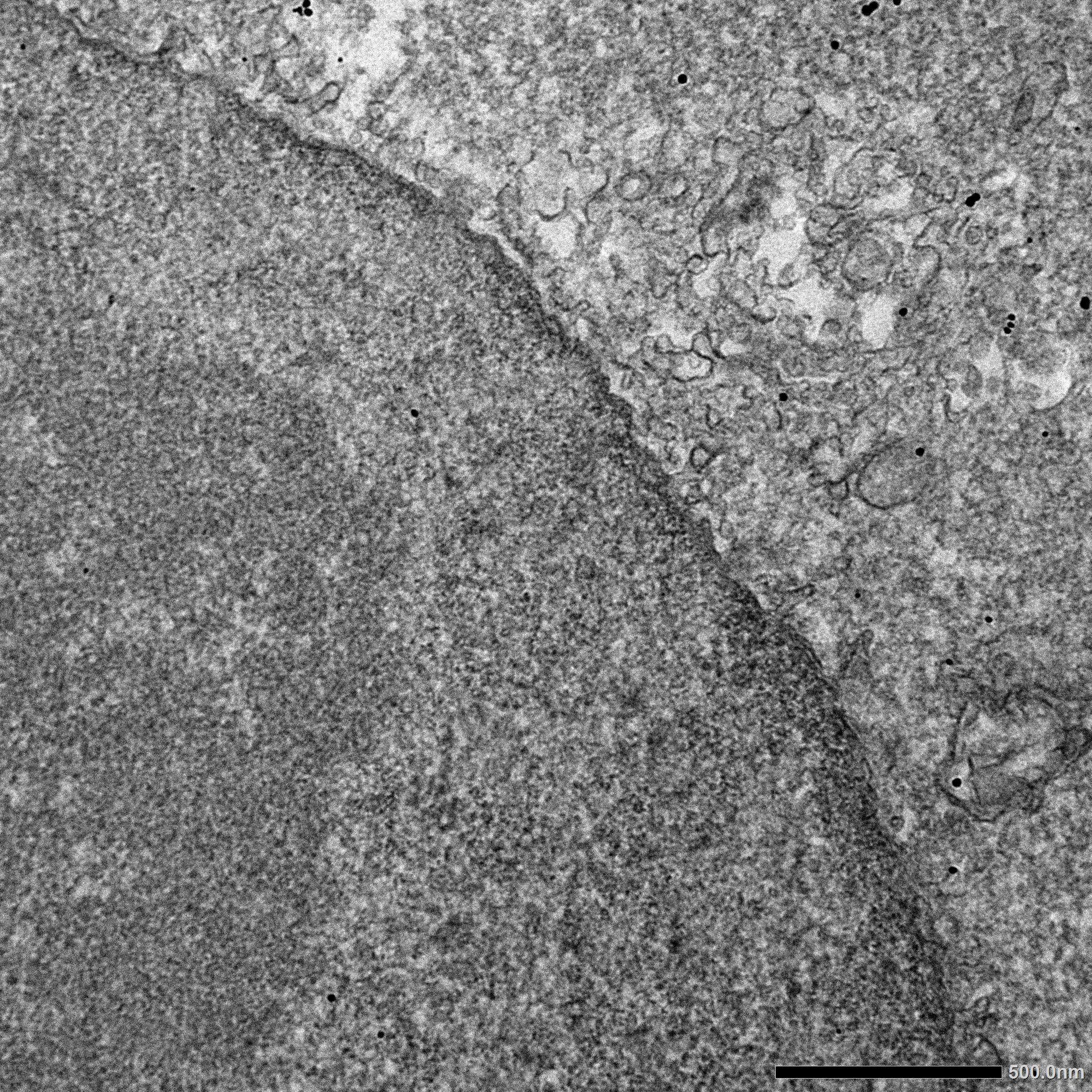

Supplement: Supplementary file 3 — Source data Fig. 2 [file 44318_2024_192_MOESM3_ESM.zip › Figure2/Figure2e/#3_right.jpg]

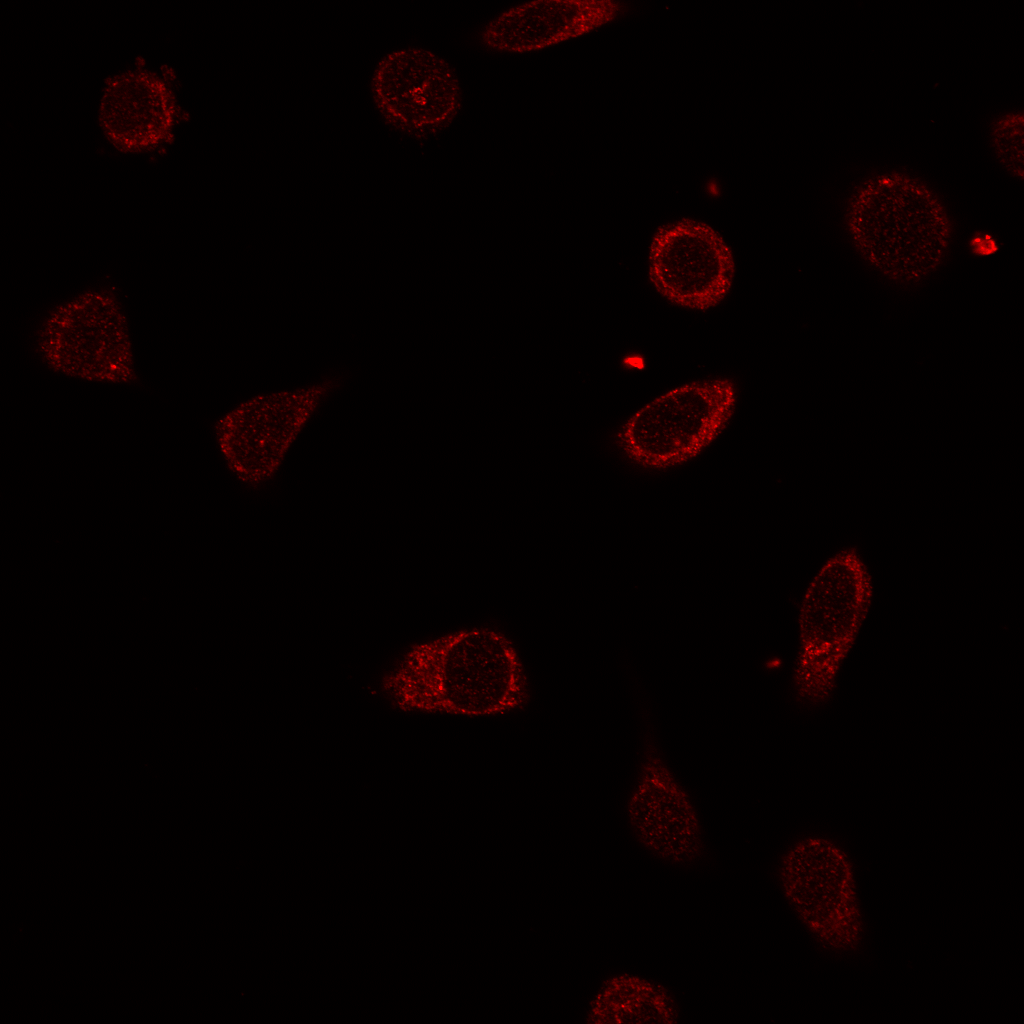

Supplement: Supplementary file 4 — Source data Fig. 3 [file 44318_2024_192_MOESM4_ESM.zip › Figure3/Figure3a/10G/beta-Gal.tif]

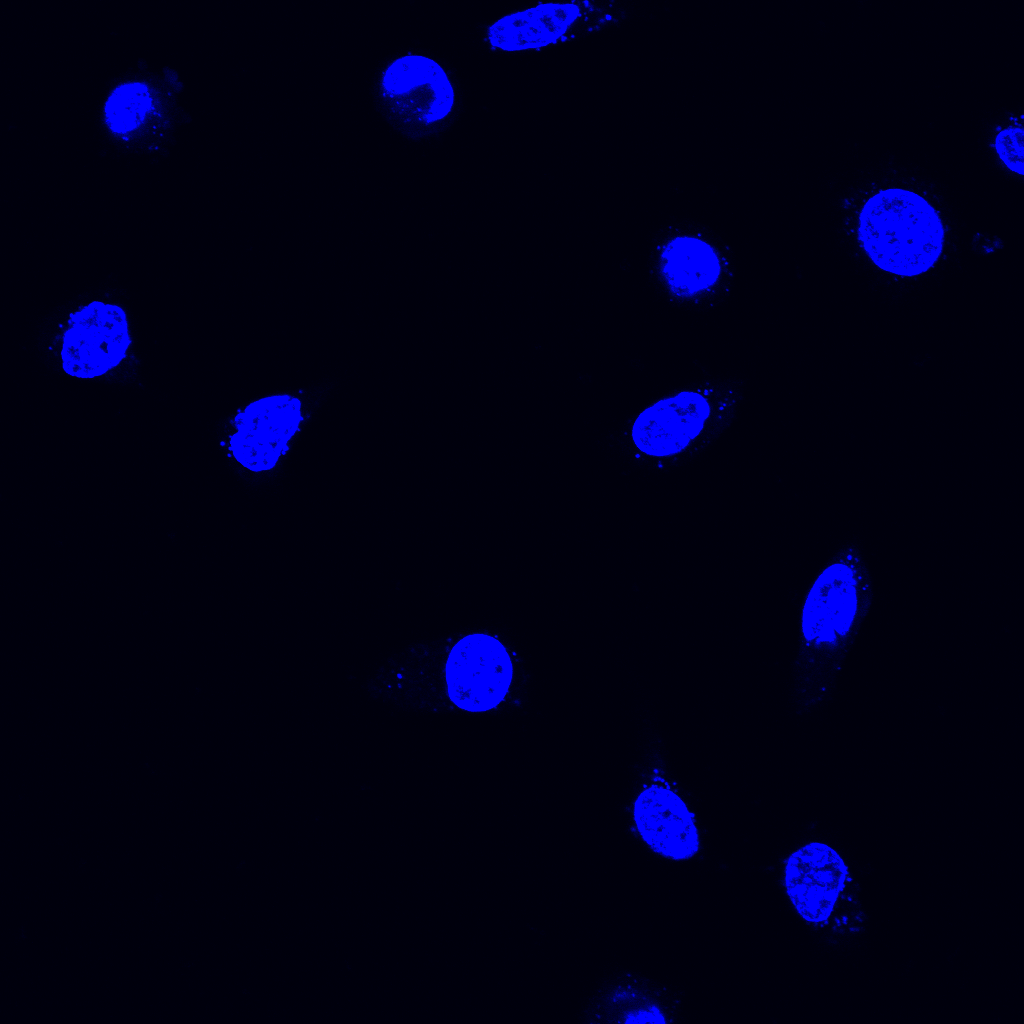

Supplement: Supplementary file 4 — Source data Fig. 3 [file 44318_2024_192_MOESM4_ESM.zip › Figure3/Figure3a/10G/Hoechst.tif]

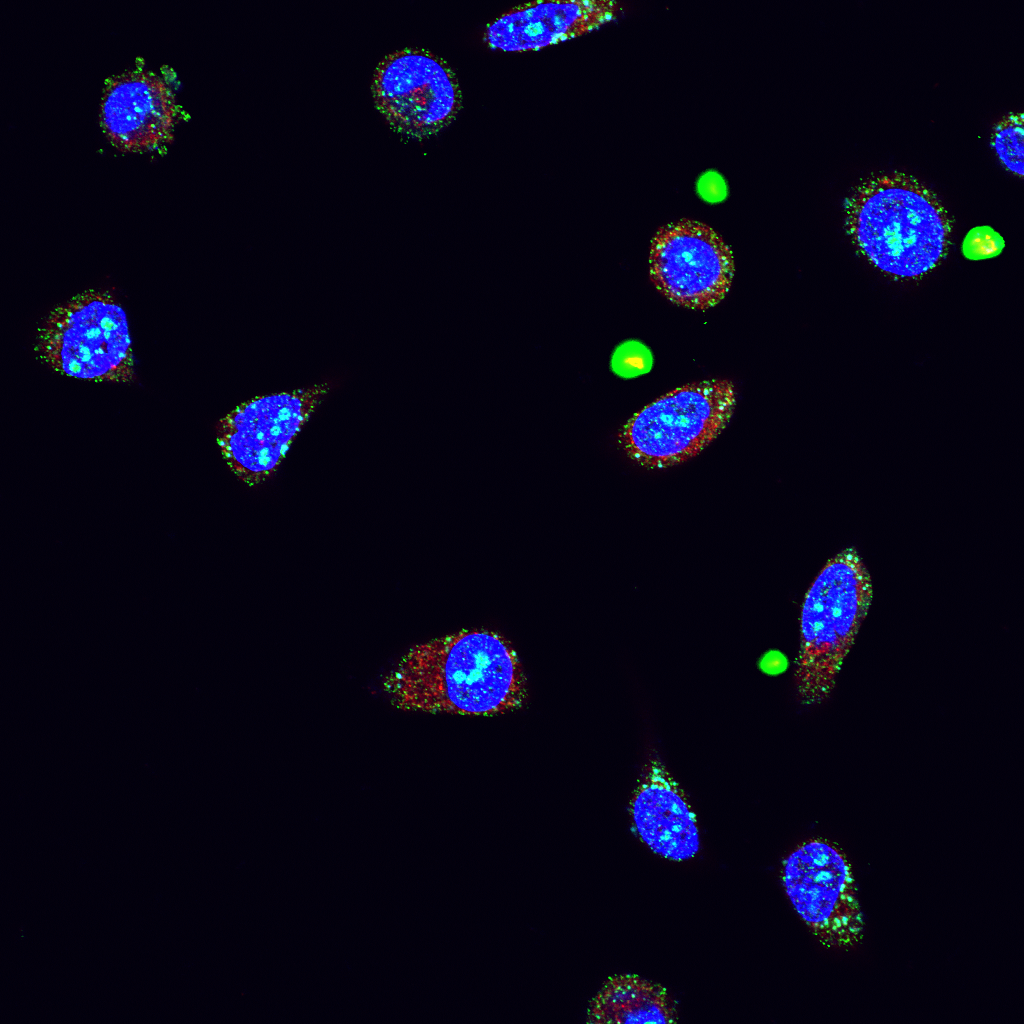

Supplement: Supplementary file 4 — Source data Fig. 3 [file 44318_2024_192_MOESM4_ESM.zip › Figure3/Figure3a/10G/Merge.tif]

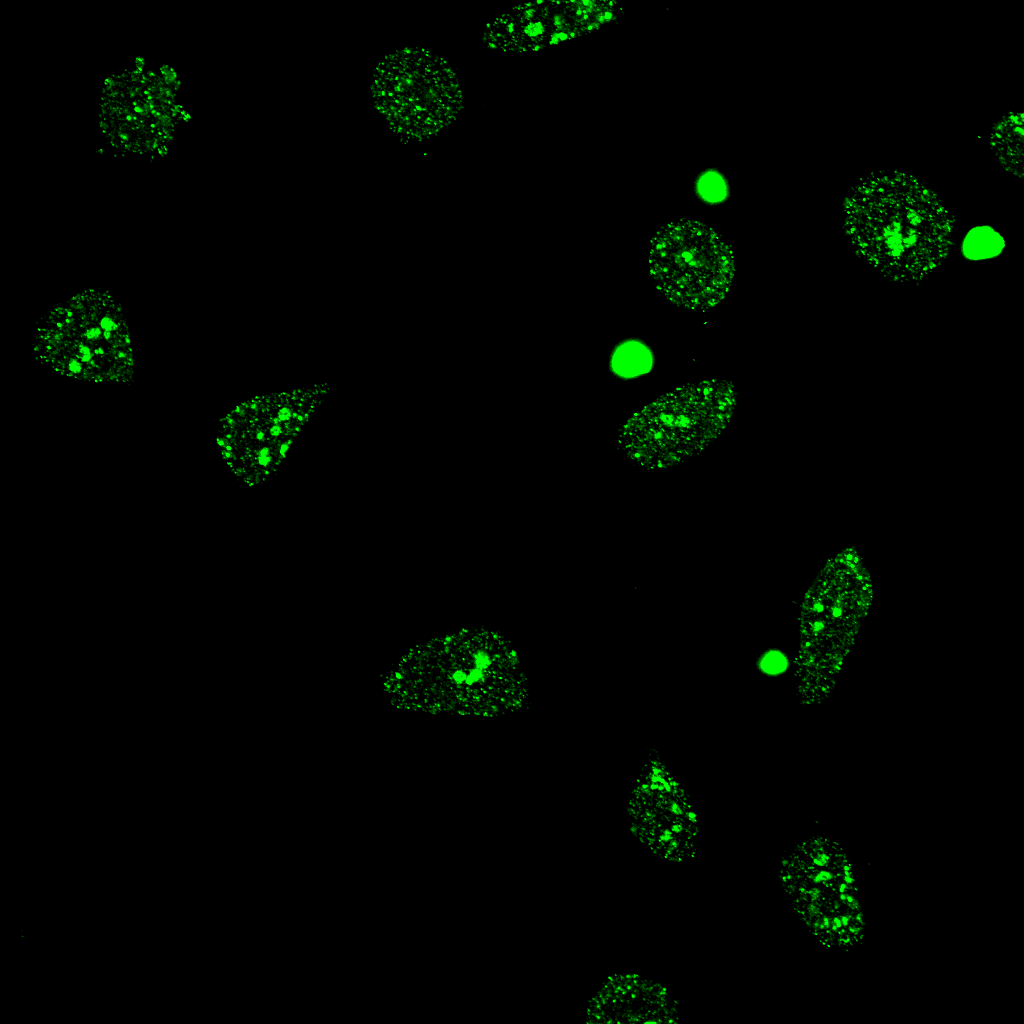

Supplement: Supplementary file 4 — Source data Fig. 3 [file 44318_2024_192_MOESM4_ESM.zip › Figure3/Figure3a/10G/PQBP3.tif]

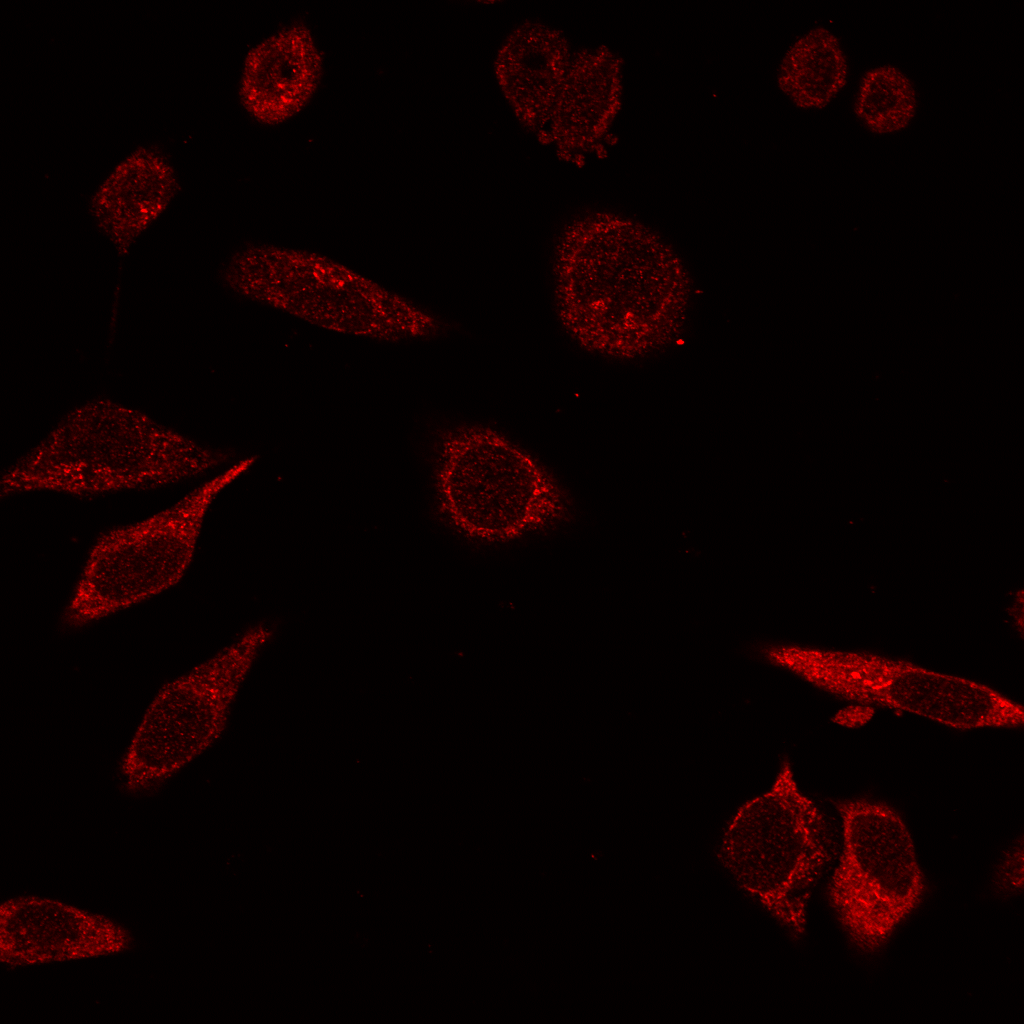

Supplement: Supplementary file 4 — Source data Fig. 3 [file 44318_2024_192_MOESM4_ESM.zip › Figure3/Figure3a/20G/beta-Gal.tif]

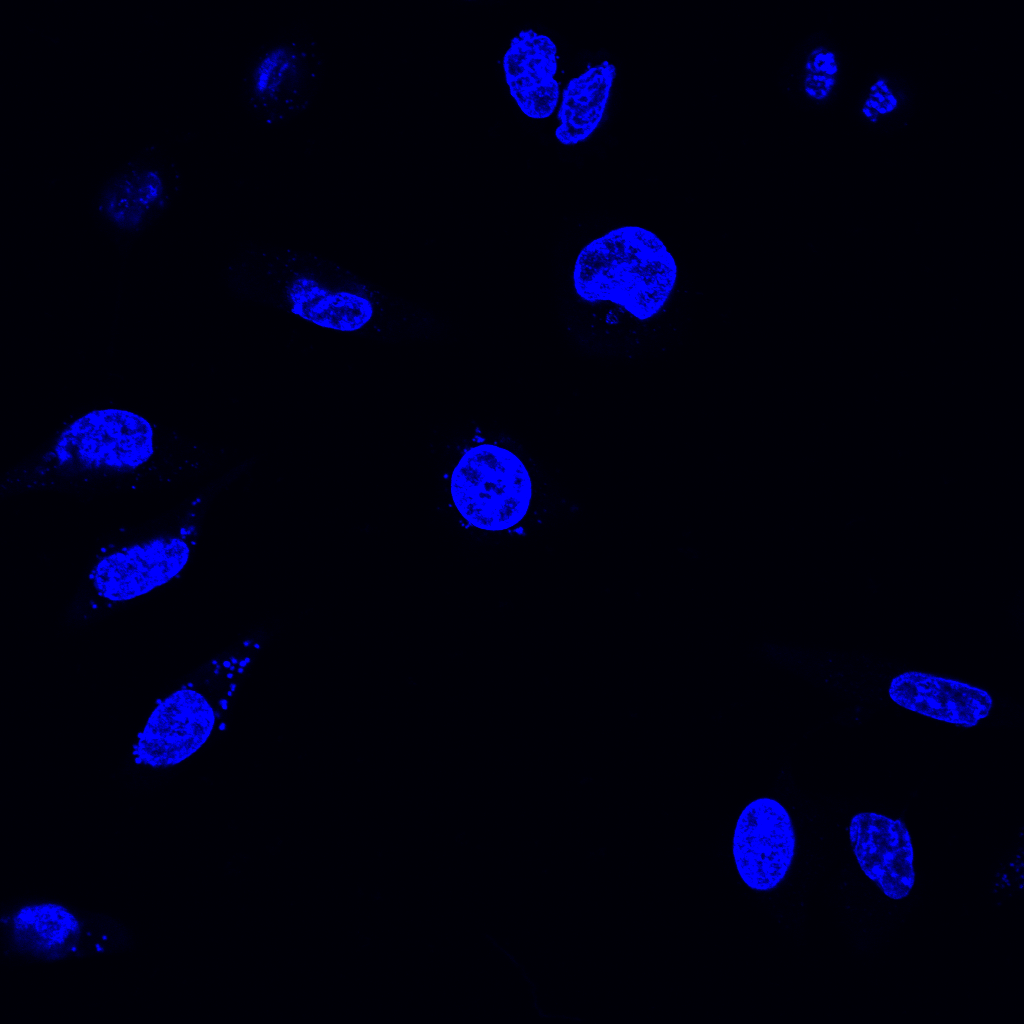

Supplement: Supplementary file 4 — Source data Fig. 3 [file 44318_2024_192_MOESM4_ESM.zip › Figure3/Figure3a/20G/Hoechst.tif]

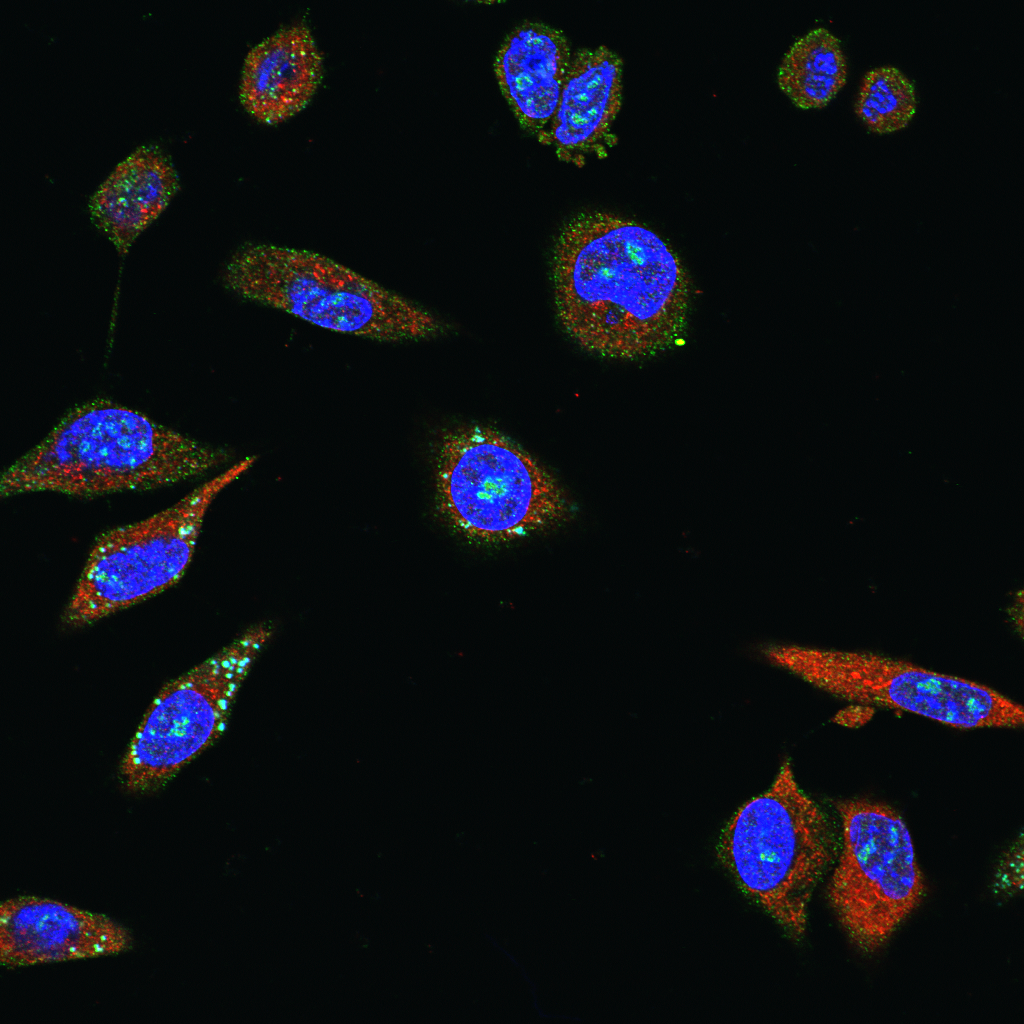

Supplement: Supplementary file 4 — Source data Fig. 3 [file 44318_2024_192_MOESM4_ESM.zip › Figure3/Figure3a/20G/Merge.tif]

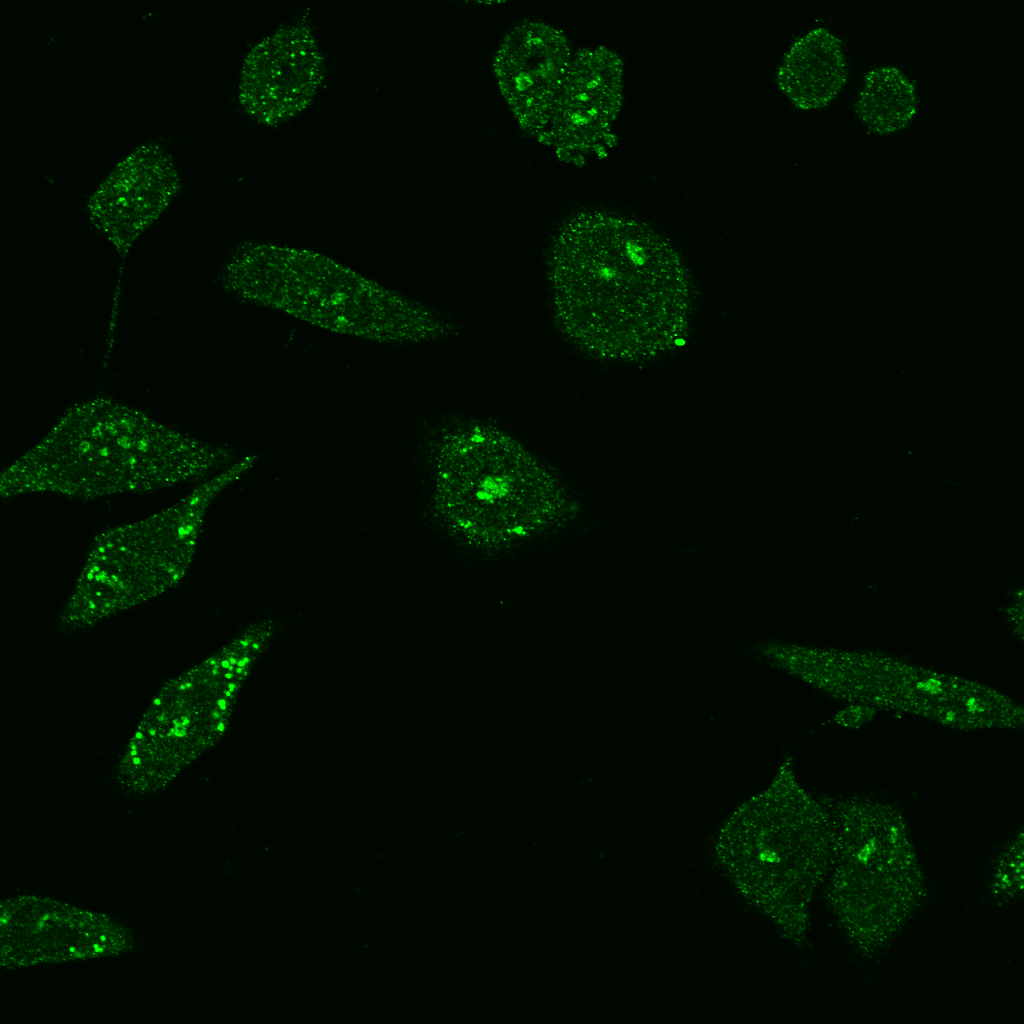

Supplement: Supplementary file 4 — Source data Fig. 3 [file 44318_2024_192_MOESM4_ESM.zip › Figure3/Figure3a/20G/PQBP3.tif]

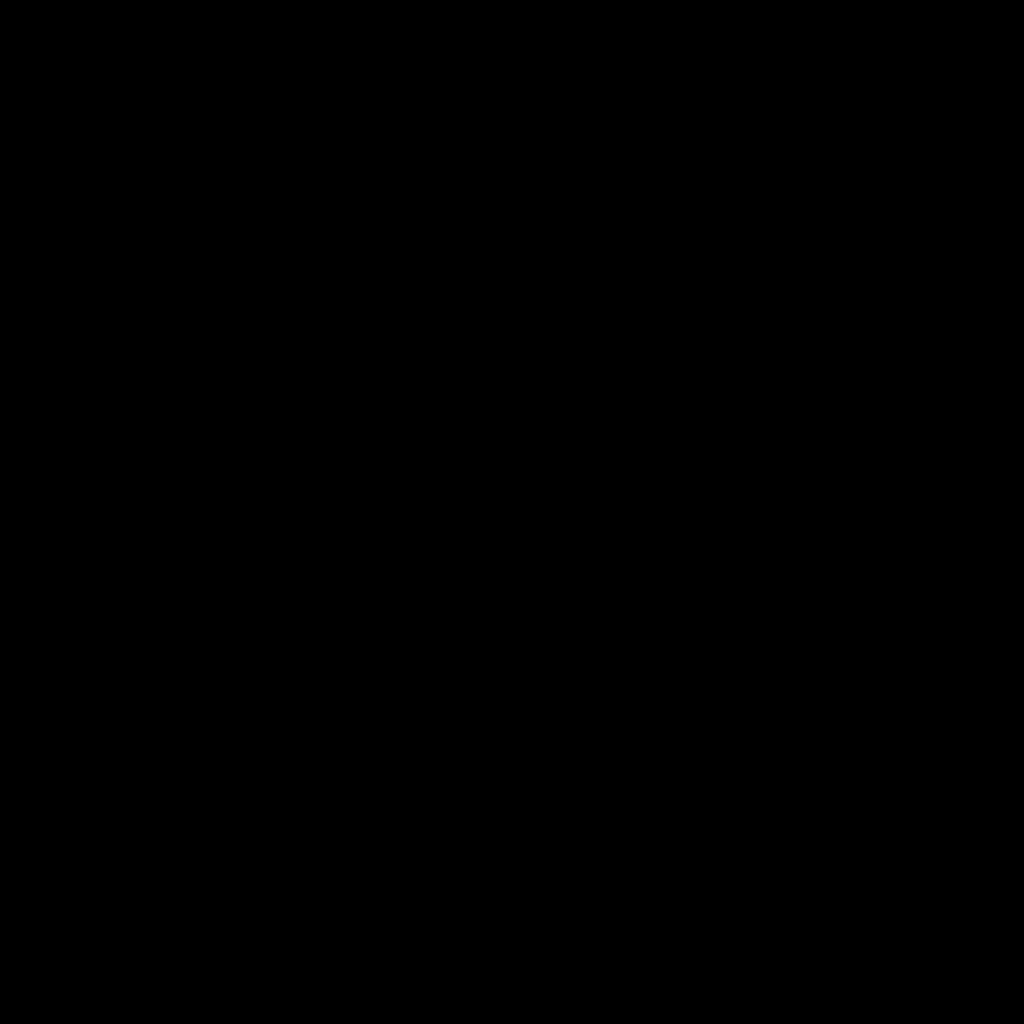

Supplement: Supplementary file 4 — Source data Fig. 3 [file 44318_2024_192_MOESM4_ESM.zip › Figure3/Figure3a/5G/beta-Gal.tif]

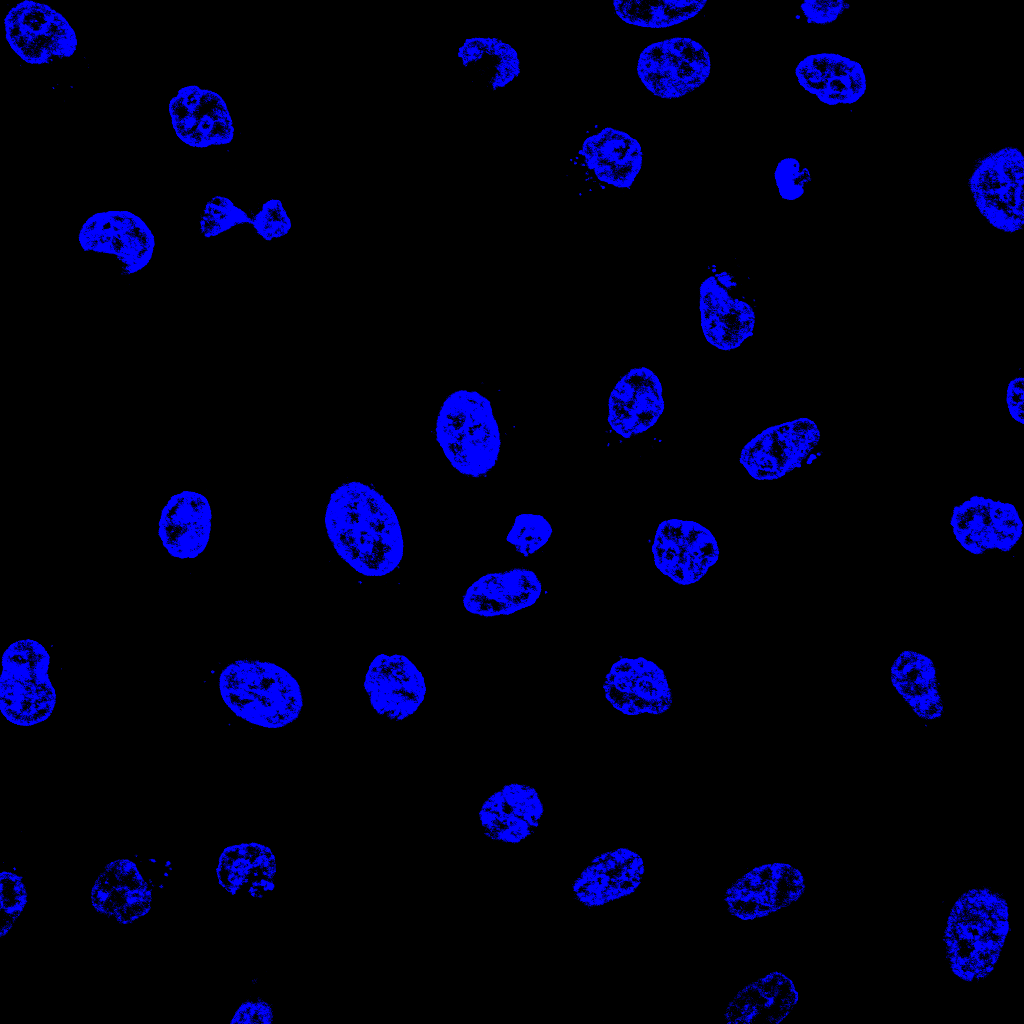

Supplement: Supplementary file 4 — Source data Fig. 3 [file 44318_2024_192_MOESM4_ESM.zip › Figure3/Figure3a/5G/Hoechst.tif]

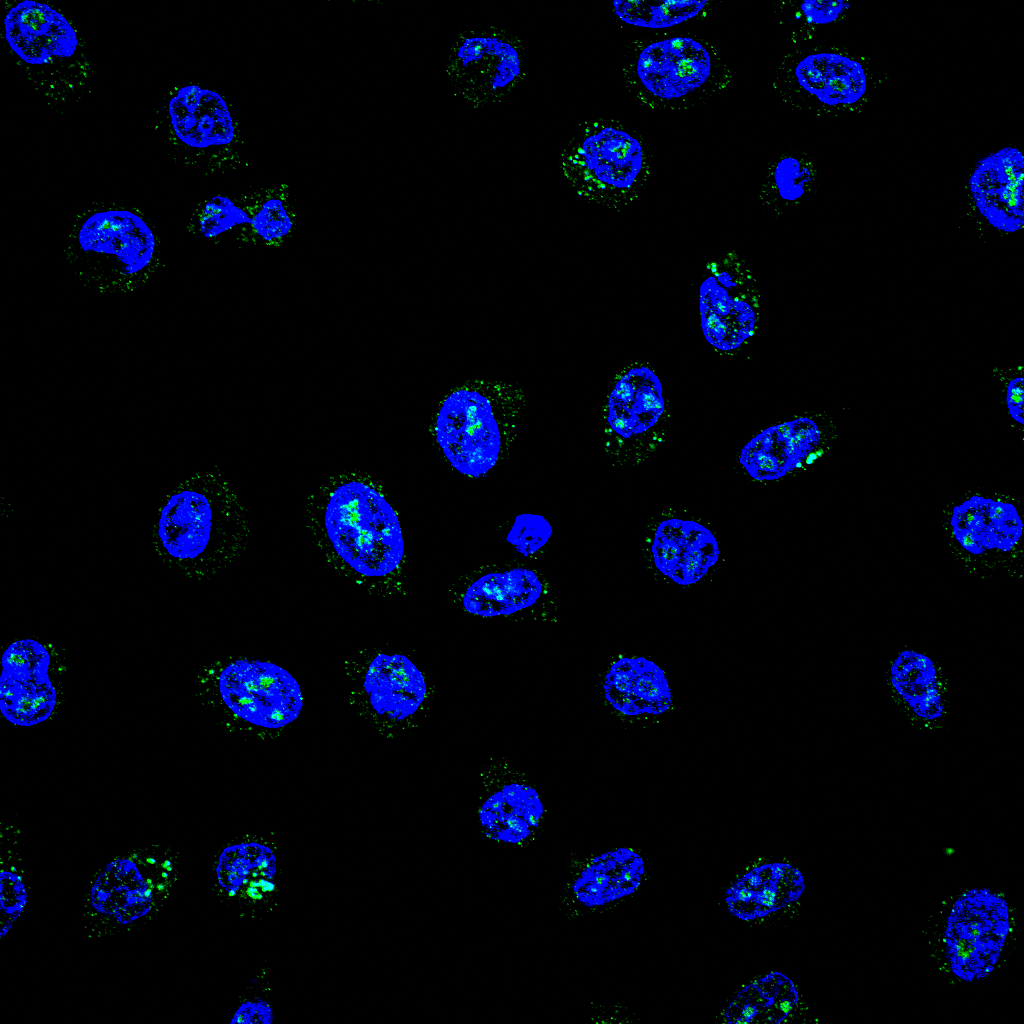

Supplement: Supplementary file 4 — Source data Fig. 3 [file 44318_2024_192_MOESM4_ESM.zip › Figure3/Figure3a/5G/Merge.tif]

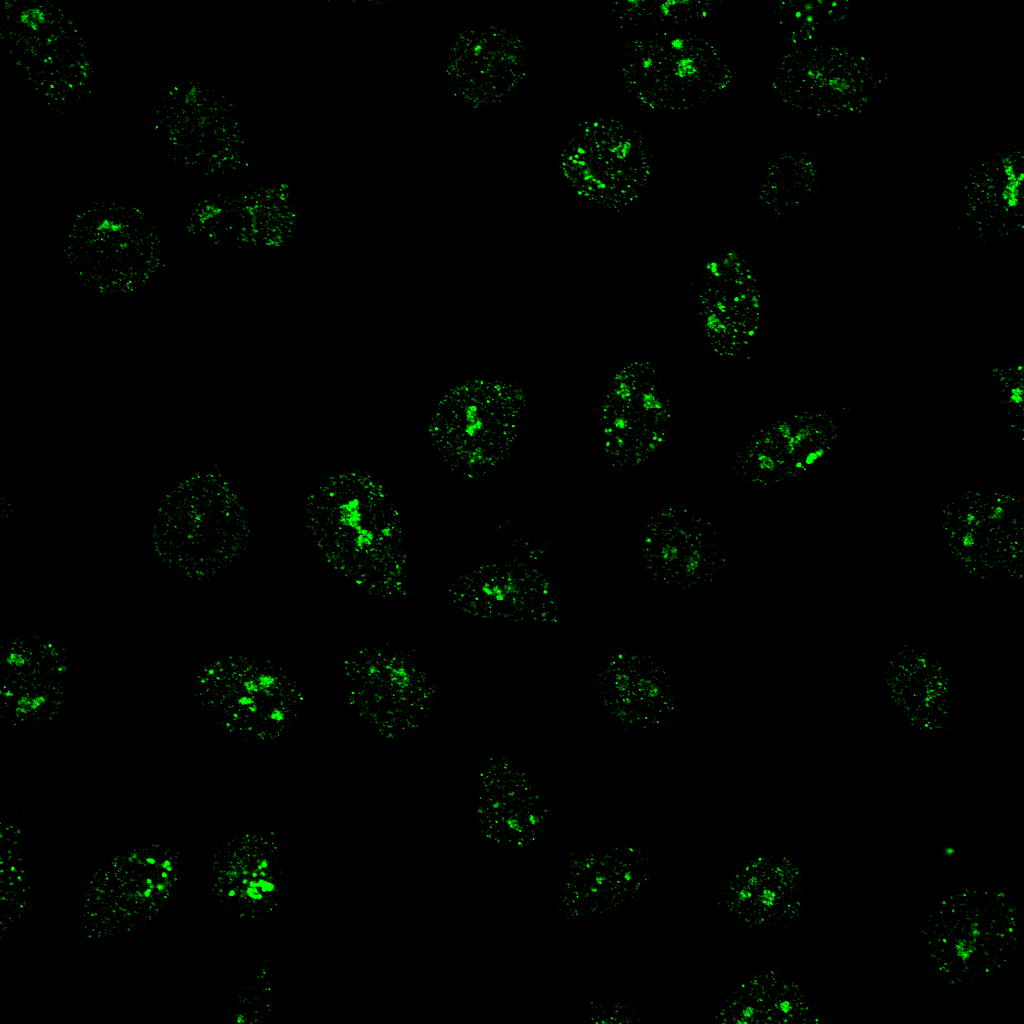

Supplement: Supplementary file 4 — Source data Fig. 3 [file 44318_2024_192_MOESM4_ESM.zip › Figure3/Figure3a/5G/PQBP3.tif]

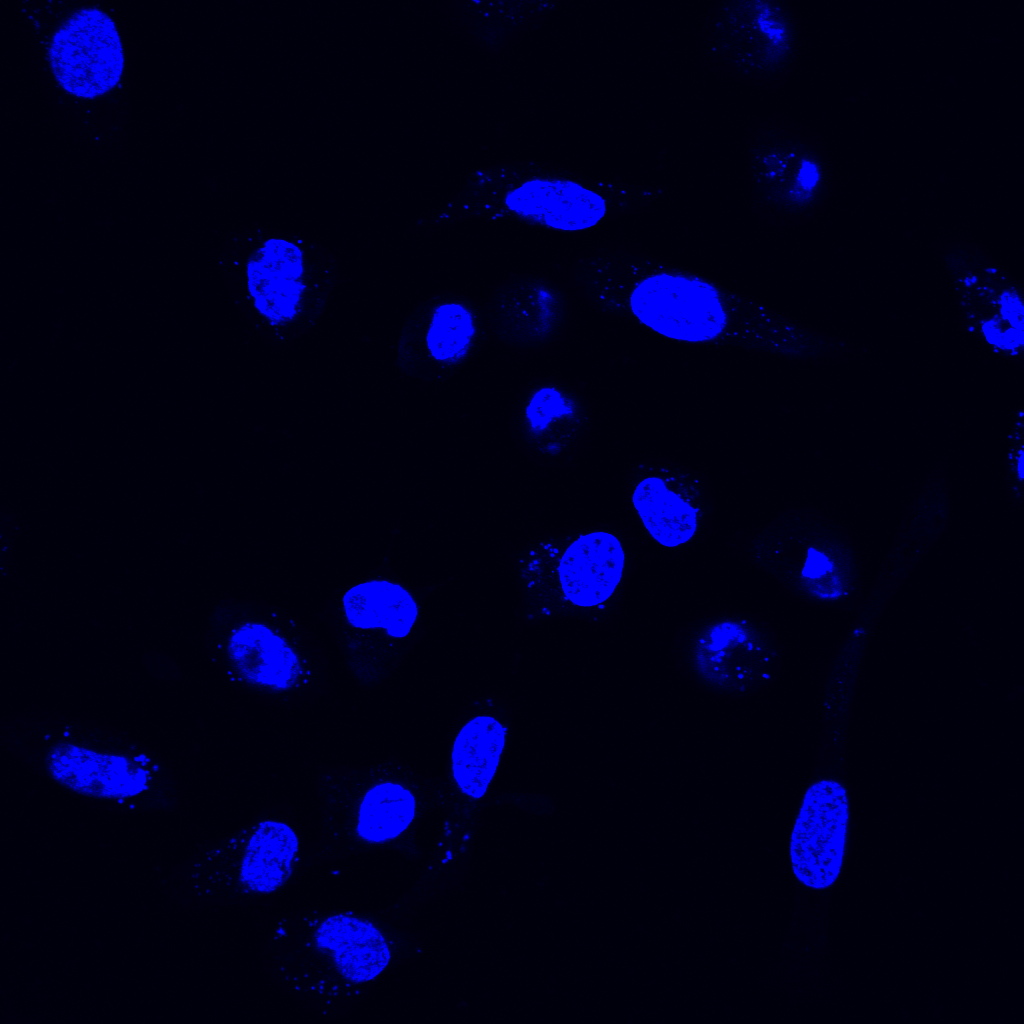

Supplement: Supplementary file 4 — Source data Fig. 3 [file 44318_2024_192_MOESM4_ESM.zip › Figure3/Figure3d/Hoechst 33342.tif]

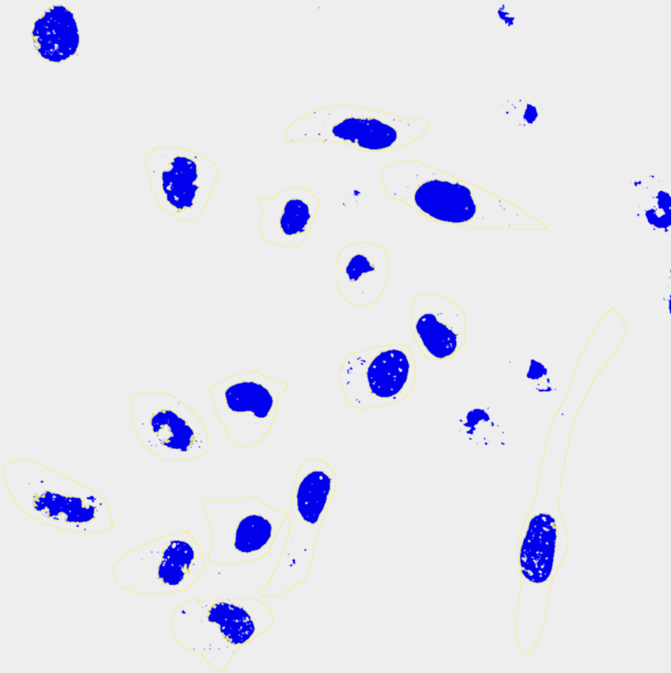

Supplement: Supplementary file 4 — Source data Fig. 3 [file 44318_2024_192_MOESM4_ESM.zip › Figure3/Figure3d/Hoechst33342_white_back.tiff]

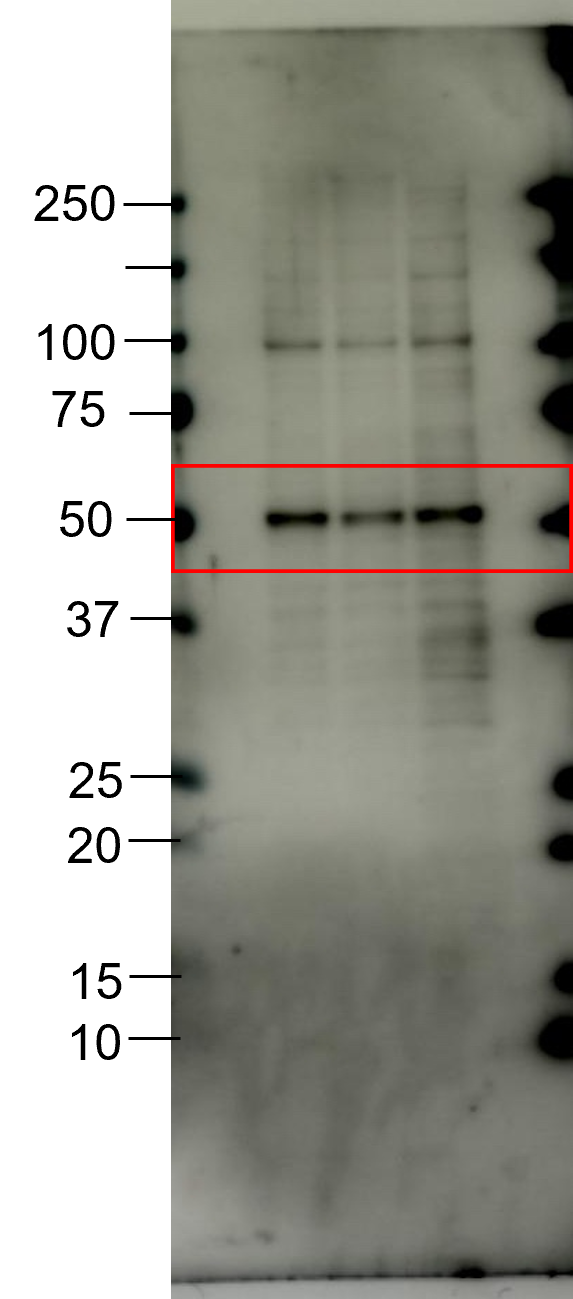

Supplement: Supplementary file 4 — Source data Fig. 3 [file 44318_2024_192_MOESM4_ESM.zip › Figure3/Figure3e/Cytoplasmic fraction_beta-Tub.tif]

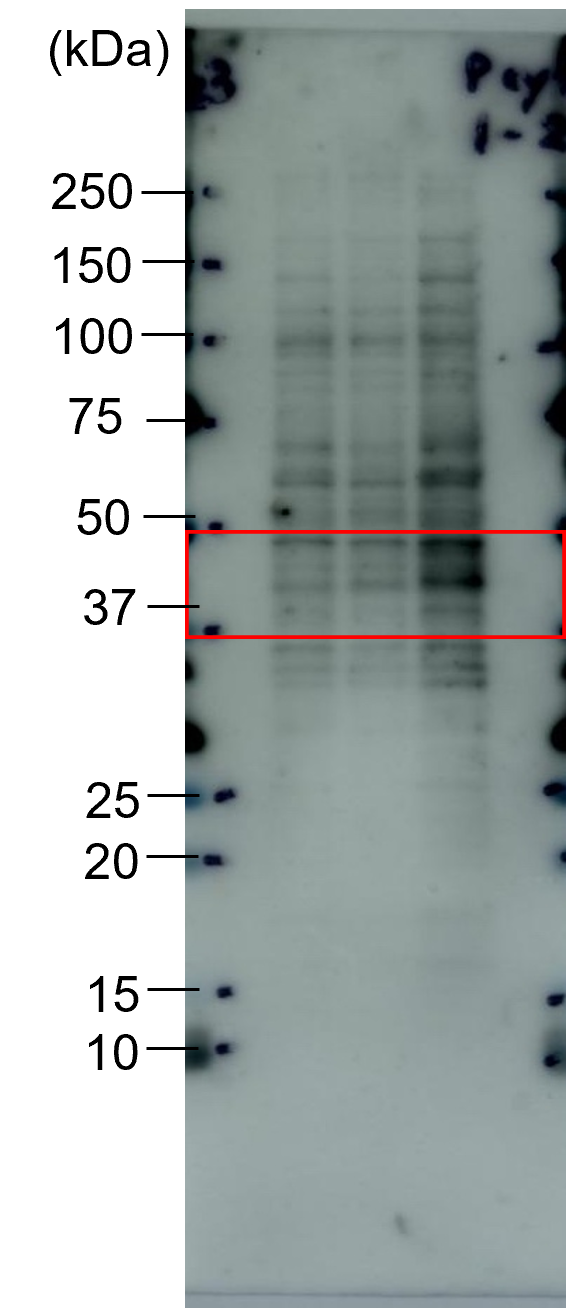

Supplement: Supplementary file 4 — Source data Fig. 3 [file 44318_2024_192_MOESM4_ESM.zip › Figure3/Figure3e/Cytoplasmic fraction_PQBP3.tif]

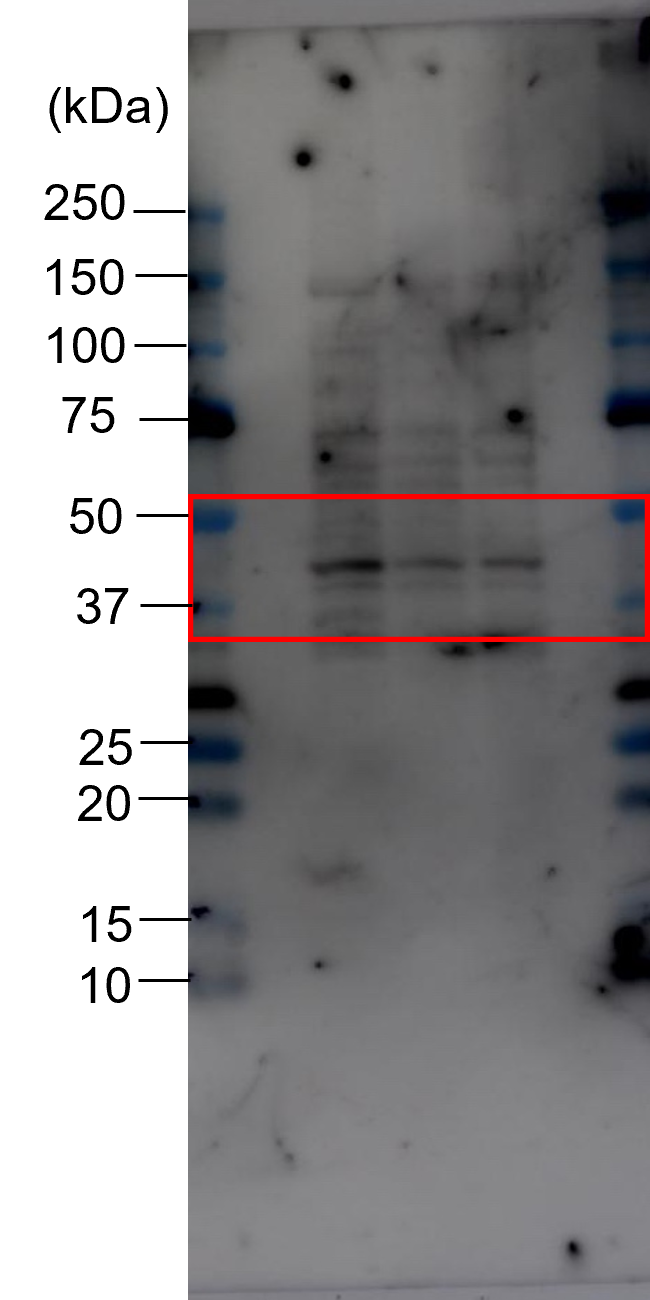

Supplement: Supplementary file 4 — Source data Fig. 3 [file 44318_2024_192_MOESM4_ESM.zip › Figure3/Figure3e/Nuclear fraction PQBP3.tif]

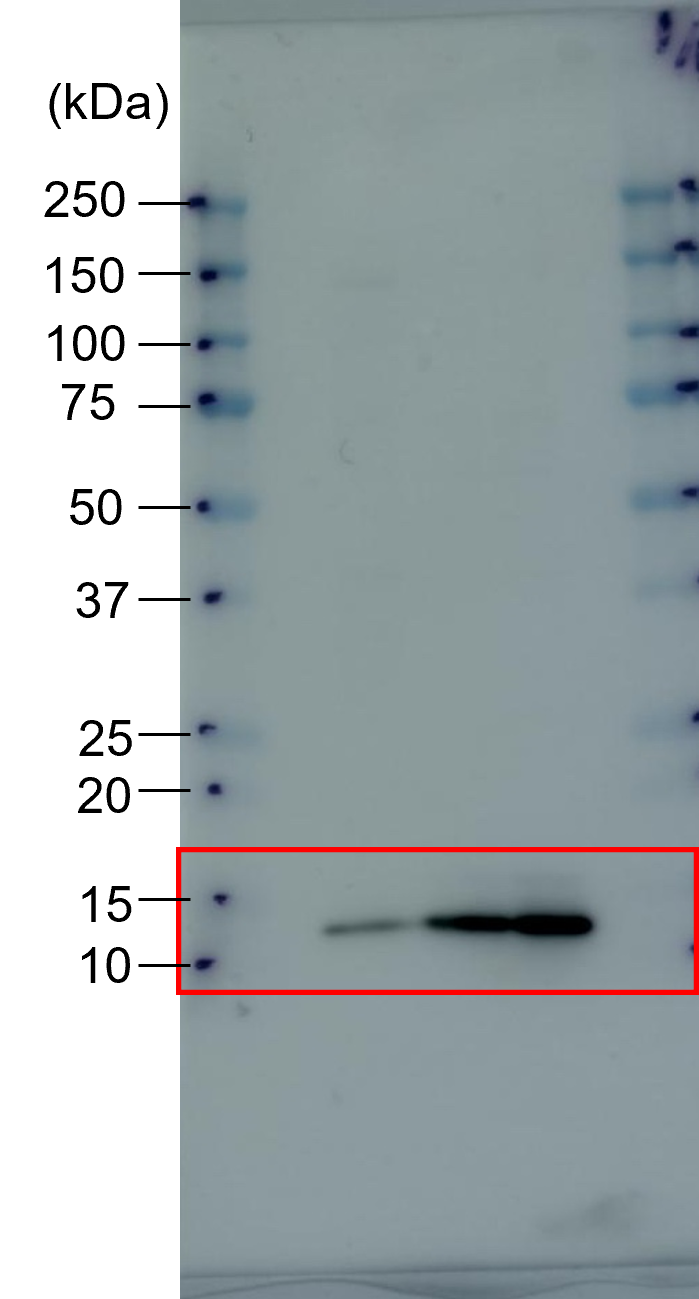

Supplement: Supplementary file 4 — Source data Fig. 3 [file 44318_2024_192_MOESM4_ESM.zip › Figure3/Figure3e/Nuclear fraction_histone.tif]

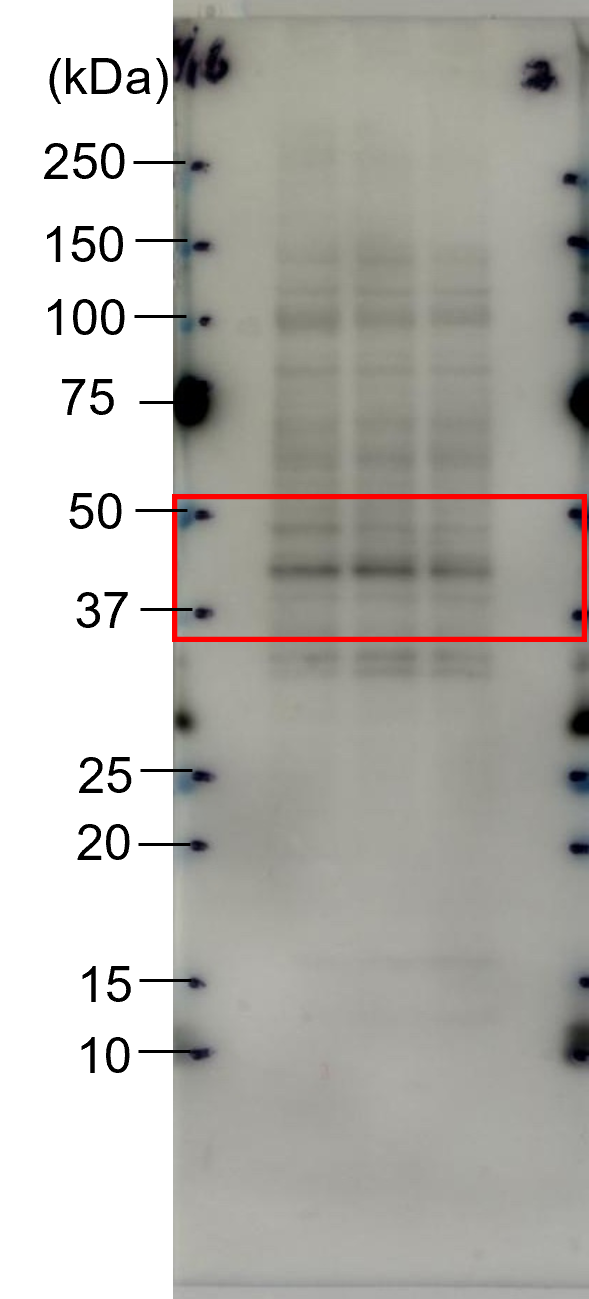

Supplement: Supplementary file 4 — Source data Fig. 3 [file 44318_2024_192_MOESM4_ESM.zip › Figure3/Figure3e/Total cell extract _PQBP3.tif]

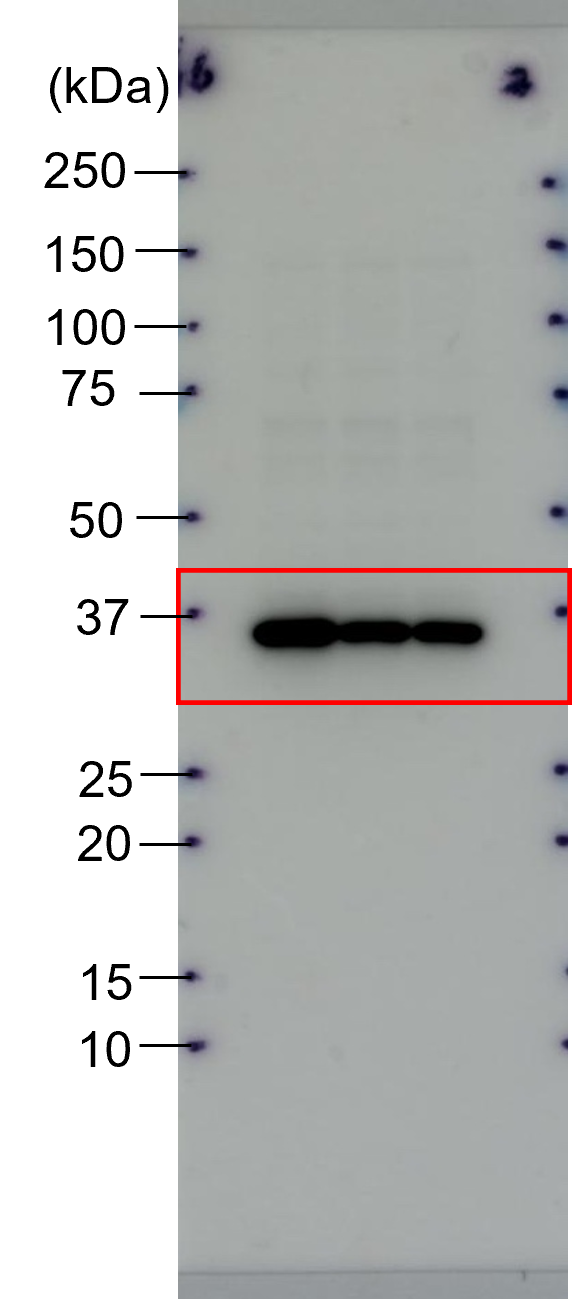

Supplement: Supplementary file 4 — Source data Fig. 3 [file 44318_2024_192_MOESM4_ESM.zip › Figure3/Figure3e/Total cell extract_GAPDH.tif]

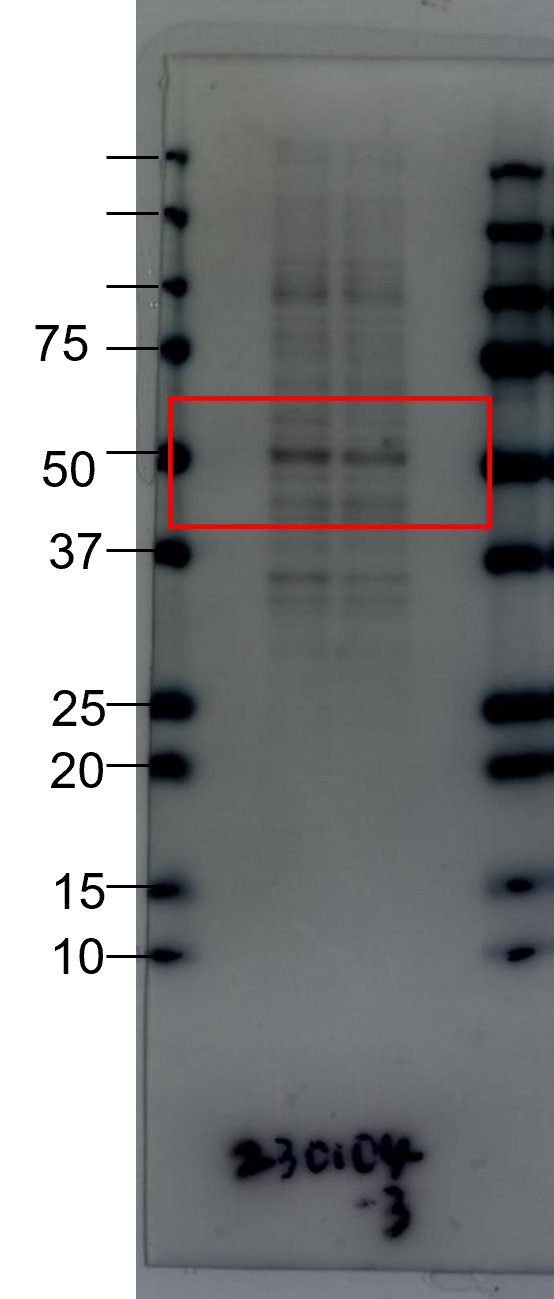

Supplement: Supplementary file 5 — Source data Fig. 4 [file 44318_2024_192_MOESM5_ESM.zip › Figure4/Figure4b/Cytoplasmic fraction_beta-tublin.tif]

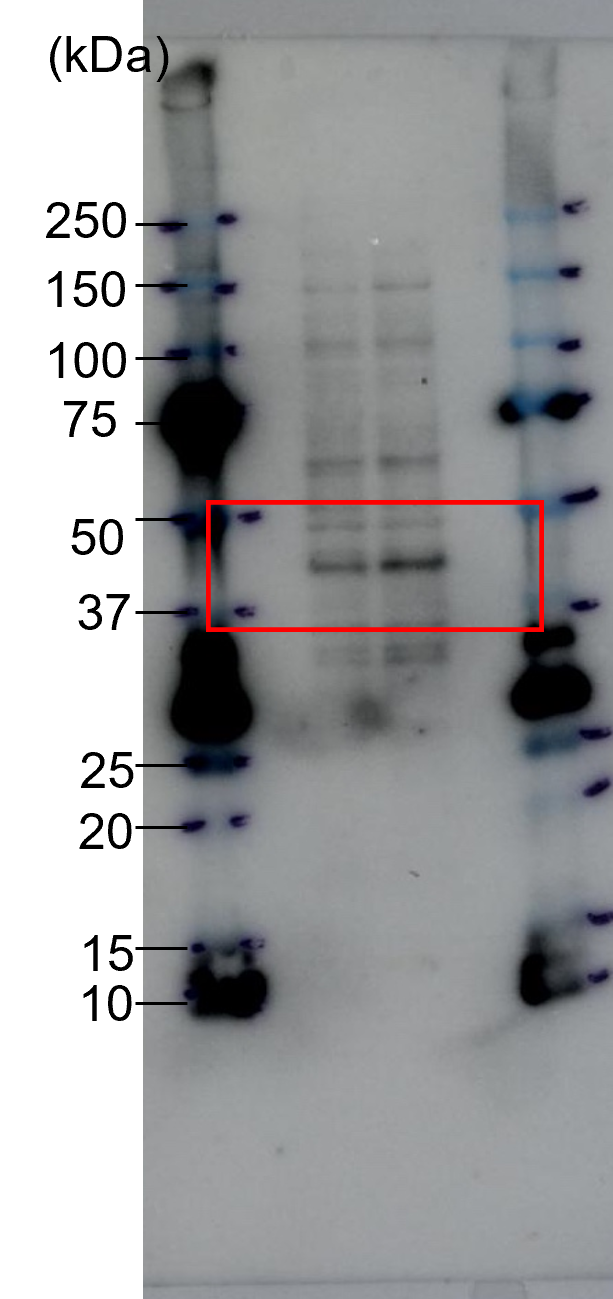

Supplement: Supplementary file 5 — Source data Fig. 4 [file 44318_2024_192_MOESM5_ESM.zip › Figure4/Figure4b/Cytoplasmic fraction_PQBP3.tif]

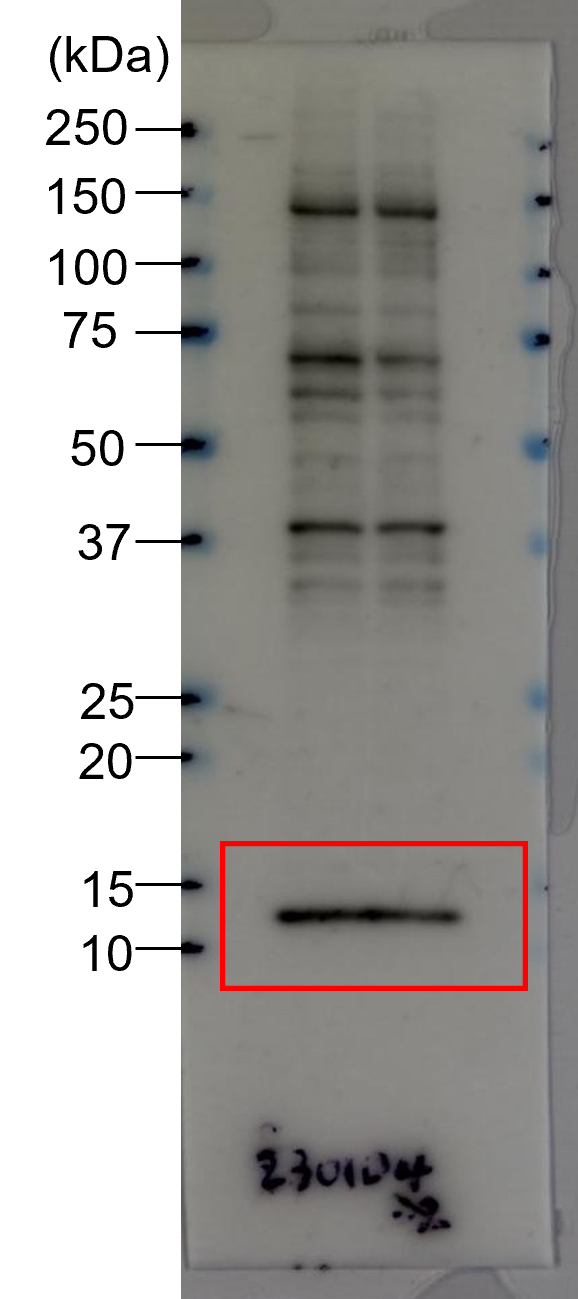

Supplement: Supplementary file 5 — Source data Fig. 4 [file 44318_2024_192_MOESM5_ESM.zip › Figure4/Figure4b/Nuclear fraction_Histone.tif]

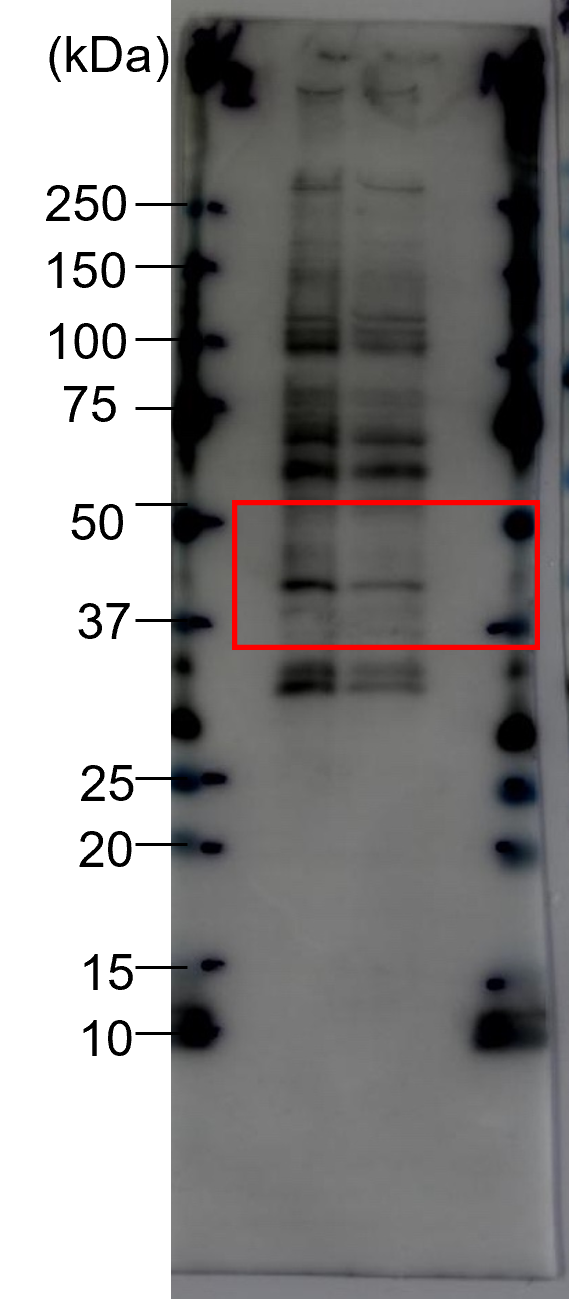

Supplement: Supplementary file 5 — Source data Fig. 4 [file 44318_2024_192_MOESM5_ESM.zip › Figure4/Figure4b/Nuclear fraction_PQBP3.tif]

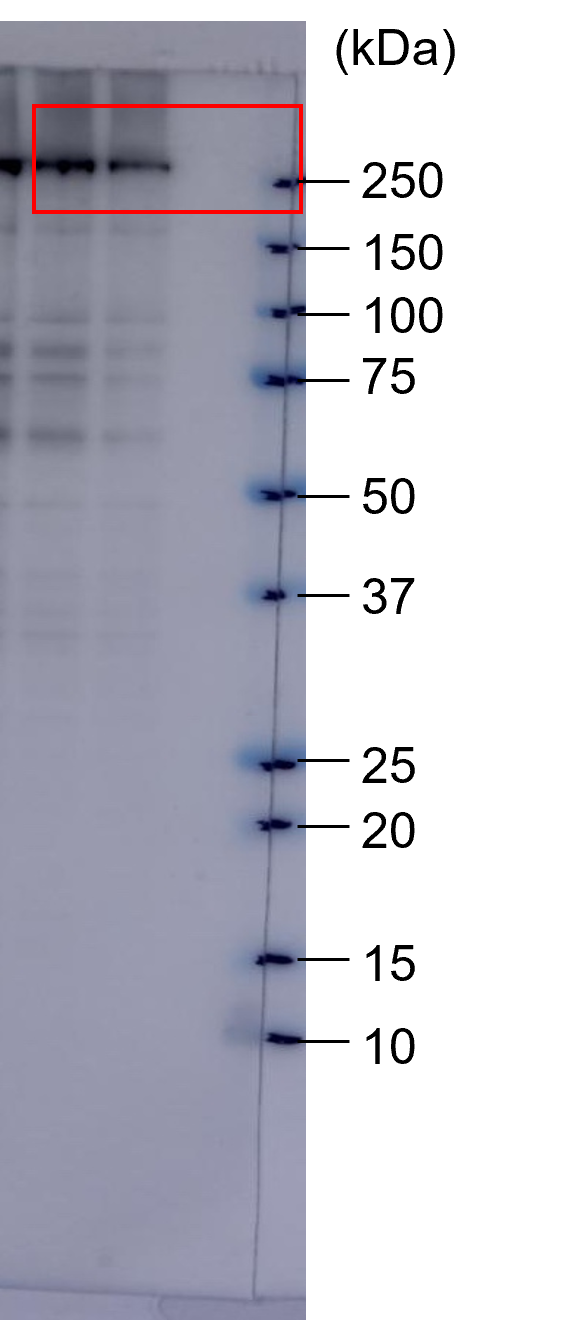

Supplement: Supplementary file 5 — Source data Fig. 4 [file 44318_2024_192_MOESM5_ESM.zip › Figure4/Figure4b/total cell extract_mTOR.tif]

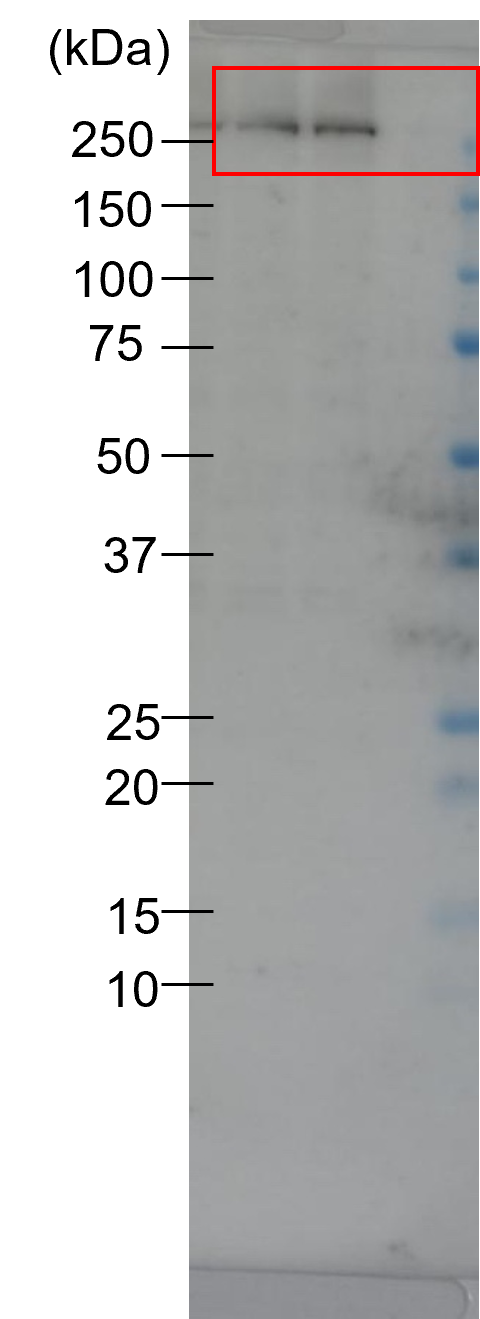

Supplement: Supplementary file 5 — Source data Fig. 4 [file 44318_2024_192_MOESM5_ESM.zip › Figure4/Figure4b/total cell extract_pSer2448-mTOR.tif]

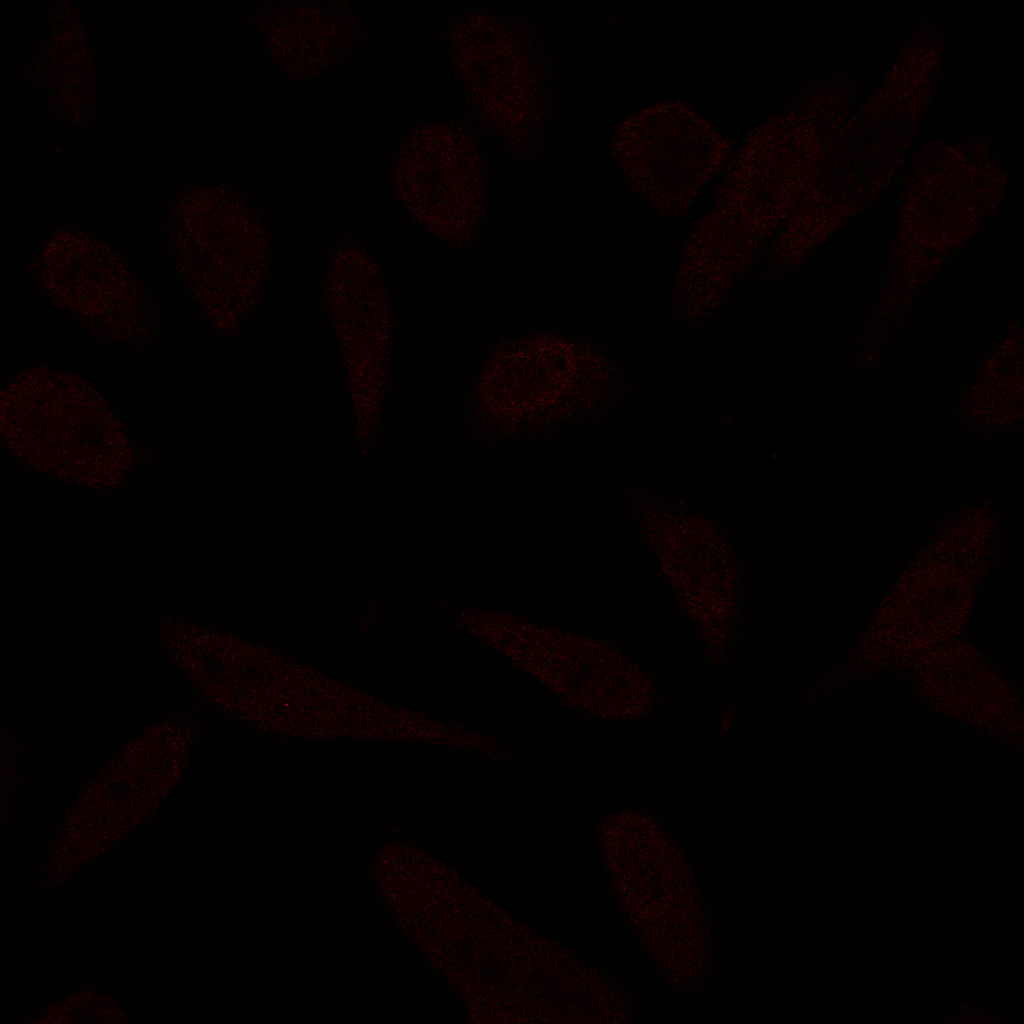

Supplement: Supplementary file 5 — Source data Fig. 4 [file 44318_2024_192_MOESM5_ESM.zip › Figure4/Figure4d/MHY1485_negative/beta-Gal.tif]

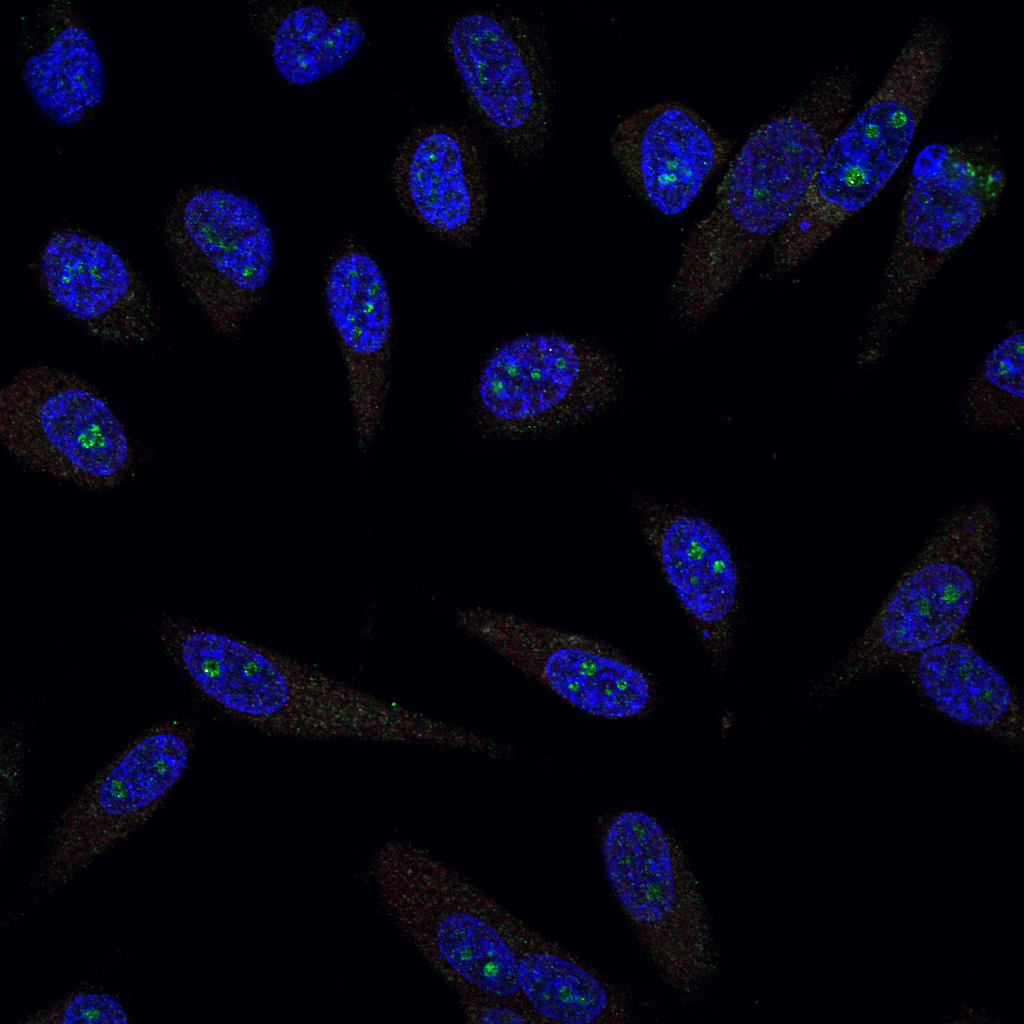

Supplement: Supplementary file 5 — Source data Fig. 4 [file 44318_2024_192_MOESM5_ESM.zip › Figure4/Figure4d/MHY1485_negative/Merge+Hoechst.tif]

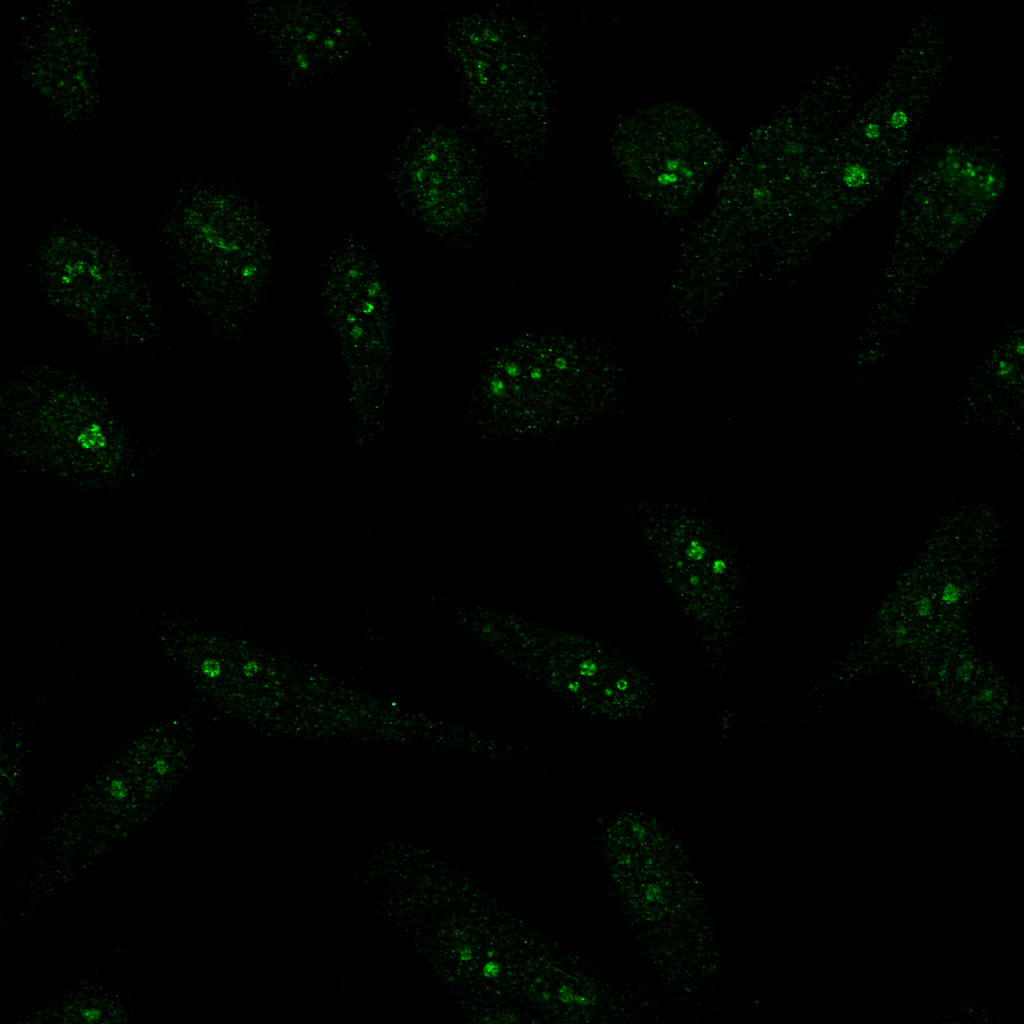

Supplement: Supplementary file 5 — Source data Fig. 4 [file 44318_2024_192_MOESM5_ESM.zip › Figure4/Figure4d/MHY1485_negative/PQBP3.tif]

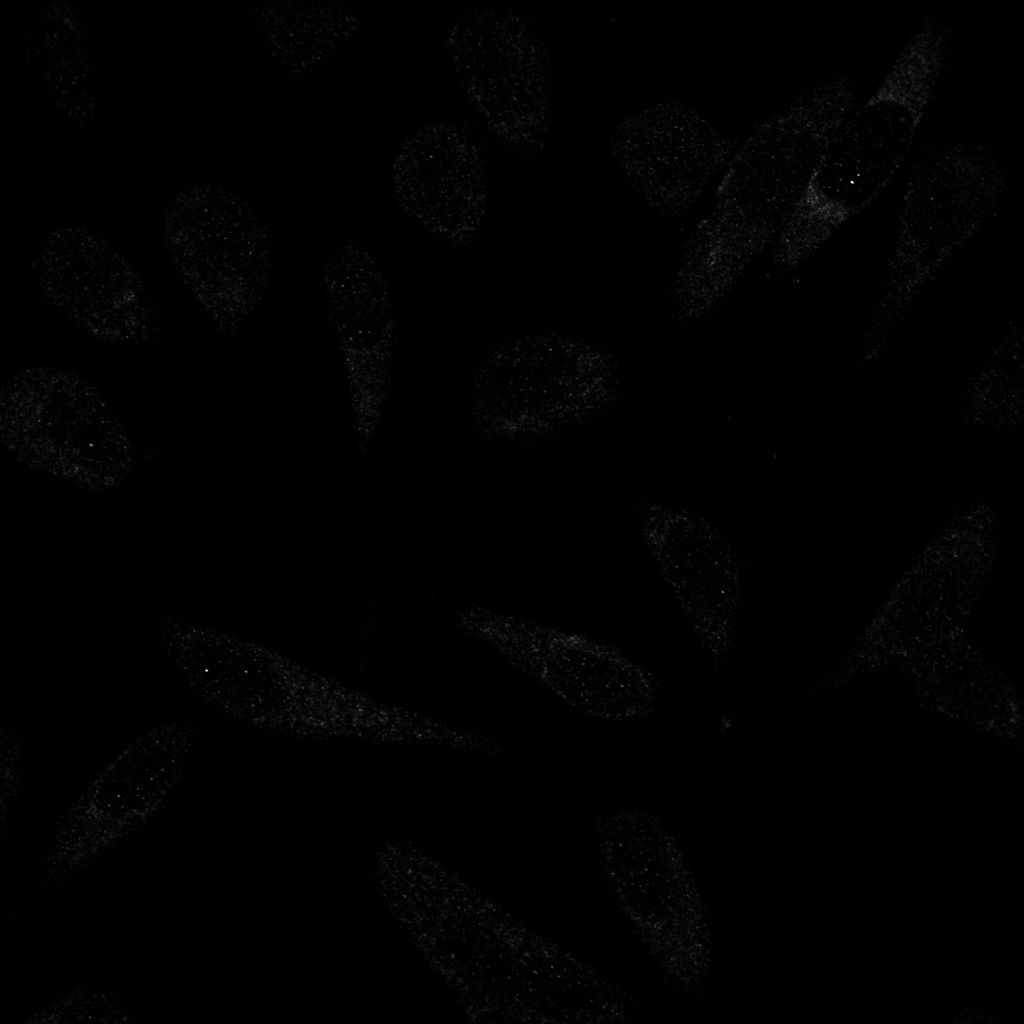

Supplement: Supplementary file 5 — Source data Fig. 4 [file 44318_2024_192_MOESM5_ESM.zip › Figure4/Figure4d/MHY1485_negative/pSer2448-mTOR.tif]

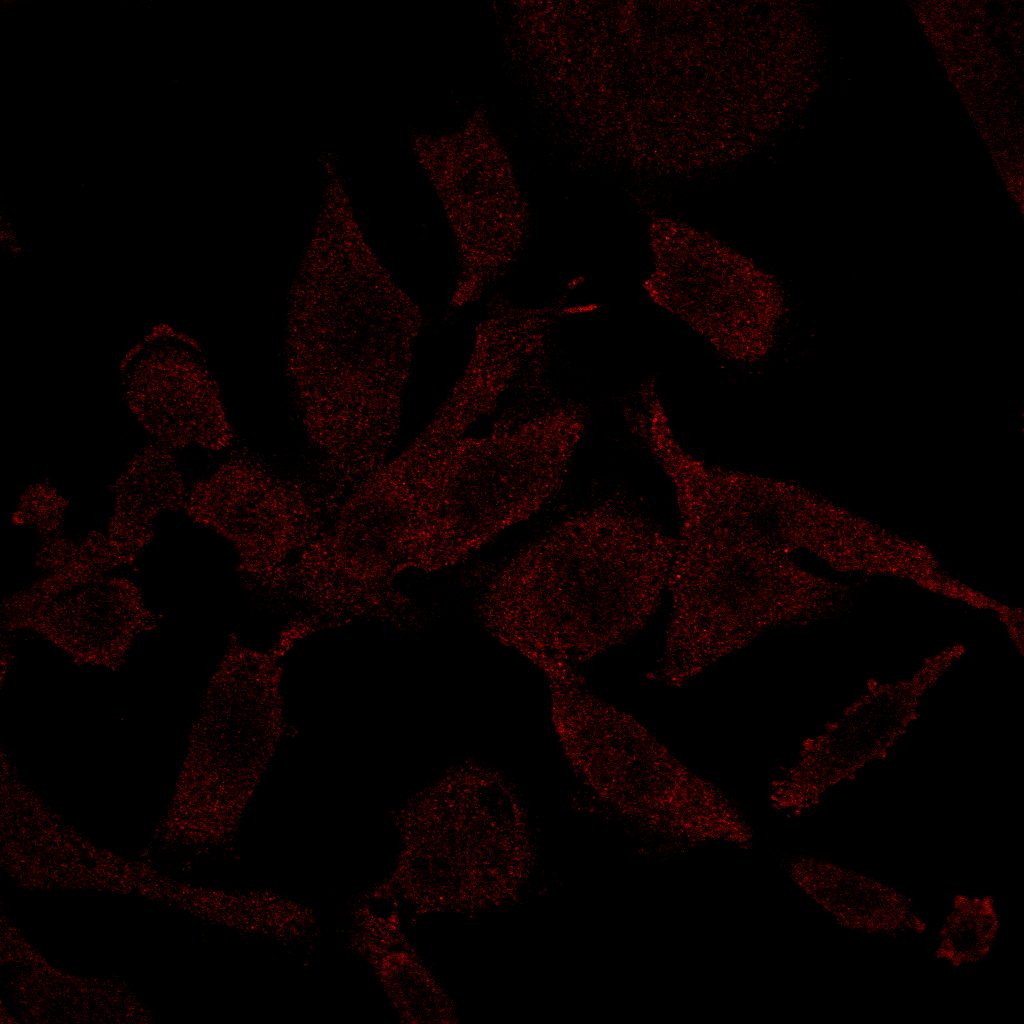

Supplement: Supplementary file 5 — Source data Fig. 4 [file 44318_2024_192_MOESM5_ESM.zip › Figure4/Figure4d/MHY1485_positive/beta-Gal.tif]

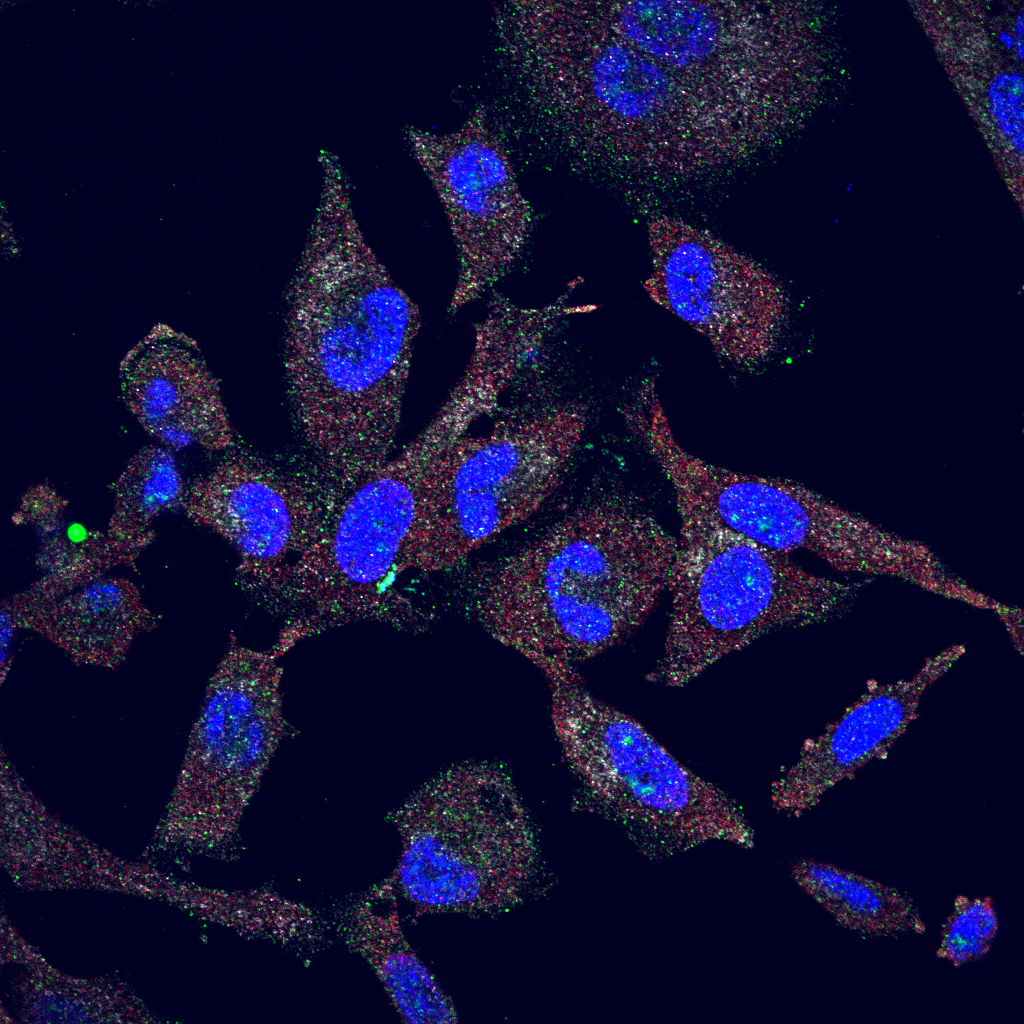

Supplement: Supplementary file 5 — Source data Fig. 4 [file 44318_2024_192_MOESM5_ESM.zip › Figure4/Figure4d/MHY1485_positive/Merge+Hoechst.tif]

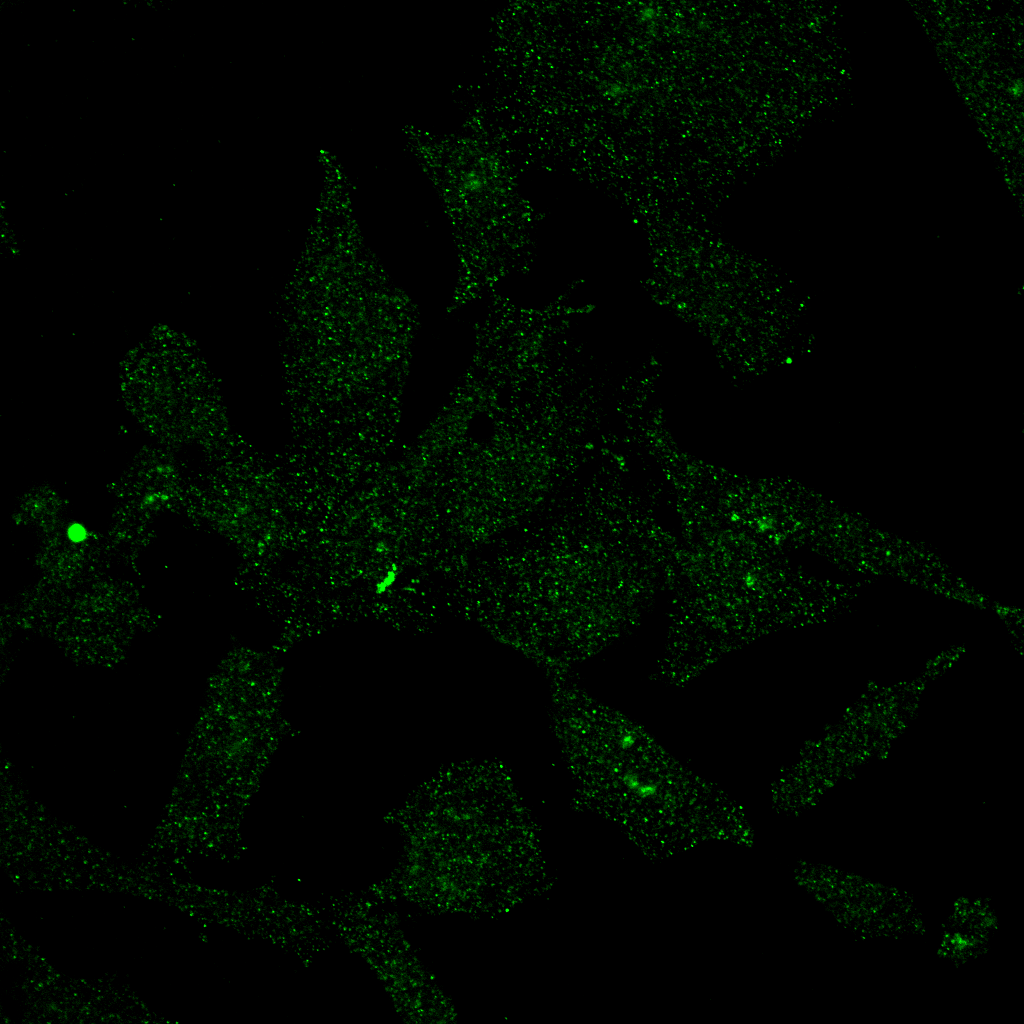

Supplement: Supplementary file 5 — Source data Fig. 4 [file 44318_2024_192_MOESM5_ESM.zip › Figure4/Figure4d/MHY1485_positive/PQBP3.tif]

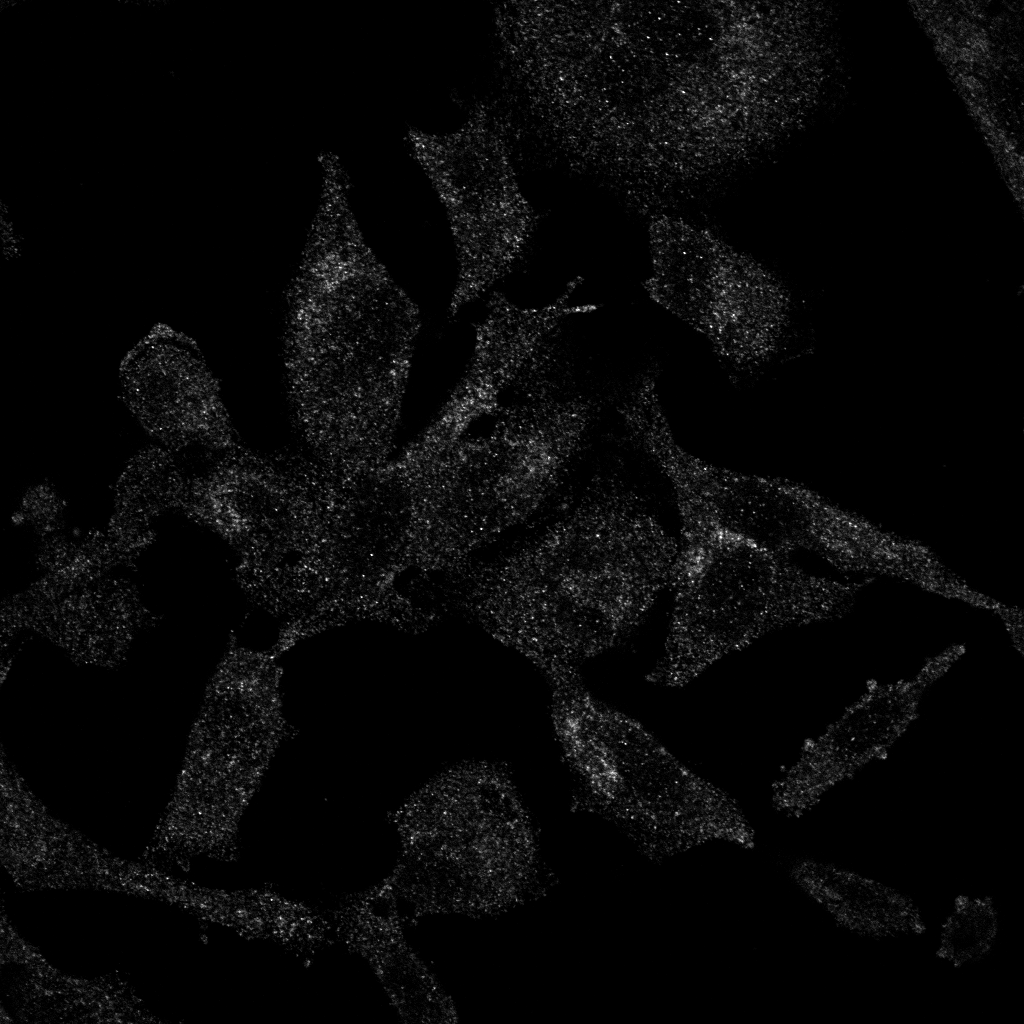

Supplement: Supplementary file 5 — Source data Fig. 4 [file 44318_2024_192_MOESM5_ESM.zip › Figure4/Figure4d/MHY1485_positive/pSer2448-mTOR.tif]

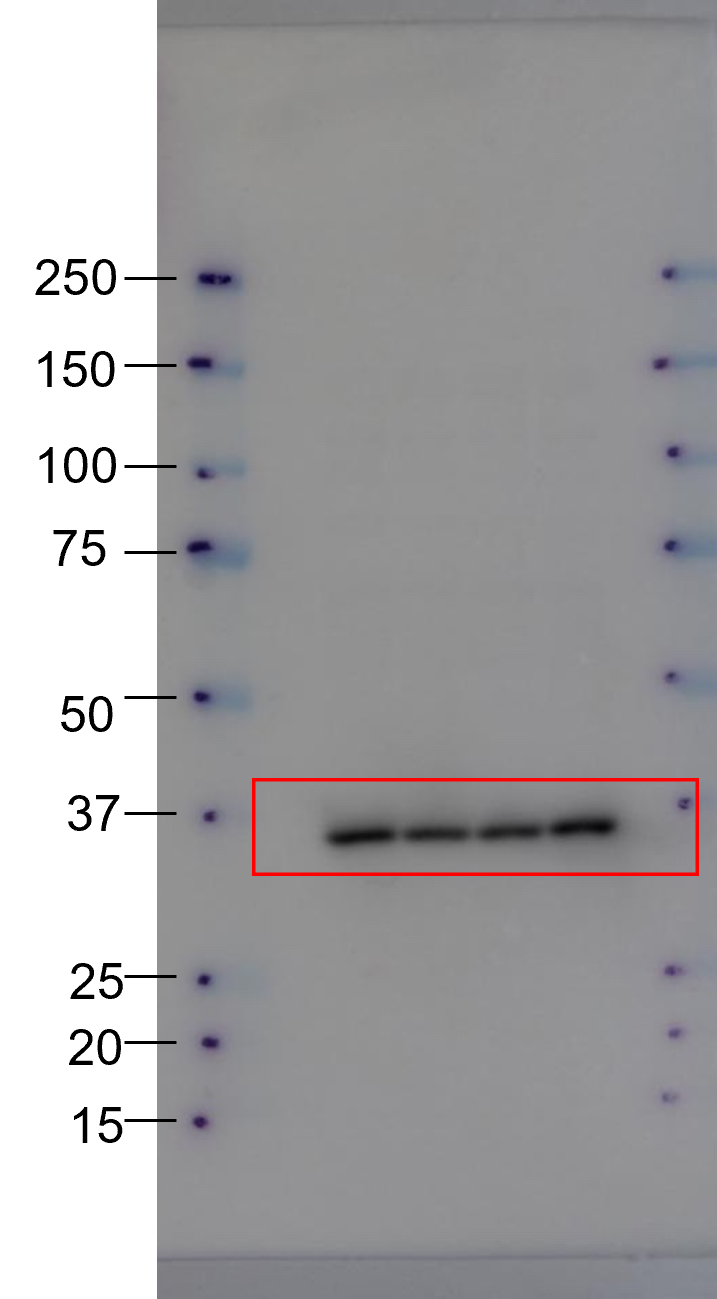

Supplement: Supplementary file 6 — Source data Fig. 5 [file 44318_2024_192_MOESM6_ESM.zip › Figure5/Figure5a/GAPDH.tif]

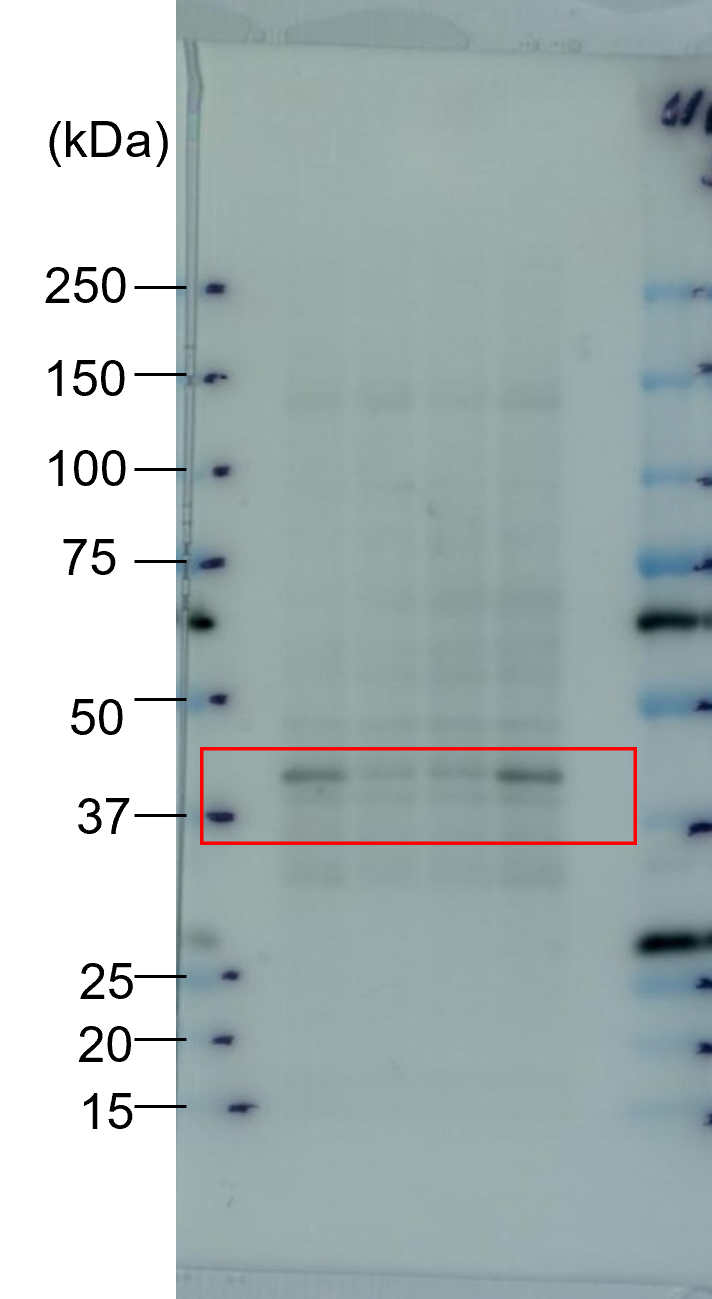

Supplement: Supplementary file 6 — Source data Fig. 5 [file 44318_2024_192_MOESM6_ESM.zip › Figure5/Figure5a/PQBP3.tif]

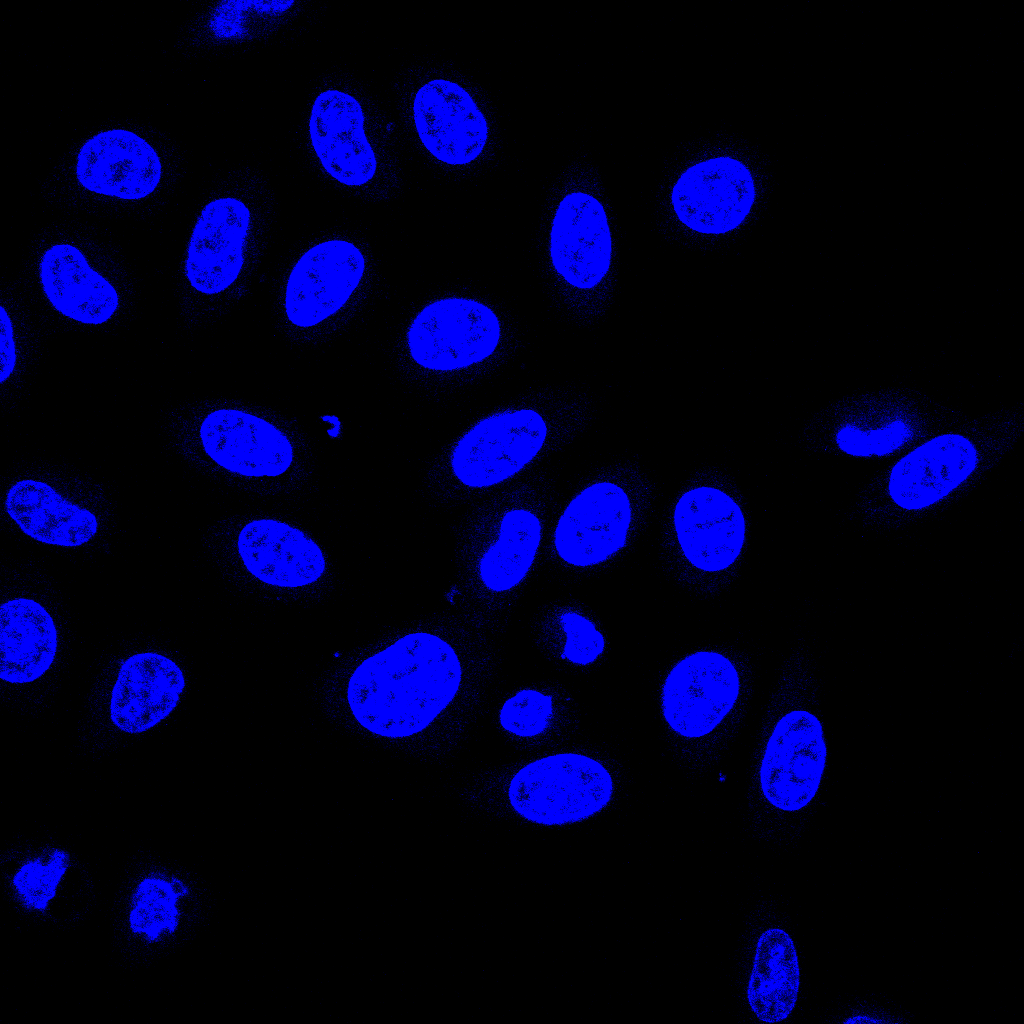

Supplement: Supplementary file 6 — Source data Fig. 5 [file 44318_2024_192_MOESM6_ESM.zip › Figure5/Figure5b/non_transfected/non-transfected_DAPI.tif]

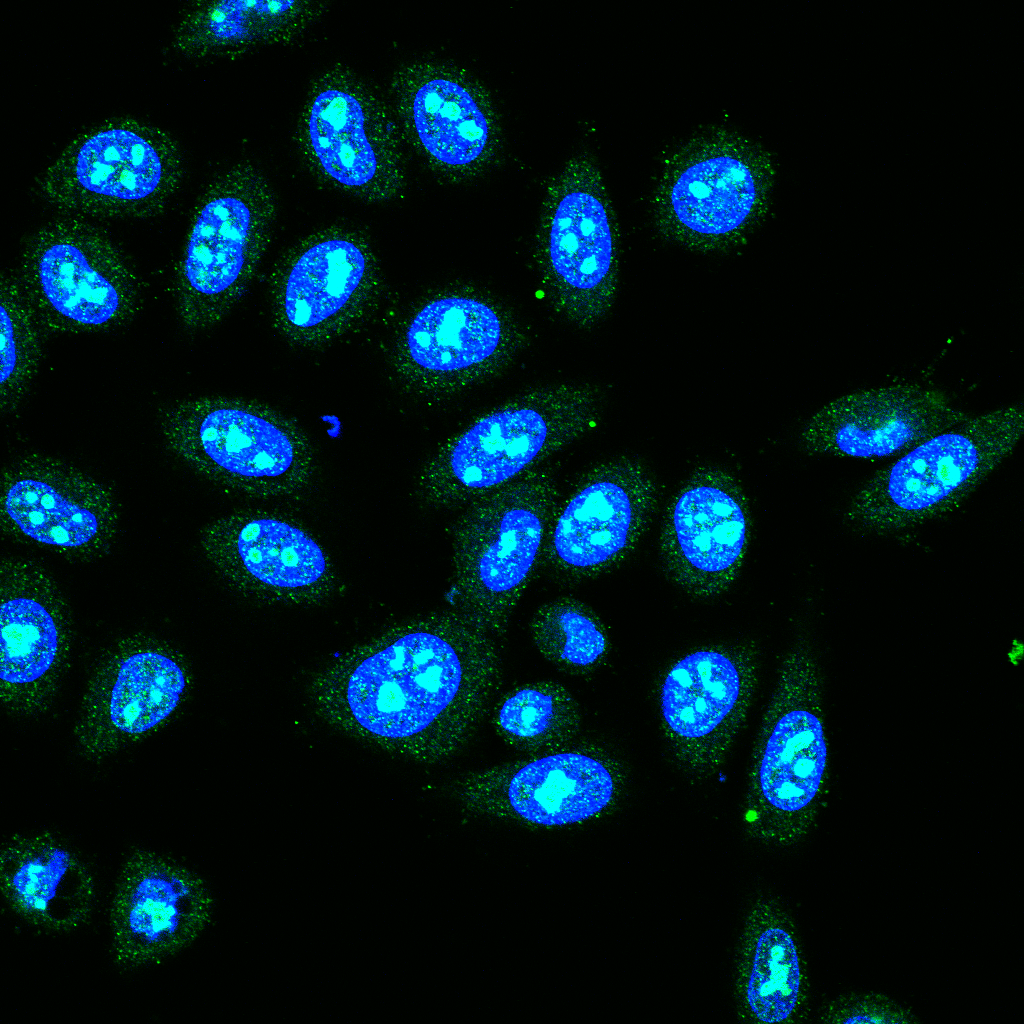

Supplement: Supplementary file 6 — Source data Fig. 5 [file 44318_2024_192_MOESM6_ESM.zip › Figure5/Figure5b/non_transfected/non-transfected_merge.tif]

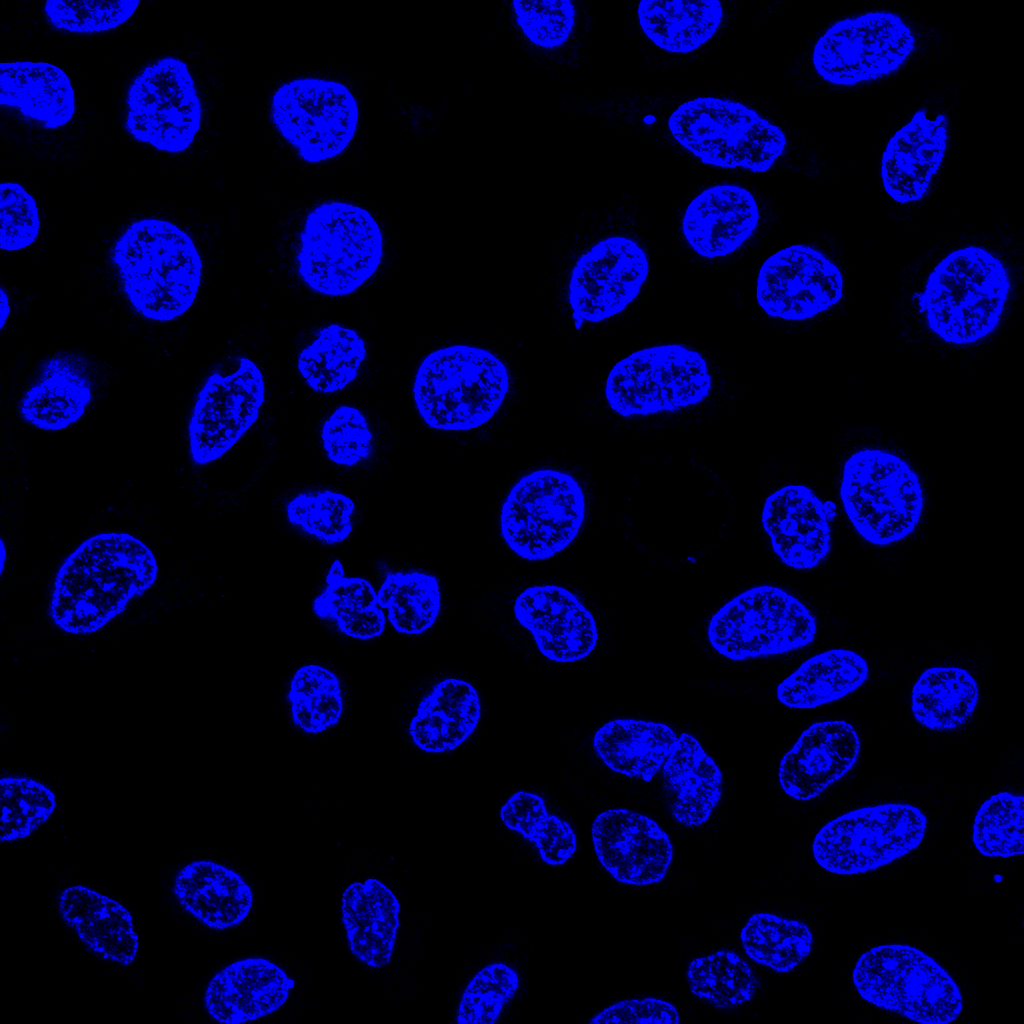

Supplement: Supplementary file 6 — Source data Fig. 5 [file 44318_2024_192_MOESM6_ESM.zip › Figure5/Figure5b/si_PQBP3#1/si-PQBP3#1_DAPI.tif]

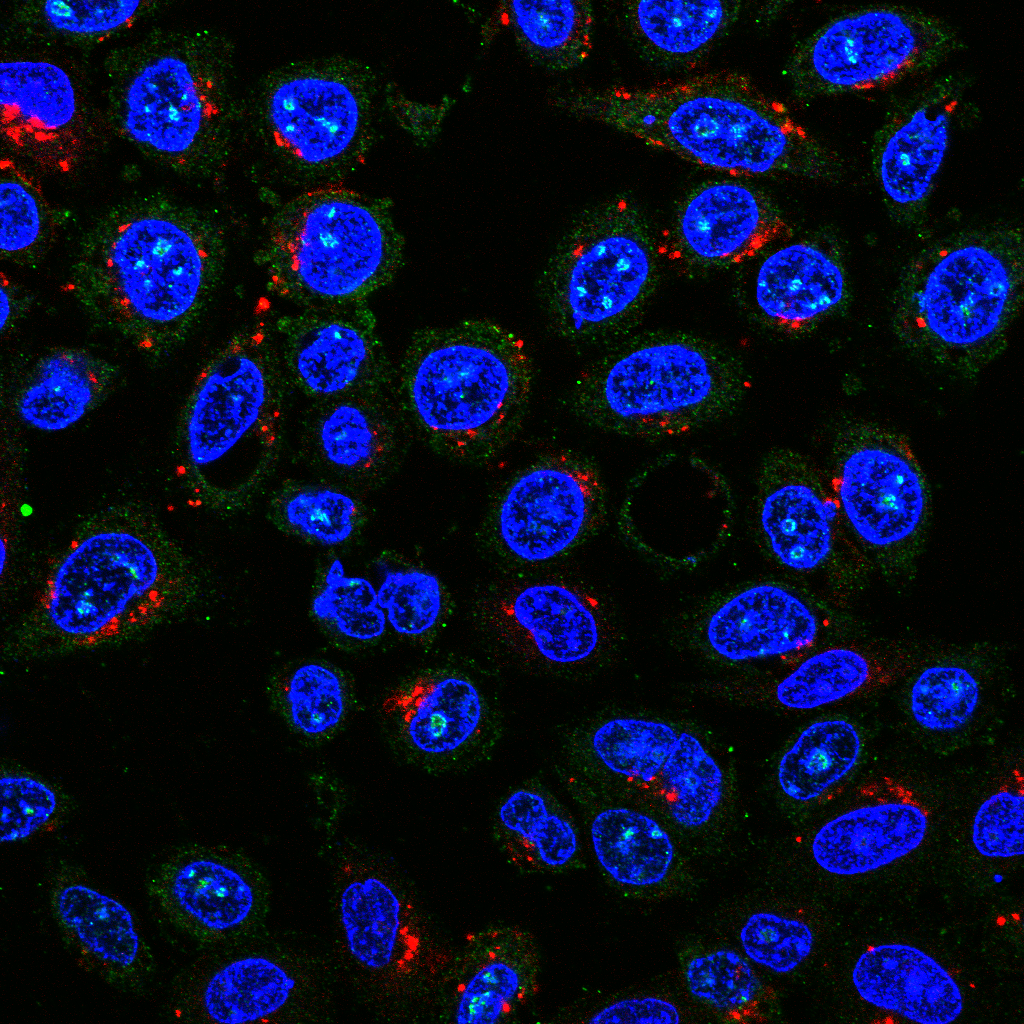

Supplement: Supplementary file 6 — Source data Fig. 5 [file 44318_2024_192_MOESM6_ESM.zip › Figure5/Figure5b/si_PQBP3#1/si-PQBP3#1_merge.tif]

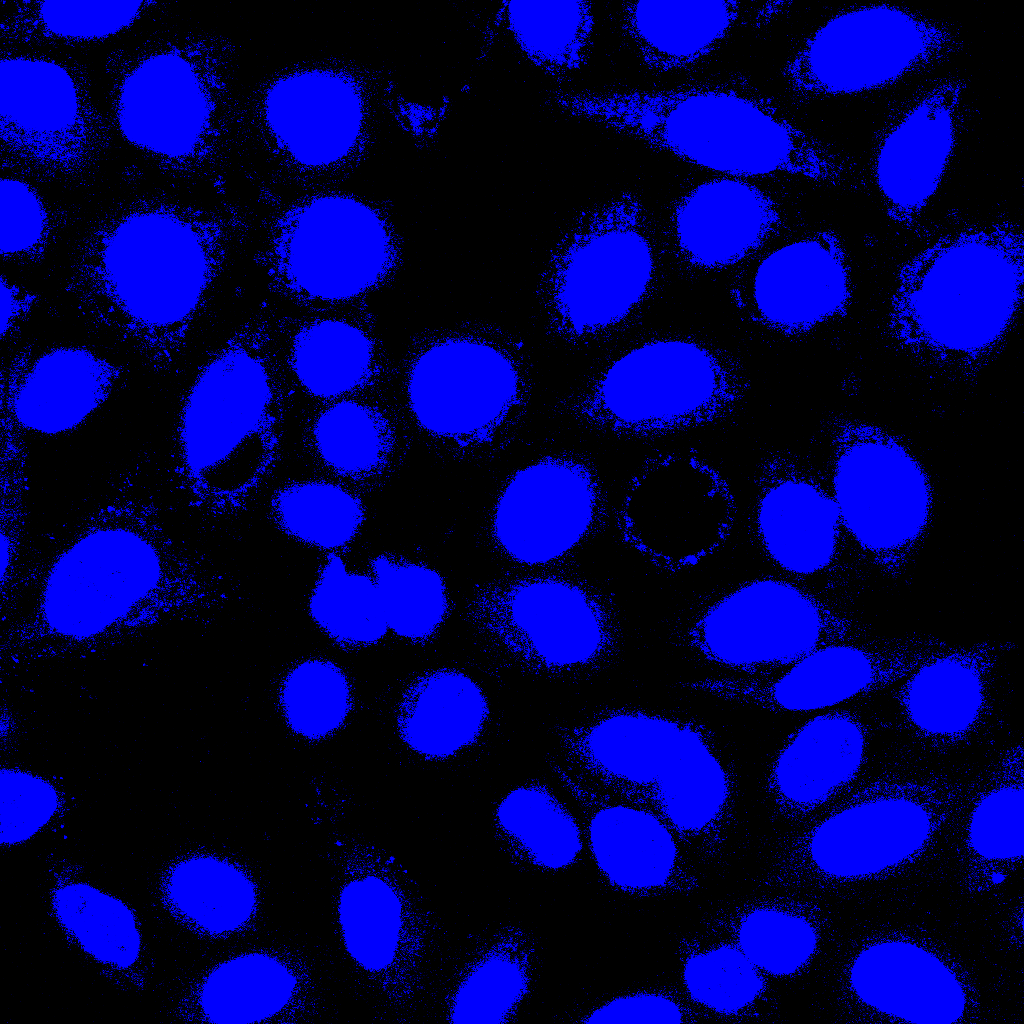

Supplement: Supplementary file 6 — Source data Fig. 5 [file 44318_2024_192_MOESM6_ESM.zip › Figure5/Figure5b/si_PQBP3#1/si-PQBP3#1_similar acquisition threshold for Hoechst 33342.tif]

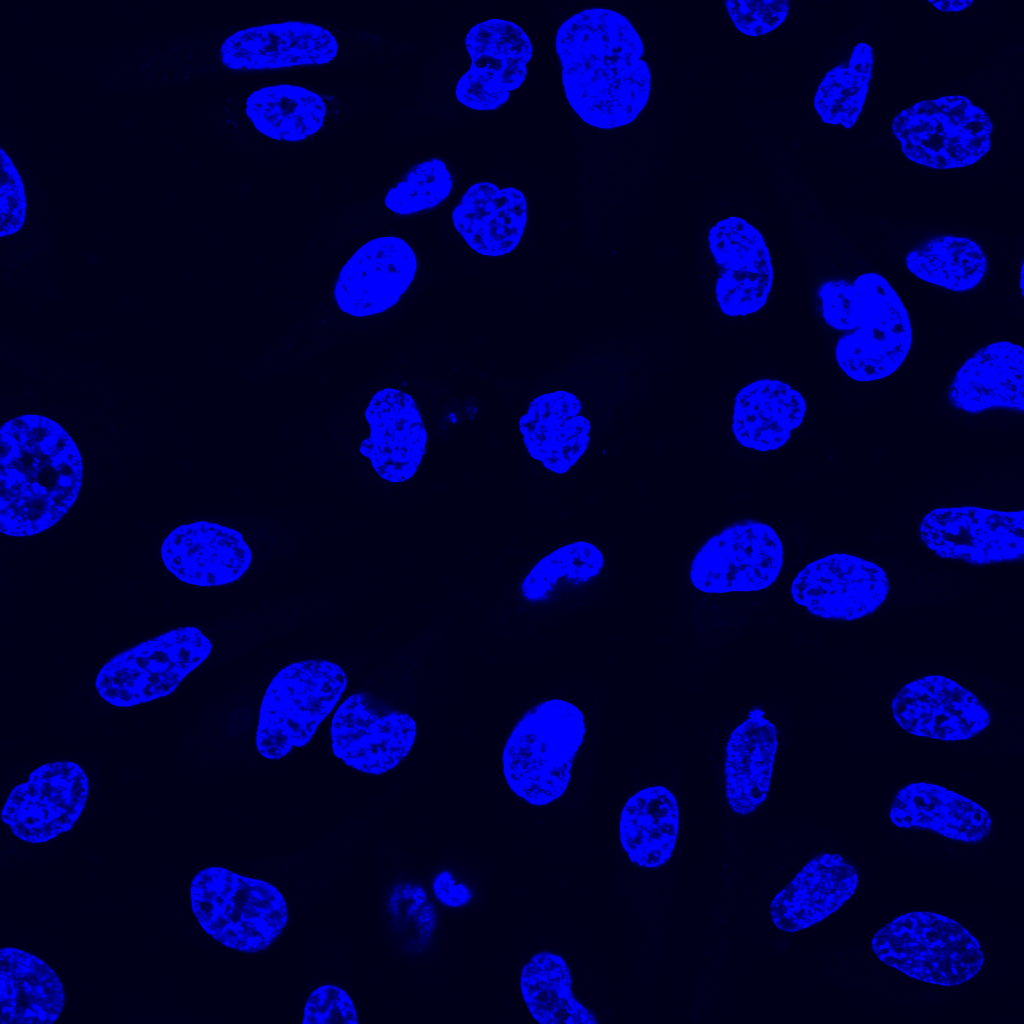

Supplement: Supplementary file 6 — Source data Fig. 5 [file 44318_2024_192_MOESM6_ESM.zip › Figure5/Figure5b/si_PQBP3#2/si-PQBP3#2_Hoechst.tif]

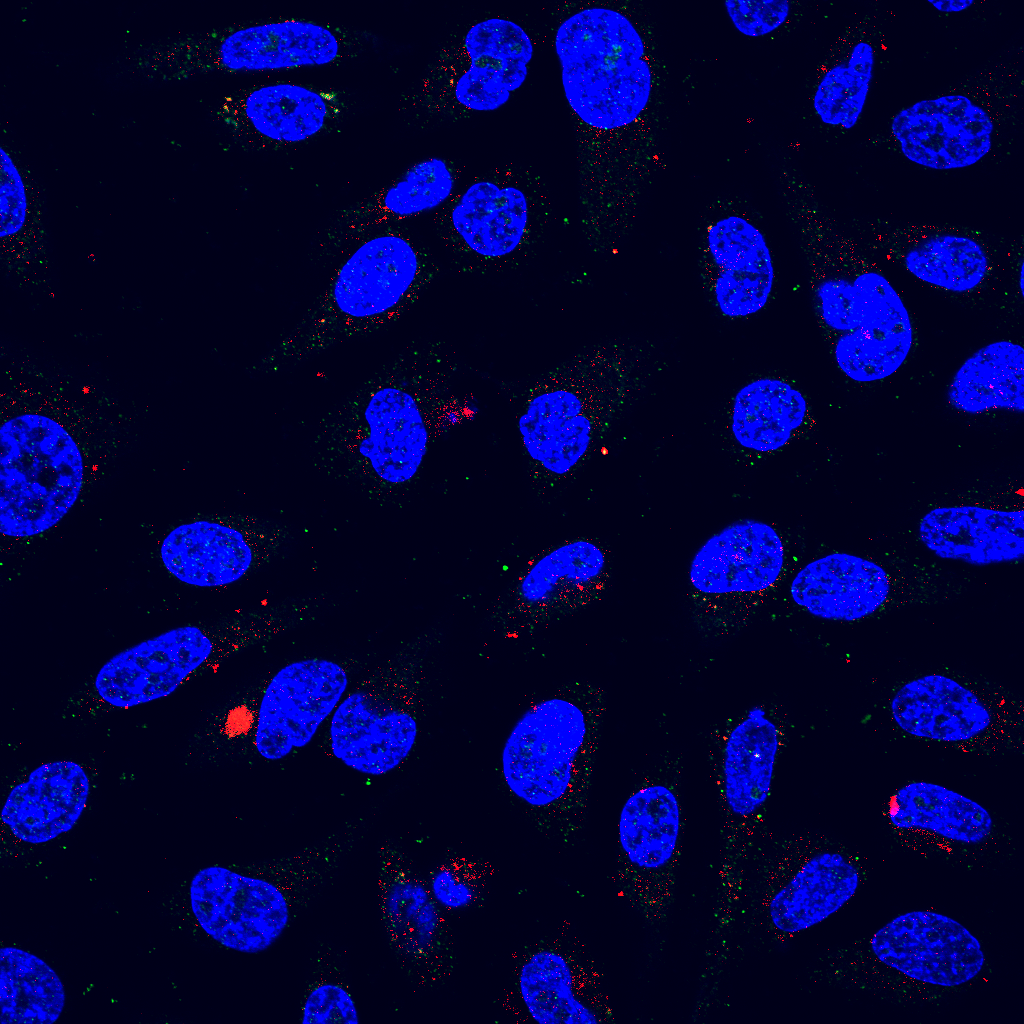

Supplement: Supplementary file 6 — Source data Fig. 5 [file 44318_2024_192_MOESM6_ESM.zip › Figure5/Figure5b/si_PQBP3#2/si-PQBP3#2_merge.tif]

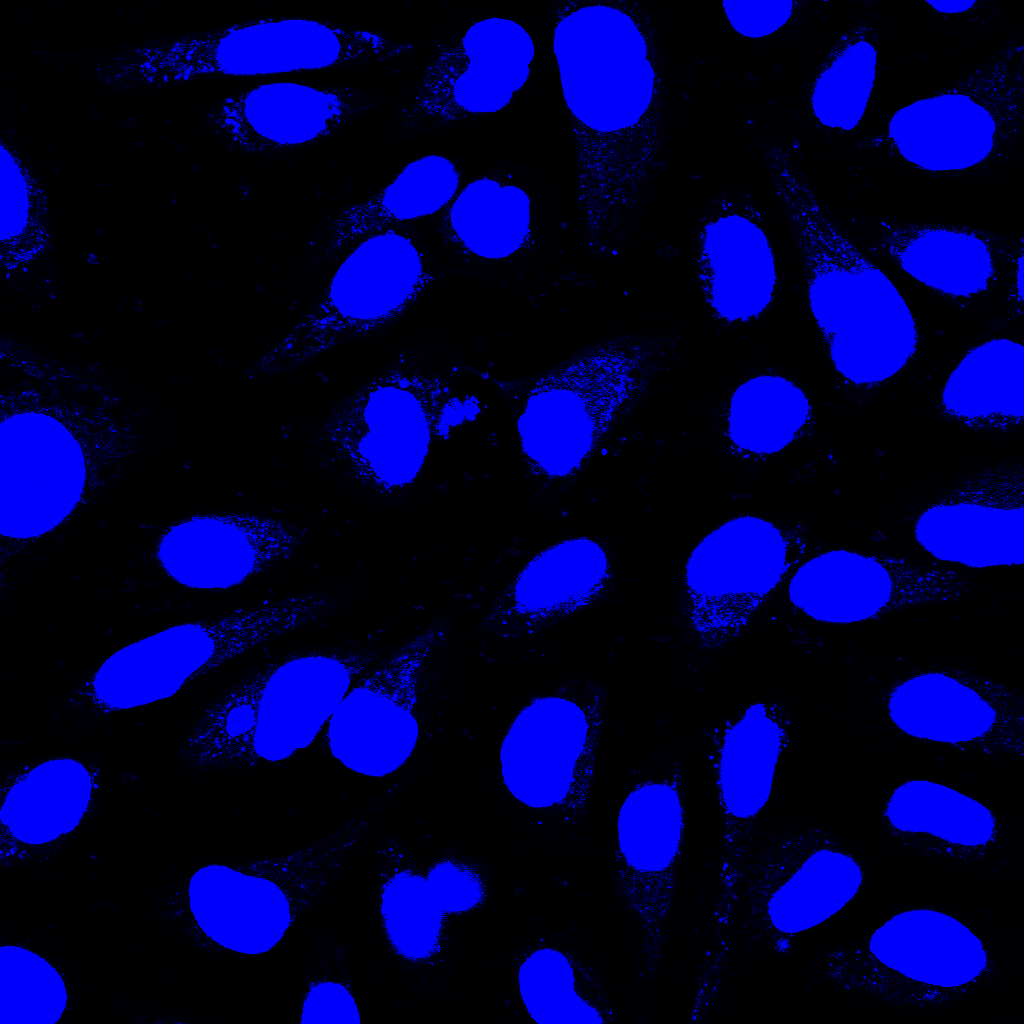

Supplement: Supplementary file 6 — Source data Fig. 5 [file 44318_2024_192_MOESM6_ESM.zip › Figure5/Figure5b/si_PQBP3#2/si-PQBP3#2_similar acquisition threshold for Hoechst 33342.tif]

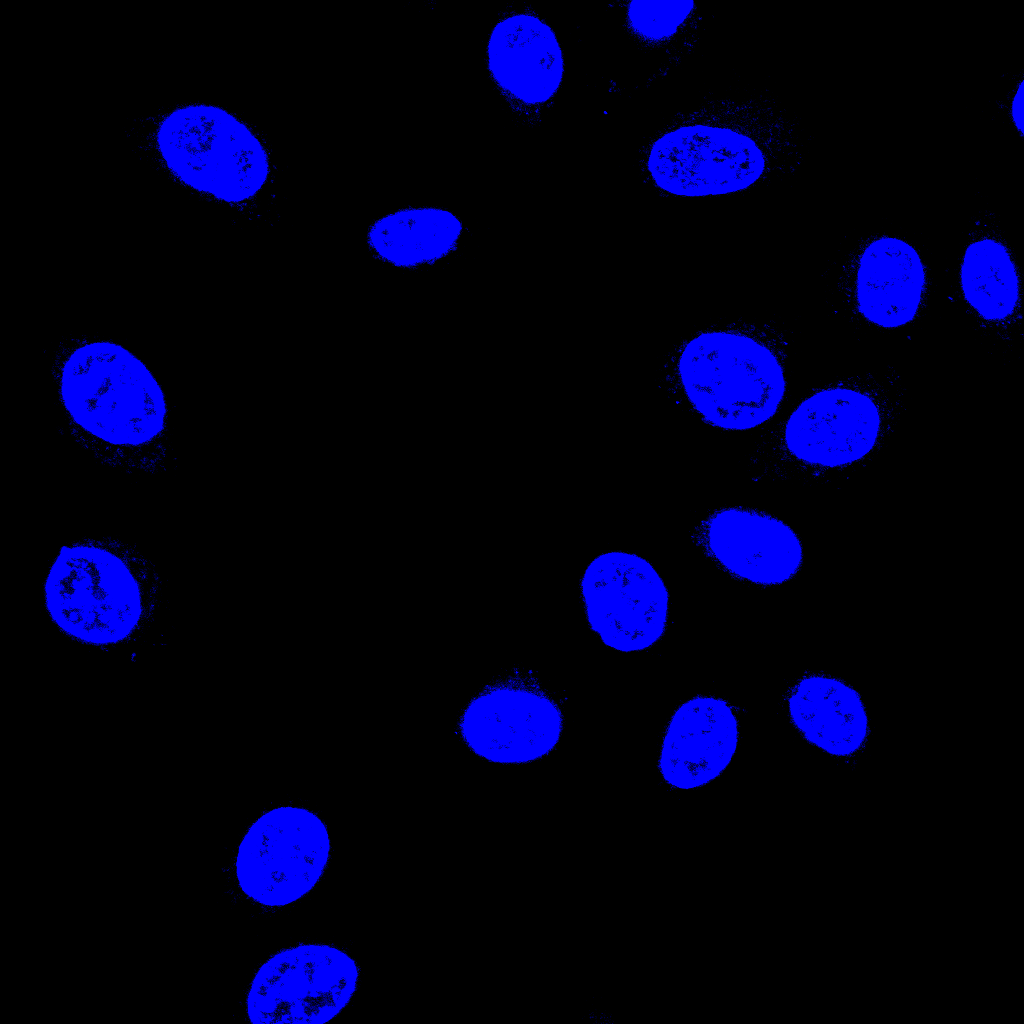

Supplement: Supplementary file 6 — Source data Fig. 5 [file 44318_2024_192_MOESM6_ESM.zip › Figure5/Figure5b/si_Scramble/si-Scramble_DAPI.tif]

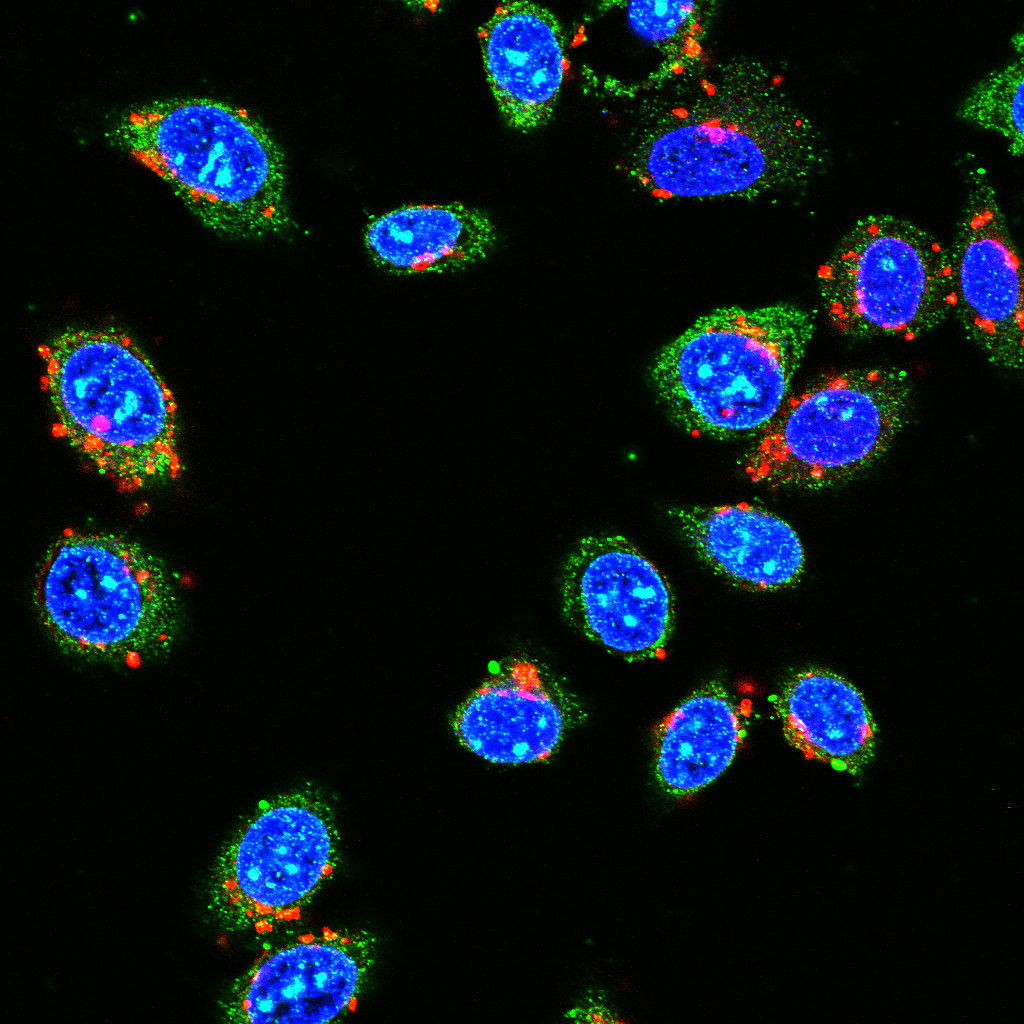

Supplement: Supplementary file 6 — Source data Fig. 5 [file 44318_2024_192_MOESM6_ESM.zip › Figure5/Figure5b/si_Scramble/si-Scramble_merge .tif]

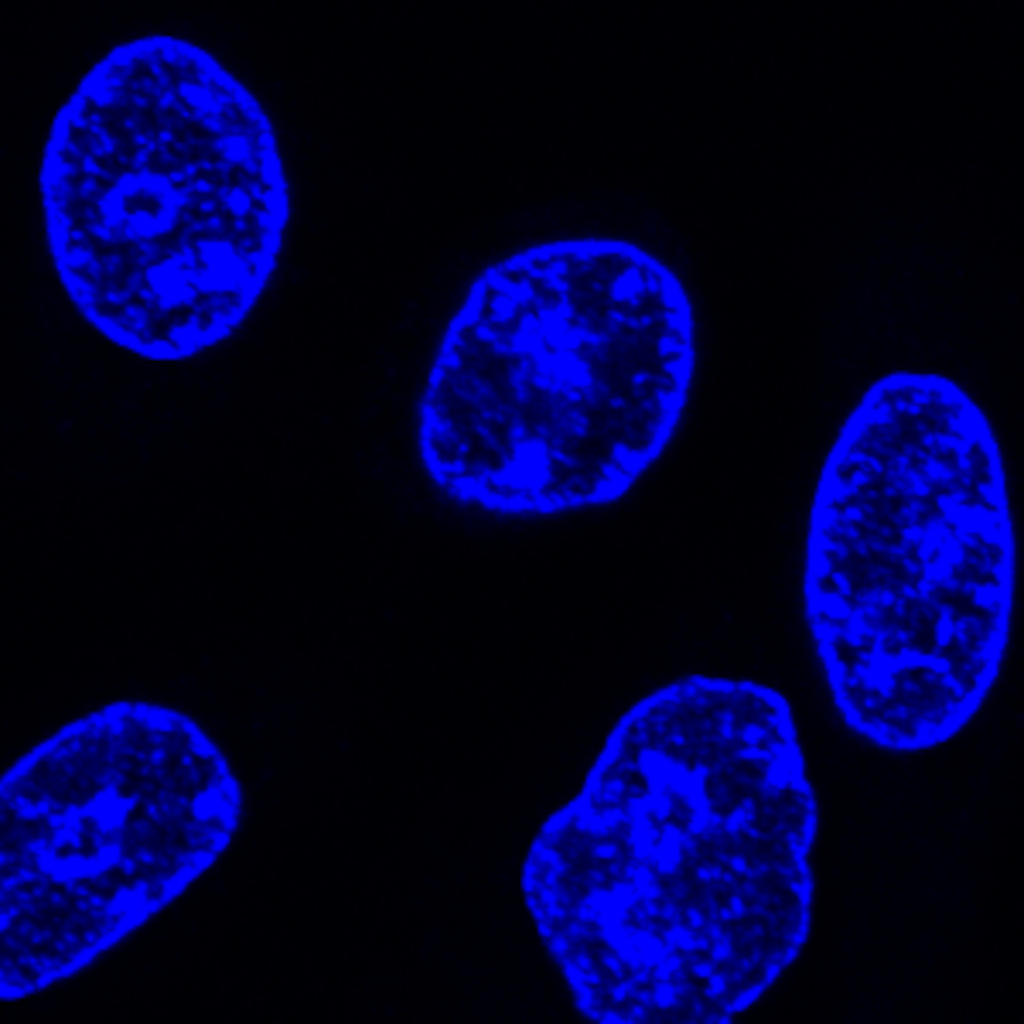

Supplement: Supplementary file 6 — Source data Fig. 5 [file 44318_2024_192_MOESM6_ESM.zip › Figure5/Figure5c/non-transfected.tif]

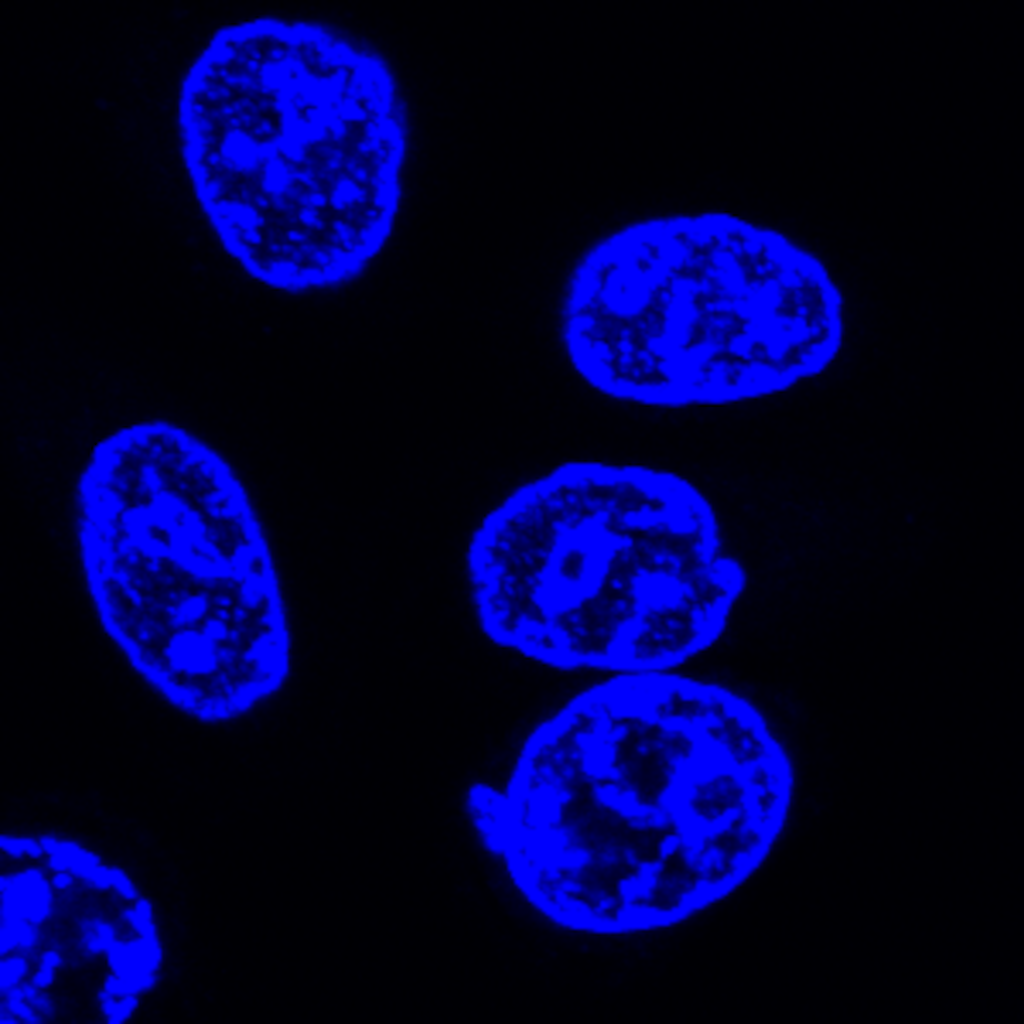

Supplement: Supplementary file 6 — Source data Fig. 5 [file 44318_2024_192_MOESM6_ESM.zip › Figure5/Figure5c/si-PQBP3#1.tif]

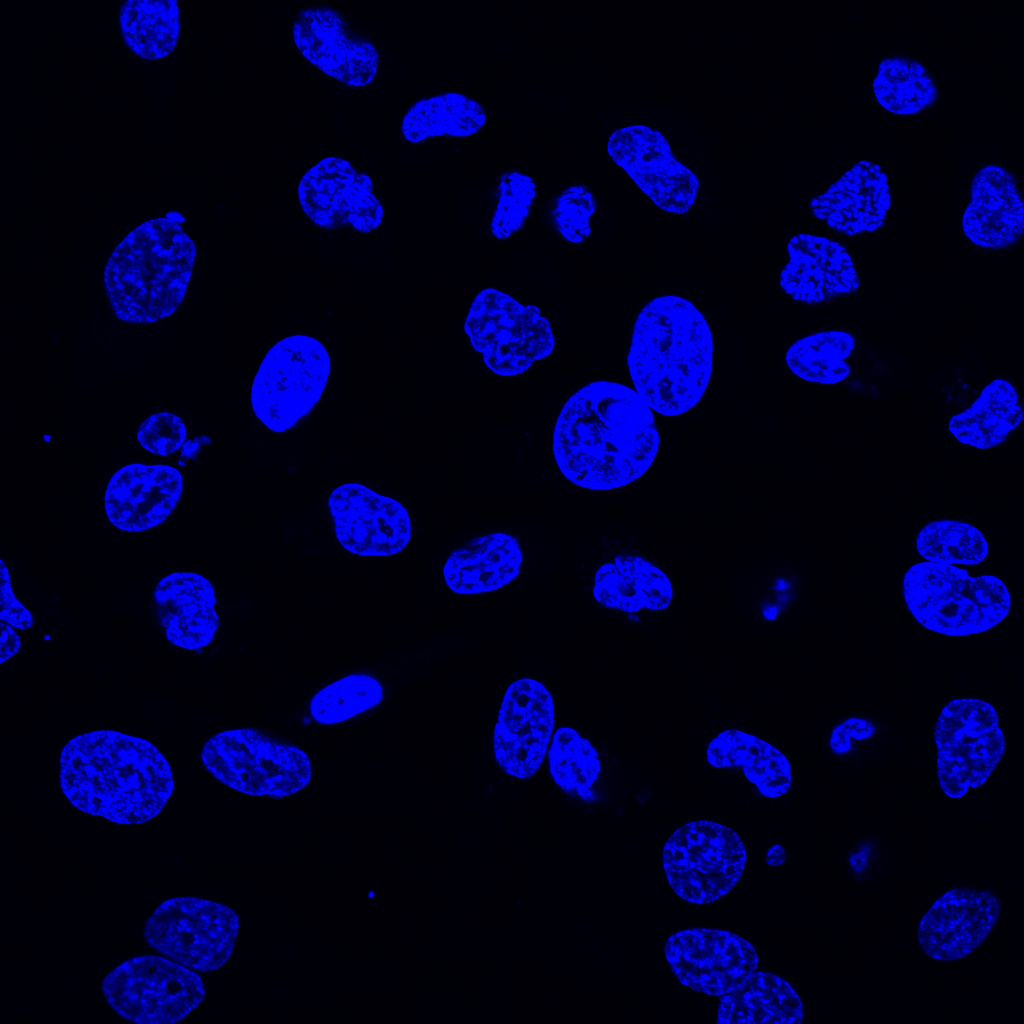

Supplement: Supplementary file 6 — Source data Fig. 5 [file 44318_2024_192_MOESM6_ESM.zip › Figure5/Figure5c/si-PQBP3#2.tif]

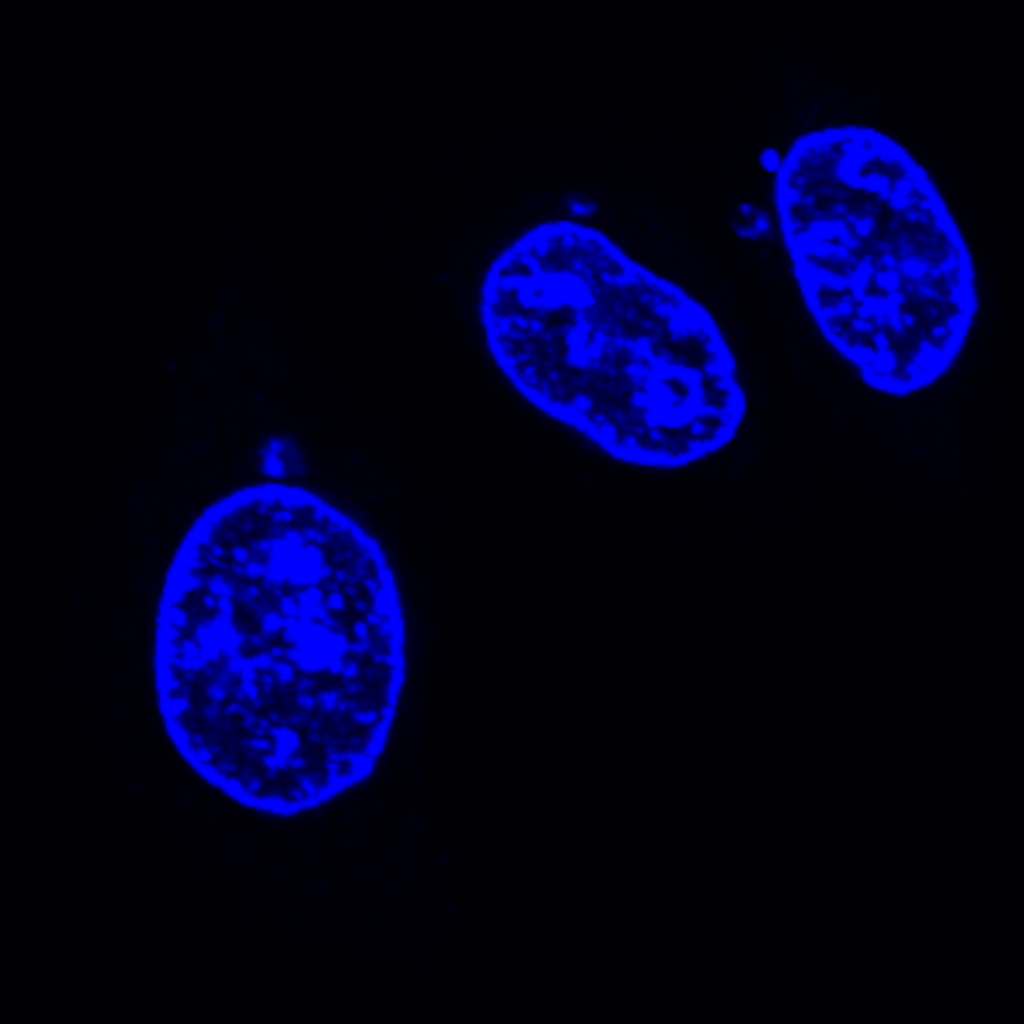

Supplement: Supplementary file 6 — Source data Fig. 5 [file 44318_2024_192_MOESM6_ESM.zip › Figure5/Figure5c/si-Scramble.tif]

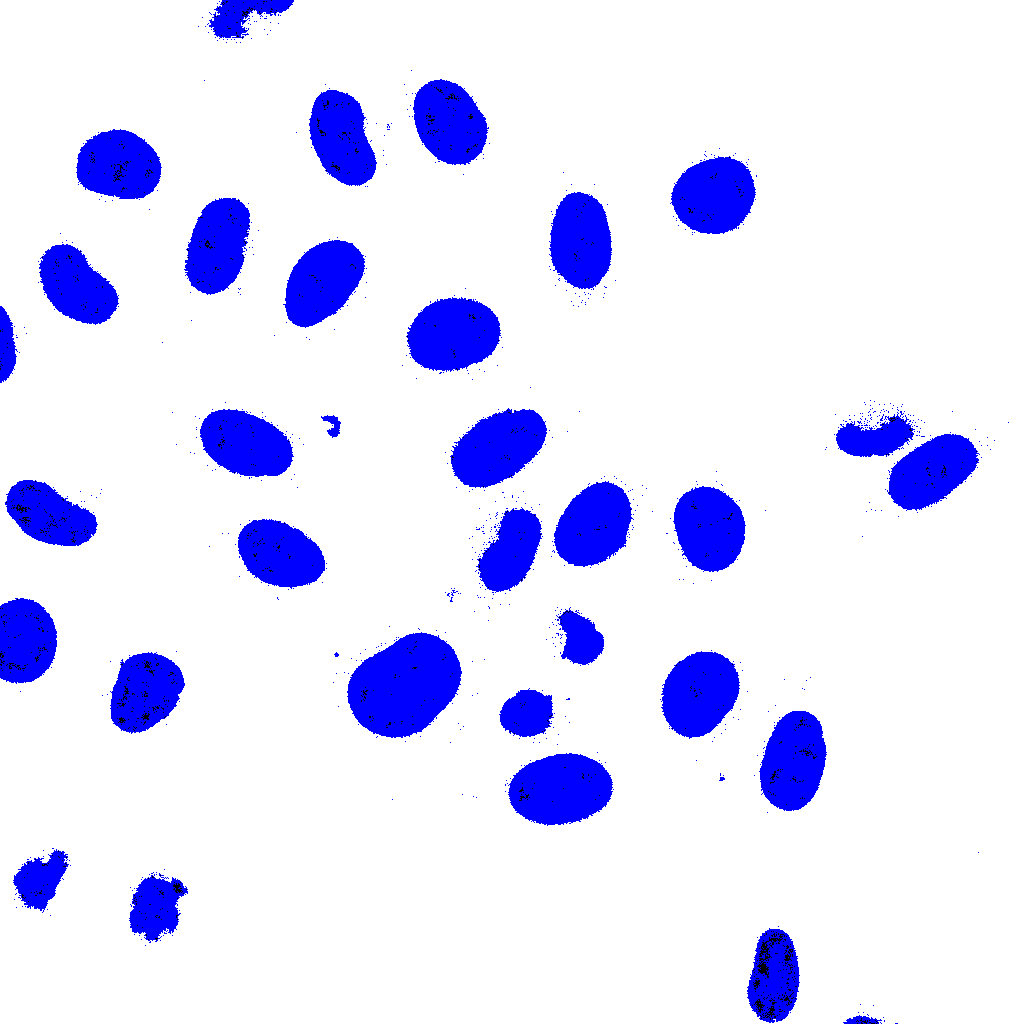

Supplement: Supplementary file 6 — Source data Fig. 5 [file 44318_2024_192_MOESM6_ESM.zip › Figure5/Figure5e/non-transfected.tif]

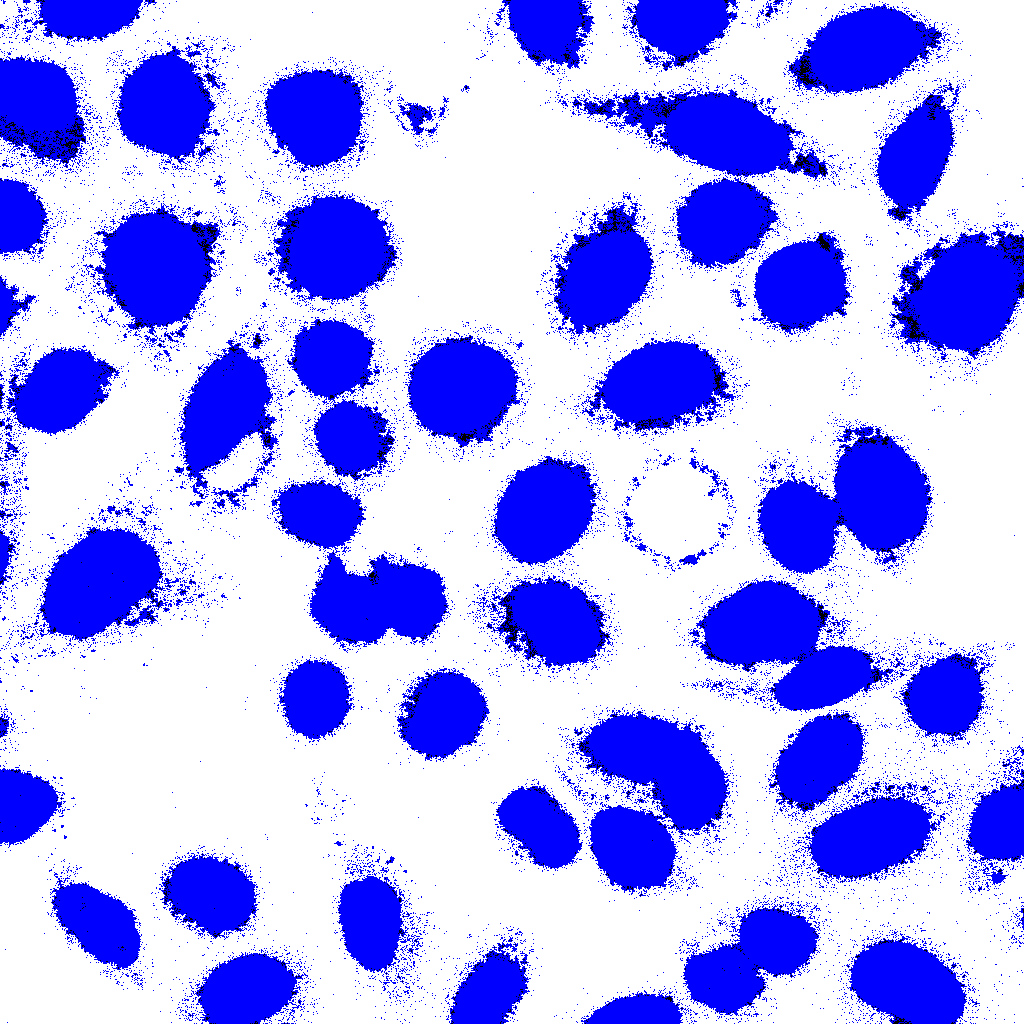

Supplement: Supplementary file 6 — Source data Fig. 5 [file 44318_2024_192_MOESM6_ESM.zip › Figure5/Figure5e/si-PQBP3#1.tif]

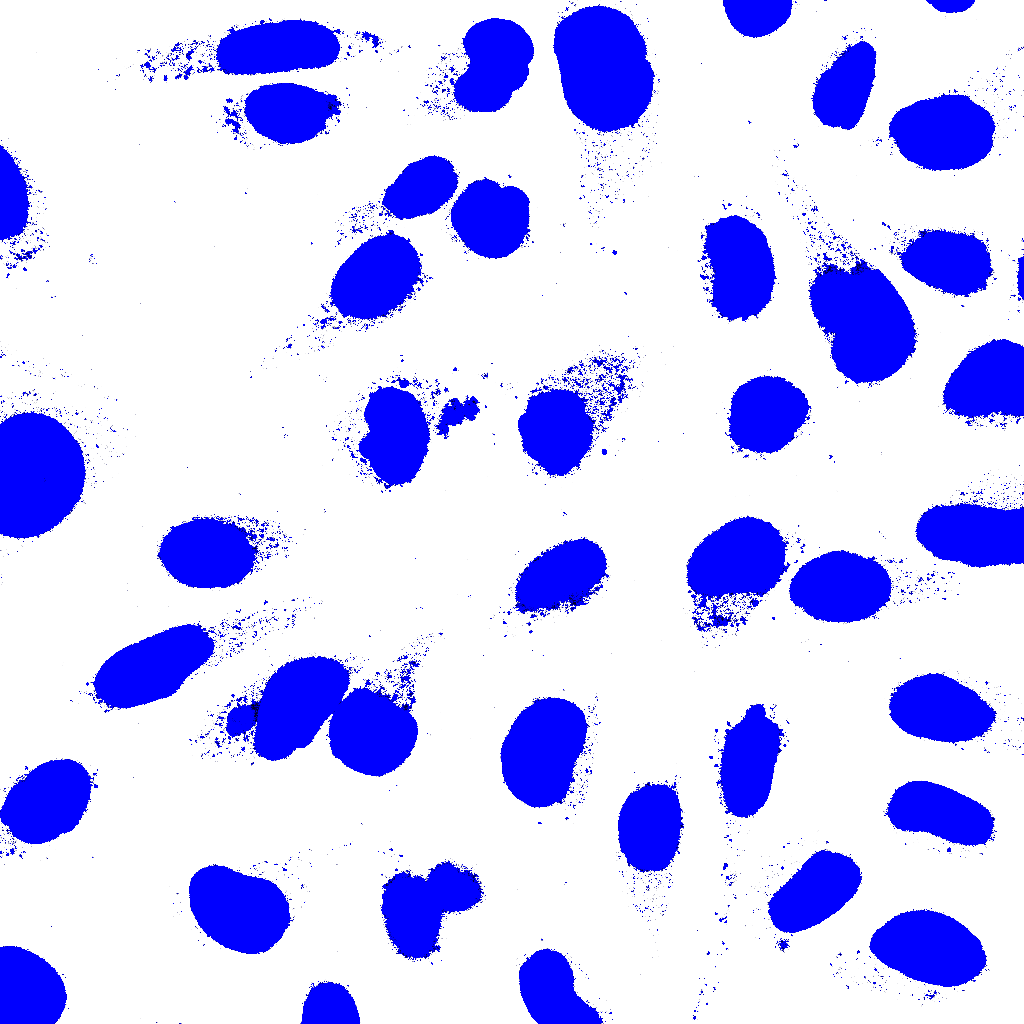

Supplement: Supplementary file 6 — Source data Fig. 5 [file 44318_2024_192_MOESM6_ESM.zip › Figure5/Figure5e/si-PQBP3#2.tif]

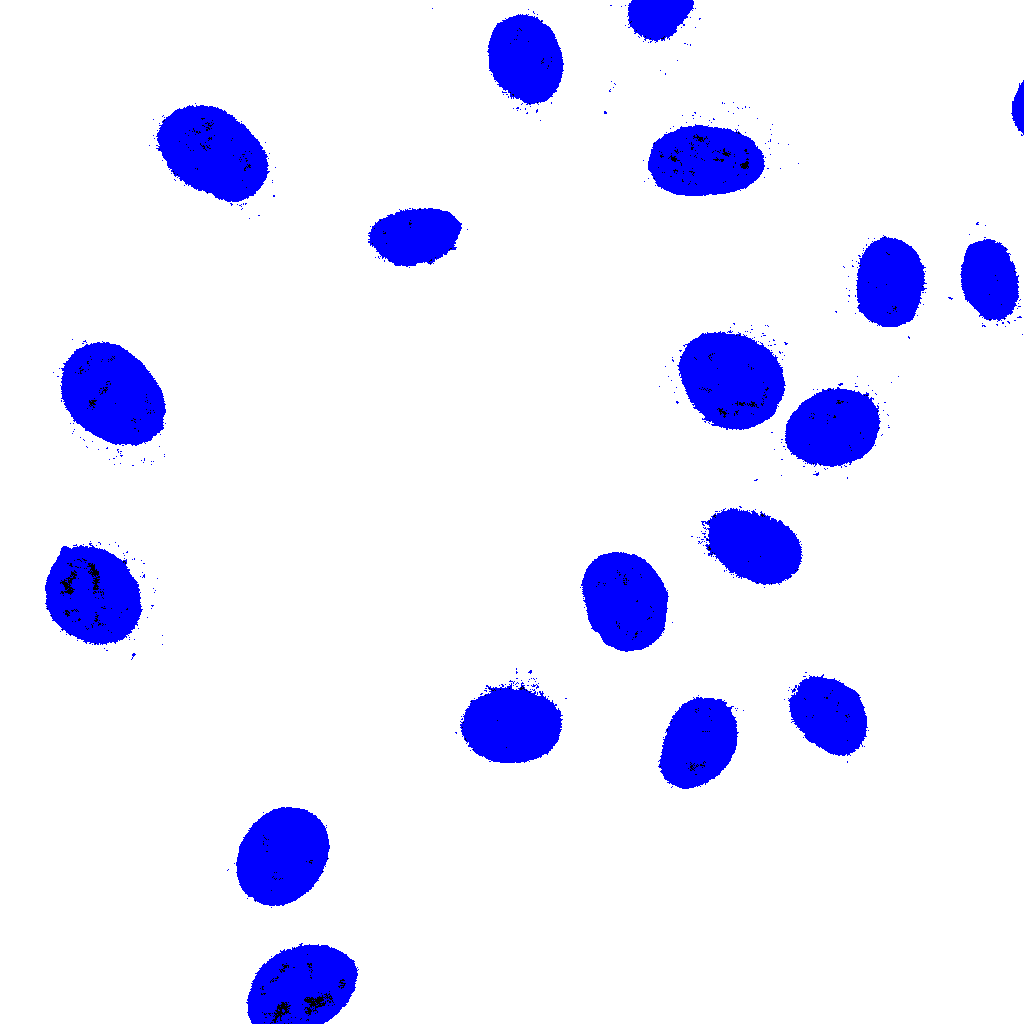

Supplement: Supplementary file 6 — Source data Fig. 5 [file 44318_2024_192_MOESM6_ESM.zip › Figure5/Figure5e/si-Scramble.tif]

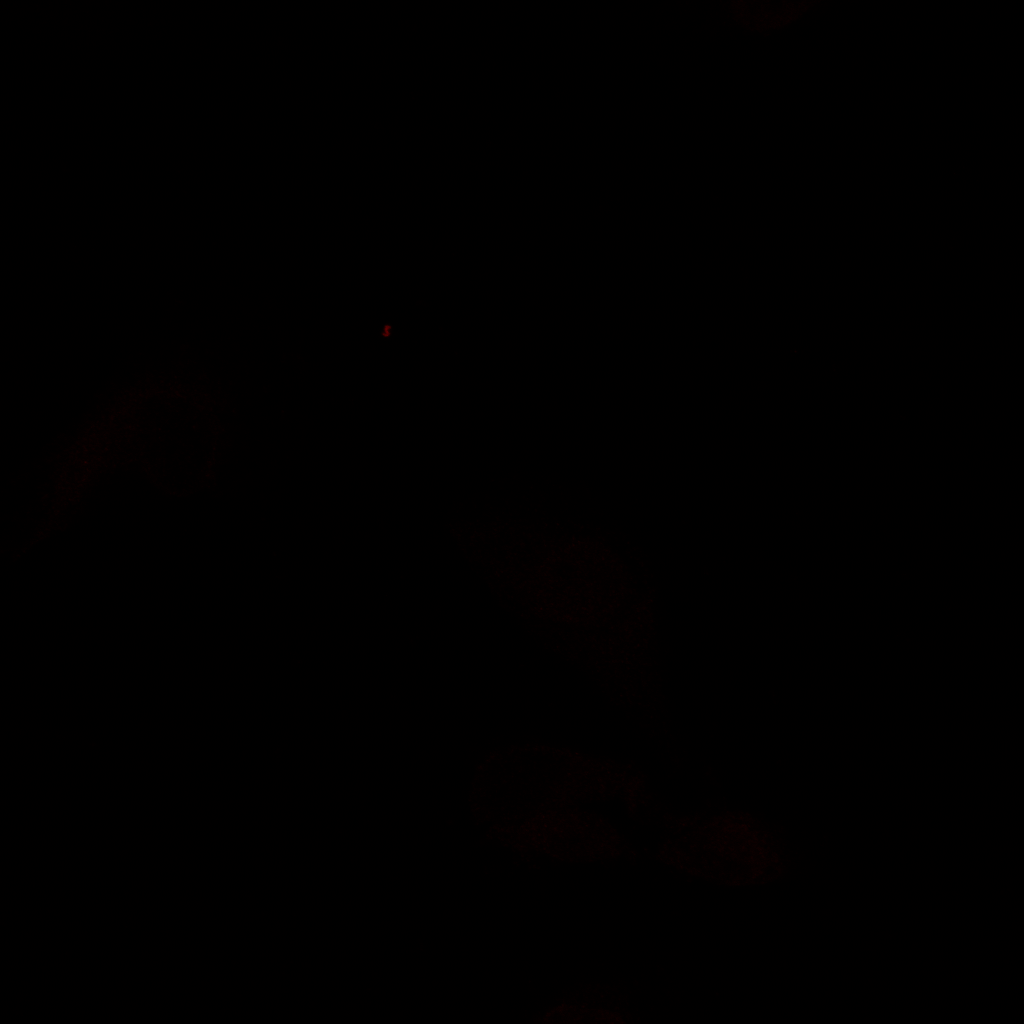

Supplement: Supplementary file 7 — Source data Fig. 6 [file 44318_2024_192_MOESM7_ESM.zip › Figure6/Figure6a/H2O2_negative/beta-Gal.tif]

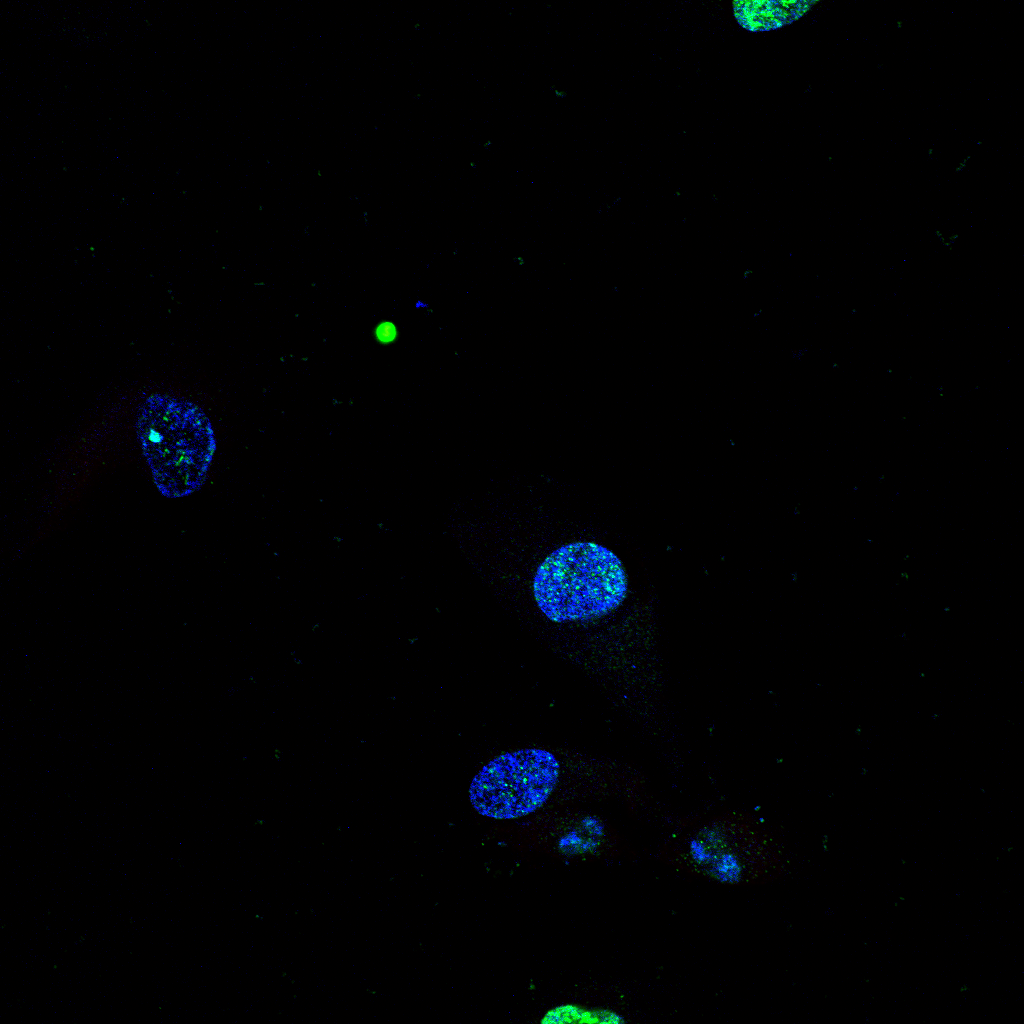

Supplement: Supplementary file 7 — Source data Fig. 6 [file 44318_2024_192_MOESM7_ESM.zip › Figure6/Figure6a/H2O2_negative/H3K9me3+Hoechst+beta-Gal.tif]

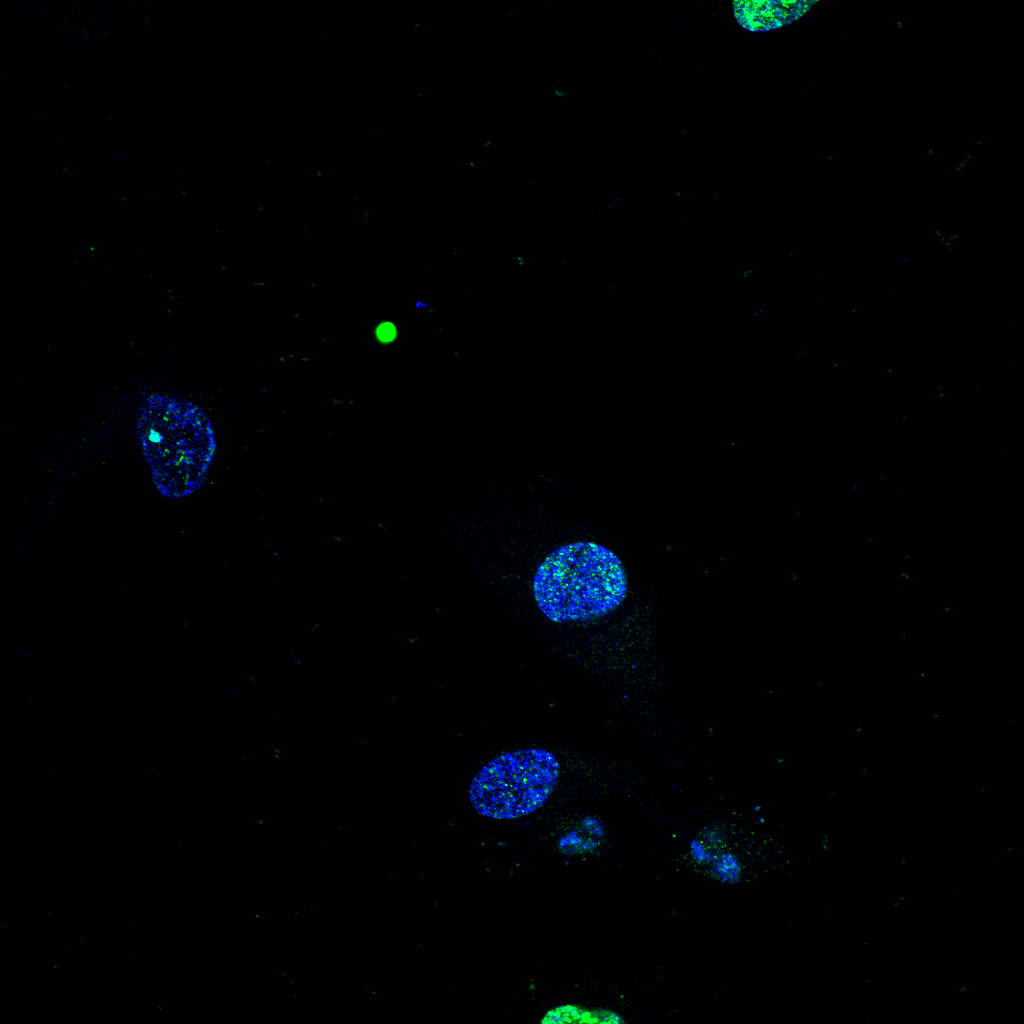

Supplement: Supplementary file 7 — Source data Fig. 6 [file 44318_2024_192_MOESM7_ESM.zip › Figure6/Figure6a/H2O2_negative/H3K9me3+Hoechst.tif]

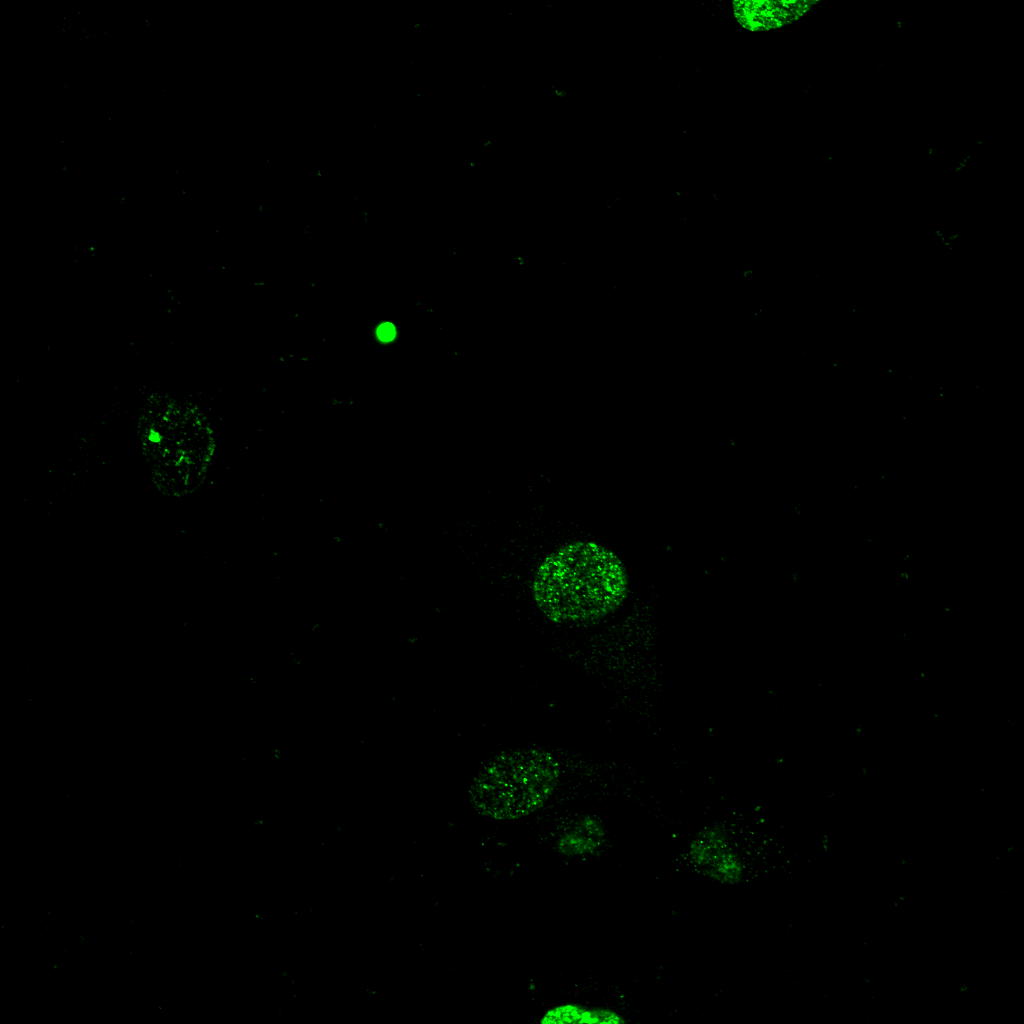

Supplement: Supplementary file 7 — Source data Fig. 6 [file 44318_2024_192_MOESM7_ESM.zip › Figure6/Figure6a/H2O2_negative/H3K9me3.tif]

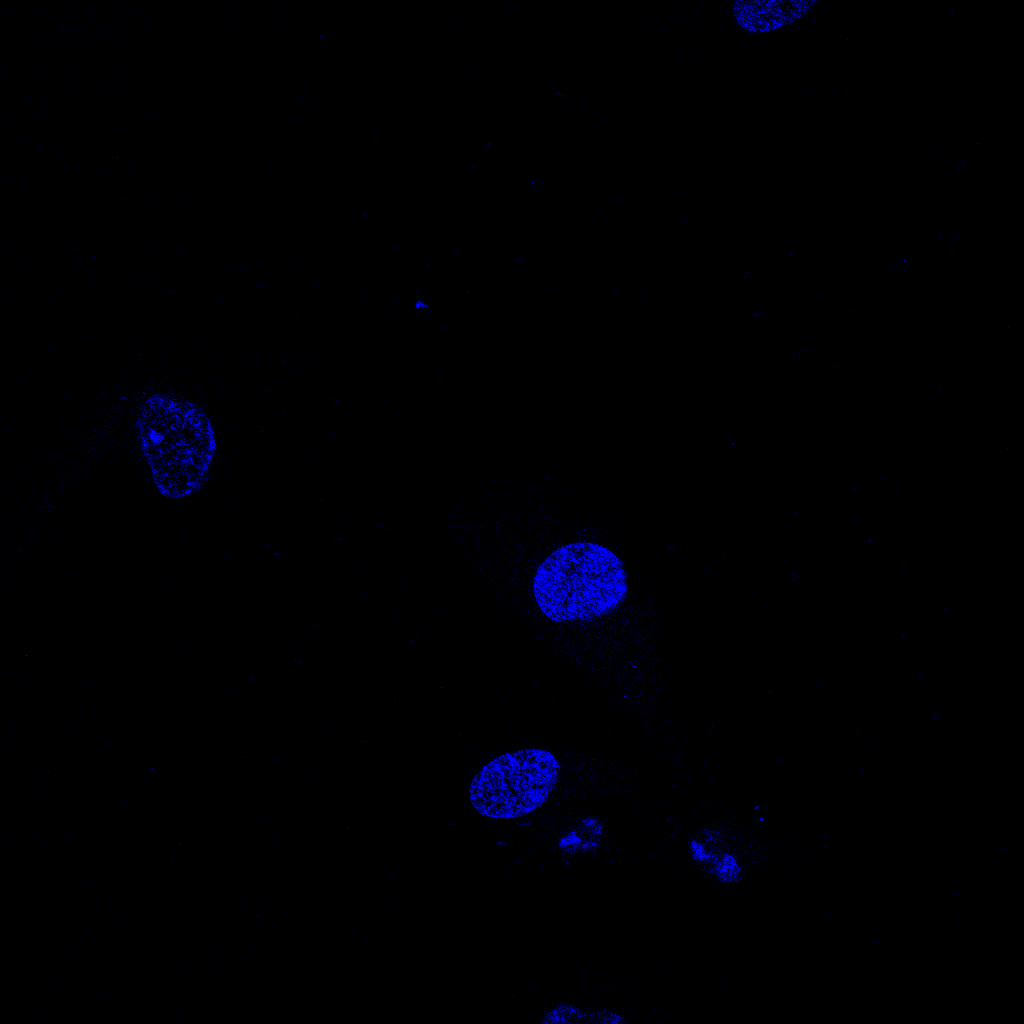

Supplement: Supplementary file 7 — Source data Fig. 6 [file 44318_2024_192_MOESM7_ESM.zip › Figure6/Figure6a/H2O2_negative/Hoechst.tif]
