# Supplementary figures and images for: PQBP3 prevents senescence by suppressing PSME3-mediated proteasomal Lamin B1 degradation (part 2 of 4)
Source: EMBO J. 2024 Aug 5;43(18):3968–99. doi: 10.1038/s44318-024-00192-4 (PMC11405525; doi:10.1038/s44318-024-00192-4)

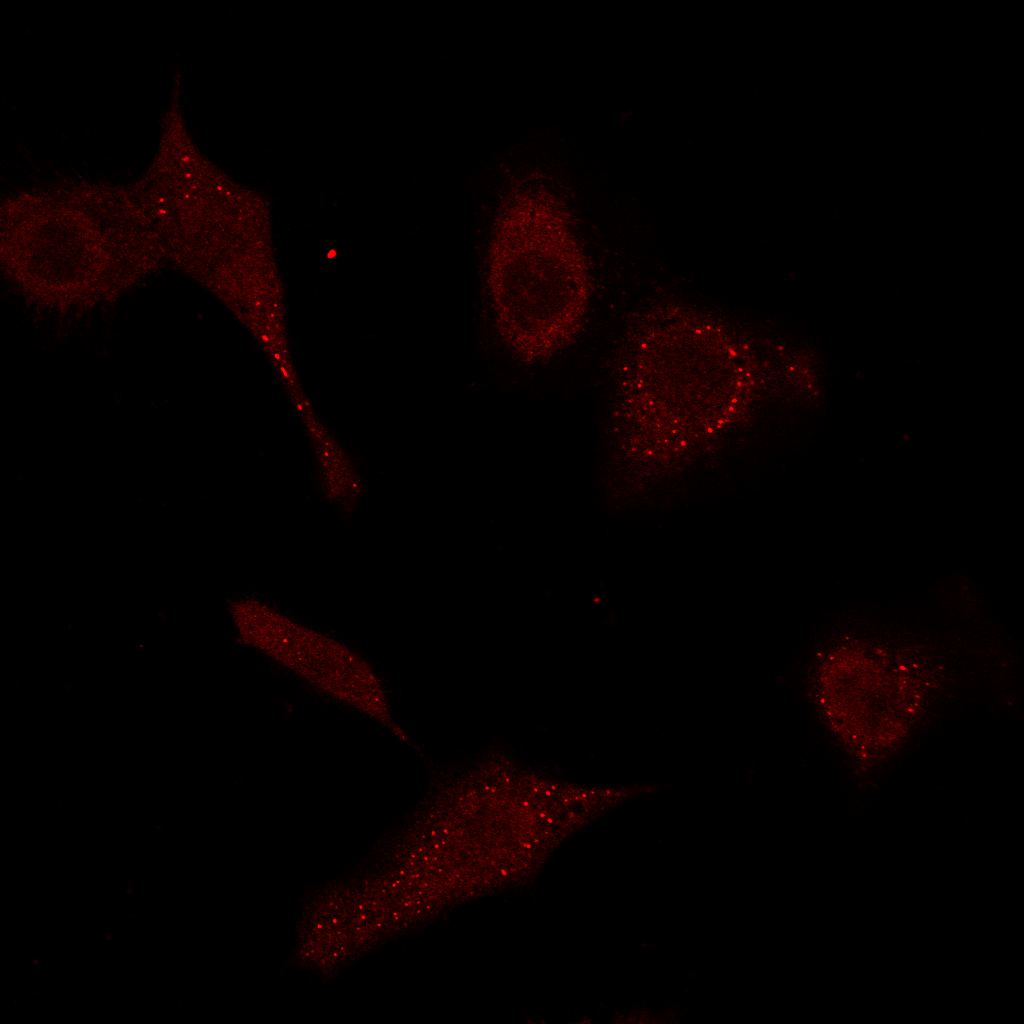

Supplement: Supplementary file 7 — Source data Fig. 6 [file 44318_2024_192_MOESM7_ESM.zip › Figure6/Figure6a/H2O2_positive/beta-Gal.tif]

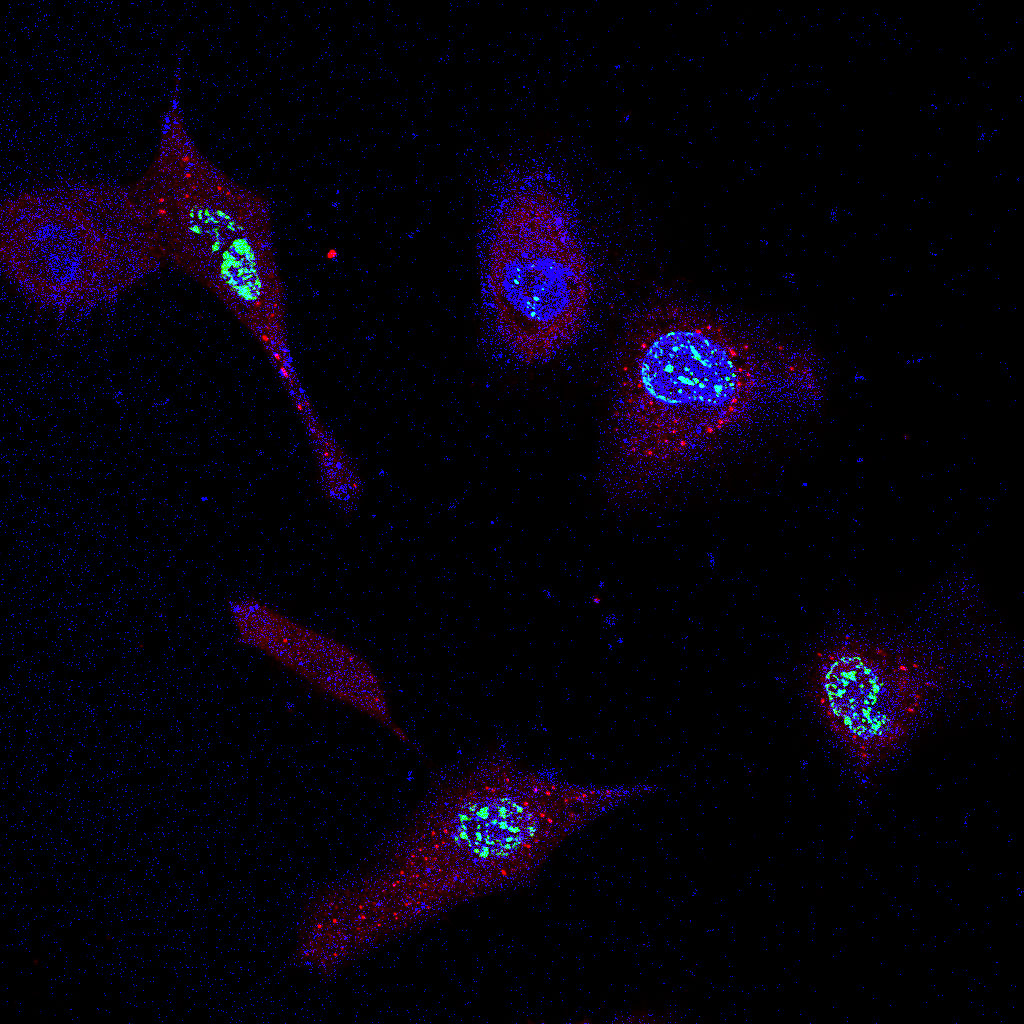

Supplement: Supplementary file 7 — Source data Fig. 6 [file 44318_2024_192_MOESM7_ESM.zip › Figure6/Figure6a/H2O2_positive/H3K9me3+Hoechst+beta-Gal.tif]

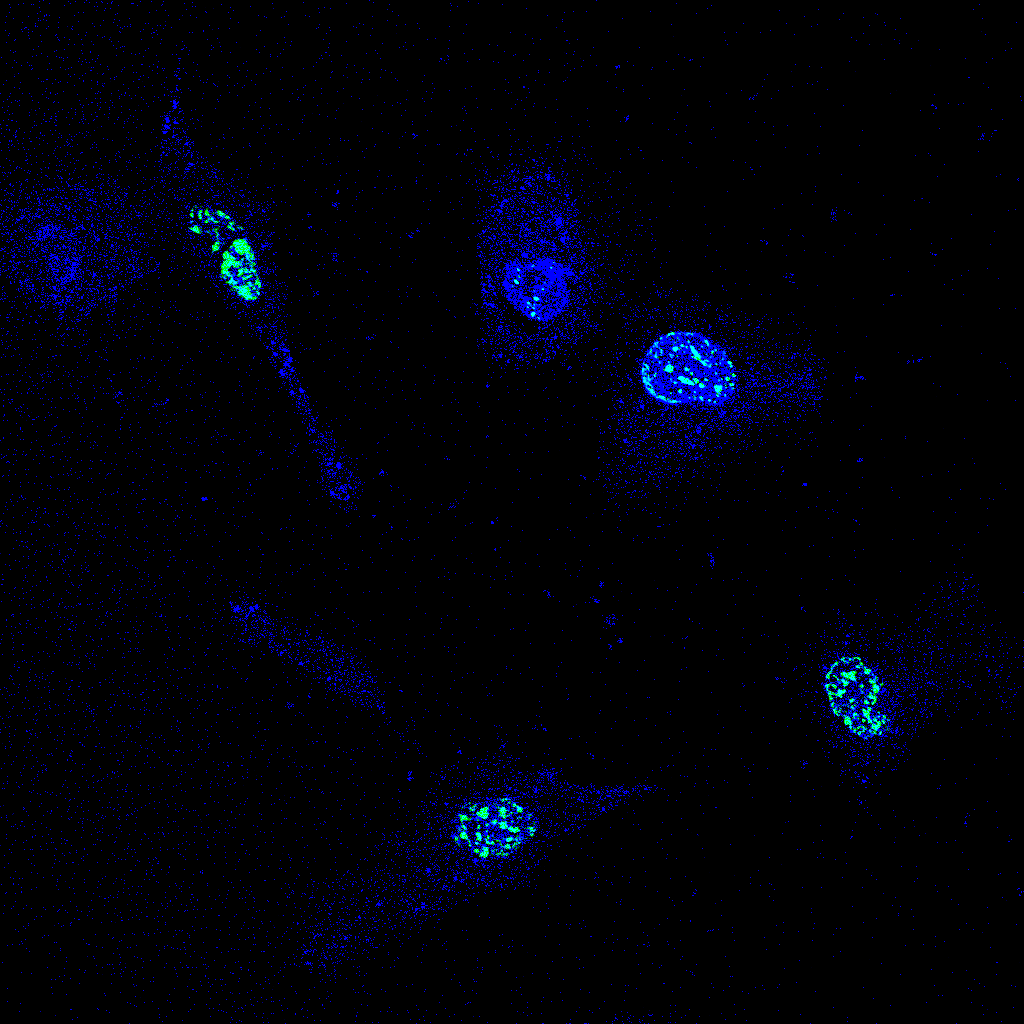

Supplement: Supplementary file 7 — Source data Fig. 6 [file 44318_2024_192_MOESM7_ESM.zip › Figure6/Figure6a/H2O2_positive/H3K9me3+Hoechst.tif]

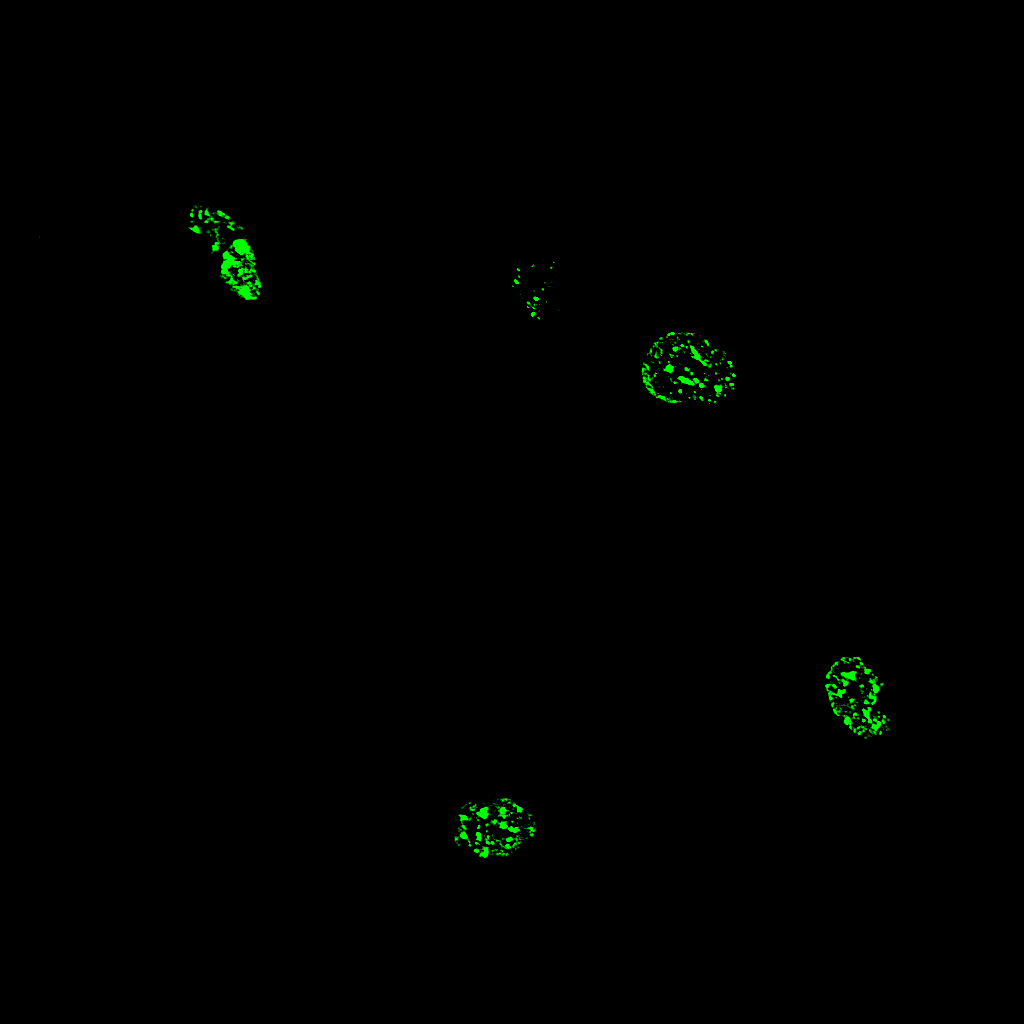

Supplement: Supplementary file 7 — Source data Fig. 6 [file 44318_2024_192_MOESM7_ESM.zip › Figure6/Figure6a/H2O2_positive/H3K9me3.tif]

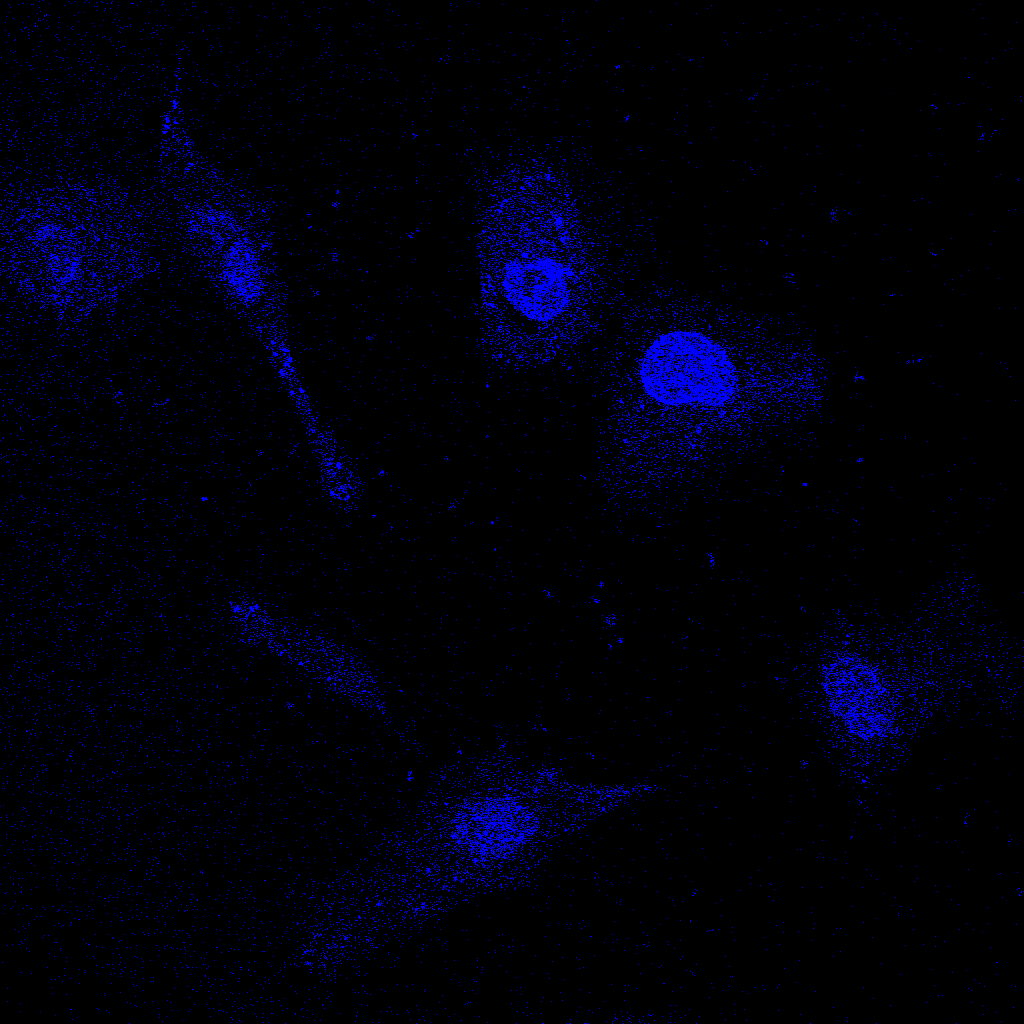

Supplement: Supplementary file 7 — Source data Fig. 6 [file 44318_2024_192_MOESM7_ESM.zip › Figure6/Figure6a/H2O2_positive/Hoechst.tif]

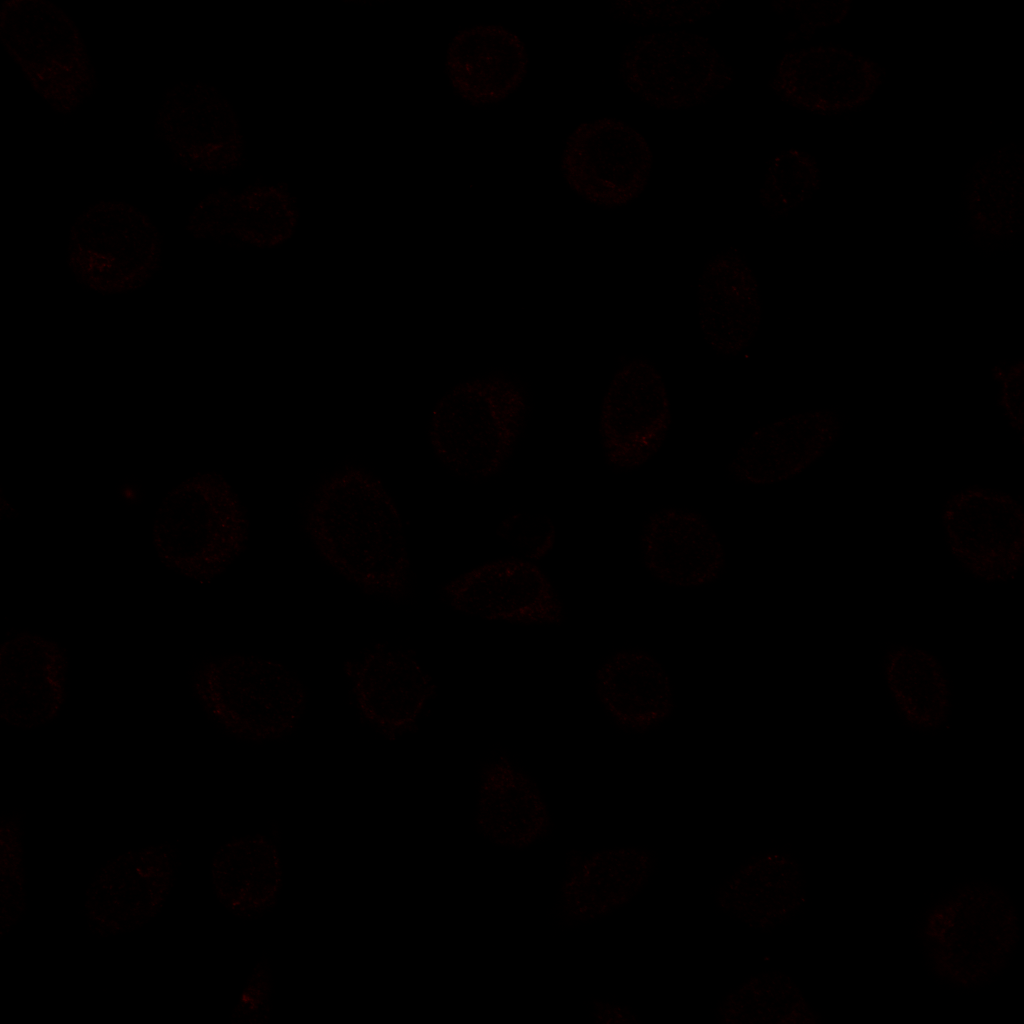

Supplement: Supplementary file 7 — Source data Fig. 6 [file 44318_2024_192_MOESM7_ESM.zip › Figure6/Figure6b/non-transfected/beta-Gal.tif]

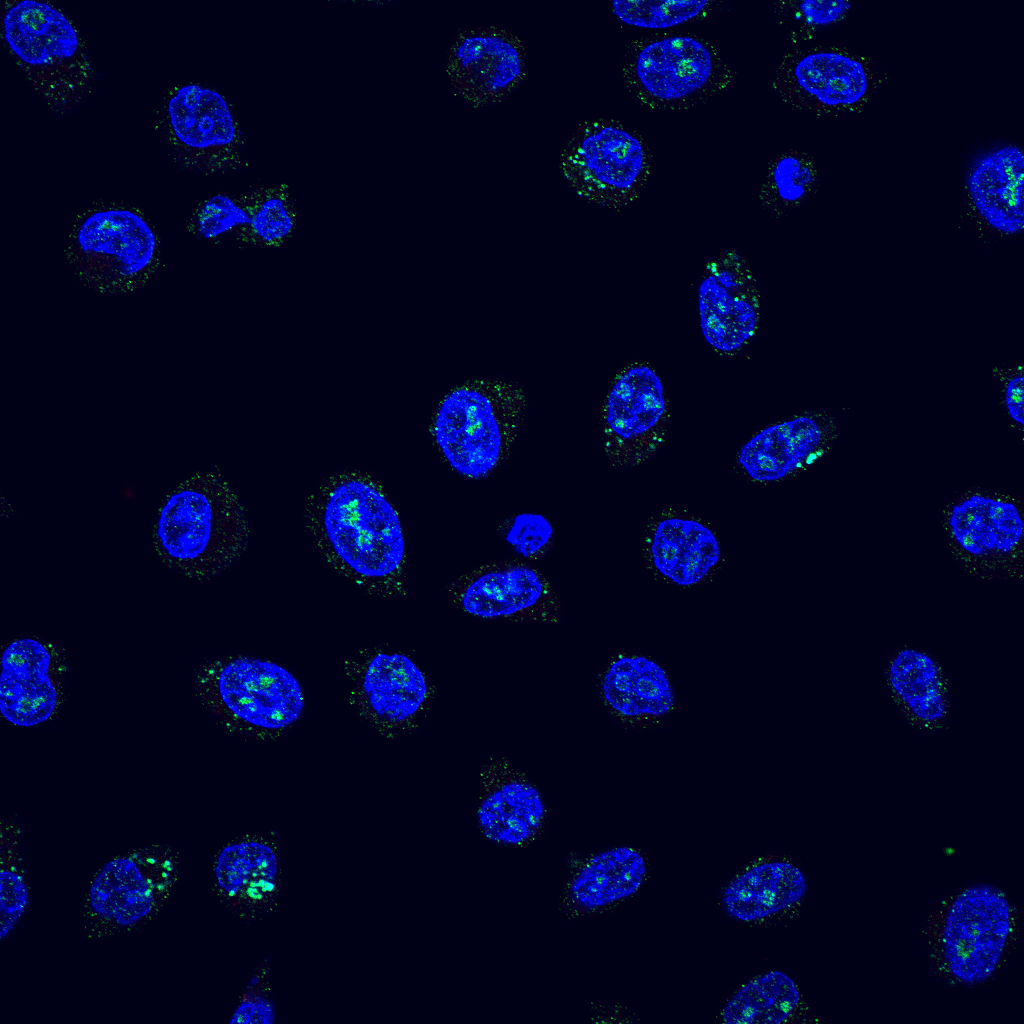

Supplement: Supplementary file 7 — Source data Fig. 6 [file 44318_2024_192_MOESM7_ESM.zip › Figure6/Figure6b/non-transfected/Merge.tif]

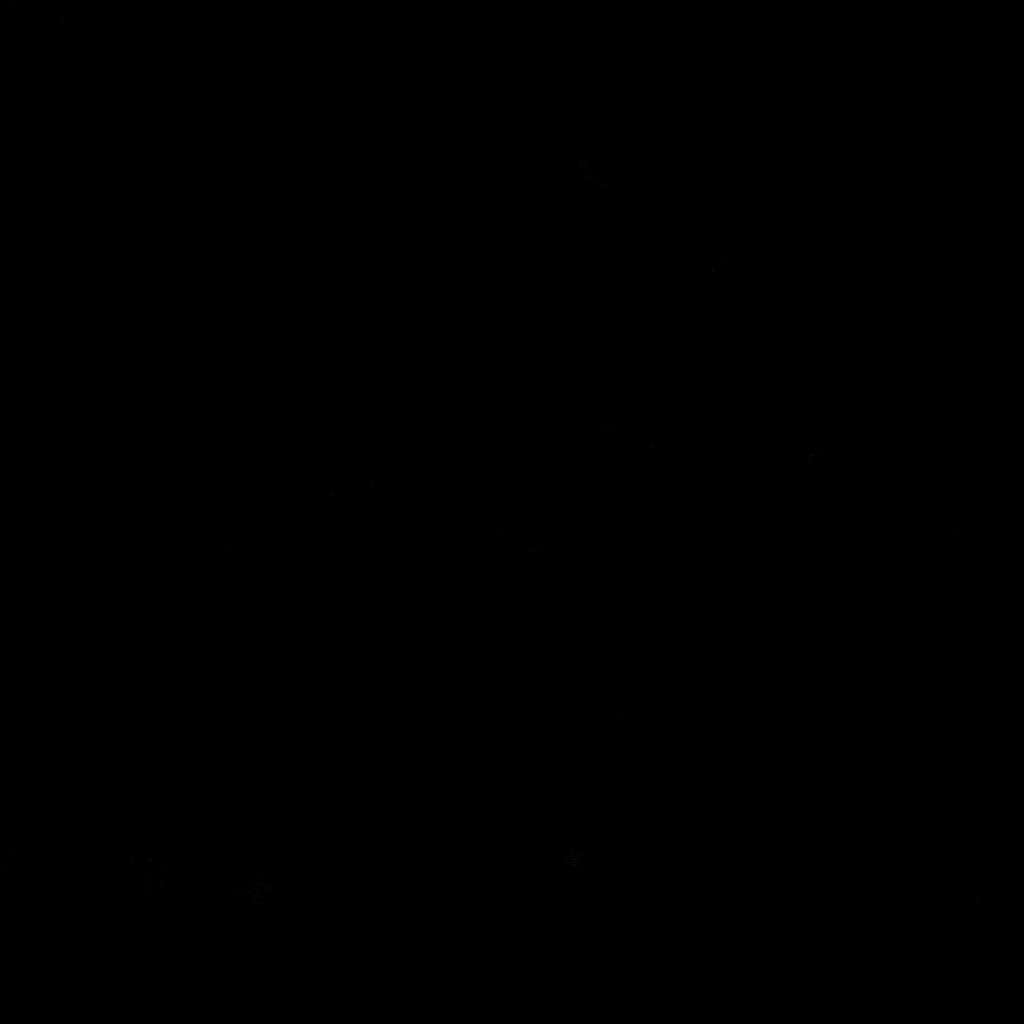

Supplement: Supplementary file 7 — Source data Fig. 6 [file 44318_2024_192_MOESM7_ESM.zip › Figure6/Figure6b/non-transfected/siRNA.tif]

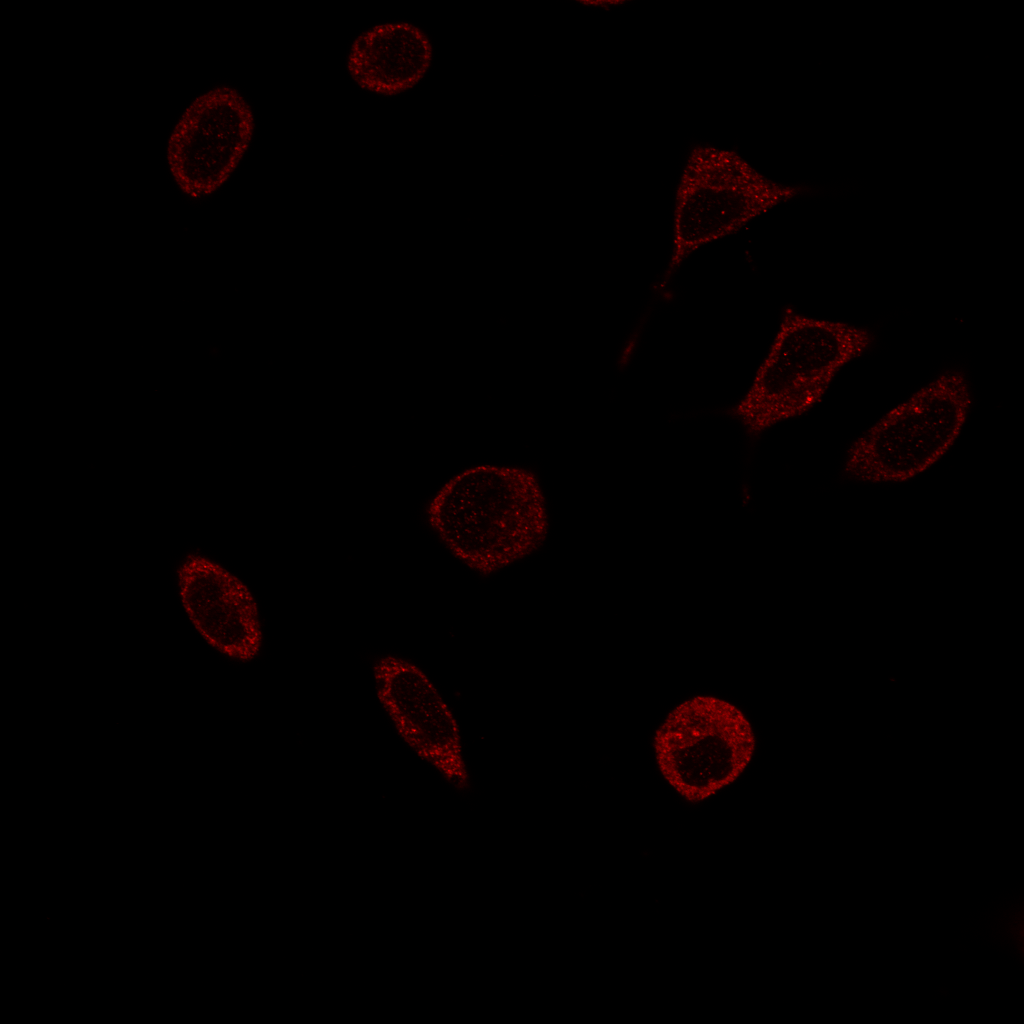

Supplement: Supplementary file 7 — Source data Fig. 6 [file 44318_2024_192_MOESM7_ESM.zip › Figure6/Figure6b/si-PQBP3#1/beta-Gal.tif]

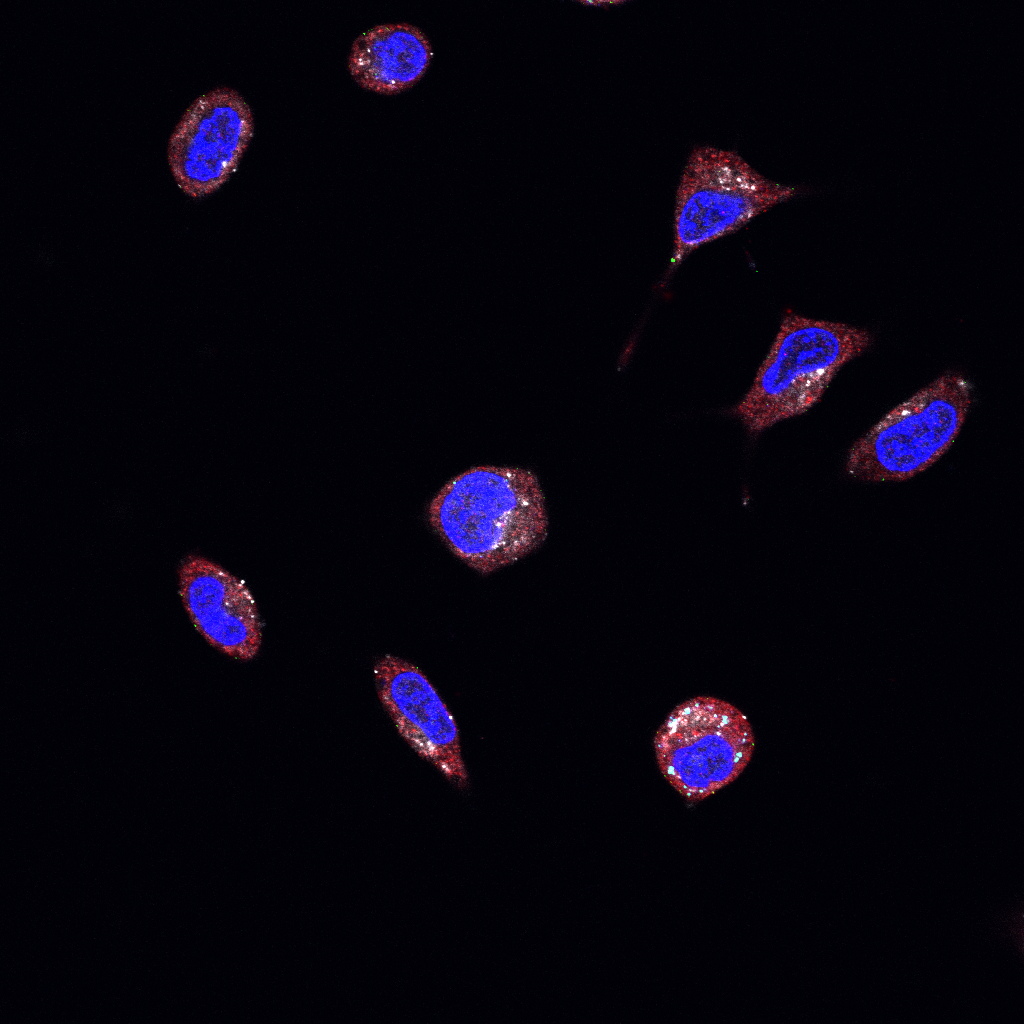

Supplement: Supplementary file 7 — Source data Fig. 6 [file 44318_2024_192_MOESM7_ESM.zip › Figure6/Figure6b/si-PQBP3#1/Merge.tif]

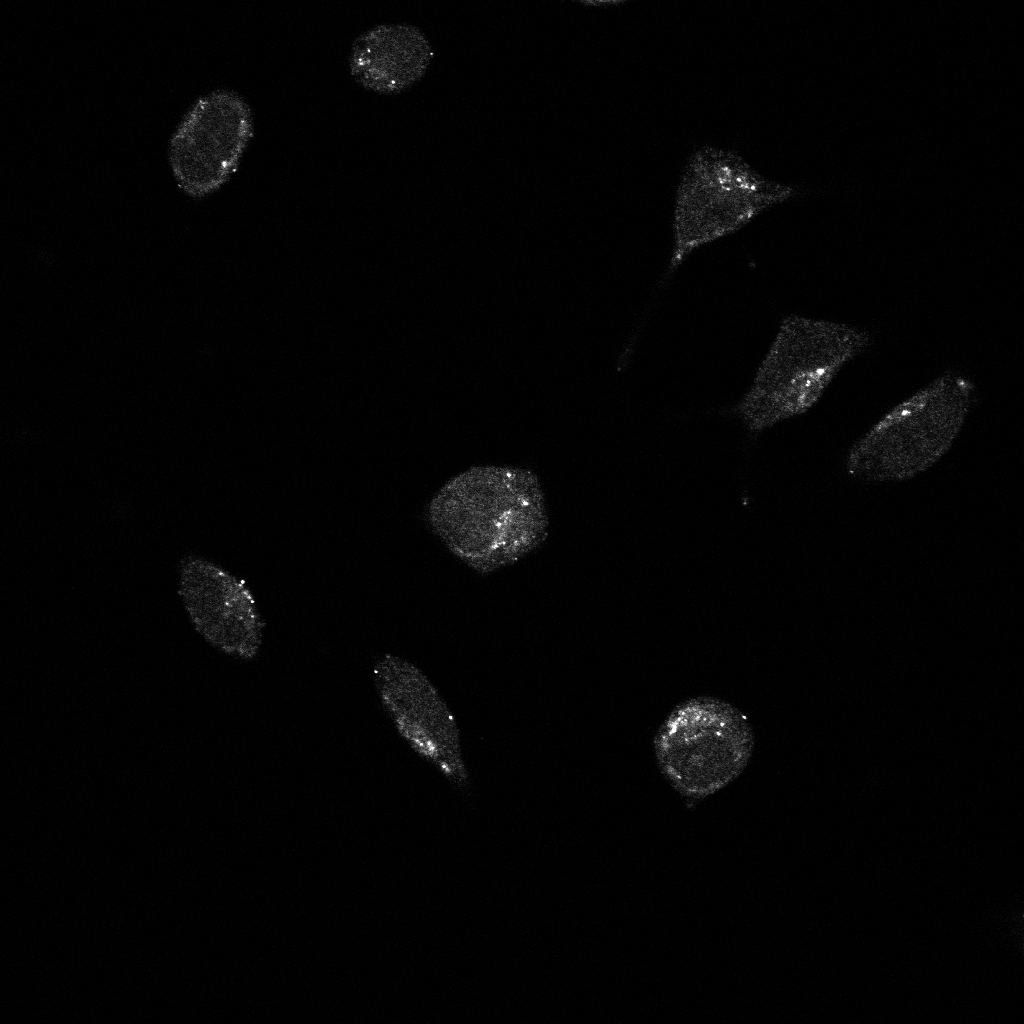

Supplement: Supplementary file 7 — Source data Fig. 6 [file 44318_2024_192_MOESM7_ESM.zip › Figure6/Figure6b/si-PQBP3#1/siRNA.tif]

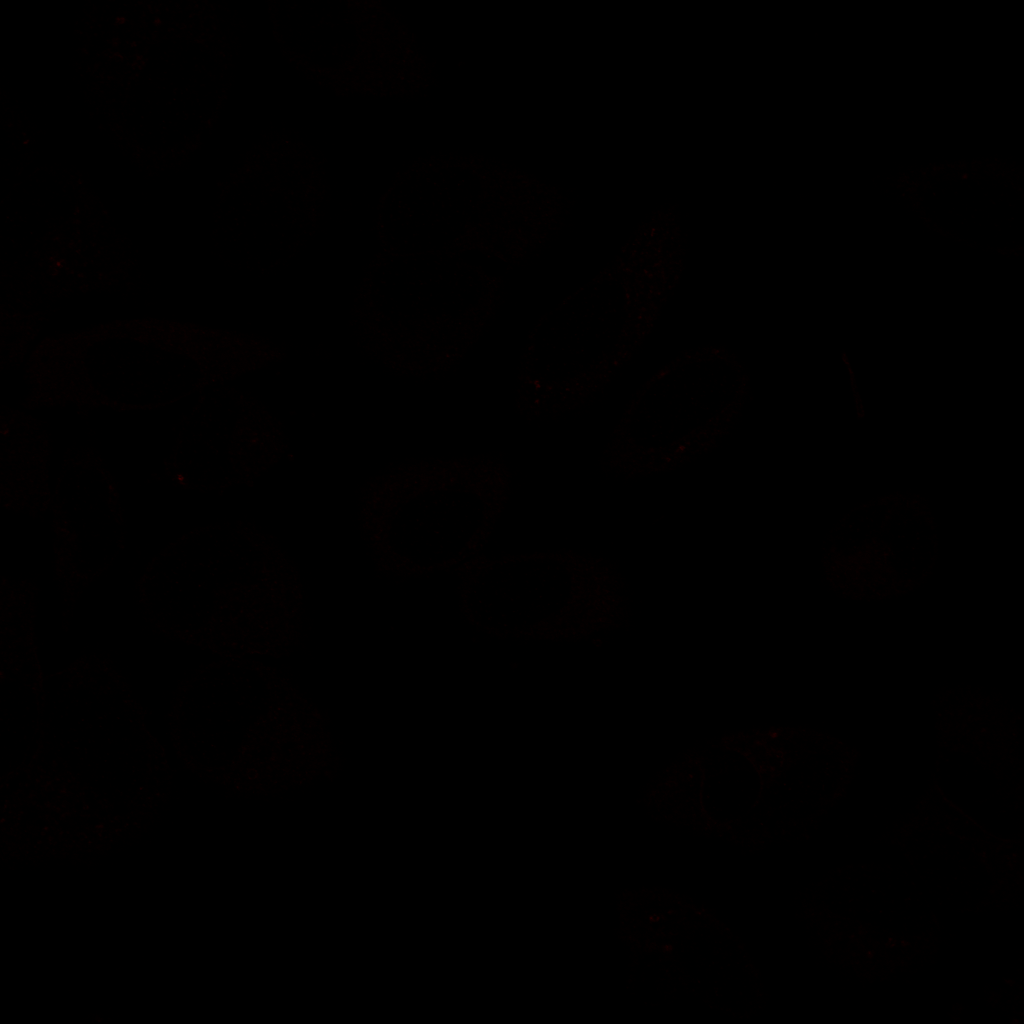

Supplement: Supplementary file 7 — Source data Fig. 6 [file 44318_2024_192_MOESM7_ESM.zip › Figure6/Figure6b/si-Scramble/beta-Gal.tif]

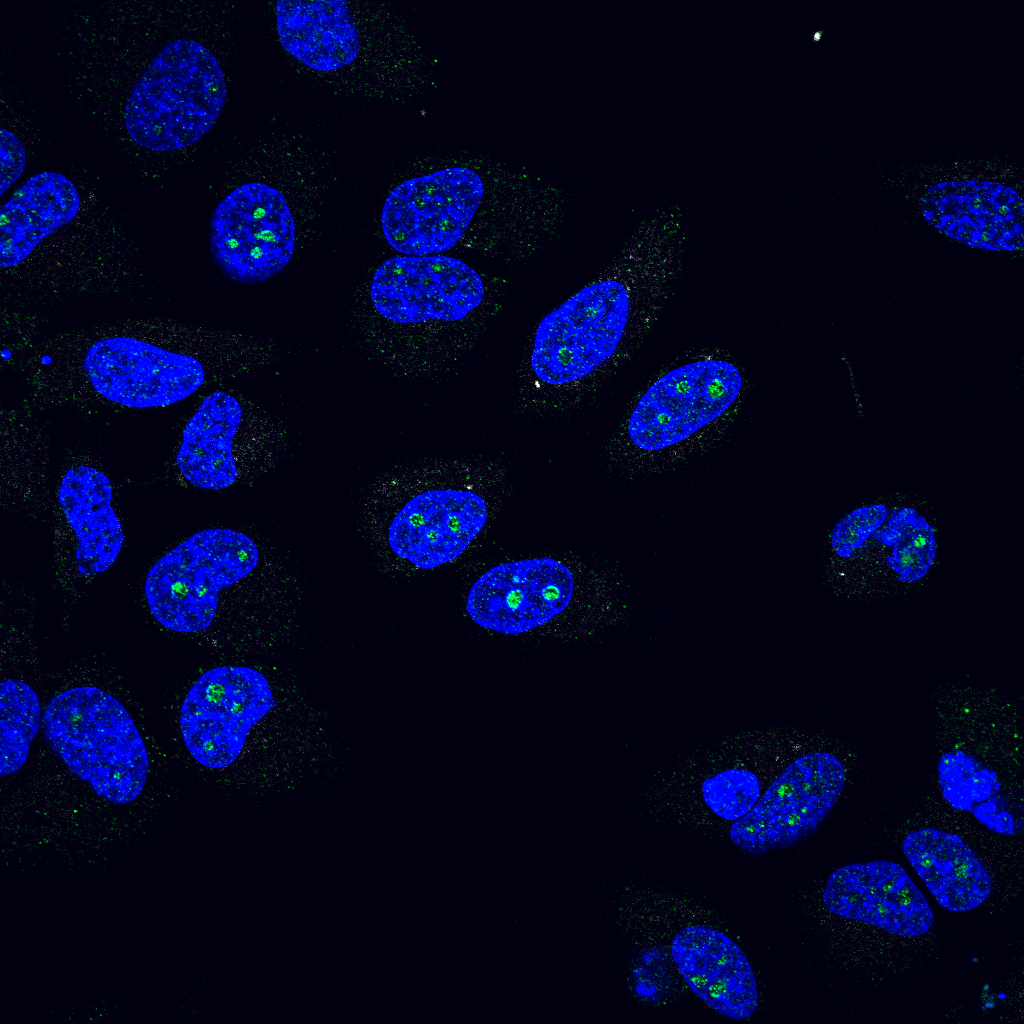

Supplement: Supplementary file 7 — Source data Fig. 6 [file 44318_2024_192_MOESM7_ESM.zip › Figure6/Figure6b/si-Scramble/Merge.tif]

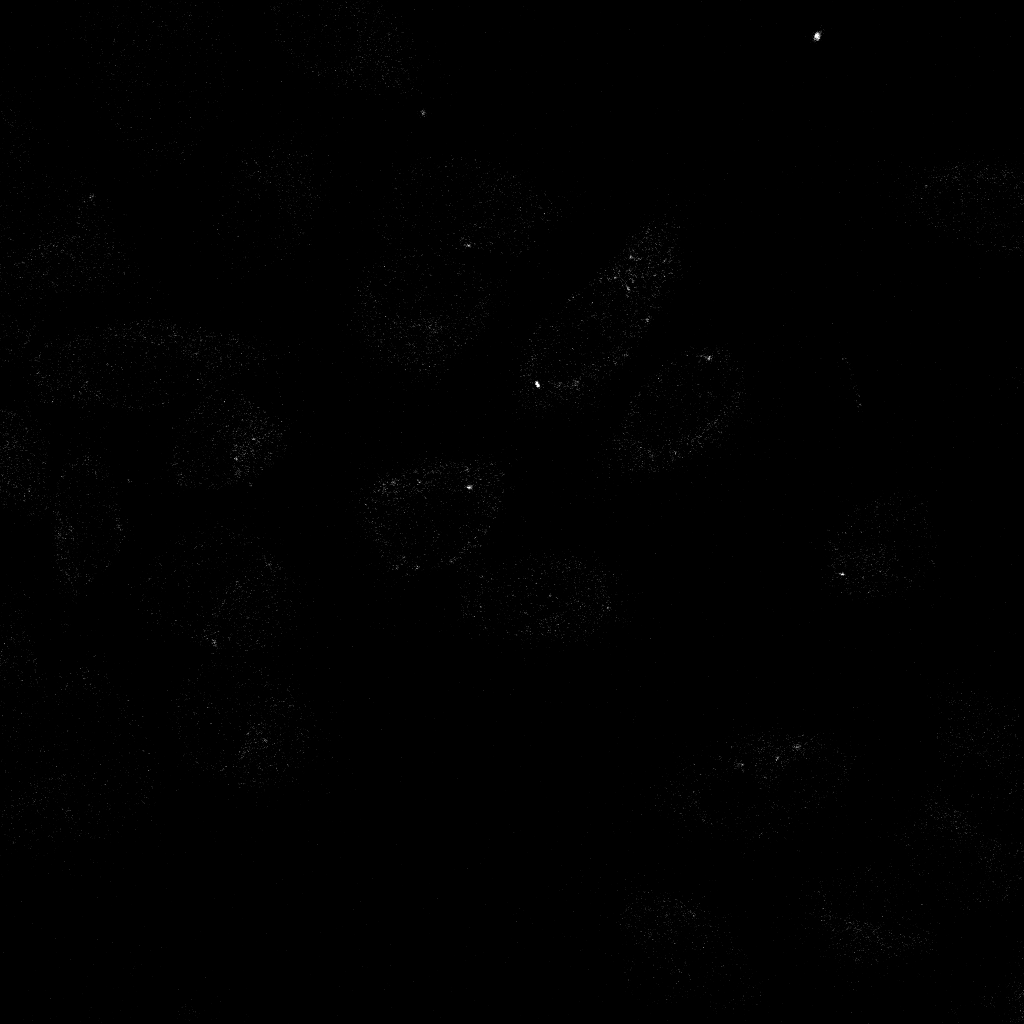

Supplement: Supplementary file 7 — Source data Fig. 6 [file 44318_2024_192_MOESM7_ESM.zip › Figure6/Figure6b/si-Scramble/siRNA.tif]

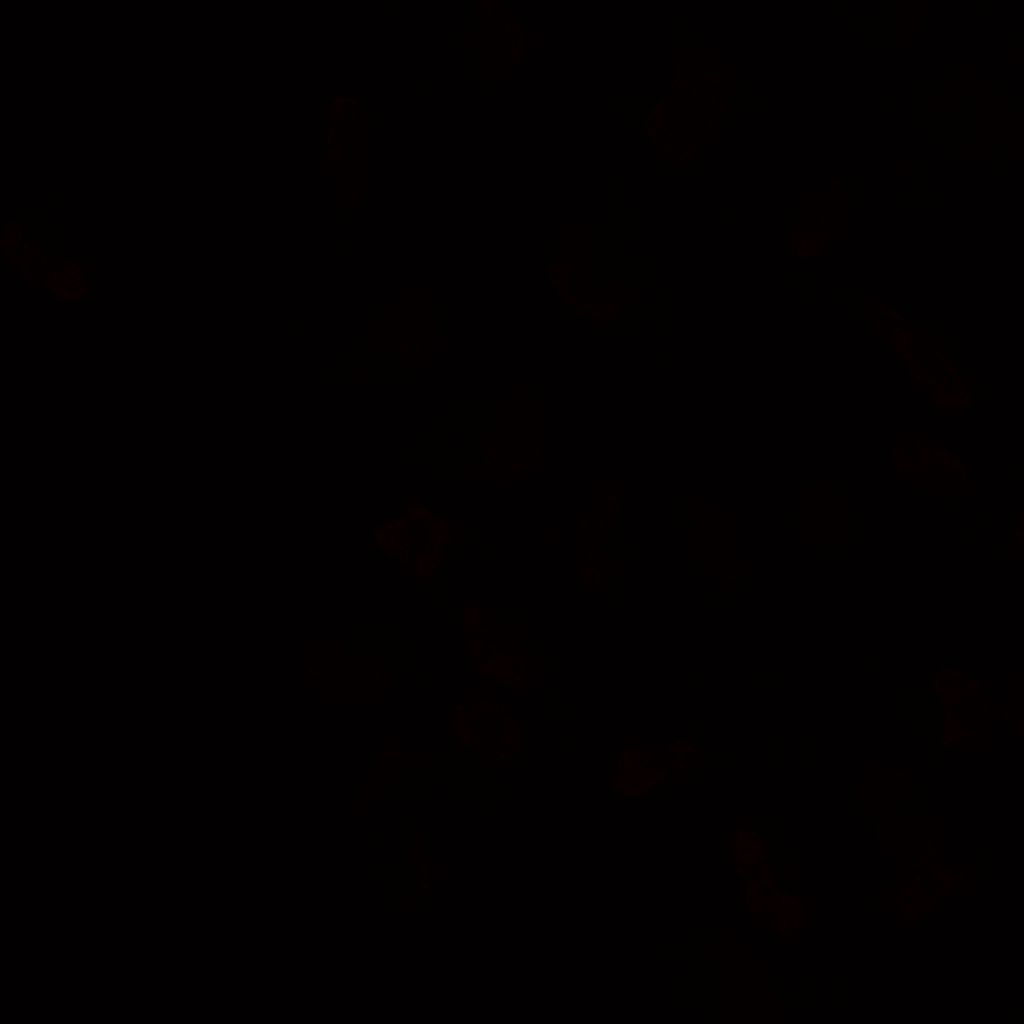

Supplement: Supplementary file 7 — Source data Fig. 6 [file 44318_2024_192_MOESM7_ESM.zip › Figure6/Figure6c/H2O2_negative/beta-Gal.tif]

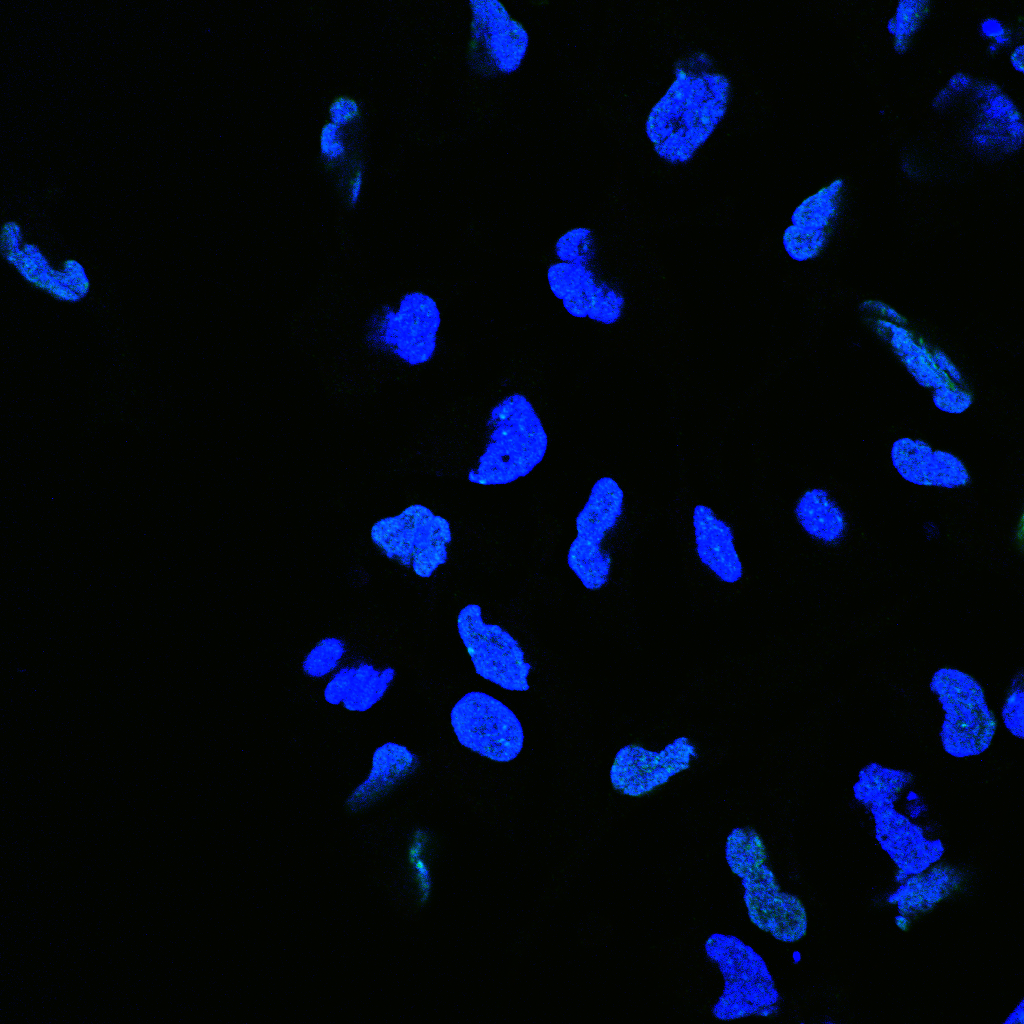

Supplement: Supplementary file 7 — Source data Fig. 6 [file 44318_2024_192_MOESM7_ESM.zip › Figure6/Figure6c/H2O2_negative/H3K9me3+Hoechst+beta-Gal.tif]

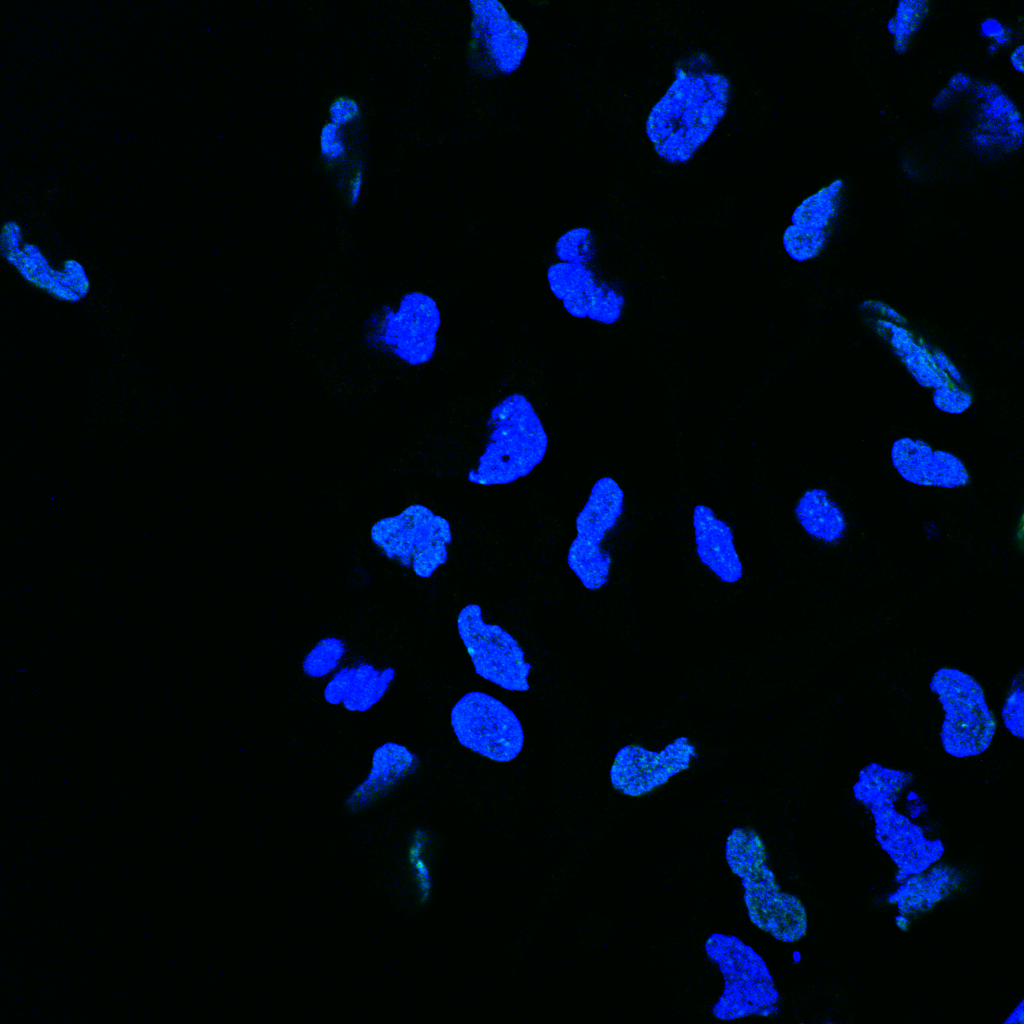

Supplement: Supplementary file 7 — Source data Fig. 6 [file 44318_2024_192_MOESM7_ESM.zip › Figure6/Figure6c/H2O2_negative/H3K9me3+Hoechst.tif]

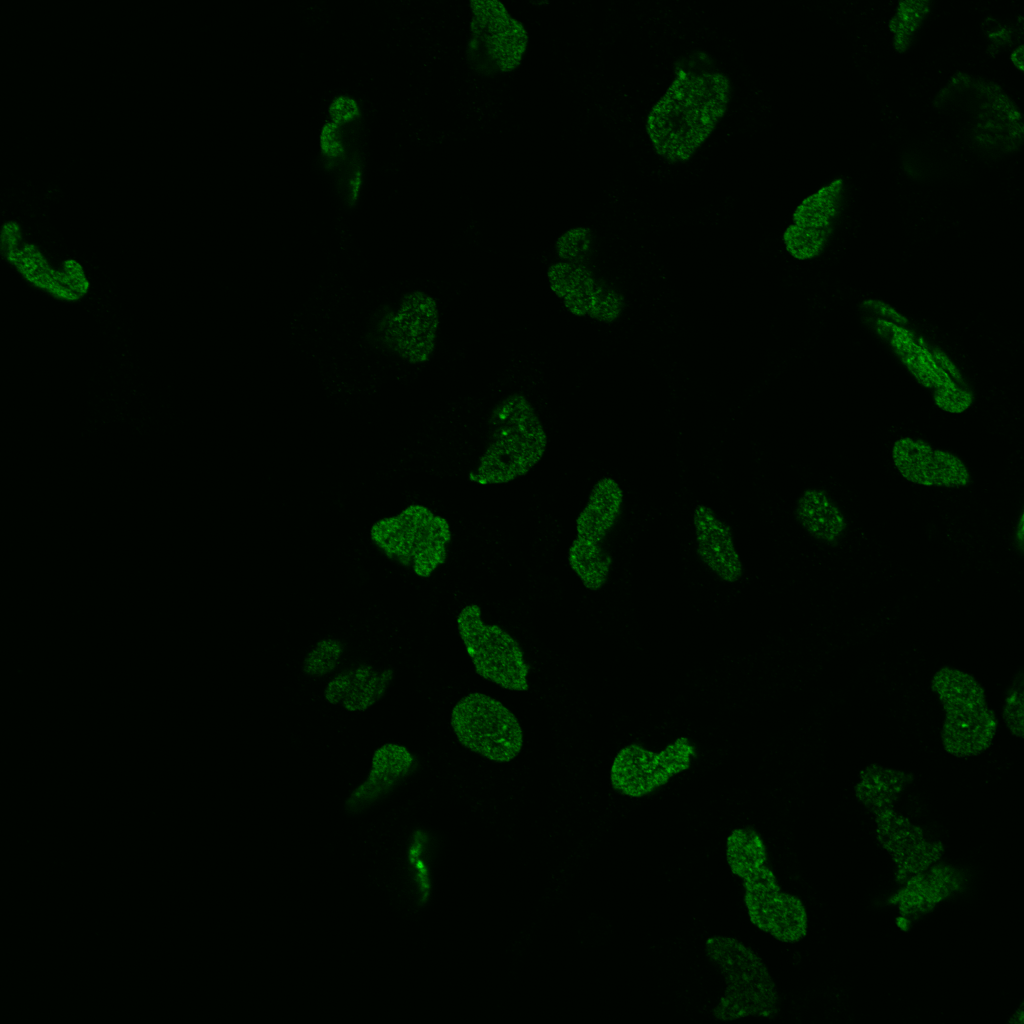

Supplement: Supplementary file 7 — Source data Fig. 6 [file 44318_2024_192_MOESM7_ESM.zip › Figure6/Figure6c/H2O2_negative/H3K9me3.tif]

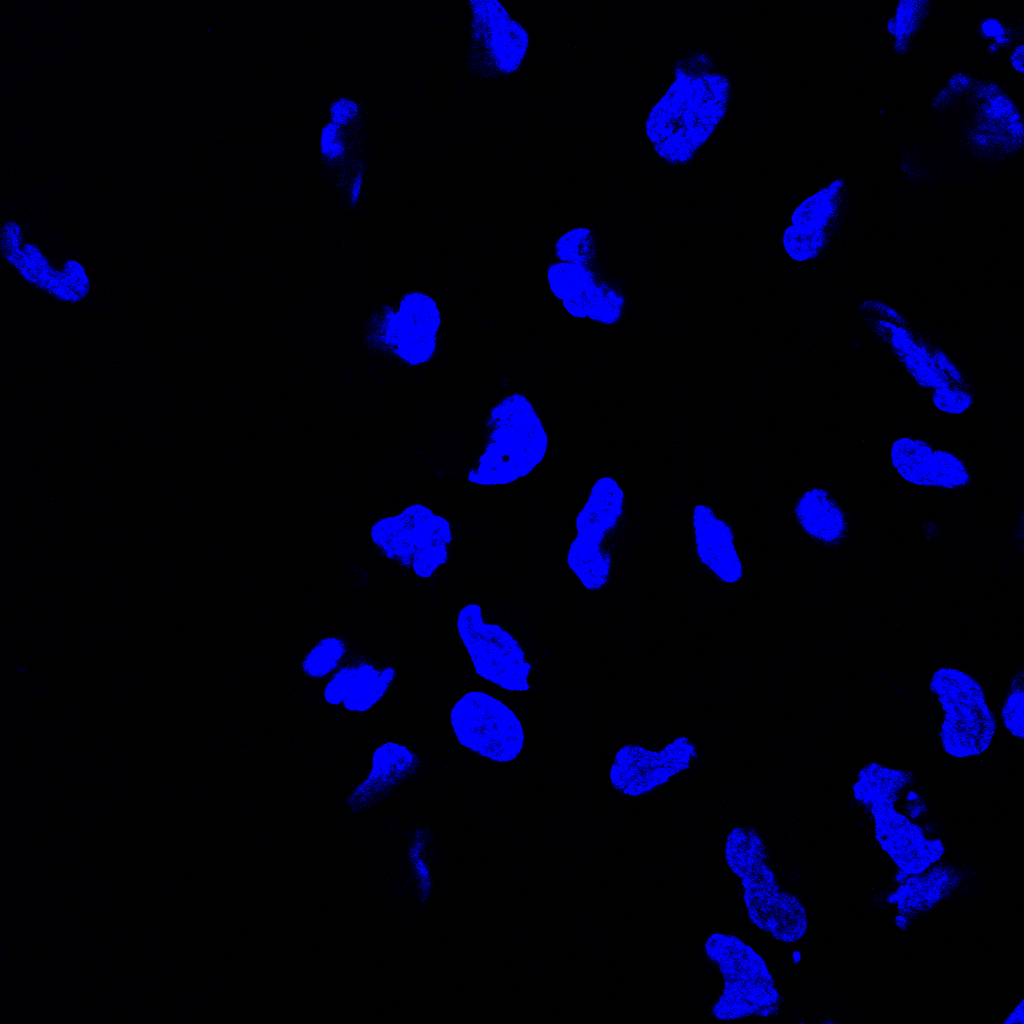

Supplement: Supplementary file 7 — Source data Fig. 6 [file 44318_2024_192_MOESM7_ESM.zip › Figure6/Figure6c/H2O2_negative/Hoechst.tif]

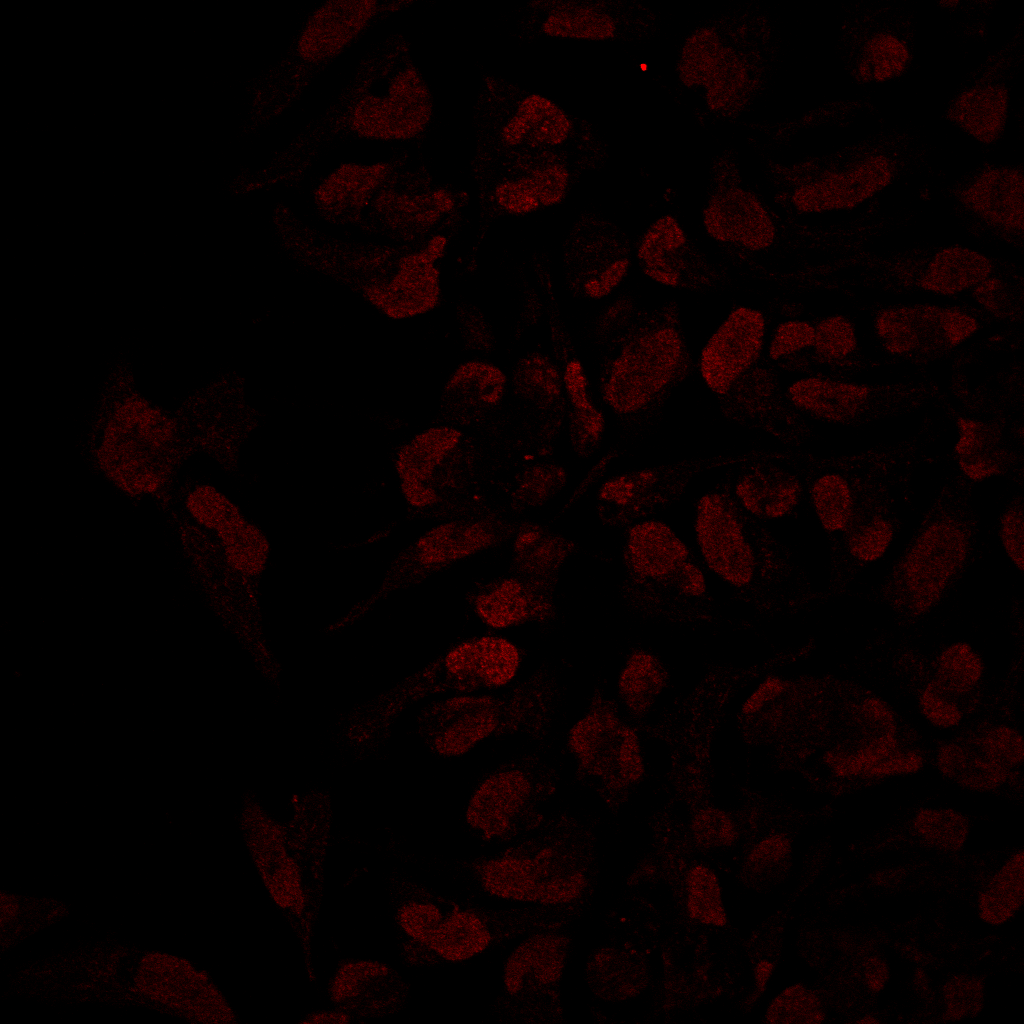

Supplement: Supplementary file 7 — Source data Fig. 6 [file 44318_2024_192_MOESM7_ESM.zip › Figure6/Figure6c/H2O2_positive/beta-Gal.tif]

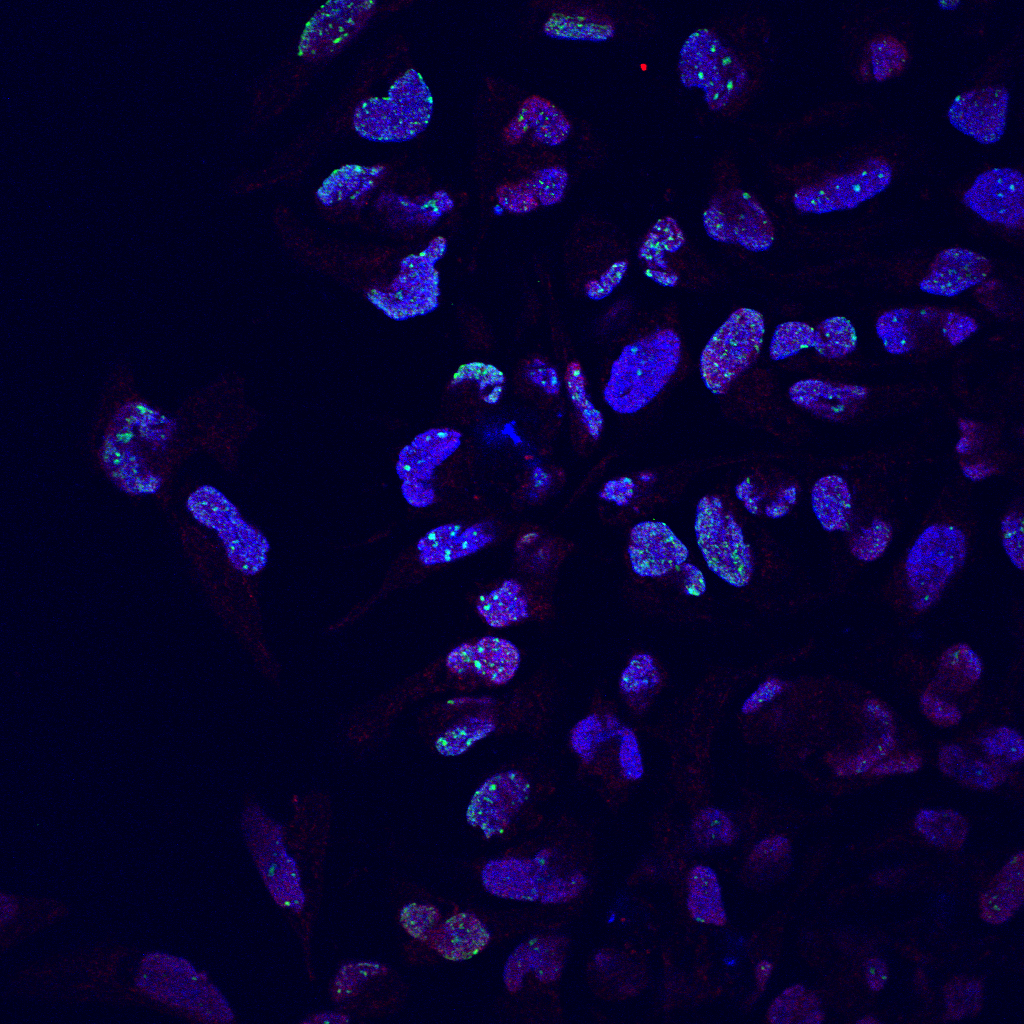

Supplement: Supplementary file 7 — Source data Fig. 6 [file 44318_2024_192_MOESM7_ESM.zip › Figure6/Figure6c/H2O2_positive/H3K9me3+Hoechst+beta-Gal.tif]

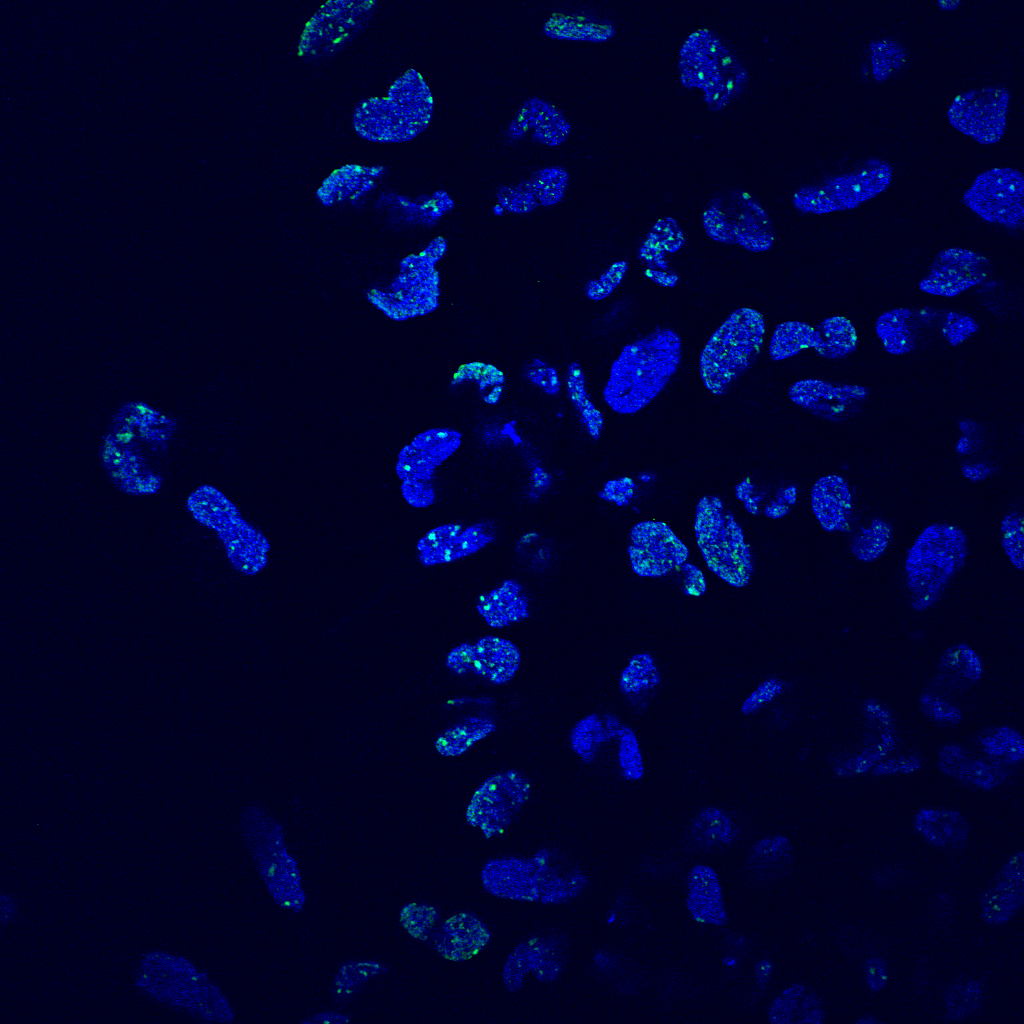

Supplement: Supplementary file 7 — Source data Fig. 6 [file 44318_2024_192_MOESM7_ESM.zip › Figure6/Figure6c/H2O2_positive/H3K9me3+Hoechst.tif]

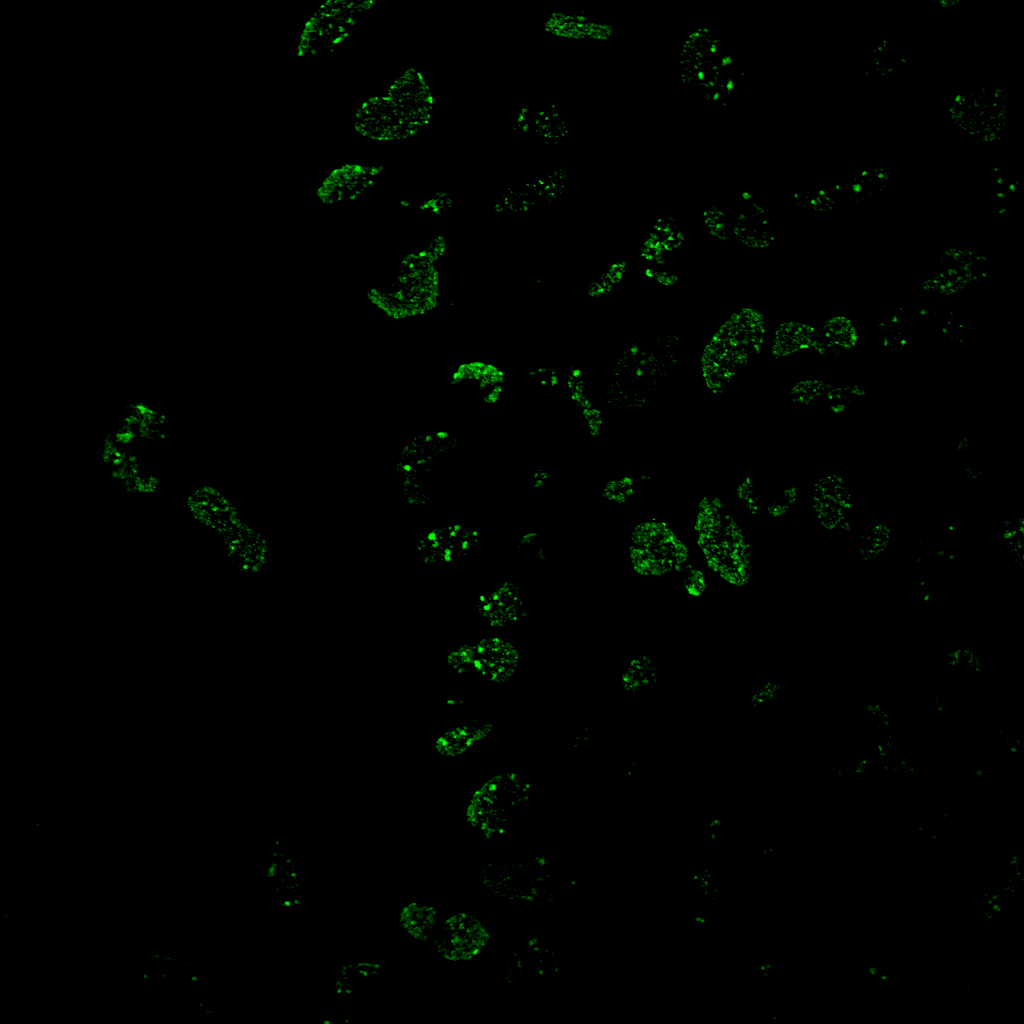

Supplement: Supplementary file 7 — Source data Fig. 6 [file 44318_2024_192_MOESM7_ESM.zip › Figure6/Figure6c/H2O2_positive/H3K9me3.tif]

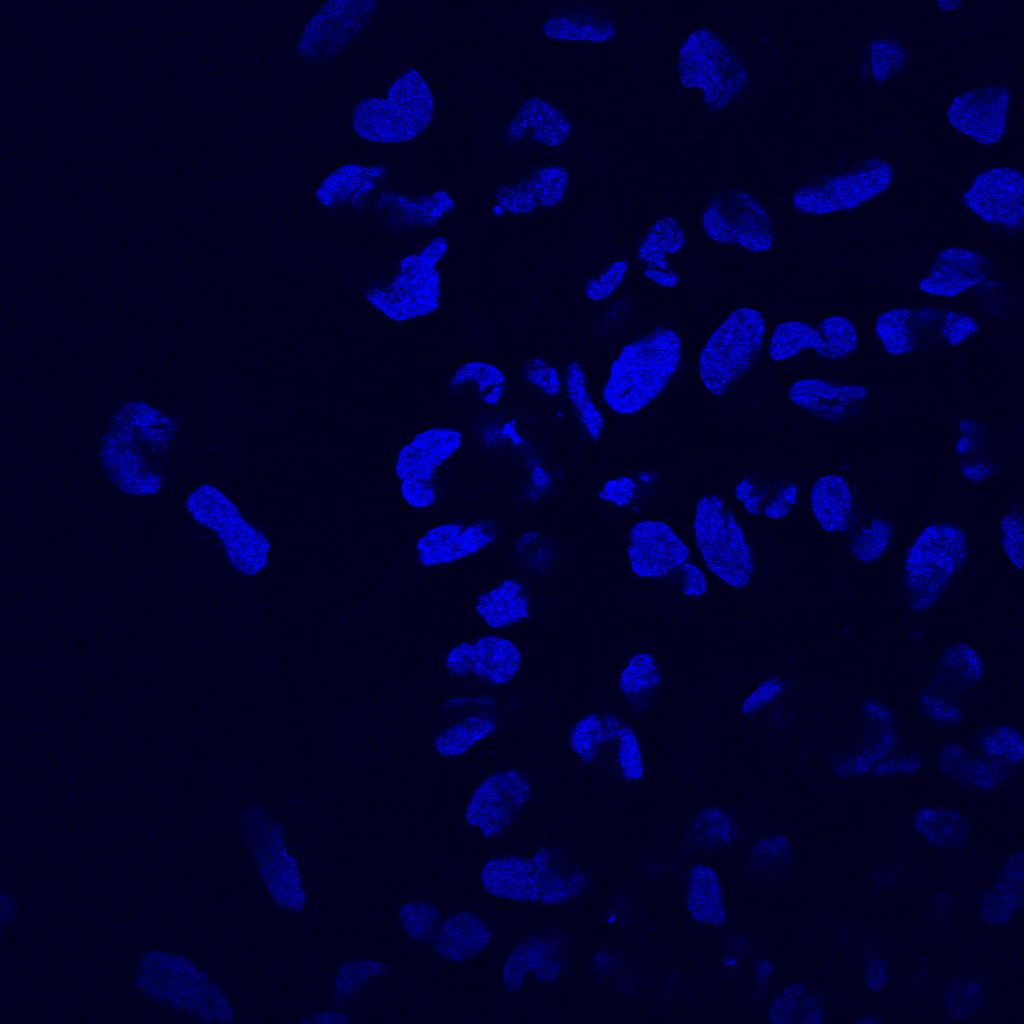

Supplement: Supplementary file 7 — Source data Fig. 6 [file 44318_2024_192_MOESM7_ESM.zip › Figure6/Figure6c/H2O2_positive/Hoechst.tif]

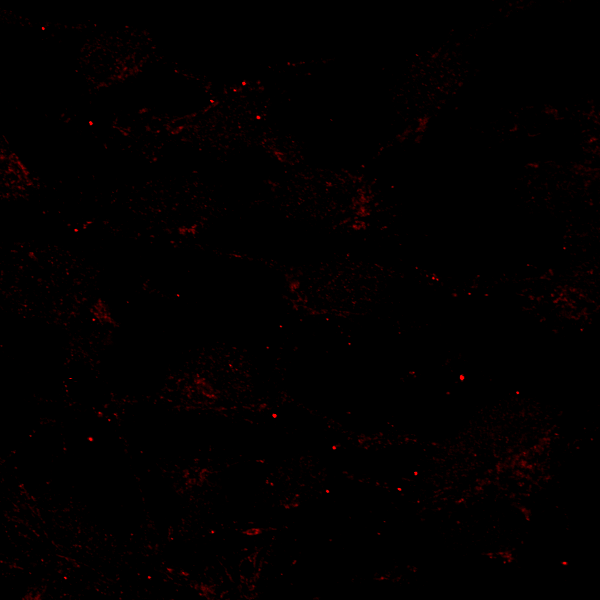

Supplement: Supplementary file 7 — Source data Fig. 6 [file 44318_2024_192_MOESM7_ESM.zip › Figure6/Figure6d/non-transfected/beta-Gal.tiff]

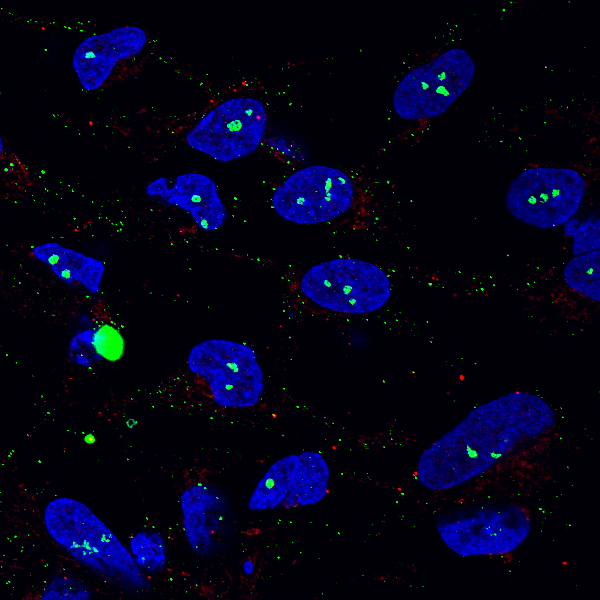

Supplement: Supplementary file 7 — Source data Fig. 6 [file 44318_2024_192_MOESM7_ESM.zip › Figure6/Figure6d/non-transfected/Merge.tiff]

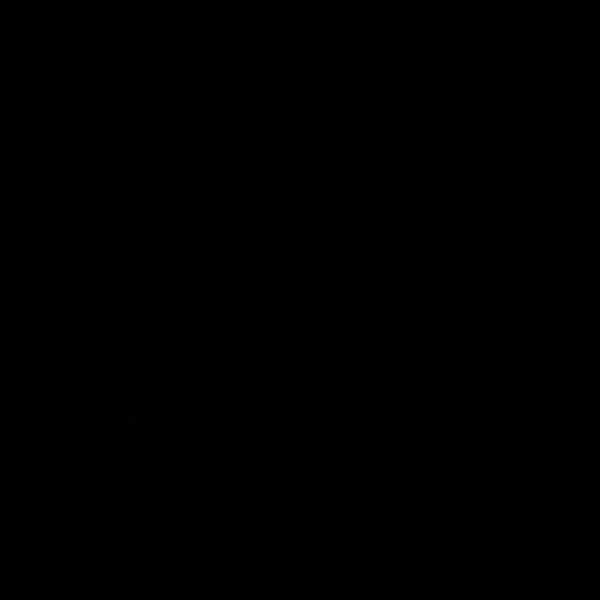

Supplement: Supplementary file 7 — Source data Fig. 6 [file 44318_2024_192_MOESM7_ESM.zip › Figure6/Figure6d/non-transfected/siRNA.tiff]

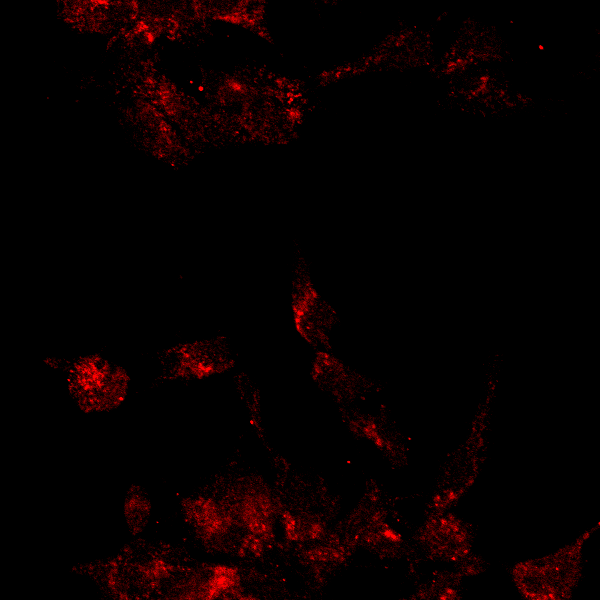

Supplement: Supplementary file 7 — Source data Fig. 6 [file 44318_2024_192_MOESM7_ESM.zip › Figure6/Figure6d/si-PQBP3#1/beta-Gal.tiff]

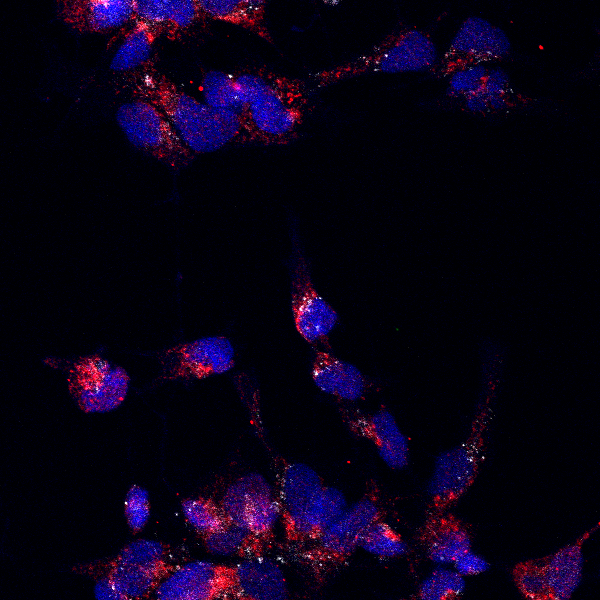

Supplement: Supplementary file 7 — Source data Fig. 6 [file 44318_2024_192_MOESM7_ESM.zip › Figure6/Figure6d/si-PQBP3#1/Merge.tiff]

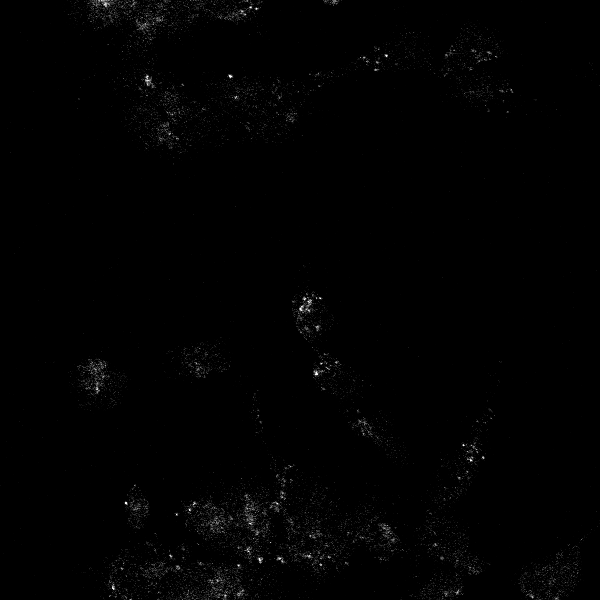

Supplement: Supplementary file 7 — Source data Fig. 6 [file 44318_2024_192_MOESM7_ESM.zip › Figure6/Figure6d/si-PQBP3#1/siRNA.tiff]

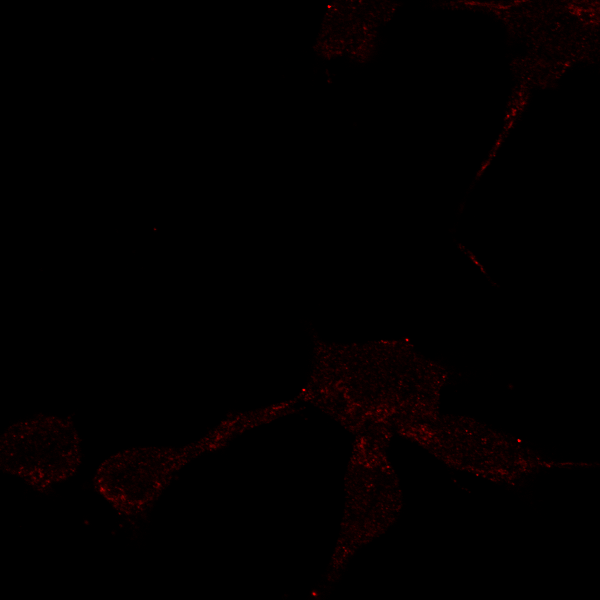

Supplement: Supplementary file 7 — Source data Fig. 6 [file 44318_2024_192_MOESM7_ESM.zip › Figure6/Figure6d/si-Scramble/beta-Gal.tiff]

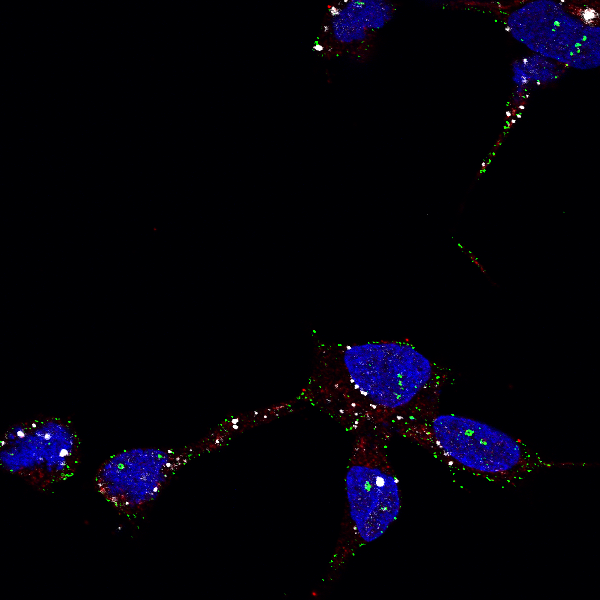

Supplement: Supplementary file 7 — Source data Fig. 6 [file 44318_2024_192_MOESM7_ESM.zip › Figure6/Figure6d/si-Scramble/Merge.tiff]

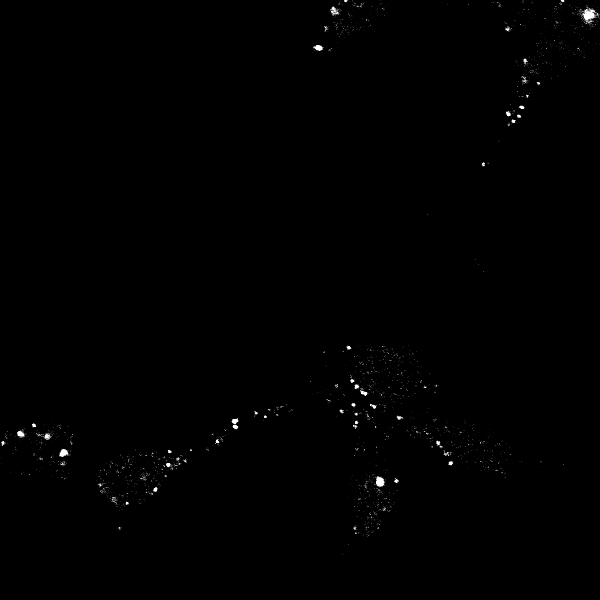

Supplement: Supplementary file 7 — Source data Fig. 6 [file 44318_2024_192_MOESM7_ESM.zip › Figure6/Figure6d/si-Scramble/siRNA.tiff]

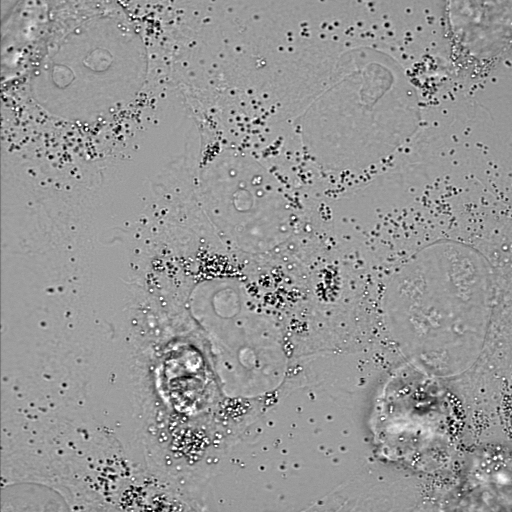

Supplement: Supplementary file 8 — Source data Fig. 7 [file 44318_2024_192_MOESM8_ESM.zip › Figure7/Figure7a/EGFP-PQBP3/Bright field.tif]

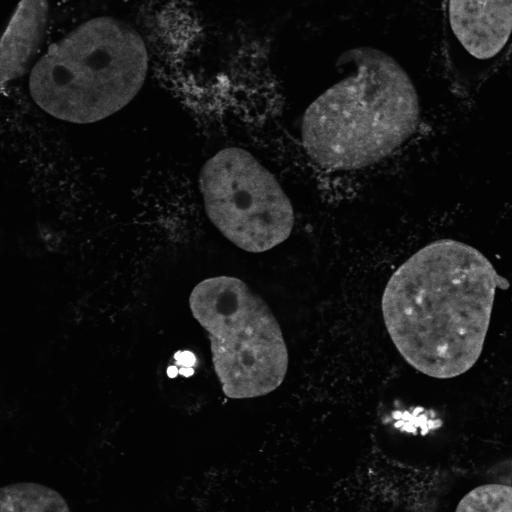

Supplement: Supplementary file 8 — Source data Fig. 7 [file 44318_2024_192_MOESM8_ESM.zip › Figure7/Figure7a/EGFP-PQBP3/DAPI.tif]

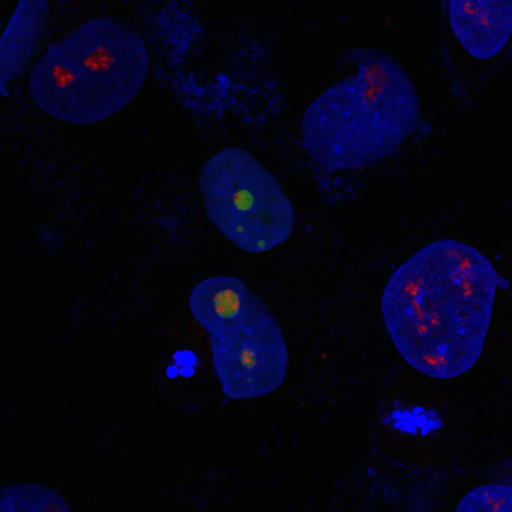

Supplement: Supplementary file 8 — Source data Fig. 7 [file 44318_2024_192_MOESM8_ESM.zip › Figure7/Figure7a/EGFP-PQBP3/EGFP-PQBP3+Fibrillarin+DAPI.tif]

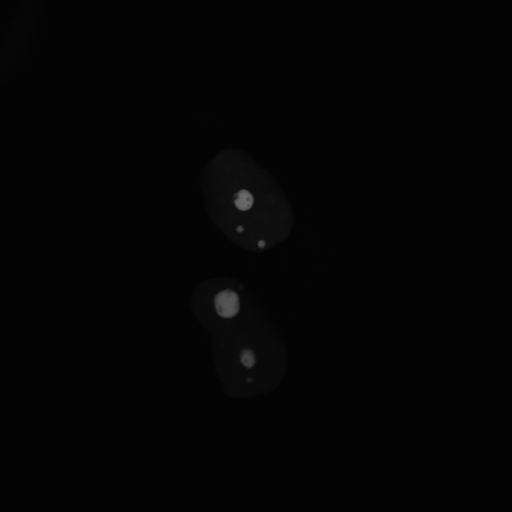

Supplement: Supplementary file 8 — Source data Fig. 7 [file 44318_2024_192_MOESM8_ESM.zip › Figure7/Figure7a/EGFP-PQBP3/EGFP-PQBP3.tif]

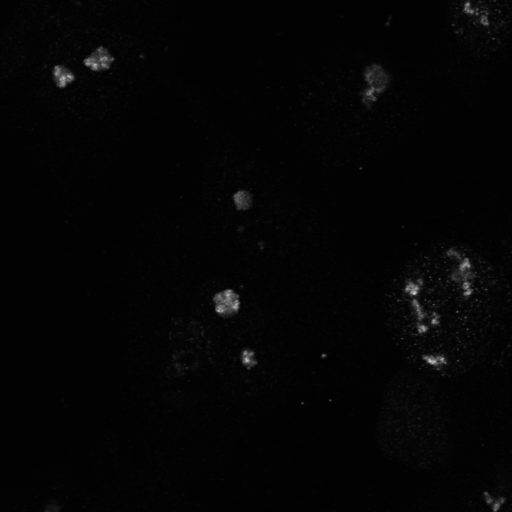

Supplement: Supplementary file 8 — Source data Fig. 7 [file 44318_2024_192_MOESM8_ESM.zip › Figure7/Figure7a/EGFP-PQBP3/Fibrillarin.tif]

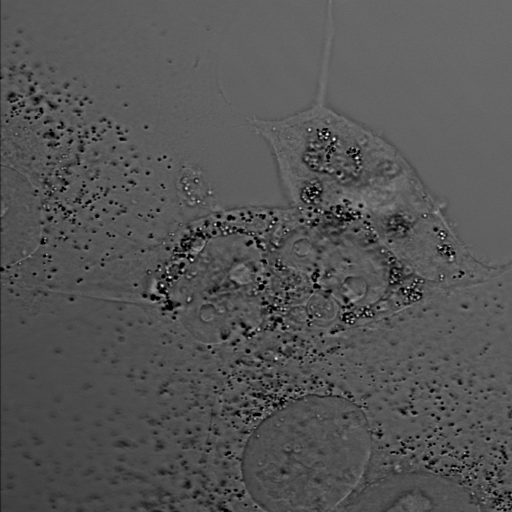

Supplement: Supplementary file 8 — Source data Fig. 7 [file 44318_2024_192_MOESM8_ESM.zip › Figure7/Figure7a/PQBP3-EGFP/Bright field.tif]

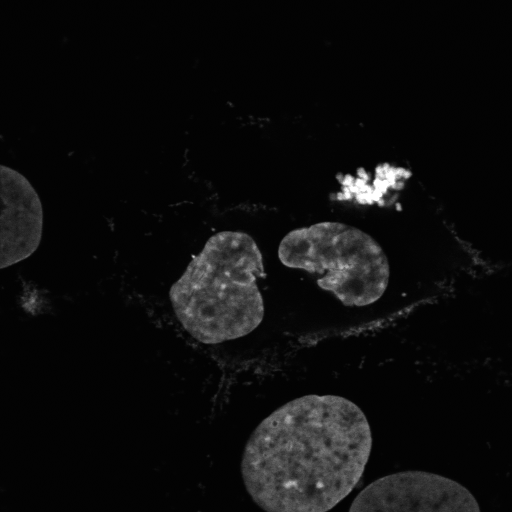

Supplement: Supplementary file 8 — Source data Fig. 7 [file 44318_2024_192_MOESM8_ESM.zip › Figure7/Figure7a/PQBP3-EGFP/DAPI.tif]

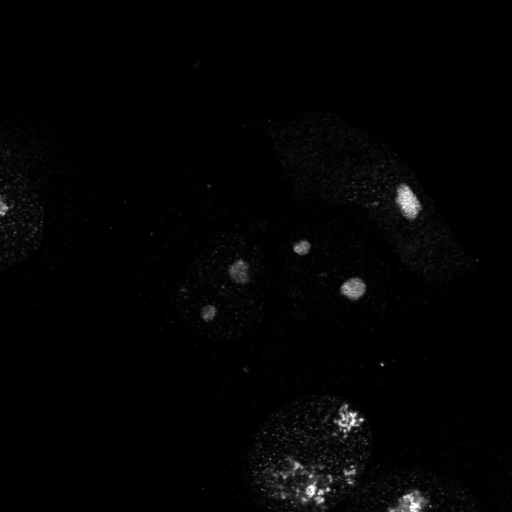

Supplement: Supplementary file 8 — Source data Fig. 7 [file 44318_2024_192_MOESM8_ESM.zip › Figure7/Figure7a/PQBP3-EGFP/Fibrillarin.tif]

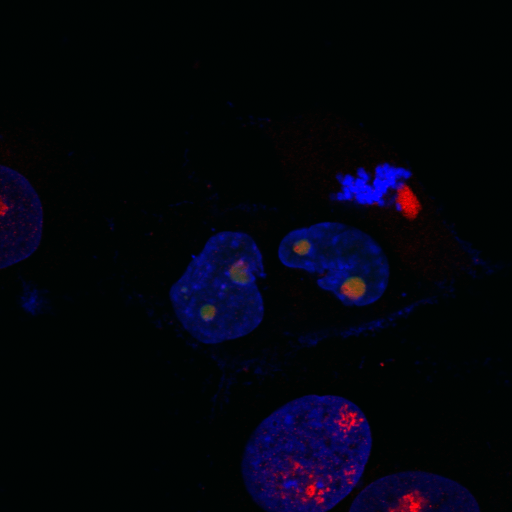

Supplement: Supplementary file 8 — Source data Fig. 7 [file 44318_2024_192_MOESM8_ESM.zip › Figure7/Figure7a/PQBP3-EGFP/PQBP3-EGFP+Fibrillarin+DAPI.tif]

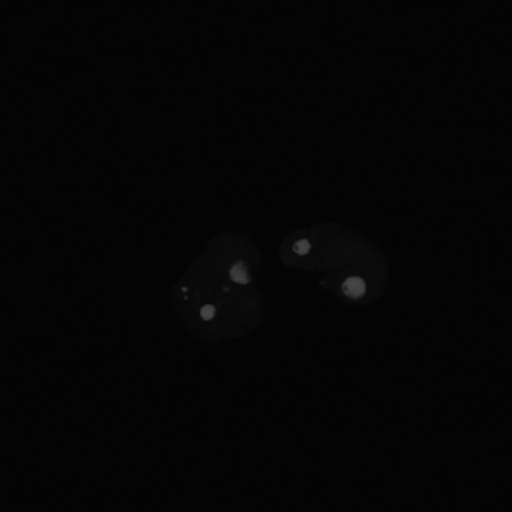

Supplement: Supplementary file 8 — Source data Fig. 7 [file 44318_2024_192_MOESM8_ESM.zip › Figure7/Figure7a/PQBP3-EGFP/PQBP3.tif]

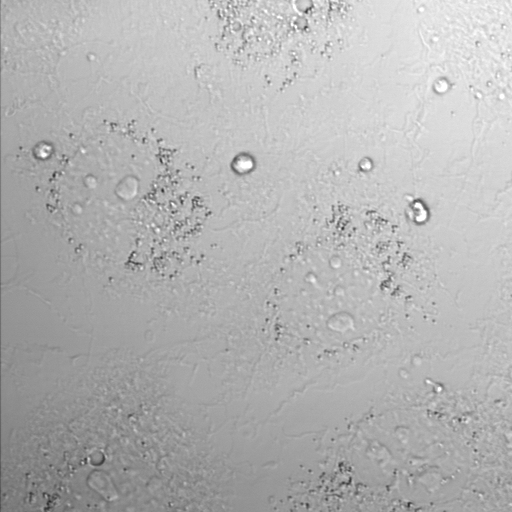

Supplement: Supplementary file 8 — Source data Fig. 7 [file 44318_2024_192_MOESM8_ESM.zip › Figure7/Figure7b/EGFP/Bright field.tif]

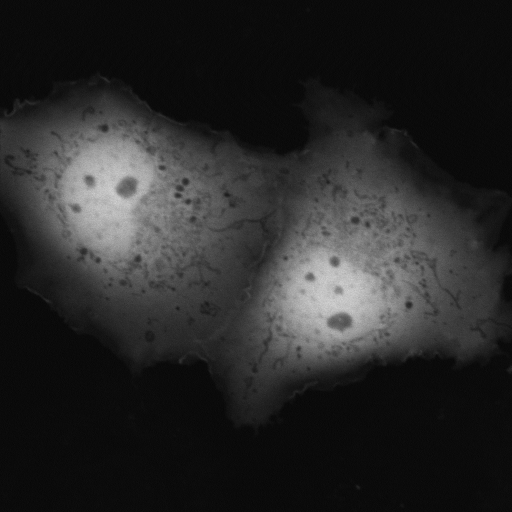

Supplement: Supplementary file 8 — Source data Fig. 7 [file 44318_2024_192_MOESM8_ESM.zip › Figure7/Figure7b/EGFP/EGFP.tif]

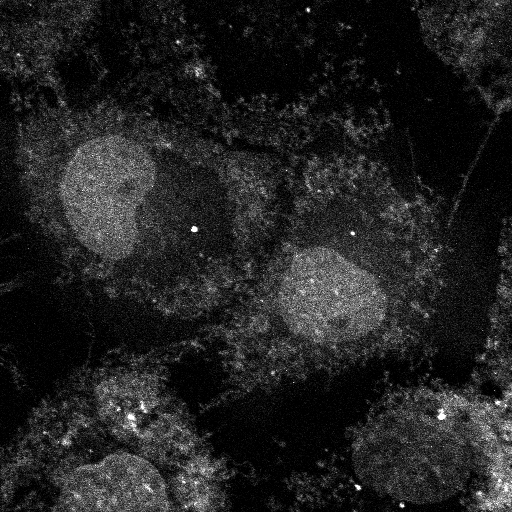

Supplement: Supplementary file 8 — Source data Fig. 7 [file 44318_2024_192_MOESM8_ESM.zip › Figure7/Figure7b/EGFP/Hoechst 33342.tif]

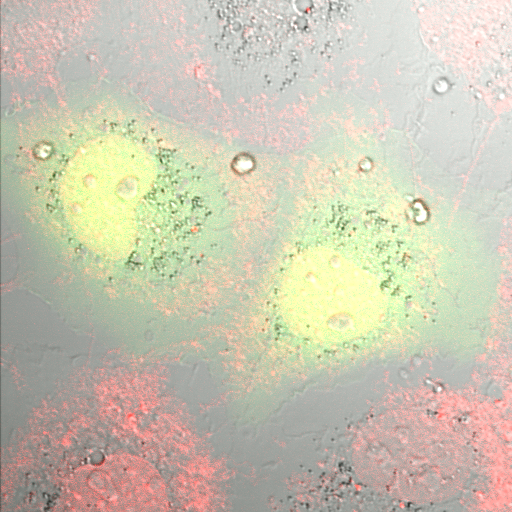

Supplement: Supplementary file 8 — Source data Fig. 7 [file 44318_2024_192_MOESM8_ESM.zip › Figure7/Figure7b/EGFP/Merge.tif]

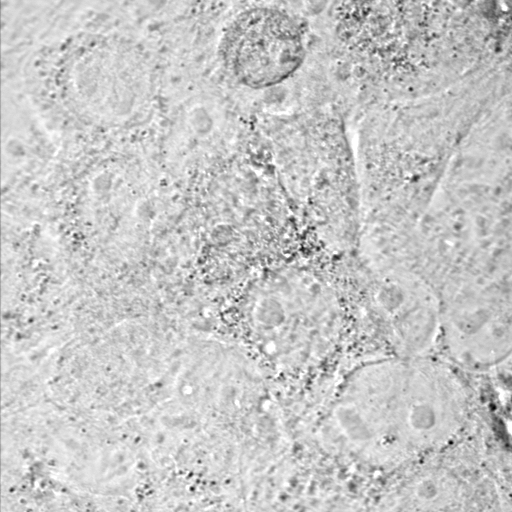

Supplement: Supplementary file 8 — Source data Fig. 7 [file 44318_2024_192_MOESM8_ESM.zip › Figure7/Figure7b/EGFP-PQBP3/Bright field.tif]

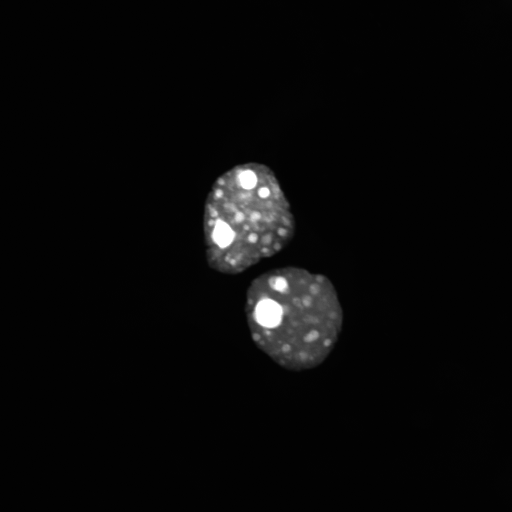

Supplement: Supplementary file 8 — Source data Fig. 7 [file 44318_2024_192_MOESM8_ESM.zip › Figure7/Figure7b/EGFP-PQBP3/EGFP-PQBP3.tif]

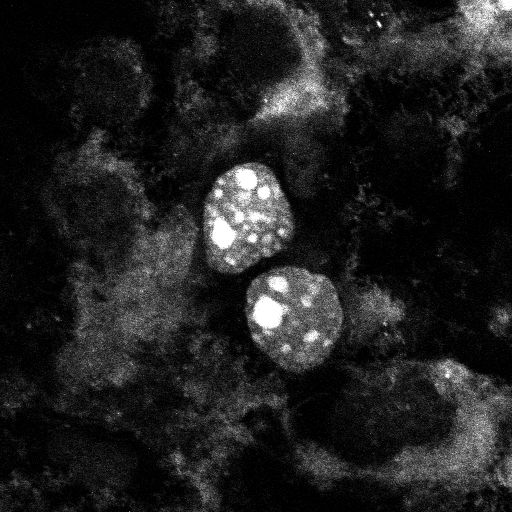

Supplement: Supplementary file 8 — Source data Fig. 7 [file 44318_2024_192_MOESM8_ESM.zip › Figure7/Figure7b/EGFP-PQBP3/Hoechst 33342.tif]

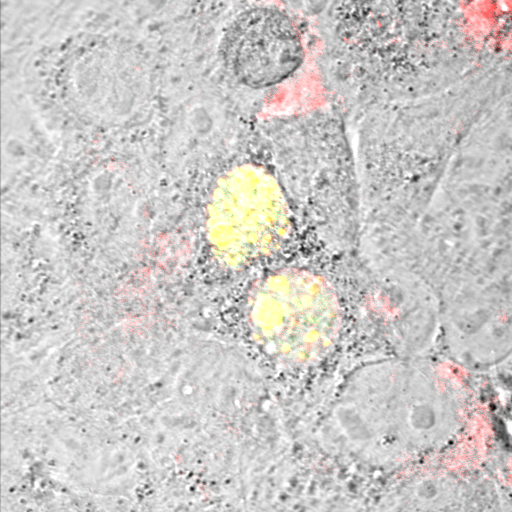

Supplement: Supplementary file 8 — Source data Fig. 7 [file 44318_2024_192_MOESM8_ESM.zip › Figure7/Figure7b/EGFP-PQBP3/Merge.tif]

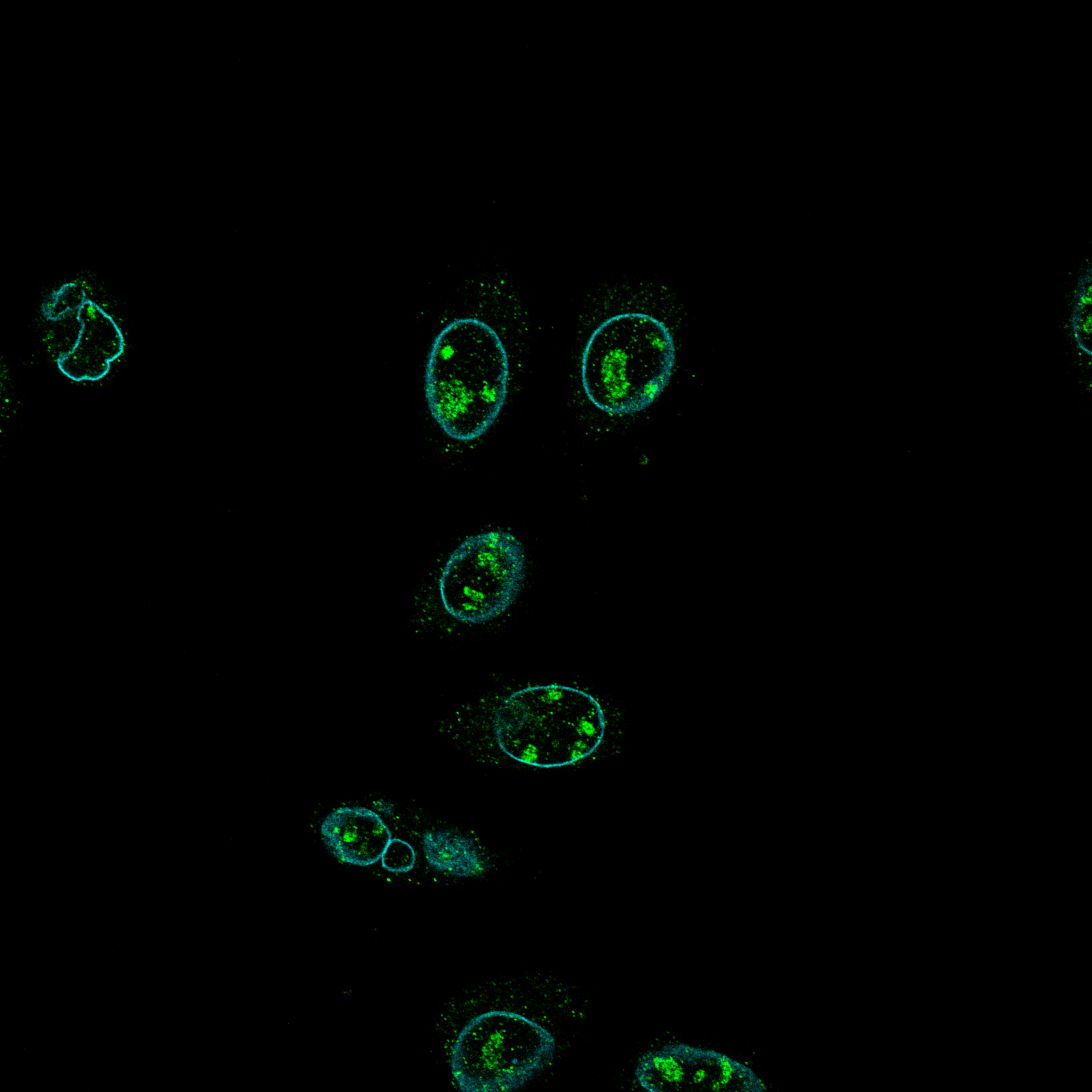

Supplement: Supplementary file 9 — Source data Fig. 8 [file 44318_2024_192_MOESM9_ESM.zip › Figure8/Figure8b/H2O2_negative/Lower images/PQBP3+Lamin B1.tif]

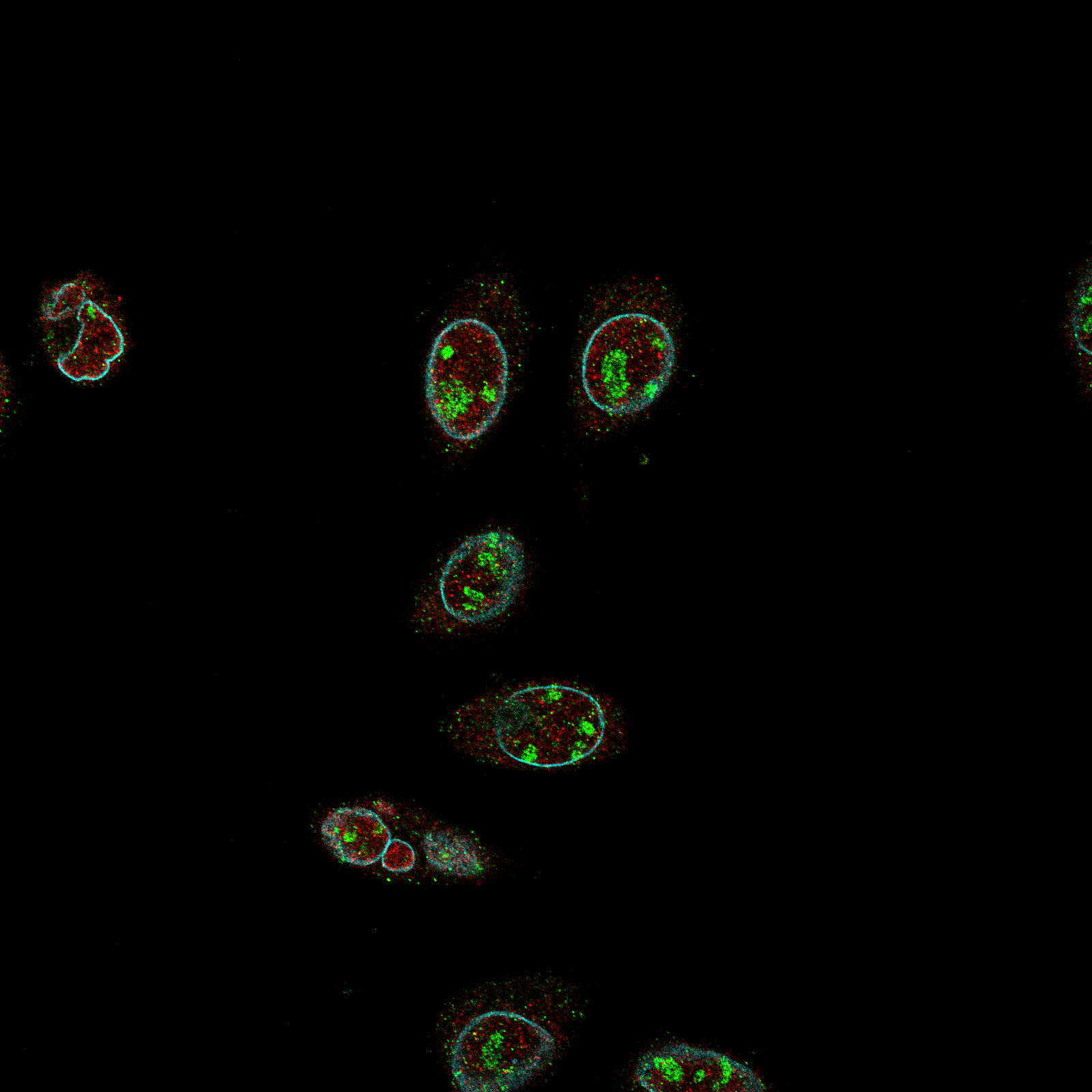

Supplement: Supplementary file 9 — Source data Fig. 8 [file 44318_2024_192_MOESM9_ESM.zip › Figure8/Figure8b/H2O2_negative/Lower images/PQBP3+PSME3+Lamin B1.tif]

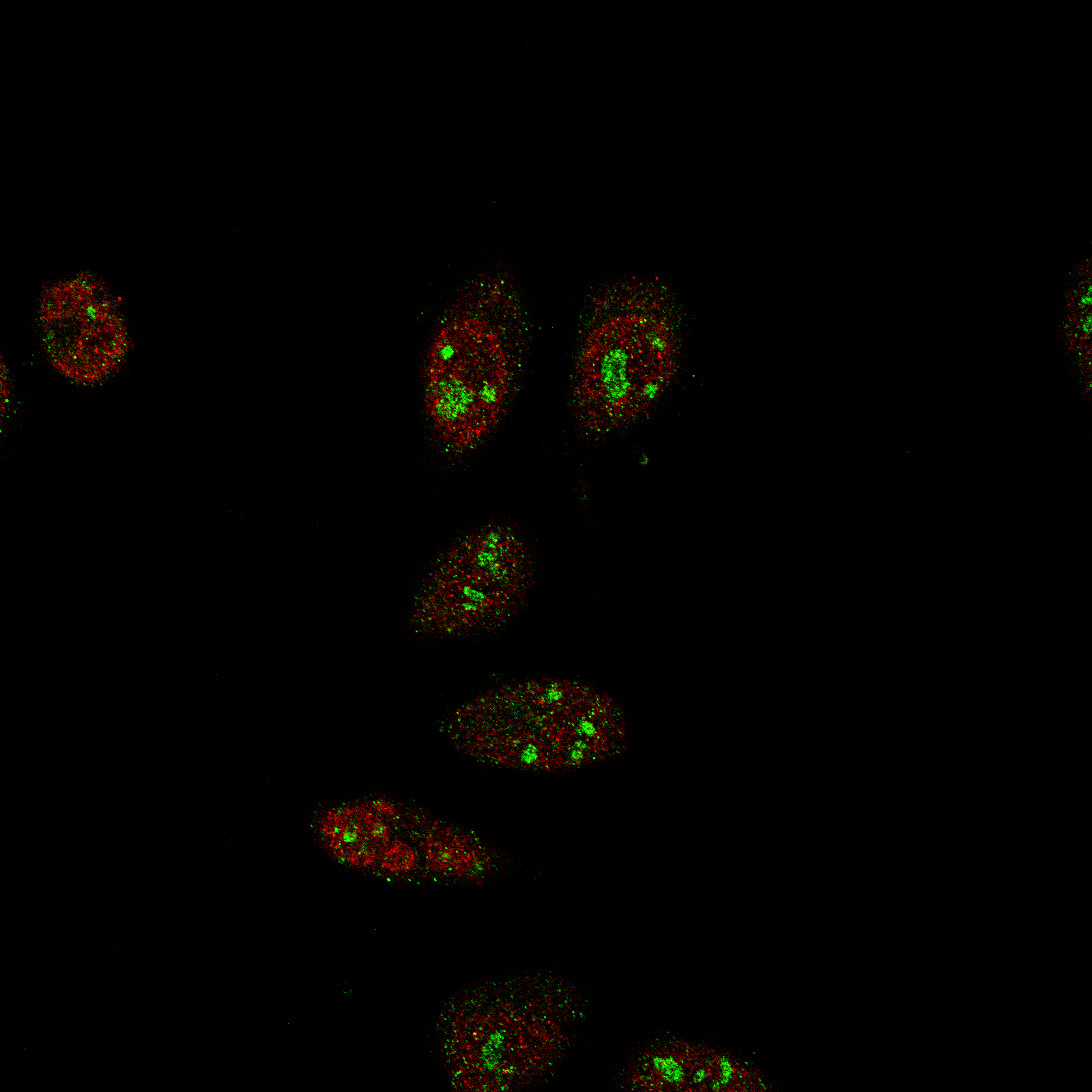

Supplement: Supplementary file 9 — Source data Fig. 8 [file 44318_2024_192_MOESM9_ESM.zip › Figure8/Figure8b/H2O2_negative/Lower images/PQBP3+PSME3.tif]

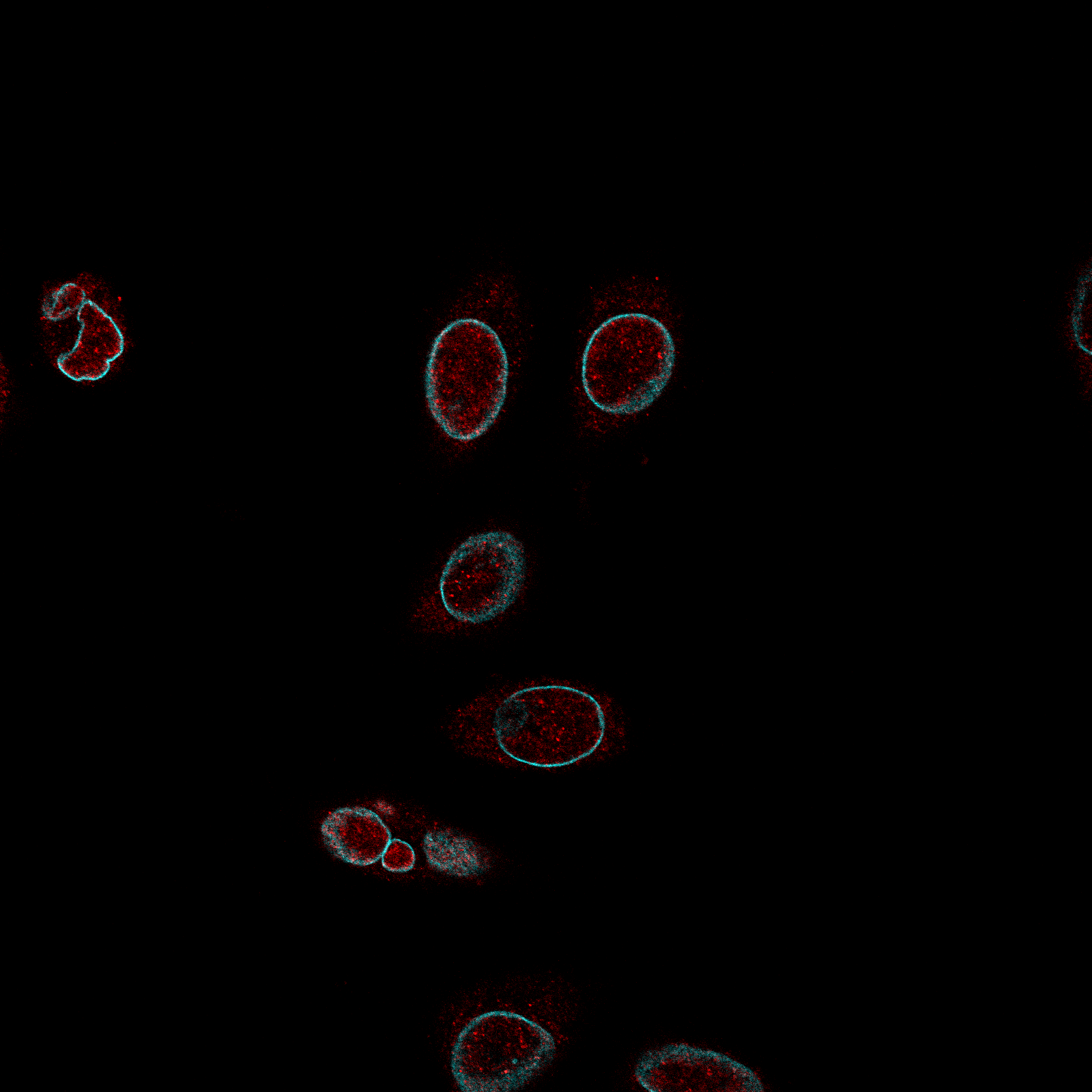

Supplement: Supplementary file 9 — Source data Fig. 8 [file 44318_2024_192_MOESM9_ESM.zip › Figure8/Figure8b/H2O2_negative/Lower images/PSME3+Lamin B1.tif]

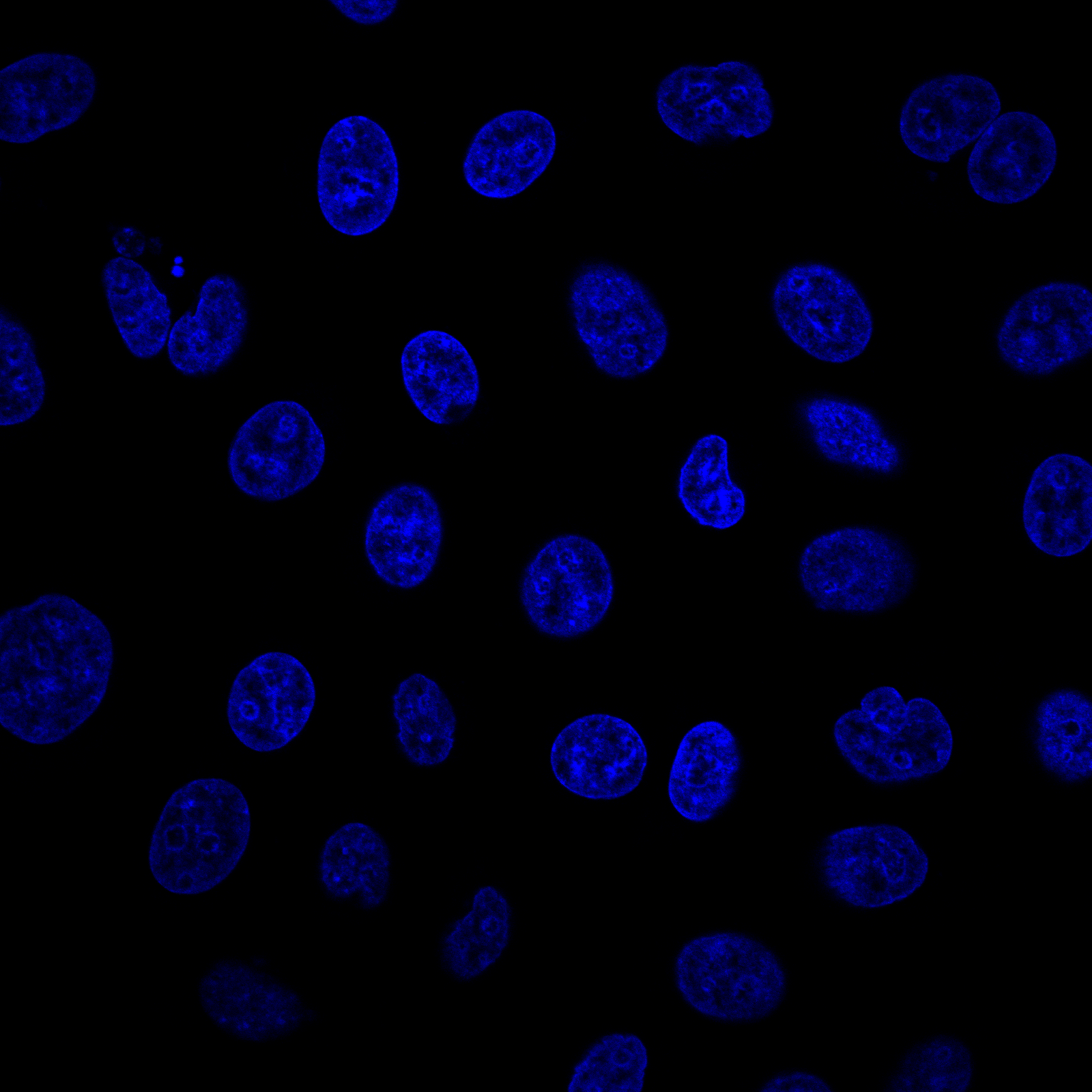

Supplement: Supplementary file 9 — Source data Fig. 8 [file 44318_2024_192_MOESM9_ESM.zip › Figure8/Figure8b/H2O2_negative/Upper images/DAPI.tif]

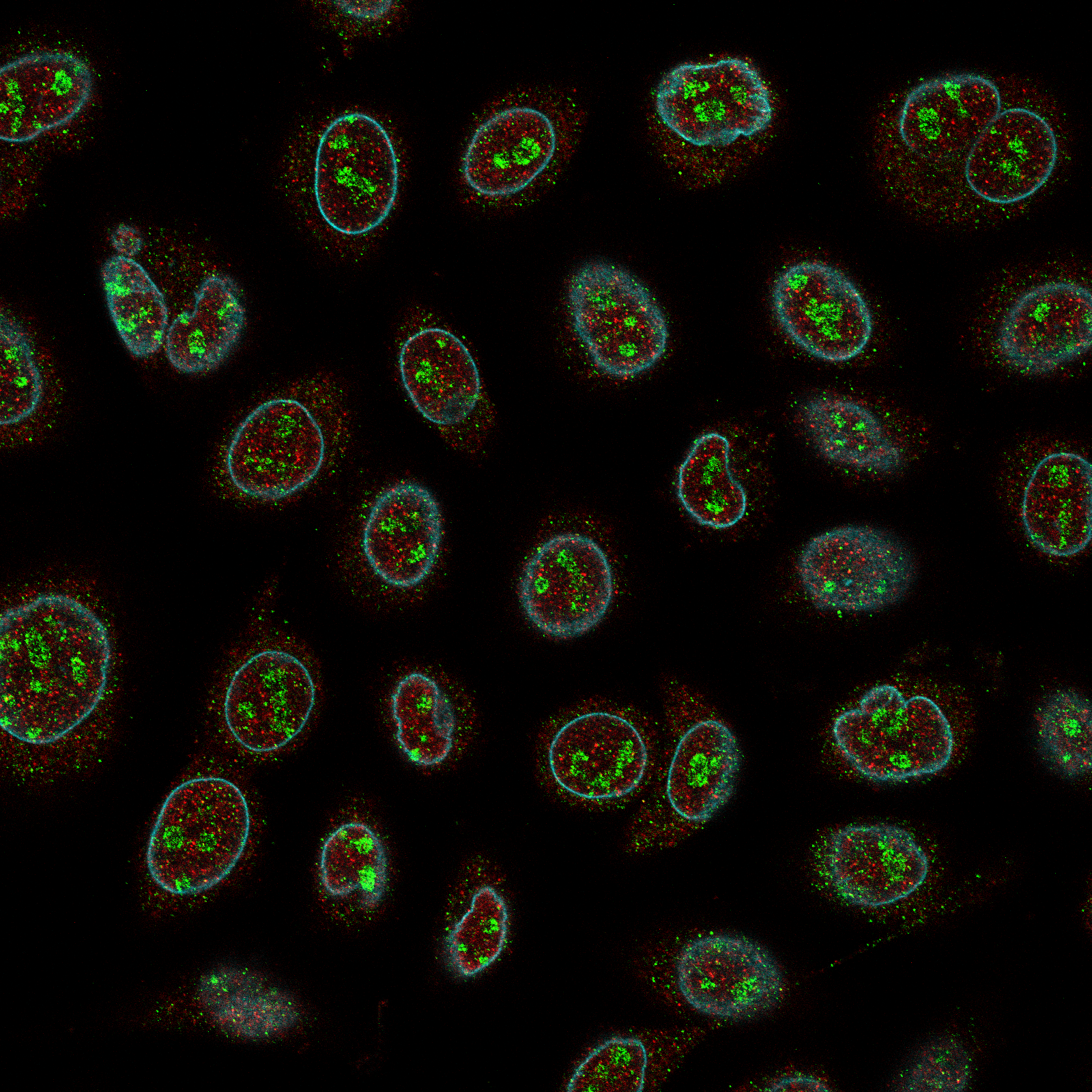

Supplement: Supplementary file 9 — Source data Fig. 8 [file 44318_2024_192_MOESM9_ESM.zip › Figure8/Figure8b/H2O2_negative/Upper images/PQBP3+PSME3+Lamin B1.tif]

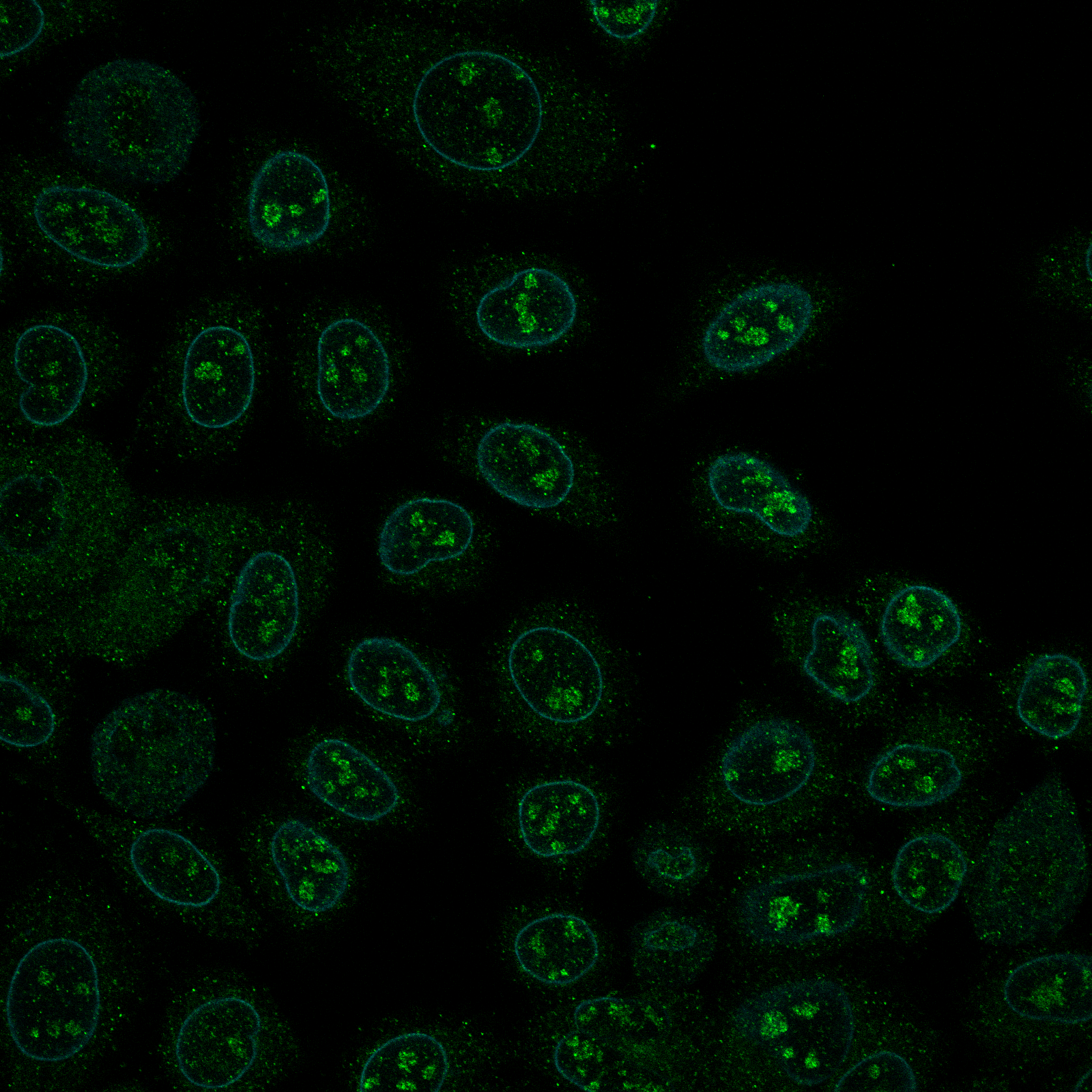

Supplement: Supplementary file 9 — Source data Fig. 8 [file 44318_2024_192_MOESM9_ESM.zip › Figure8/Figure8b/H2O2_positive/Lower images/PQBP3+Lamin B1.tif]

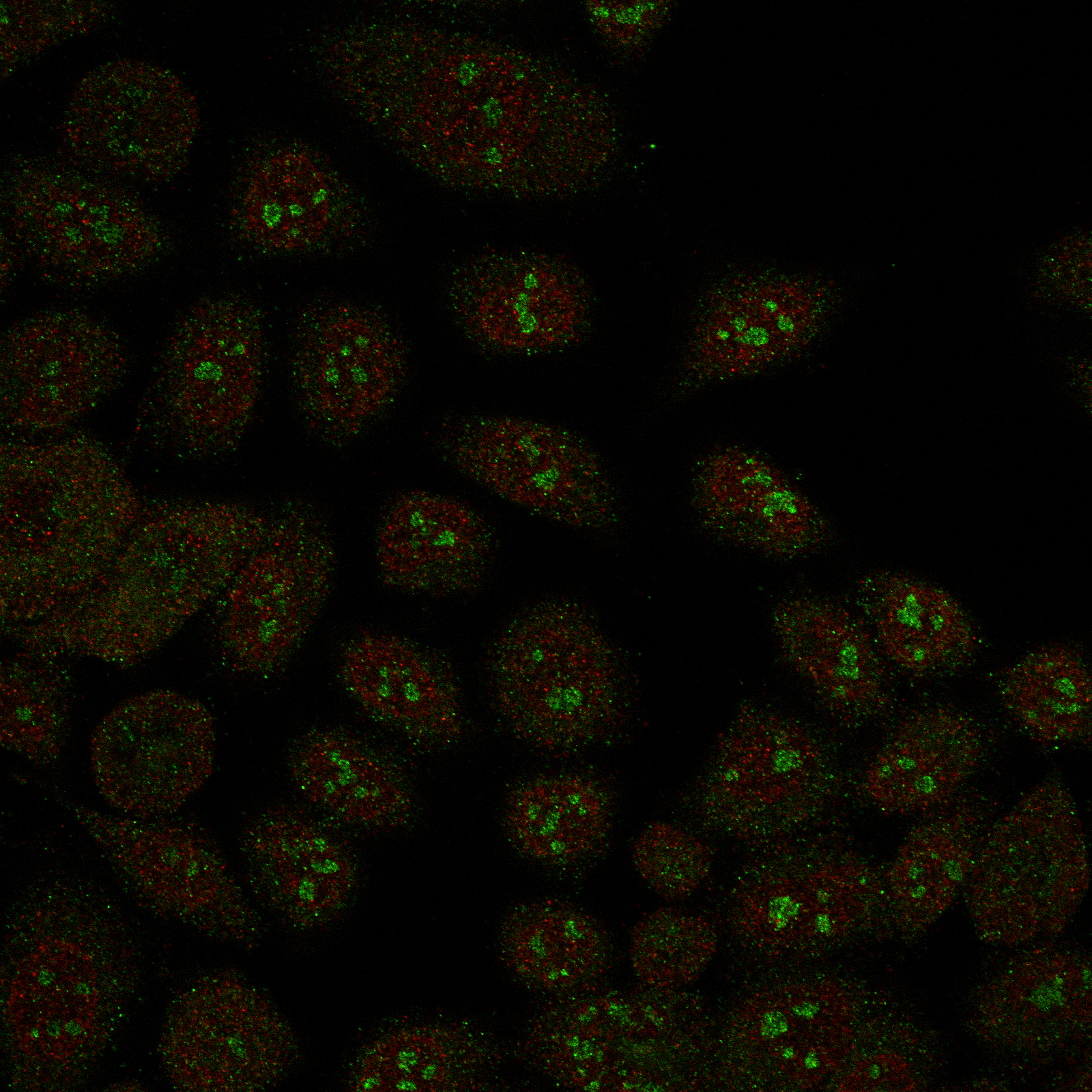

Supplement: Supplementary file 9 — Source data Fig. 8 [file 44318_2024_192_MOESM9_ESM.zip › Figure8/Figure8b/H2O2_positive/Lower images/PQBP3+PSME3.tif]

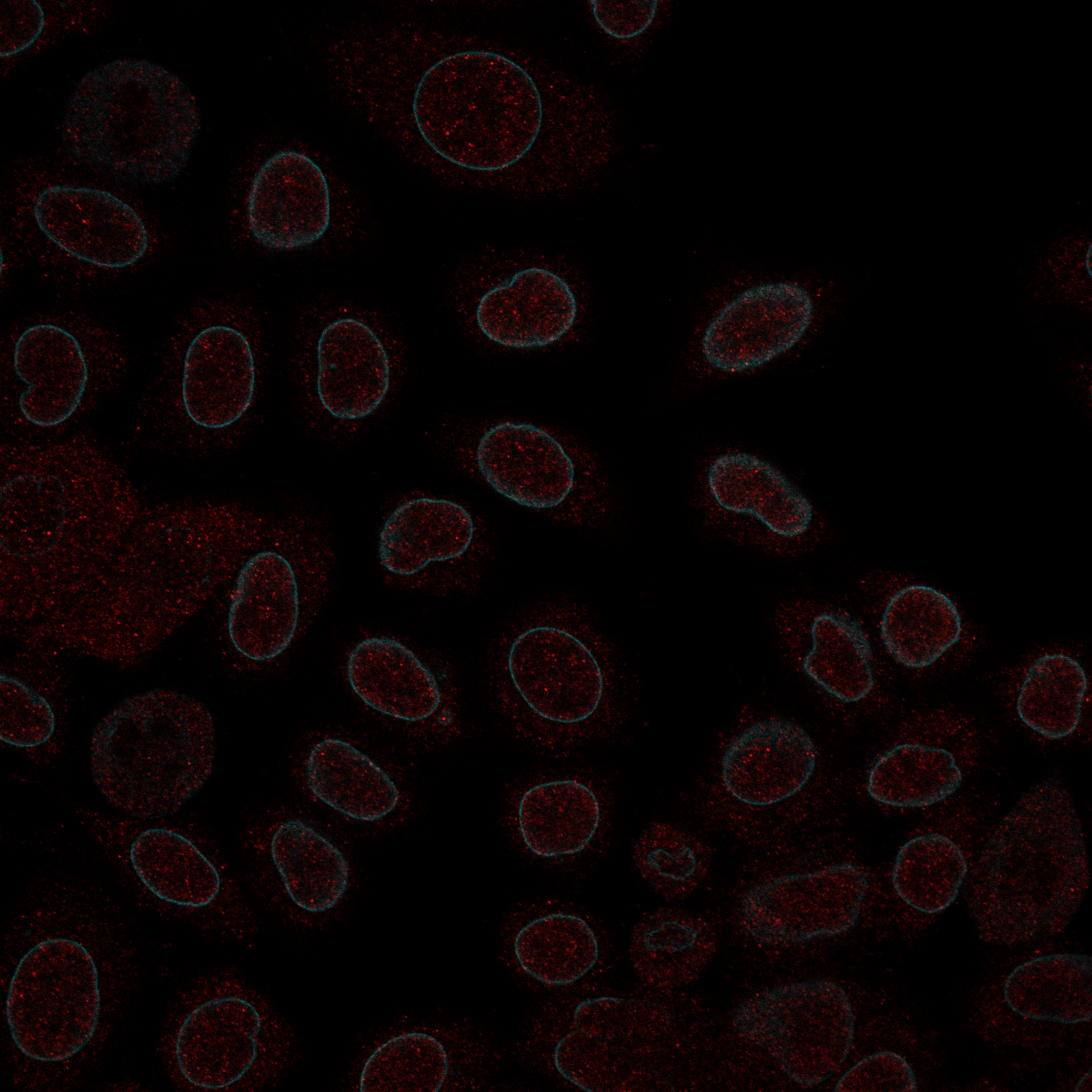

Supplement: Supplementary file 9 — Source data Fig. 8 [file 44318_2024_192_MOESM9_ESM.zip › Figure8/Figure8b/H2O2_positive/Lower images/PSME3+Lamin B1.tif]

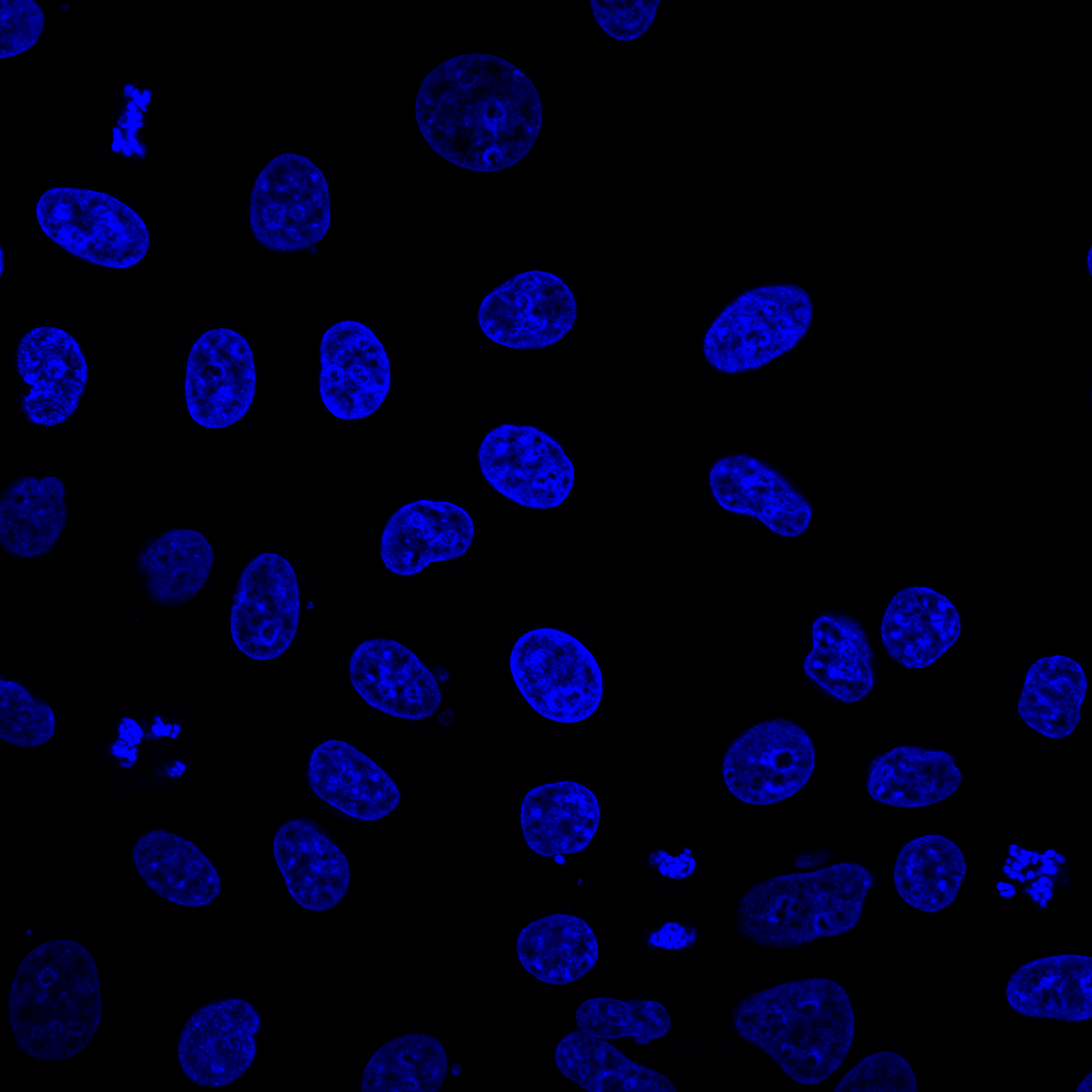

Supplement: Supplementary file 9 — Source data Fig. 8 [file 44318_2024_192_MOESM9_ESM.zip › Figure8/Figure8b/H2O2_positive/Upper images/DAPI.tif]

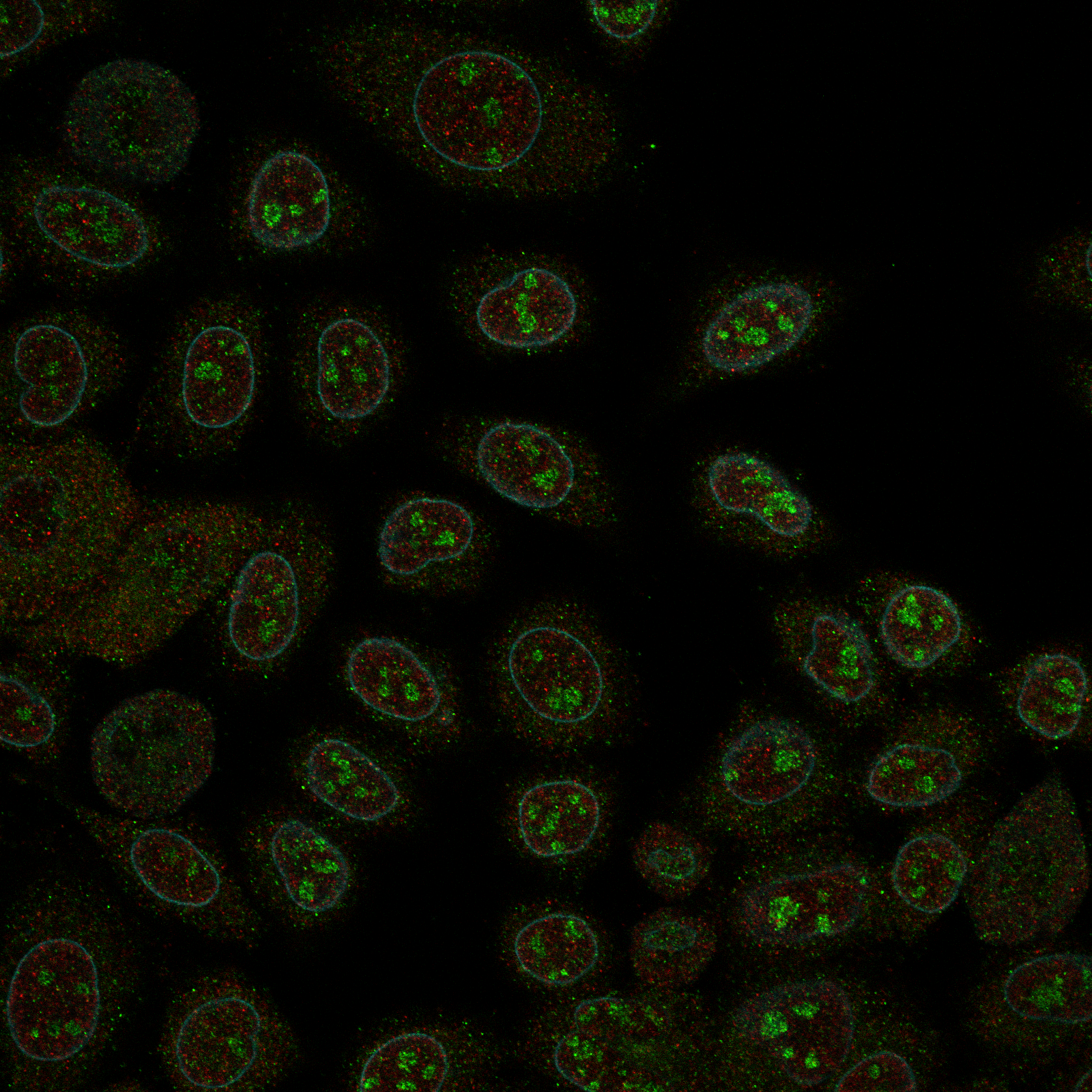

Supplement: Supplementary file 9 — Source data Fig. 8 [file 44318_2024_192_MOESM9_ESM.zip › Figure8/Figure8b/H2O2_positive/Upper images/PQBP3+PSME3+Lamin B1.tif]

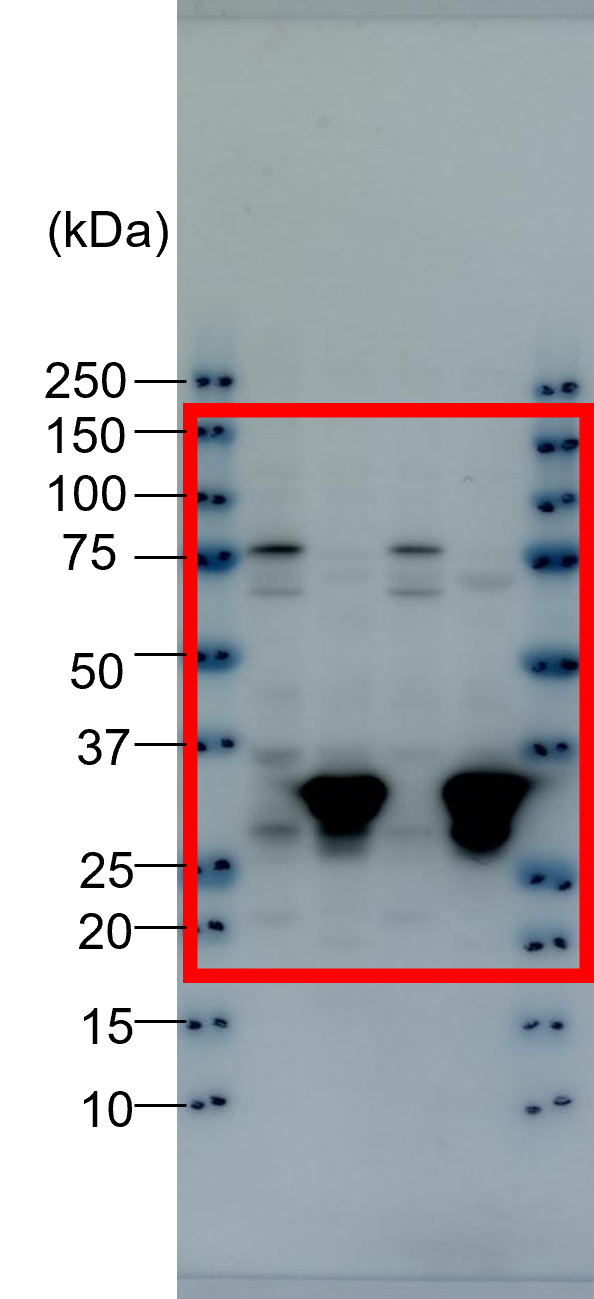

Supplement: Supplementary file 9 — Source data Fig. 8 [file 44318_2024_192_MOESM9_ESM.zip › Figure8/Figure8c/Input EGFP.tif]

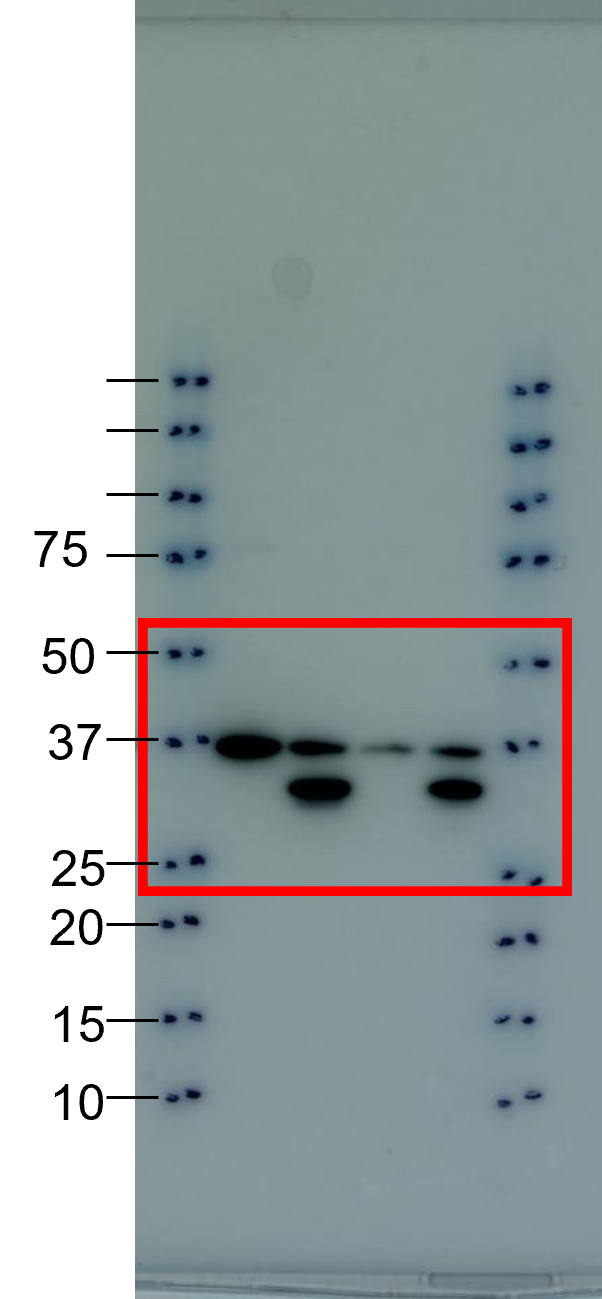

Supplement: Supplementary file 9 — Source data Fig. 8 [file 44318_2024_192_MOESM9_ESM.zip › Figure8/Figure8c/Input GAPDH.tif]

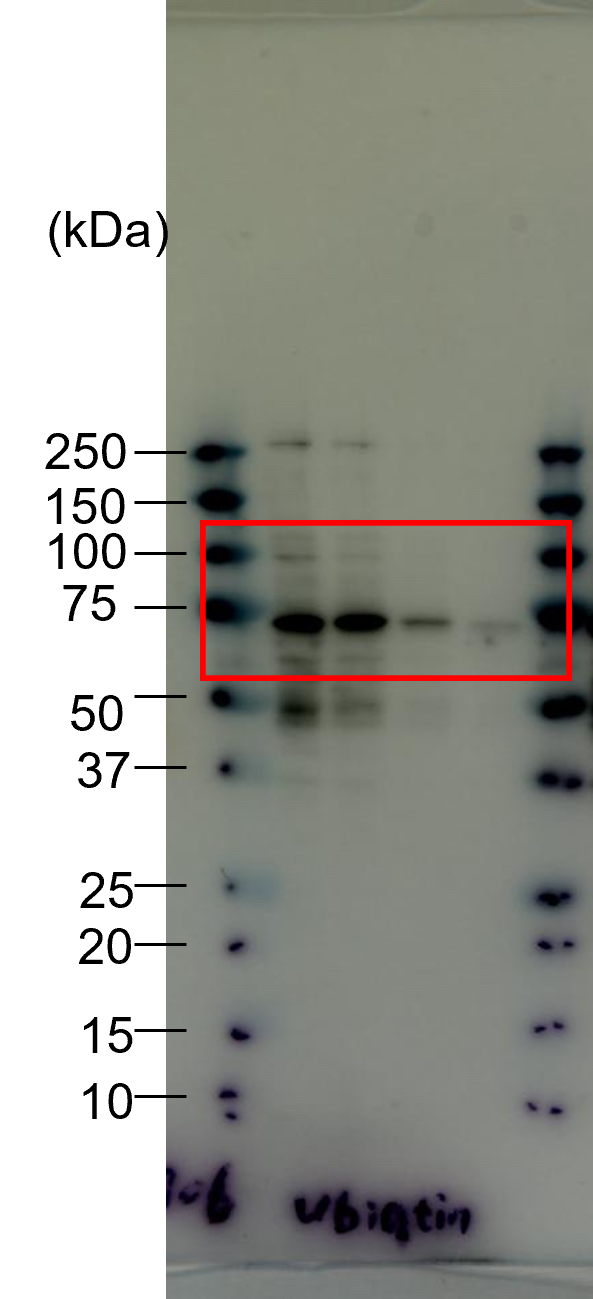

Supplement: Supplementary file 9 — Source data Fig. 8 [file 44318_2024_192_MOESM9_ESM.zip › Figure8/Figure8c/Input laminB1.tif]

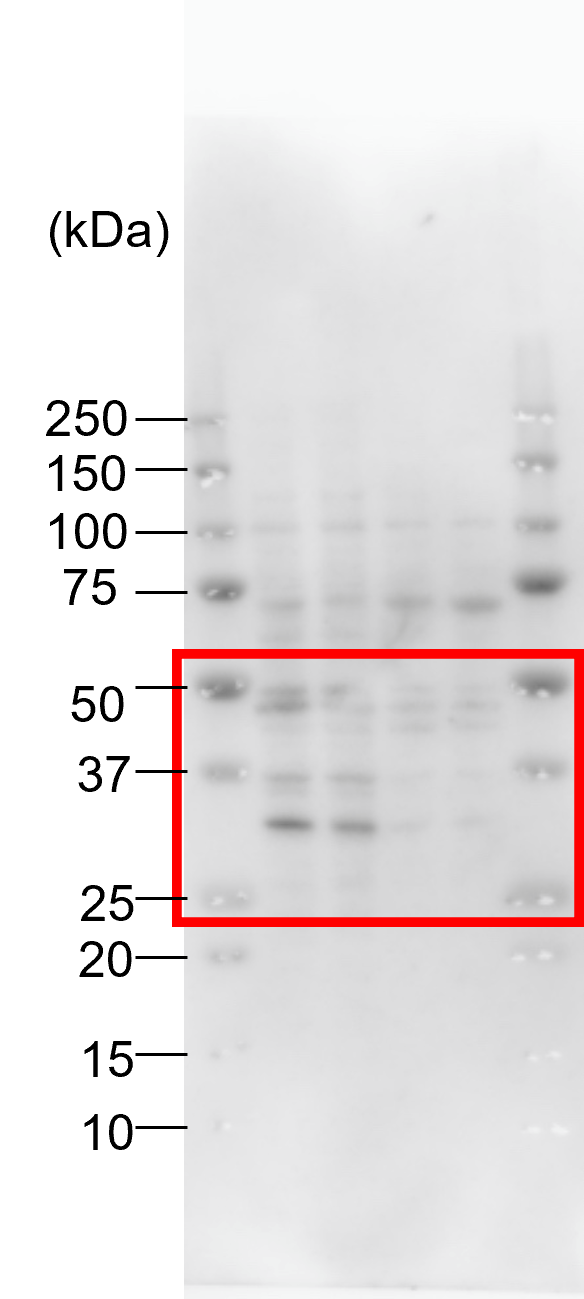

Supplement: Supplementary file 9 — Source data Fig. 8 [file 44318_2024_192_MOESM9_ESM.zip › Figure8/Figure8c/Input PSME3.tif]

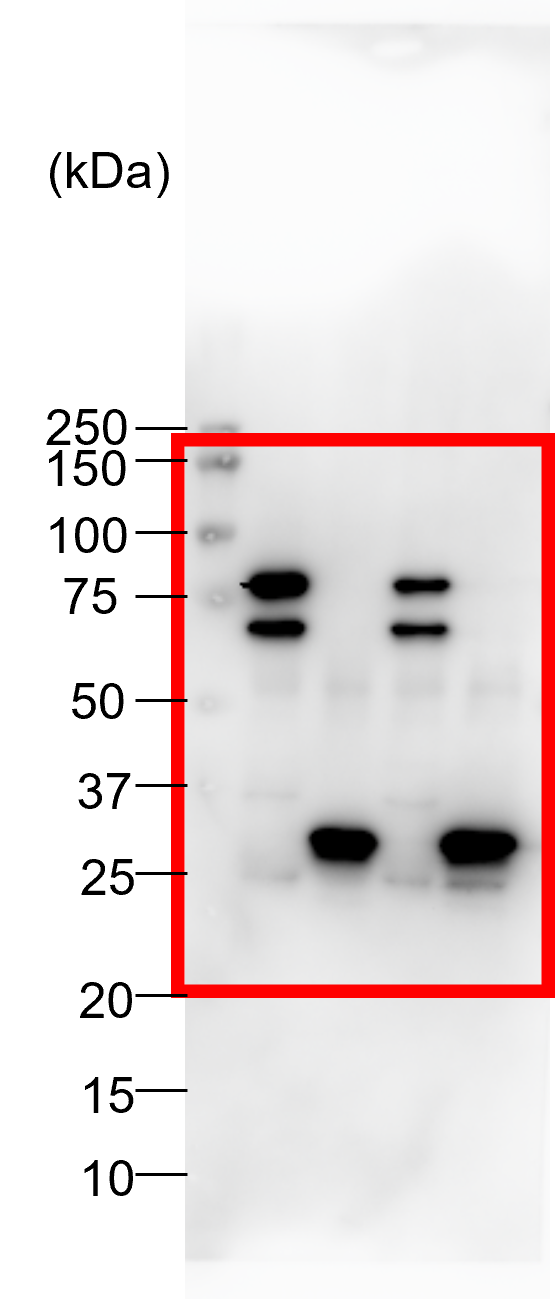

Supplement: Supplementary file 9 — Source data Fig. 8 [file 44318_2024_192_MOESM9_ESM.zip › Figure8/Figure8c/IP-EGFP EGFP.tif]

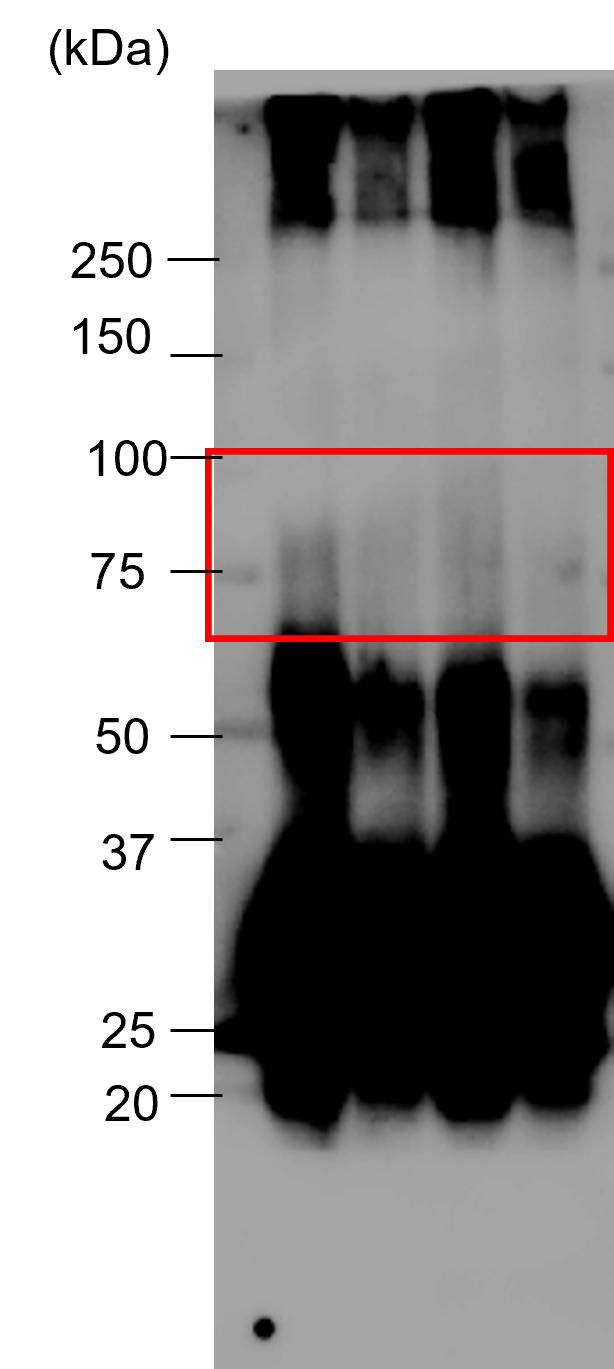

Supplement: Supplementary file 9 — Source data Fig. 8 [file 44318_2024_192_MOESM9_ESM.zip › Figure8/Figure8c/IP-EGFP LaminB1.tif]

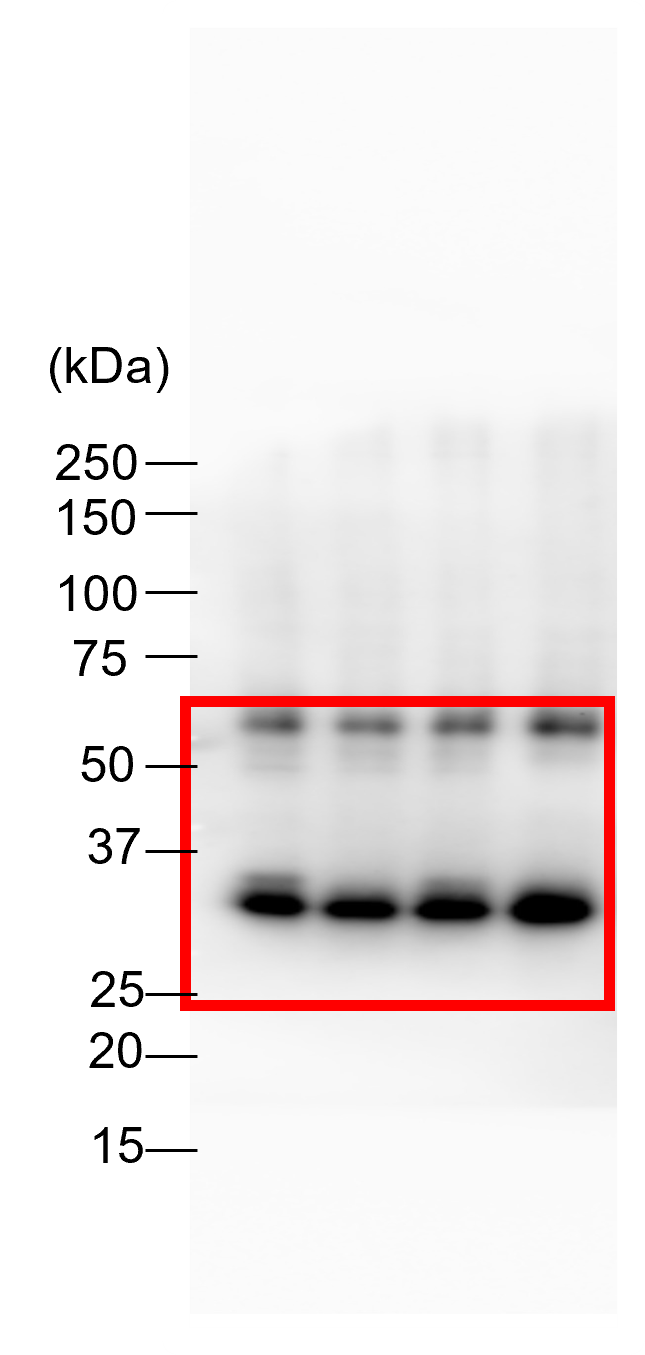

Supplement: Supplementary file 9 — Source data Fig. 8 [file 44318_2024_192_MOESM9_ESM.zip › Figure8/Figure8c/IP-EGFP PSME3.tif]

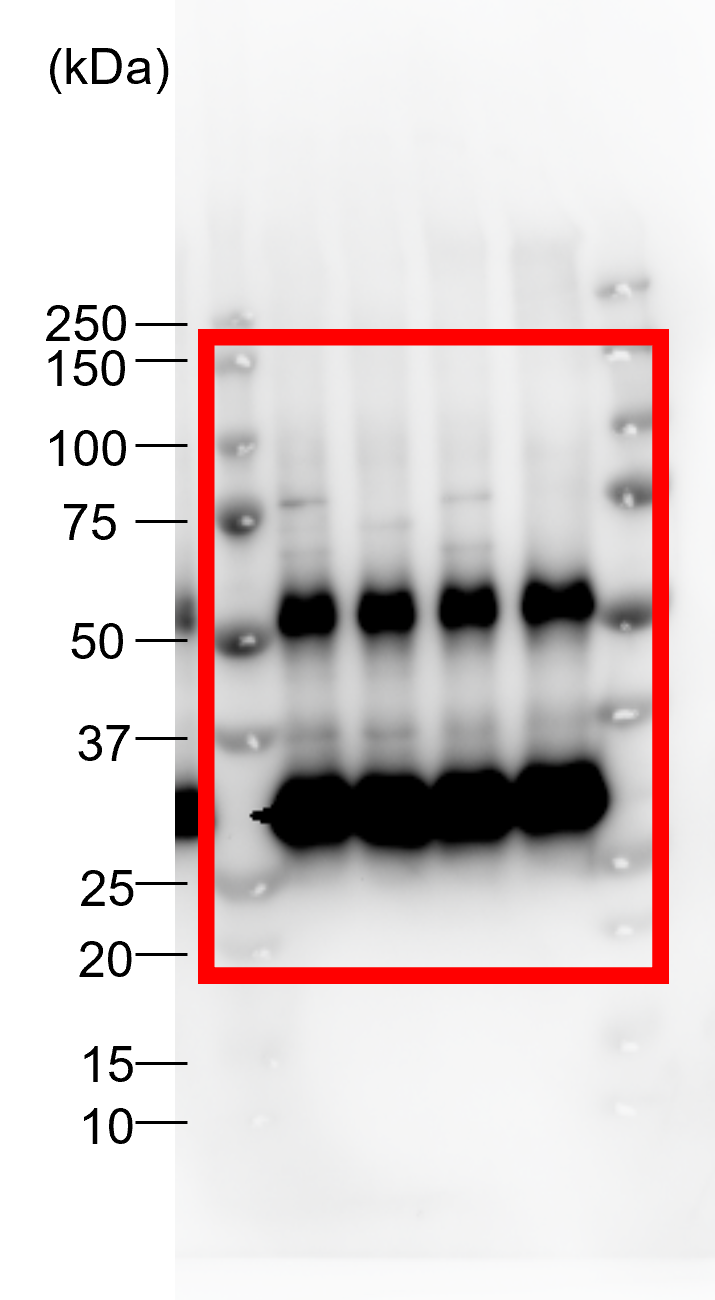

Supplement: Supplementary file 9 — Source data Fig. 8 [file 44318_2024_192_MOESM9_ESM.zip › Figure8/Figure8c/IP-LaminB1 EGFP.tif]

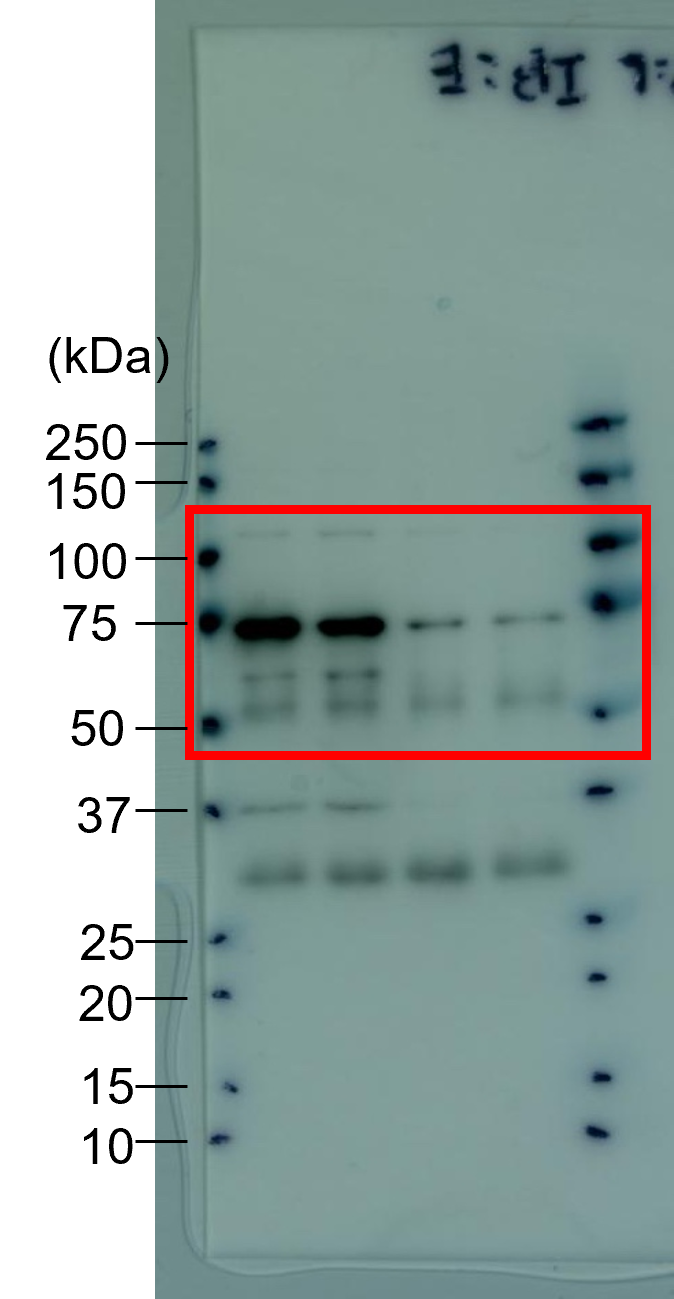

Supplement: Supplementary file 9 — Source data Fig. 8 [file 44318_2024_192_MOESM9_ESM.zip › Figure8/Figure8c/IP-LaminB1 LaminB1.tif]

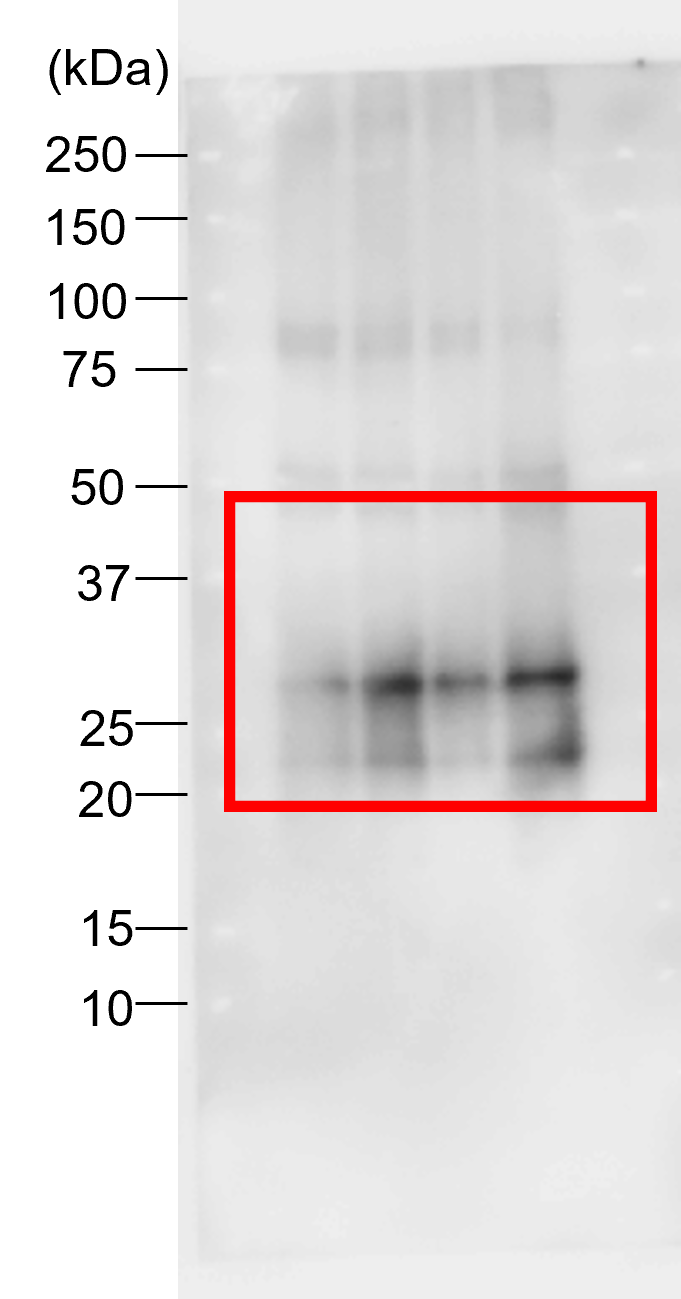

Supplement: Supplementary file 9 — Source data Fig. 8 [file 44318_2024_192_MOESM9_ESM.zip › Figure8/Figure8c/IP-LaminB1 PSME3.tif]

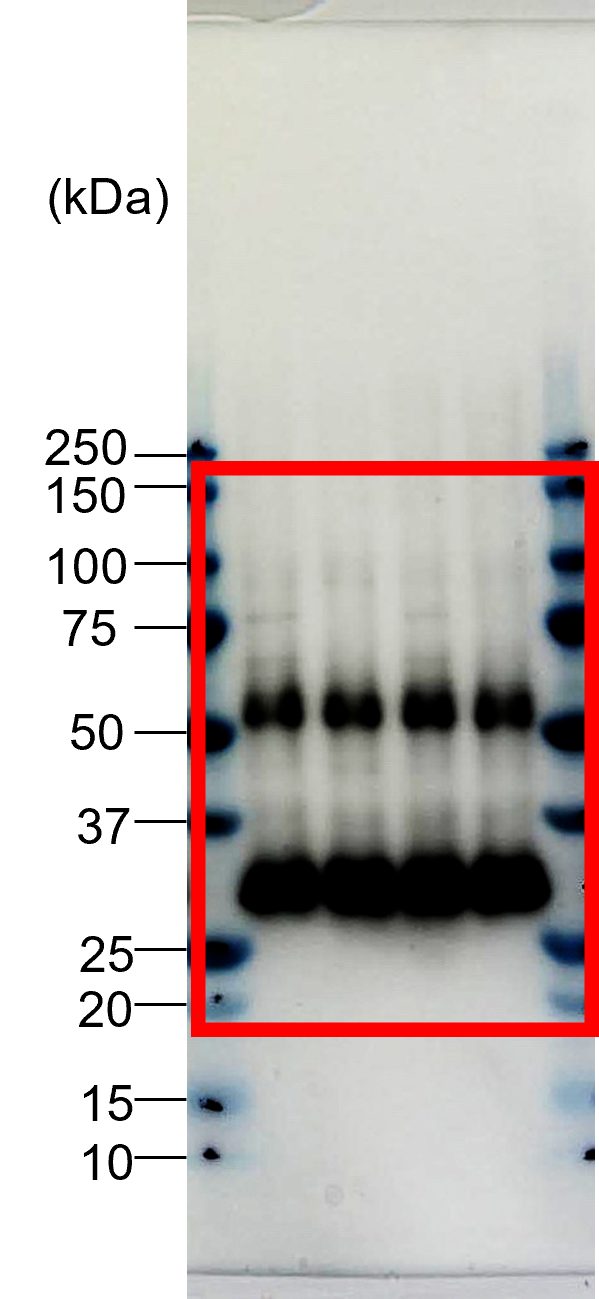

Supplement: Supplementary file 9 — Source data Fig. 8 [file 44318_2024_192_MOESM9_ESM.zip › Figure8/Figure8c/IP-PSME3 EGFP.tif]

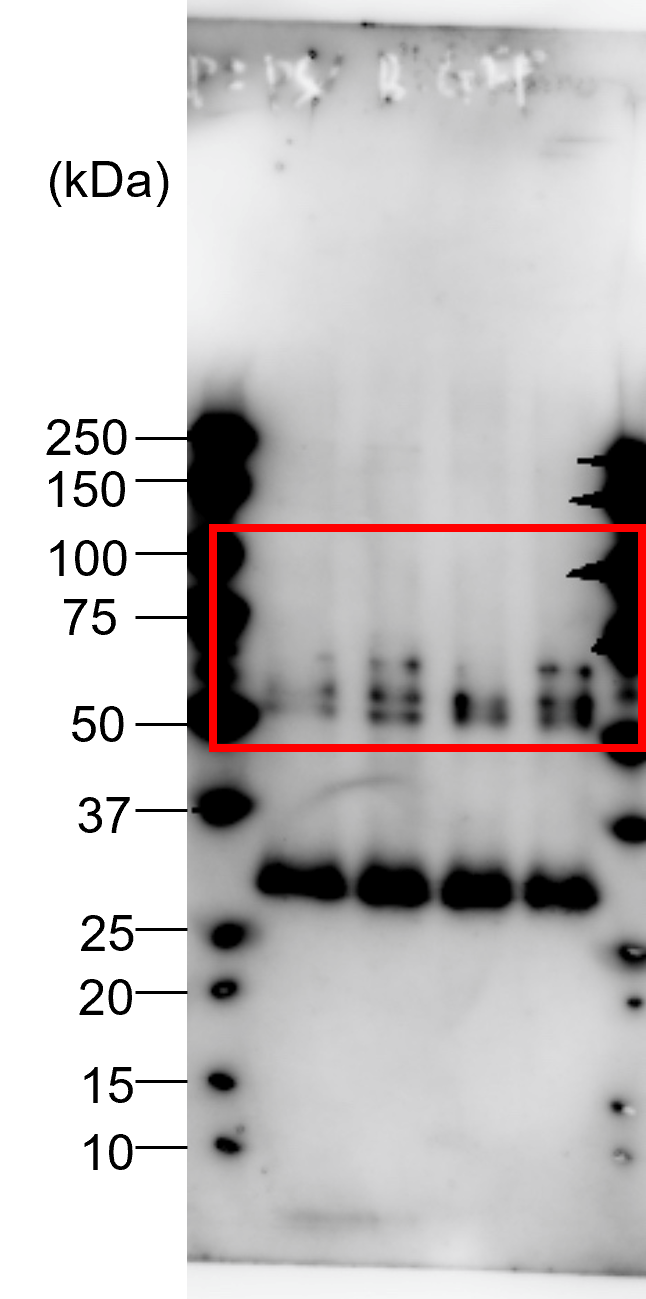

Supplement: Supplementary file 9 — Source data Fig. 8 [file 44318_2024_192_MOESM9_ESM.zip › Figure8/Figure8c/IP-PSME3 LaminB1.tif]

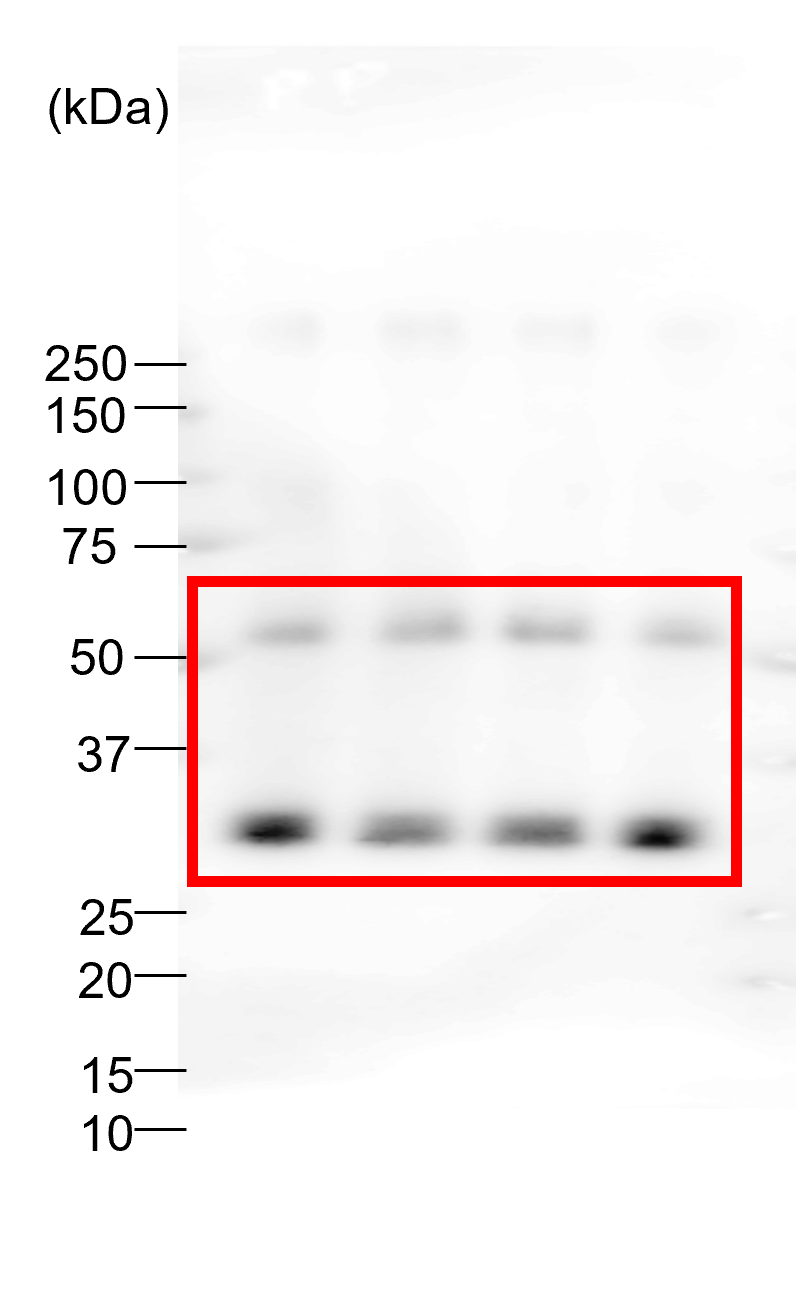

Supplement: Supplementary file 9 — Source data Fig. 8 [file 44318_2024_192_MOESM9_ESM.zip › Figure8/Figure8c/IP-PSME3 PSME3.tif]

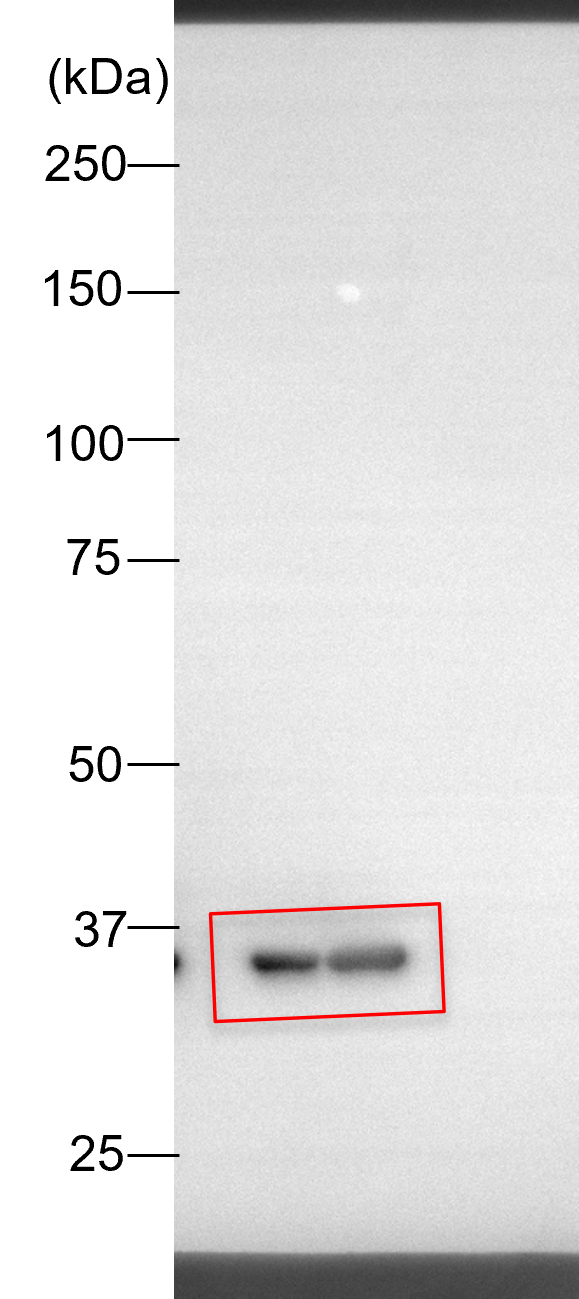

Supplement: Supplementary file 9 — Source data Fig. 8 [file 44318_2024_192_MOESM9_ESM.zip › Figure8/Figure8e/Input GAPDH.tif]

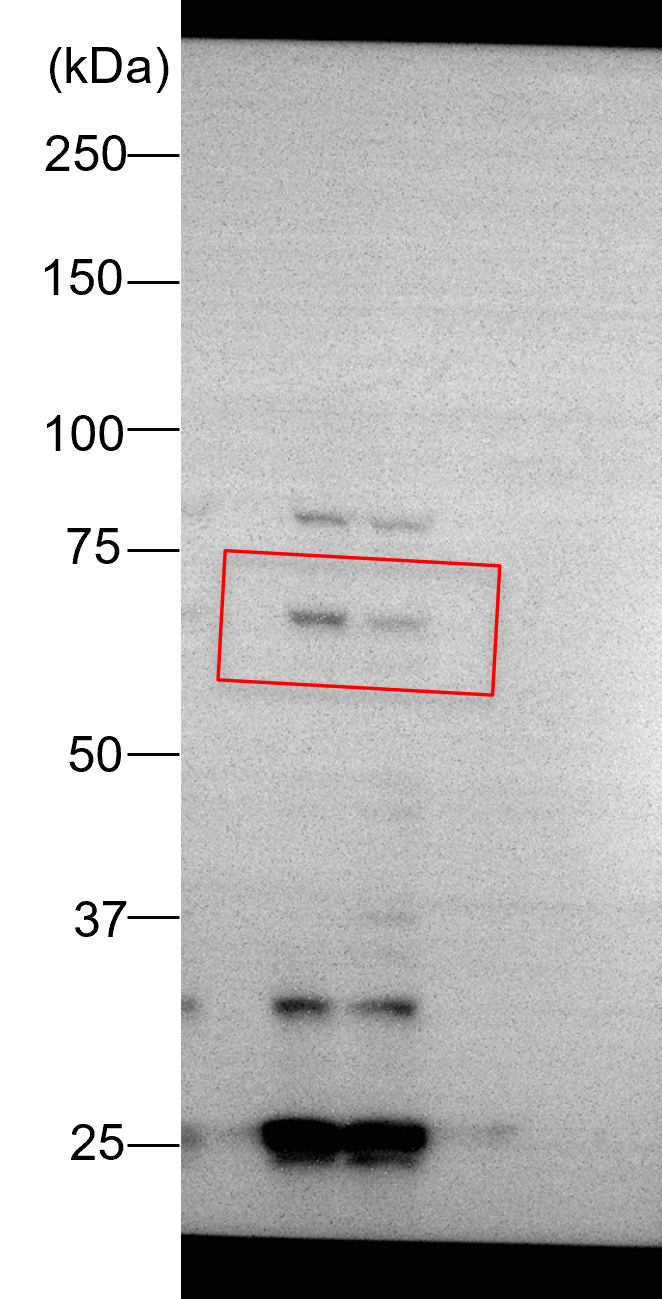

Supplement: Supplementary file 9 — Source data Fig. 8 [file 44318_2024_192_MOESM9_ESM.zip › Figure8/Figure8e/Input Lamin B1.tif]

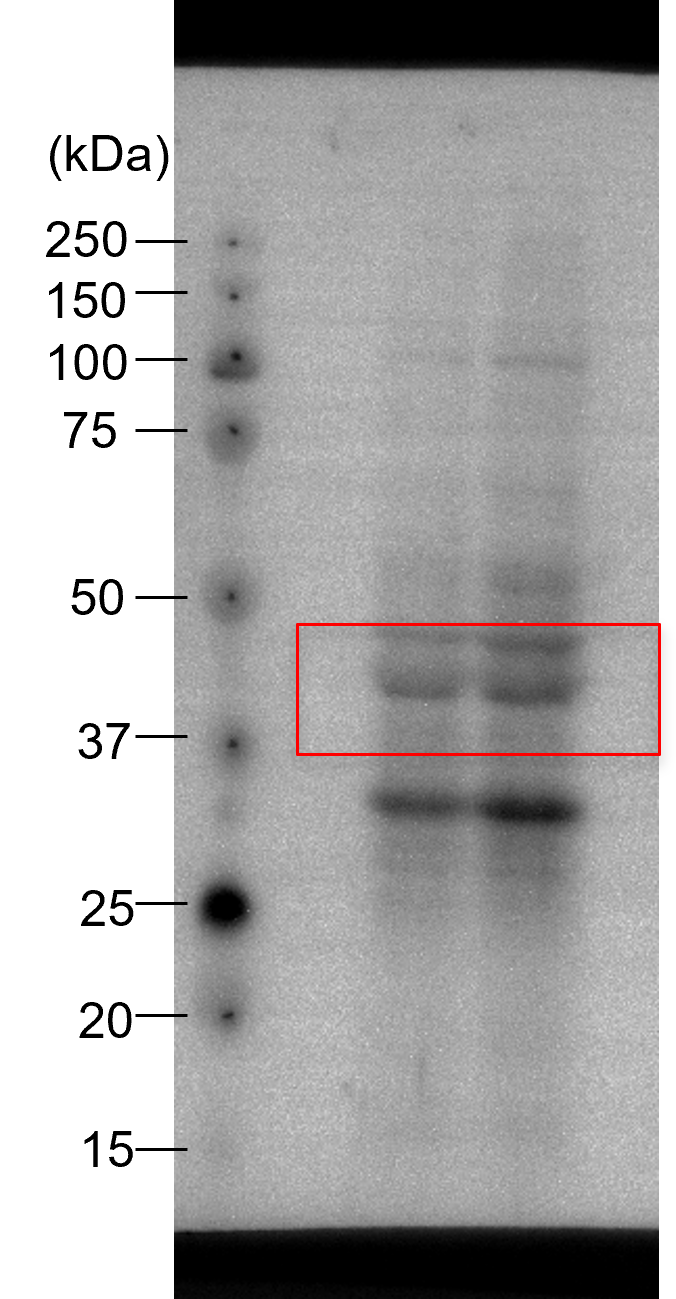

Supplement: Supplementary file 9 — Source data Fig. 8 [file 44318_2024_192_MOESM9_ESM.zip › Figure8/Figure8e/Input PQBP3.tif]

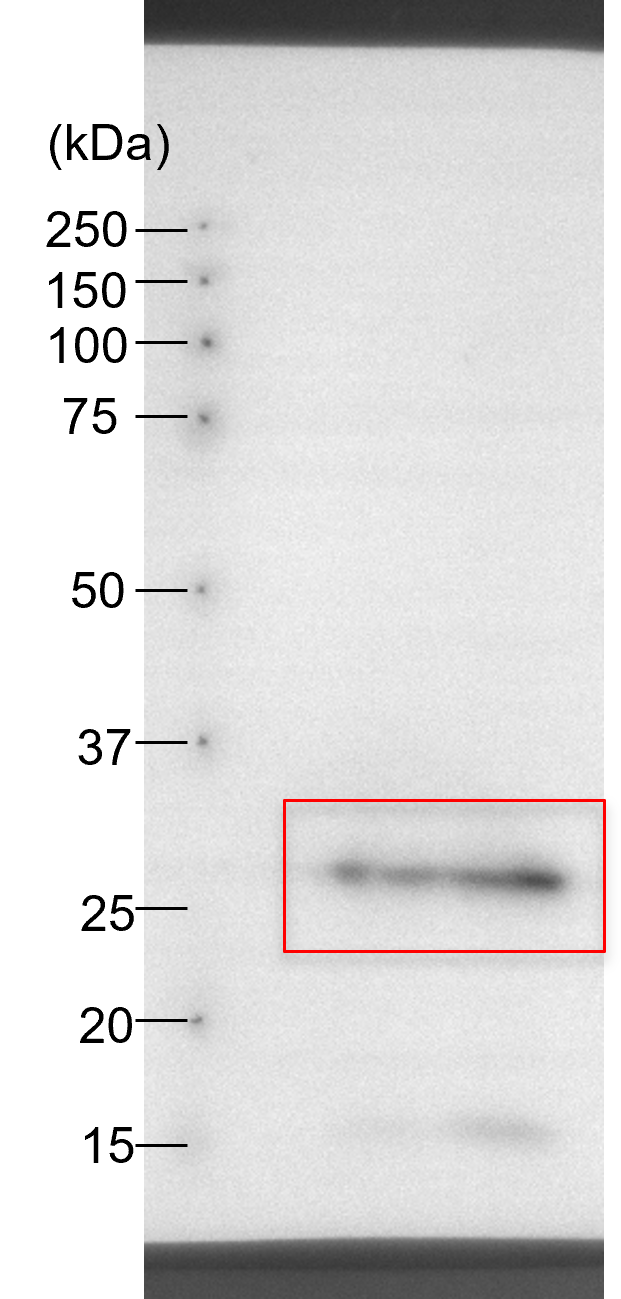

Supplement: Supplementary file 9 — Source data Fig. 8 [file 44318_2024_192_MOESM9_ESM.zip › Figure8/Figure8e/Input PSME3.tif]

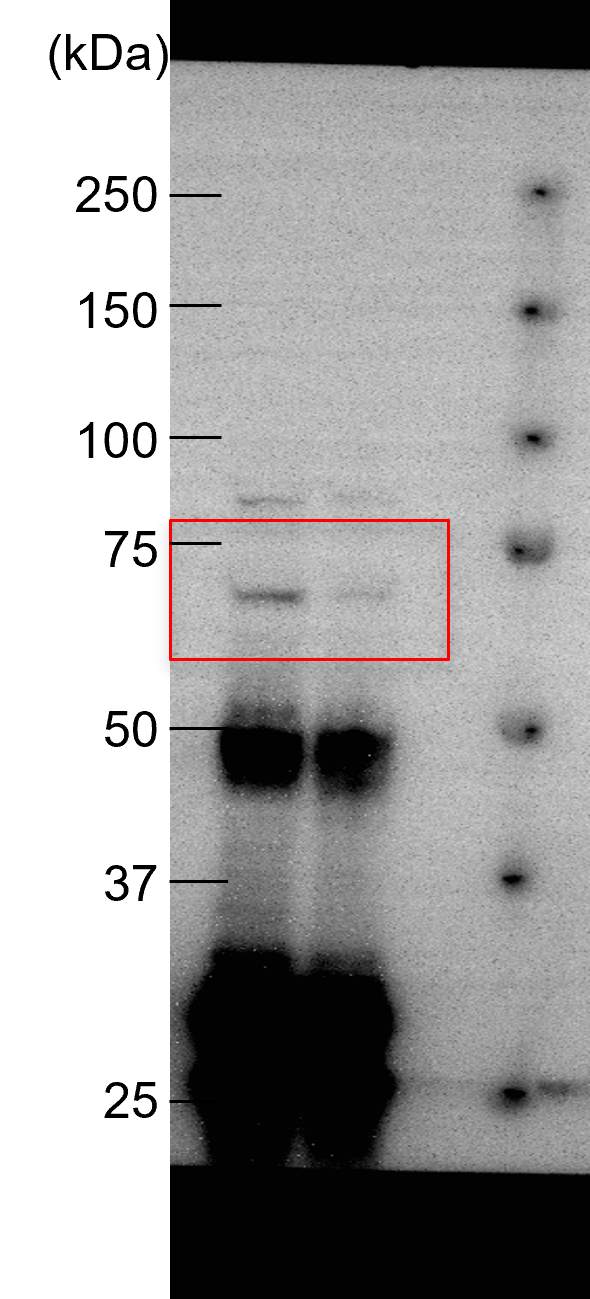

Supplement: Supplementary file 9 — Source data Fig. 8 [file 44318_2024_192_MOESM9_ESM.zip › Figure8/Figure8e/IP-LaminB1 LaminB1.tif]

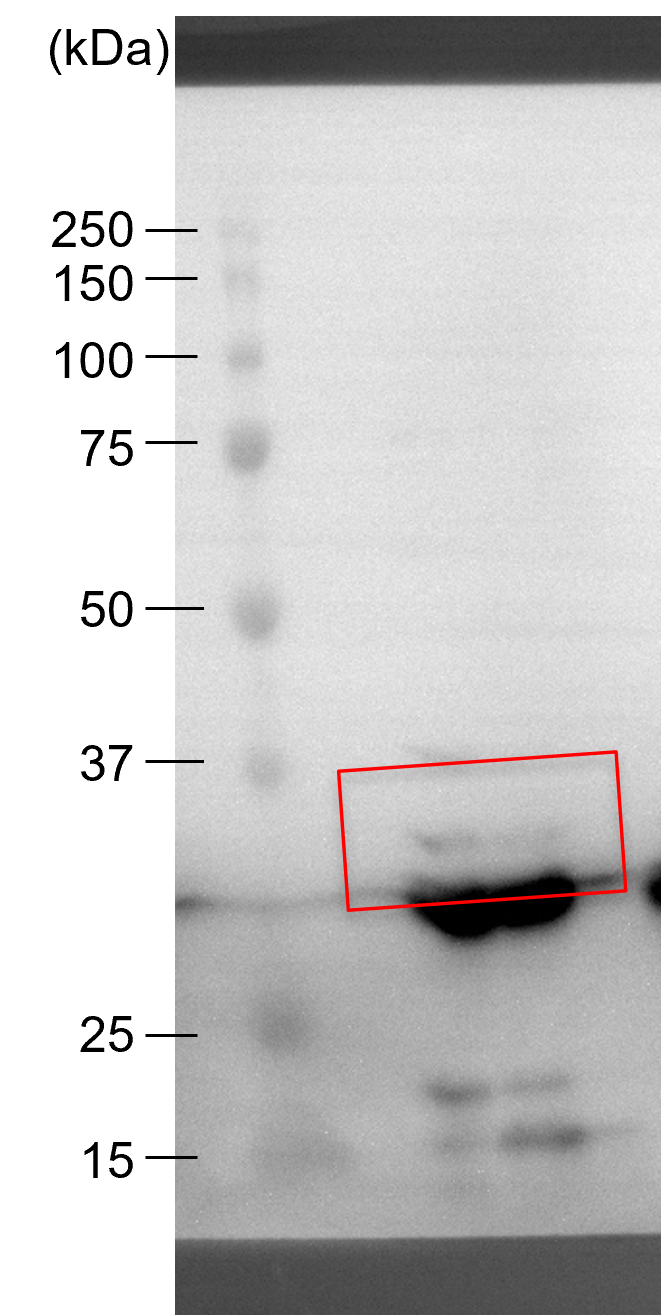

Supplement: Supplementary file 9 — Source data Fig. 8 [file 44318_2024_192_MOESM9_ESM.zip › Figure8/Figure8e/IP-LaminB1 PSME3.tif]

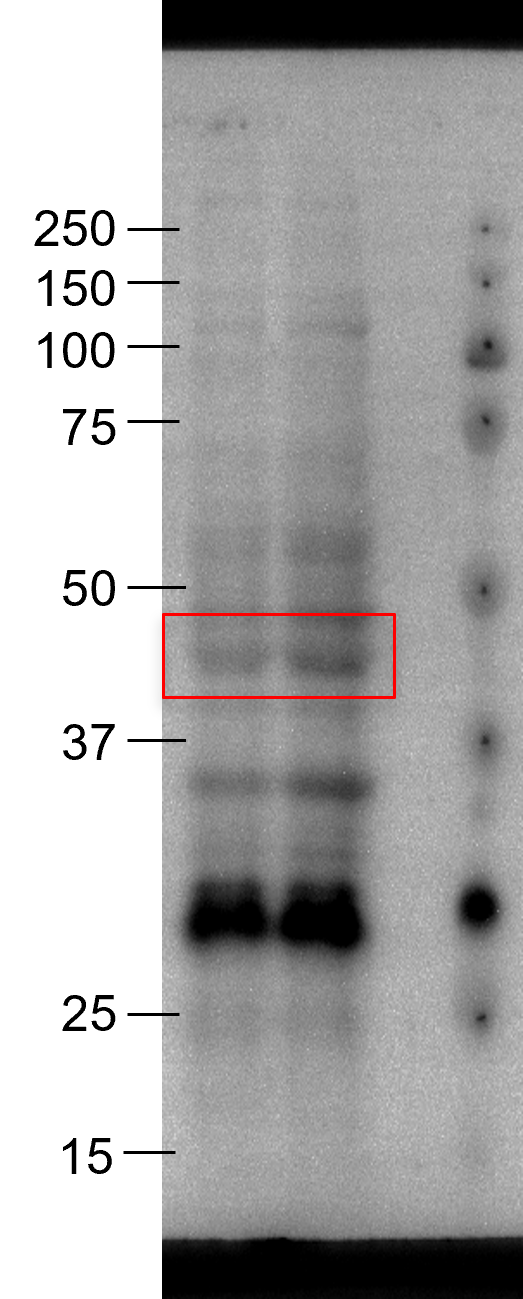

Supplement: Supplementary file 9 — Source data Fig. 8 [file 44318_2024_192_MOESM9_ESM.zip › Figure8/Figure8e/IP-LaminB1 PQBP3.tif]

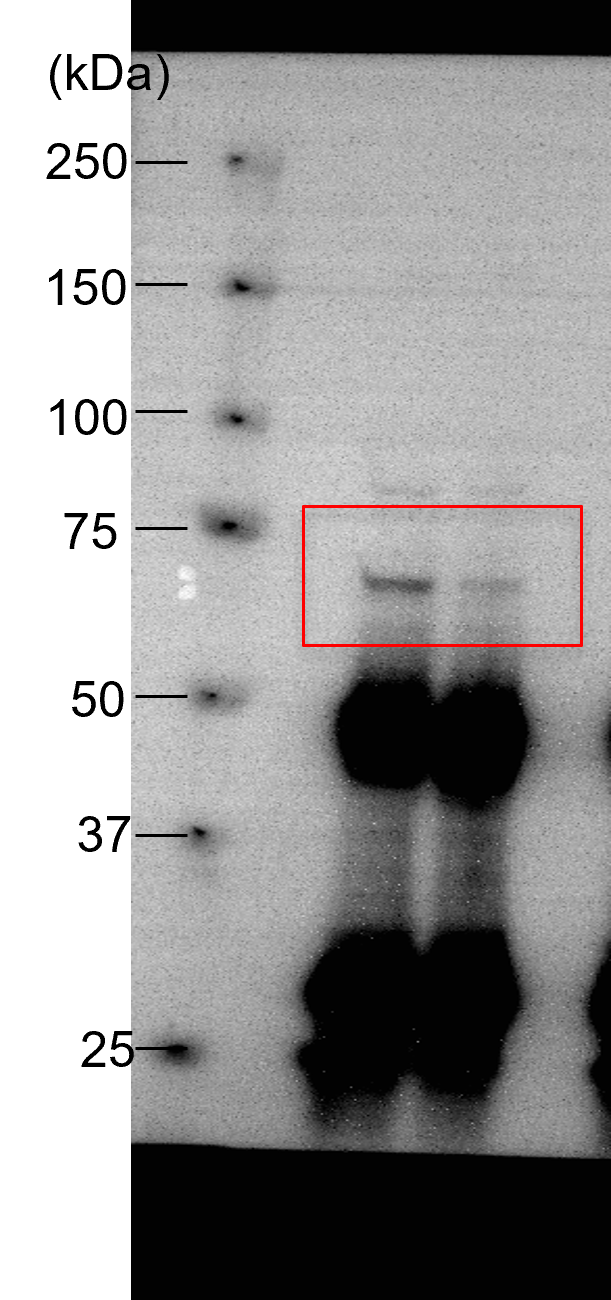

Supplement: Supplementary file 9 — Source data Fig. 8 [file 44318_2024_192_MOESM9_ESM.zip › Figure8/Figure8e/IP-PQBP3 LaminB1.tif]

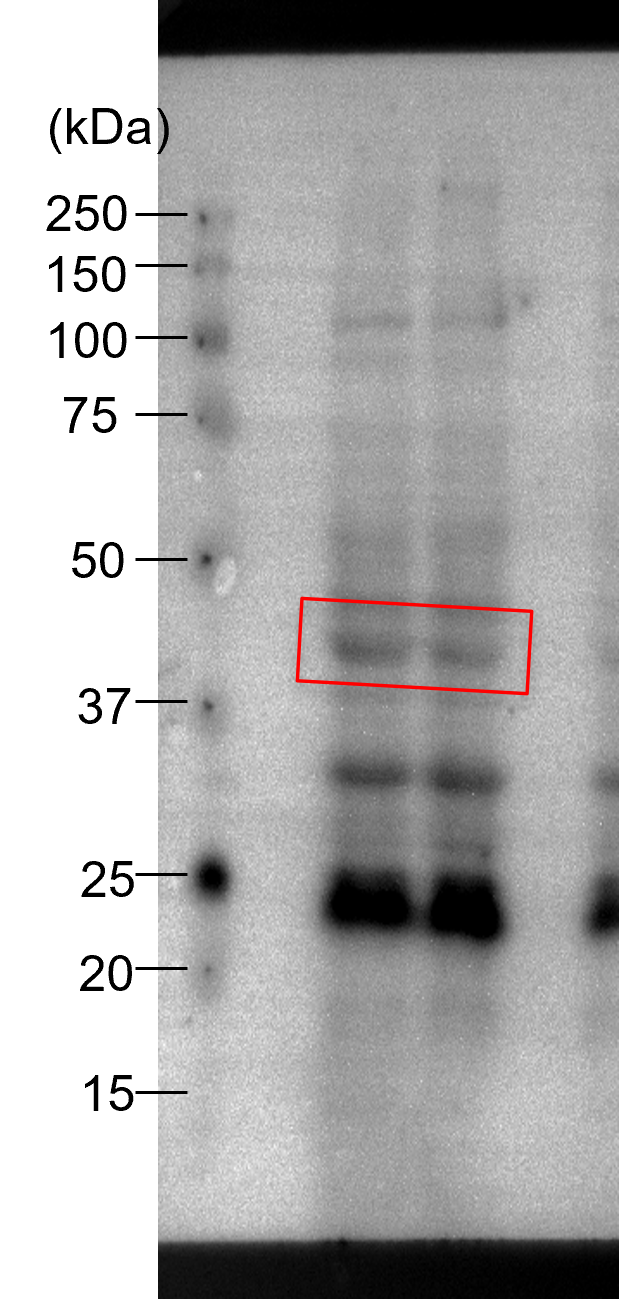

Supplement: Supplementary file 9 — Source data Fig. 8 [file 44318_2024_192_MOESM9_ESM.zip › Figure8/Figure8e/IP-PQBP3 PQBP3.tif]

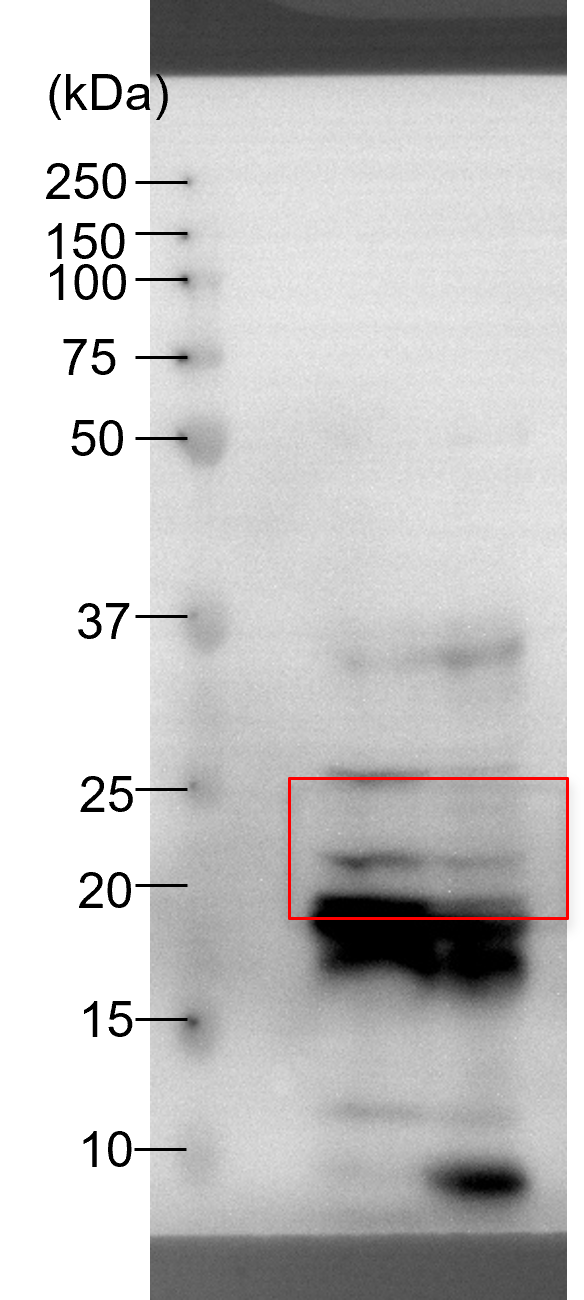

Supplement: Supplementary file 9 — Source data Fig. 8 [file 44318_2024_192_MOESM9_ESM.zip › Figure8/Figure8e/IP-PQBP3 PSME3.tif]

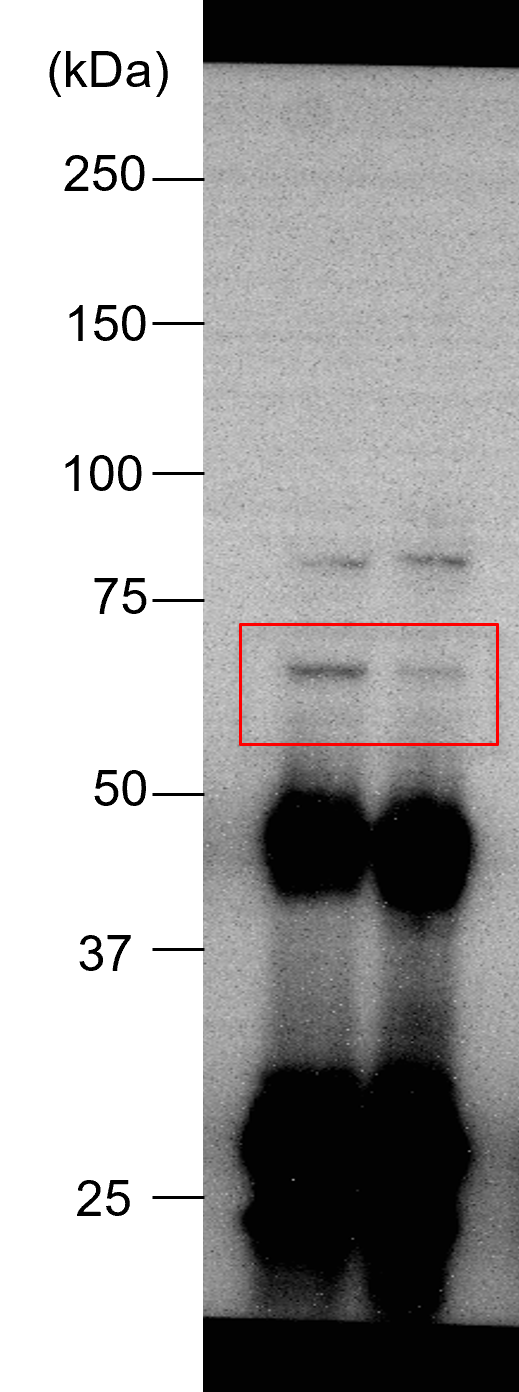

Supplement: Supplementary file 9 — Source data Fig. 8 [file 44318_2024_192_MOESM9_ESM.zip › Figure8/Figure8e/IP-PSME3 LaminB1.tif]

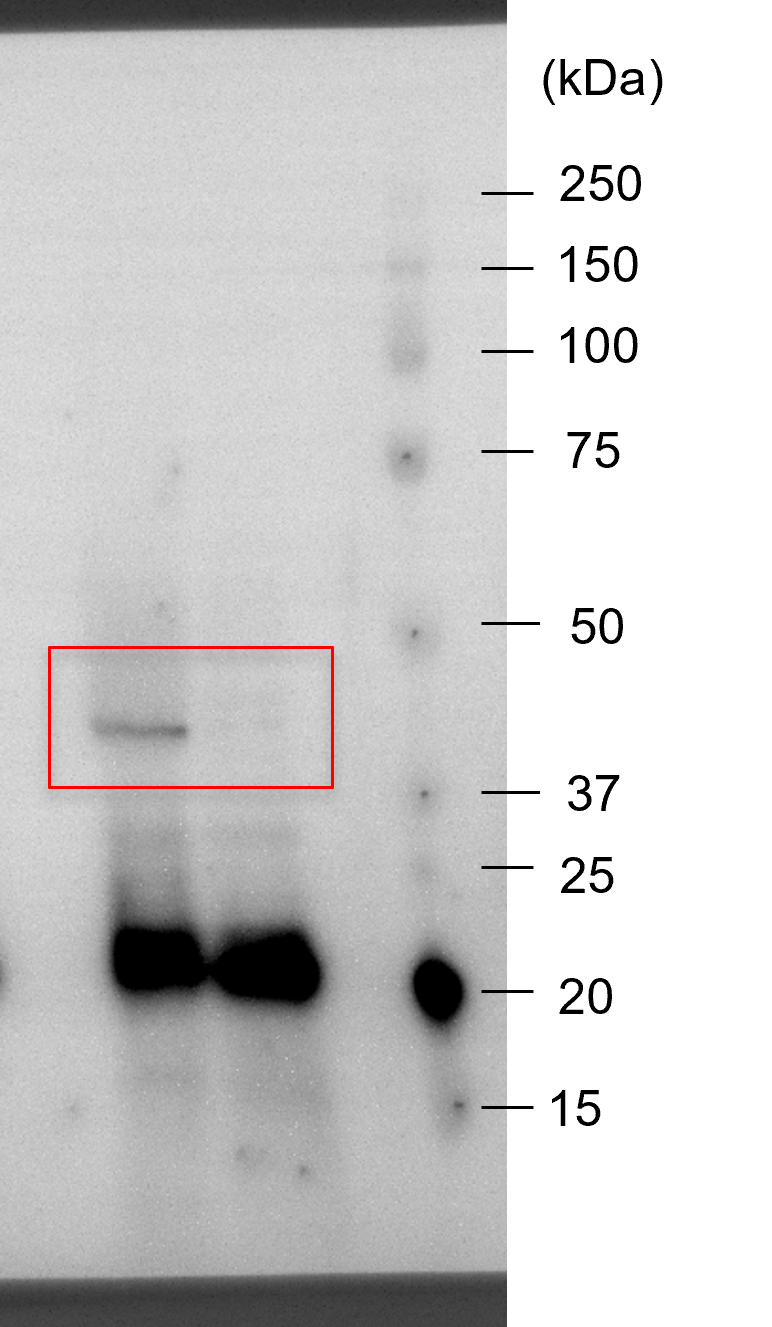

Supplement: Supplementary file 9 — Source data Fig. 8 [file 44318_2024_192_MOESM9_ESM.zip › Figure8/Figure8e/IP-PSME3 PQBP3.tif]

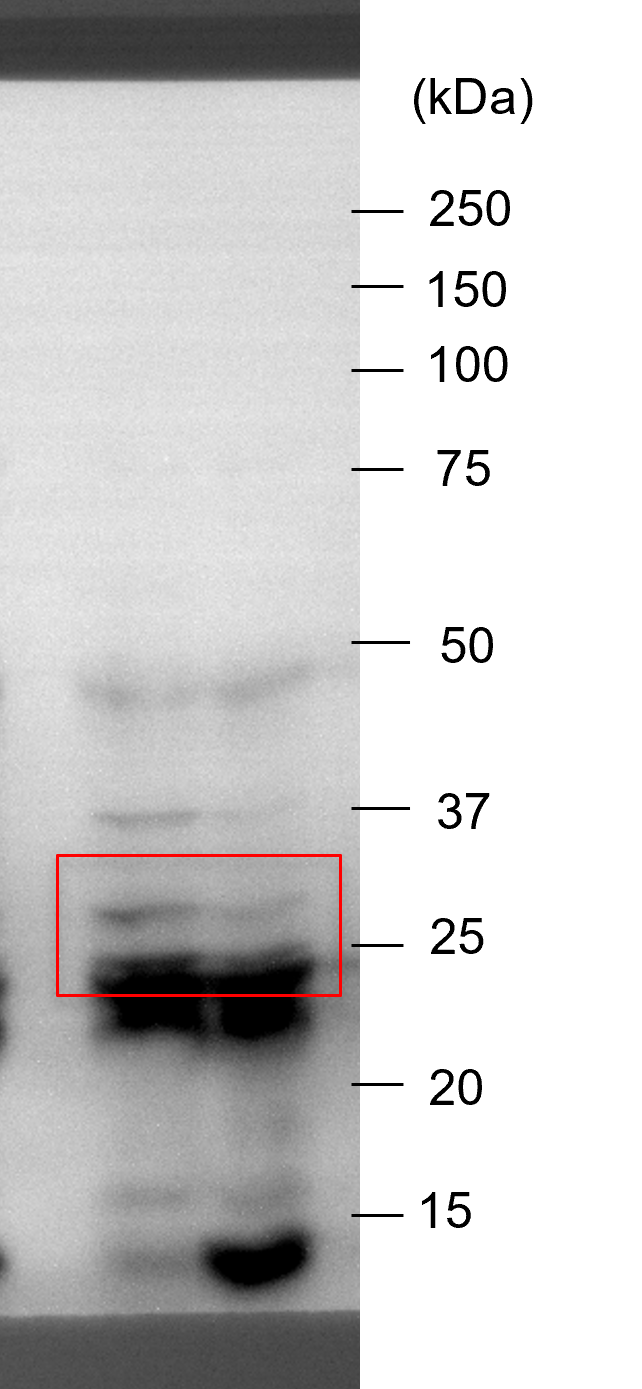

Supplement: Supplementary file 9 — Source data Fig. 8 [file 44318_2024_192_MOESM9_ESM.zip › Figure8/Figure8e/IP-PSME3 PSME3.tif]

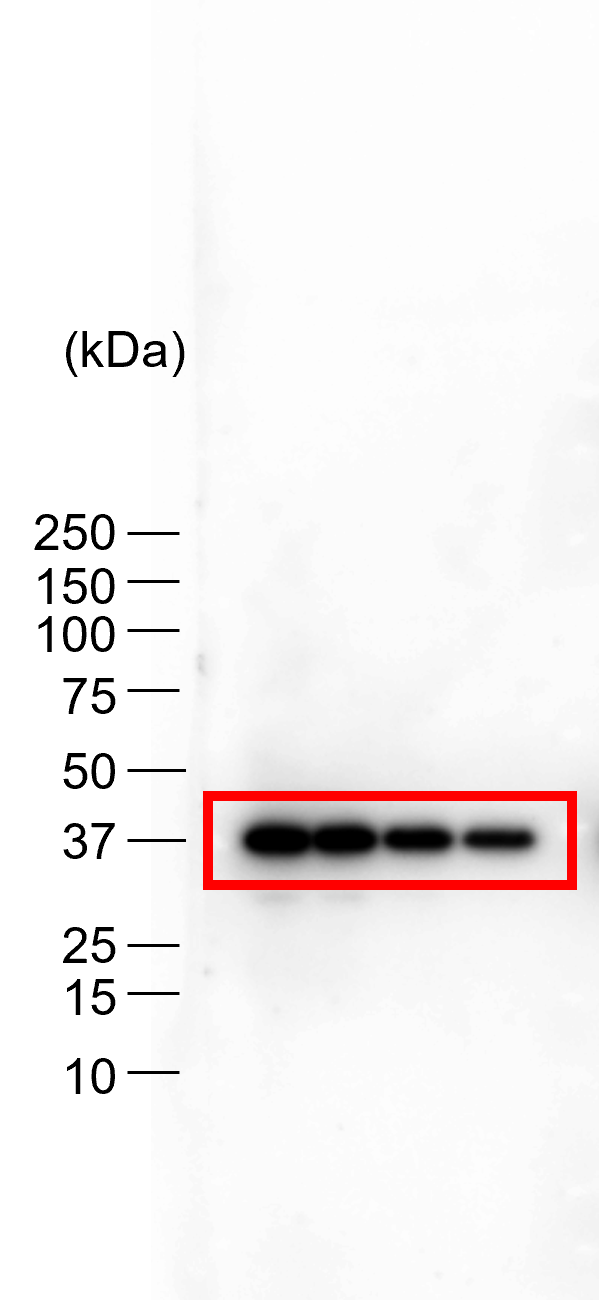

Supplement: Supplementary file 10 — Source data Fig. 9 [file 44318_2024_192_MOESM10_ESM.zip › Figure9/Figure9a/GAPDH.tif]

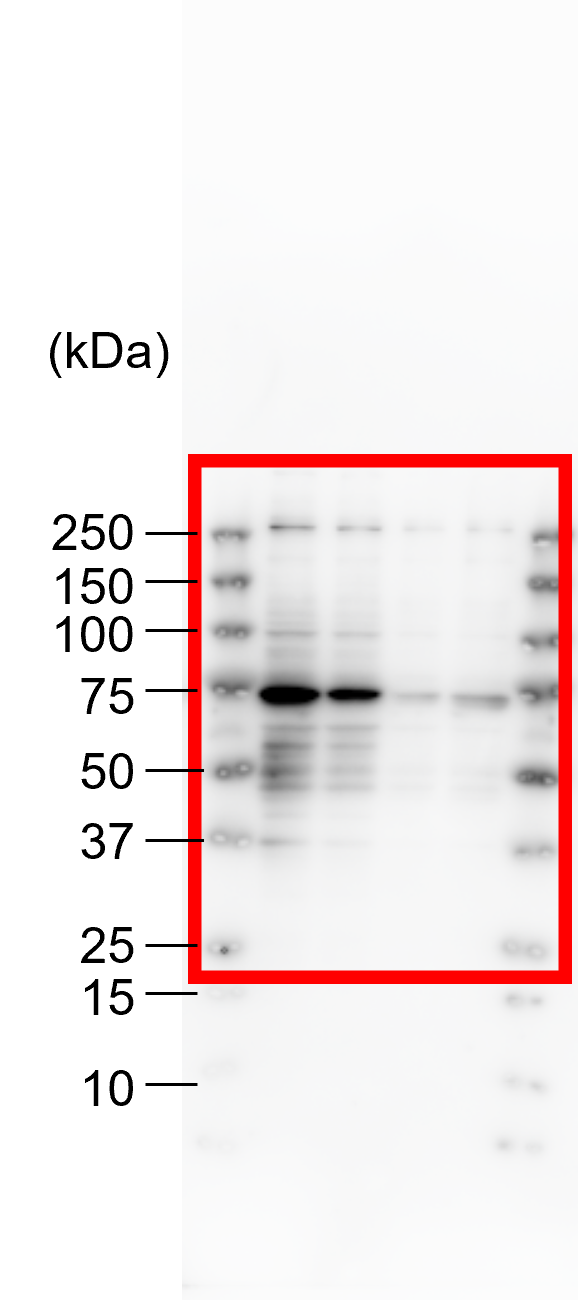

Supplement: Supplementary file 10 — Source data Fig. 9 [file 44318_2024_192_MOESM10_ESM.zip › Figure9/Figure9a/LaminB1.tif]

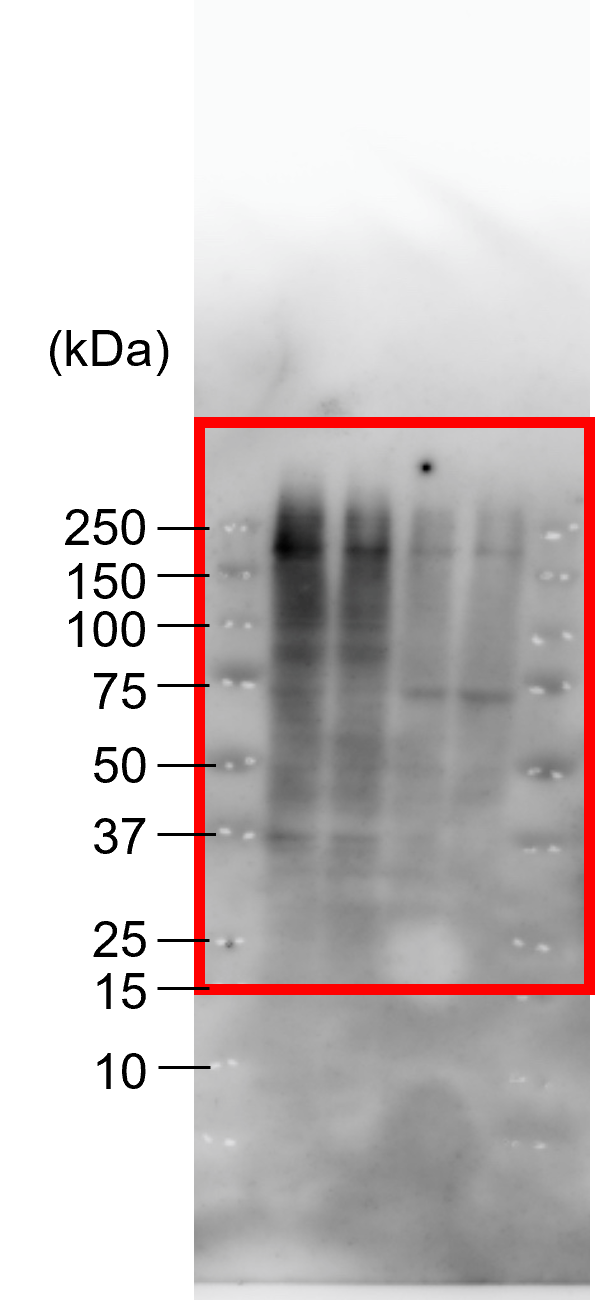

Supplement: Supplementary file 10 — Source data Fig. 9 [file 44318_2024_192_MOESM10_ESM.zip › Figure9/Figure9a/Ub.tif]

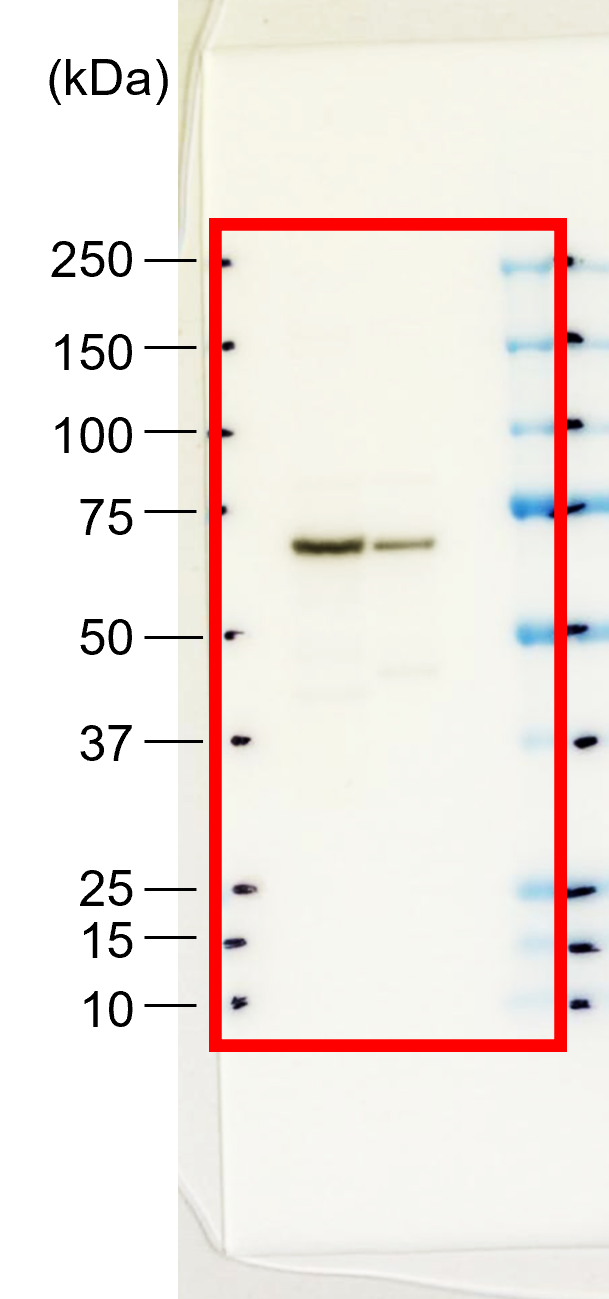

Supplement: Supplementary file 10 — Source data Fig. 9 [file 44318_2024_192_MOESM10_ESM.zip › Figure9/Figure9b/Input LaminB1.tif]

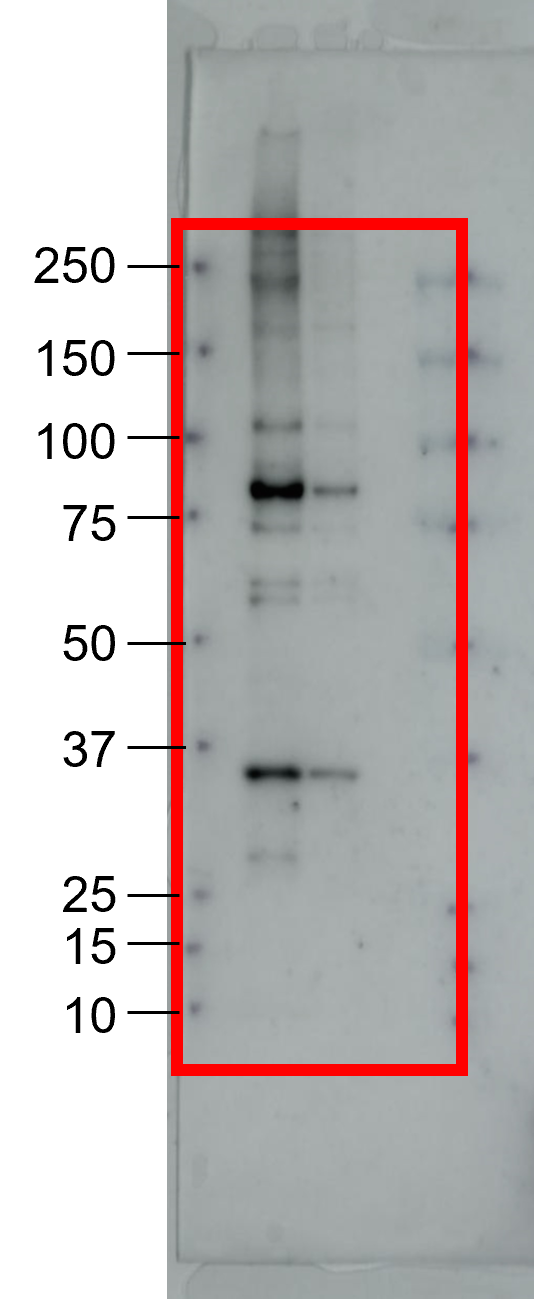

Supplement: Supplementary file 10 — Source data Fig. 9 [file 44318_2024_192_MOESM10_ESM.zip › Figure9/Figure9b/Input SUMO1.tif]

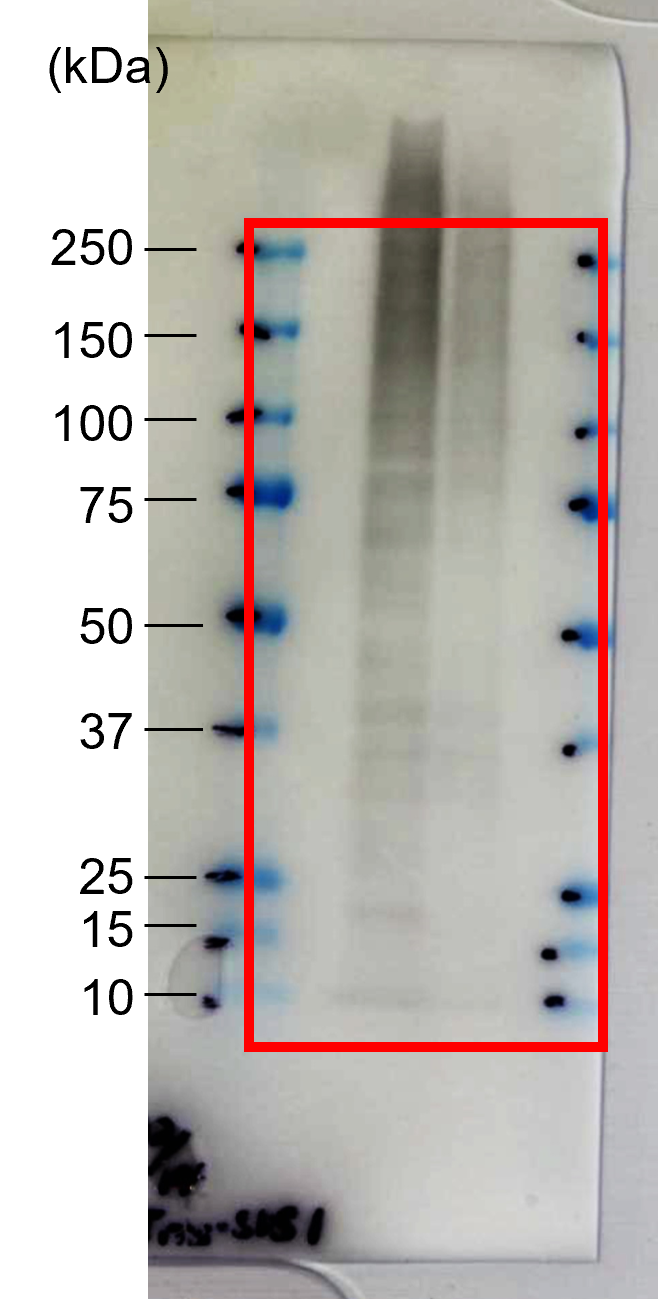

Supplement: Supplementary file 10 — Source data Fig. 9 [file 44318_2024_192_MOESM10_ESM.zip › Figure9/Figure9b/Input Ubiqutin.tif]

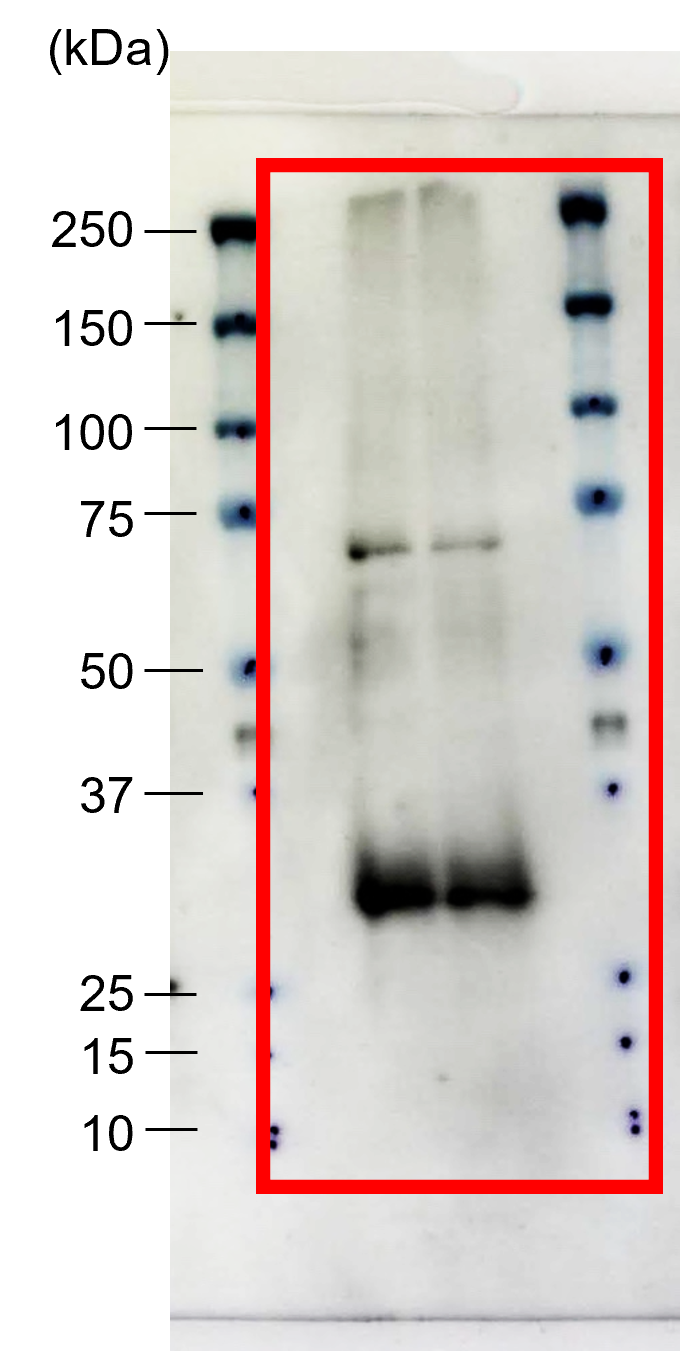

Supplement: Supplementary file 10 — Source data Fig. 9 [file 44318_2024_192_MOESM10_ESM.zip › Figure9/Figure9b/IP-LaminB1 LaminB1.tif]

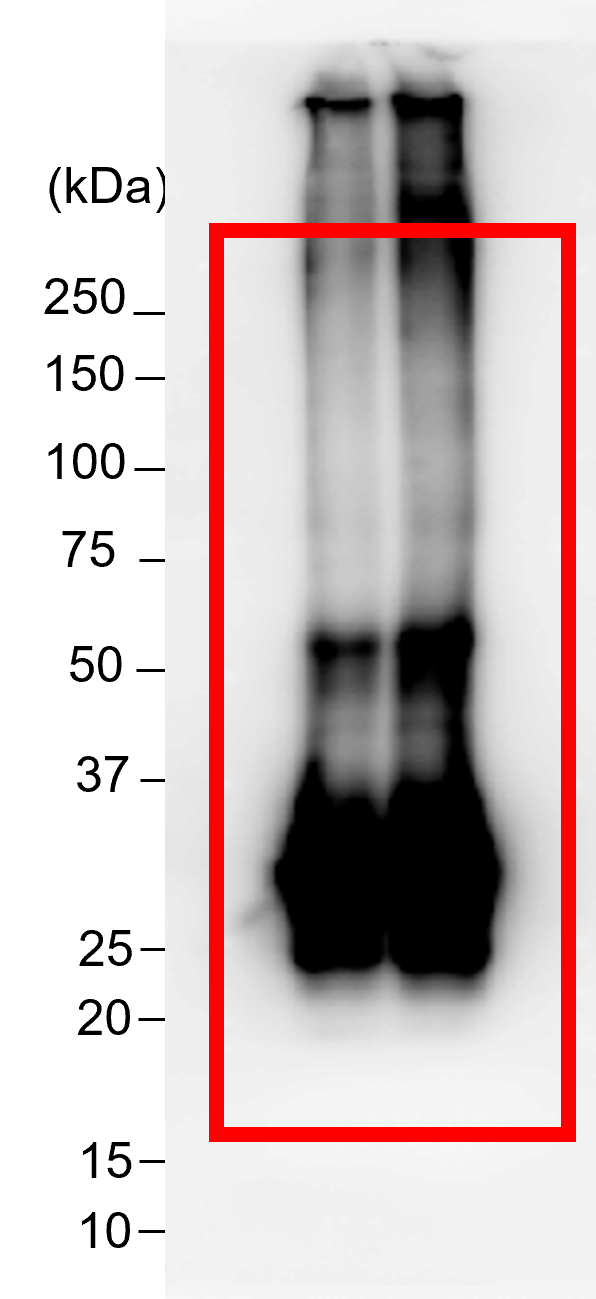

Supplement: Supplementary file 10 — Source data Fig. 9 [file 44318_2024_192_MOESM10_ESM.zip › Figure9/Figure9b/IP-LaminB1 SUMO1.tif]

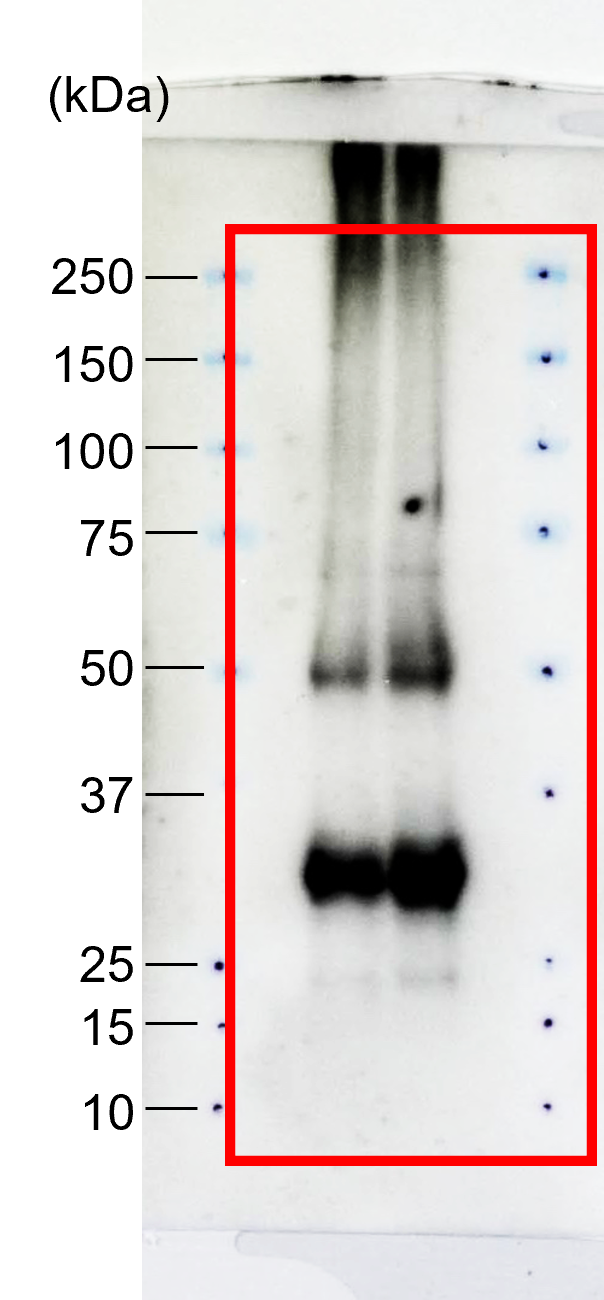

Supplement: Supplementary file 10 — Source data Fig. 9 [file 44318_2024_192_MOESM10_ESM.zip › Figure9/Figure9b/IP-LaminB1 Ubiquitin.tif]

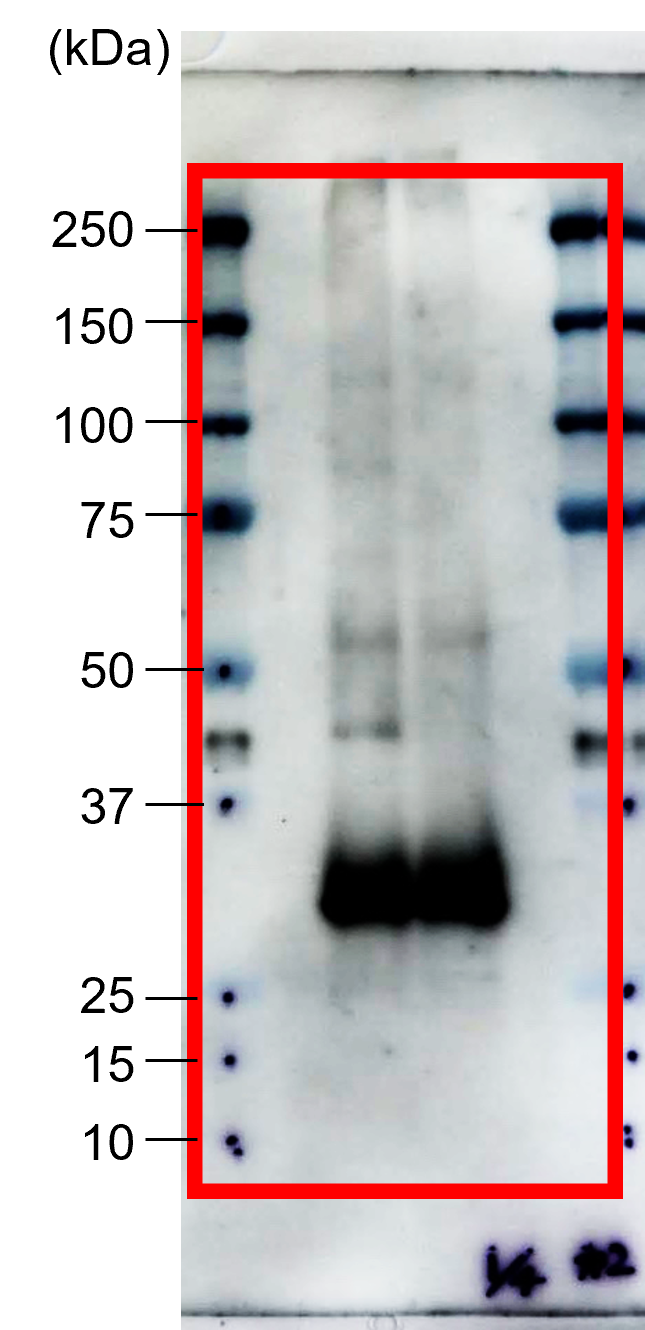

Supplement: Supplementary file 10 — Source data Fig. 9 [file 44318_2024_192_MOESM10_ESM.zip › Figure9/Figure9b/IP-Ub LaminB1.tif]

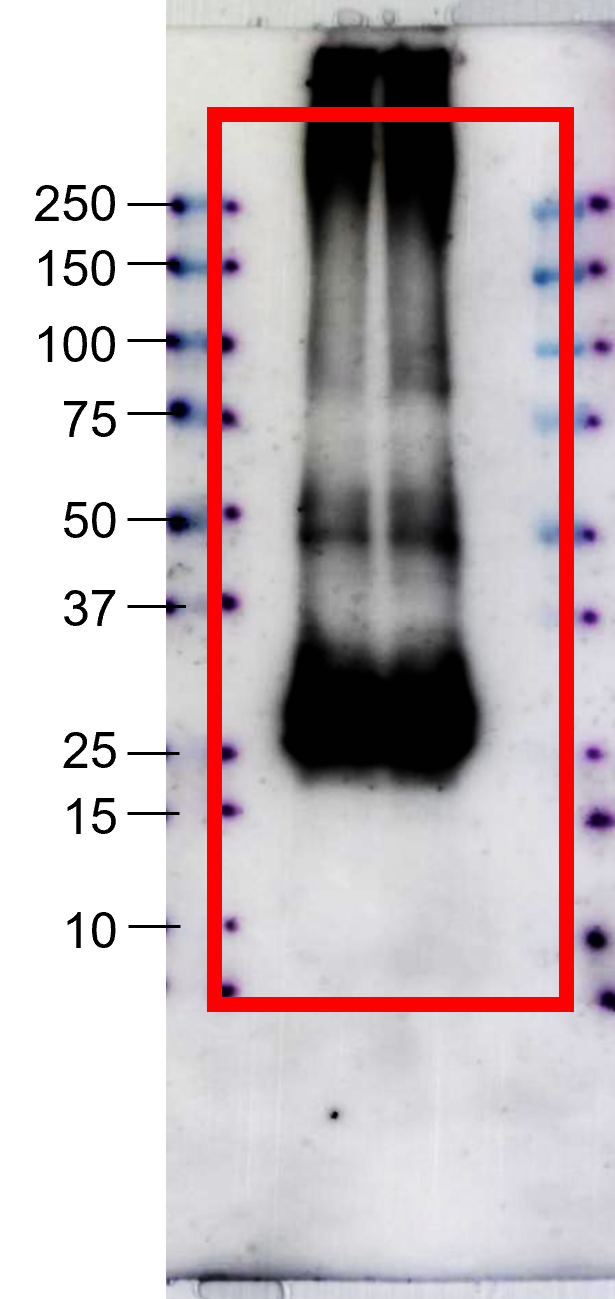

Supplement: Supplementary file 10 — Source data Fig. 9 [file 44318_2024_192_MOESM10_ESM.zip › Figure9/Figure9b/IP-Ub SUMO1.tif]

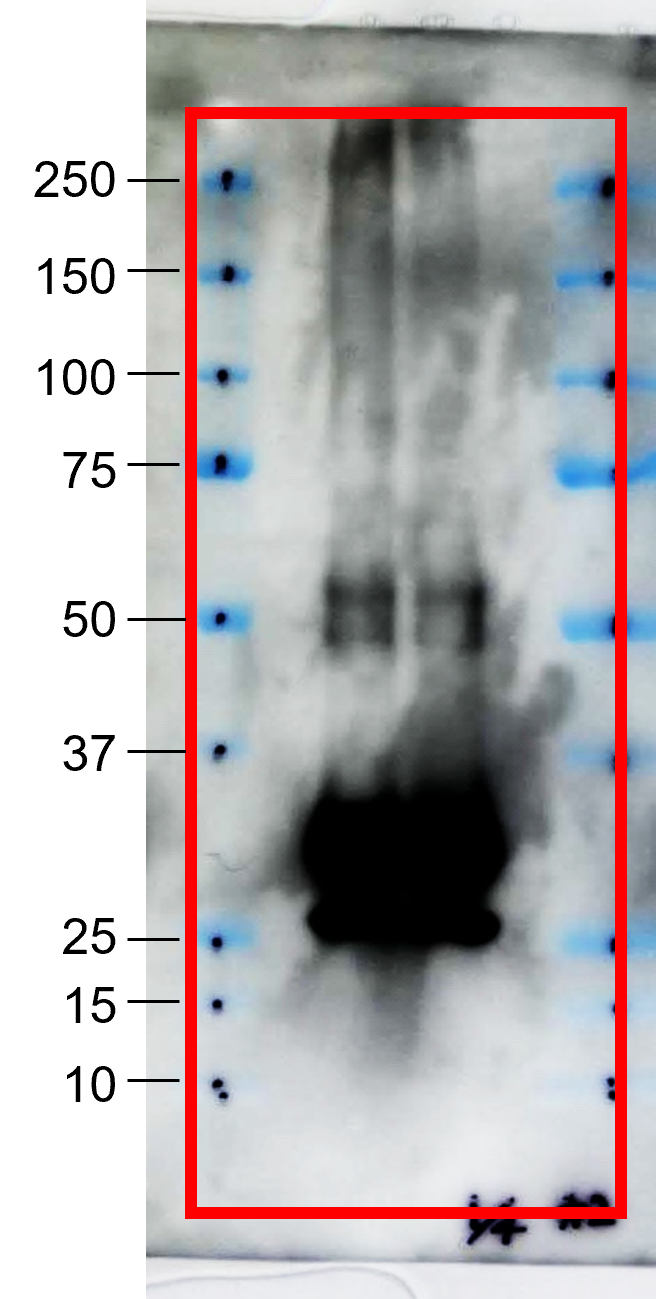

Supplement: Supplementary file 10 — Source data Fig. 9 [file 44318_2024_192_MOESM10_ESM.zip › Figure9/Figure9b/IP-Ub Ubiquitin.tif]
