# Supplementary figures and images for: PQBP3 prevents senescence by suppressing PSME3-mediated proteasomal Lamin B1 degradation (part 3 of 4)
Source: EMBO J. 2024 Aug 5;43(18):3968–99. doi: 10.1038/s44318-024-00192-4 (PMC11405525; doi:10.1038/s44318-024-00192-4)

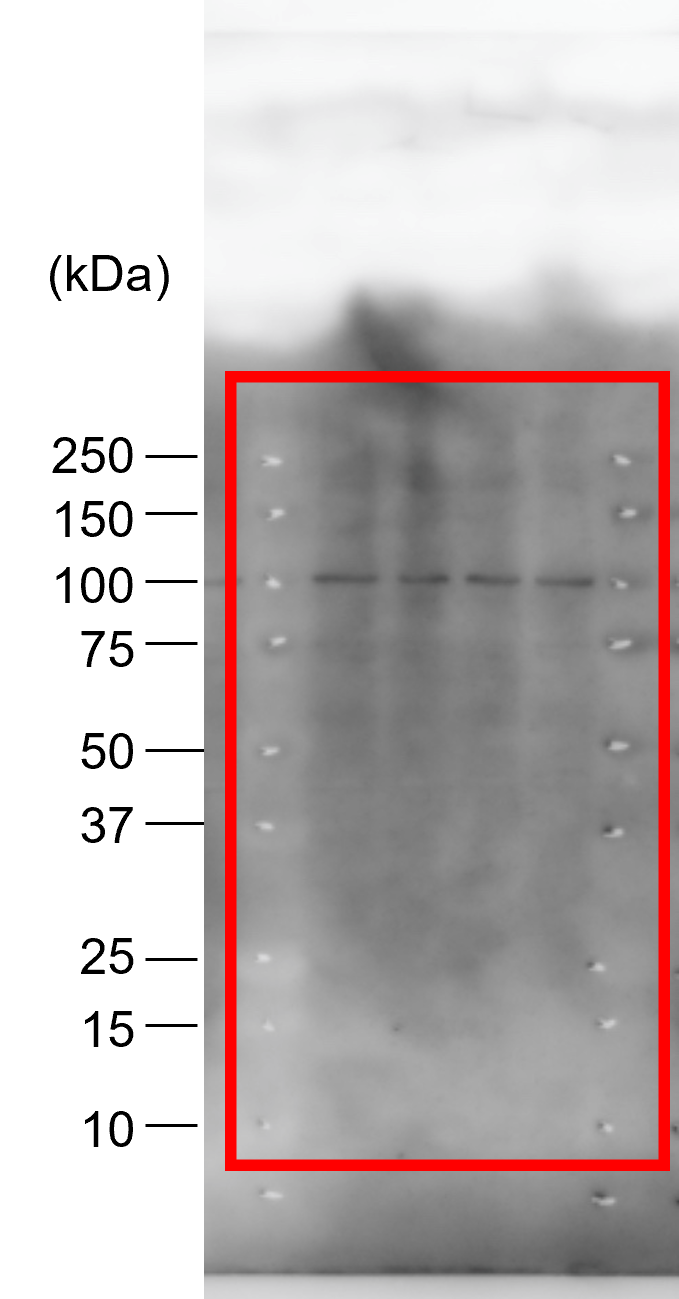

Supplement: Supplementary file 10 — Source data Fig. 9 [file 44318_2024_192_MOESM10_ESM.zip › Figure9/Figure9c/GST.tif]

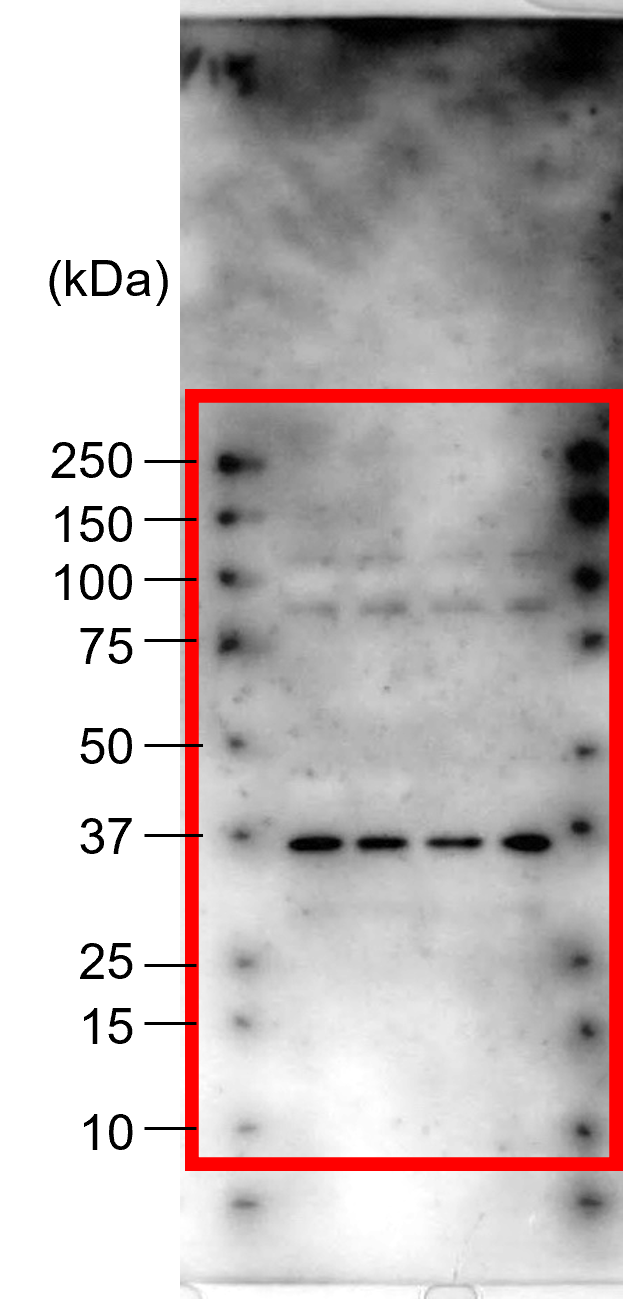

Supplement: Supplementary file 10 — Source data Fig. 9 [file 44318_2024_192_MOESM10_ESM.zip › Figure9/Figure9c/SUMO1.tif]

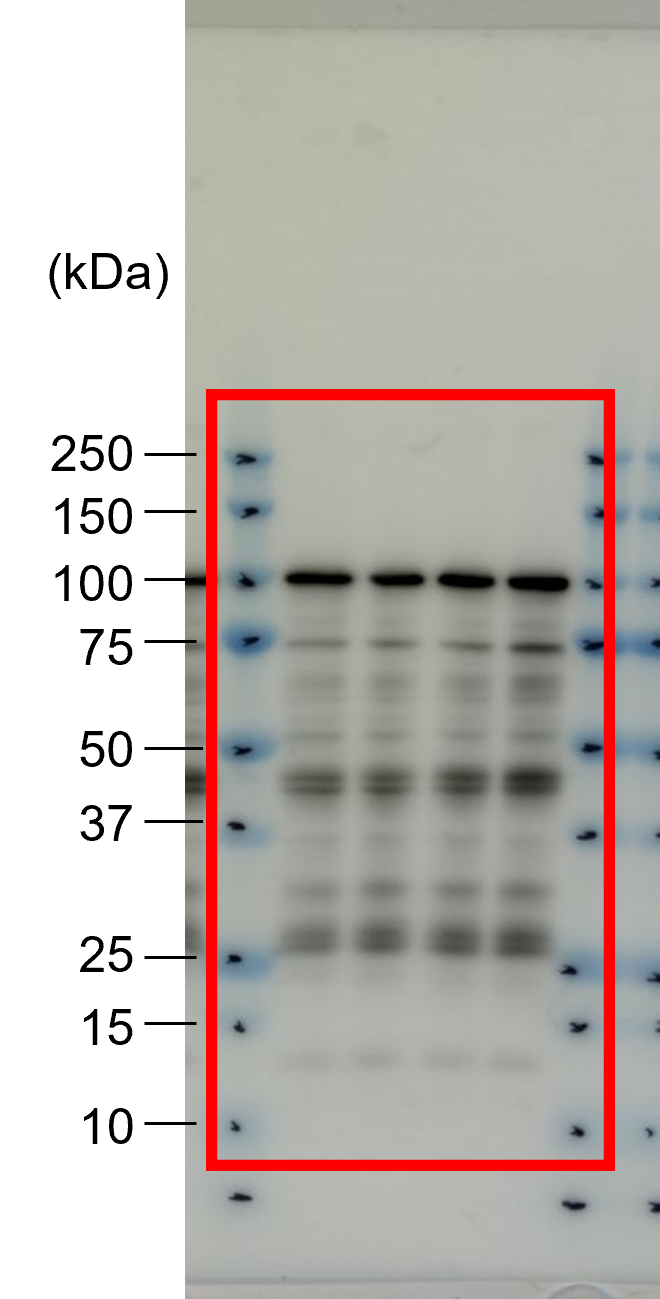

Supplement: Supplementary file 10 — Source data Fig. 9 [file 44318_2024_192_MOESM10_ESM.zip › Figure9/Figure9c/Ub.tif]

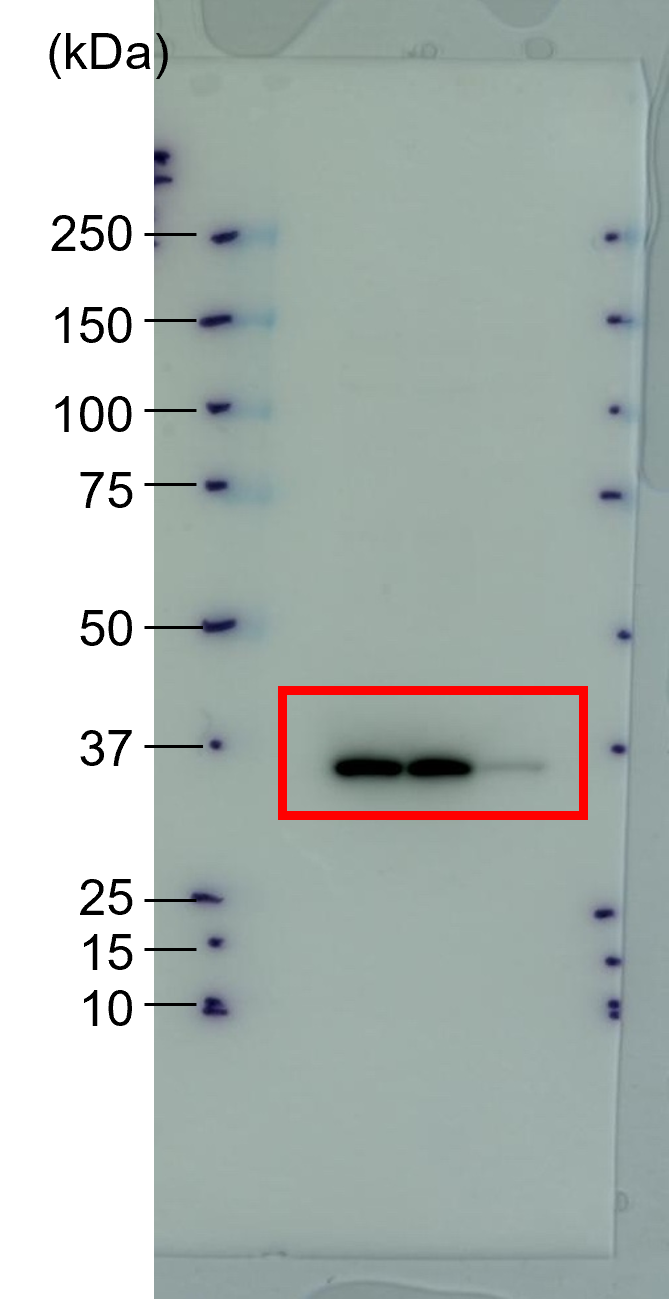

Supplement: Supplementary file 10 — Source data Fig. 9 [file 44318_2024_192_MOESM10_ESM.zip › Figure9/Figure9d/GAPDH.tif]

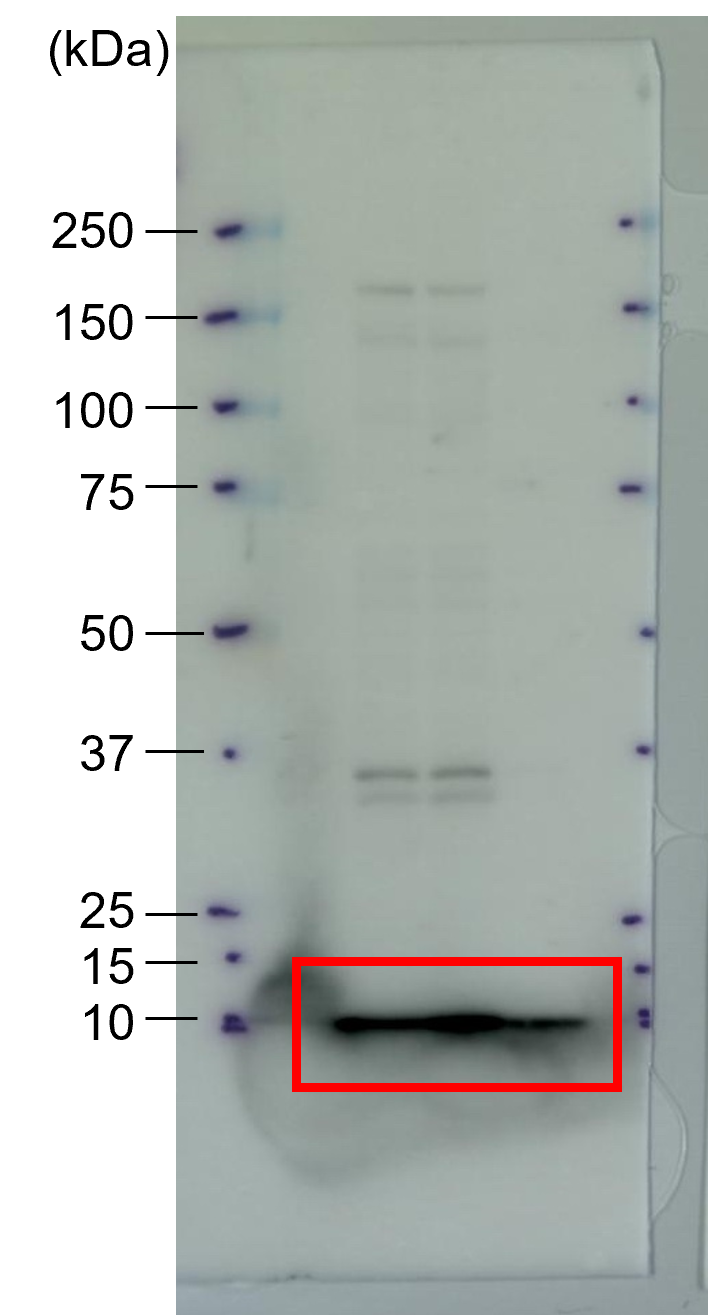

Supplement: Supplementary file 10 — Source data Fig. 9 [file 44318_2024_192_MOESM10_ESM.zip › Figure9/Figure9d/Histone.tif]

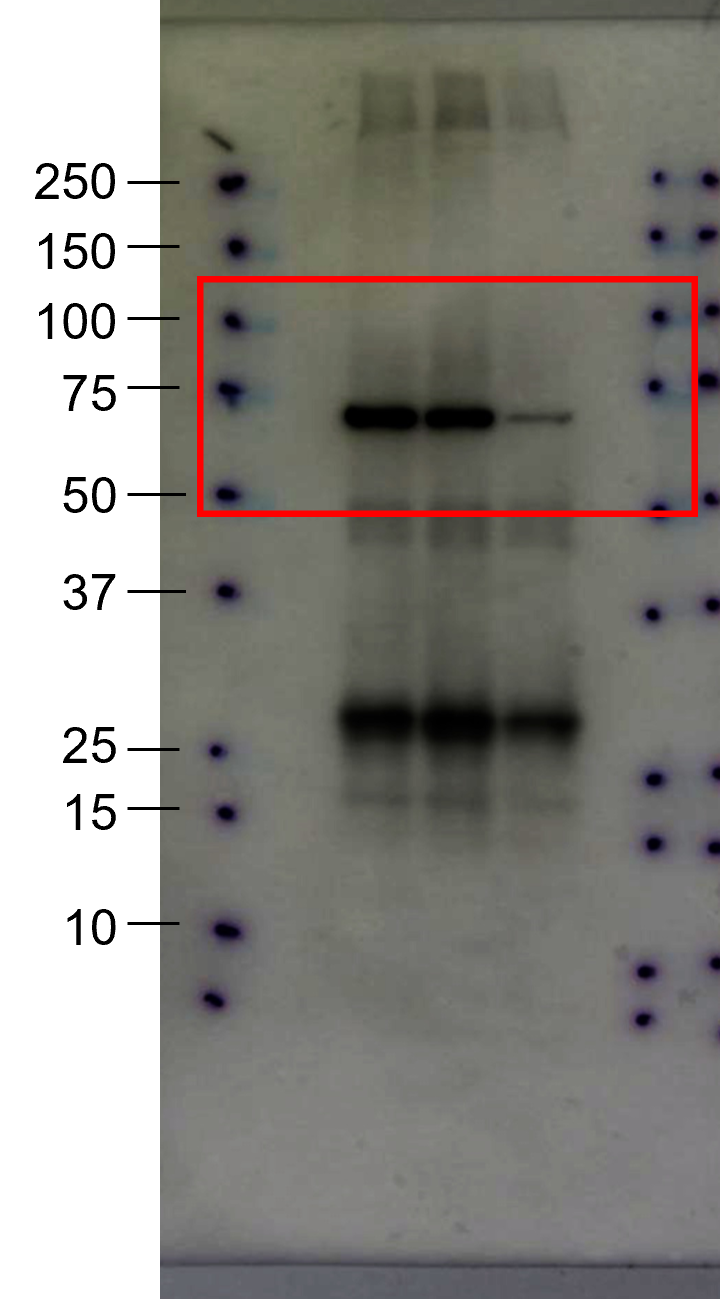

Supplement: Supplementary file 10 — Source data Fig. 9 [file 44318_2024_192_MOESM10_ESM.zip › Figure9/Figure9d/IP-LaminB1 GAPDH.tif]

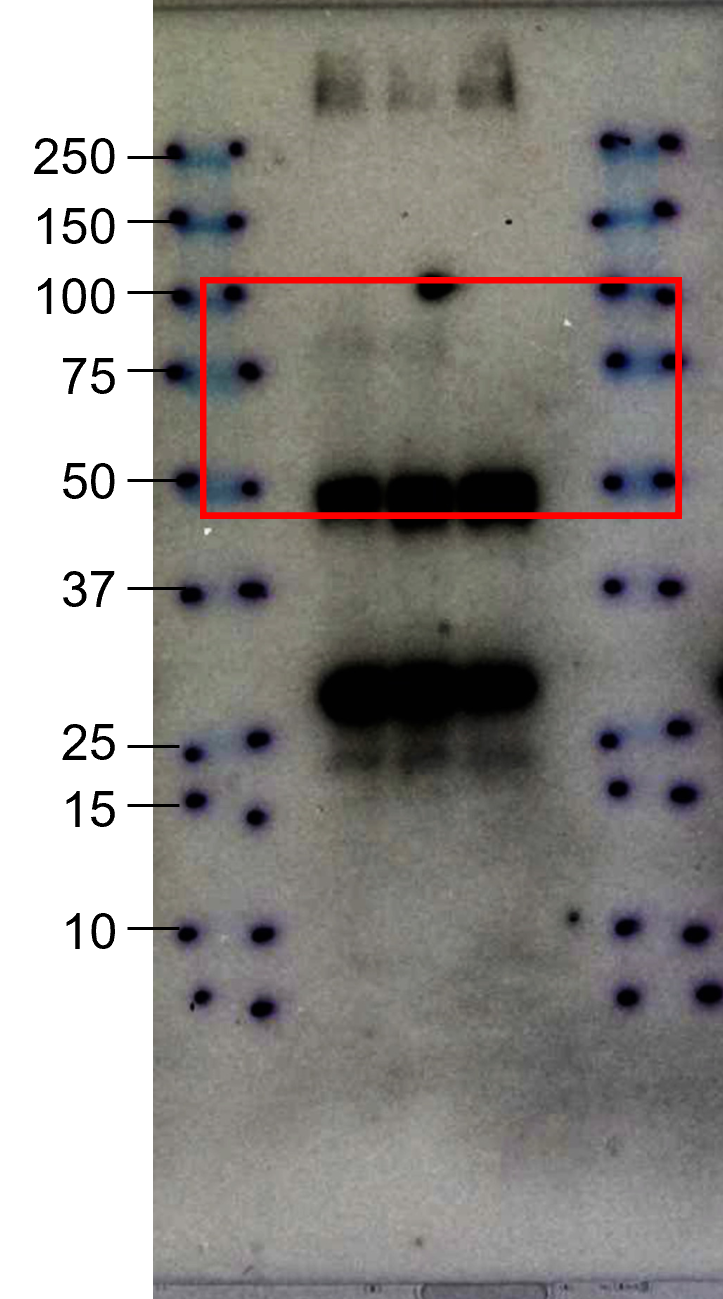

Supplement: Supplementary file 10 — Source data Fig. 9 [file 44318_2024_192_MOESM10_ESM.zip › Figure9/Figure9d/IP-LaminB1 Ub.tif]

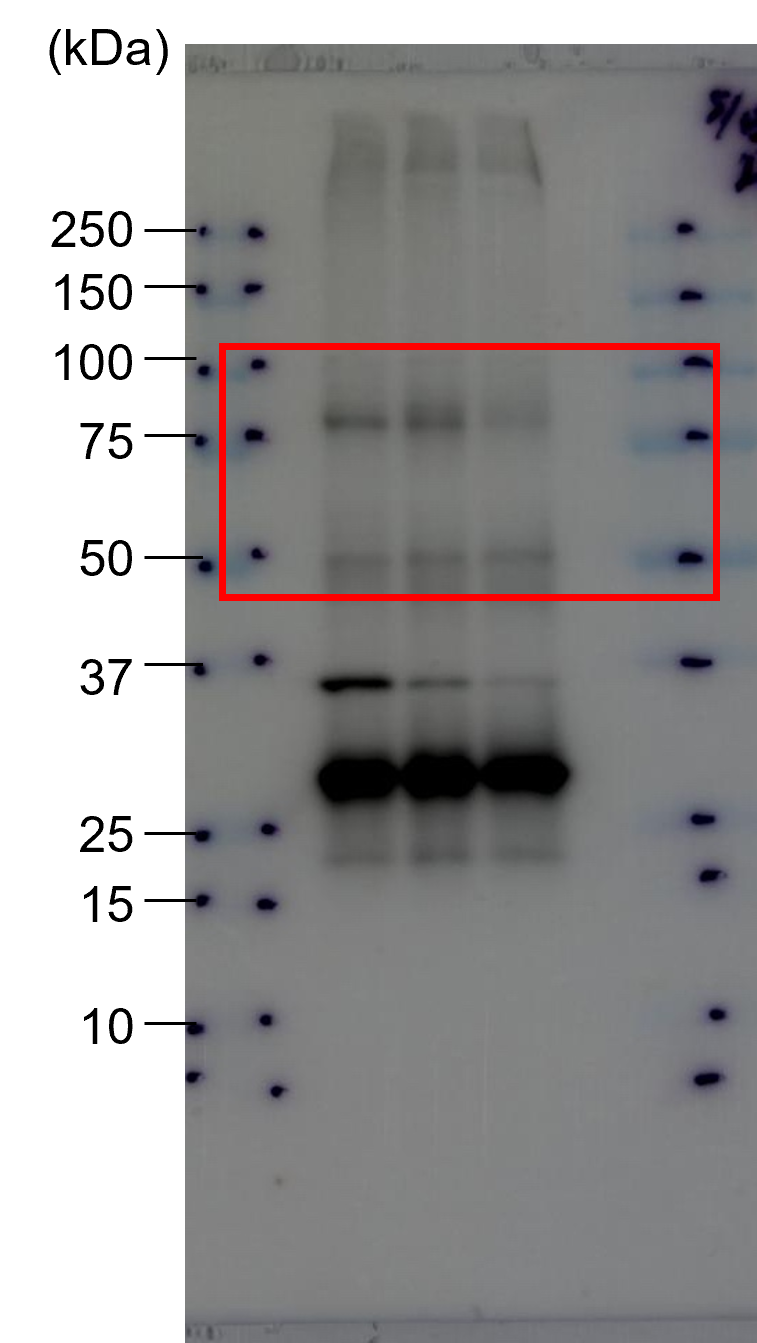

Supplement: Supplementary file 10 — Source data Fig. 9 [file 44318_2024_192_MOESM10_ESM.zip › Figure9/Figure9d/IP-LaminB1 SUMO1.tif]

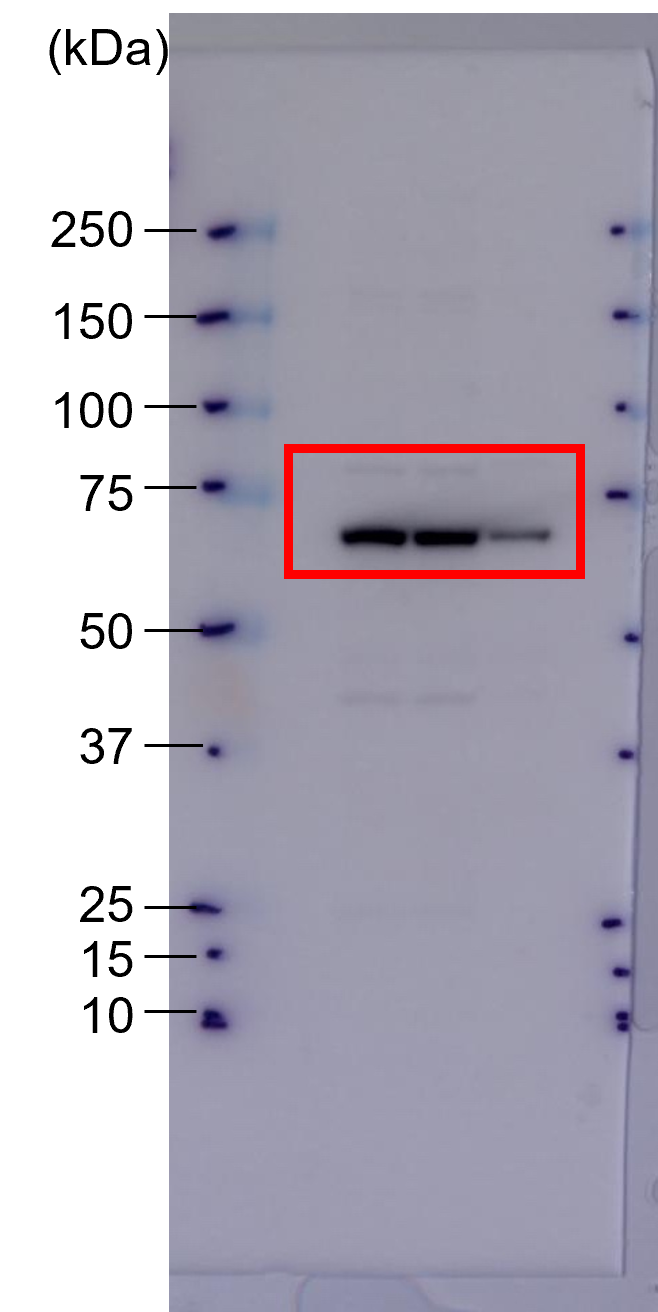

Supplement: Supplementary file 10 — Source data Fig. 9 [file 44318_2024_192_MOESM10_ESM.zip › Figure9/Figure9d/LaminB1.tif]

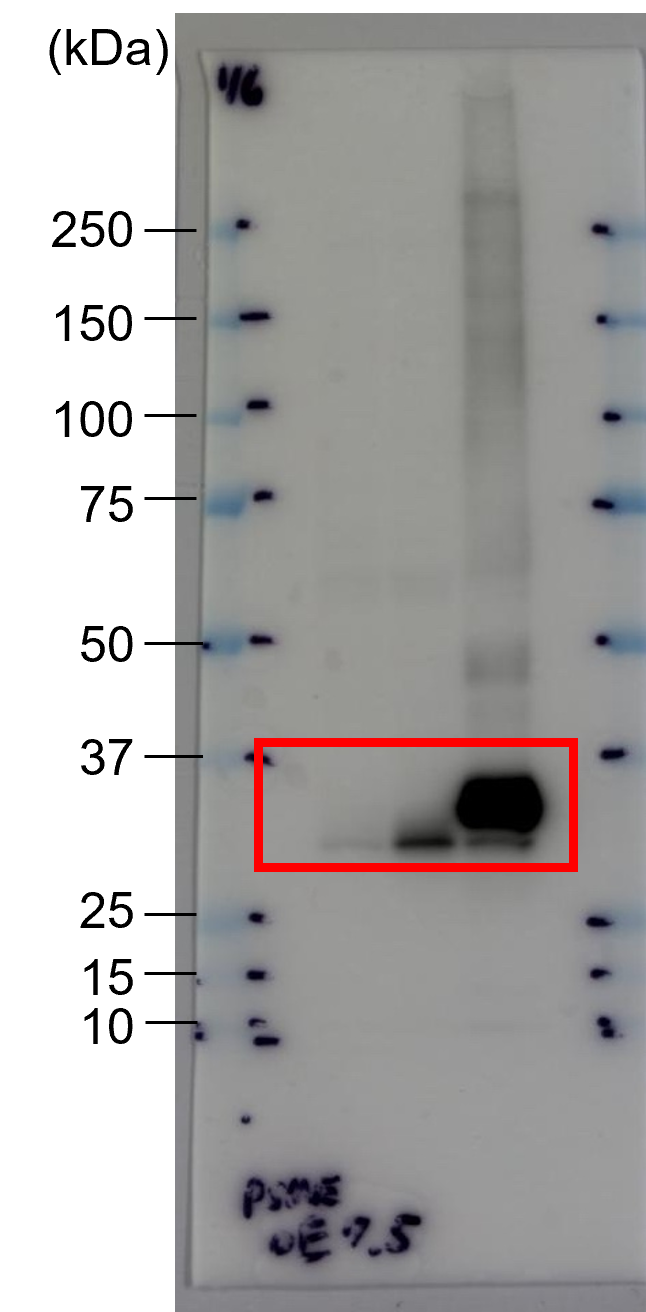

Supplement: Supplementary file 10 — Source data Fig. 9 [file 44318_2024_192_MOESM10_ESM.zip › Figure9/Figure9d/PSME3.tif]

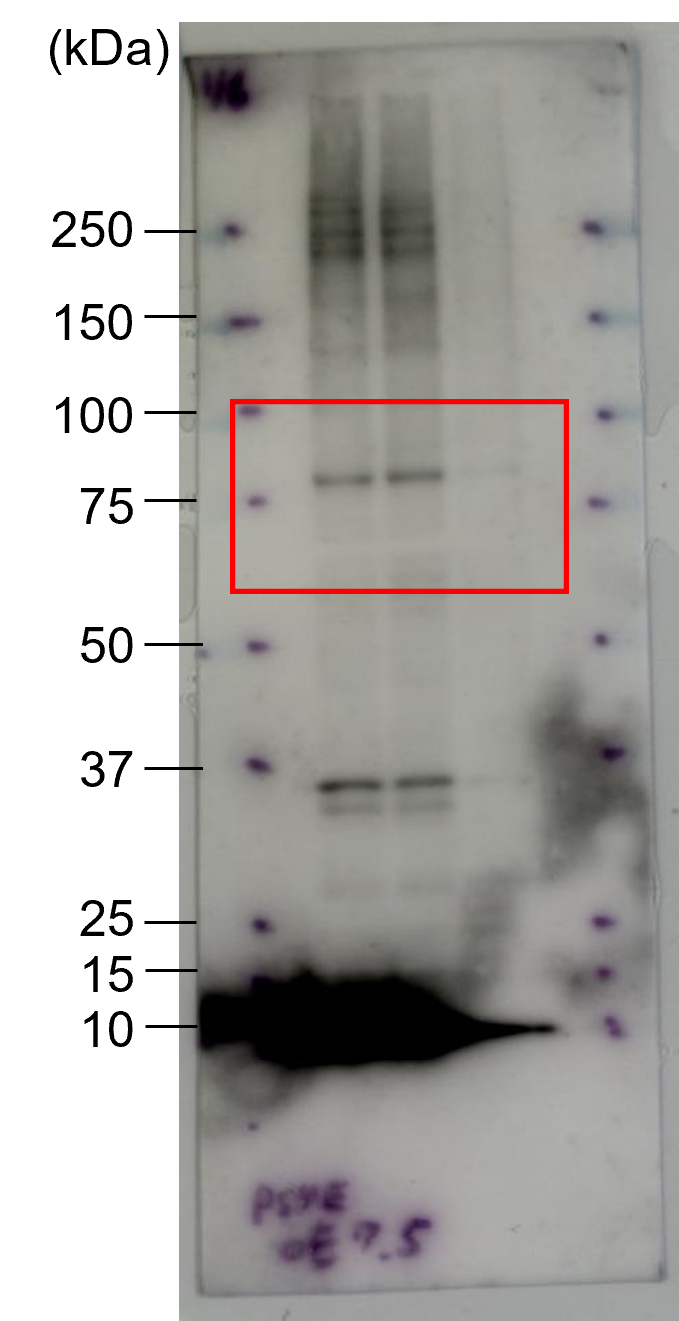

Supplement: Supplementary file 10 — Source data Fig. 9 [file 44318_2024_192_MOESM10_ESM.zip › Figure9/Figure9d/SUMO1.tif]

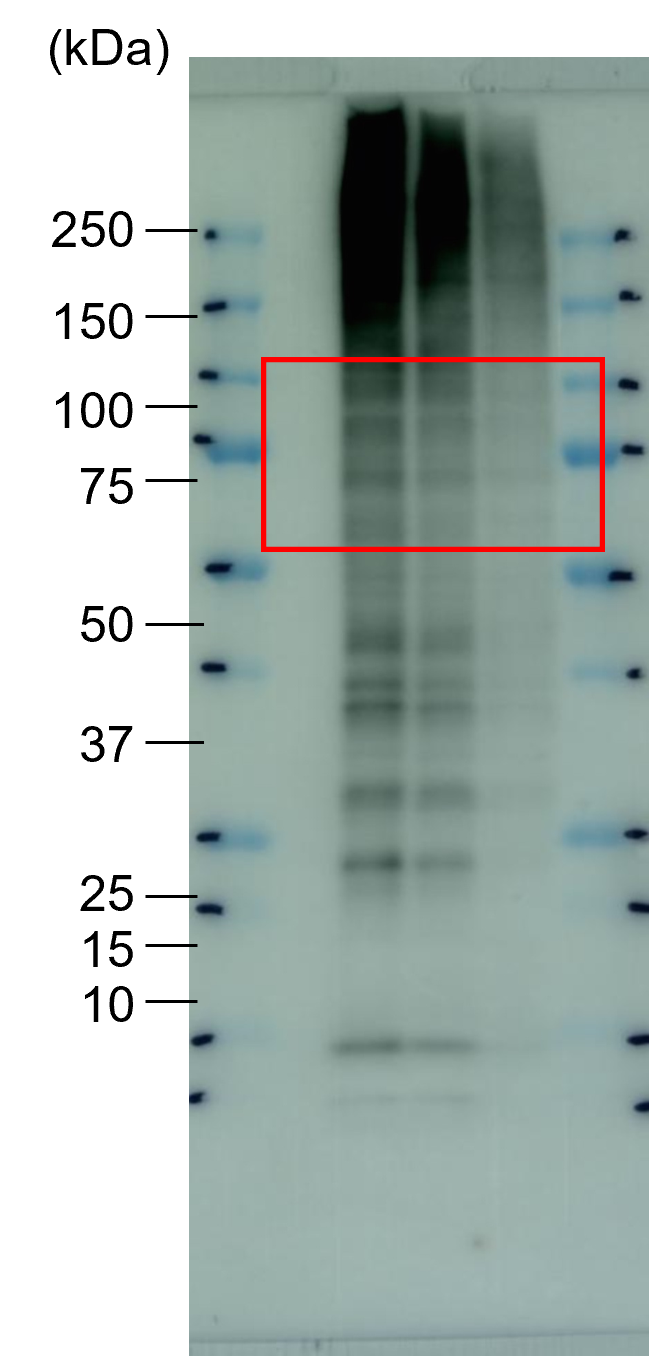

Supplement: Supplementary file 10 — Source data Fig. 9 [file 44318_2024_192_MOESM10_ESM.zip › Figure9/Figure9d/Ub.tif]

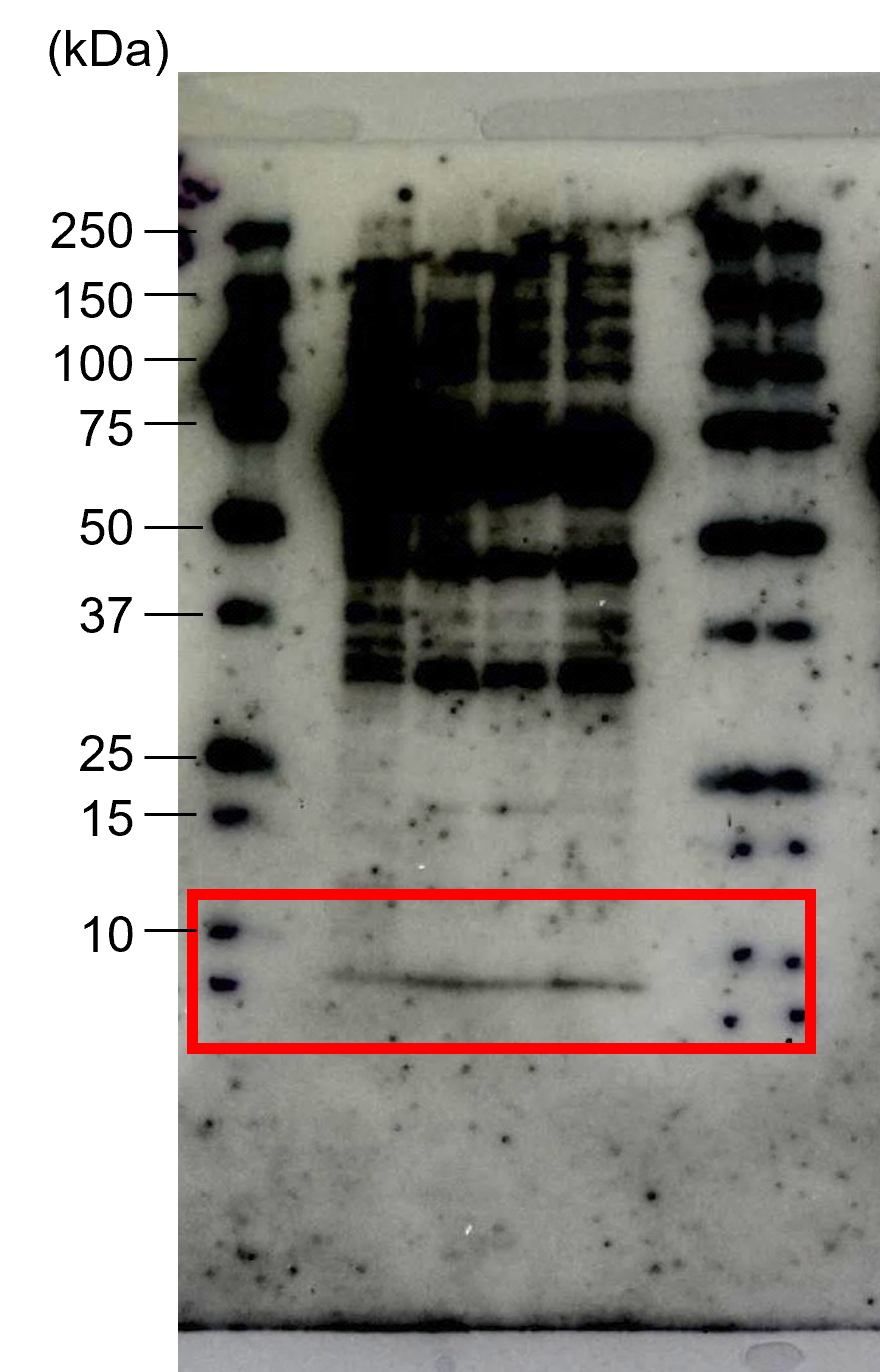

Supplement: Supplementary file 10 — Source data Fig. 9 [file 44318_2024_192_MOESM10_ESM.zip › Figure9/Figure9e/Histone.tif]

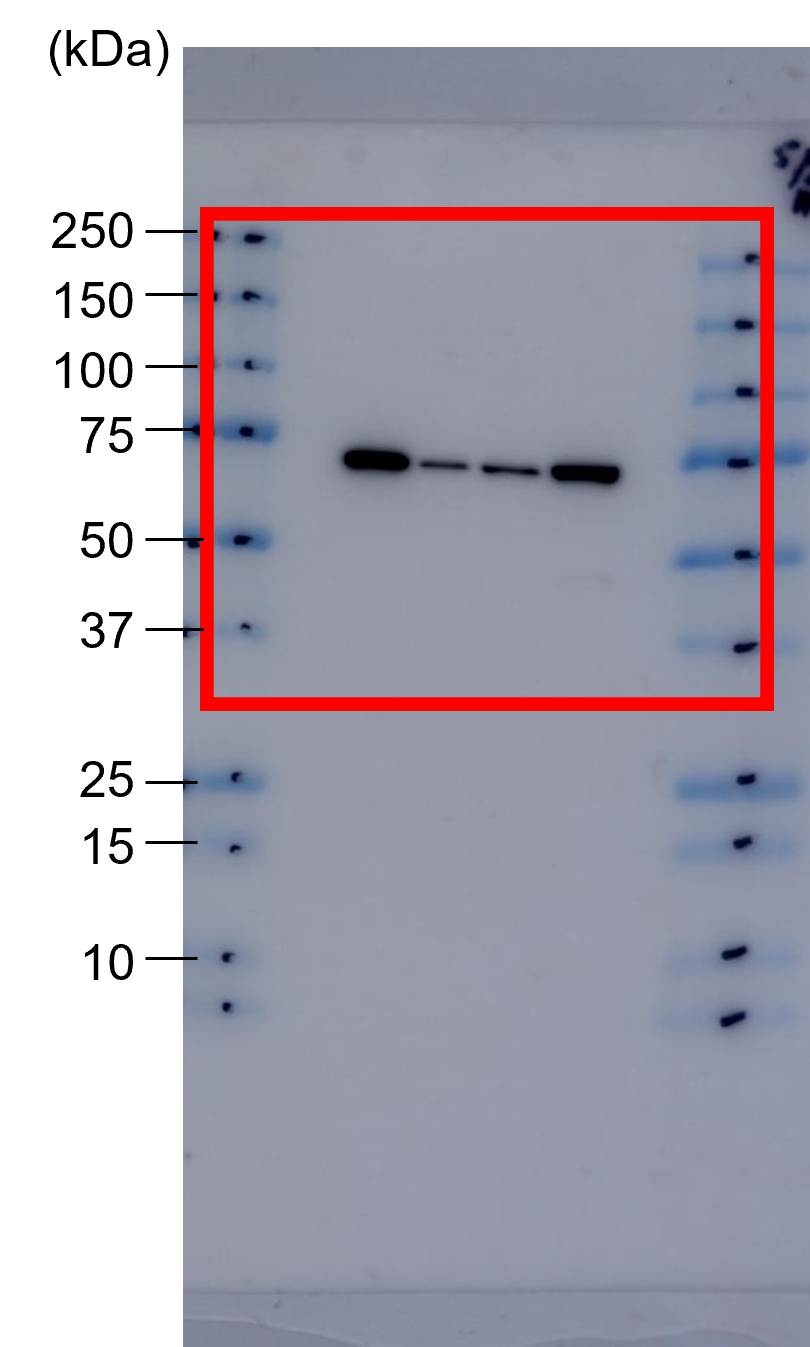

Supplement: Supplementary file 10 — Source data Fig. 9 [file 44318_2024_192_MOESM10_ESM.zip › Figure9/Figure9e/LaminB1.tif]

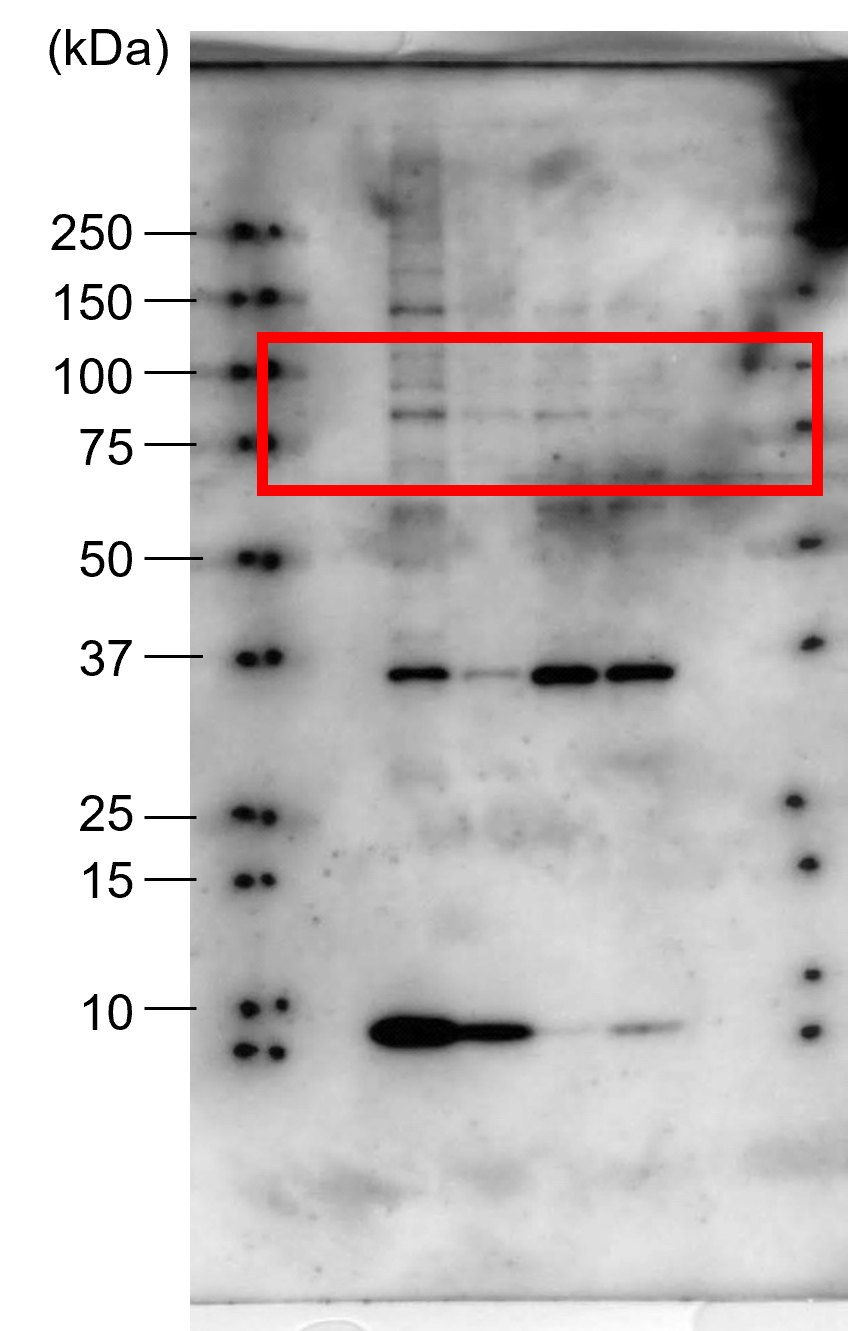

Supplement: Supplementary file 10 — Source data Fig. 9 [file 44318_2024_192_MOESM10_ESM.zip › Figure9/Figure9e/SUMO1.tif]

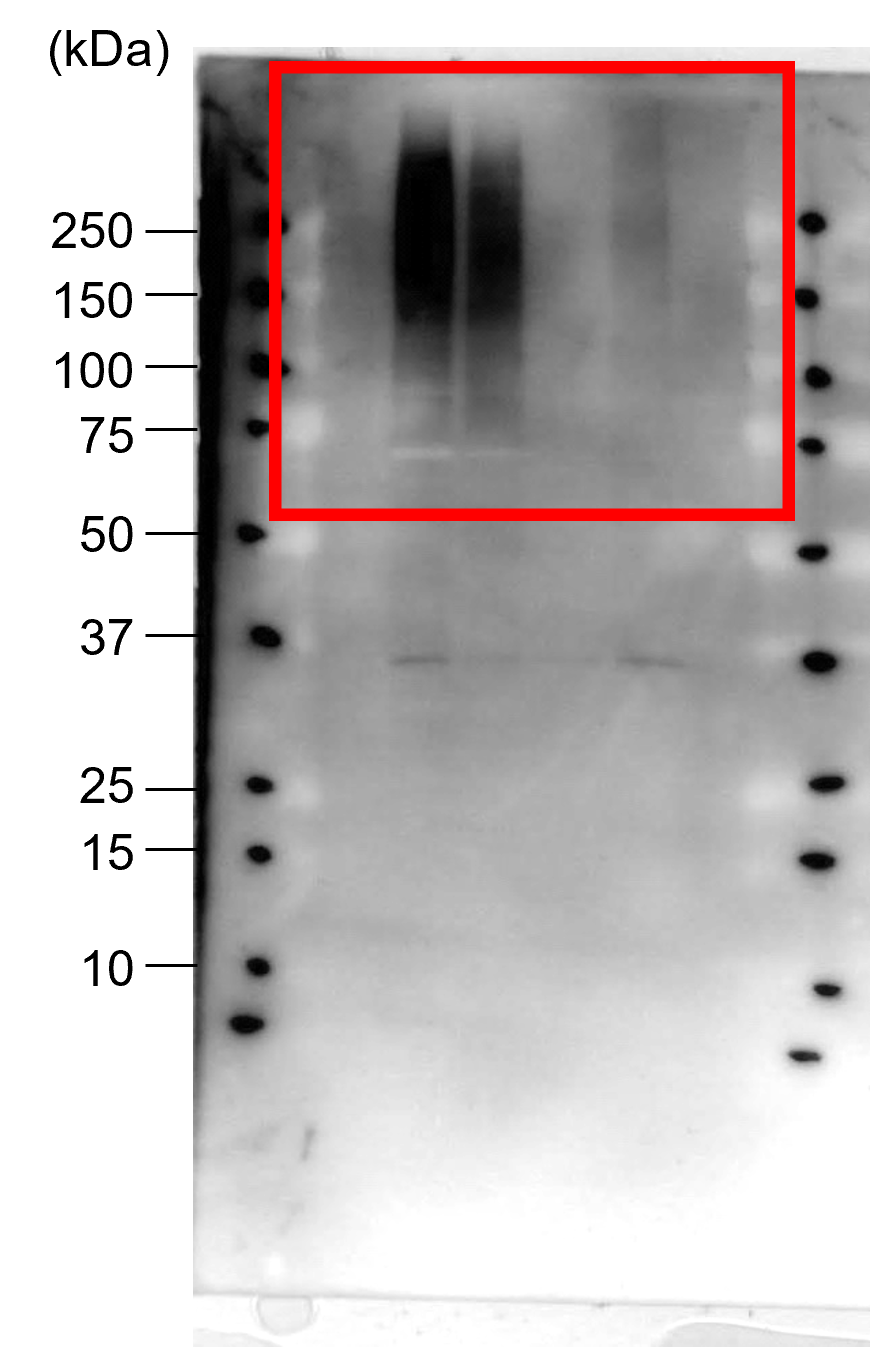

Supplement: Supplementary file 10 — Source data Fig. 9 [file 44318_2024_192_MOESM10_ESM.zip › Figure9/Figure9e/Ub.tif]

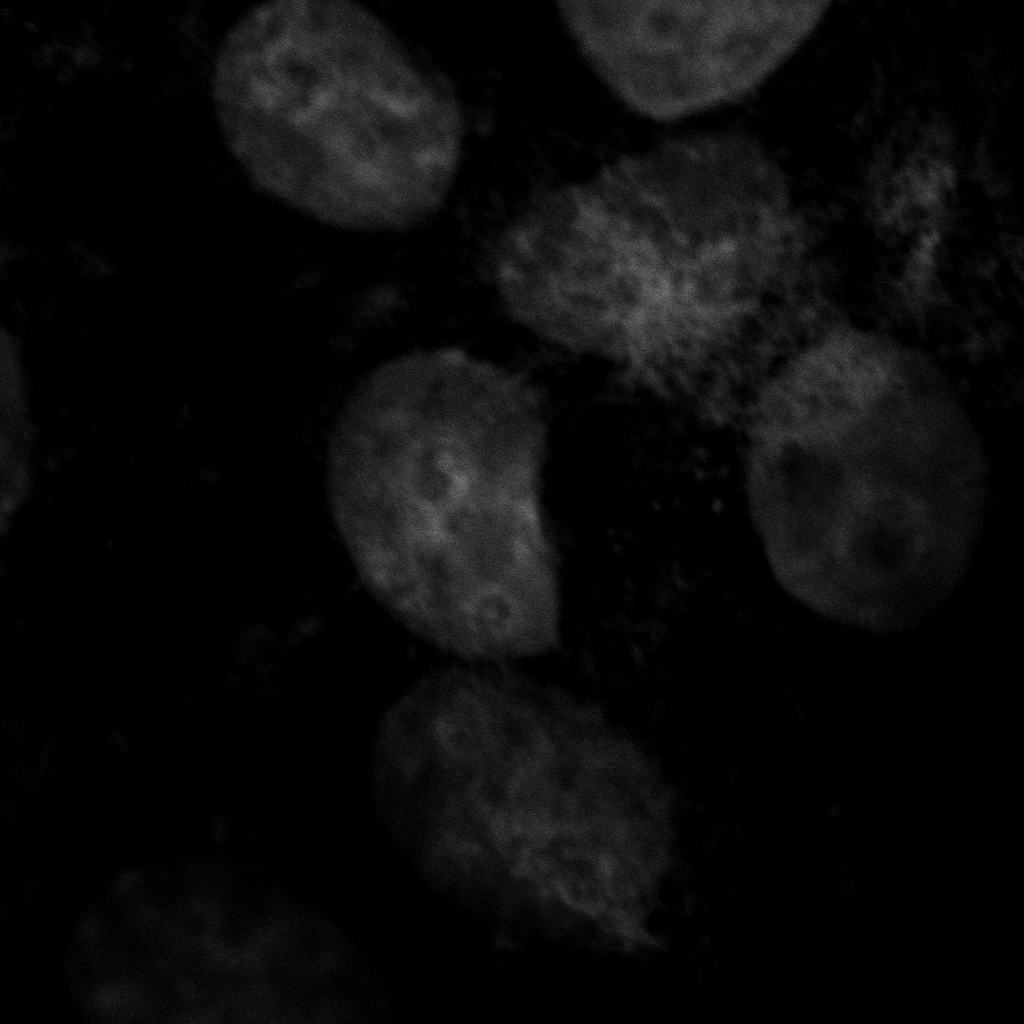

Supplement: Supplementary file 11 — Source data Fig. 10 [file 44318_2024_192_MOESM11_ESM.zip › Figure10/Figure10a/AR/24Q_DAPI.tif]

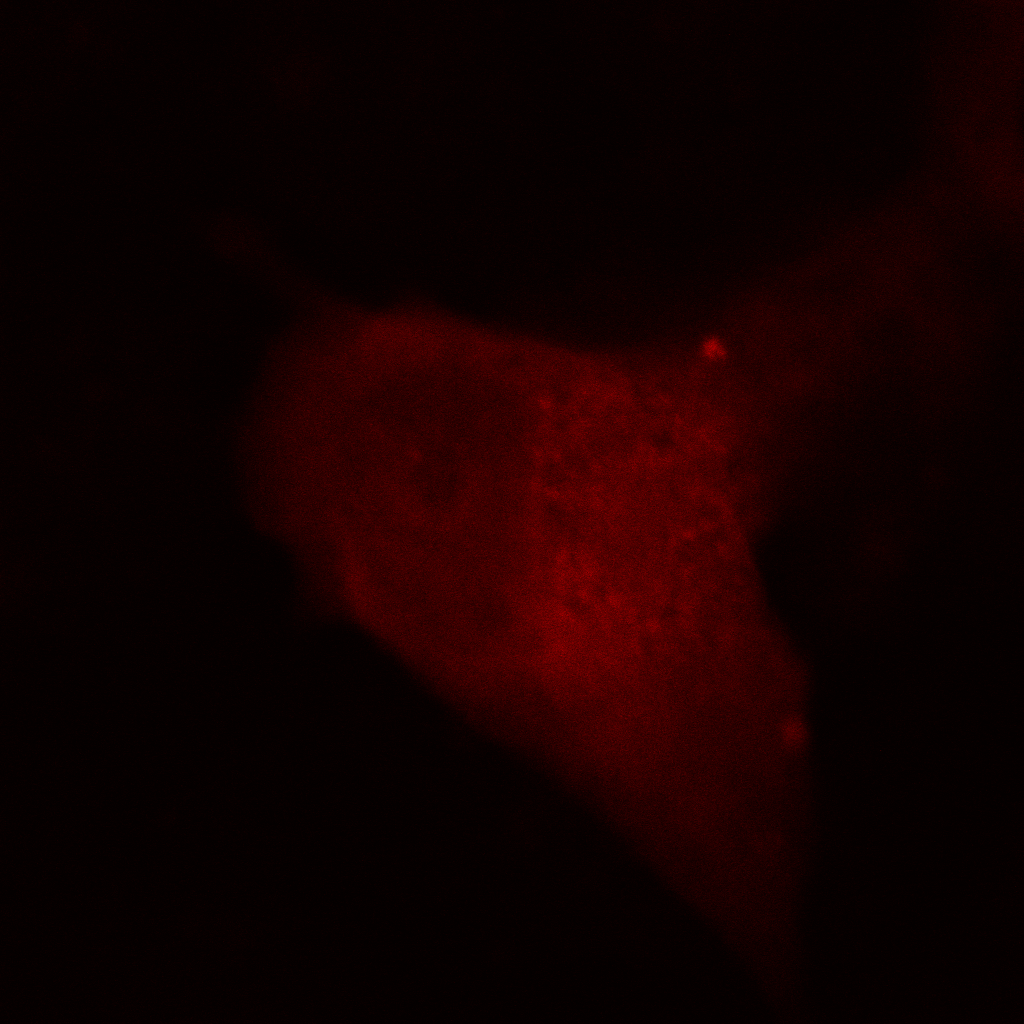

Supplement: Supplementary file 11 — Source data Fig. 10 [file 44318_2024_192_MOESM11_ESM.zip › Figure10/Figure10a/AR/24Q_DsRed-AR.tif]

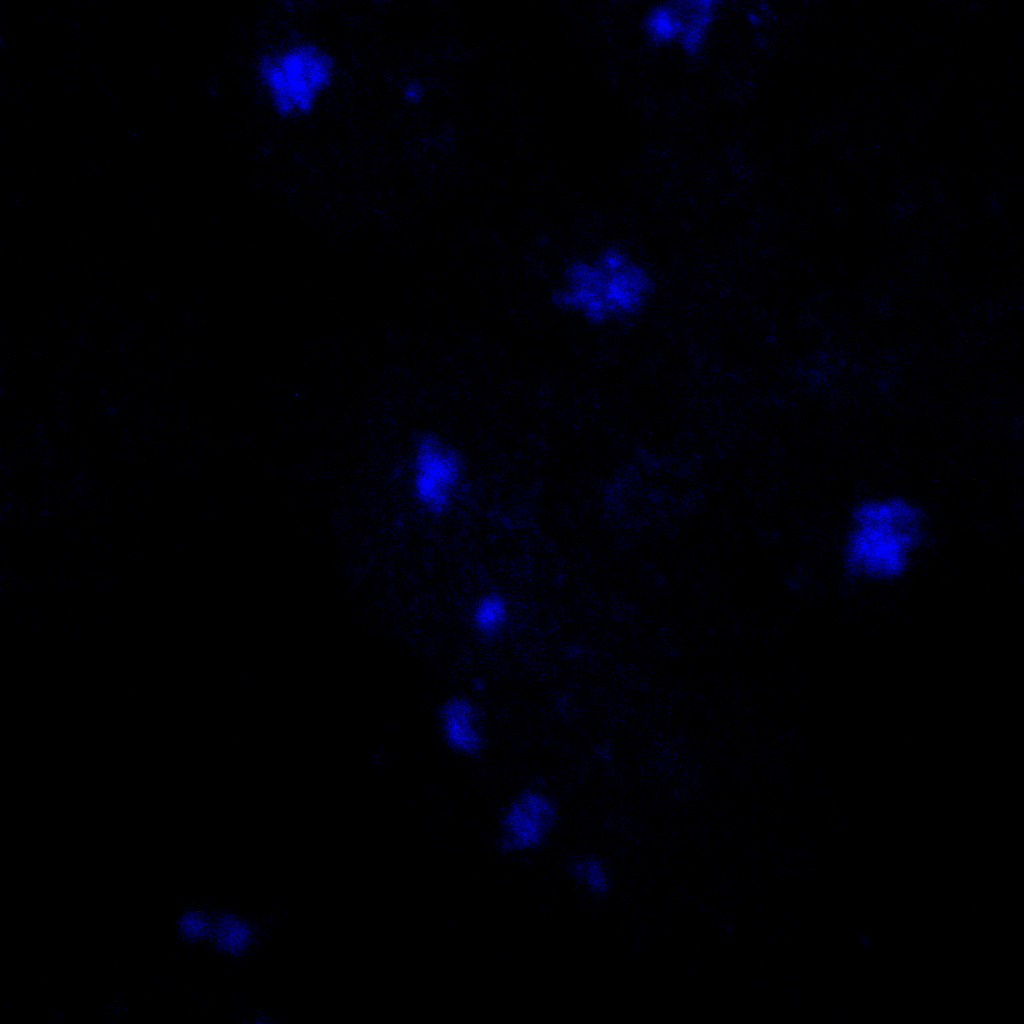

Supplement: Supplementary file 11 — Source data Fig. 10 [file 44318_2024_192_MOESM11_ESM.zip › Figure10/Figure10a/AR/24Q_Fibrillarin.tif]

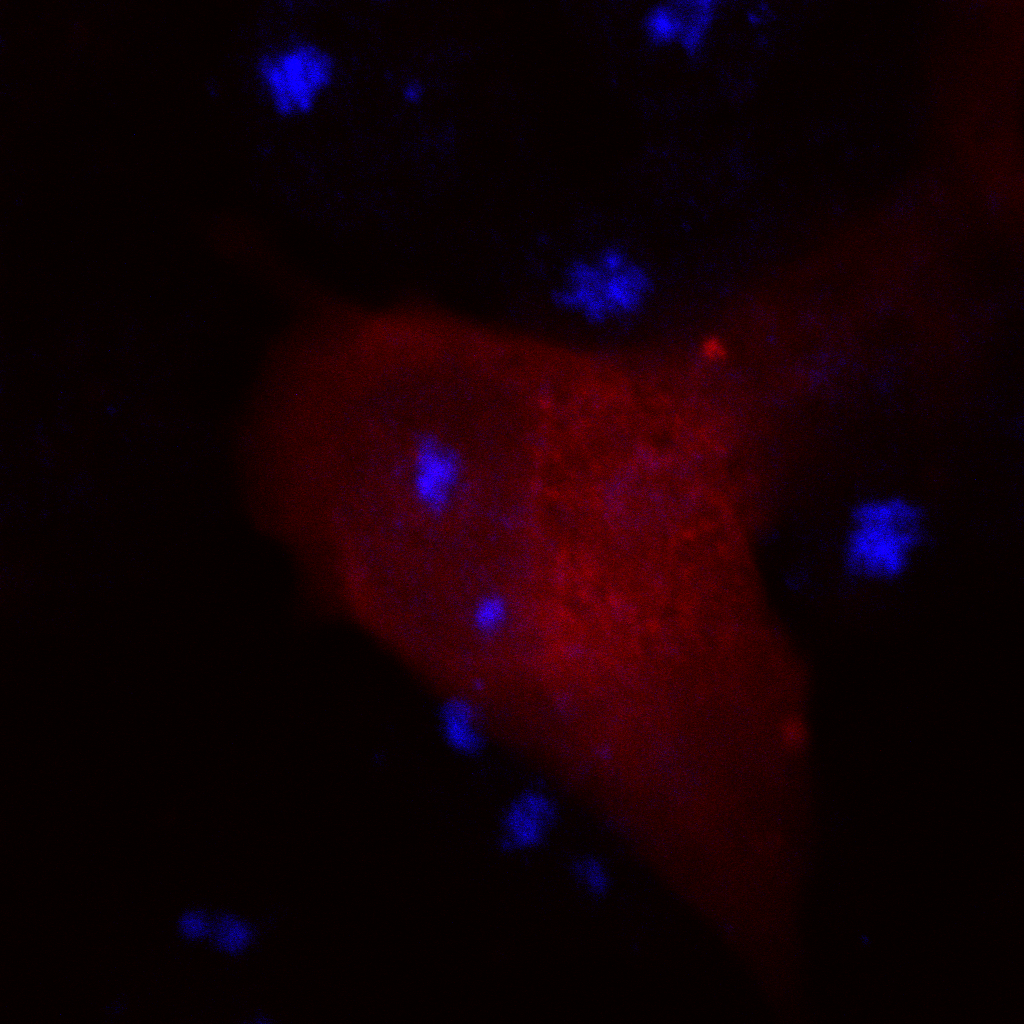

Supplement: Supplementary file 11 — Source data Fig. 10 [file 44318_2024_192_MOESM11_ESM.zip › Figure10/Figure10a/AR/24Q_Merge.tif]

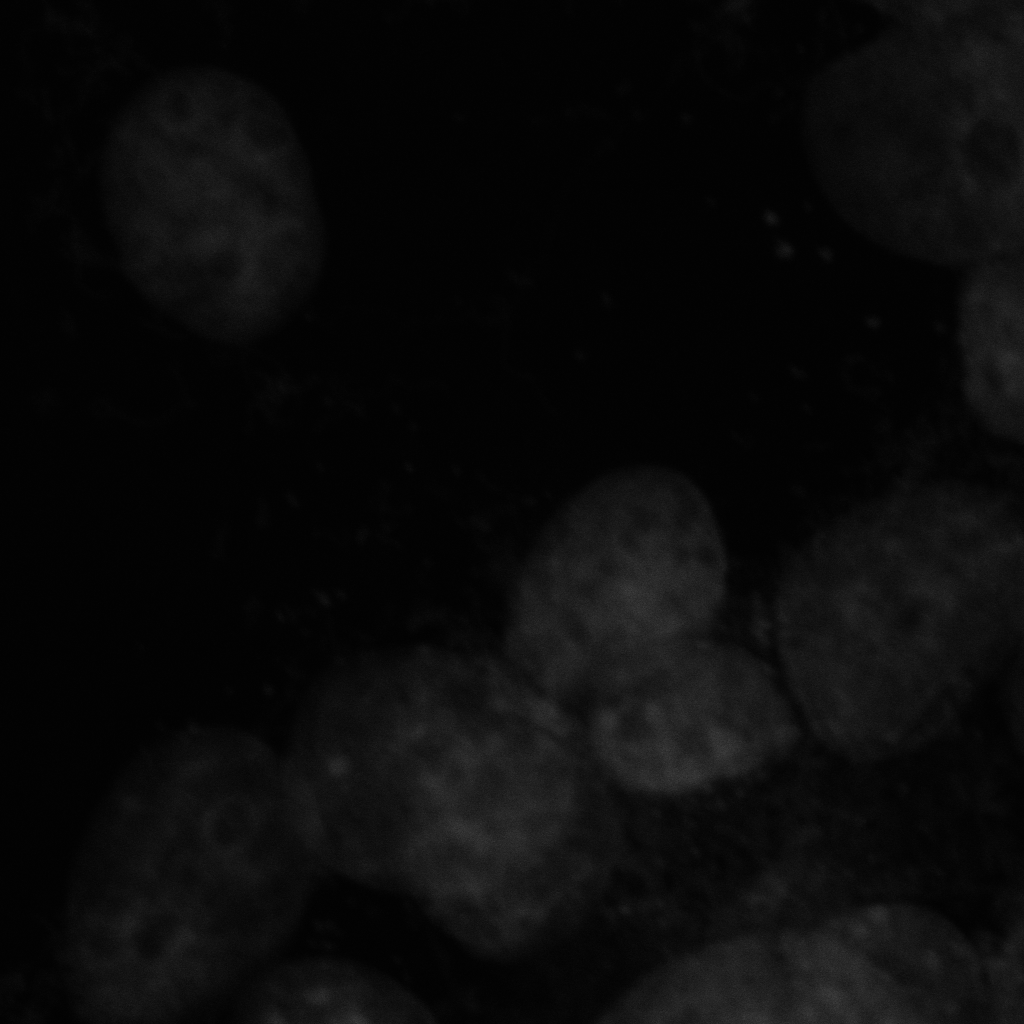

Supplement: Supplementary file 11 — Source data Fig. 10 [file 44318_2024_192_MOESM11_ESM.zip › Figure10/Figure10a/AR/64Q_DAPI.tif]

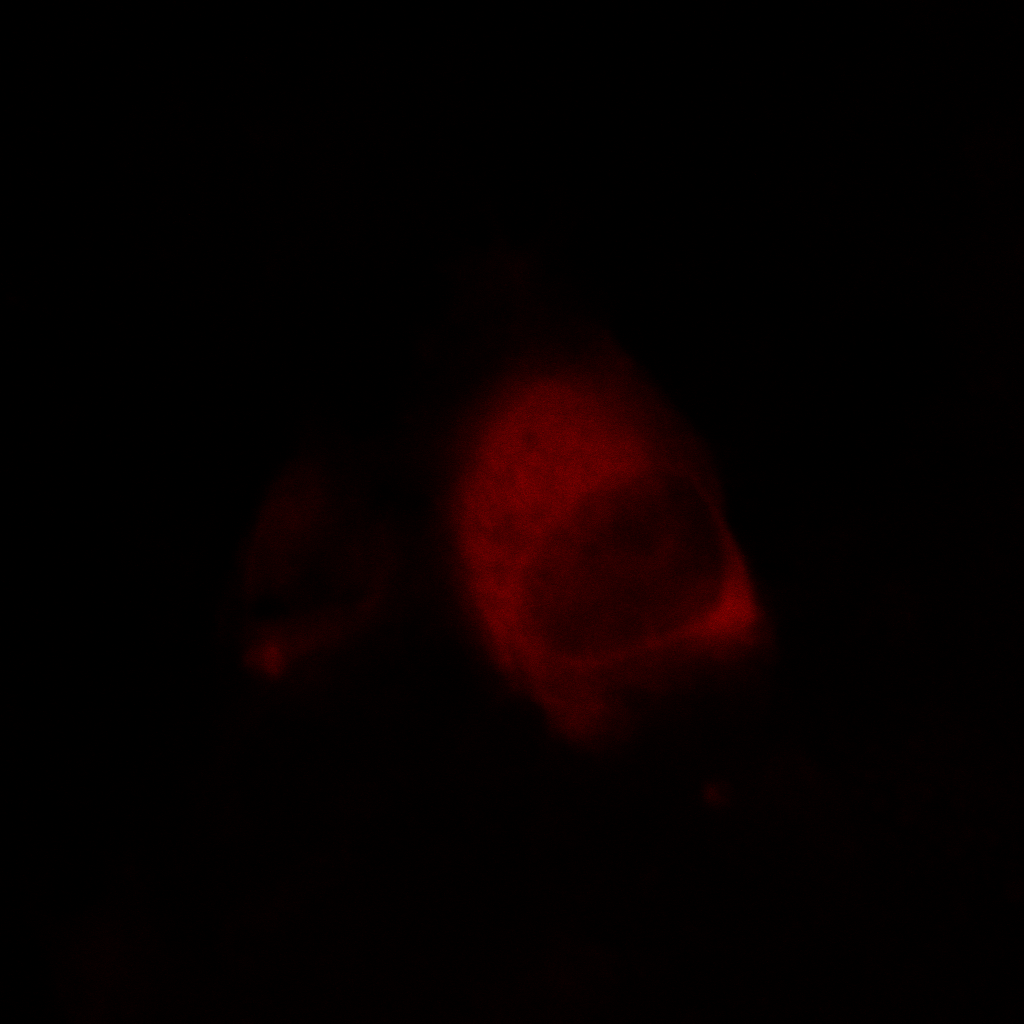

Supplement: Supplementary file 11 — Source data Fig. 10 [file 44318_2024_192_MOESM11_ESM.zip › Figure10/Figure10a/AR/64Q_DsRed-AR.tif]

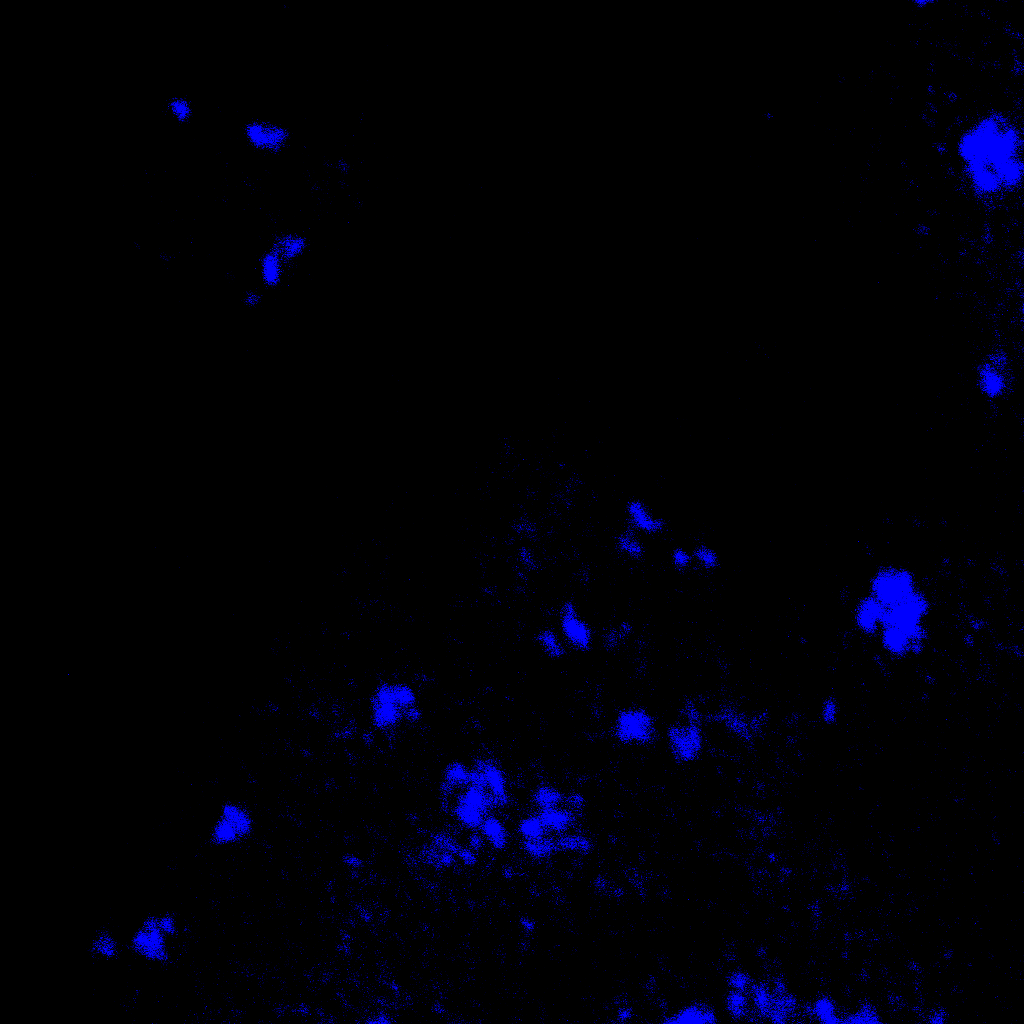

Supplement: Supplementary file 11 — Source data Fig. 10 [file 44318_2024_192_MOESM11_ESM.zip › Figure10/Figure10a/AR/64Q_Fibrillarin.tif]

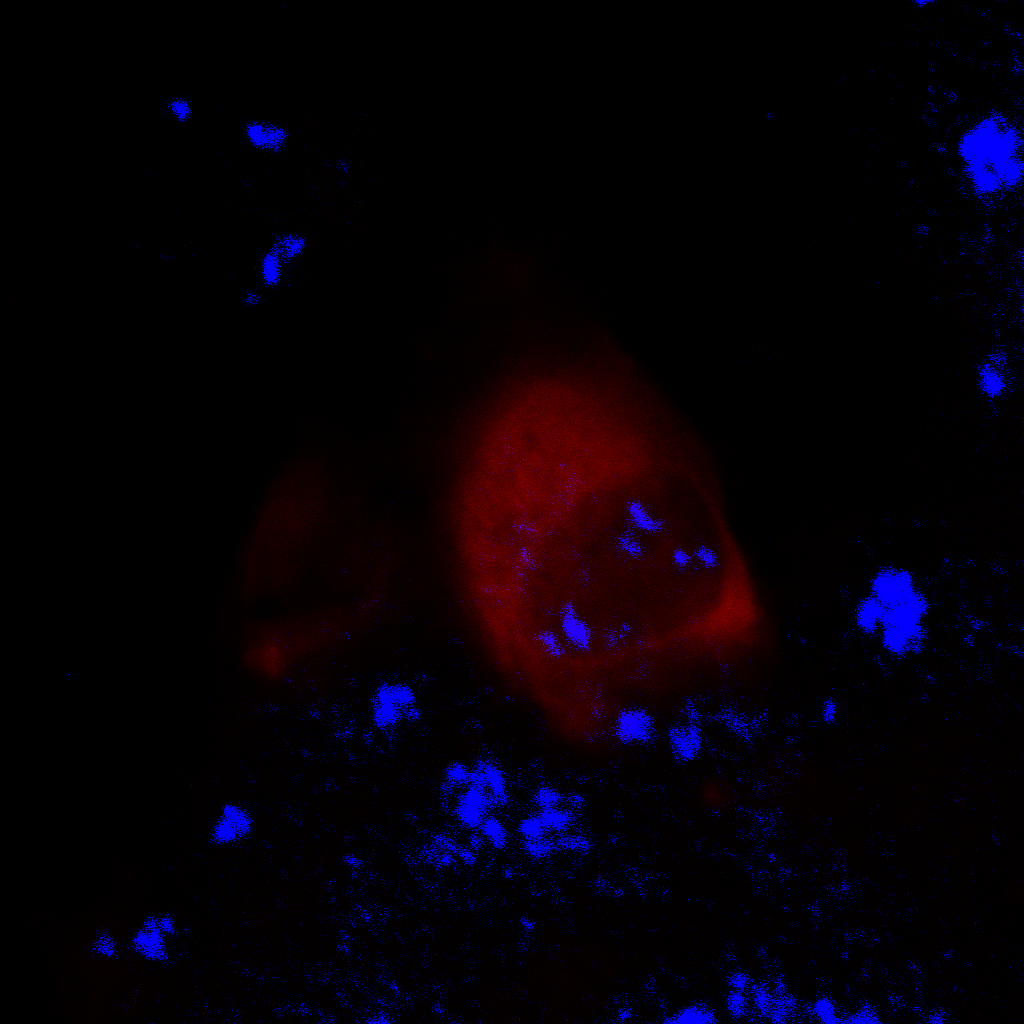

Supplement: Supplementary file 11 — Source data Fig. 10 [file 44318_2024_192_MOESM11_ESM.zip › Figure10/Figure10a/AR/64Q_Merge.tif]

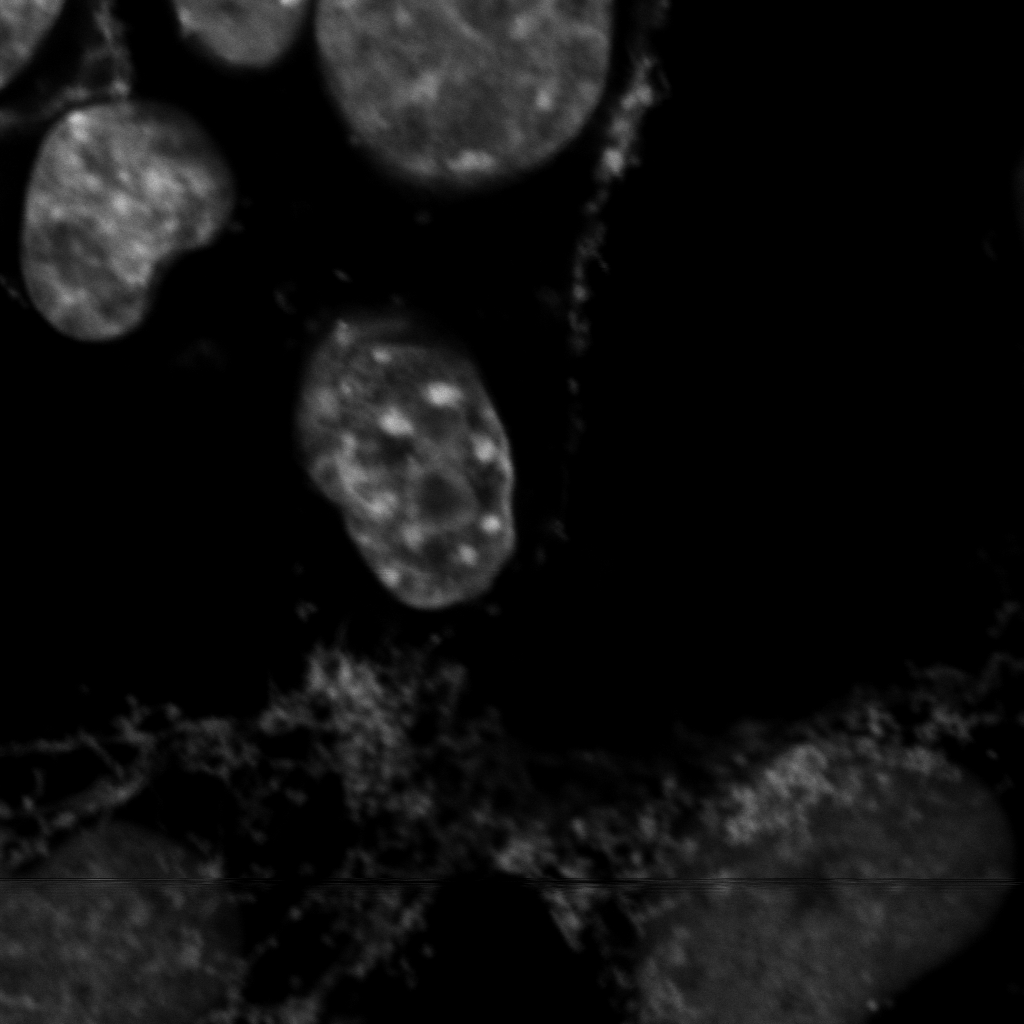

Supplement: Supplementary file 11 — Source data Fig. 10 [file 44318_2024_192_MOESM11_ESM.zip › Figure10/Figure10a/Atxn1/33Q_DAPI.tif]

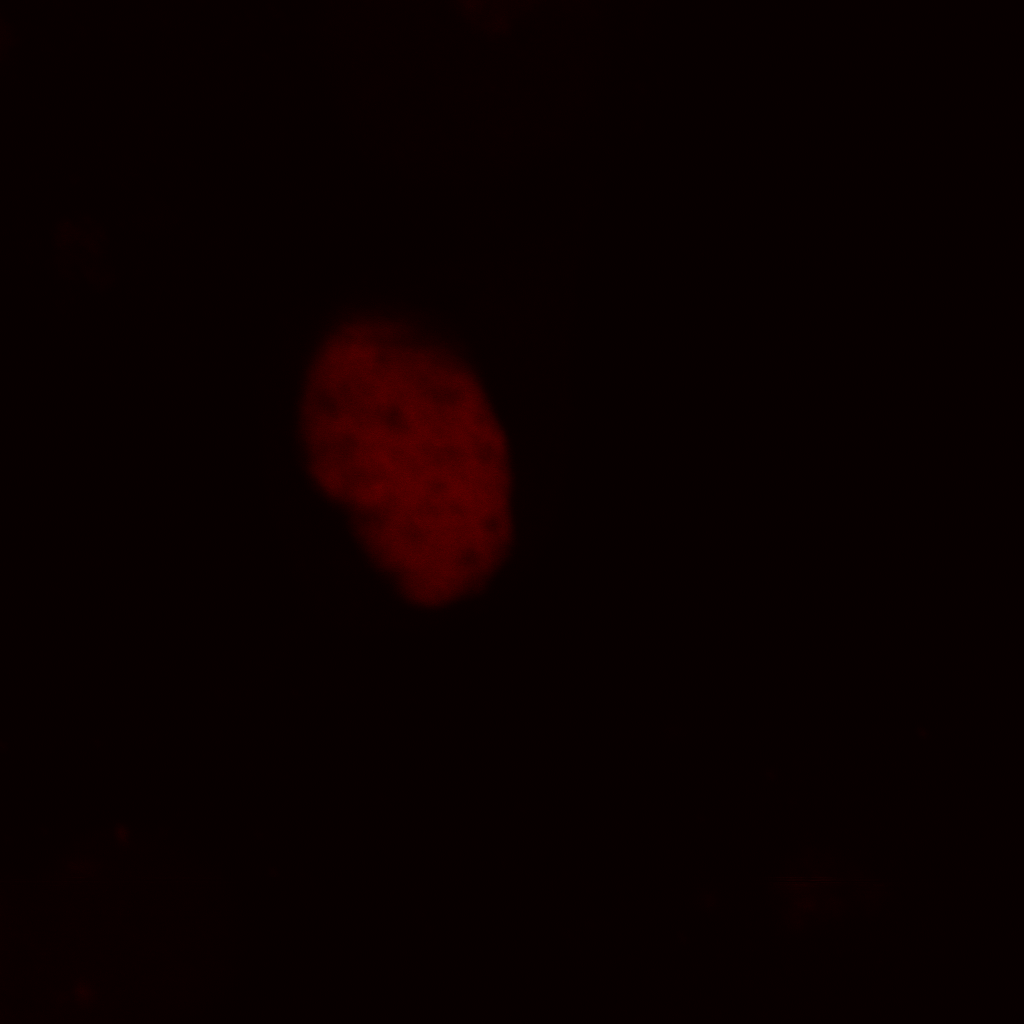

Supplement: Supplementary file 11 — Source data Fig. 10 [file 44318_2024_192_MOESM11_ESM.zip › Figure10/Figure10a/Atxn1/33Q_DsRed-Atxn1.tif]

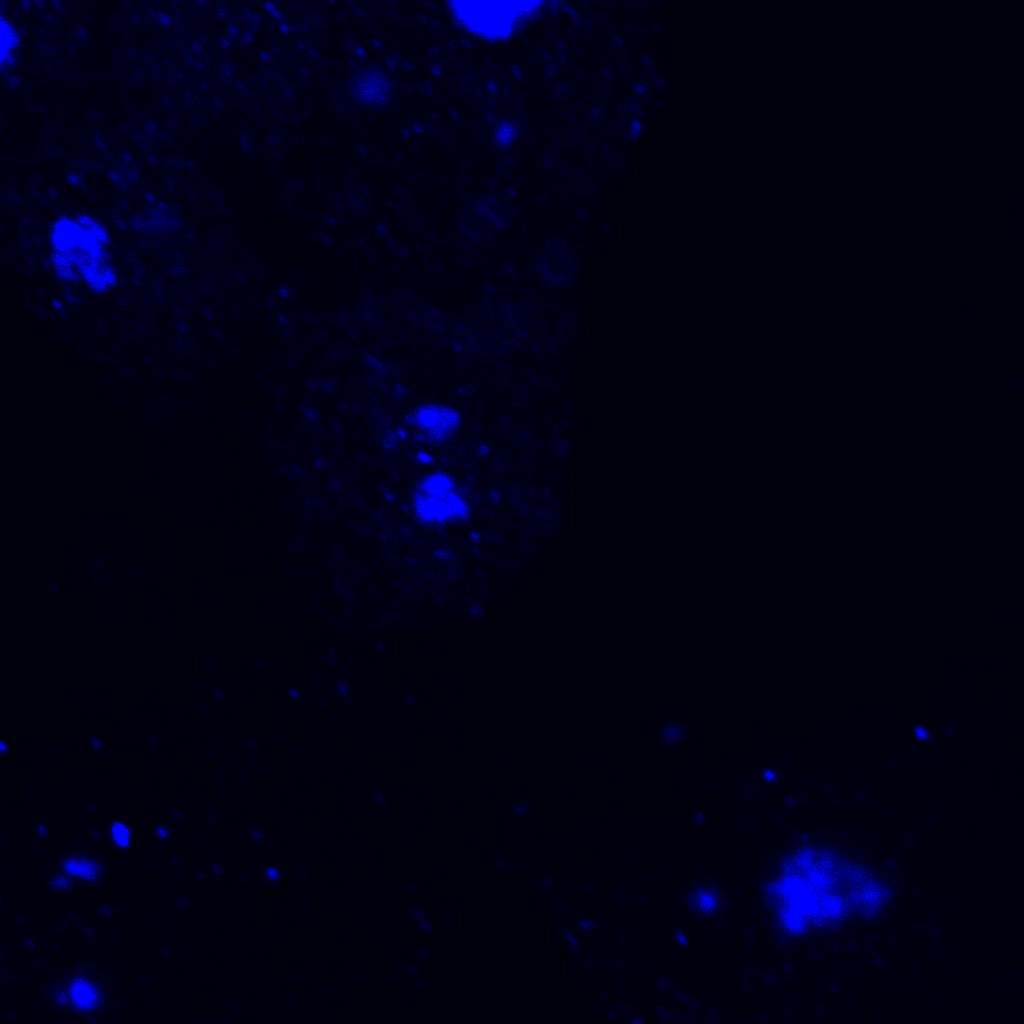

Supplement: Supplementary file 11 — Source data Fig. 10 [file 44318_2024_192_MOESM11_ESM.zip › Figure10/Figure10a/Atxn1/33Q_Fibrillarin.tif]

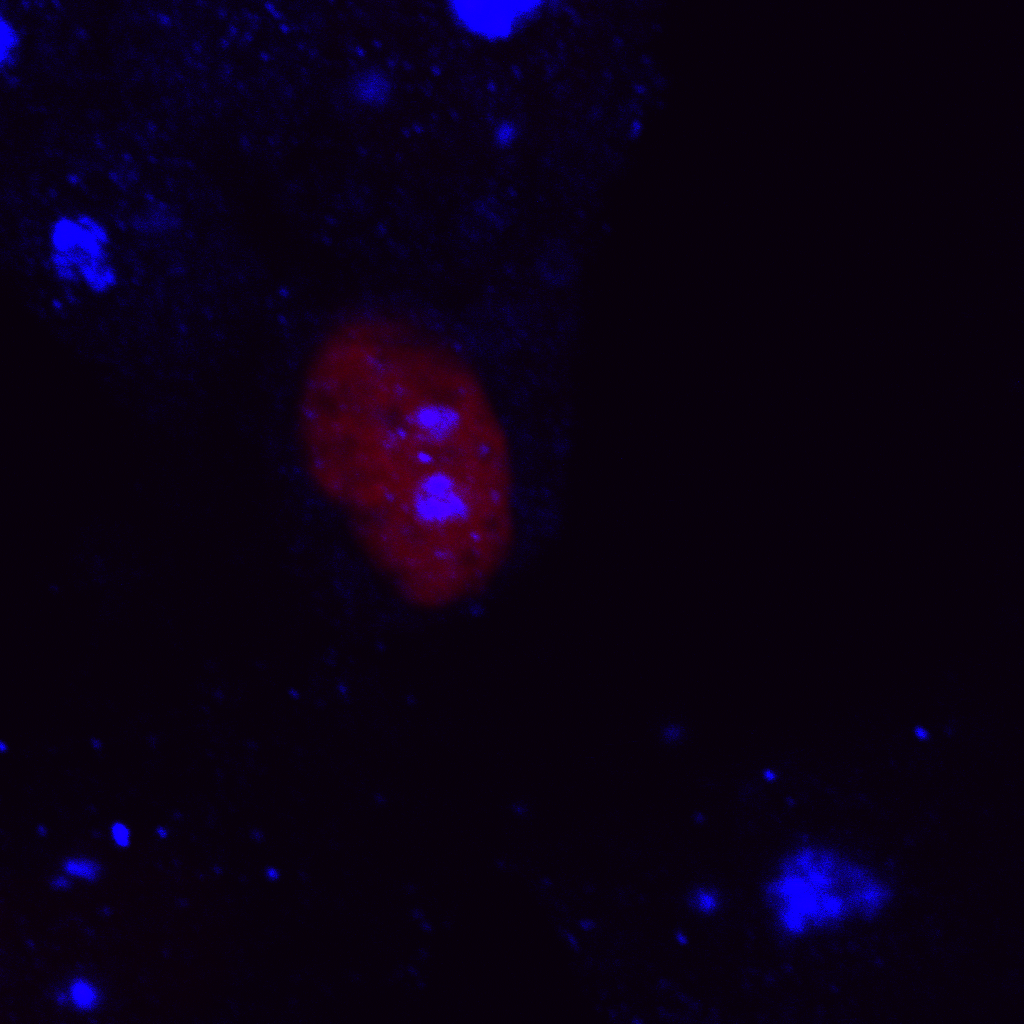

Supplement: Supplementary file 11 — Source data Fig. 10 [file 44318_2024_192_MOESM11_ESM.zip › Figure10/Figure10a/Atxn1/33Q_Merge.tif]

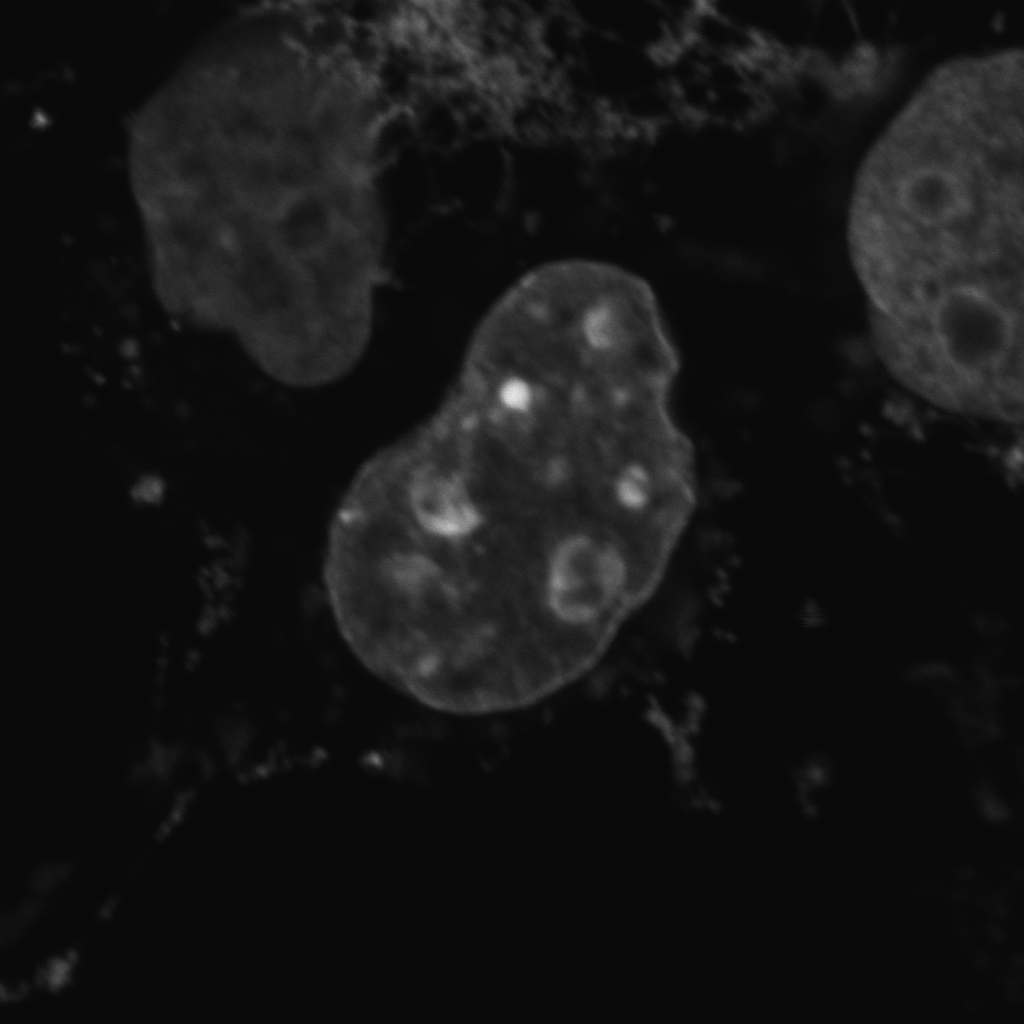

Supplement: Supplementary file 11 — Source data Fig. 10 [file 44318_2024_192_MOESM11_ESM.zip › Figure10/Figure10a/Atxn1/86Q_DAPI.tif]

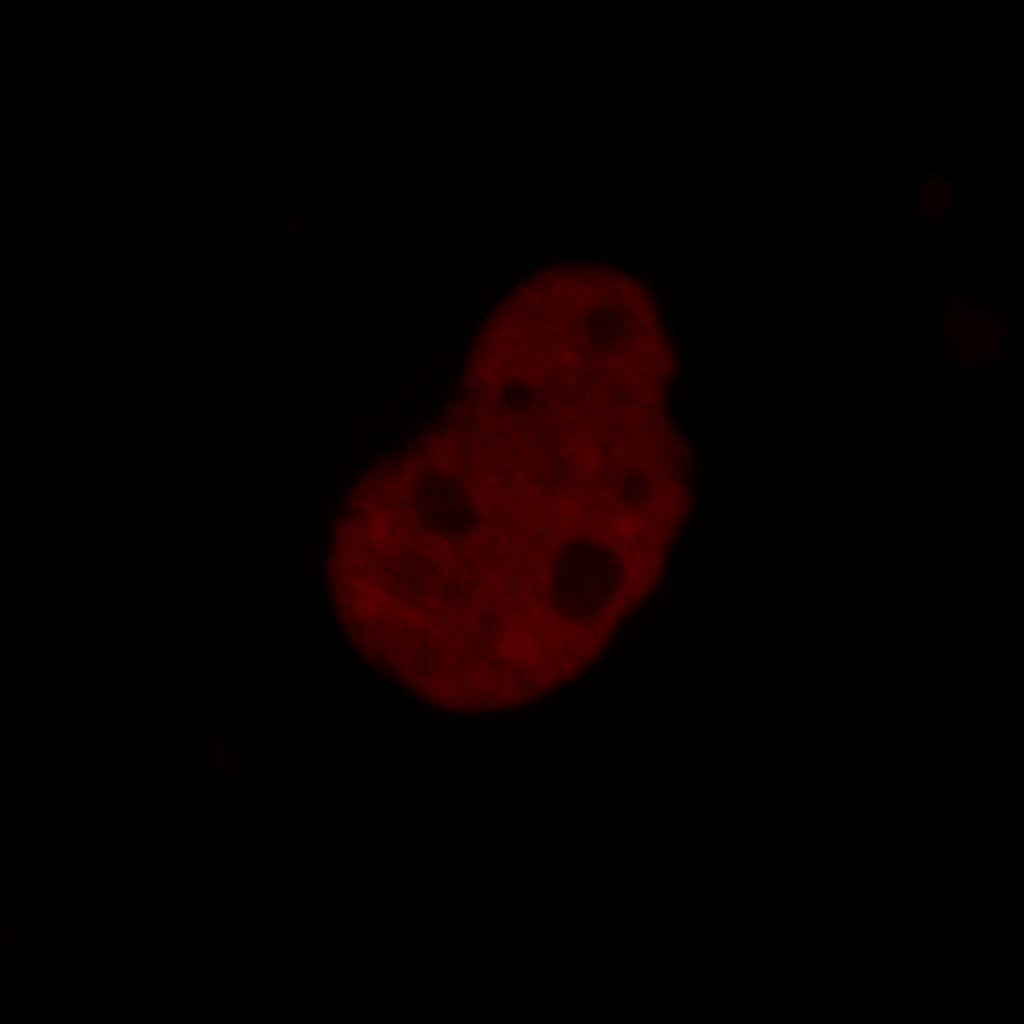

Supplement: Supplementary file 11 — Source data Fig. 10 [file 44318_2024_192_MOESM11_ESM.zip › Figure10/Figure10a/Atxn1/86Q_DsRed-Atxn1.tif]

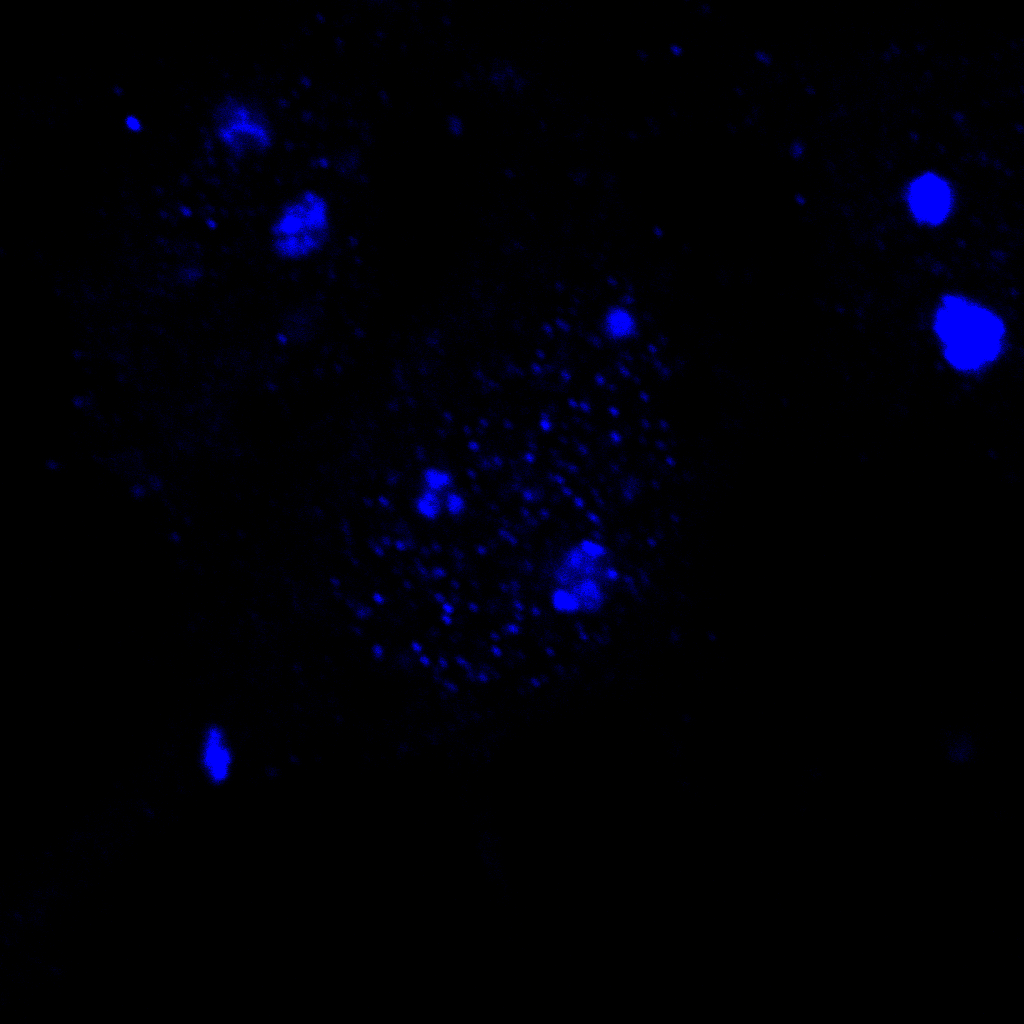

Supplement: Supplementary file 11 — Source data Fig. 10 [file 44318_2024_192_MOESM11_ESM.zip › Figure10/Figure10a/Atxn1/86Q_Fibrillarin.tif]

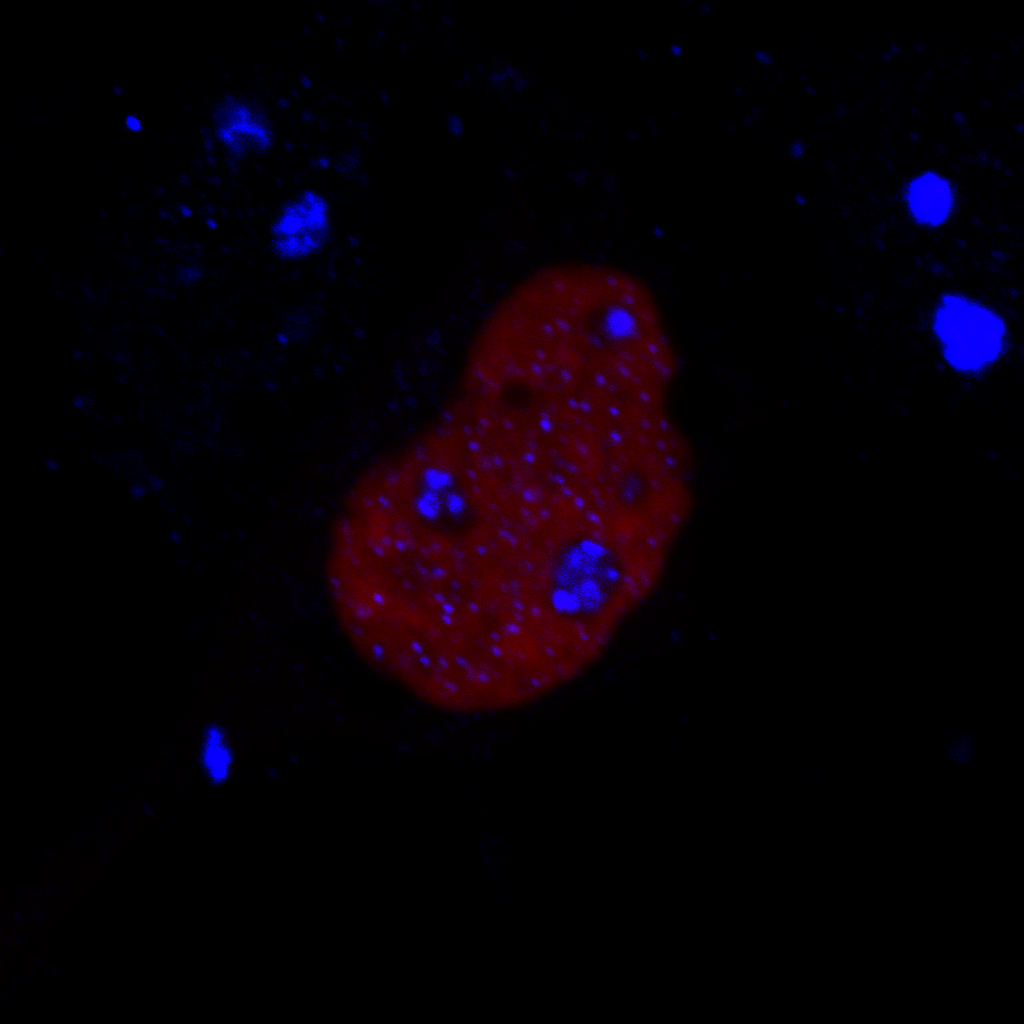

Supplement: Supplementary file 11 — Source data Fig. 10 [file 44318_2024_192_MOESM11_ESM.zip › Figure10/Figure10a/Atxn1/86Q_Merge.tif]

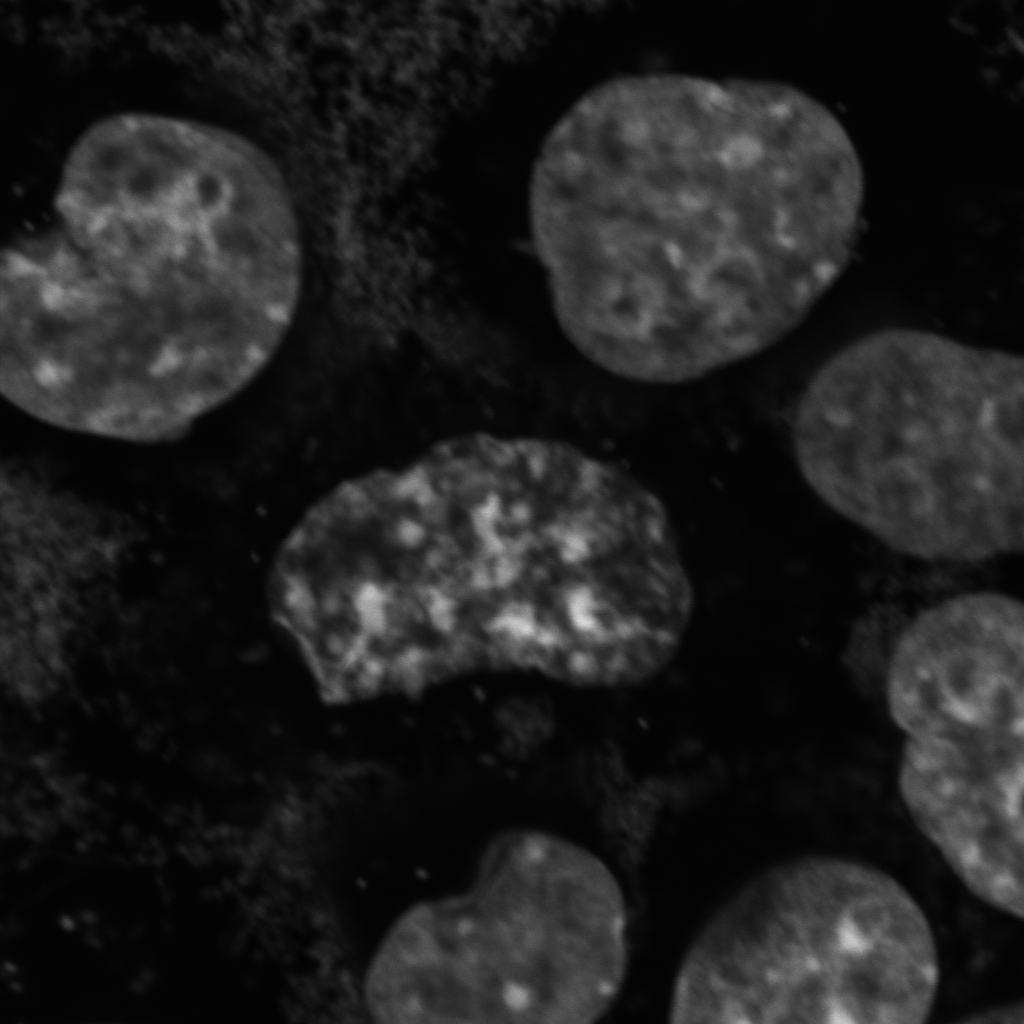

Supplement: Supplementary file 11 — Source data Fig. 10 [file 44318_2024_192_MOESM11_ESM.zip › Figure10/Figure10a/Atxn7/10Q_DAPI.tif]

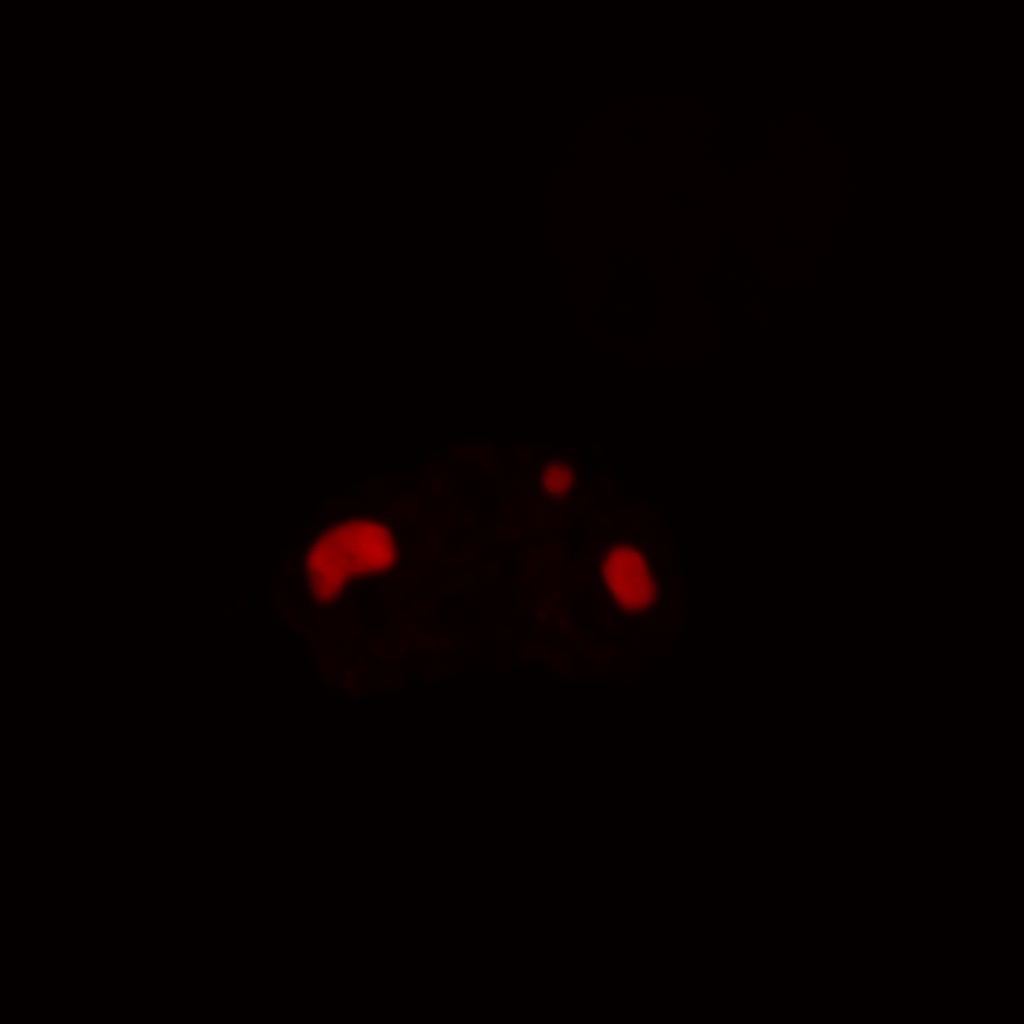

Supplement: Supplementary file 11 — Source data Fig. 10 [file 44318_2024_192_MOESM11_ESM.zip › Figure10/Figure10a/Atxn7/10Q_DsRed-Atxn7.tif]

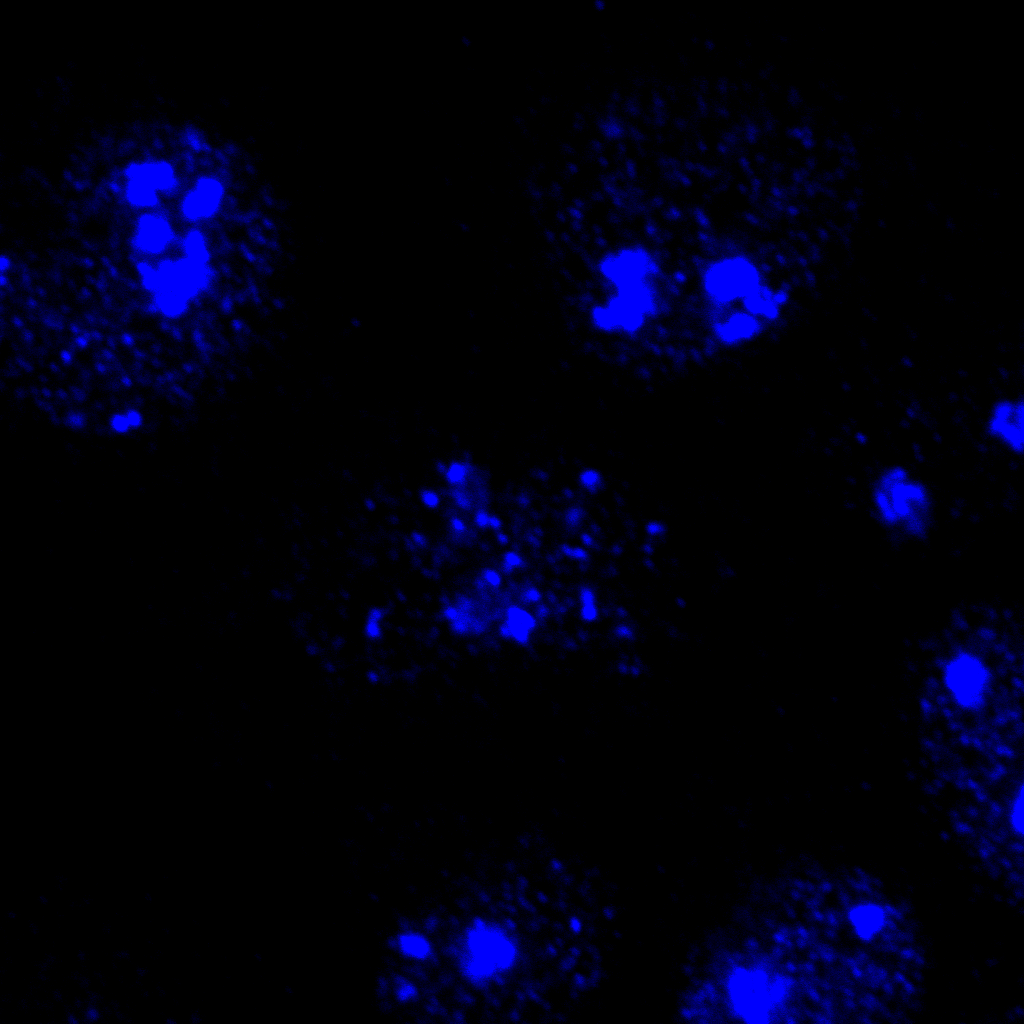

Supplement: Supplementary file 11 — Source data Fig. 10 [file 44318_2024_192_MOESM11_ESM.zip › Figure10/Figure10a/Atxn7/10Q_Fibrillarin.tif]

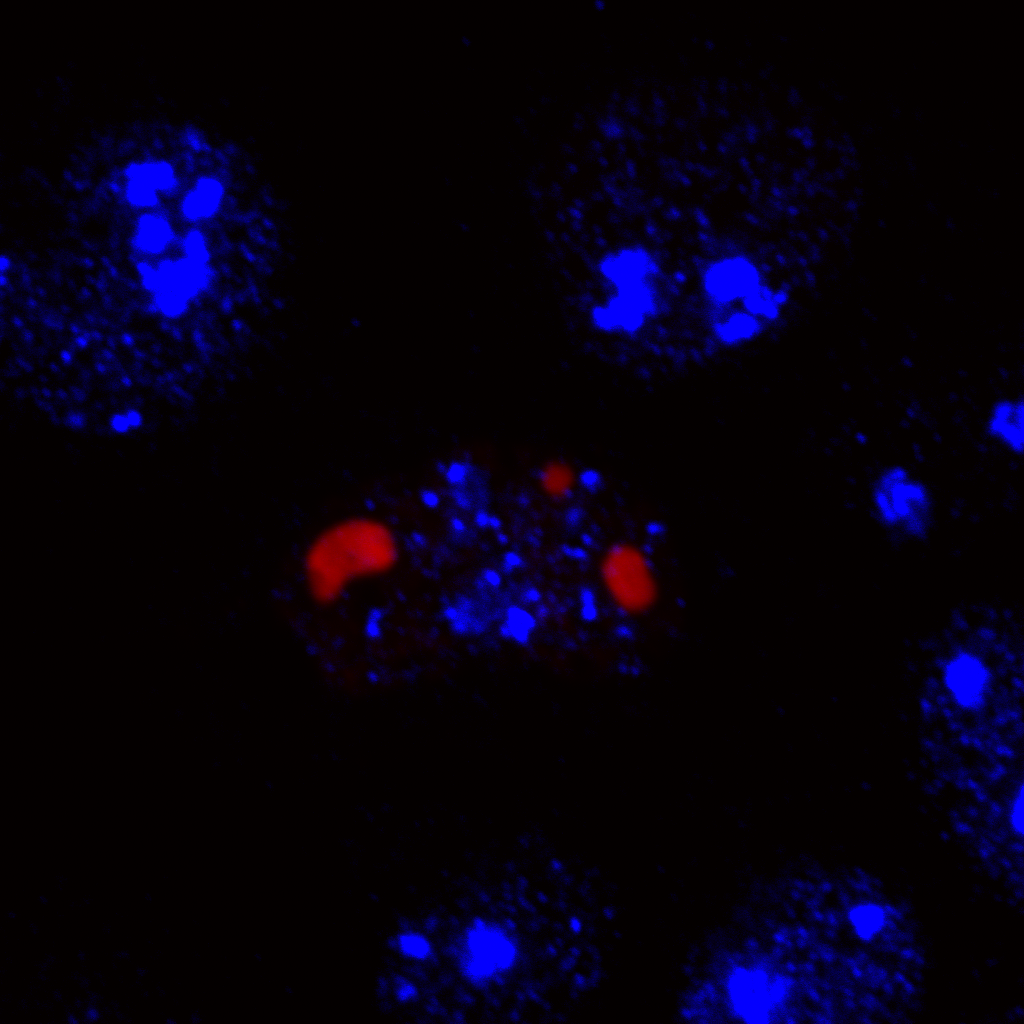

Supplement: Supplementary file 11 — Source data Fig. 10 [file 44318_2024_192_MOESM11_ESM.zip › Figure10/Figure10a/Atxn7/10Q_Merge.tif]

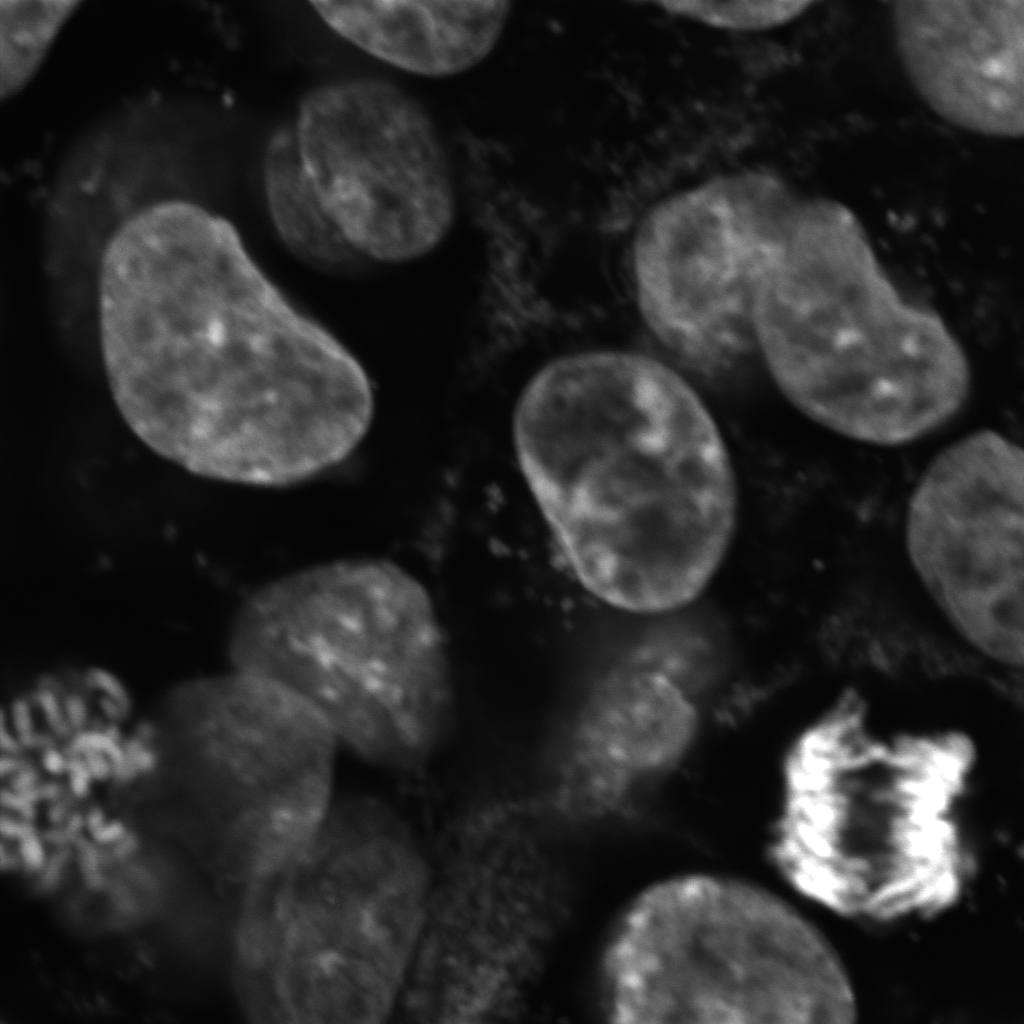

Supplement: Supplementary file 11 — Source data Fig. 10 [file 44318_2024_192_MOESM11_ESM.zip › Figure10/Figure10a/Atxn7/92Q_DAPI.tif]

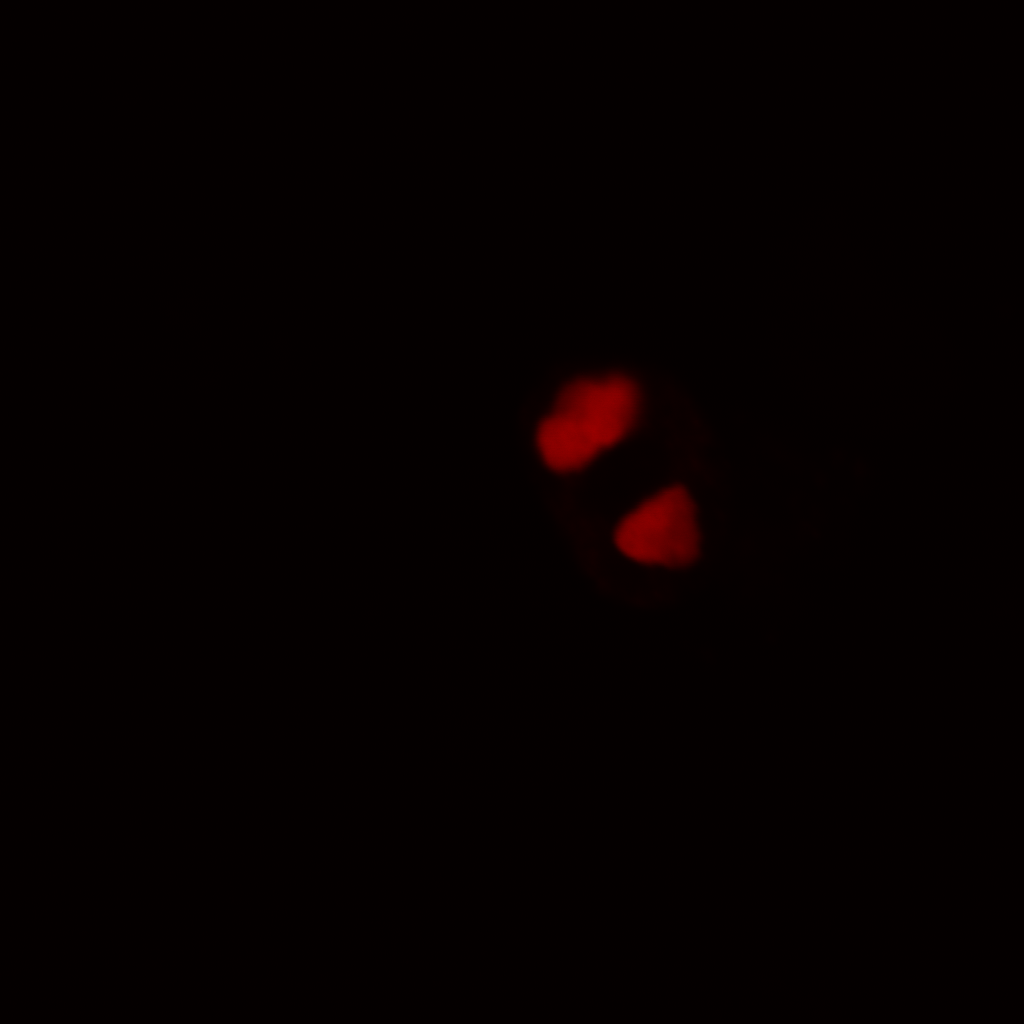

Supplement: Supplementary file 11 — Source data Fig. 10 [file 44318_2024_192_MOESM11_ESM.zip › Figure10/Figure10a/Atxn7/92Q_DsRed-Atxn7.tif]

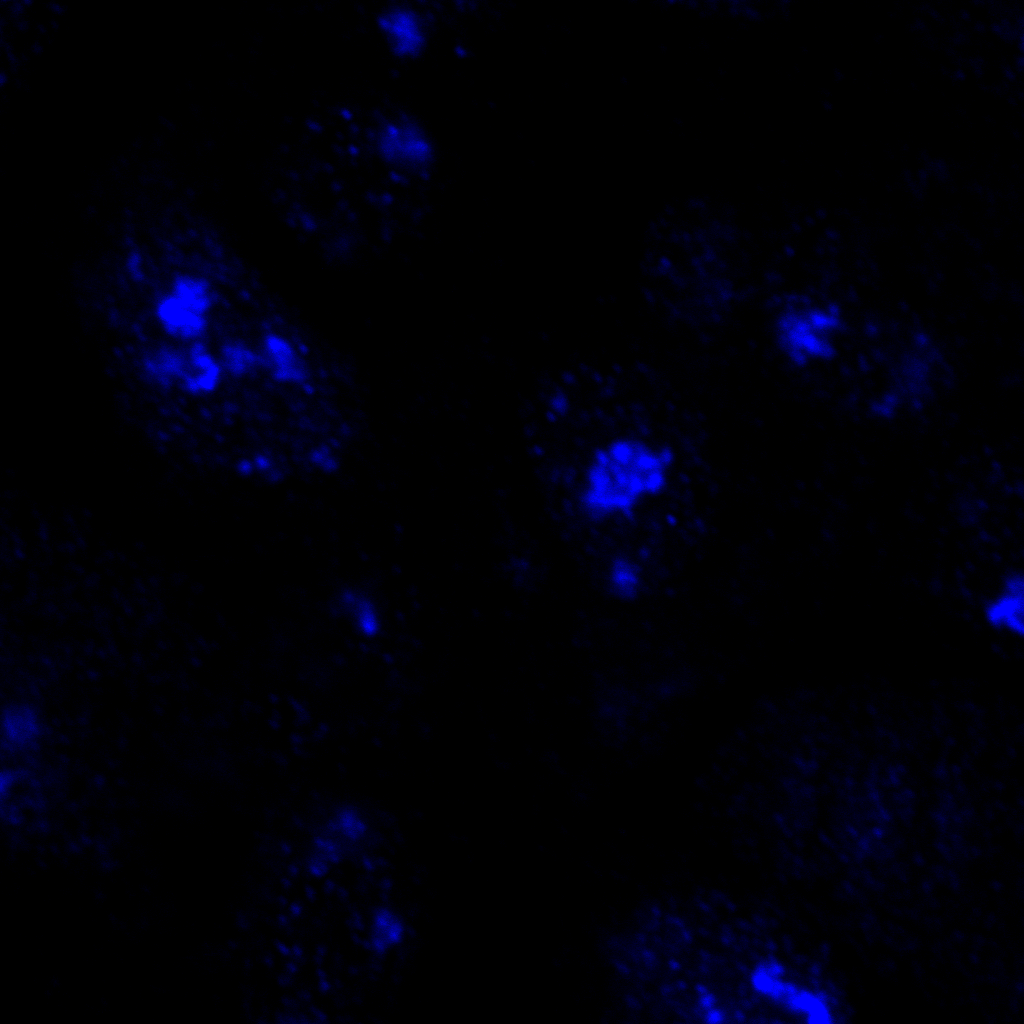

Supplement: Supplementary file 11 — Source data Fig. 10 [file 44318_2024_192_MOESM11_ESM.zip › Figure10/Figure10a/Atxn7/92Q_Fibrillarin.tif]

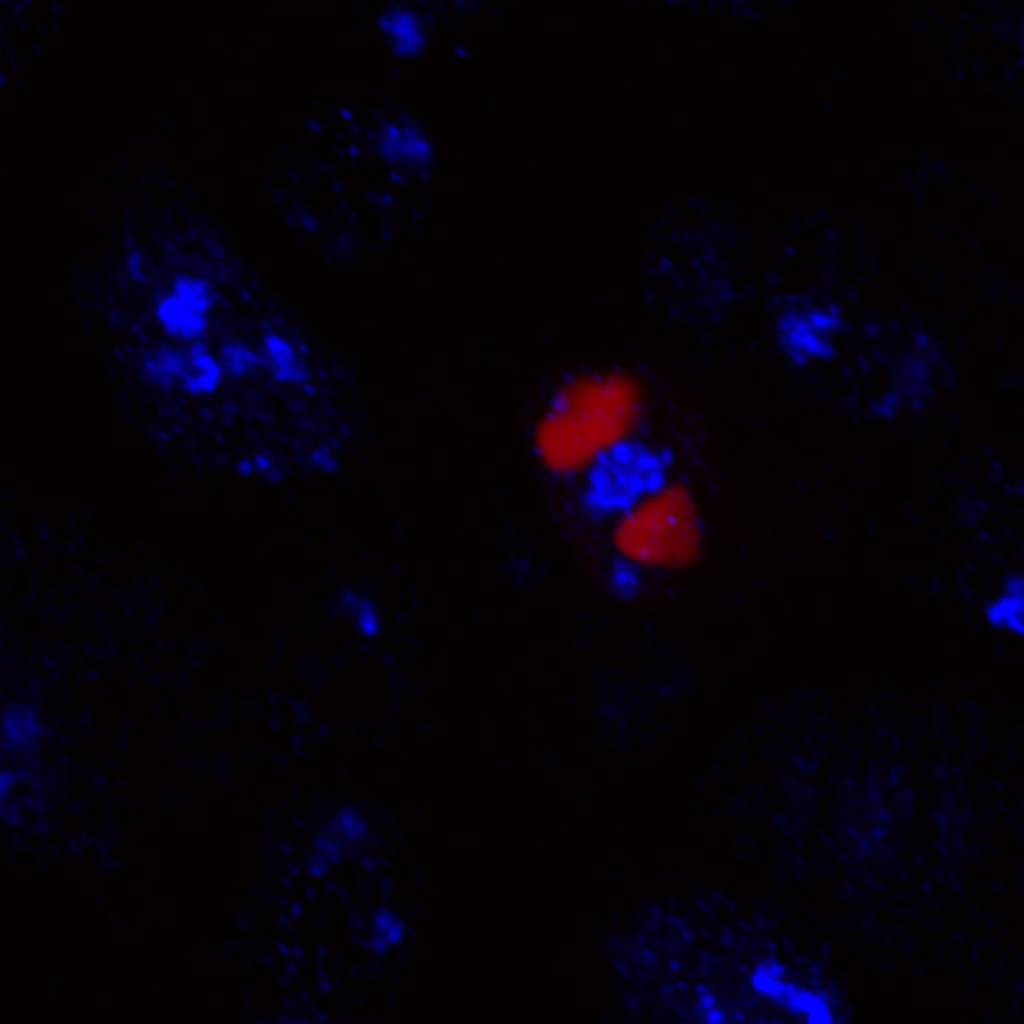

Supplement: Supplementary file 11 — Source data Fig. 10 [file 44318_2024_192_MOESM11_ESM.zip › Figure10/Figure10a/Atxn7/92Q_Merge.tif]

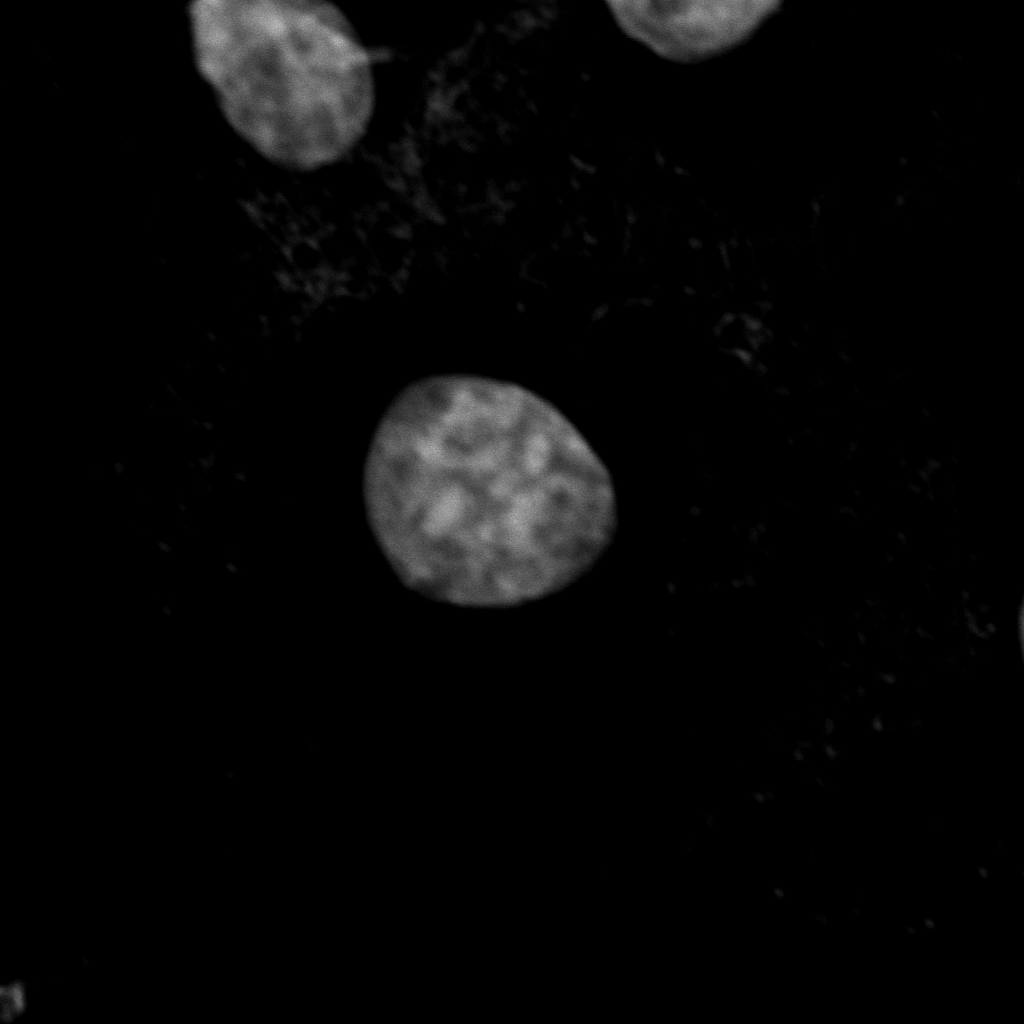

Supplement: Supplementary file 11 — Source data Fig. 10 [file 44318_2024_192_MOESM11_ESM.zip › Figure10/Figure10a/Htt/103Q_DAPI.tif]

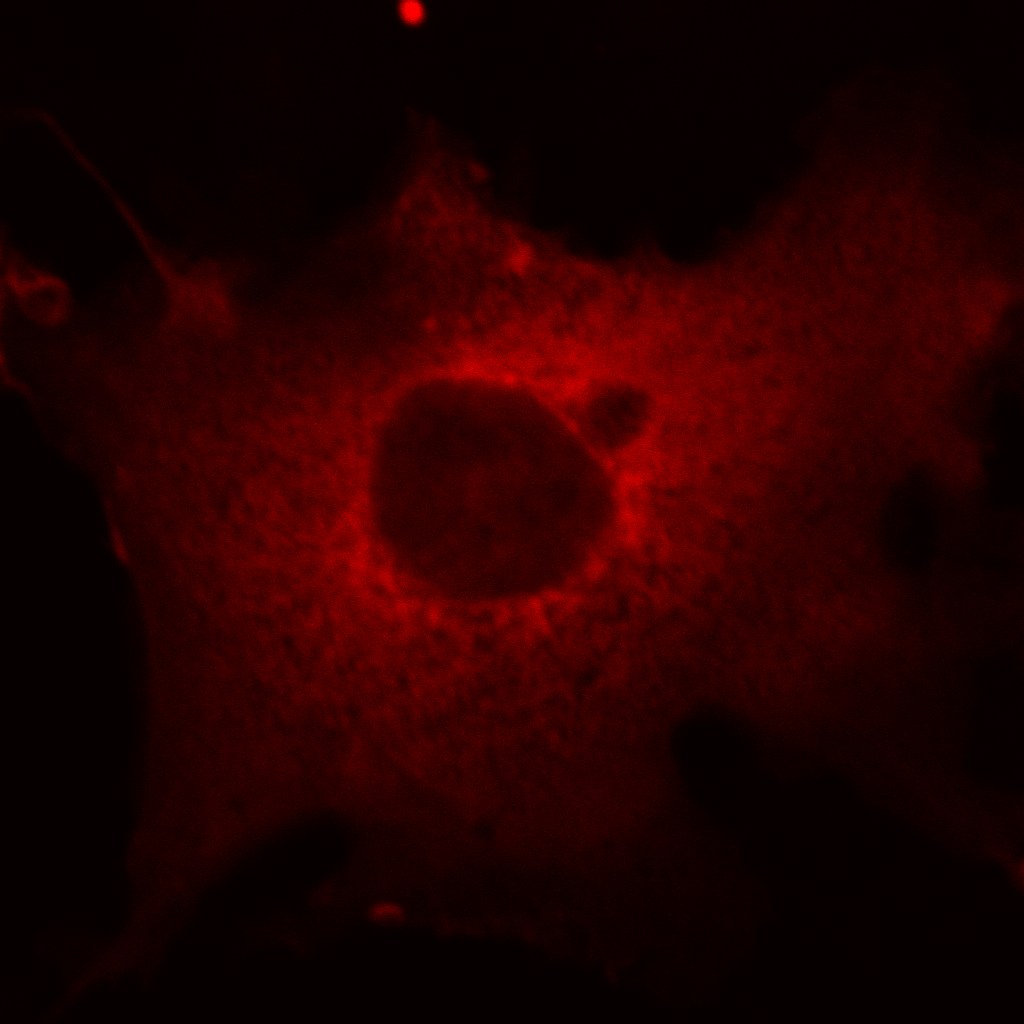

Supplement: Supplementary file 11 — Source data Fig. 10 [file 44318_2024_192_MOESM11_ESM.zip › Figure10/Figure10a/Htt/103Q_DsRed-Htt.tif]

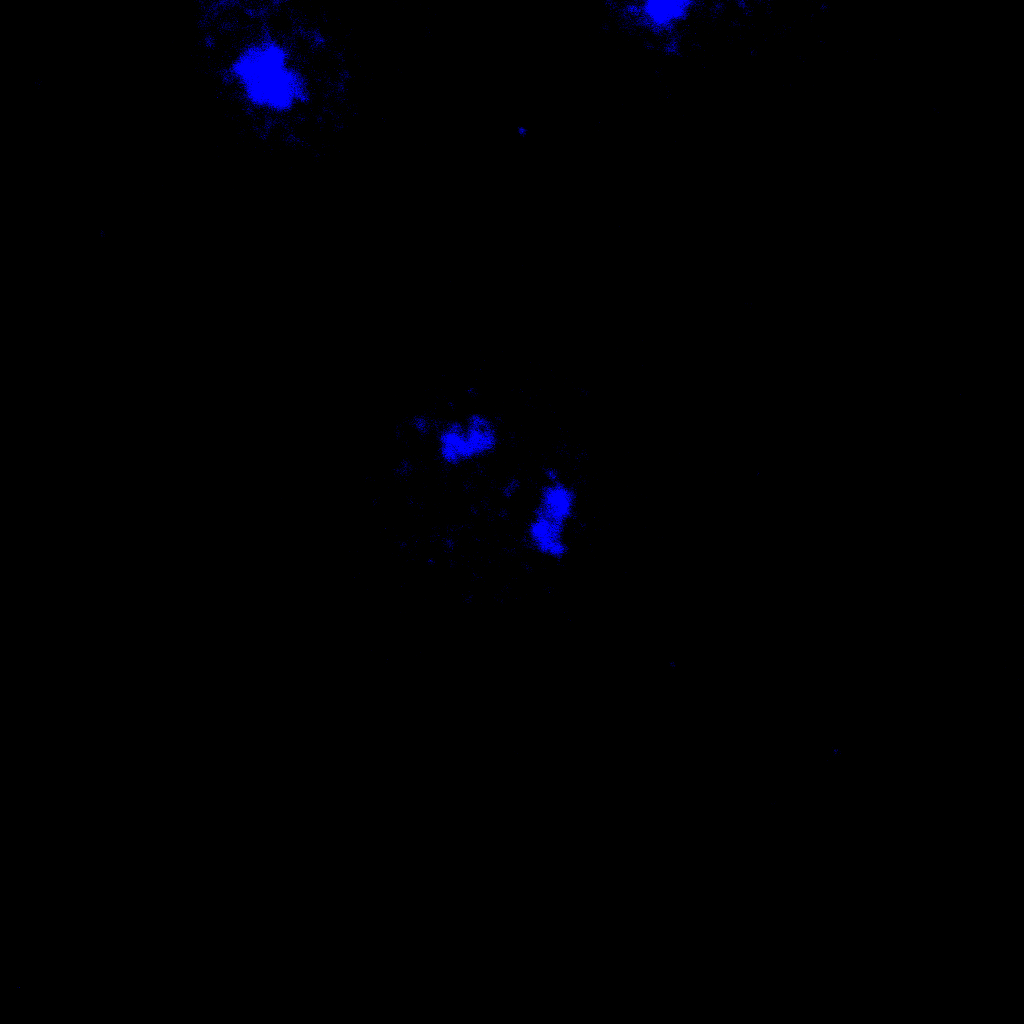

Supplement: Supplementary file 11 — Source data Fig. 10 [file 44318_2024_192_MOESM11_ESM.zip › Figure10/Figure10a/Htt/103Q_Fibrillarin.tif]

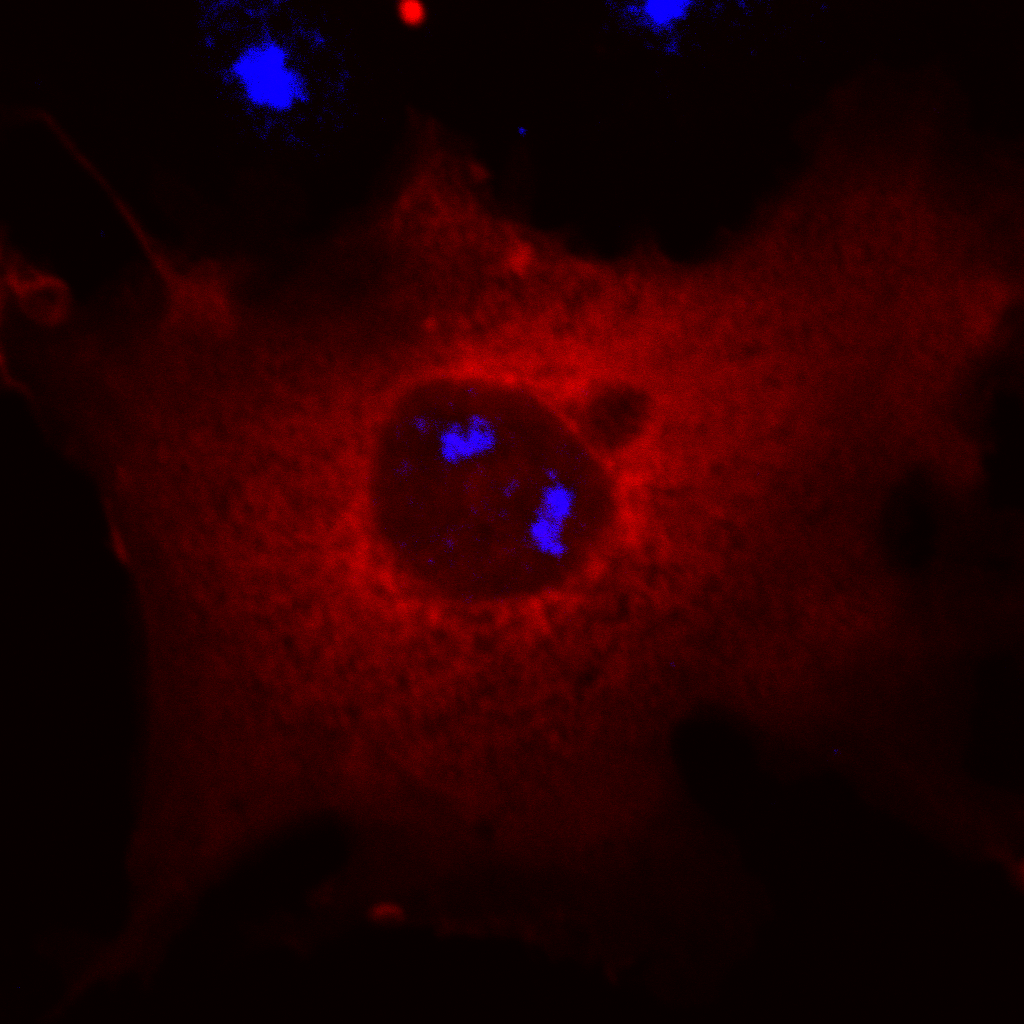

Supplement: Supplementary file 11 — Source data Fig. 10 [file 44318_2024_192_MOESM11_ESM.zip › Figure10/Figure10a/Htt/103Q_Merge.tif]

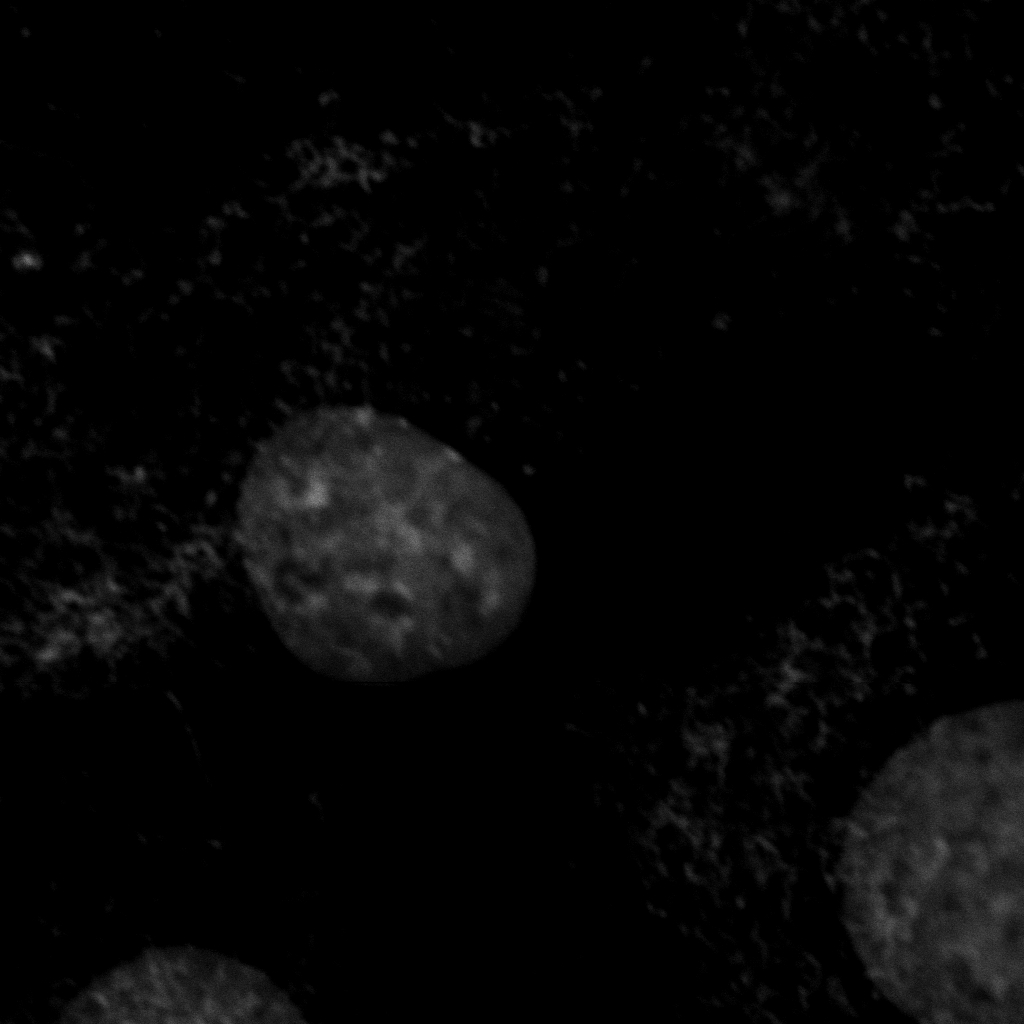

Supplement: Supplementary file 11 — Source data Fig. 10 [file 44318_2024_192_MOESM11_ESM.zip › Figure10/Figure10a/Htt/20Q_DAPI.tif]

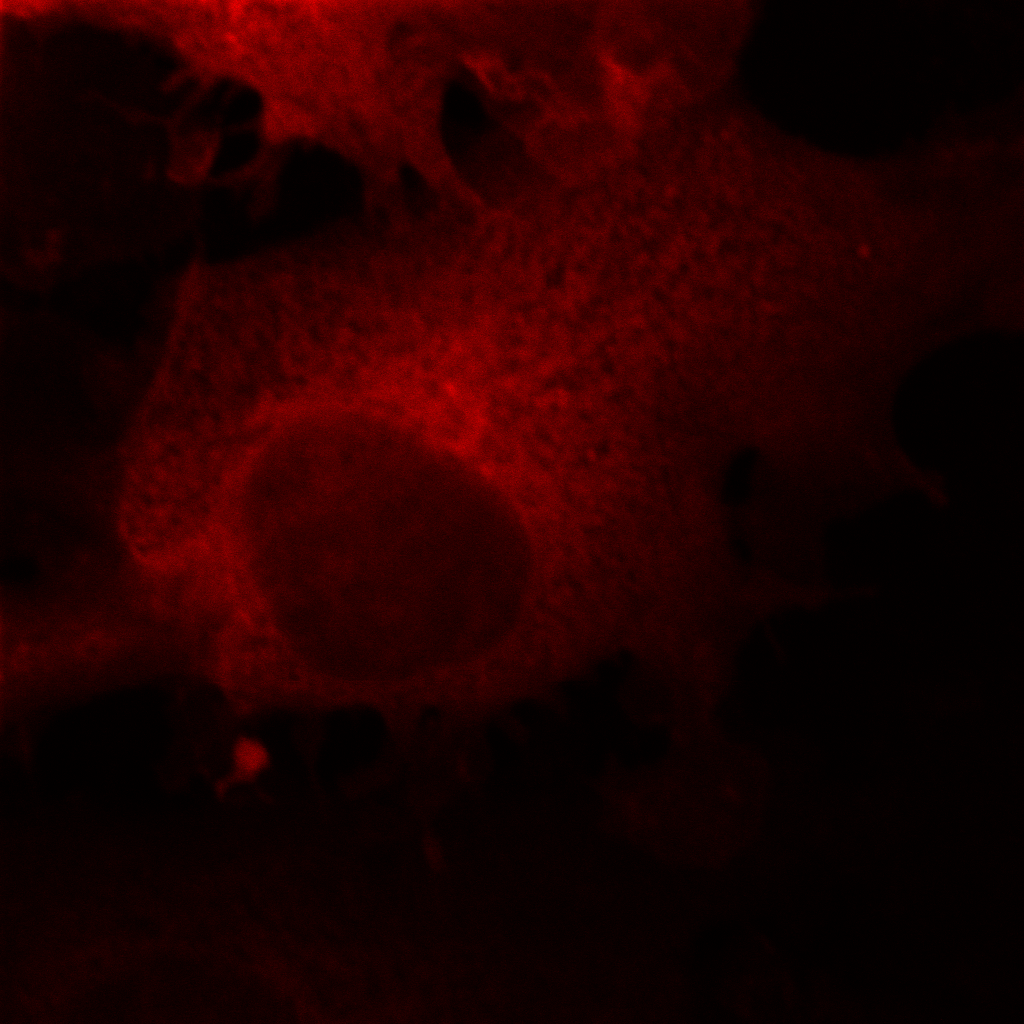

Supplement: Supplementary file 11 — Source data Fig. 10 [file 44318_2024_192_MOESM11_ESM.zip › Figure10/Figure10a/Htt/20Q_DsRed-Htt.tif]

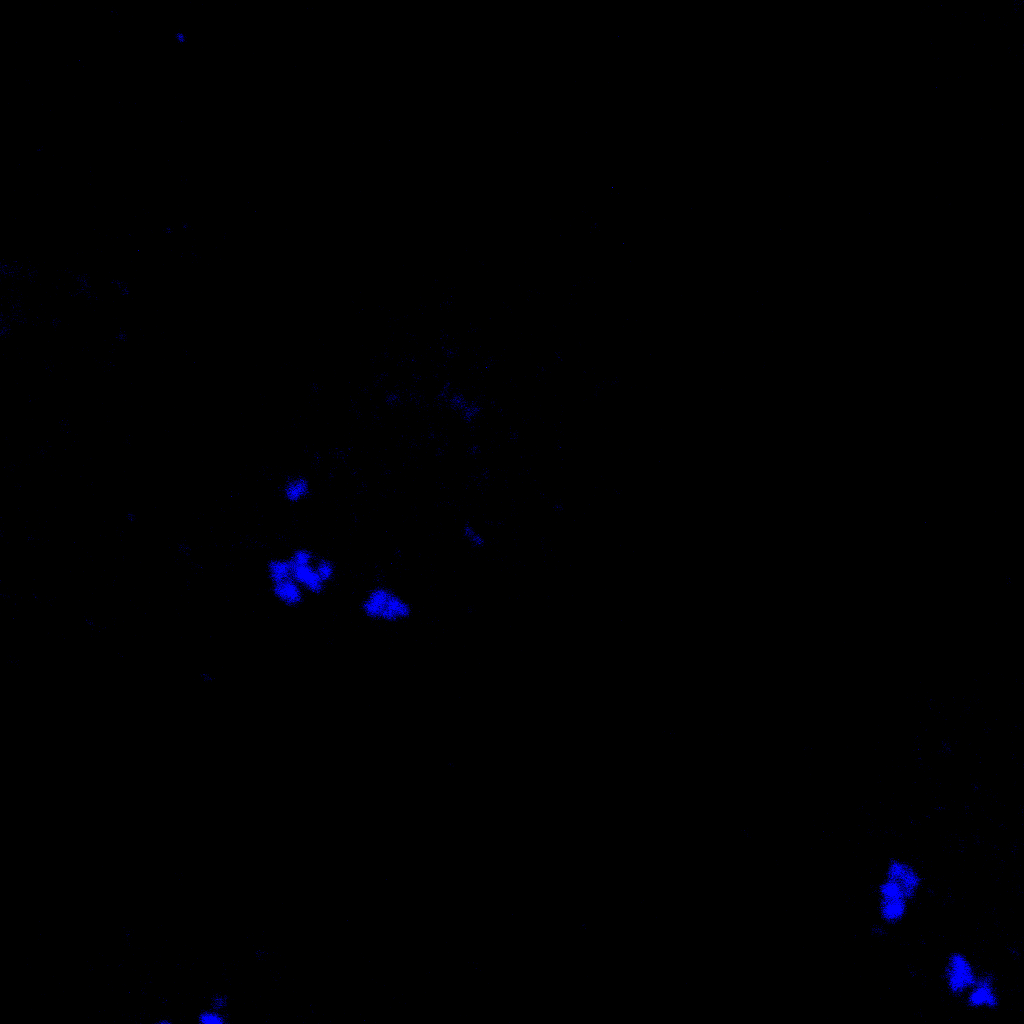

Supplement: Supplementary file 11 — Source data Fig. 10 [file 44318_2024_192_MOESM11_ESM.zip › Figure10/Figure10a/Htt/20Q_Fibrillarin.tif]

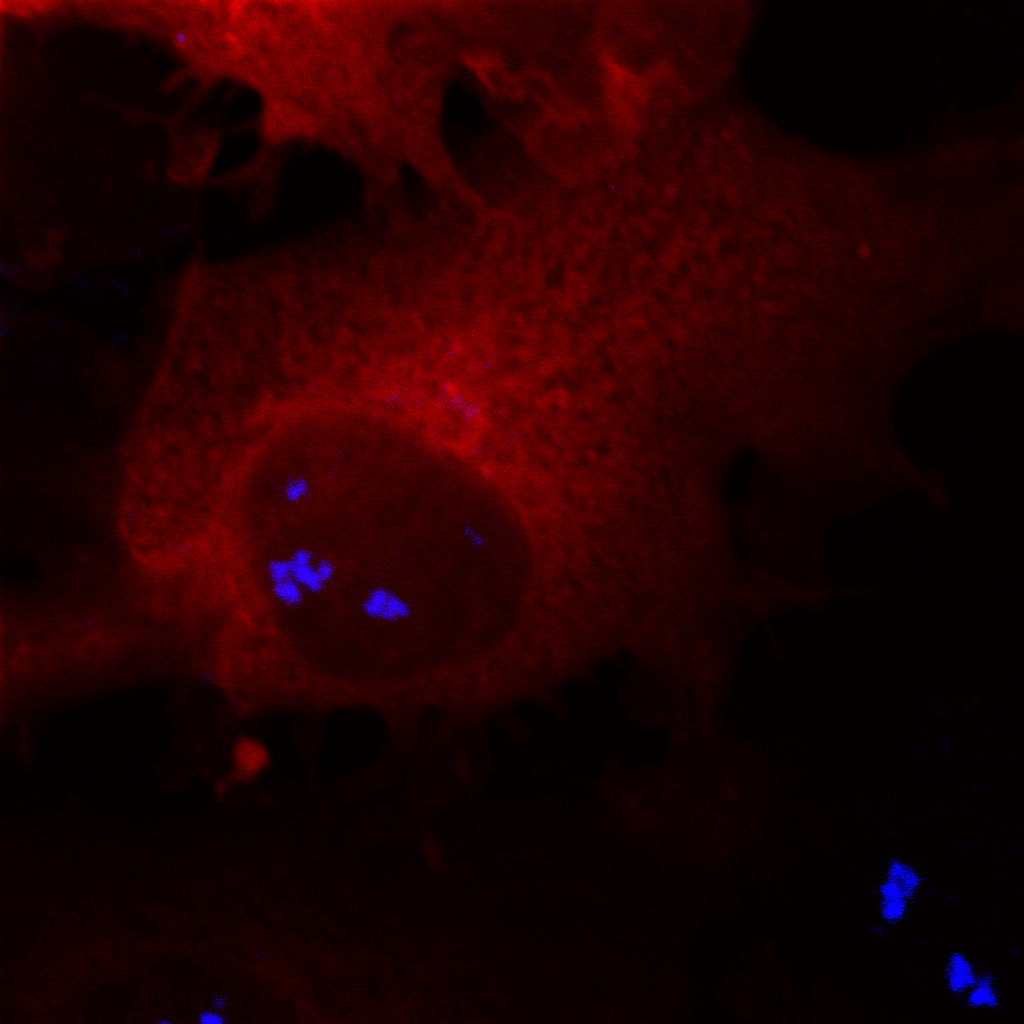

Supplement: Supplementary file 11 — Source data Fig. 10 [file 44318_2024_192_MOESM11_ESM.zip › Figure10/Figure10a/Htt/20Q_Merge.tif]

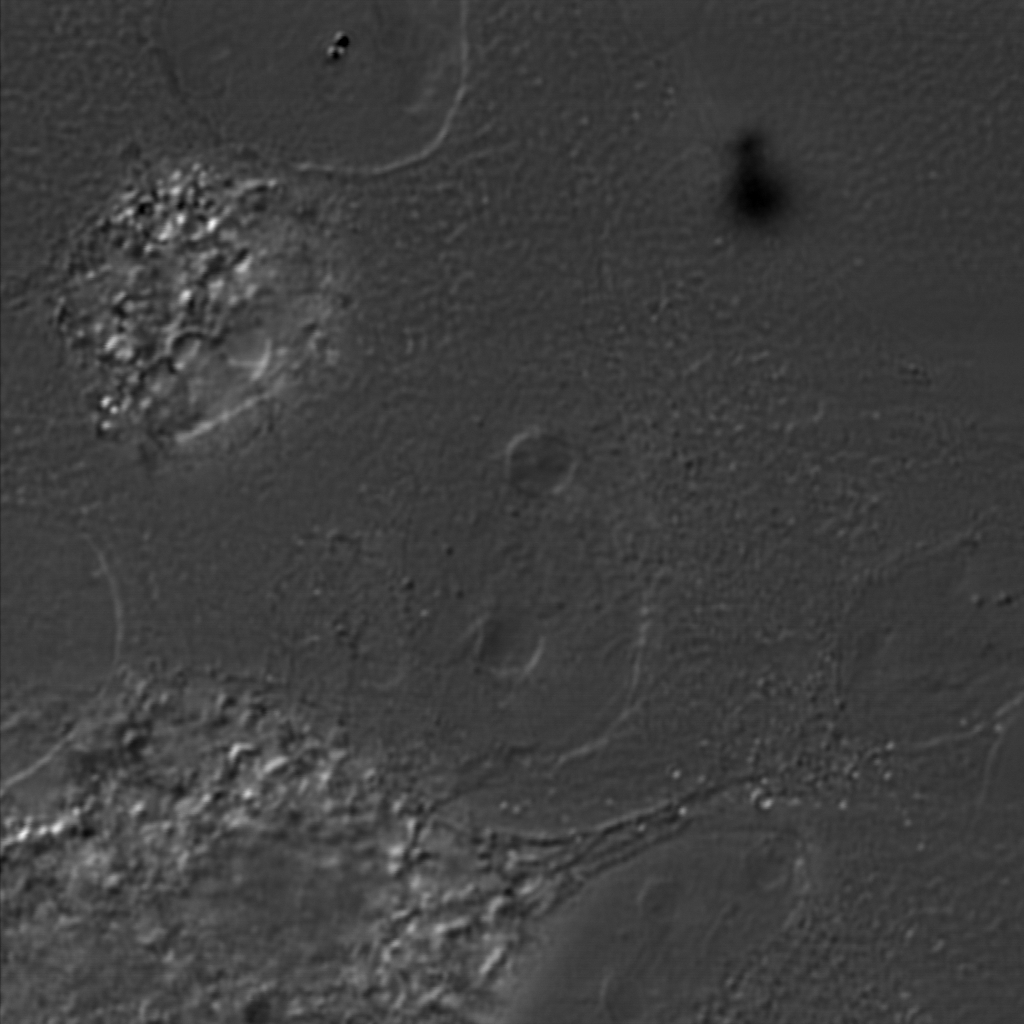

Supplement: Supplementary file 11 — Source data Fig. 10 [file 44318_2024_192_MOESM11_ESM.zip › Figure10/Figure10b/AR/24Q_Bright field.tif]

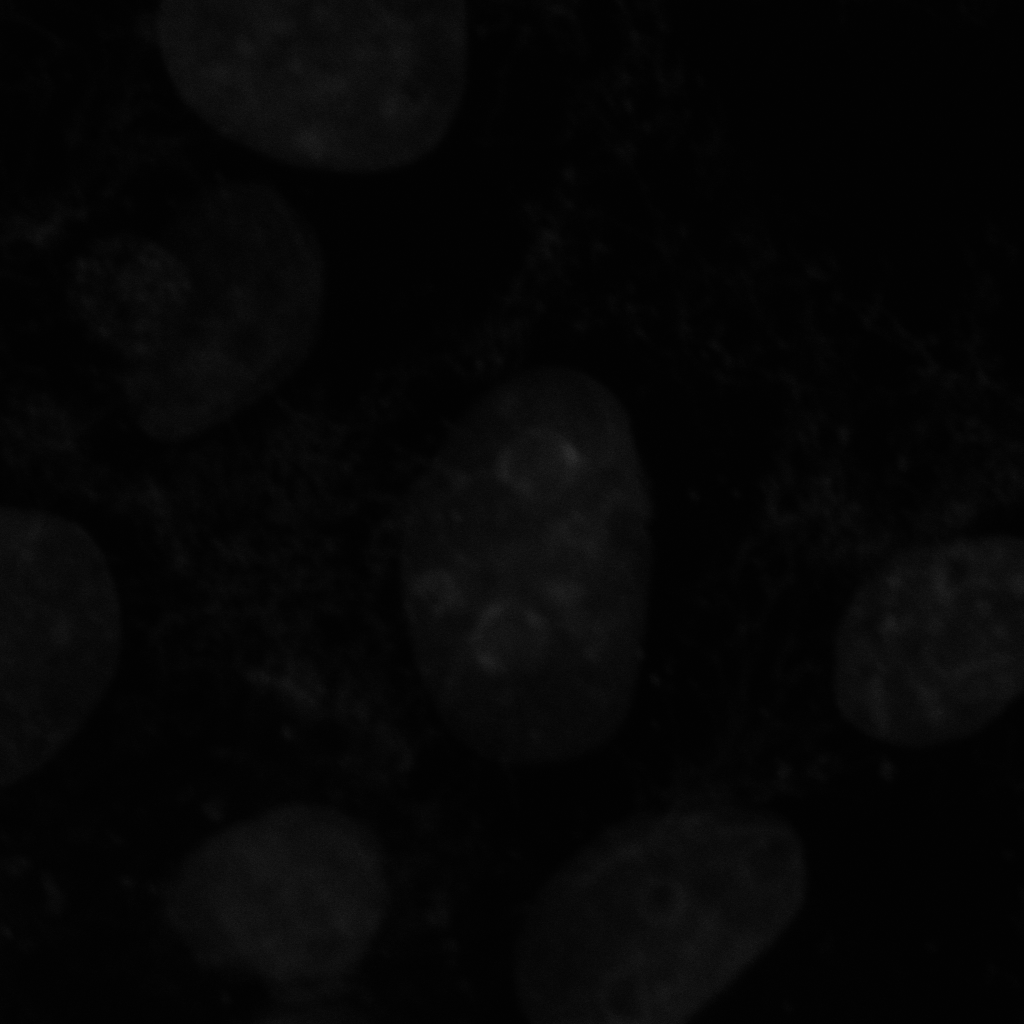

Supplement: Supplementary file 11 — Source data Fig. 10 [file 44318_2024_192_MOESM11_ESM.zip › Figure10/Figure10b/AR/24Q_DAPI.tif]

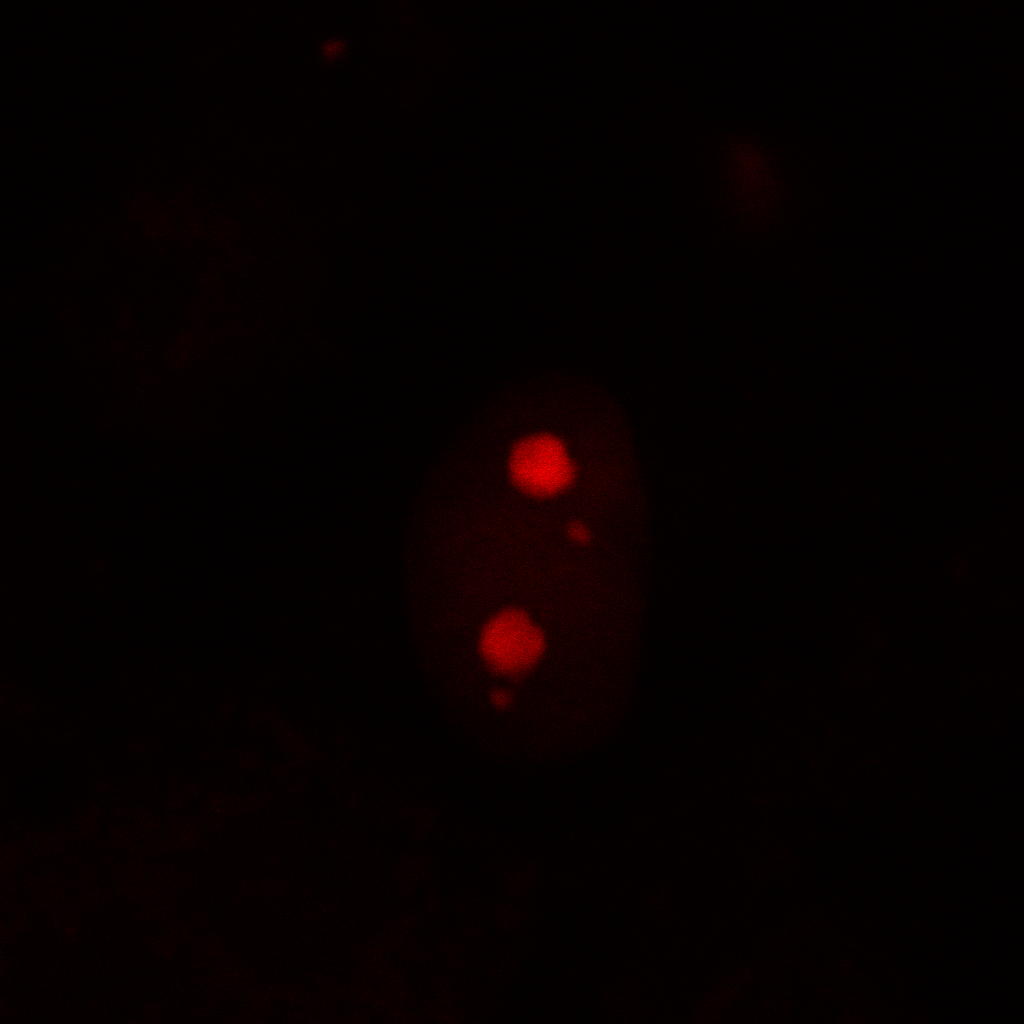

Supplement: Supplementary file 11 — Source data Fig. 10 [file 44318_2024_192_MOESM11_ESM.zip › Figure10/Figure10b/AR/24Q_DsRed-AR.tif]

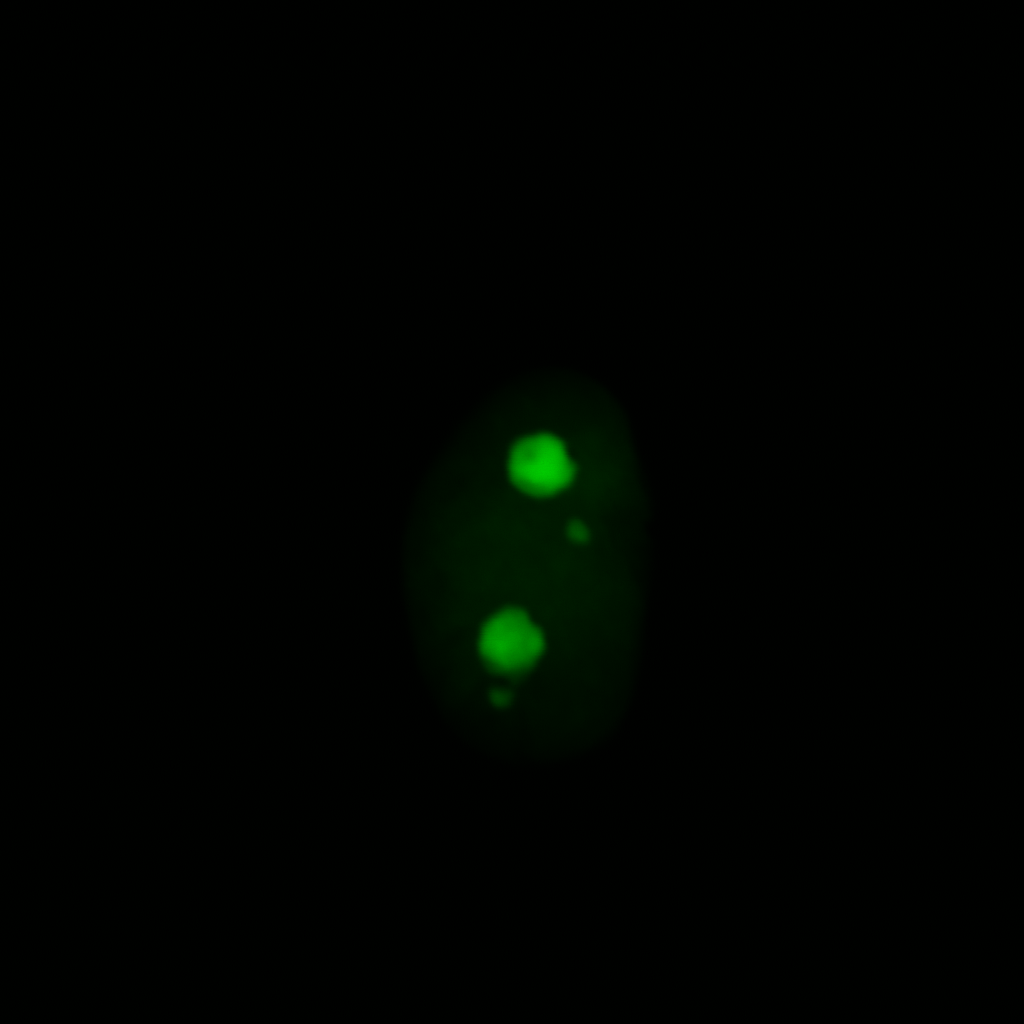

Supplement: Supplementary file 11 — Source data Fig. 10 [file 44318_2024_192_MOESM11_ESM.zip › Figure10/Figure10b/AR/24Q_EGFP-PQBP3.tif]

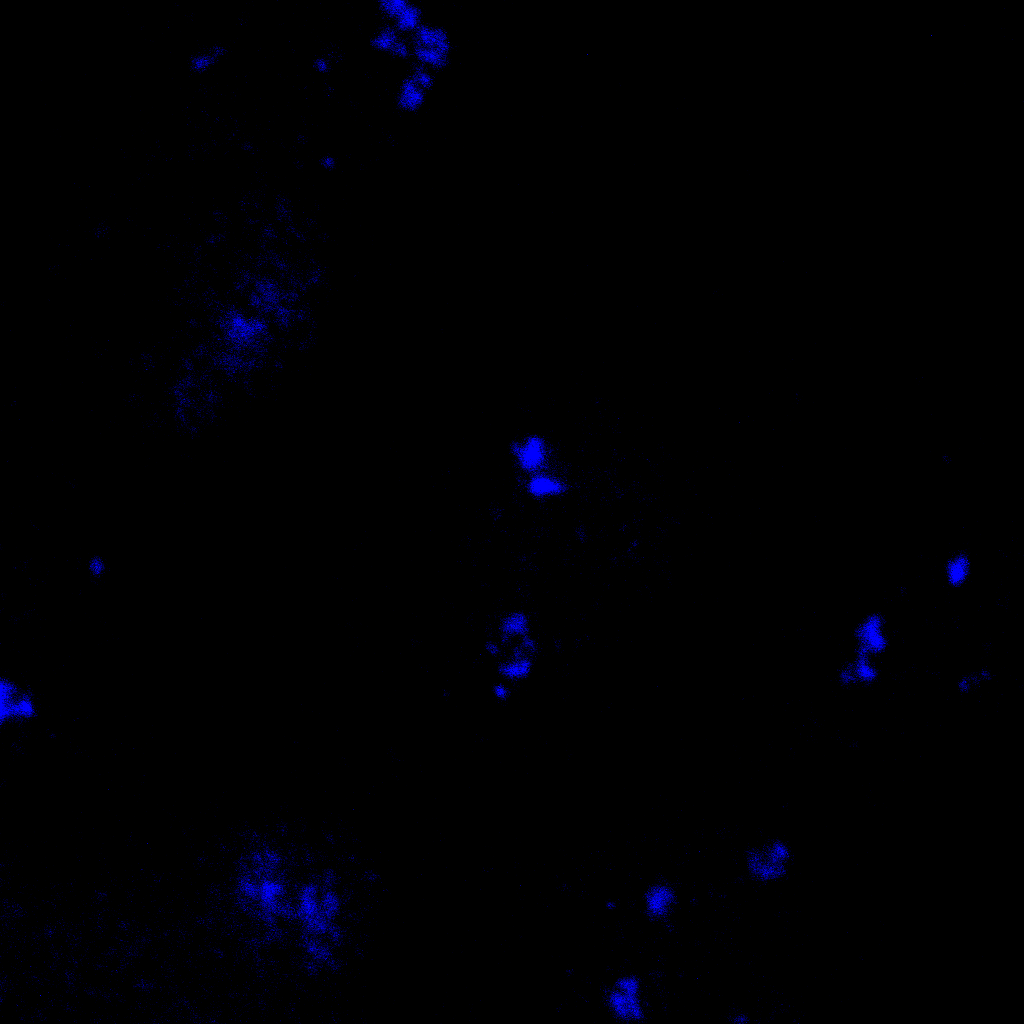

Supplement: Supplementary file 11 — Source data Fig. 10 [file 44318_2024_192_MOESM11_ESM.zip › Figure10/Figure10b/AR/24Q_Fibrillarin.tif]

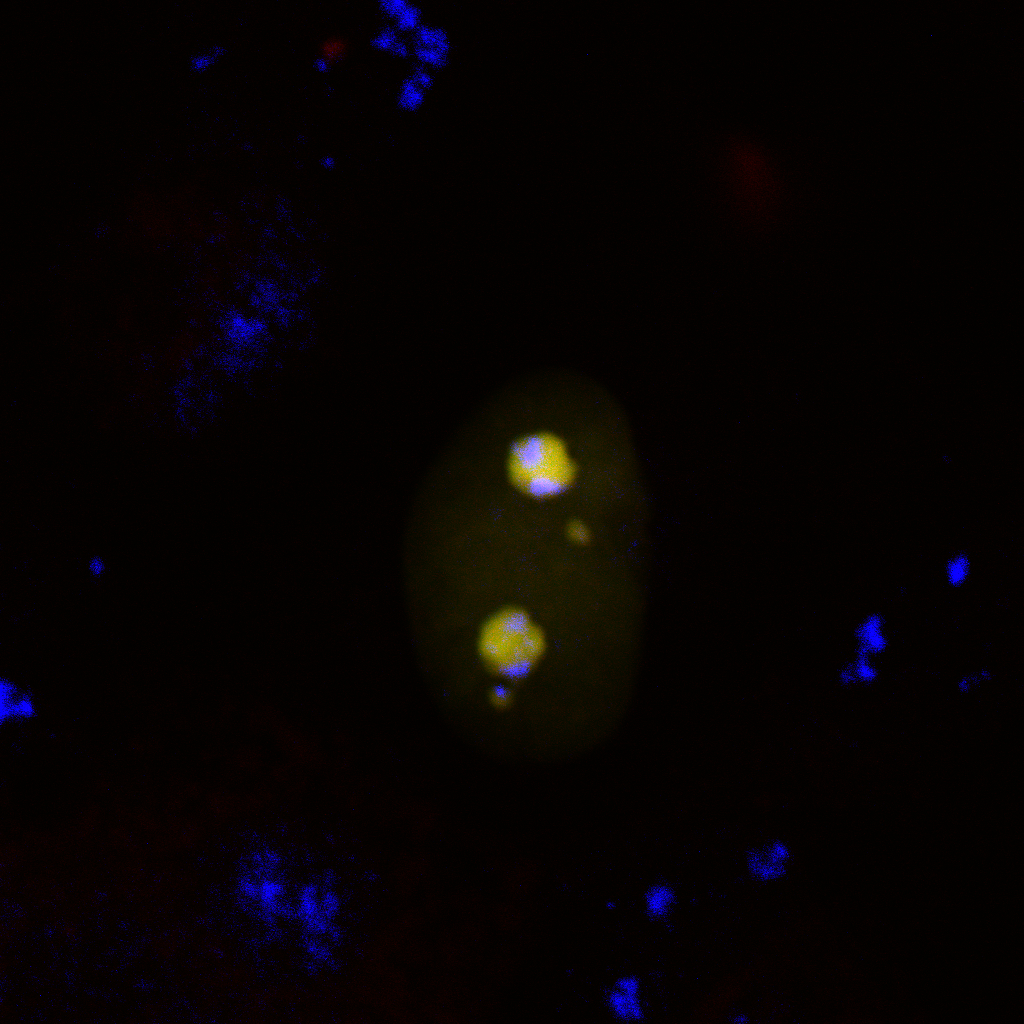

Supplement: Supplementary file 11 — Source data Fig. 10 [file 44318_2024_192_MOESM11_ESM.zip › Figure10/Figure10b/AR/24Q_Merge.tif]

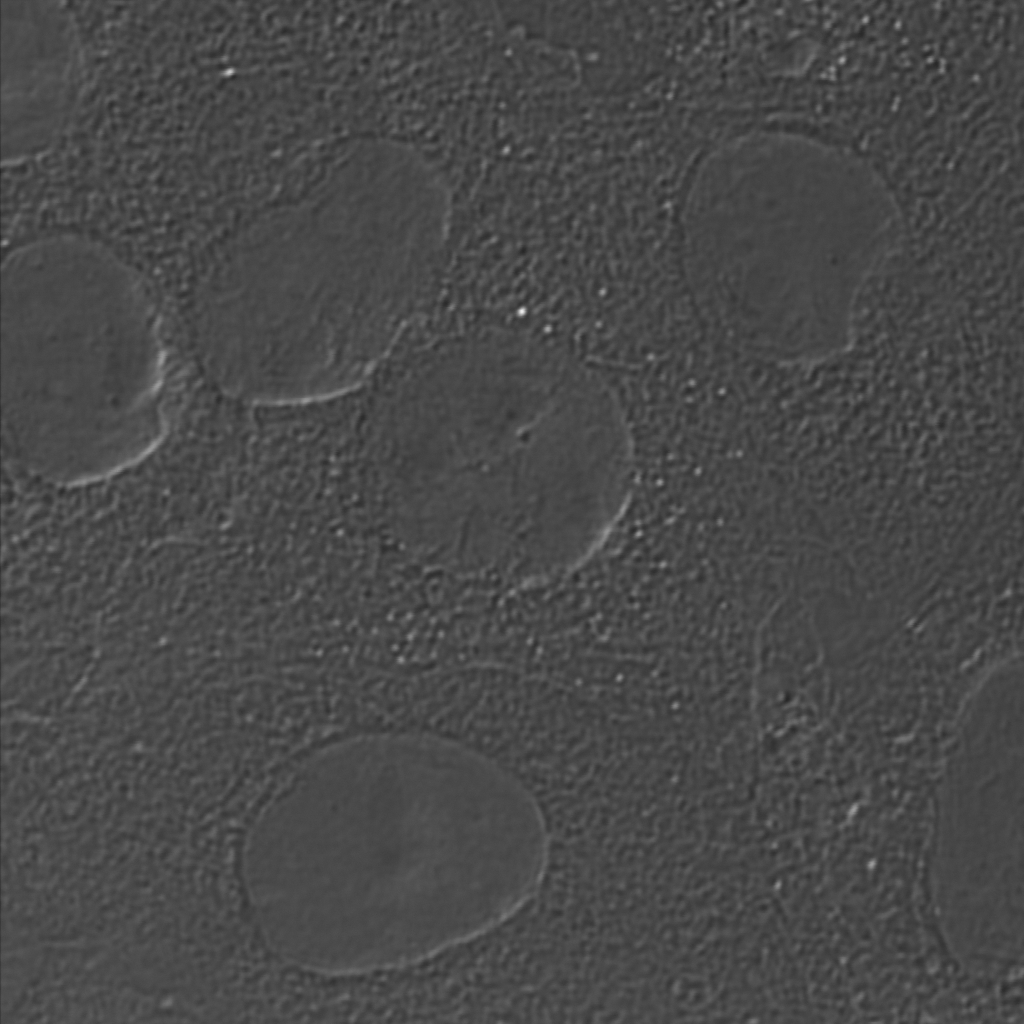

Supplement: Supplementary file 11 — Source data Fig. 10 [file 44318_2024_192_MOESM11_ESM.zip › Figure10/Figure10b/AR/64Q_Bright field.tif]

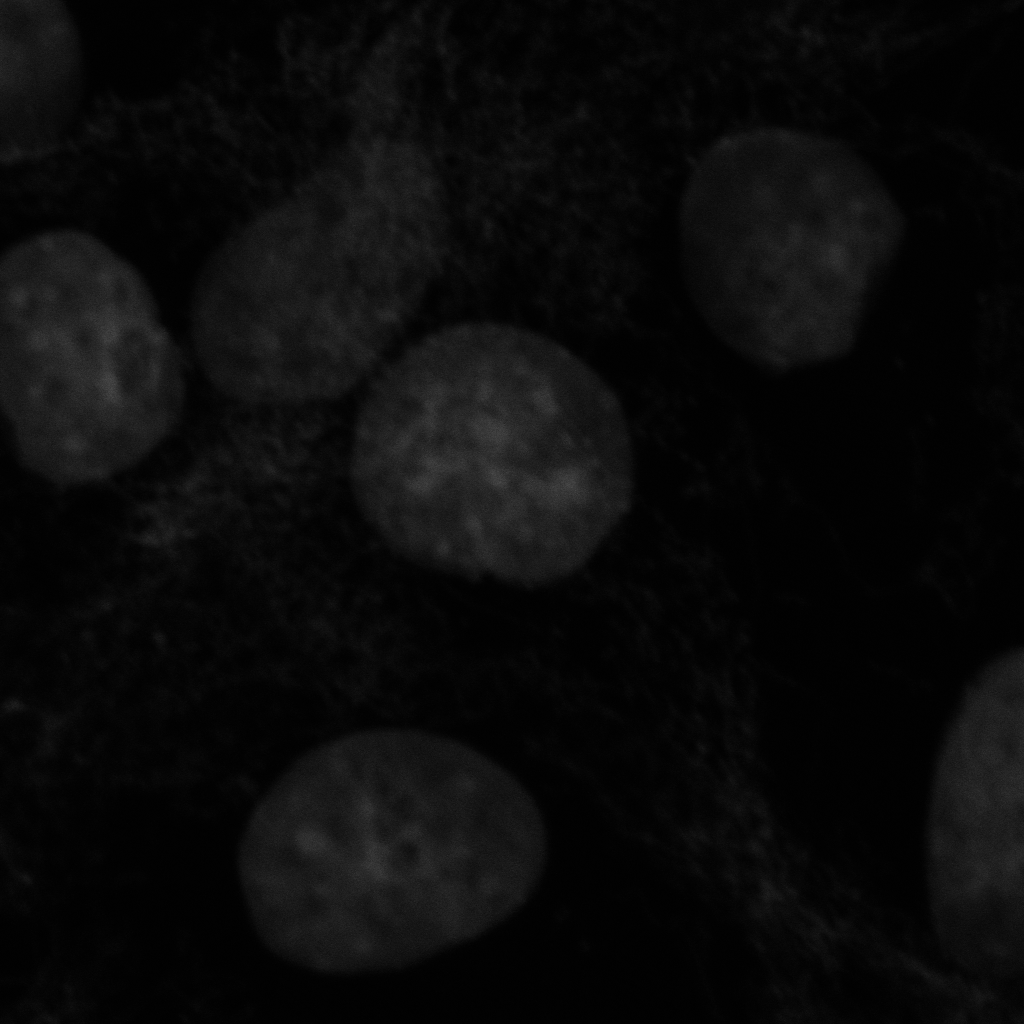

Supplement: Supplementary file 11 — Source data Fig. 10 [file 44318_2024_192_MOESM11_ESM.zip › Figure10/Figure10b/AR/64Q_DAPI.tif]

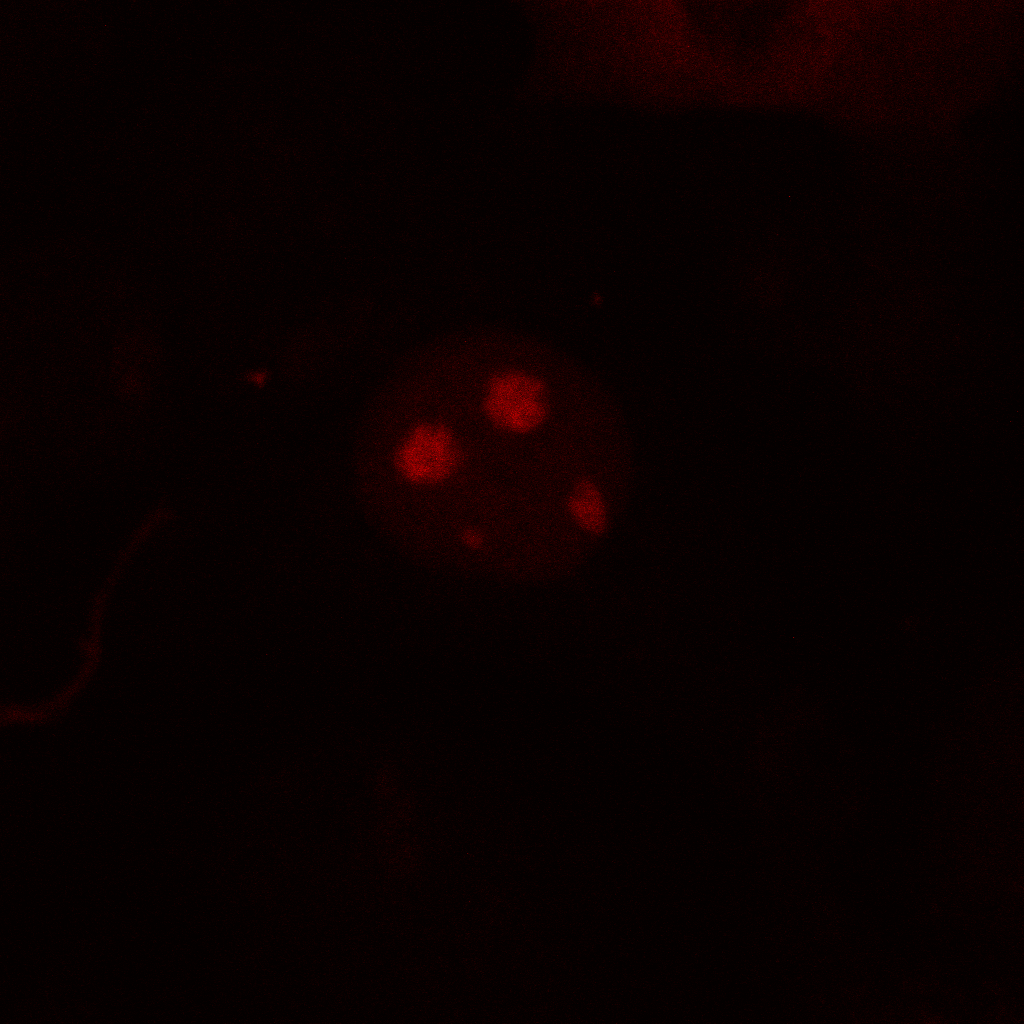

Supplement: Supplementary file 11 — Source data Fig. 10 [file 44318_2024_192_MOESM11_ESM.zip › Figure10/Figure10b/AR/64Q_DsRed-AR.tif]

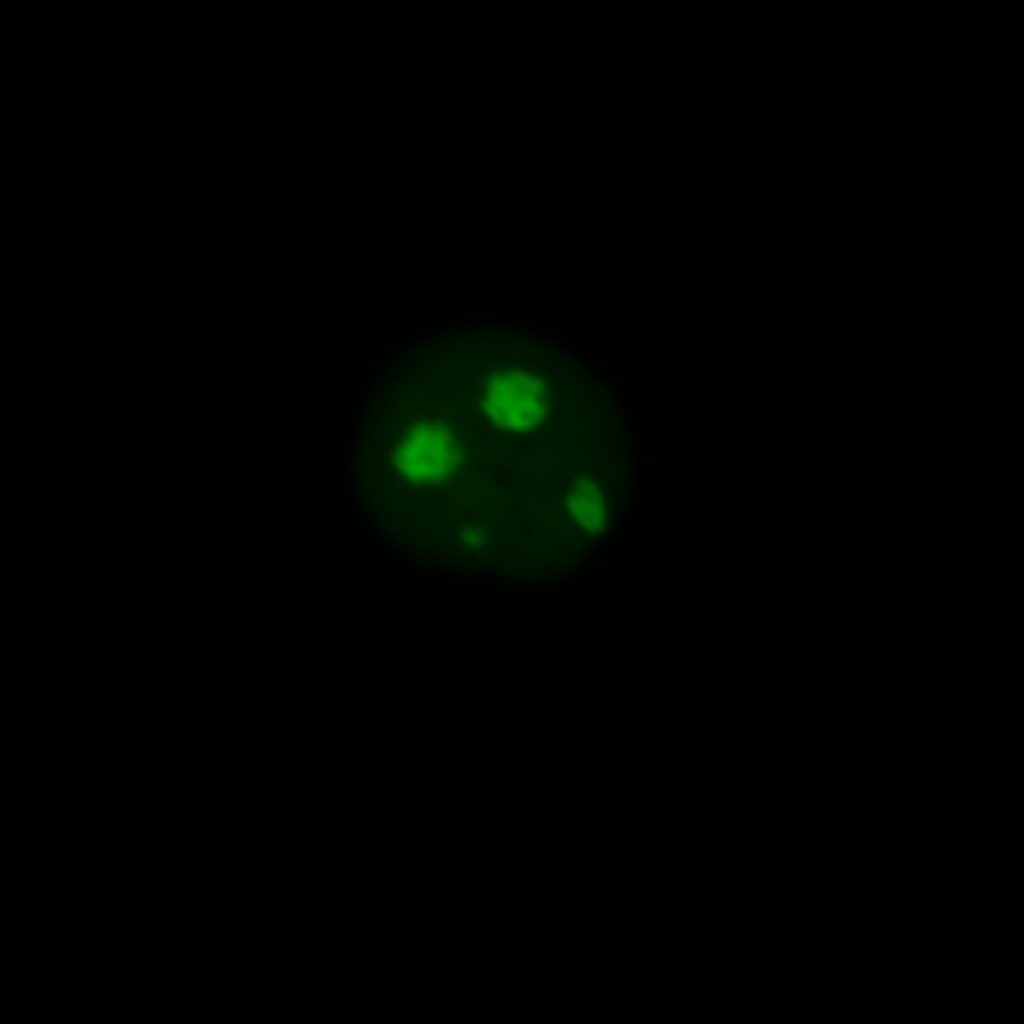

Supplement: Supplementary file 11 — Source data Fig. 10 [file 44318_2024_192_MOESM11_ESM.zip › Figure10/Figure10b/AR/64Q_EGFP-PQBP3.tif]

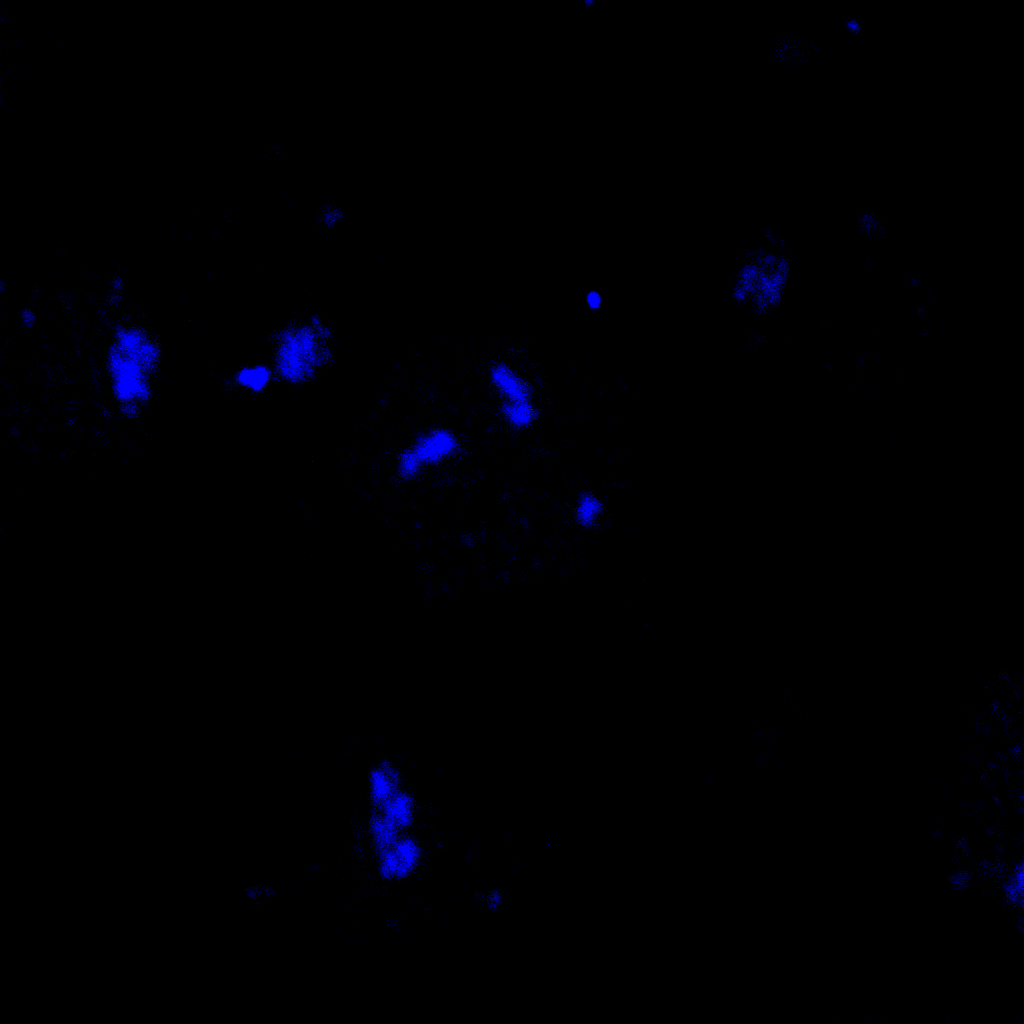

Supplement: Supplementary file 11 — Source data Fig. 10 [file 44318_2024_192_MOESM11_ESM.zip › Figure10/Figure10b/AR/64Q_Fibrillarin.tif]

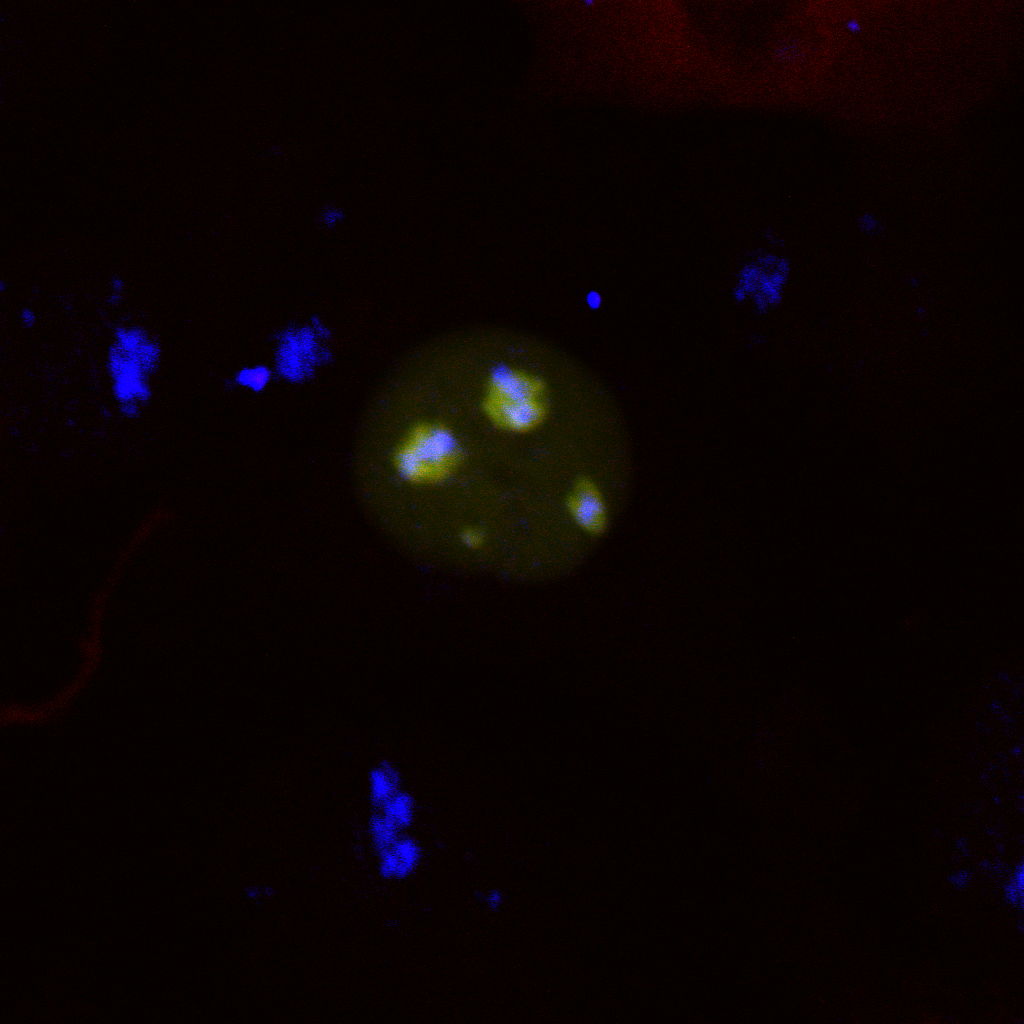

Supplement: Supplementary file 11 — Source data Fig. 10 [file 44318_2024_192_MOESM11_ESM.zip › Figure10/Figure10b/AR/64Q_Merge.tif]

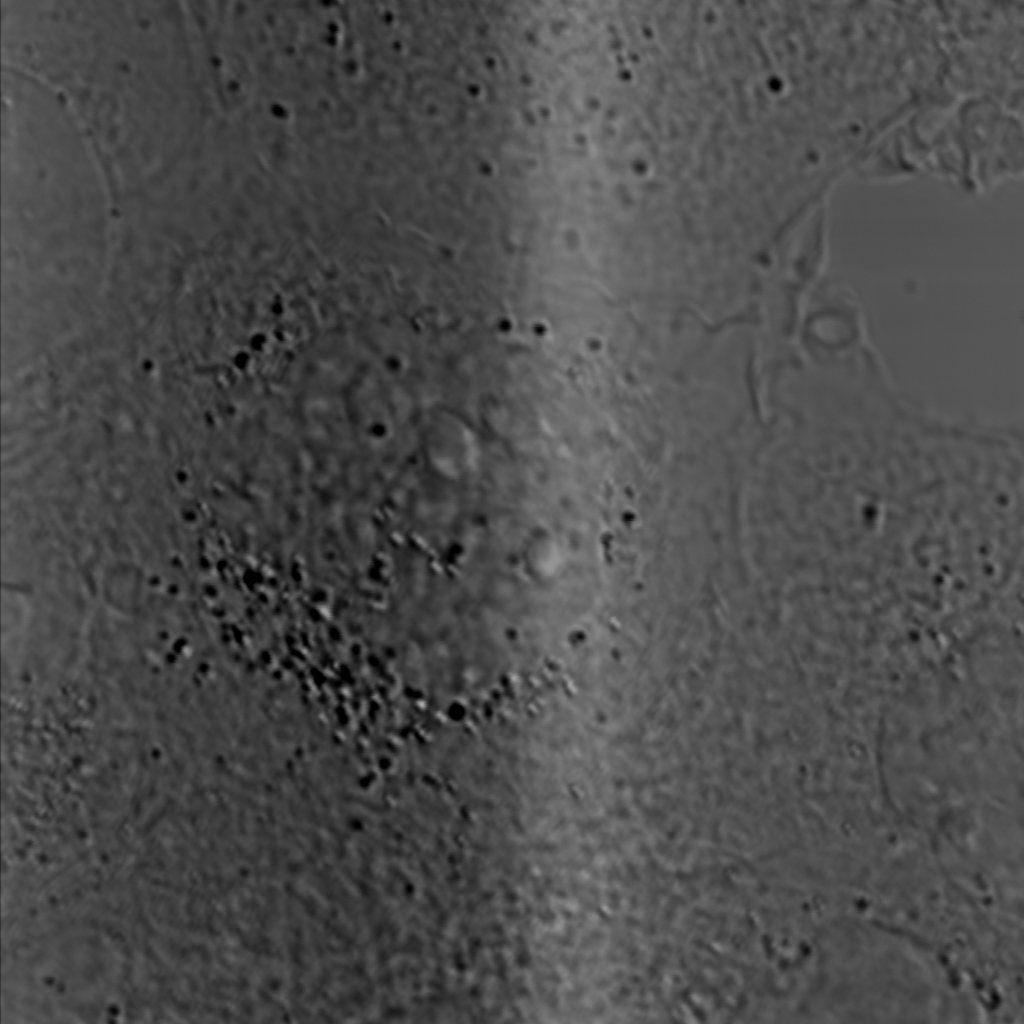

Supplement: Supplementary file 11 — Source data Fig. 10 [file 44318_2024_192_MOESM11_ESM.zip › Figure10/Figure10b/Atxn1/33Q_Bright field.tif]

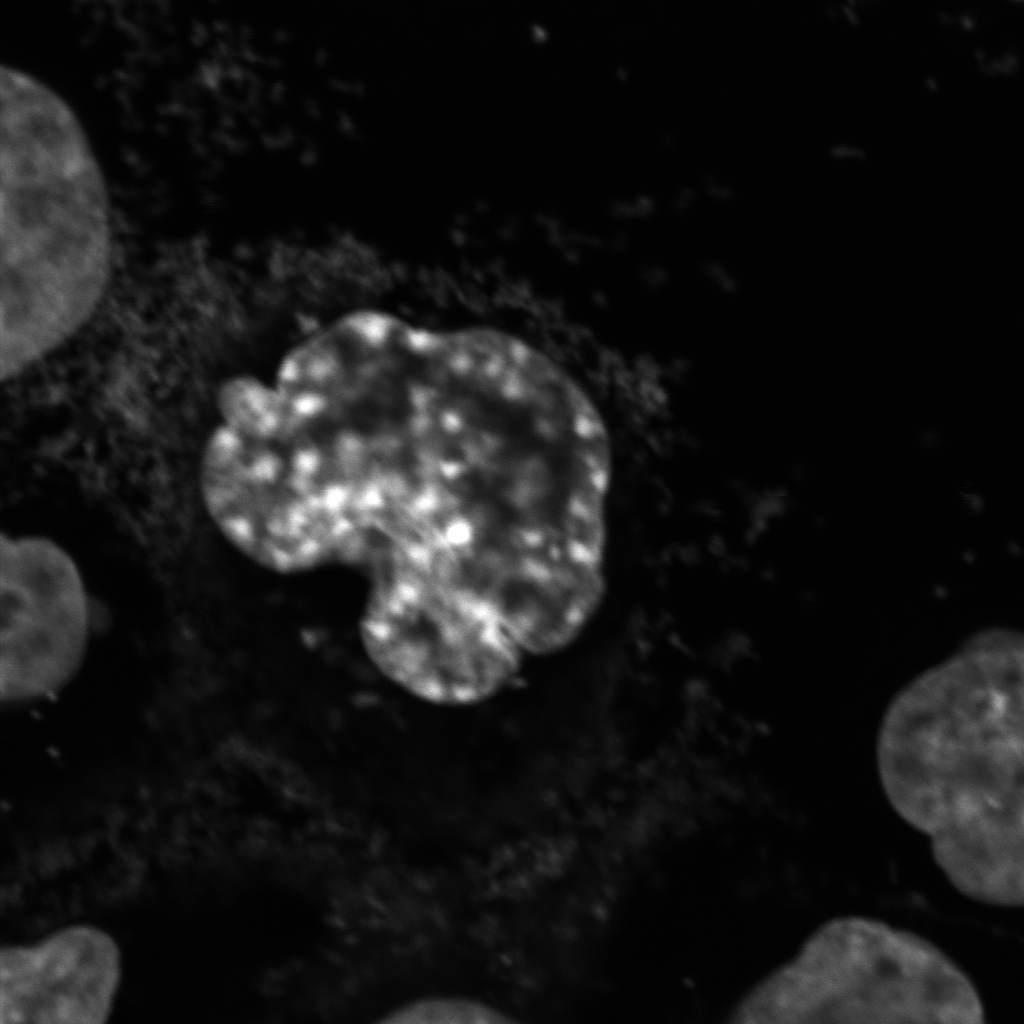

Supplement: Supplementary file 11 — Source data Fig. 10 [file 44318_2024_192_MOESM11_ESM.zip › Figure10/Figure10b/Atxn1/33Q_DAPI.tif]

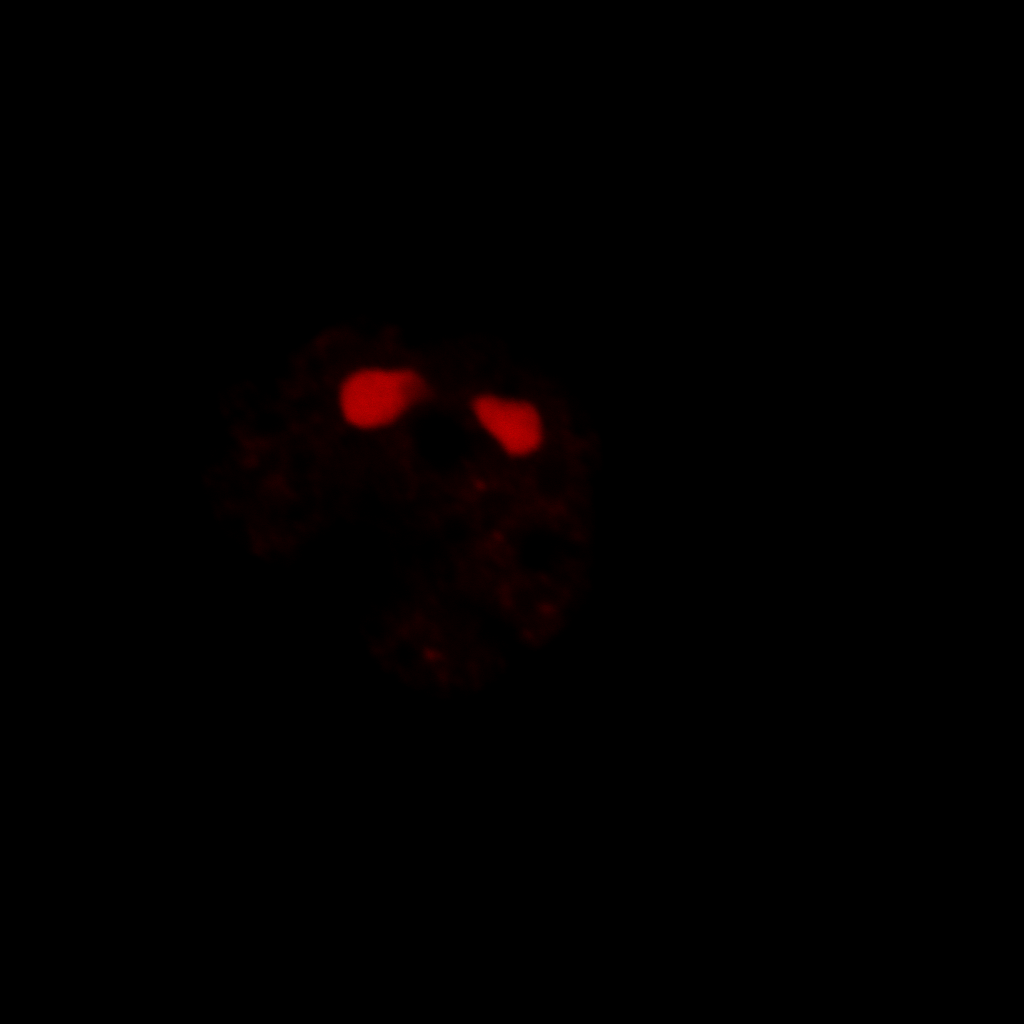

Supplement: Supplementary file 11 — Source data Fig. 10 [file 44318_2024_192_MOESM11_ESM.zip › Figure10/Figure10b/Atxn1/33Q_DsRed-Atxn1.tif]

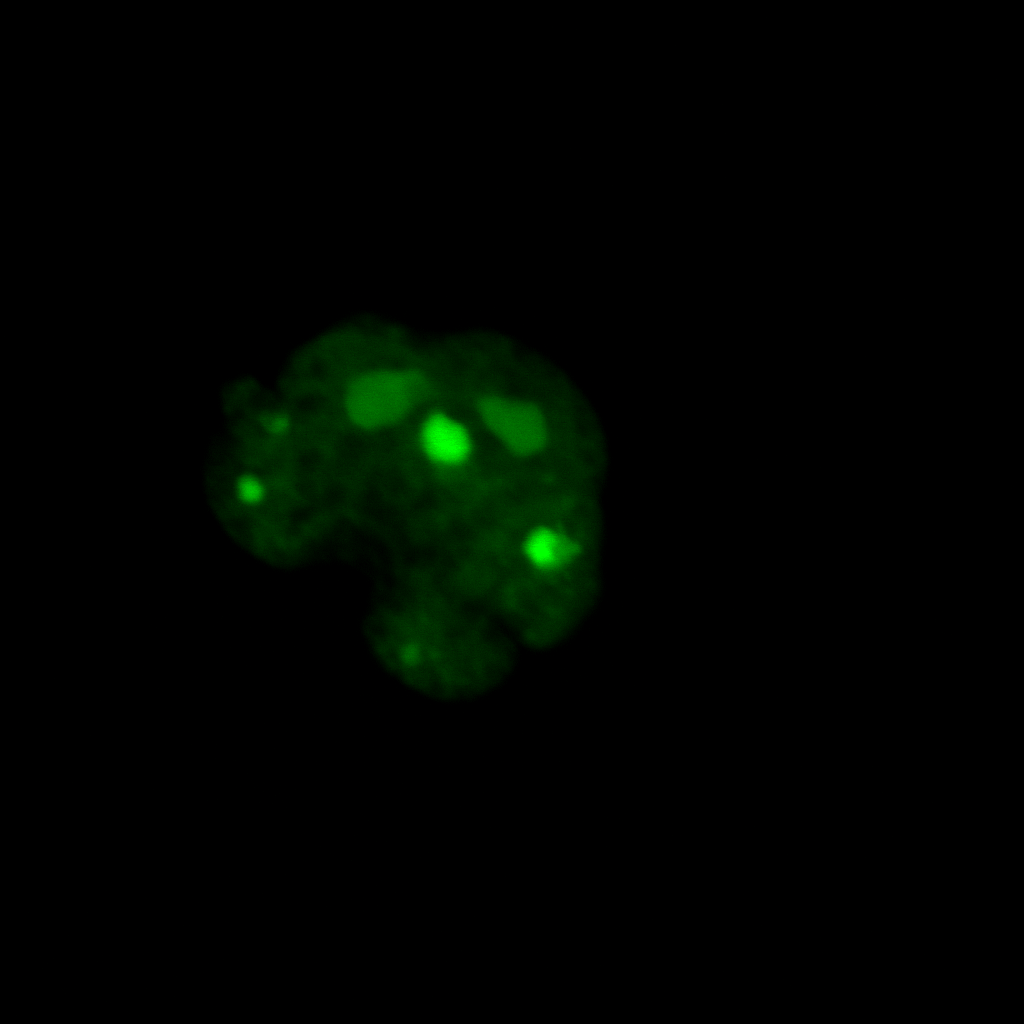

Supplement: Supplementary file 11 — Source data Fig. 10 [file 44318_2024_192_MOESM11_ESM.zip › Figure10/Figure10b/Atxn1/33Q_EGFP-PQBP3.tif]

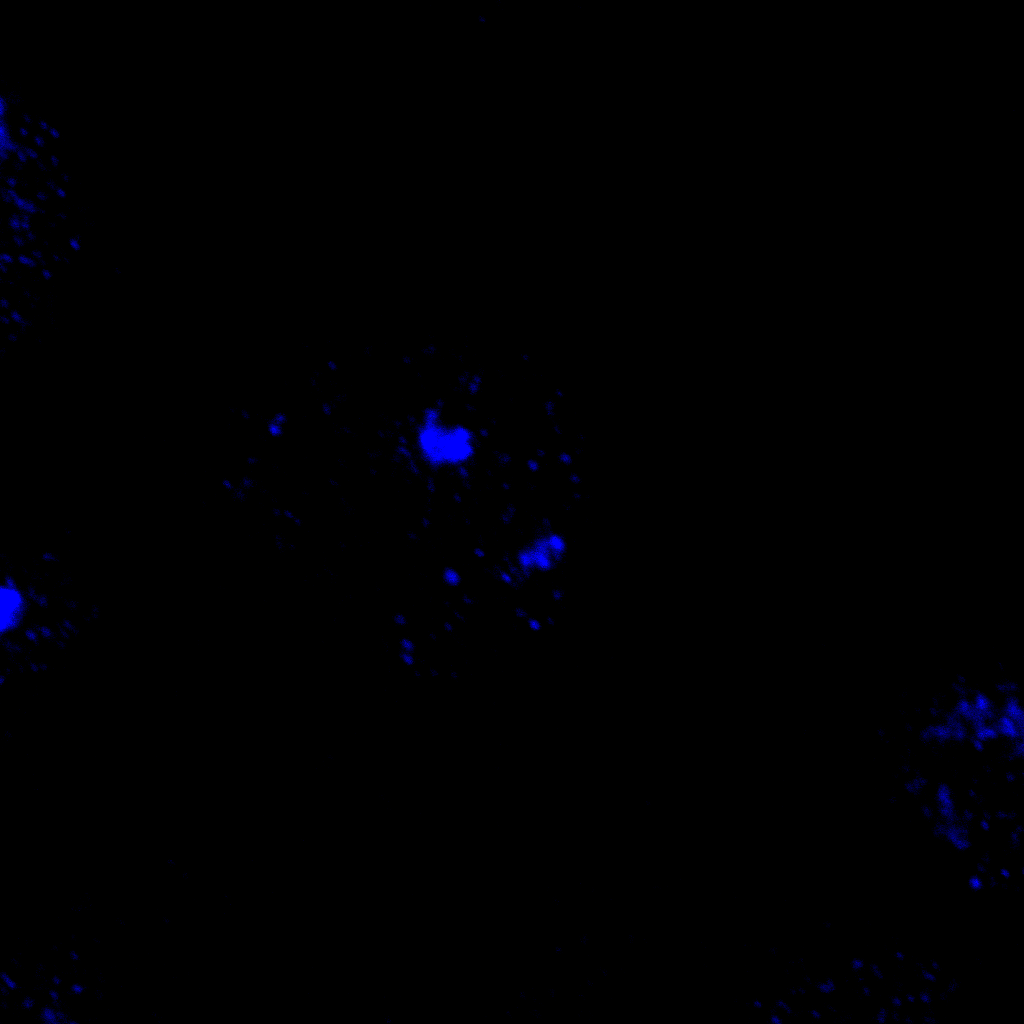

Supplement: Supplementary file 11 — Source data Fig. 10 [file 44318_2024_192_MOESM11_ESM.zip › Figure10/Figure10b/Atxn1/33Q_Fibrillarin.tif]

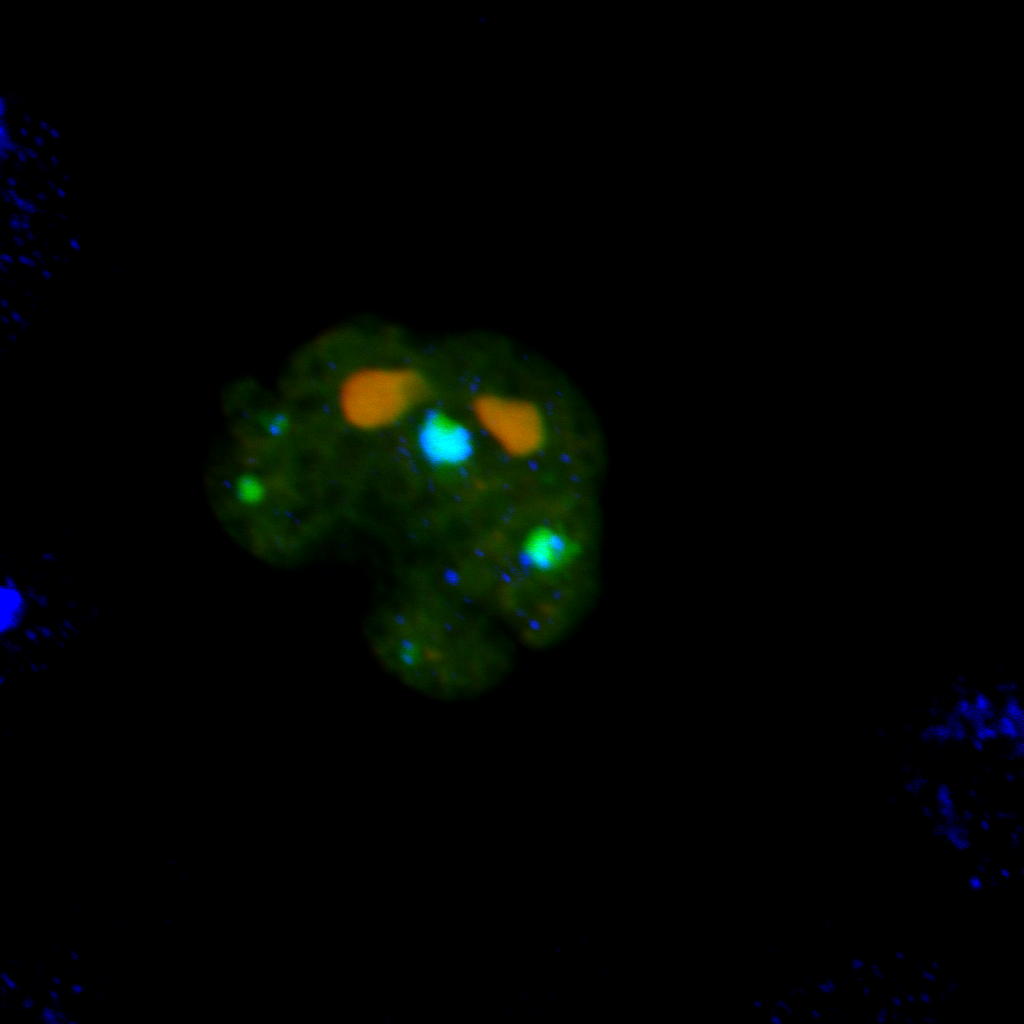

Supplement: Supplementary file 11 — Source data Fig. 10 [file 44318_2024_192_MOESM11_ESM.zip › Figure10/Figure10b/Atxn1/33Q_Merge.tif]

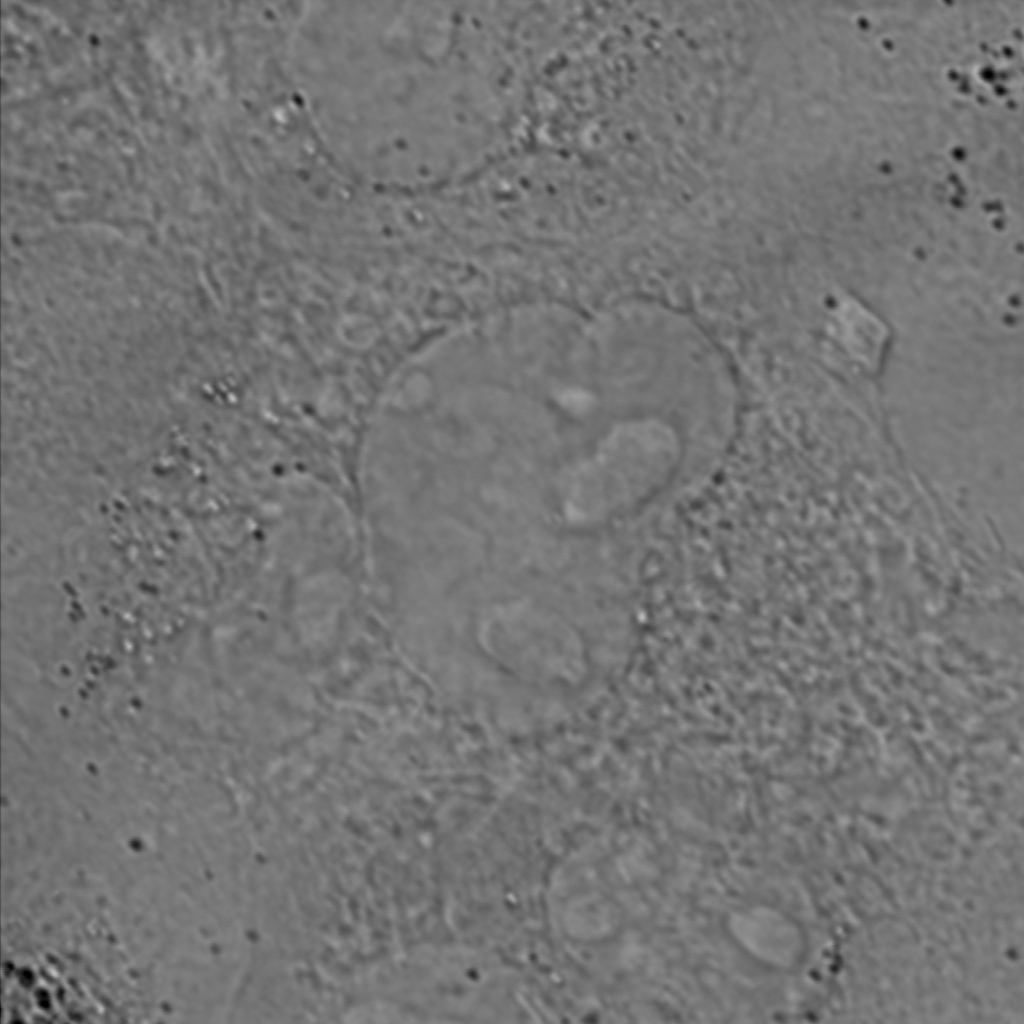

Supplement: Supplementary file 11 — Source data Fig. 10 [file 44318_2024_192_MOESM11_ESM.zip › Figure10/Figure10b/Atxn1/86Q_Bright field.tif]

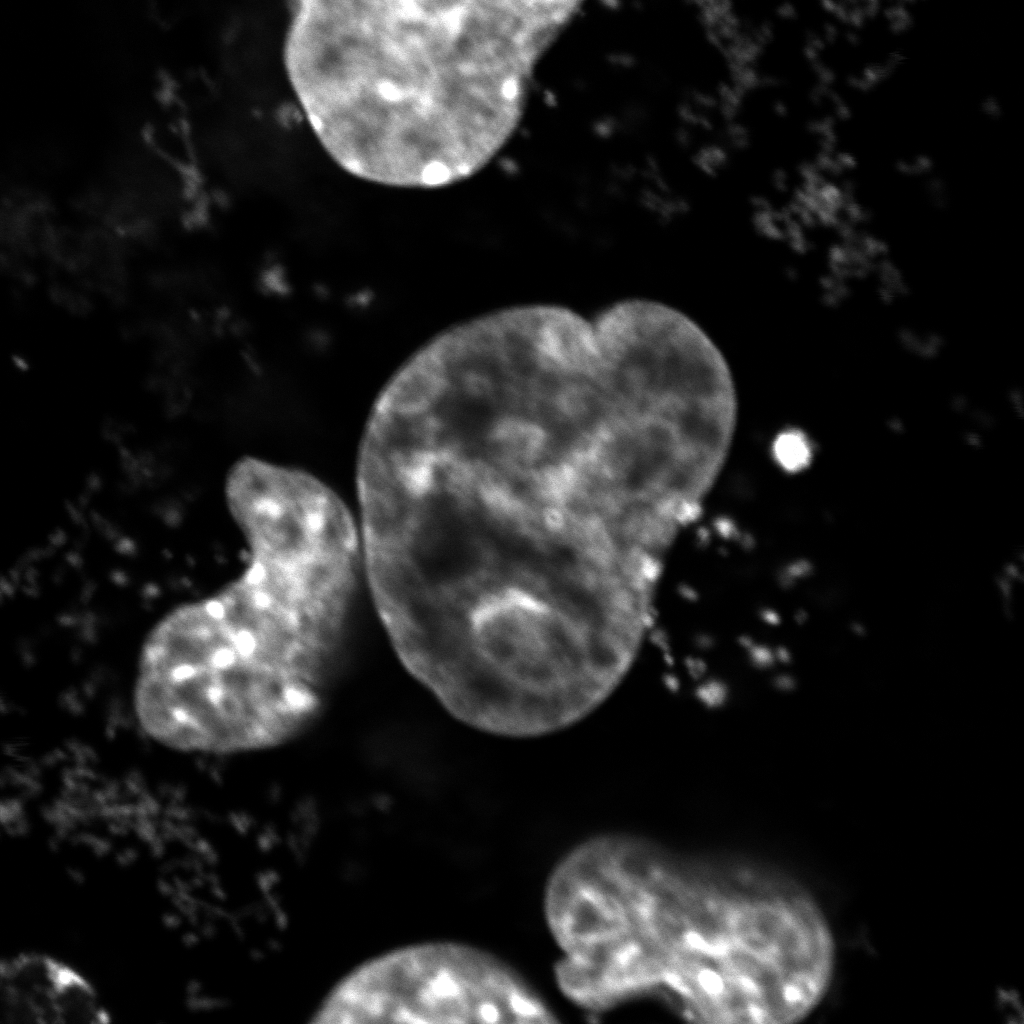

Supplement: Supplementary file 11 — Source data Fig. 10 [file 44318_2024_192_MOESM11_ESM.zip › Figure10/Figure10b/Atxn1/86Q_DAPI.tif]

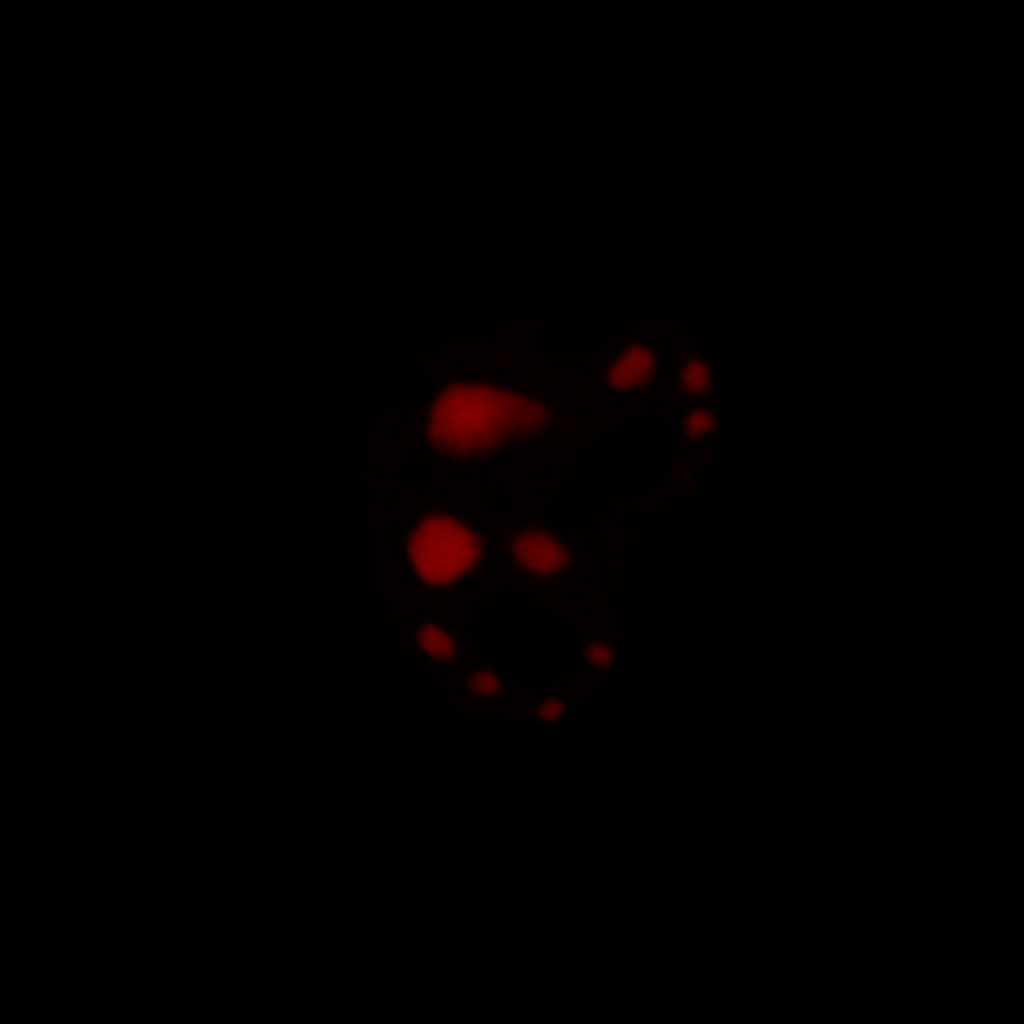

Supplement: Supplementary file 11 — Source data Fig. 10 [file 44318_2024_192_MOESM11_ESM.zip › Figure10/Figure10b/Atxn1/86Q_DsRed-Atxn1.tif]

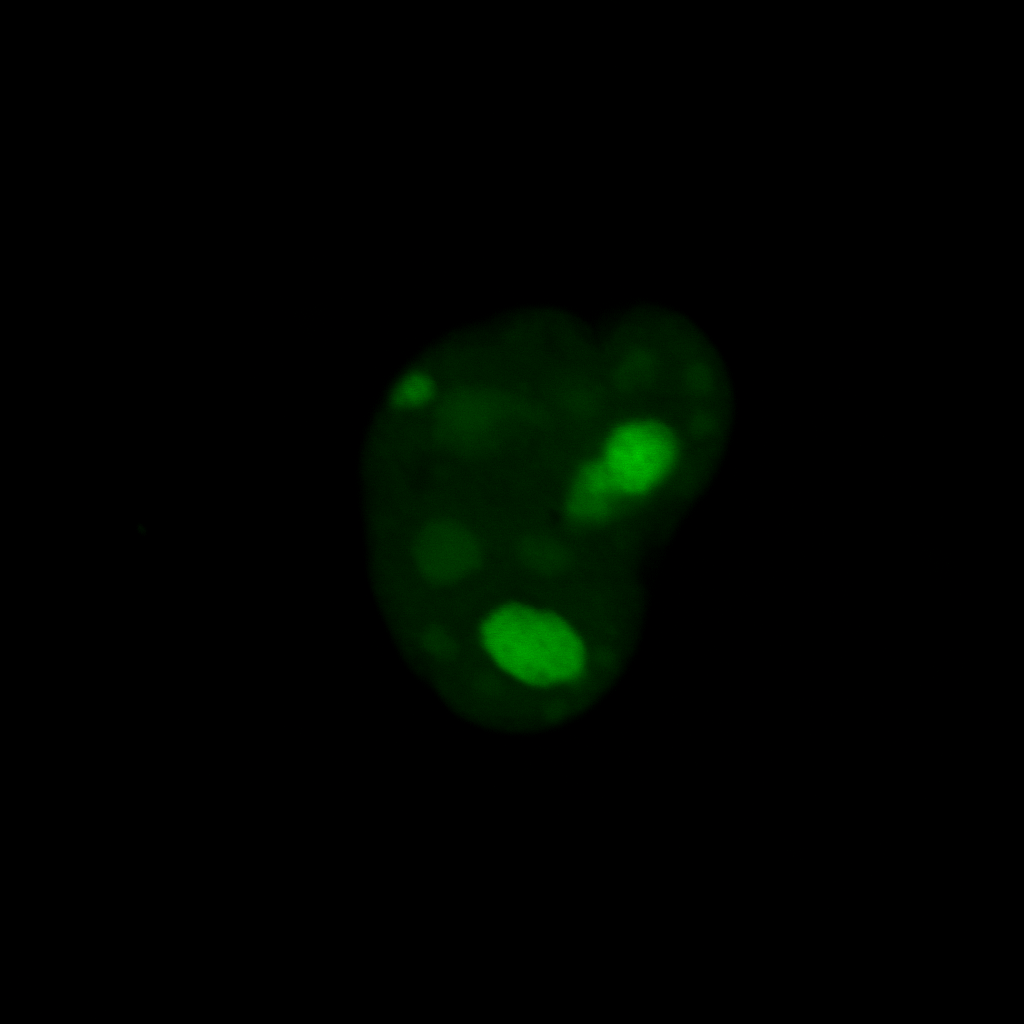

Supplement: Supplementary file 11 — Source data Fig. 10 [file 44318_2024_192_MOESM11_ESM.zip › Figure10/Figure10b/Atxn1/86Q_EGFP-PQBP3.tif]

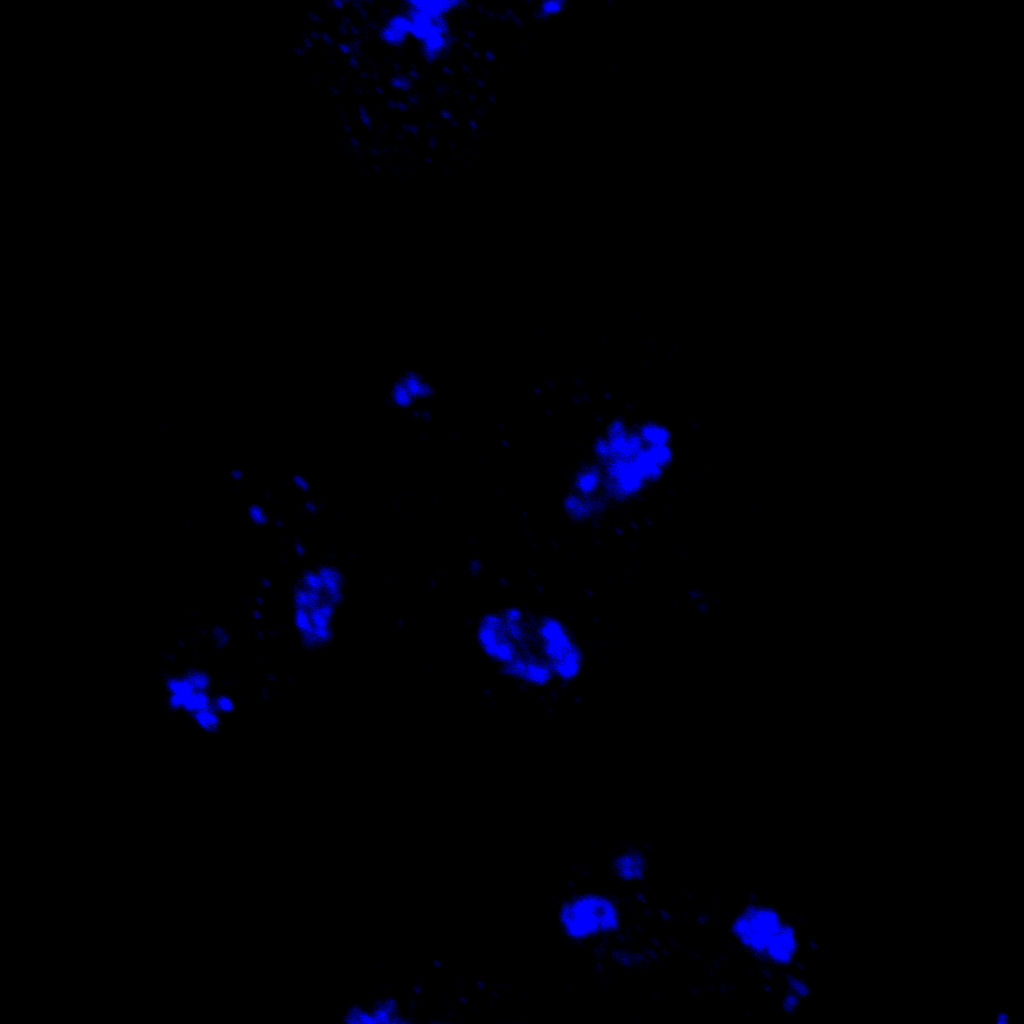

Supplement: Supplementary file 11 — Source data Fig. 10 [file 44318_2024_192_MOESM11_ESM.zip › Figure10/Figure10b/Atxn1/86Q_Fibrillarin.tif]

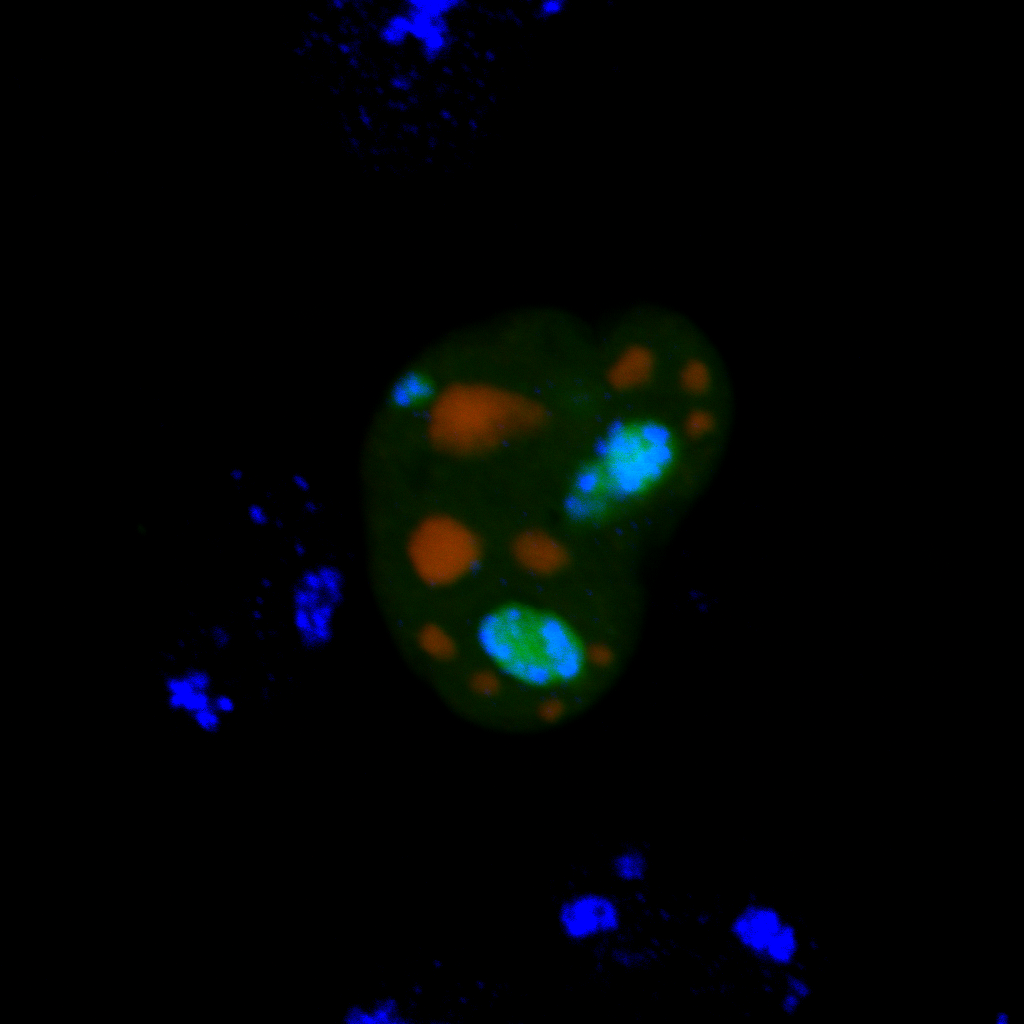

Supplement: Supplementary file 11 — Source data Fig. 10 [file 44318_2024_192_MOESM11_ESM.zip › Figure10/Figure10b/Atxn1/86Q_Merge.tif]

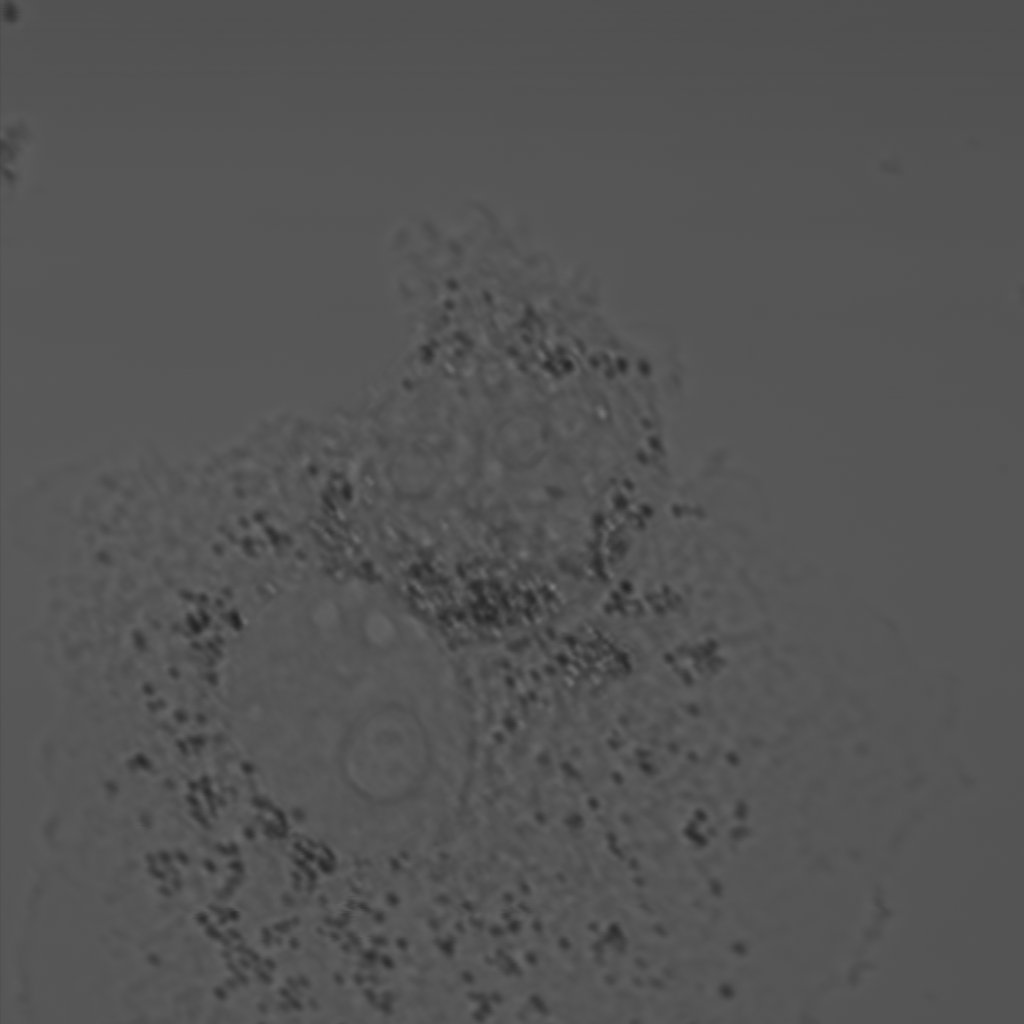

Supplement: Supplementary file 11 — Source data Fig. 10 [file 44318_2024_192_MOESM11_ESM.zip › Figure10/Figure10b/Atxn7/10Q_Bright field.tif]

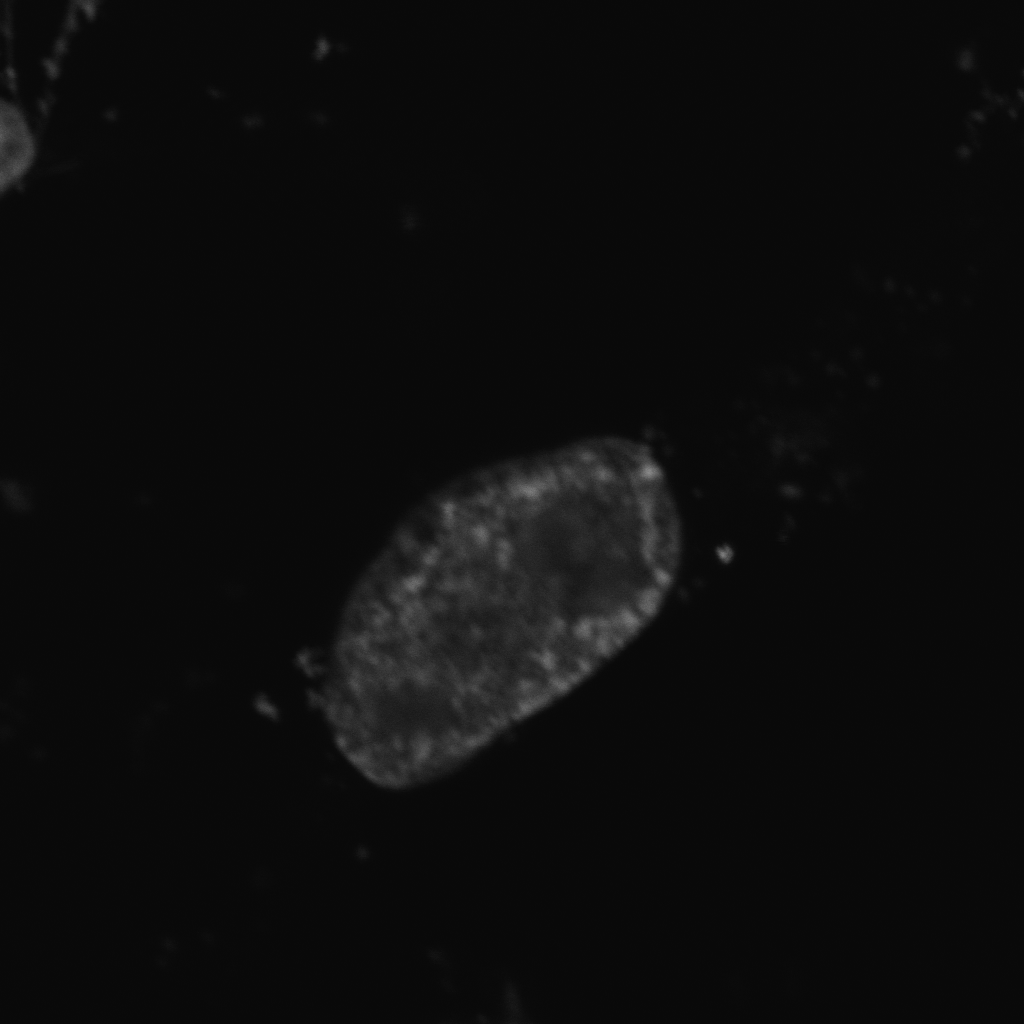

Supplement: Supplementary file 11 — Source data Fig. 10 [file 44318_2024_192_MOESM11_ESM.zip › Figure10/Figure10b/Atxn7/10Q_DAPI.tif]

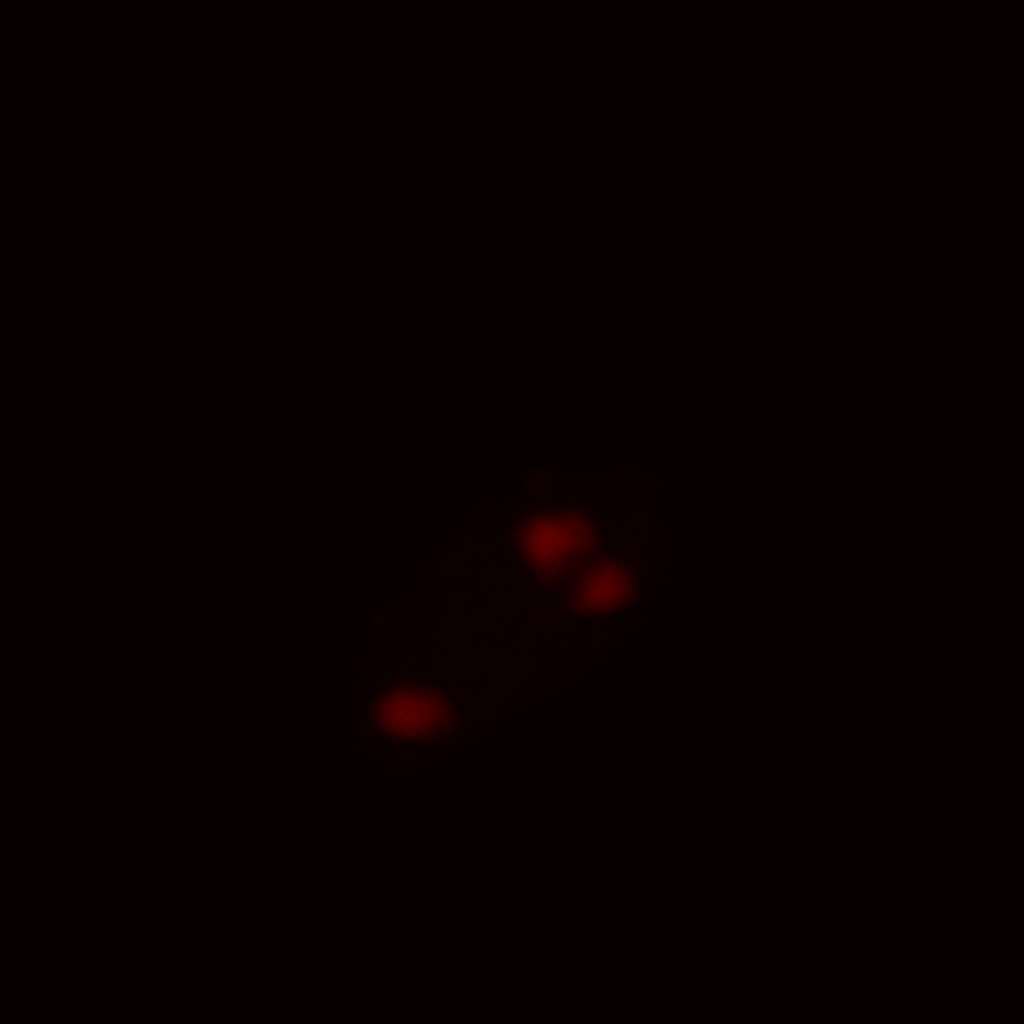

Supplement: Supplementary file 11 — Source data Fig. 10 [file 44318_2024_192_MOESM11_ESM.zip › Figure10/Figure10b/Atxn7/10Q_DsRed-Atxn7.tif]

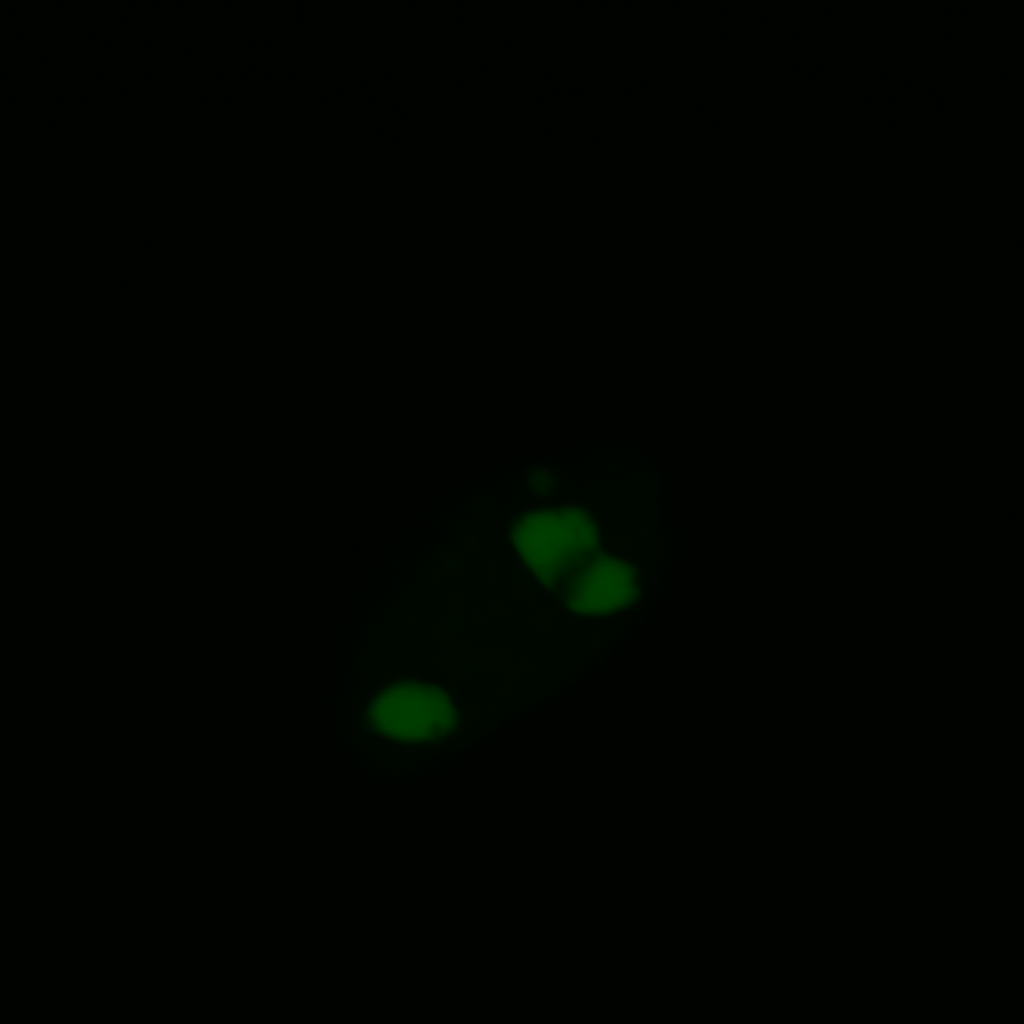

Supplement: Supplementary file 11 — Source data Fig. 10 [file 44318_2024_192_MOESM11_ESM.zip › Figure10/Figure10b/Atxn7/10Q_EGFP-PQBP3.tif]

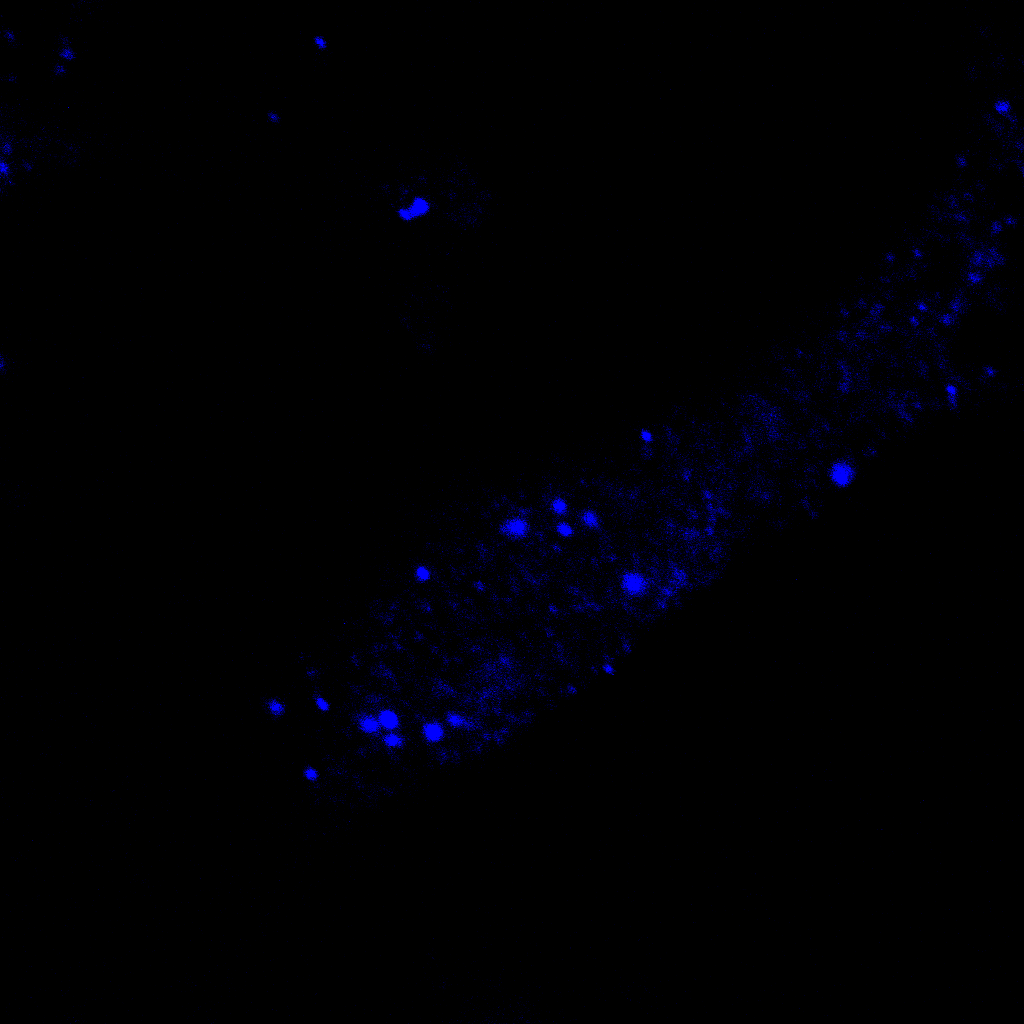

Supplement: Supplementary file 11 — Source data Fig. 10 [file 44318_2024_192_MOESM11_ESM.zip › Figure10/Figure10b/Atxn7/10Q_Fibrillarin.tif]

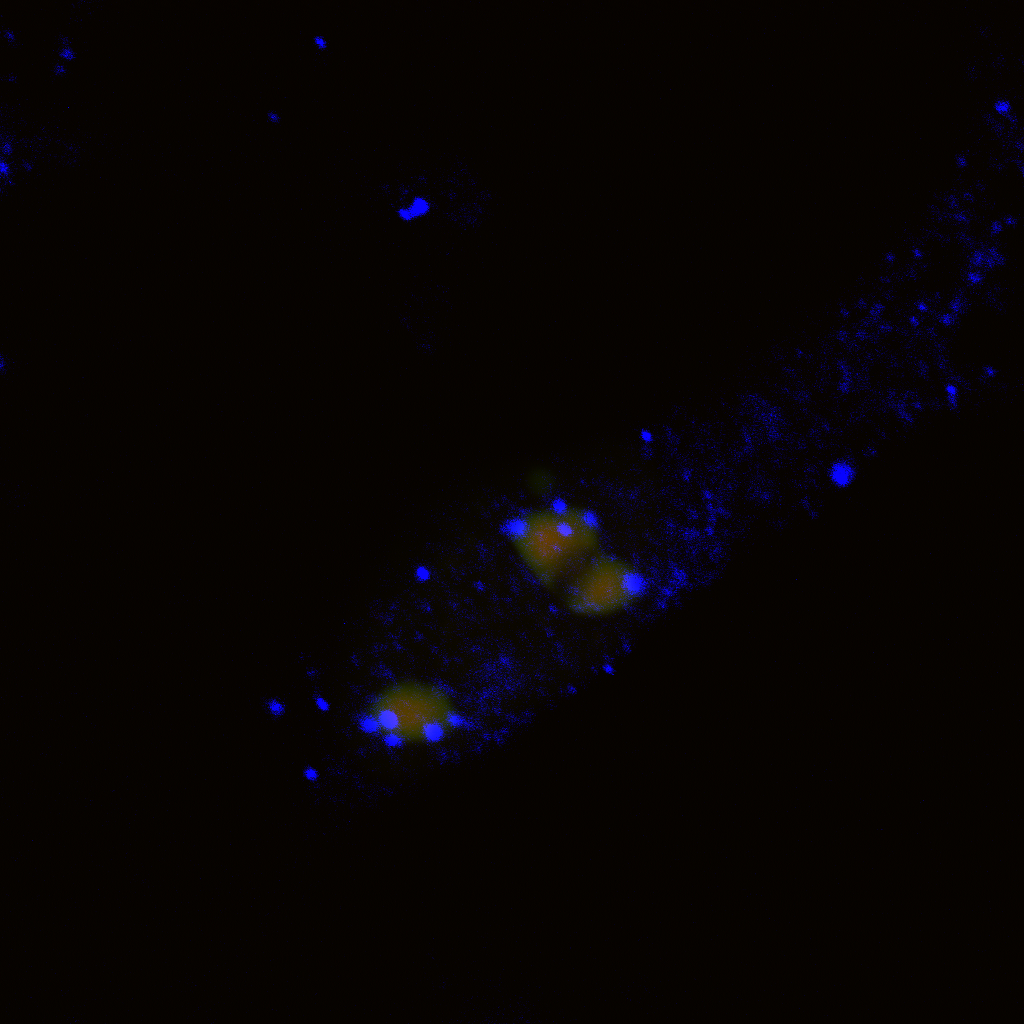

Supplement: Supplementary file 11 — Source data Fig. 10 [file 44318_2024_192_MOESM11_ESM.zip › Figure10/Figure10b/Atxn7/10Q_Merge.tif]

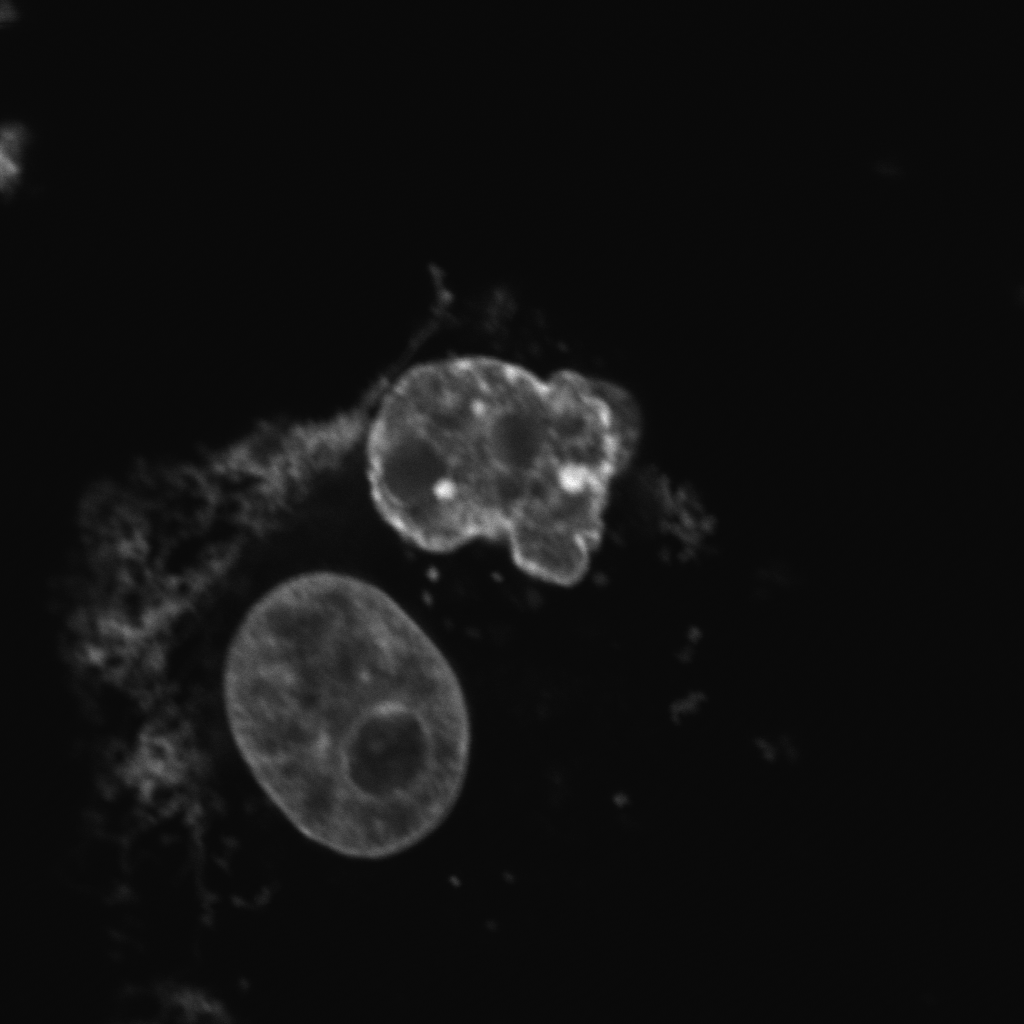

Supplement: Supplementary file 11 — Source data Fig. 10 [file 44318_2024_192_MOESM11_ESM.zip › Figure10/Figure10b/Atxn7/92Q_DAPI.tif]

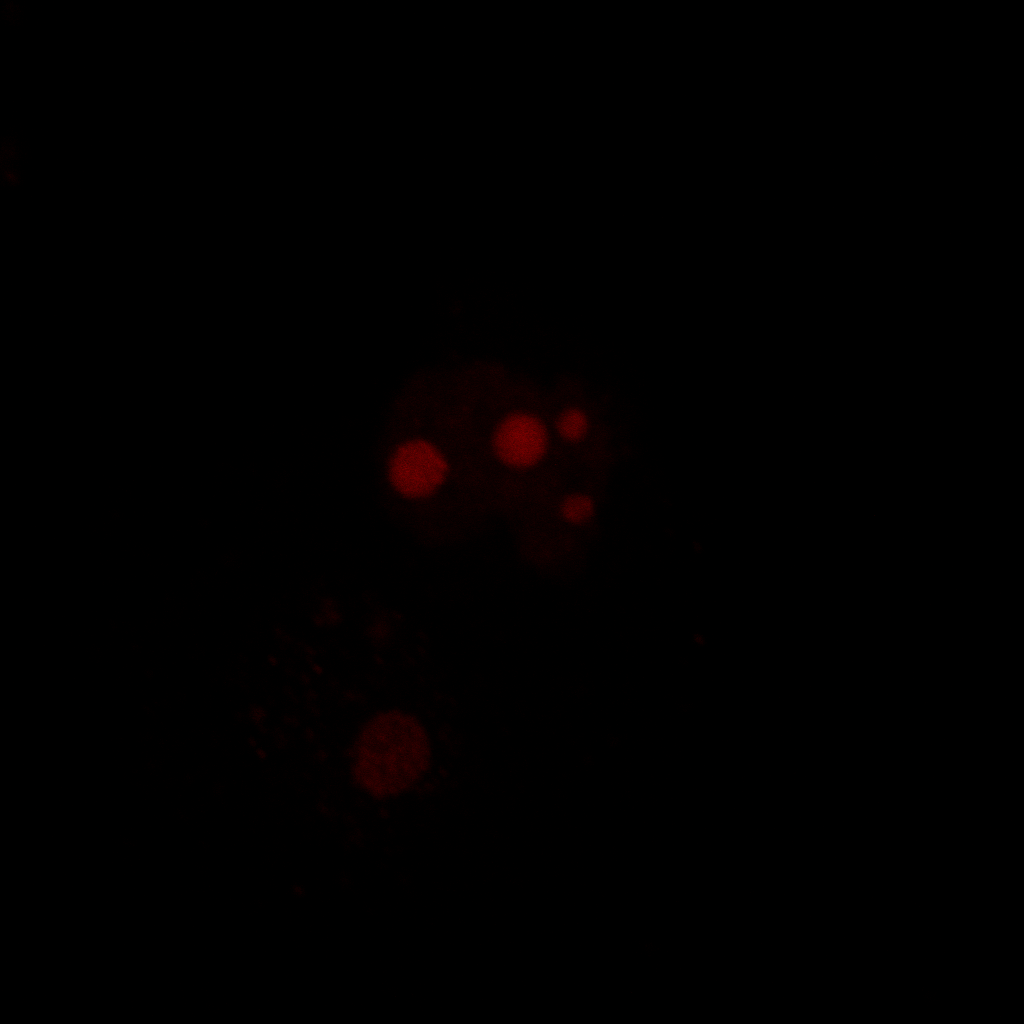

Supplement: Supplementary file 11 — Source data Fig. 10 [file 44318_2024_192_MOESM11_ESM.zip › Figure10/Figure10b/Atxn7/92Q_DsRed-Atxn7.tif]

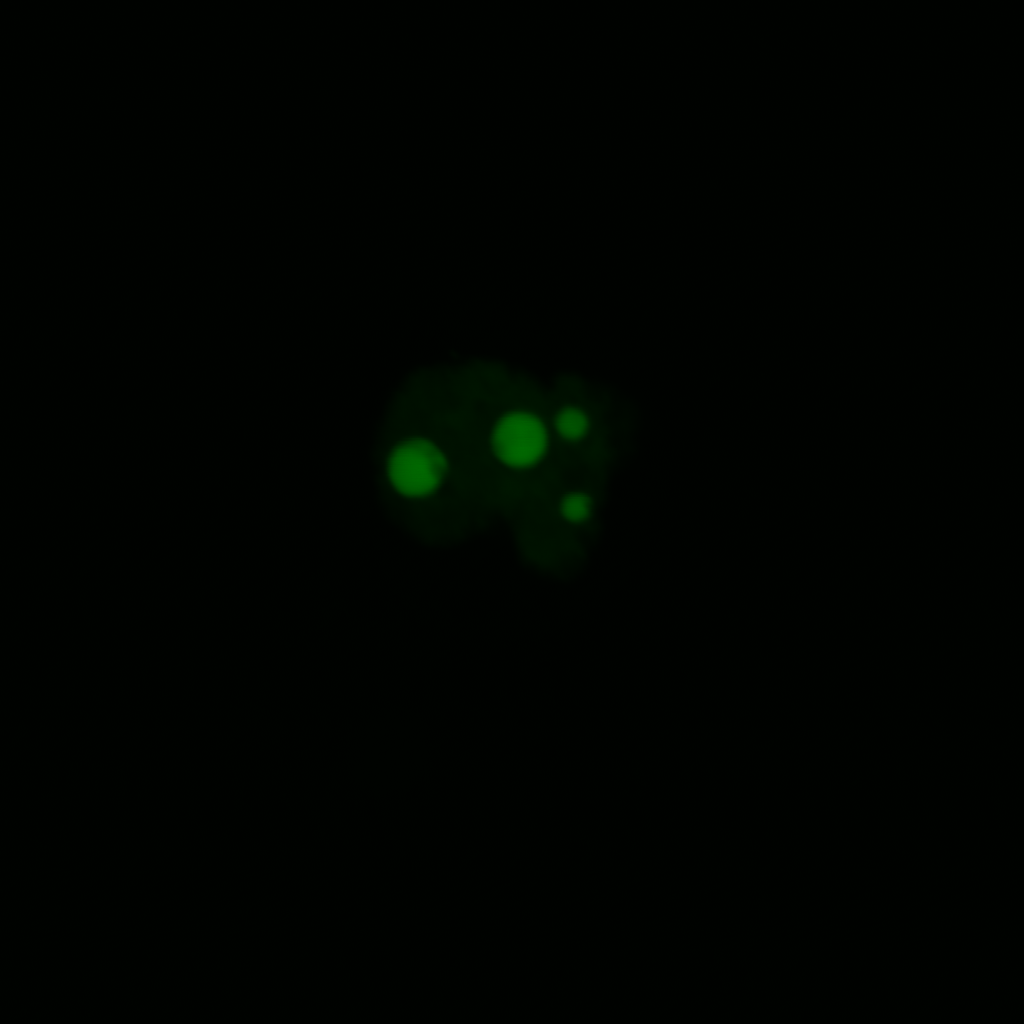

Supplement: Supplementary file 11 — Source data Fig. 10 [file 44318_2024_192_MOESM11_ESM.zip › Figure10/Figure10b/Atxn7/92Q_EGFP-PQBP3.tif]

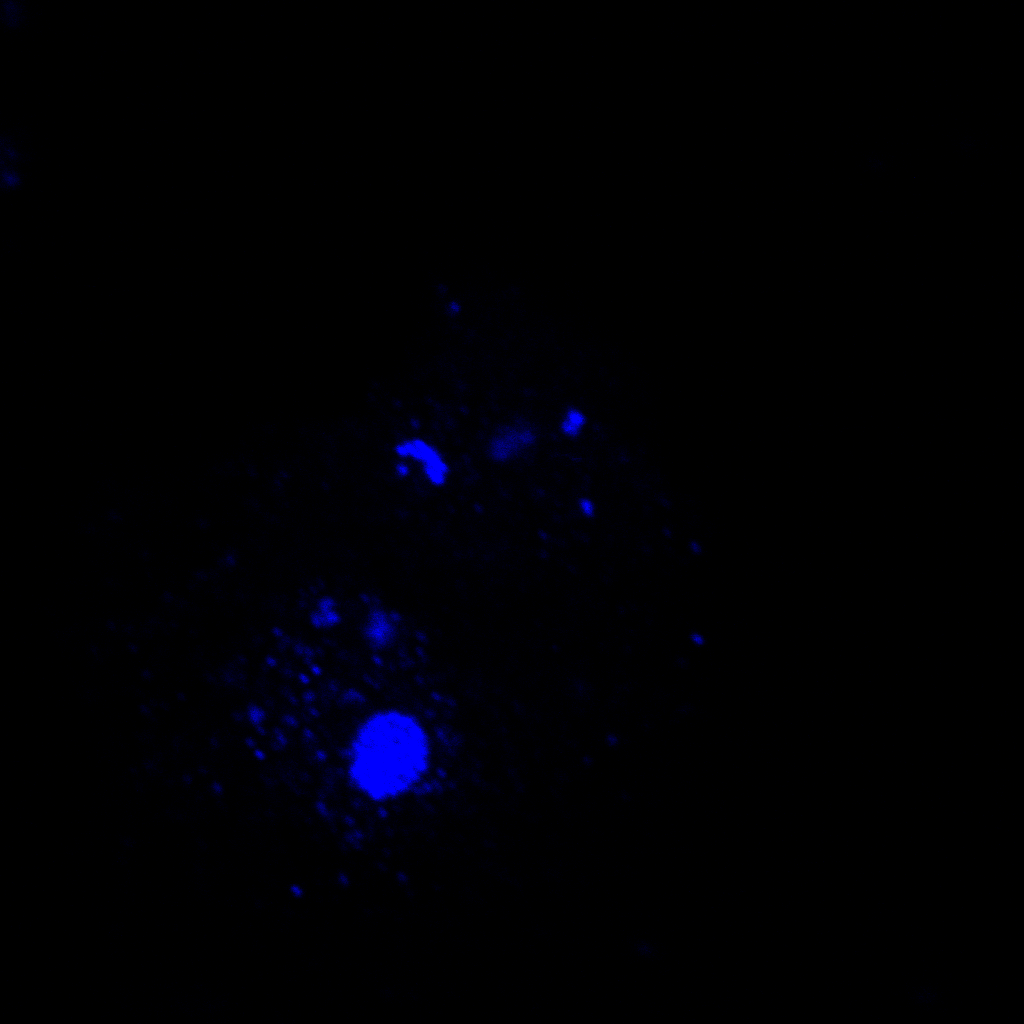

Supplement: Supplementary file 11 — Source data Fig. 10 [file 44318_2024_192_MOESM11_ESM.zip › Figure10/Figure10b/Atxn7/92Q_Fibrillarin.tif]

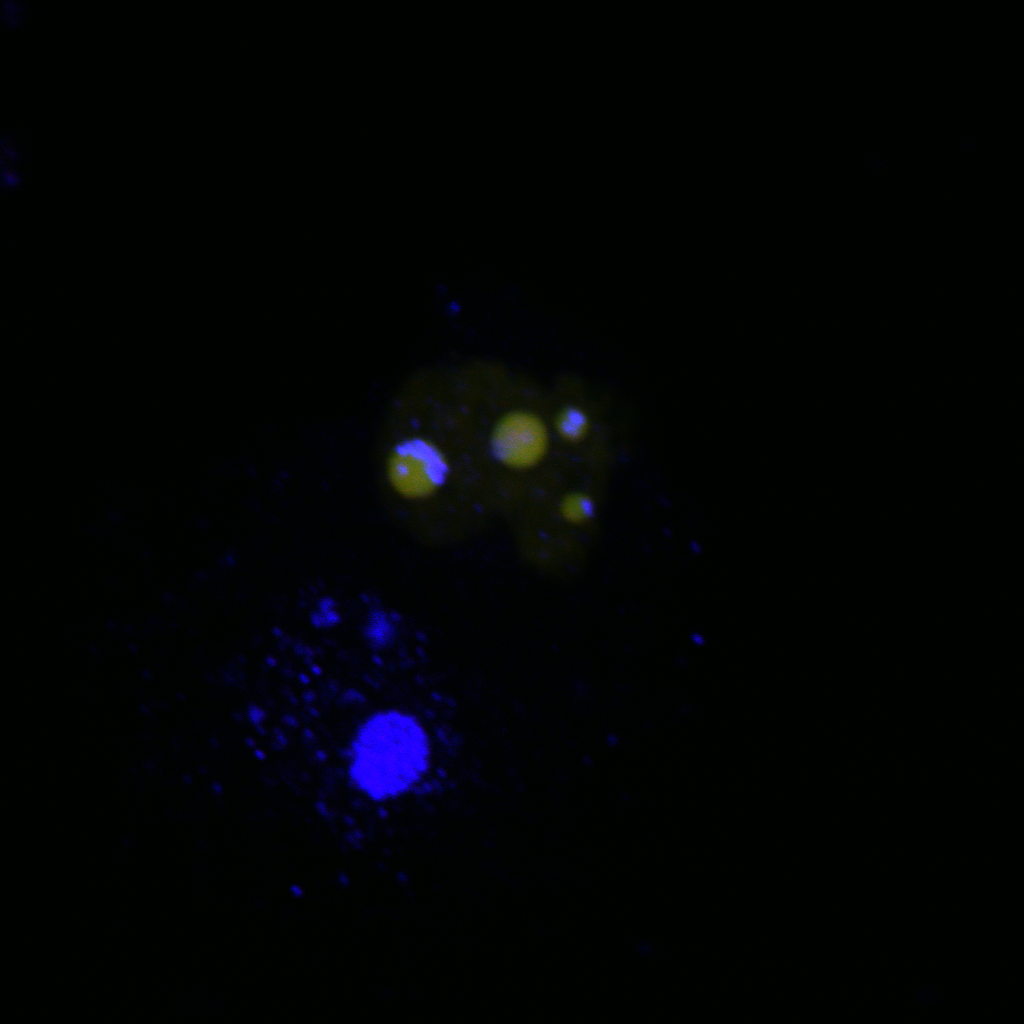

Supplement: Supplementary file 11 — Source data Fig. 10 [file 44318_2024_192_MOESM11_ESM.zip › Figure10/Figure10b/Atxn7/92Q_Merge.tif]

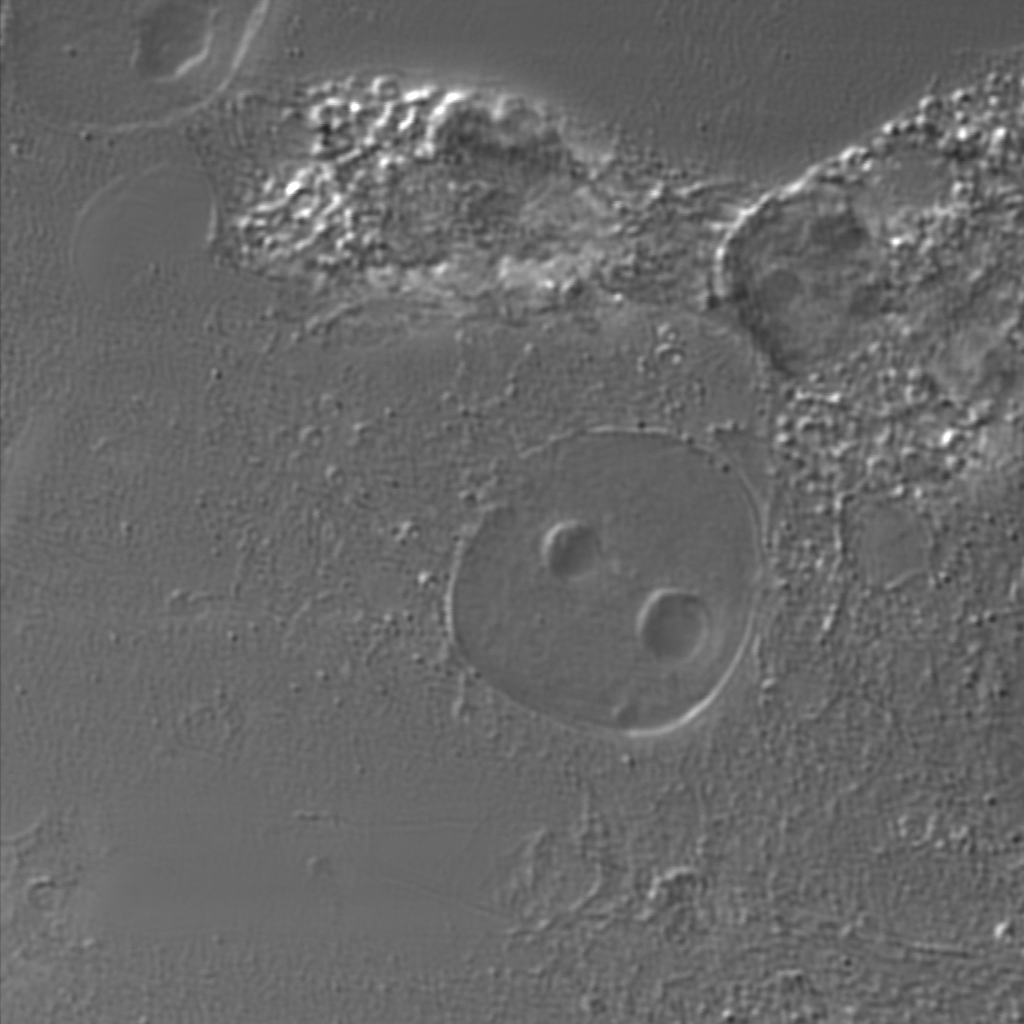

Supplement: Supplementary file 11 — Source data Fig. 10 [file 44318_2024_192_MOESM11_ESM.zip › Figure10/Figure10b/Htt/103Q_Bright field.tif]

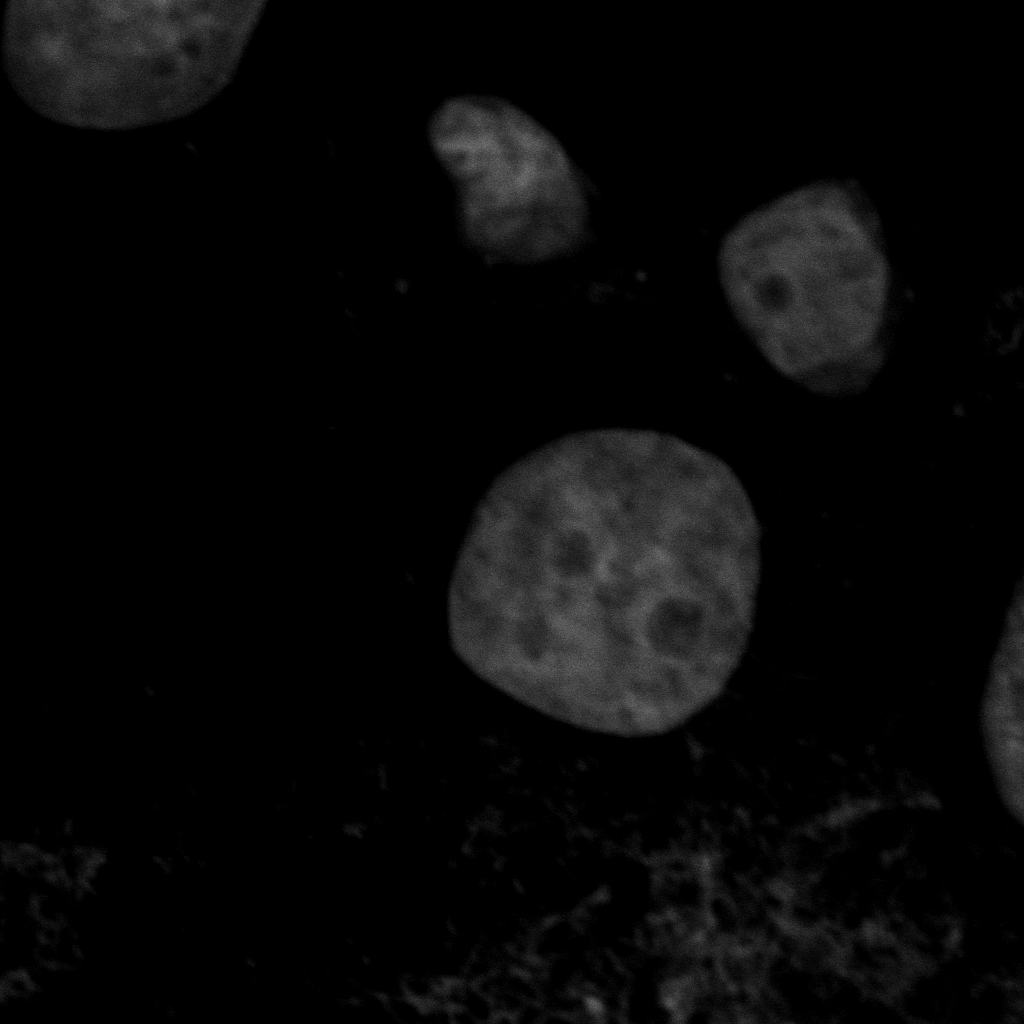

Supplement: Supplementary file 11 — Source data Fig. 10 [file 44318_2024_192_MOESM11_ESM.zip › Figure10/Figure10b/Htt/103Q_DAPI.tif]

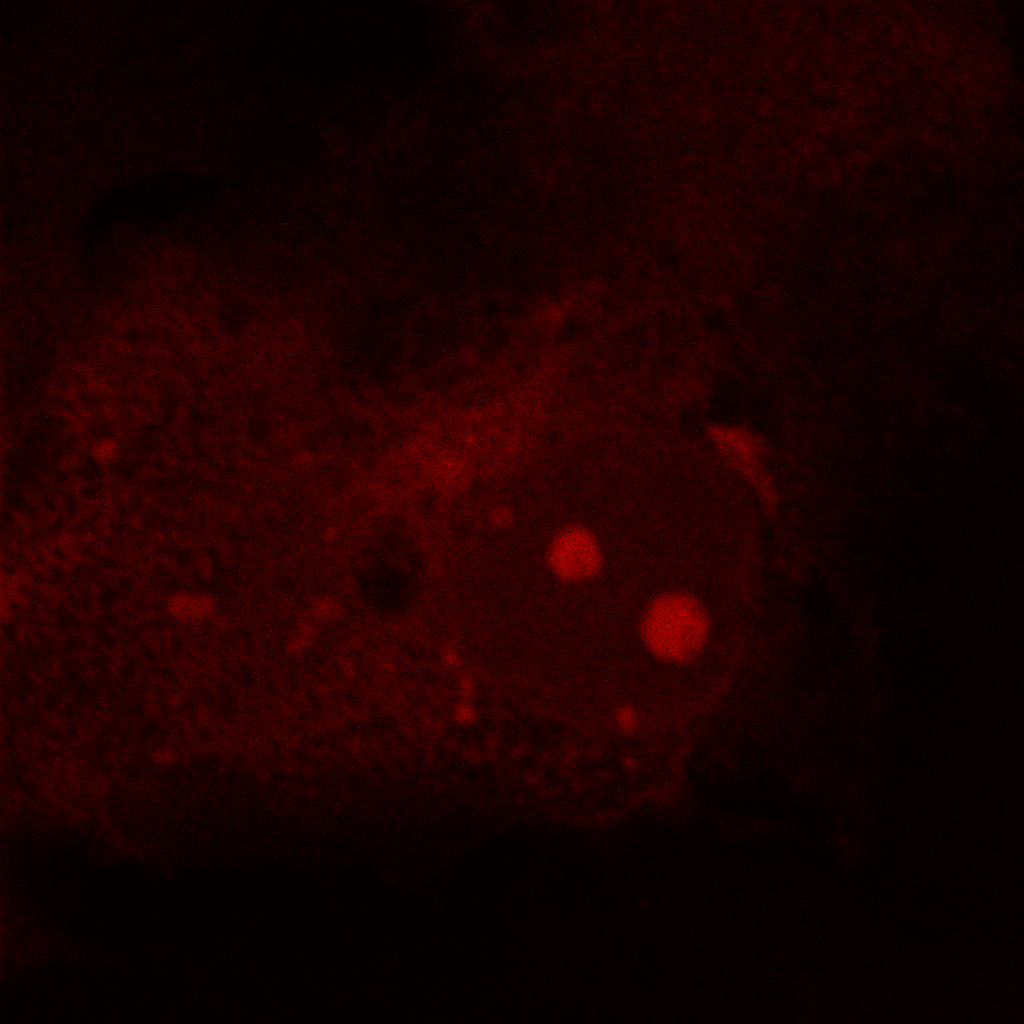

Supplement: Supplementary file 11 — Source data Fig. 10 [file 44318_2024_192_MOESM11_ESM.zip › Figure10/Figure10b/Htt/103Q_DsRed-Htt.tif]

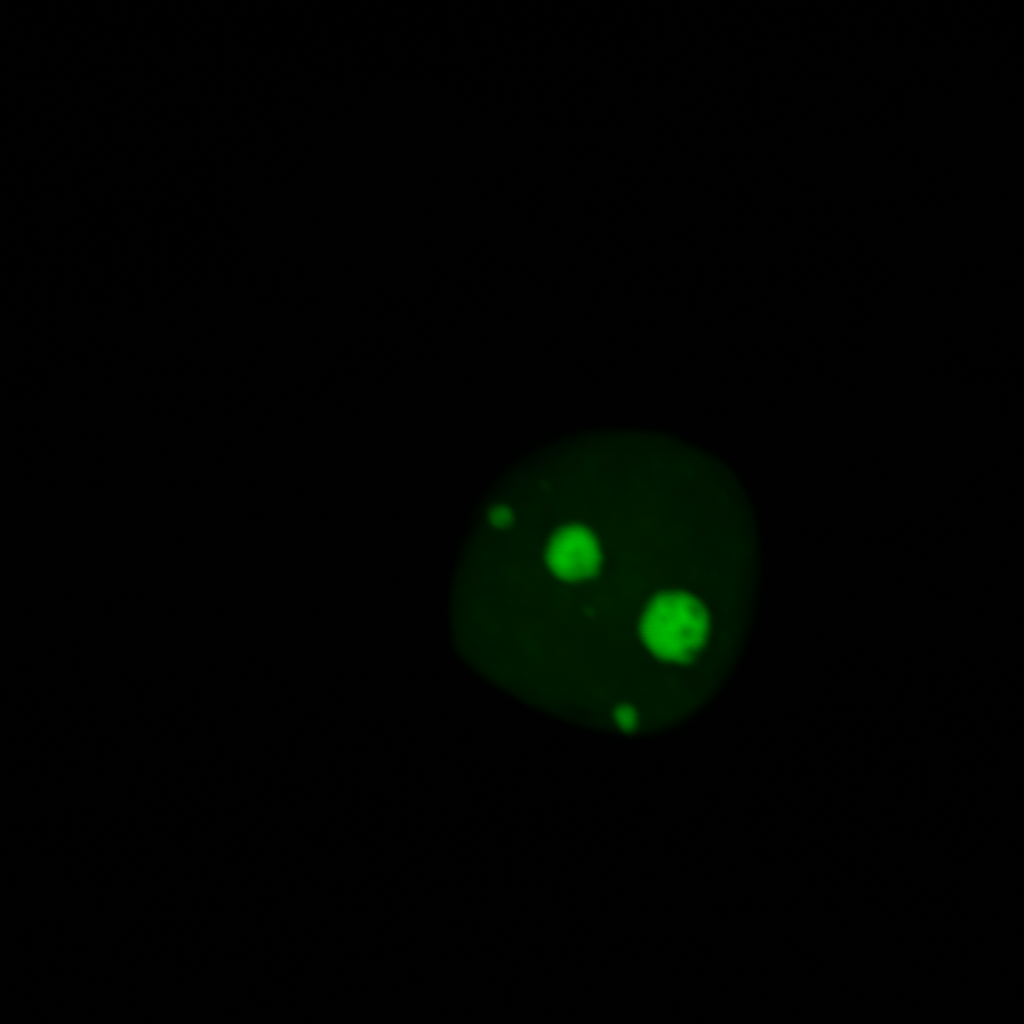

Supplement: Supplementary file 11 — Source data Fig. 10 [file 44318_2024_192_MOESM11_ESM.zip › Figure10/Figure10b/Htt/103Q_EGFP-PQBP3.tif]

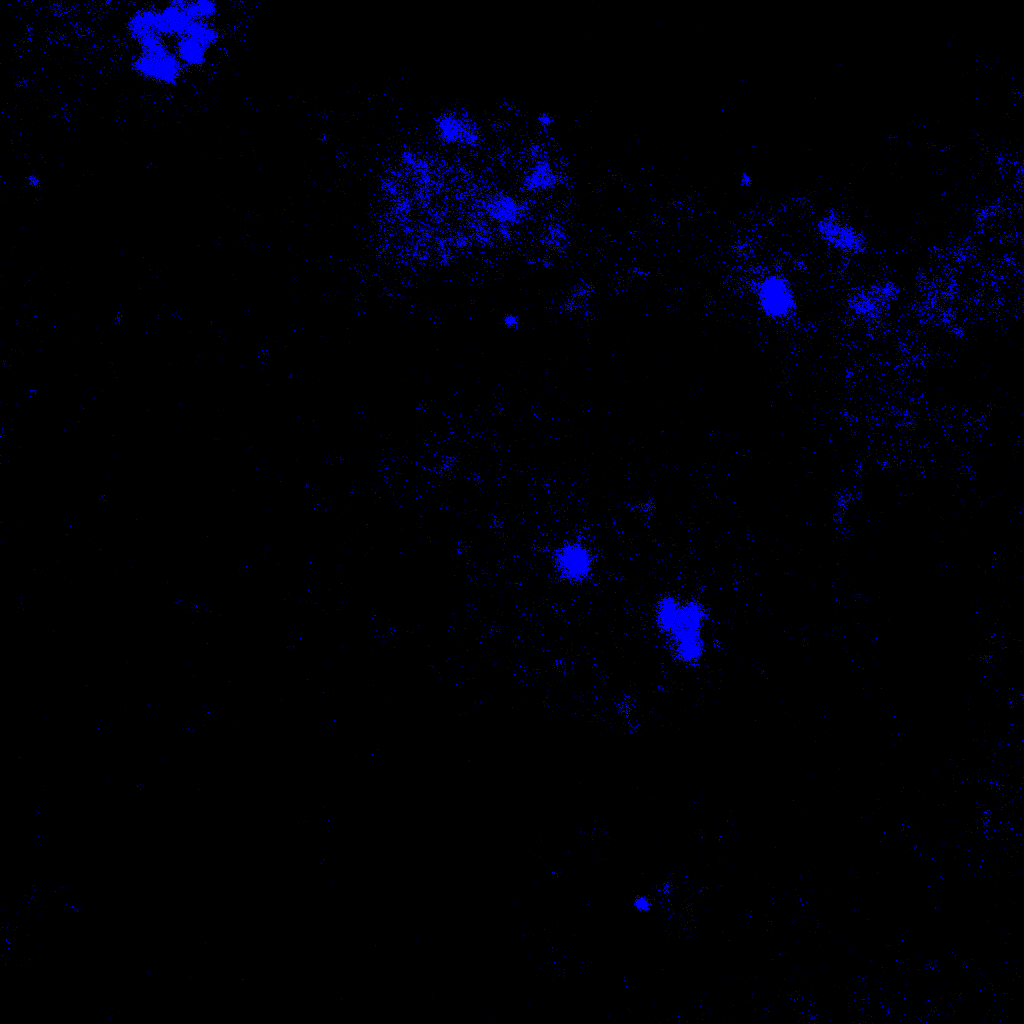

Supplement: Supplementary file 11 — Source data Fig. 10 [file 44318_2024_192_MOESM11_ESM.zip › Figure10/Figure10b/Htt/103Q_Fibrillarin.tif]

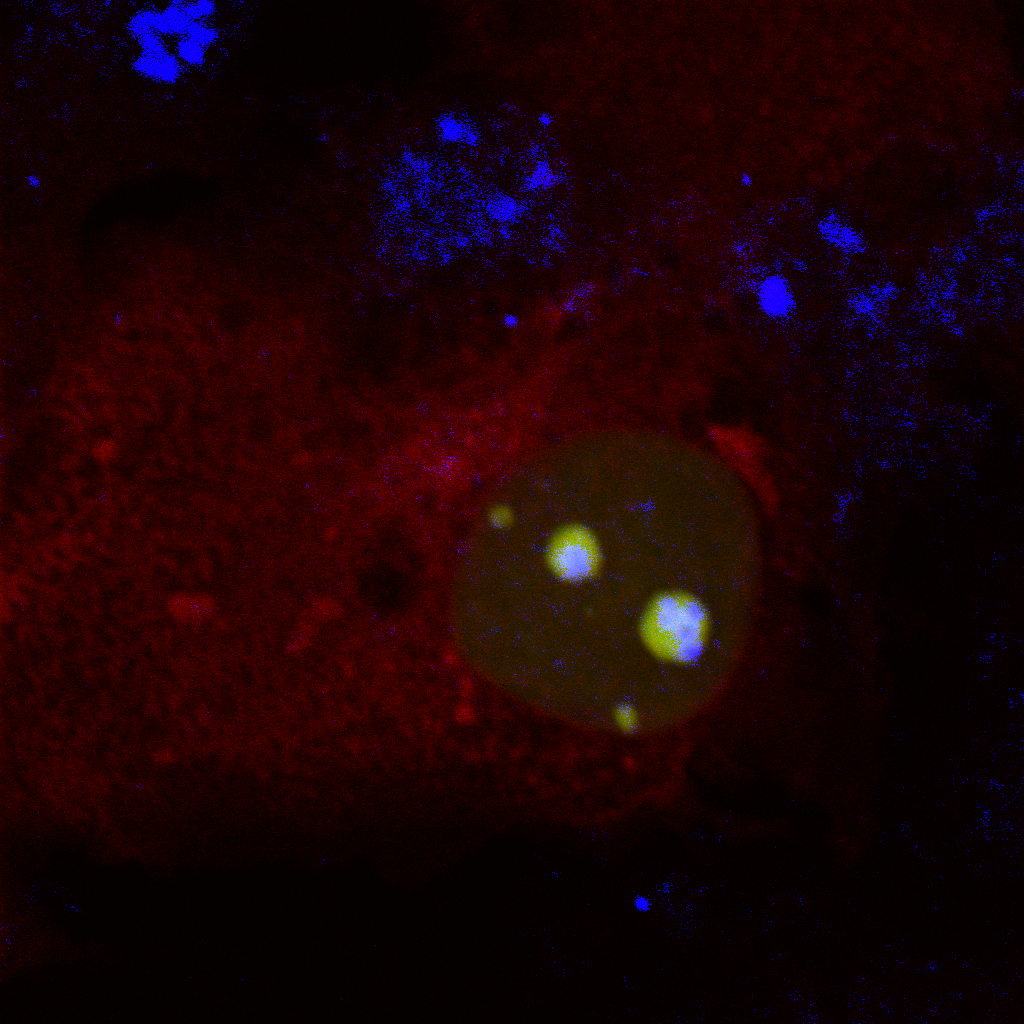

Supplement: Supplementary file 11 — Source data Fig. 10 [file 44318_2024_192_MOESM11_ESM.zip › Figure10/Figure10b/Htt/103Q_Merge.tif]

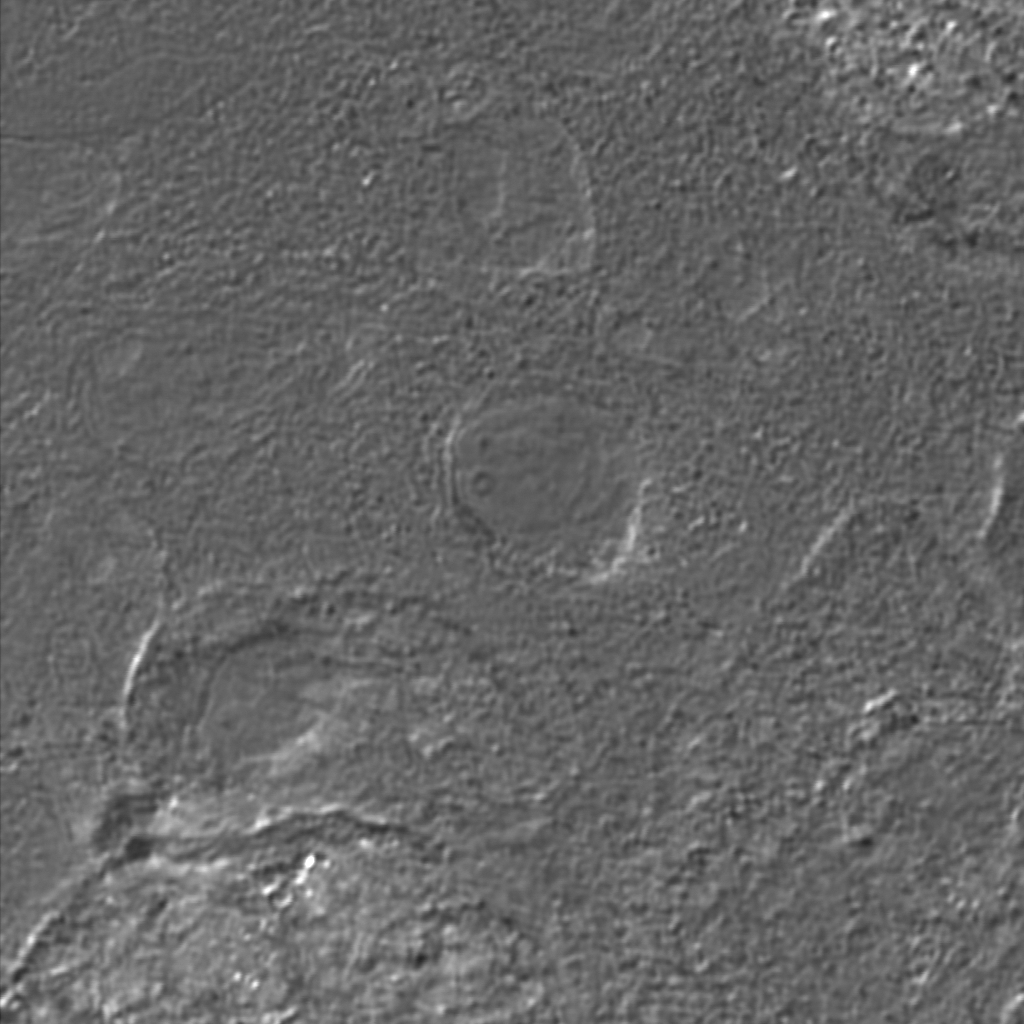

Supplement: Supplementary file 11 — Source data Fig. 10 [file 44318_2024_192_MOESM11_ESM.zip › Figure10/Figure10b/Htt/20Q_Bright field.tif]

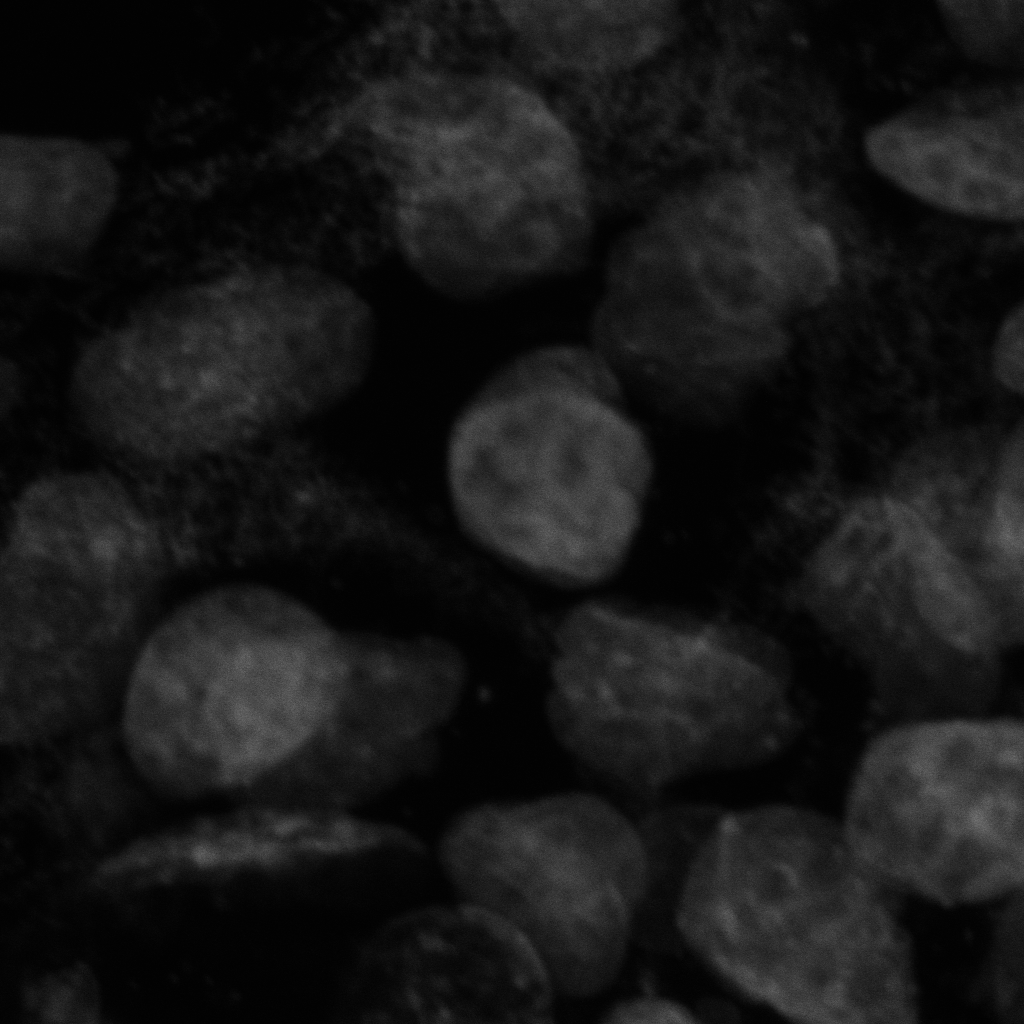

Supplement: Supplementary file 11 — Source data Fig. 10 [file 44318_2024_192_MOESM11_ESM.zip › Figure10/Figure10b/Htt/20Q_DAPI.tif]

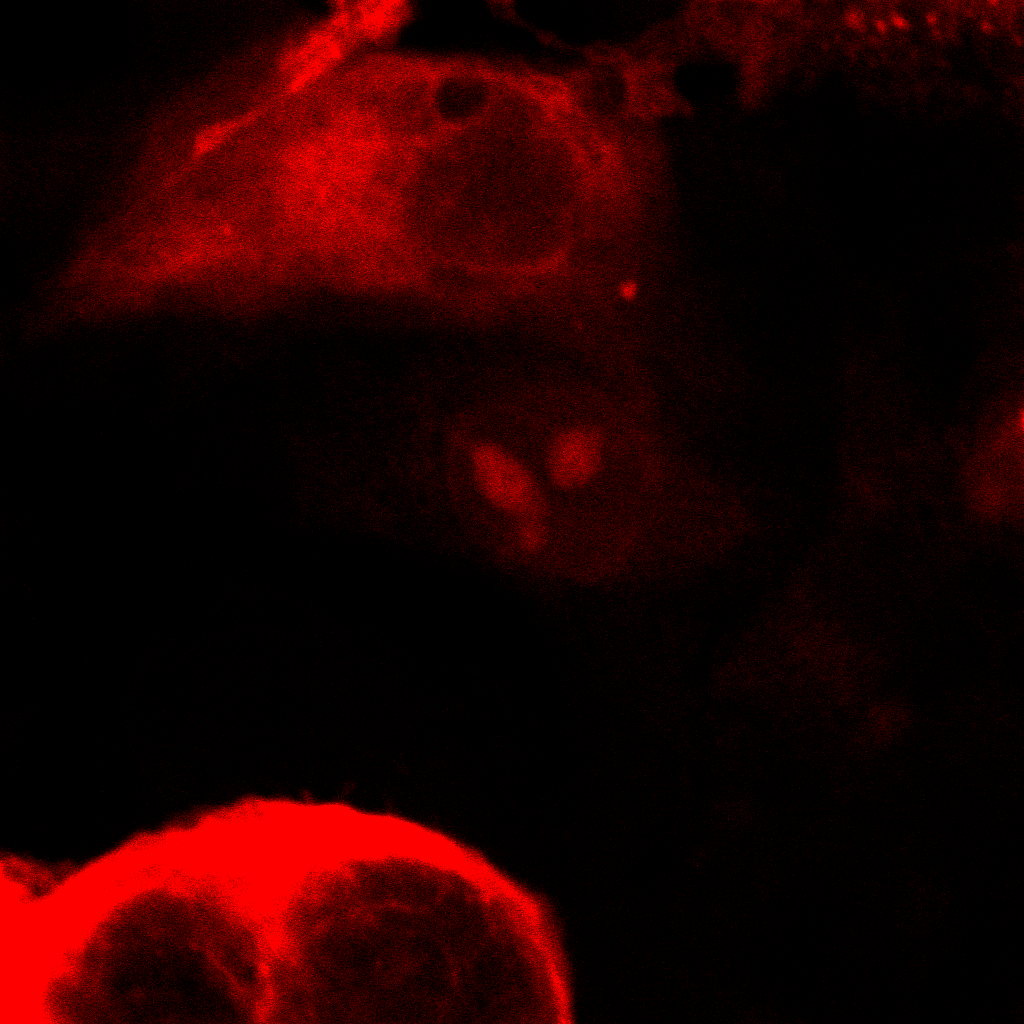

Supplement: Supplementary file 11 — Source data Fig. 10 [file 44318_2024_192_MOESM11_ESM.zip › Figure10/Figure10b/Htt/20Q_DsRed-Htt.tif]

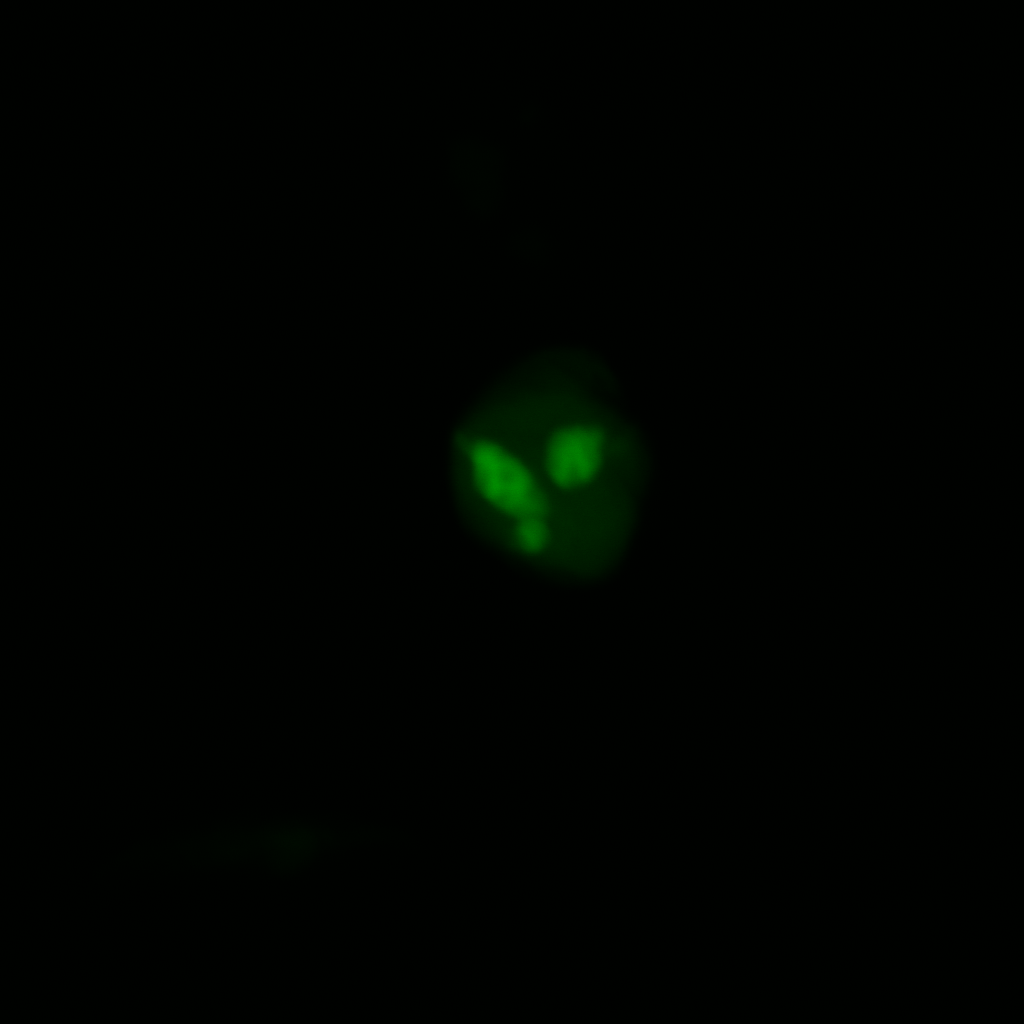

Supplement: Supplementary file 11 — Source data Fig. 10 [file 44318_2024_192_MOESM11_ESM.zip › Figure10/Figure10b/Htt/20Q_EGFP-PQBP3.tif]

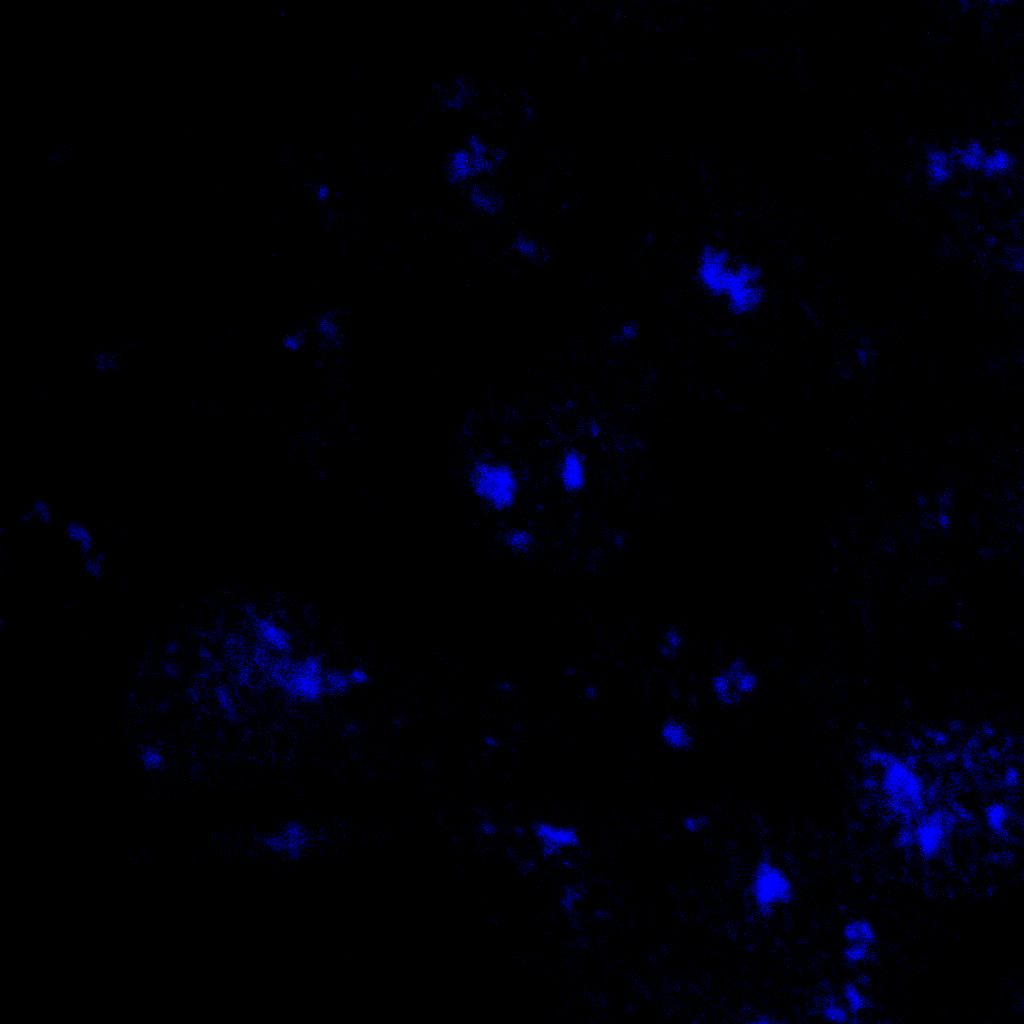

Supplement: Supplementary file 11 — Source data Fig. 10 [file 44318_2024_192_MOESM11_ESM.zip › Figure10/Figure10b/Htt/20Q_Fibrillarin.tif]

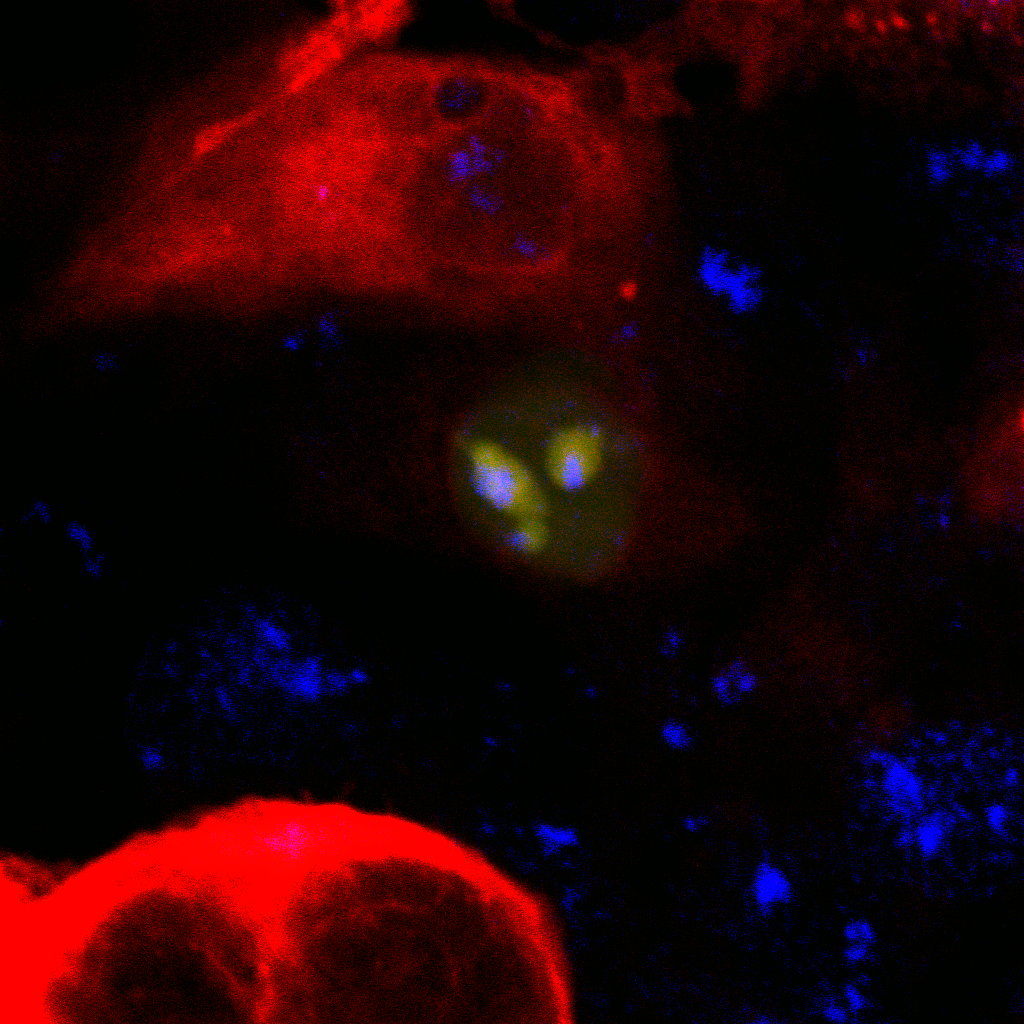

Supplement: Supplementary file 11 — Source data Fig. 10 [file 44318_2024_192_MOESM11_ESM.zip › Figure10/Figure10b/Htt/20Q_Merge.tif]

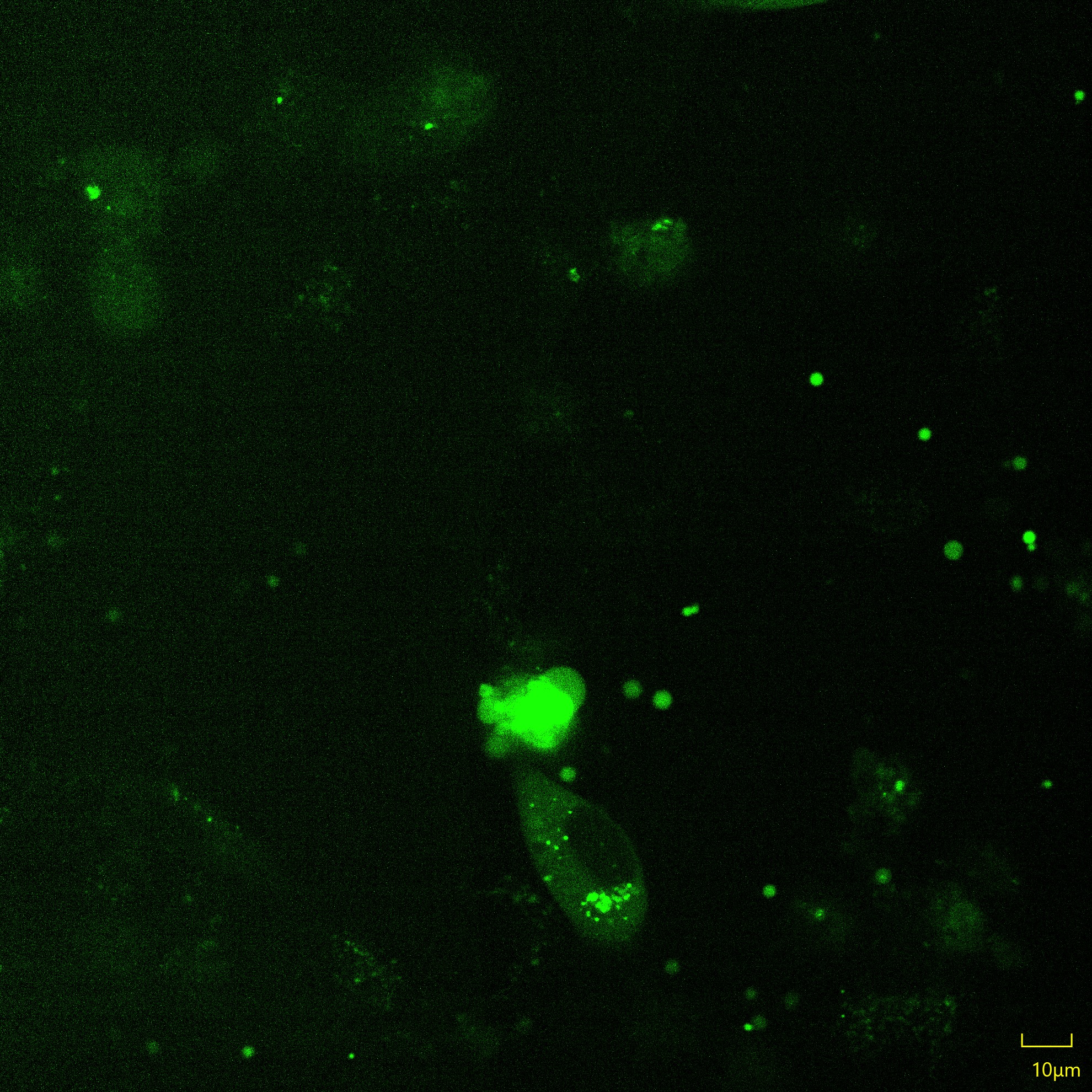

Supplement: Supplementary file 11 — Source data Fig. 10 [file 44318_2024_192_MOESM11_ESM.zip › Figure10/Figure10c/confluence_cyto.jpg]

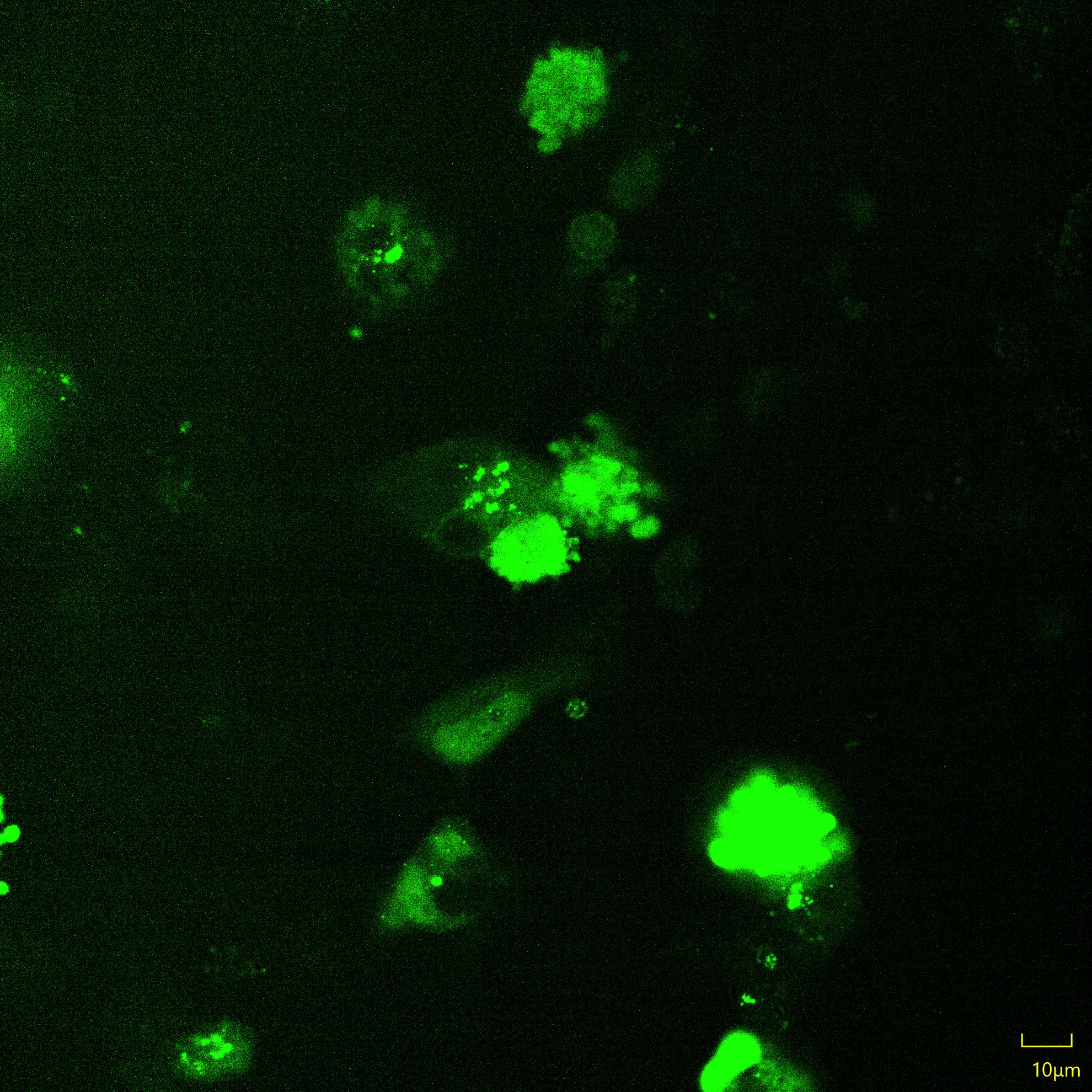

Supplement: Supplementary file 11 — Source data Fig. 10 [file 44318_2024_192_MOESM11_ESM.zip › Figure10/Figure10c/confluence_nuc.jpg]

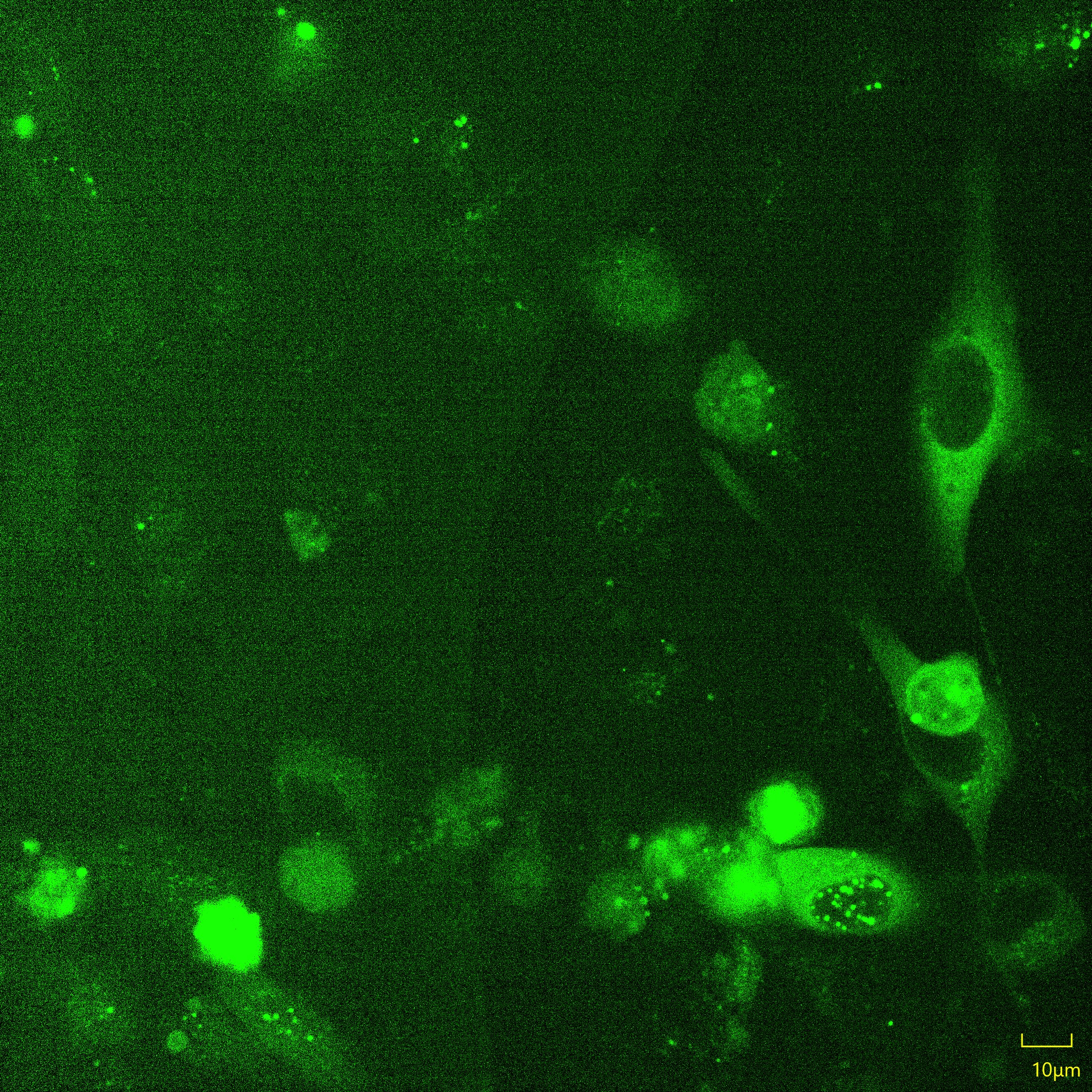

Supplement: Supplementary file 11 — Source data Fig. 10 [file 44318_2024_192_MOESM11_ESM.zip › Figure10/Figure10c/H2O2_cyto.jpg]

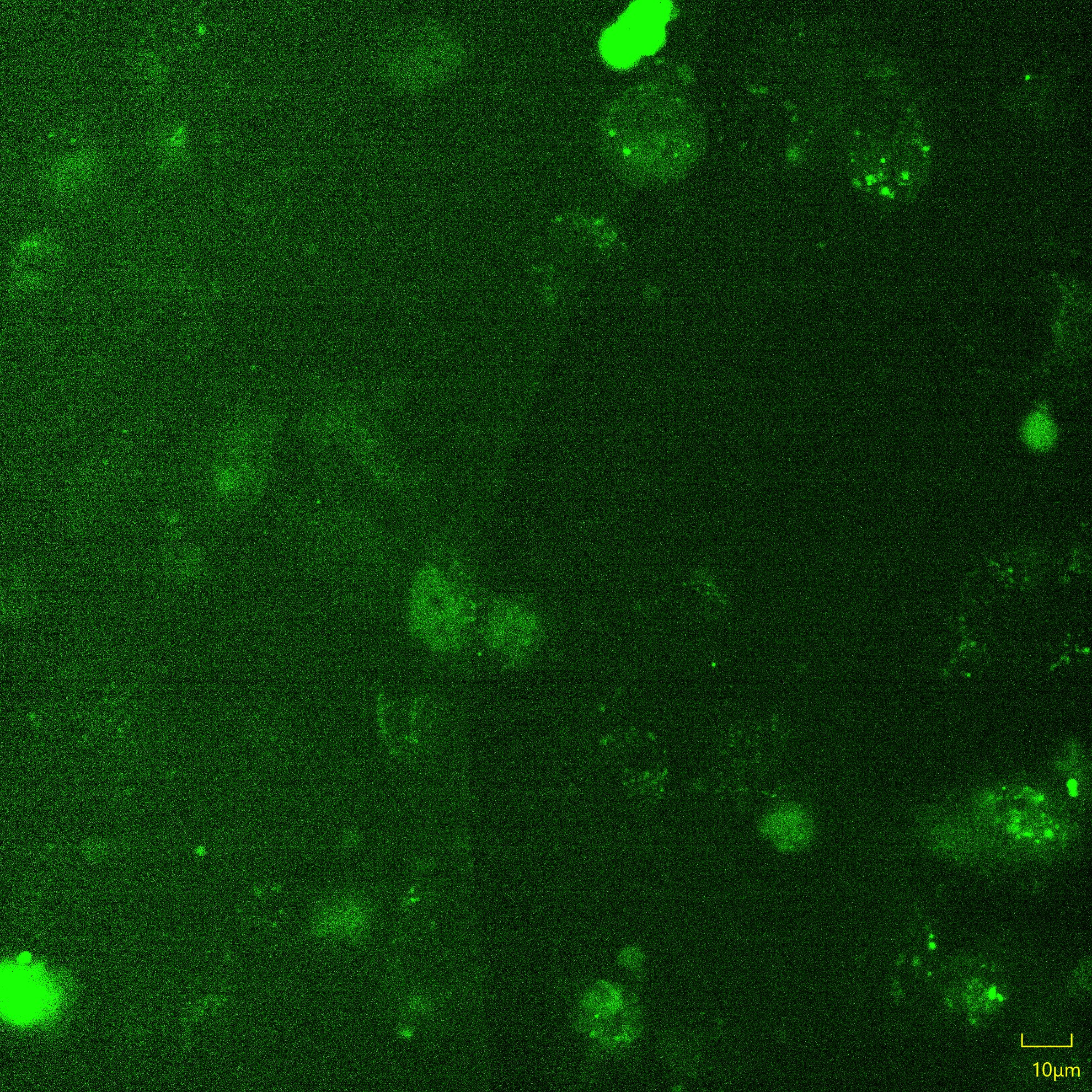

Supplement: Supplementary file 11 — Source data Fig. 10 [file 44318_2024_192_MOESM11_ESM.zip › Figure10/Figure10c/H2O2_nuc.jpg]

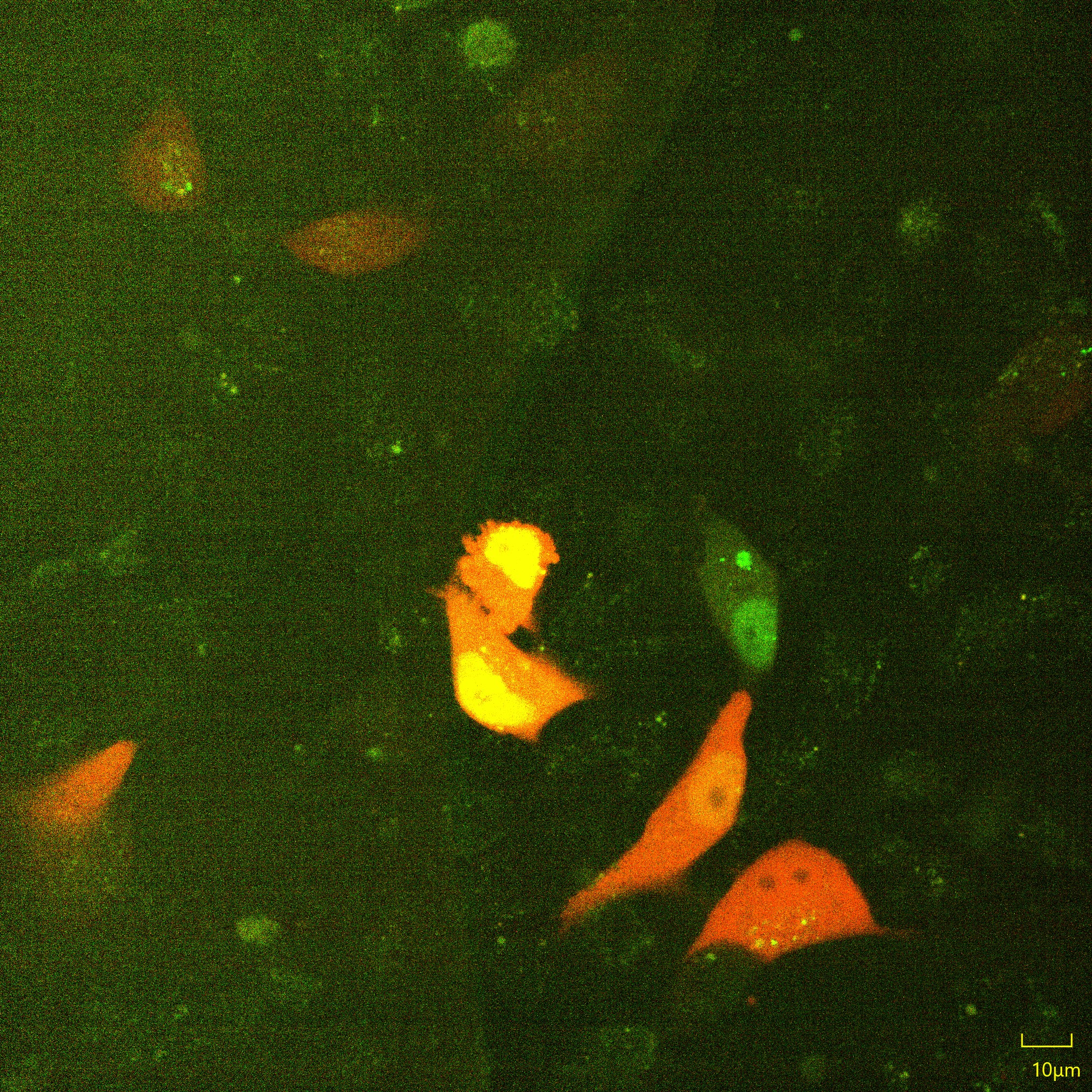

Supplement: Supplementary file 11 — Source data Fig. 10 [file 44318_2024_192_MOESM11_ESM.zip › Figure10/Figure10c/mutan_Atxn1_cyto_merge.jpg]
