# Supplementary figures and images for: PQBP3 prevents senescence by suppressing PSME3-mediated proteasomal Lamin B1 degradation (part 4 of 4)
Source: EMBO J. 2024 Aug 5;43(18):3968–99. doi: 10.1038/s44318-024-00192-4 (PMC11405525; doi:10.1038/s44318-024-00192-4)

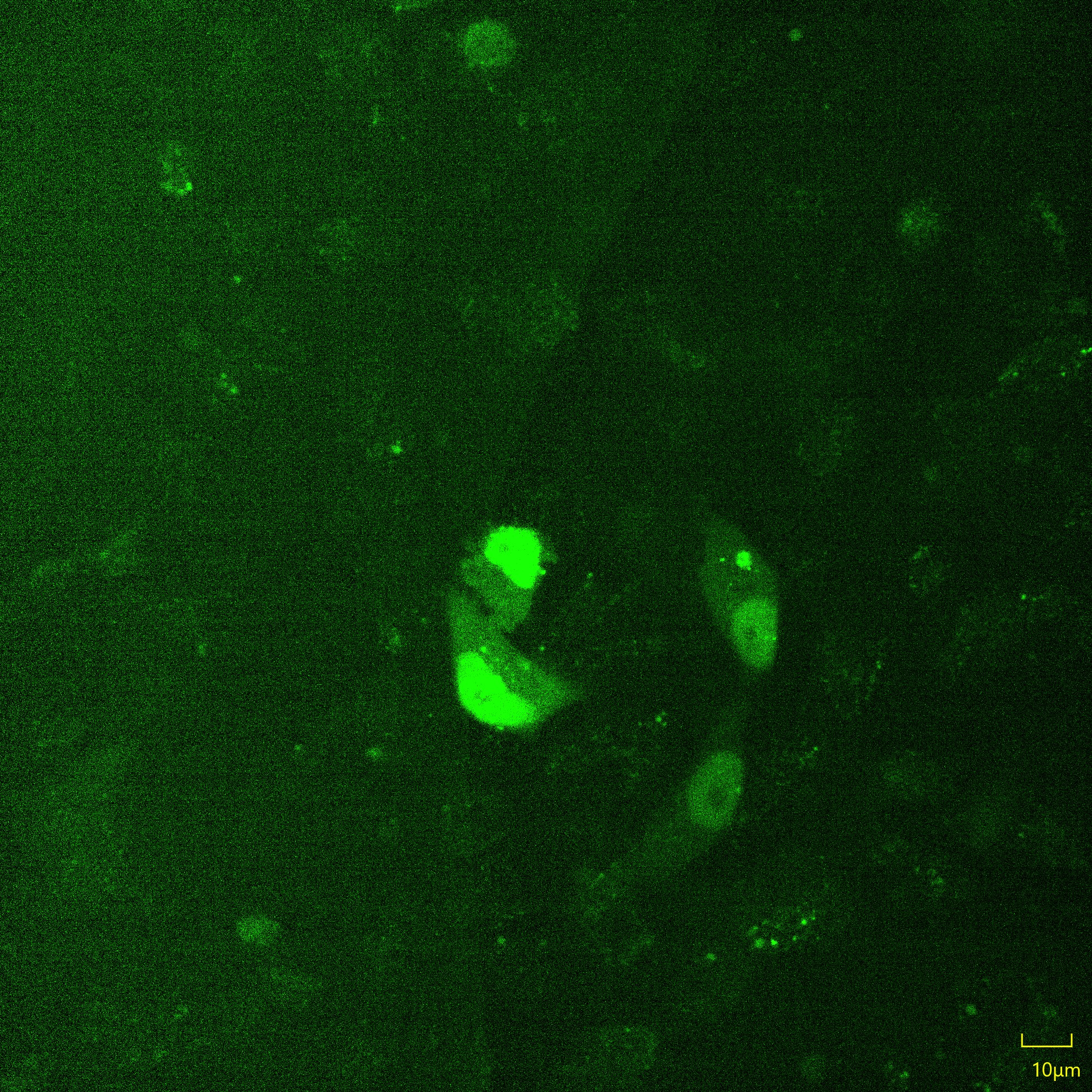

Supplement: Supplementary file 11 — Source data Fig. 10 [file 44318_2024_192_MOESM11_ESM.zip › Figure10/Figure10c/mutan_Atxn1_cyto_Venus.jpg]

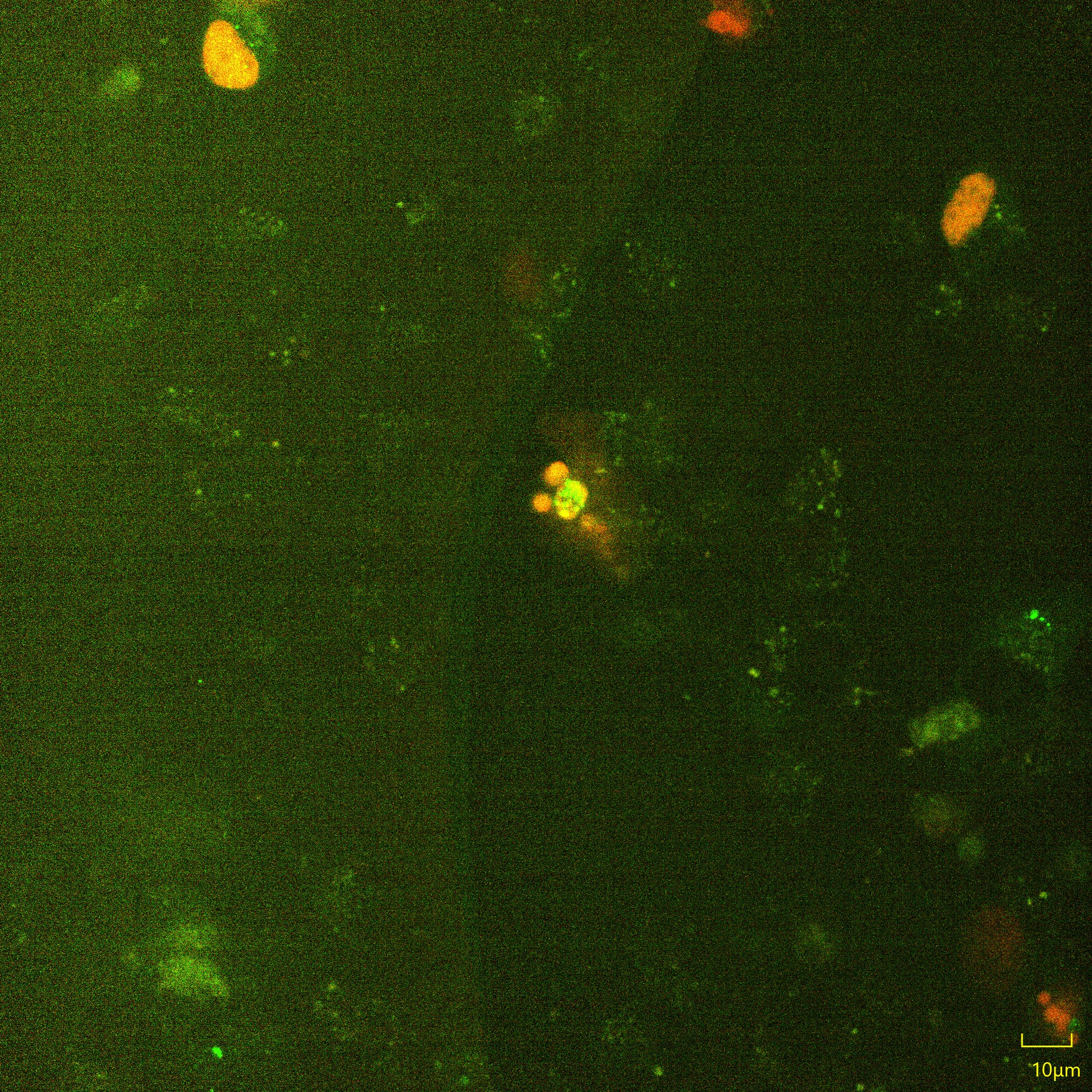

Supplement: Supplementary file 11 — Source data Fig. 10 [file 44318_2024_192_MOESM11_ESM.zip › Figure10/Figure10c/mutan_Atxn1_nuc_merge.jpg]

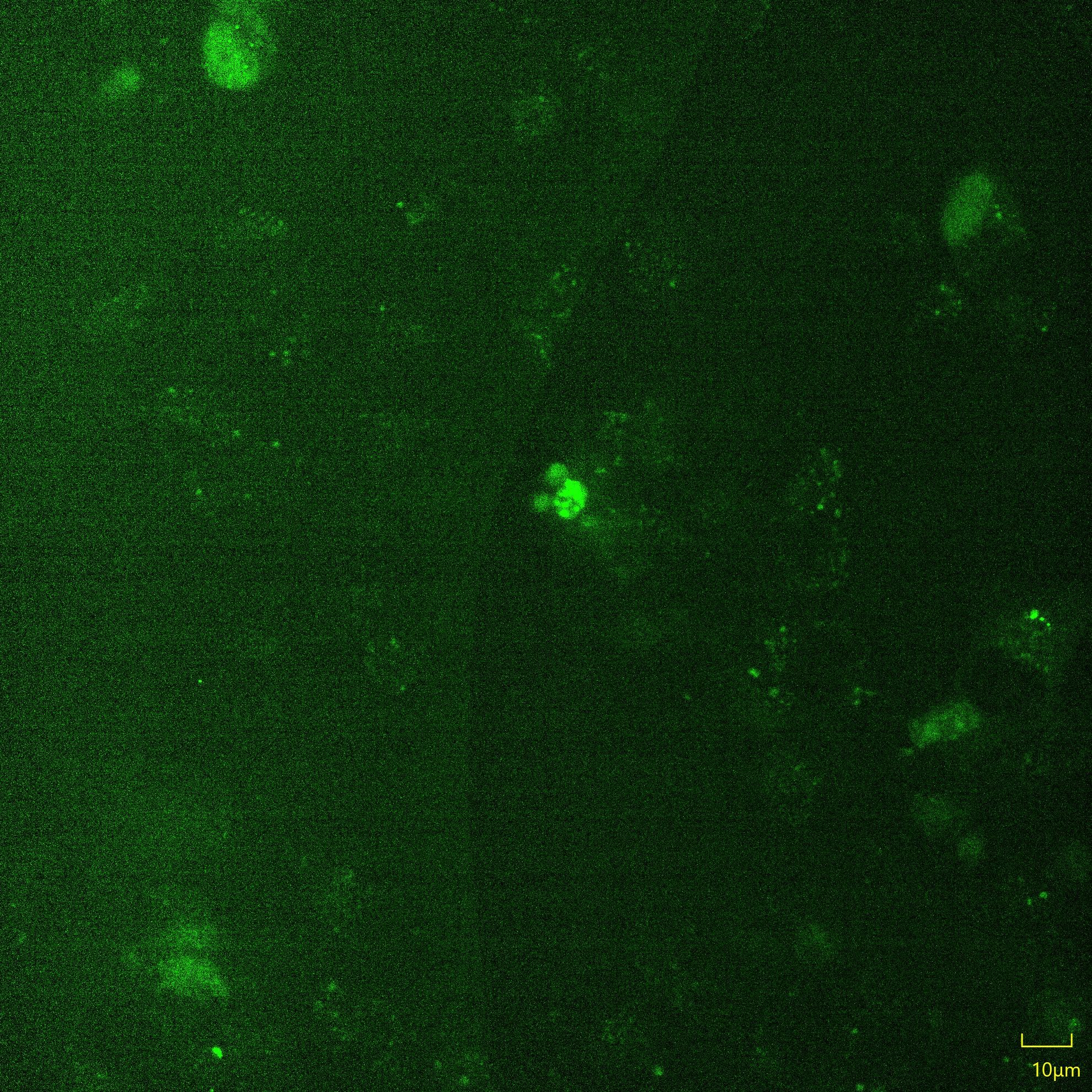

Supplement: Supplementary file 11 — Source data Fig. 10 [file 44318_2024_192_MOESM11_ESM.zip › Figure10/Figure10c/mutan_Atxn1_nuc_Venus.jpg]

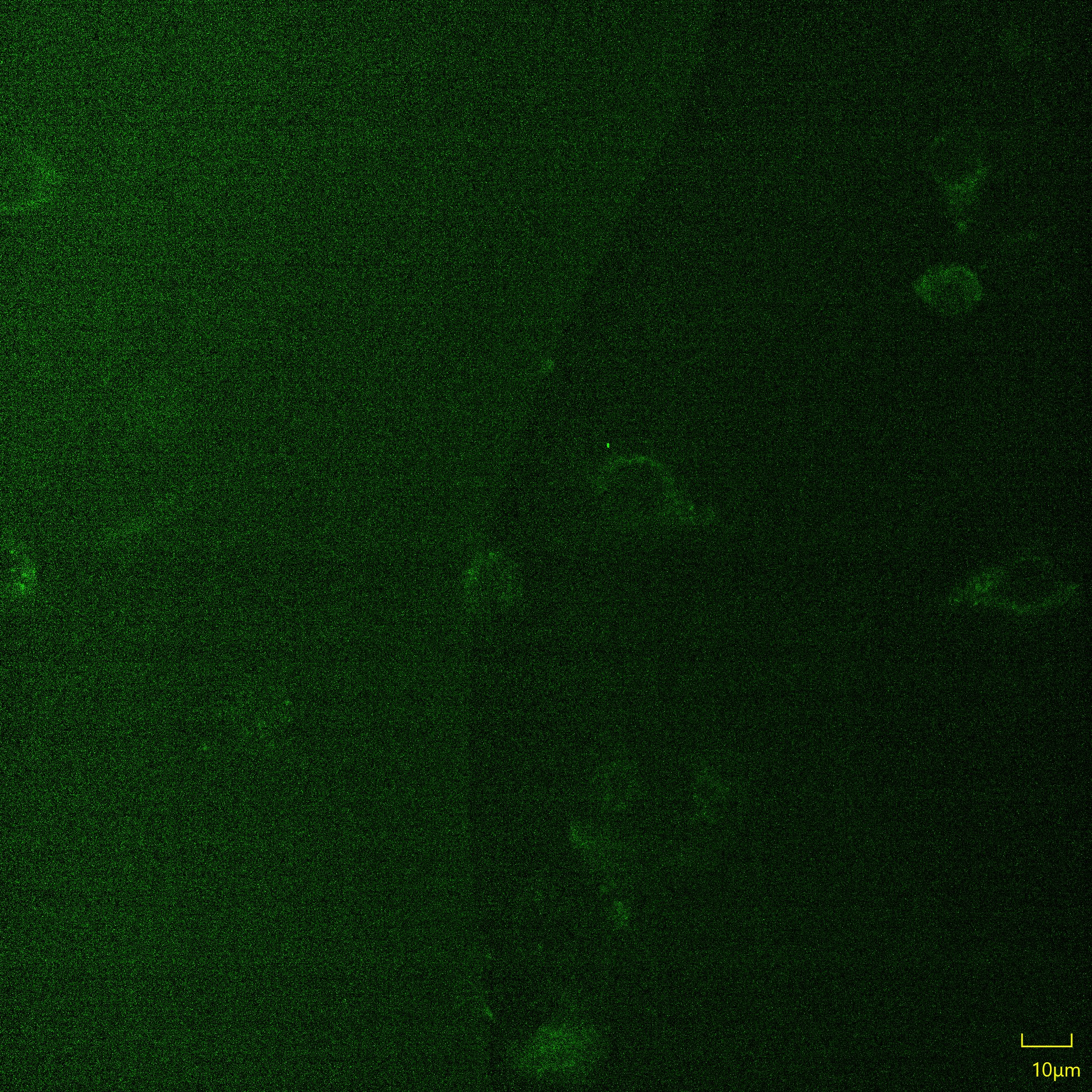

Supplement: Supplementary file 11 — Source data Fig. 10 [file 44318_2024_192_MOESM11_ESM.zip › Figure10/Figure10c/proliferation_cyto.jpg]

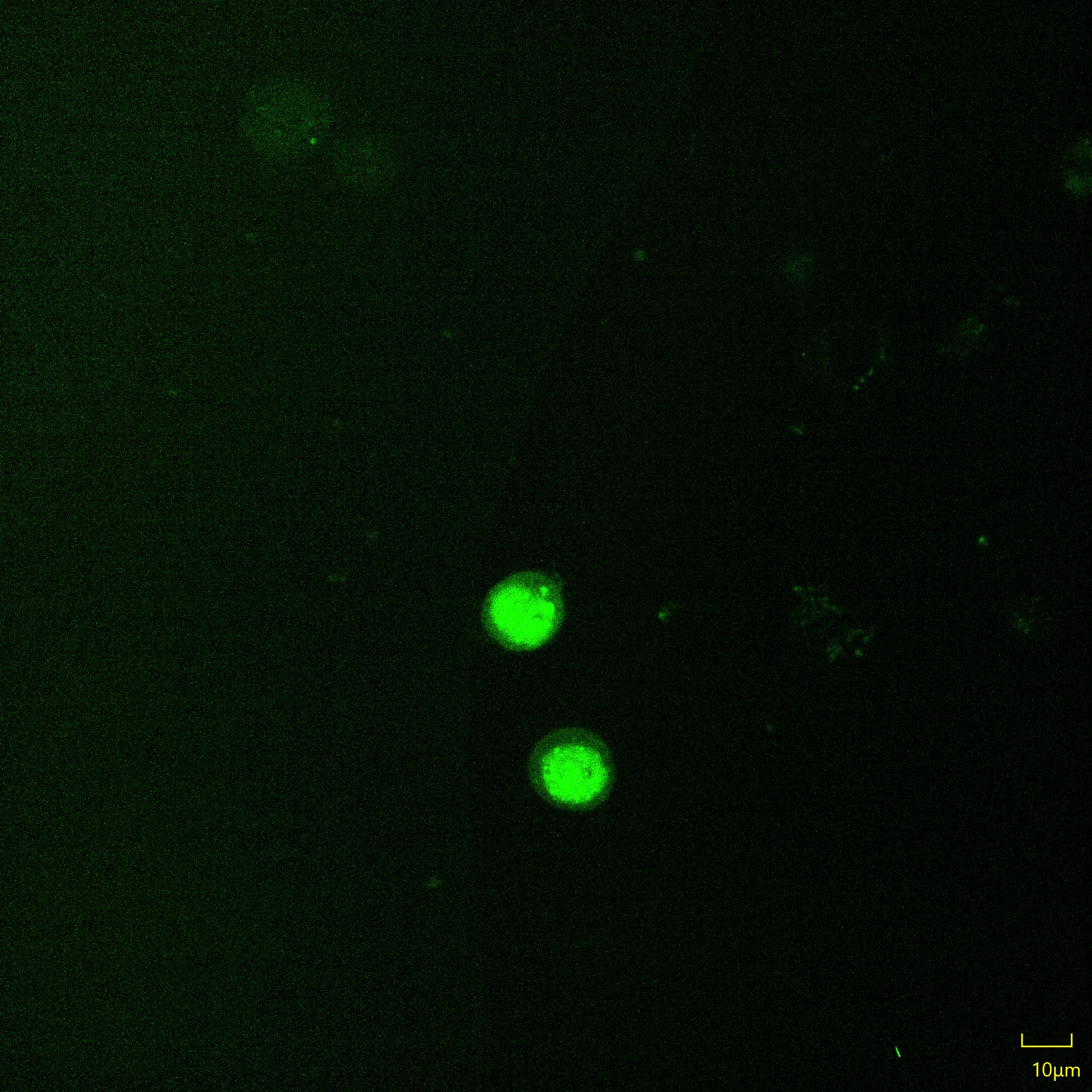

Supplement: Supplementary file 11 — Source data Fig. 10 [file 44318_2024_192_MOESM11_ESM.zip › Figure10/Figure10c/proliferation_nuc.jpg]

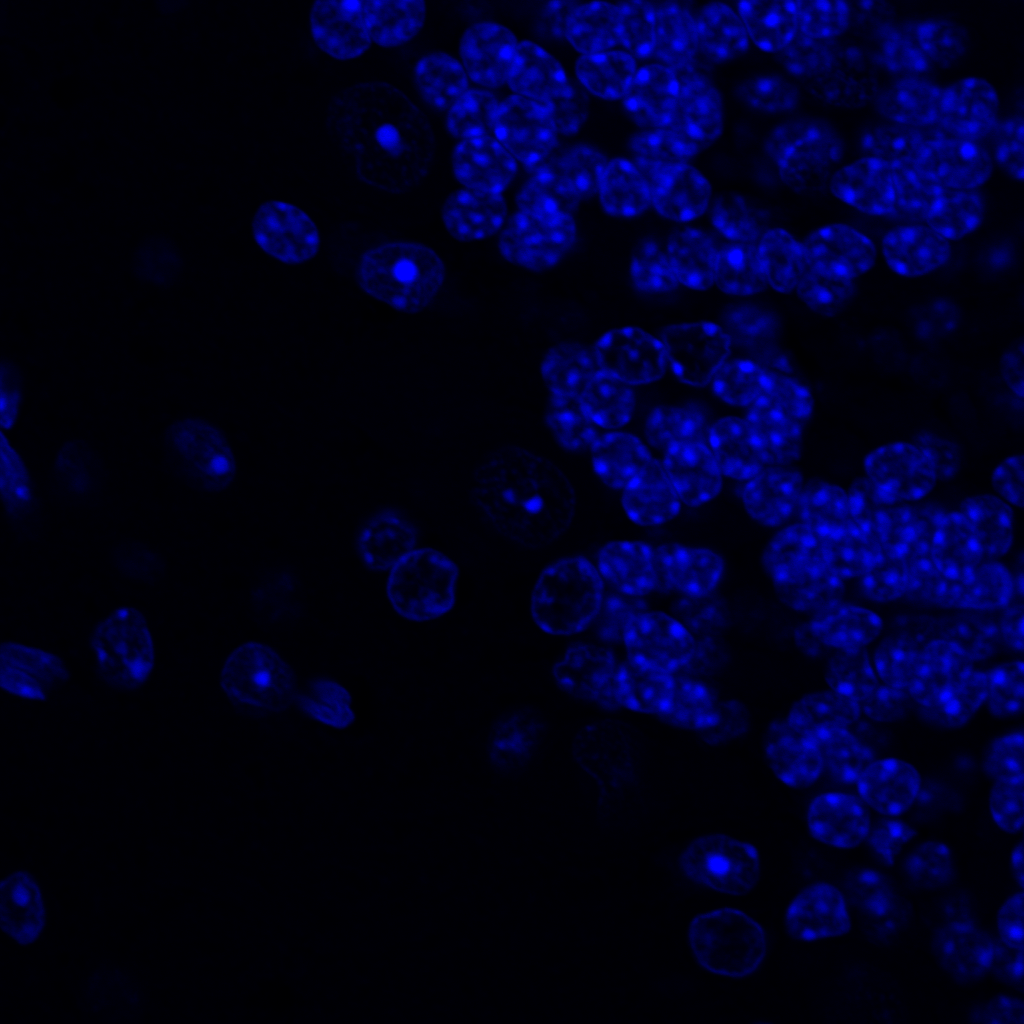

Supplement: Supplementary file 11 — Source data Fig. 10 [file 44318_2024_192_MOESM11_ESM.zip › Figure10/Figure10d/Atxn1-KI, 9weeks/enlarge_DAPI.tif]

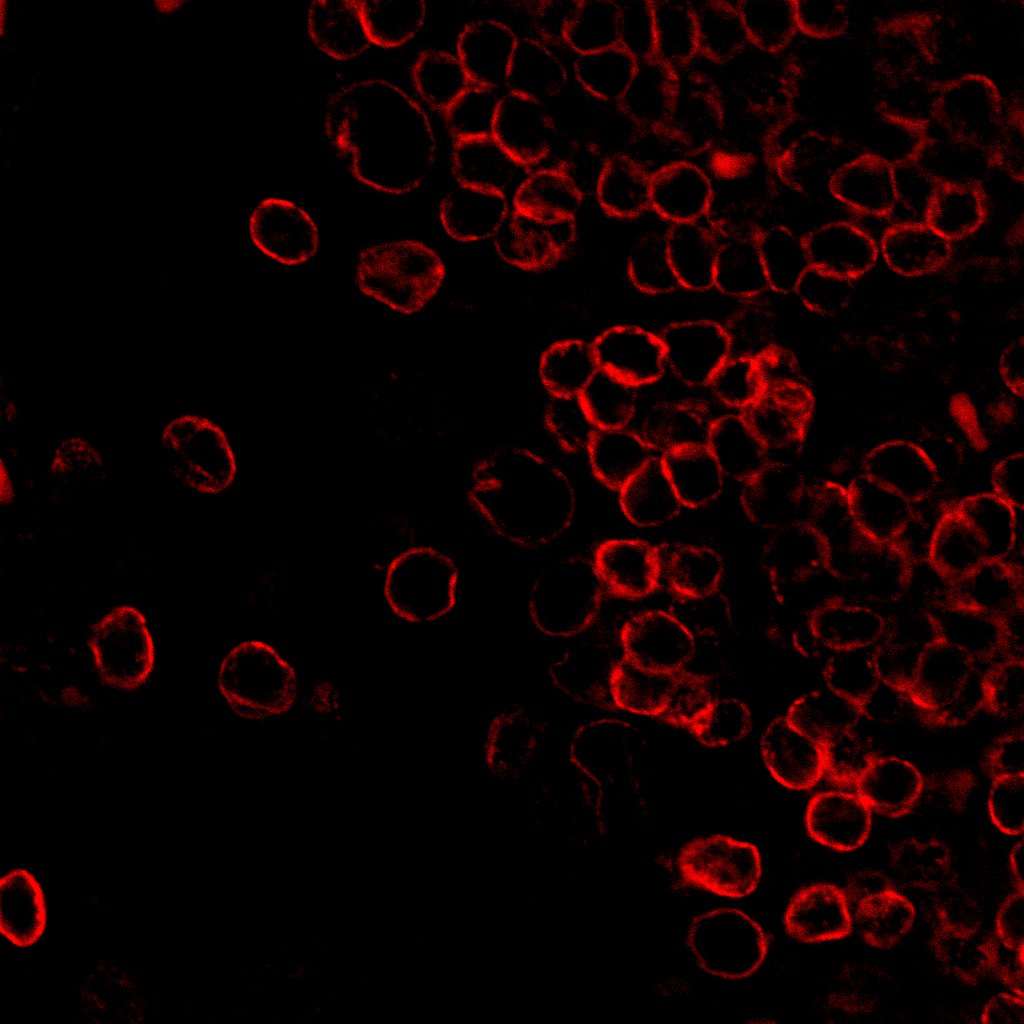

Supplement: Supplementary file 11 — Source data Fig. 10 [file 44318_2024_192_MOESM11_ESM.zip › Figure10/Figure10d/Atxn1-KI, 9weeks/enlarge_Lamin B1.tif]

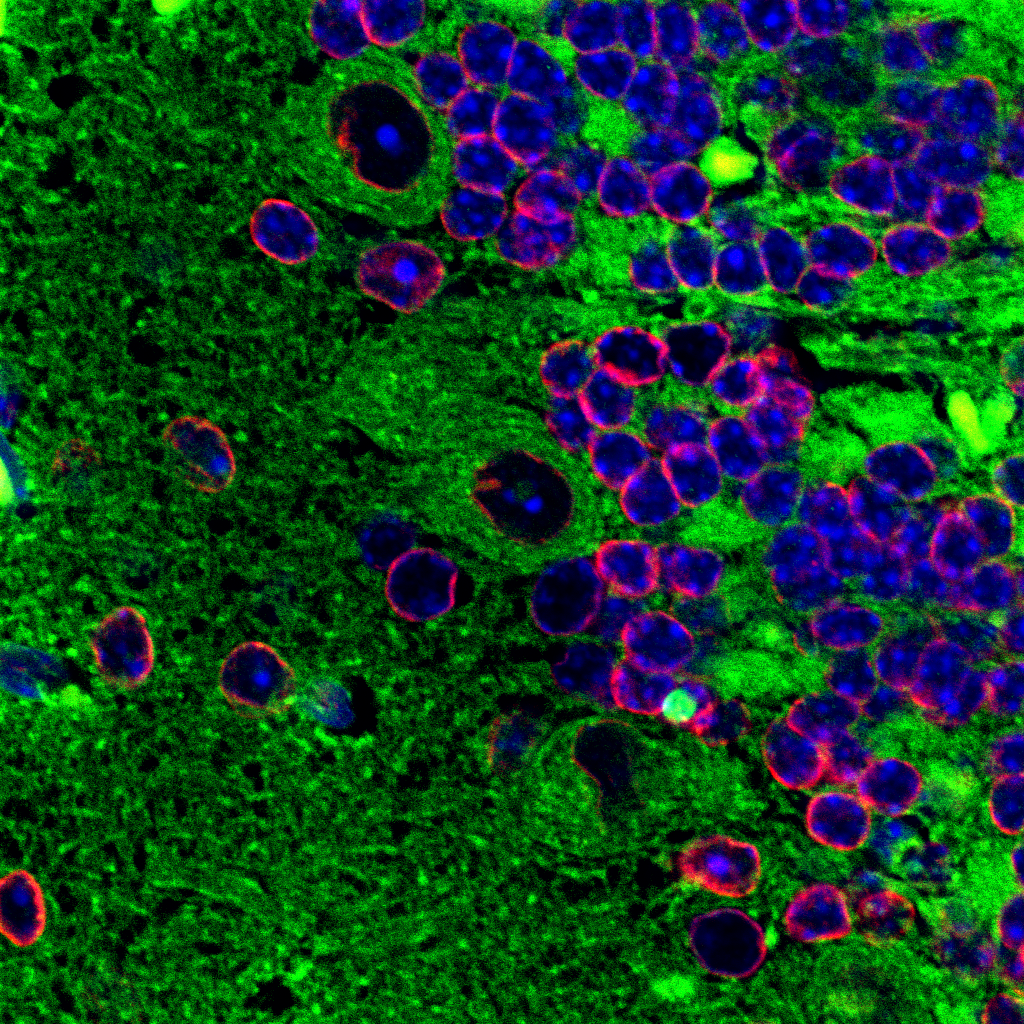

Supplement: Supplementary file 11 — Source data Fig. 10 [file 44318_2024_192_MOESM11_ESM.zip › Figure10/Figure10d/Atxn1-KI, 9weeks/enlarge_PQBP3+Lamin B1+DAPI.tif]

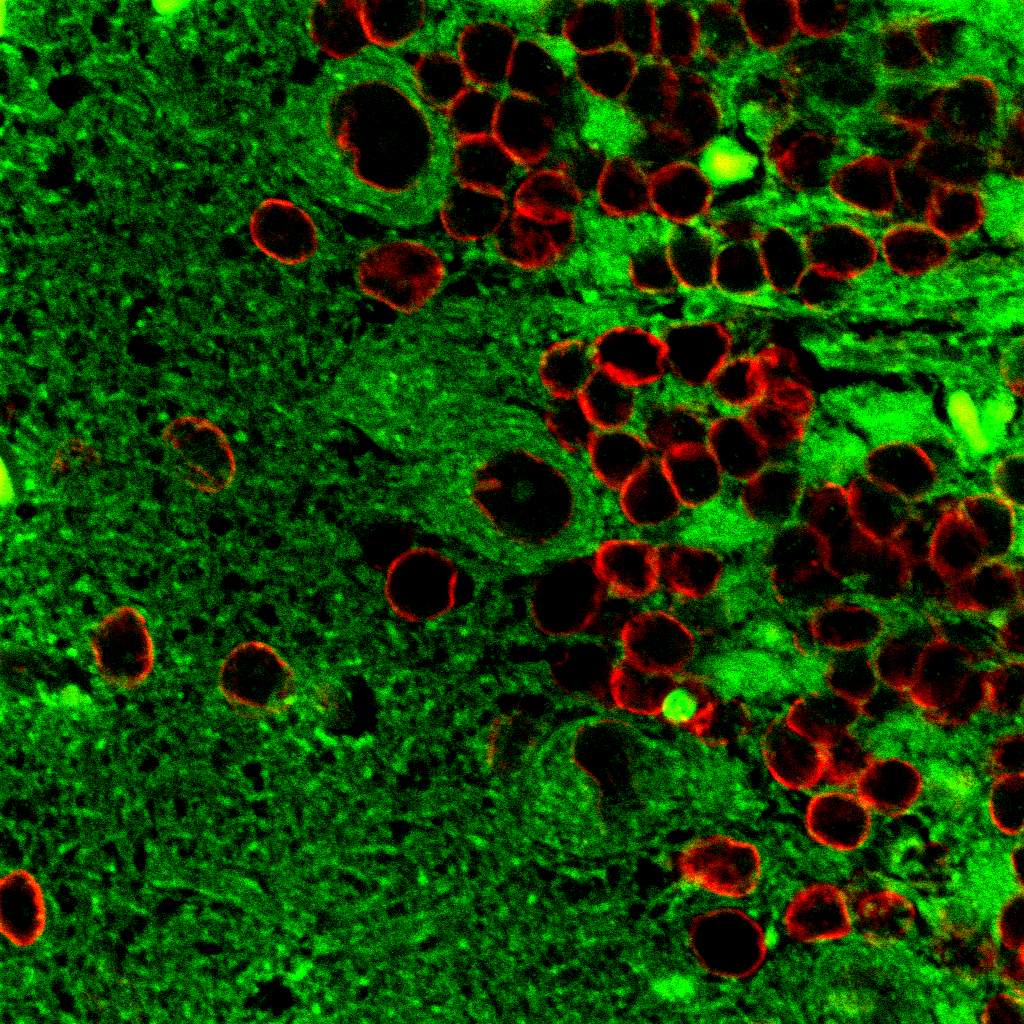

Supplement: Supplementary file 11 — Source data Fig. 10 [file 44318_2024_192_MOESM11_ESM.zip › Figure10/Figure10d/Atxn1-KI, 9weeks/enlarge_PQBP3+Lamin B1.tif]

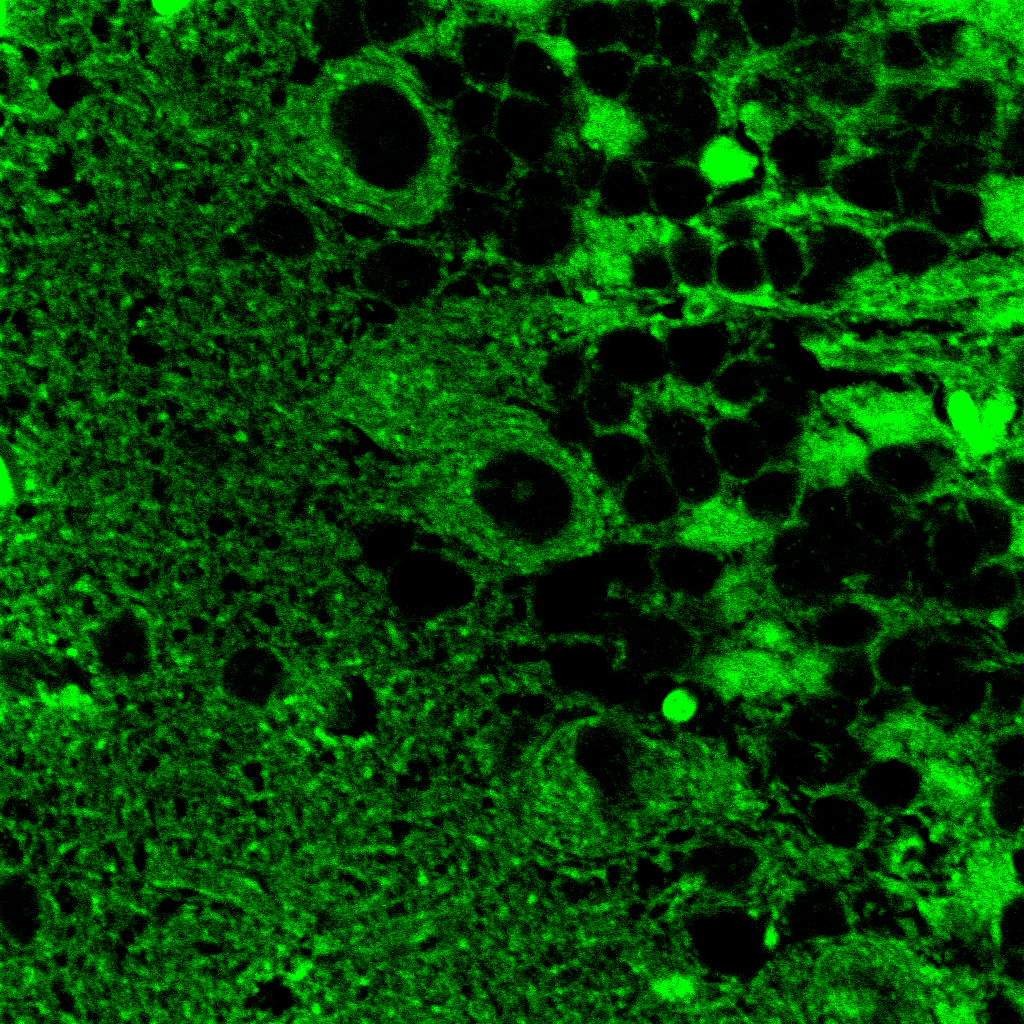

Supplement: Supplementary file 11 — Source data Fig. 10 [file 44318_2024_192_MOESM11_ESM.zip › Figure10/Figure10d/Atxn1-KI, 9weeks/enlarge_PQBP3.tif]

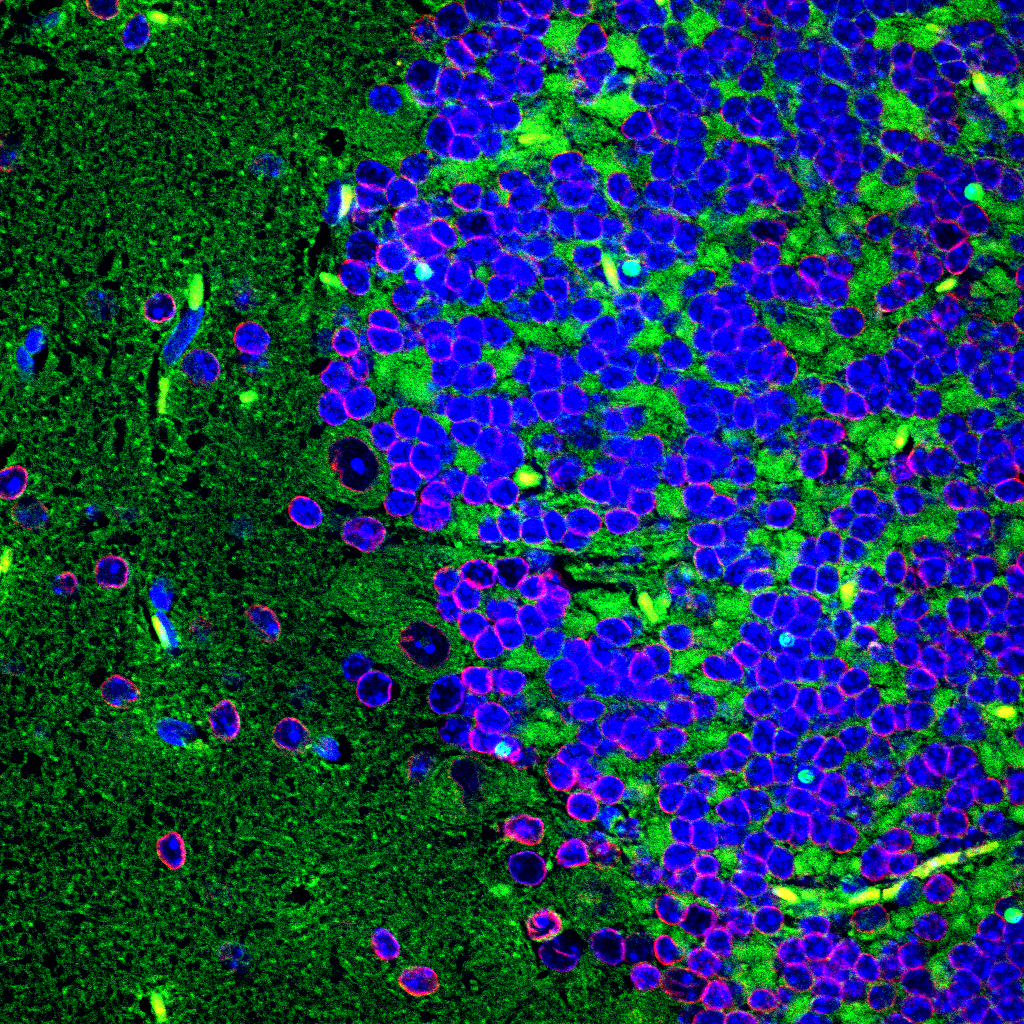

Supplement: Supplementary file 11 — Source data Fig. 10 [file 44318_2024_192_MOESM11_ESM.zip › Figure10/Figure10d/Atxn1-KI, 9weeks/PQBP3+Lamin B1+DAPI.tif]

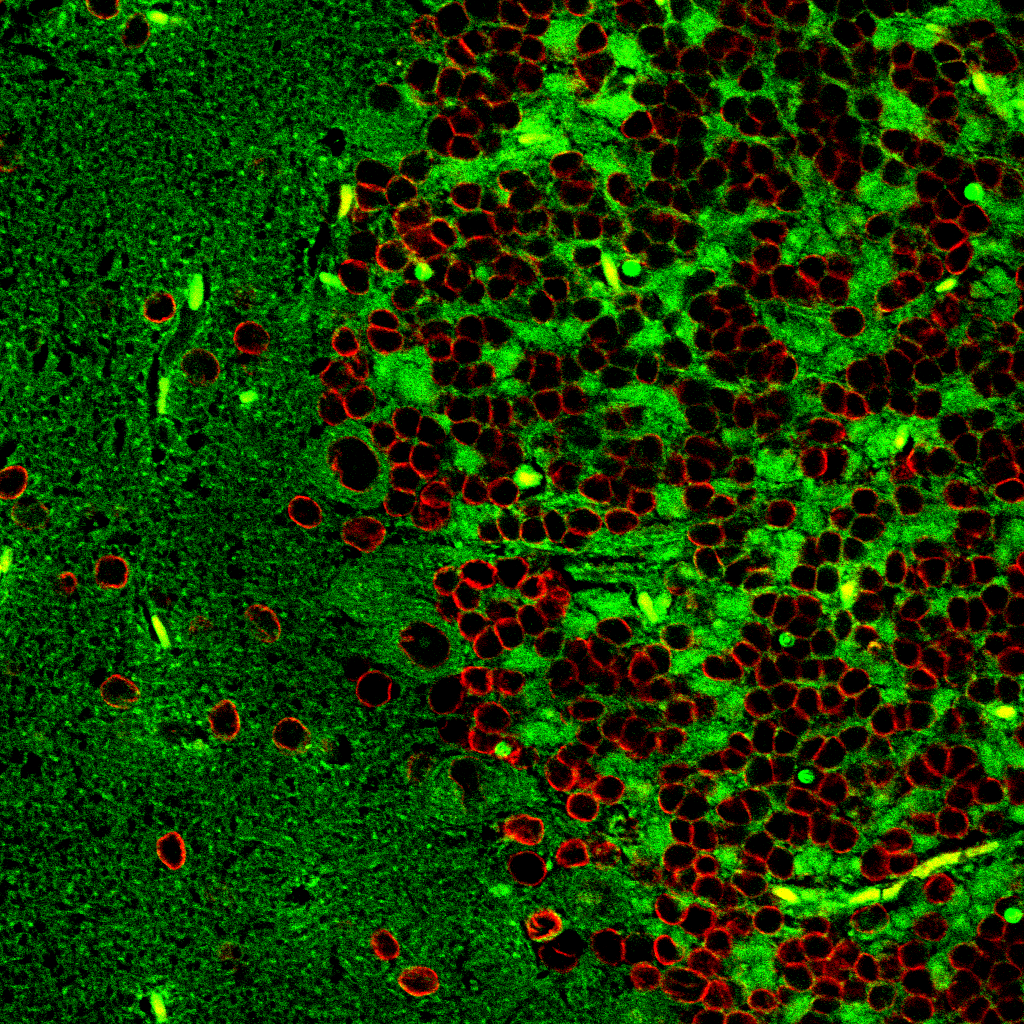

Supplement: Supplementary file 11 — Source data Fig. 10 [file 44318_2024_192_MOESM11_ESM.zip › Figure10/Figure10d/Atxn1-KI, 9weeks/PQBP3+Lamin B1.tif]

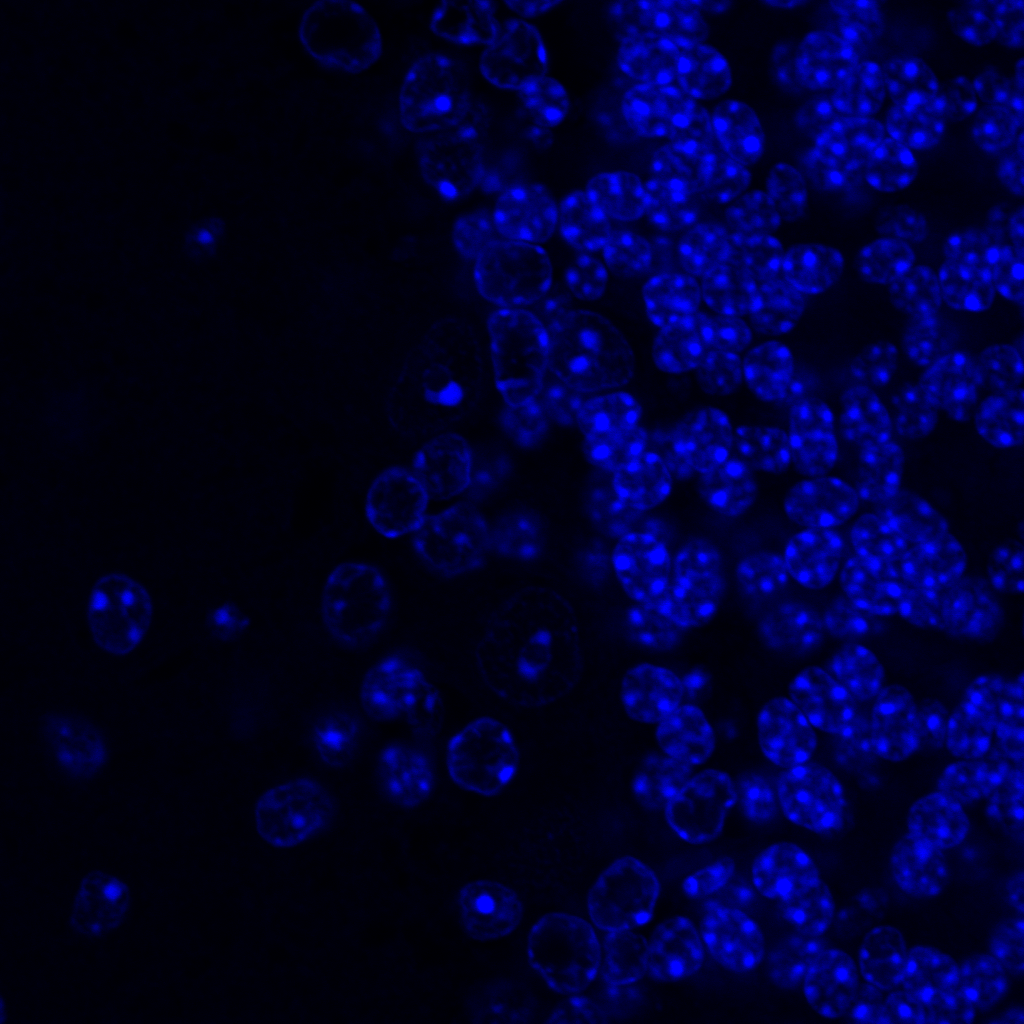

Supplement: Supplementary file 11 — Source data Fig. 10 [file 44318_2024_192_MOESM11_ESM.zip › Figure10/Figure10d/Sibling non-Tg (C57BL6), 9weeks/enlarge_DAPI.tif]

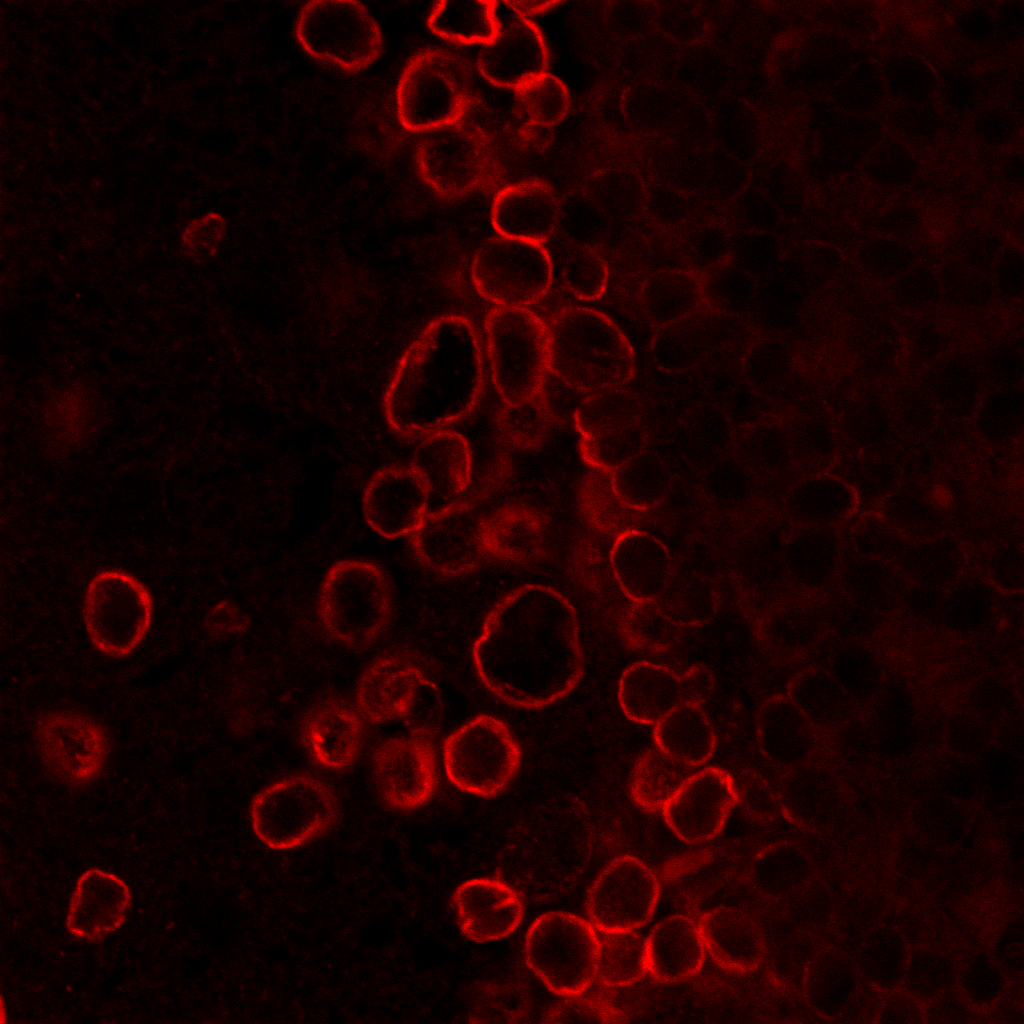

Supplement: Supplementary file 11 — Source data Fig. 10 [file 44318_2024_192_MOESM11_ESM.zip › Figure10/Figure10d/Sibling non-Tg (C57BL6), 9weeks/enlarge_Lamin B1.tif]

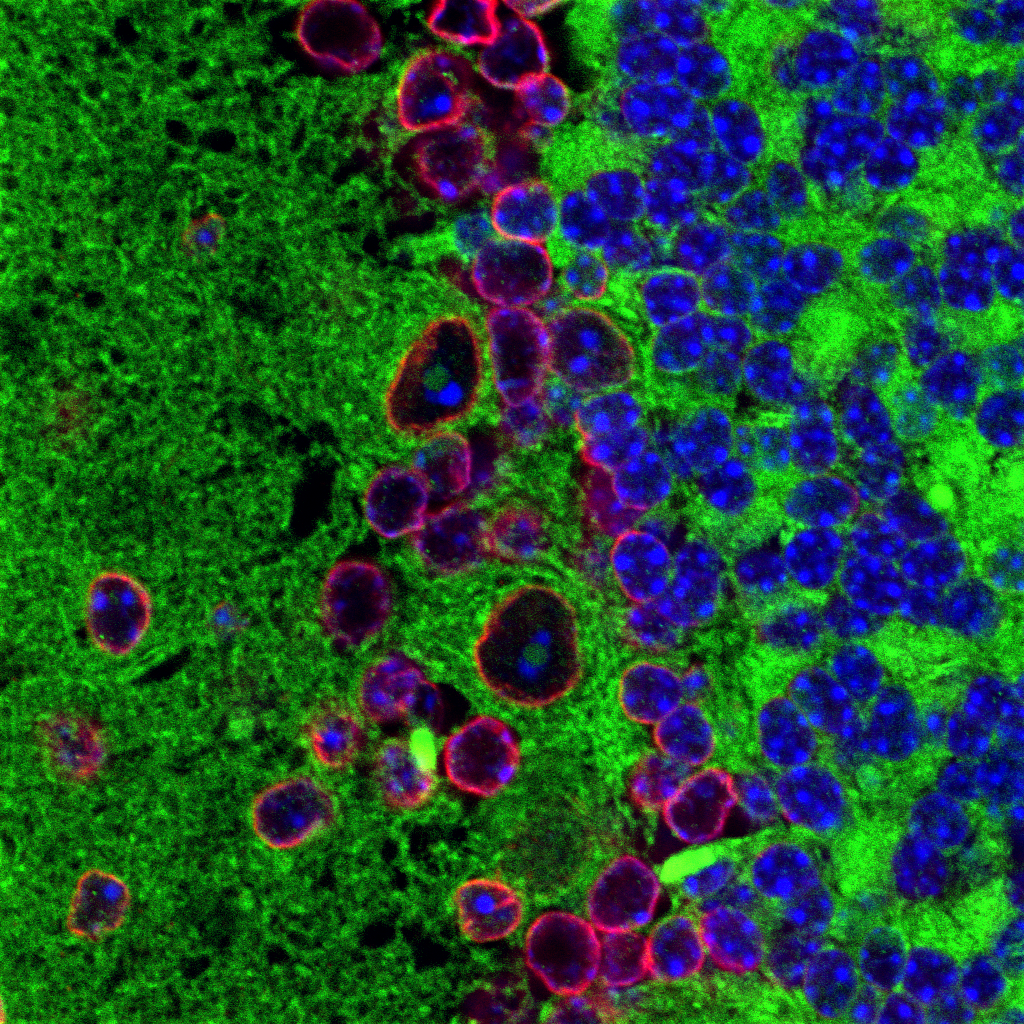

Supplement: Supplementary file 11 — Source data Fig. 10 [file 44318_2024_192_MOESM11_ESM.zip › Figure10/Figure10d/Sibling non-Tg (C57BL6), 9weeks/enlarge_PQBP3+Lamin B1+DAPI.tif]

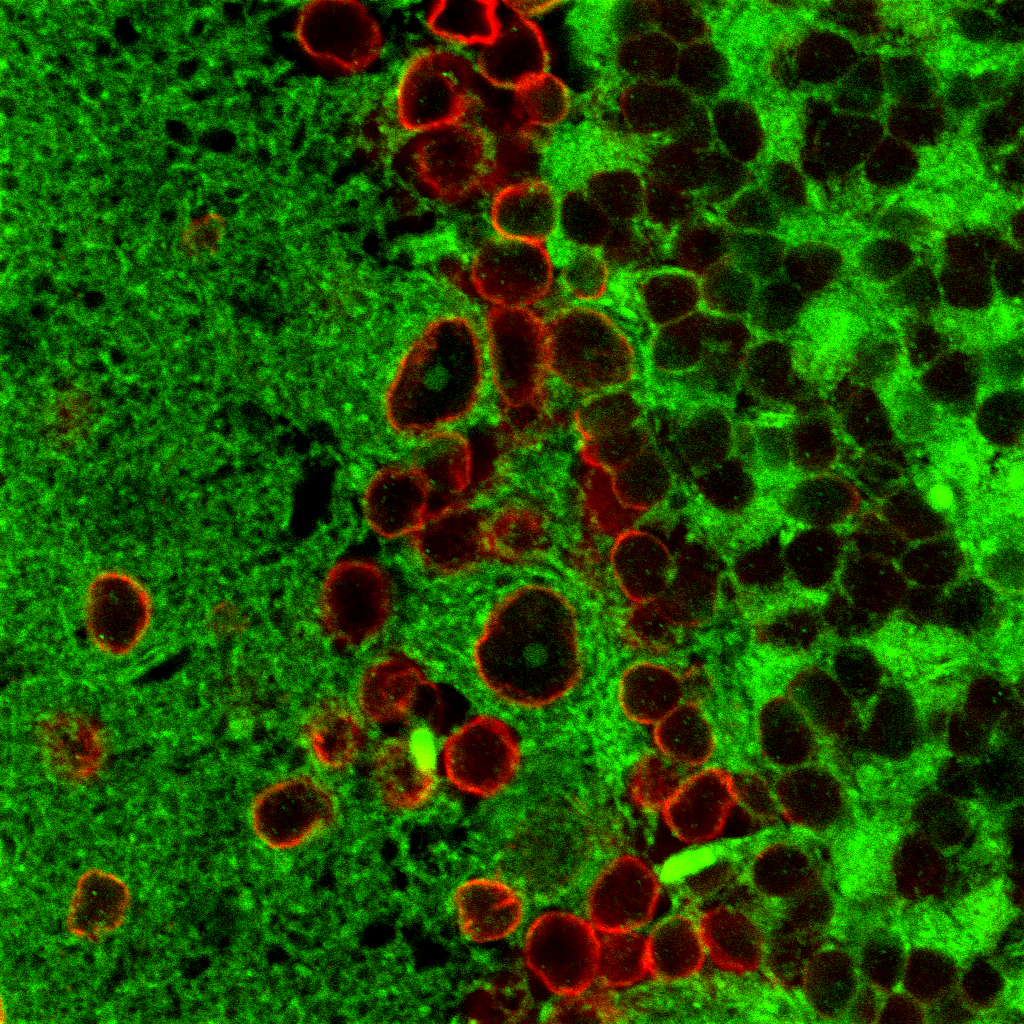

Supplement: Supplementary file 11 — Source data Fig. 10 [file 44318_2024_192_MOESM11_ESM.zip › Figure10/Figure10d/Sibling non-Tg (C57BL6), 9weeks/enlarge_PQBP3+Lamin B1.tif]

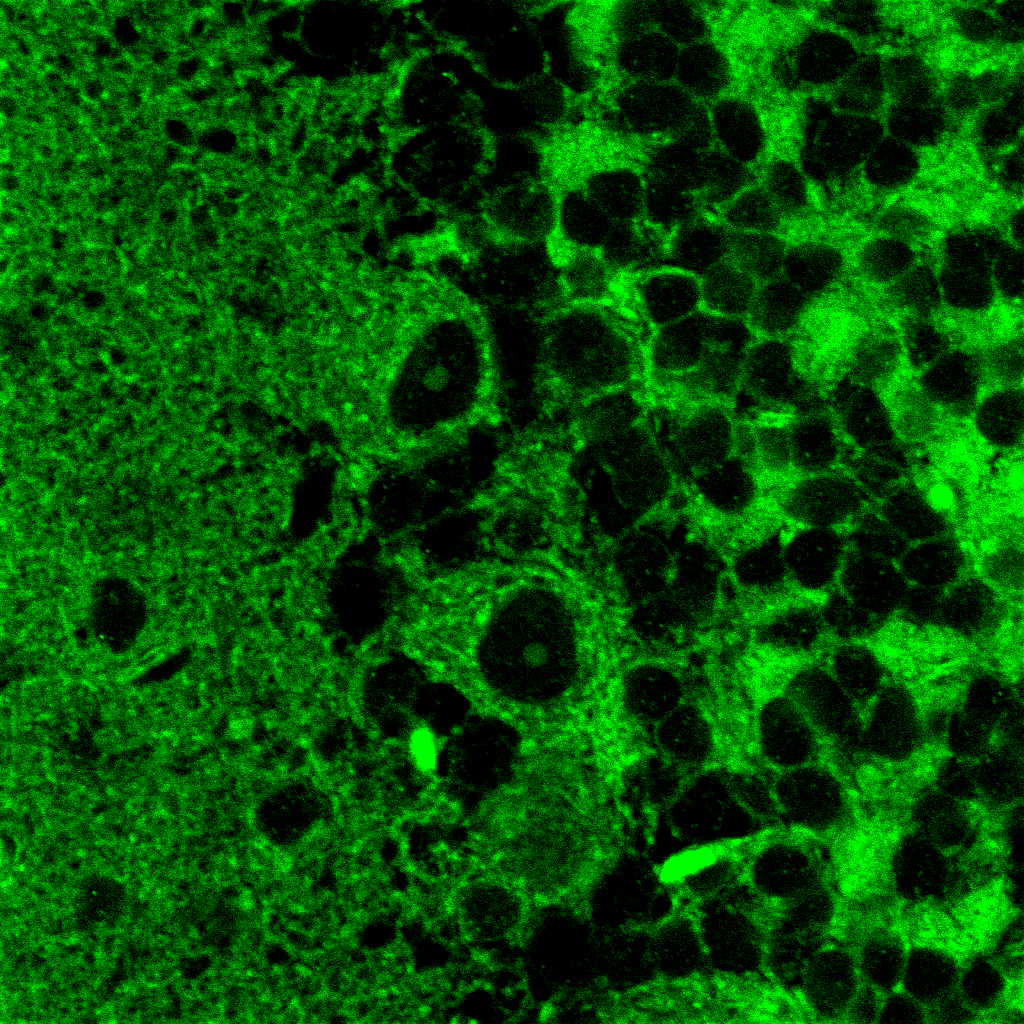

Supplement: Supplementary file 11 — Source data Fig. 10 [file 44318_2024_192_MOESM11_ESM.zip › Figure10/Figure10d/Sibling non-Tg (C57BL6), 9weeks/enlarge_PQBP3.tif]

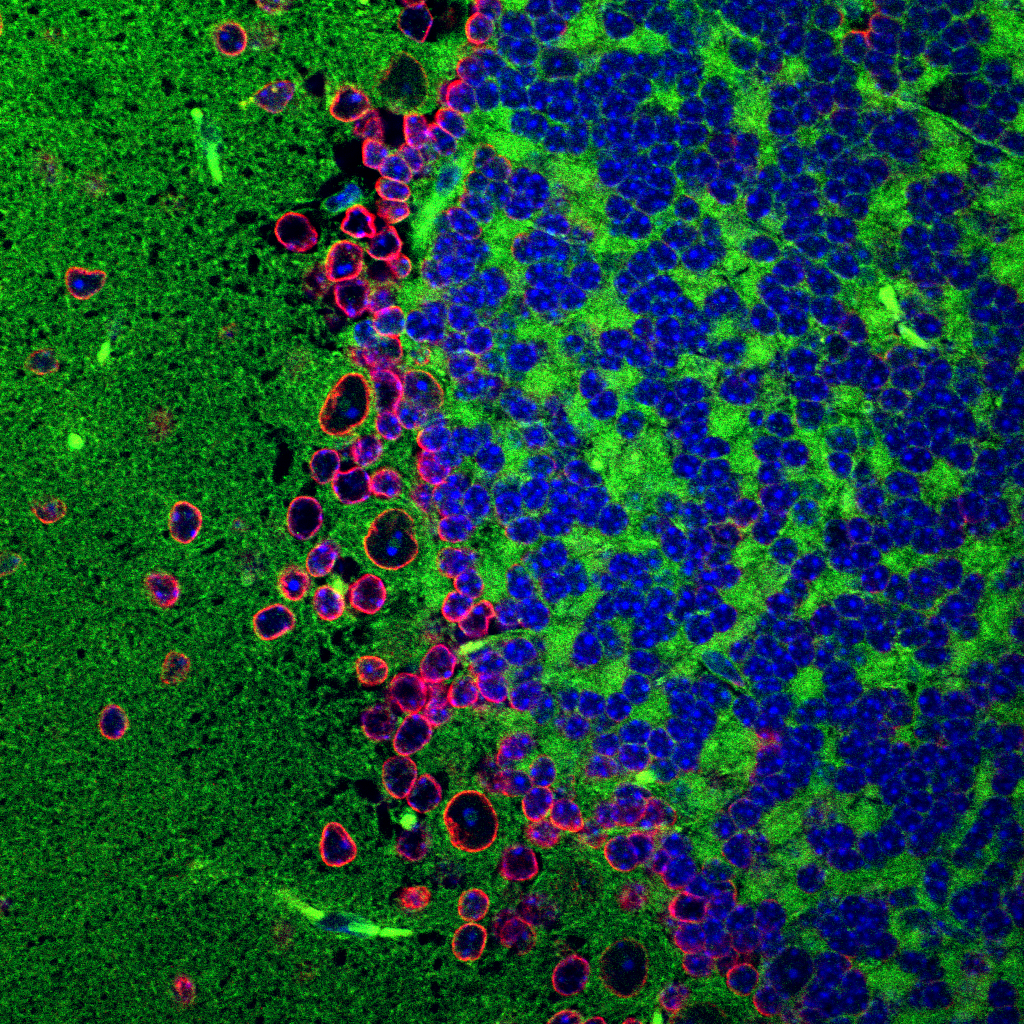

Supplement: Supplementary file 11 — Source data Fig. 10 [file 44318_2024_192_MOESM11_ESM.zip › Figure10/Figure10d/Sibling non-Tg (C57BL6), 9weeks/PQBP3+Lamin B1+DAPI.tif]

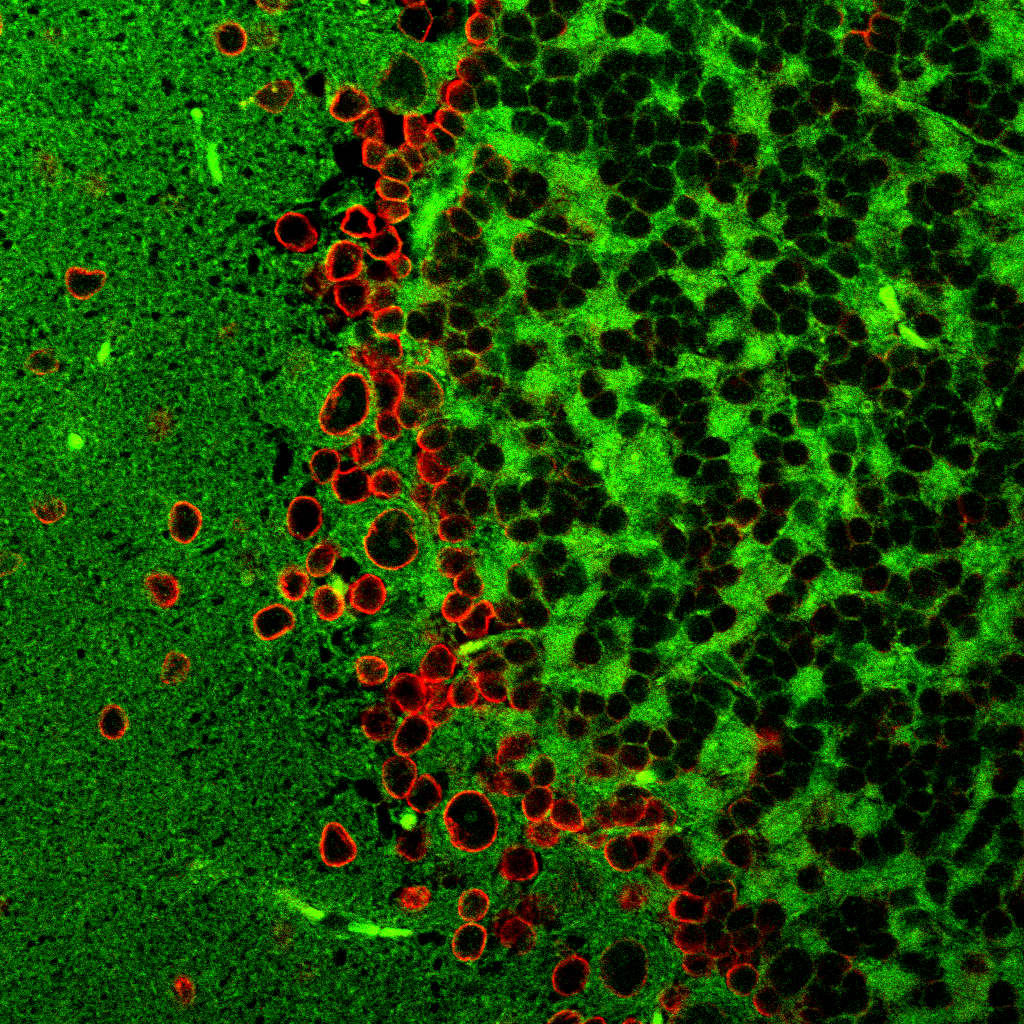

Supplement: Supplementary file 11 — Source data Fig. 10 [file 44318_2024_192_MOESM11_ESM.zip › Figure10/Figure10d/Sibling non-Tg (C57BL6), 9weeks/PQBP3+Lamin B1.tif]

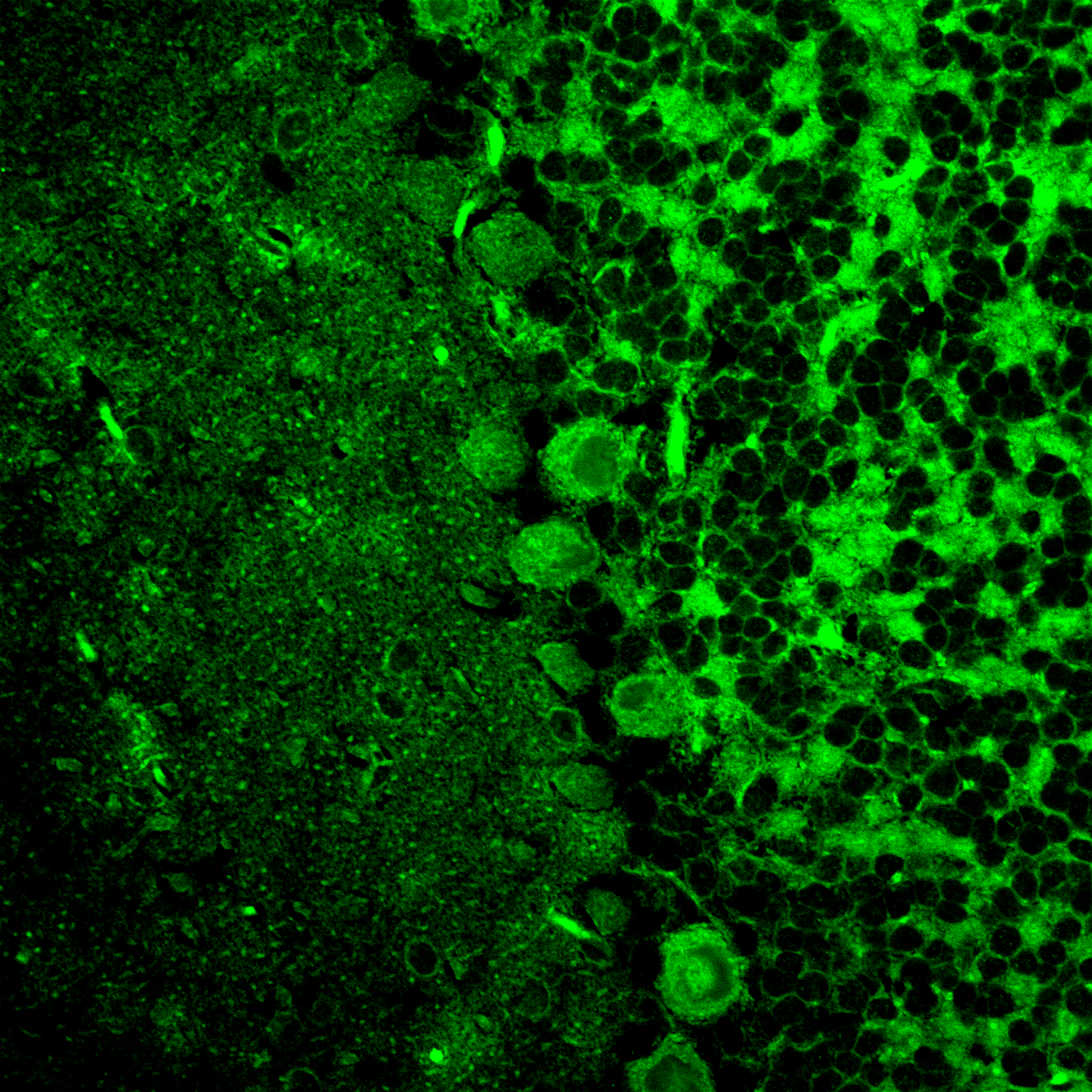

Supplement: Supplementary file 11 — Source data Fig. 10 [file 44318_2024_192_MOESM11_ESM.zip › Figure10/Figure10e/Atxn1-KI, 9weeks_Atxn1+PSME3/Atxn1.tif]

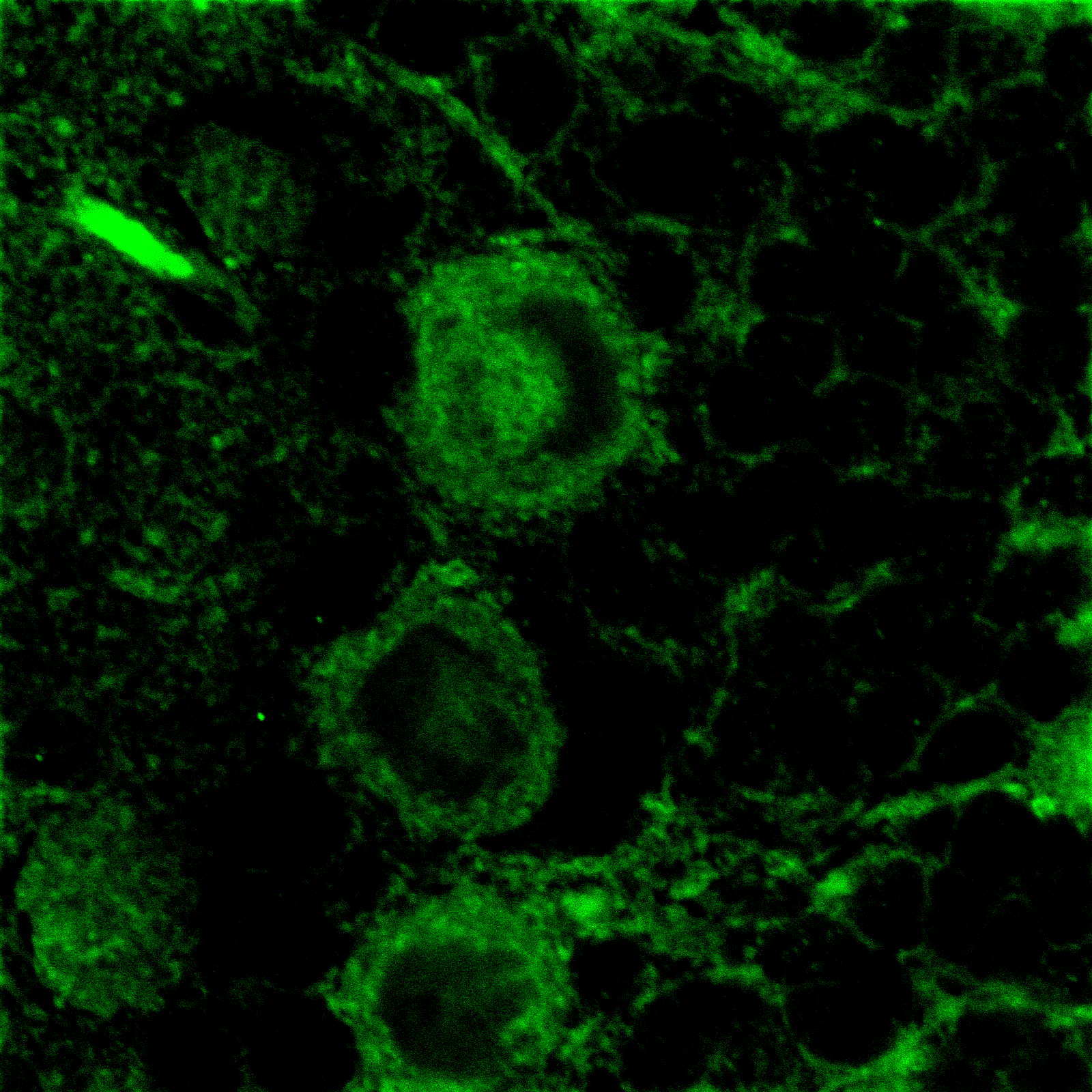

Supplement: Supplementary file 11 — Source data Fig. 10 [file 44318_2024_192_MOESM11_ESM.zip › Figure10/Figure10e/Atxn1-KI, 9weeks_Atxn1+PSME3/enlarge_Atxn1.tif]

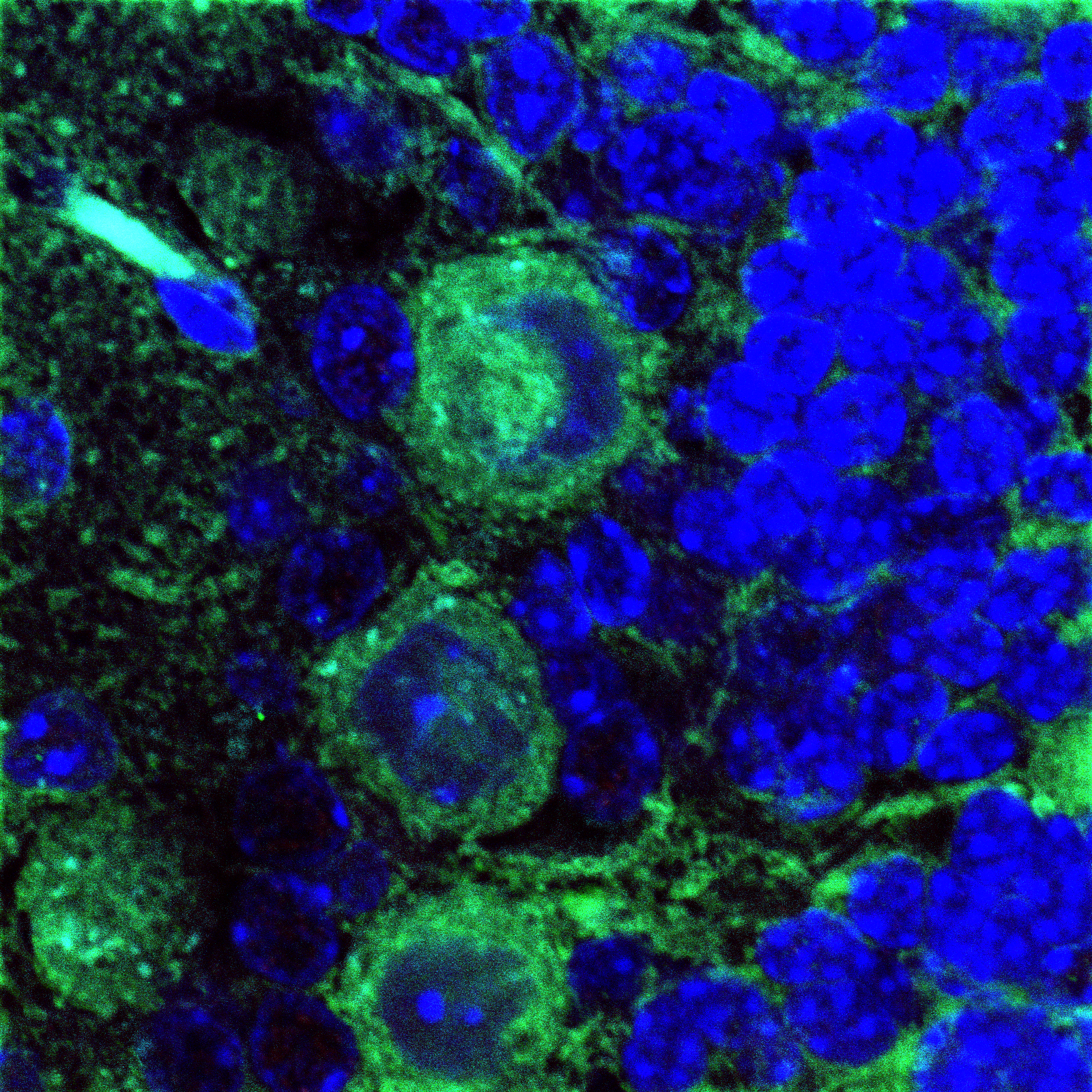

Supplement: Supplementary file 11 — Source data Fig. 10 [file 44318_2024_192_MOESM11_ESM.zip › Figure10/Figure10e/Atxn1-KI, 9weeks_Atxn1+PSME3/enlarge_Merge.tif]

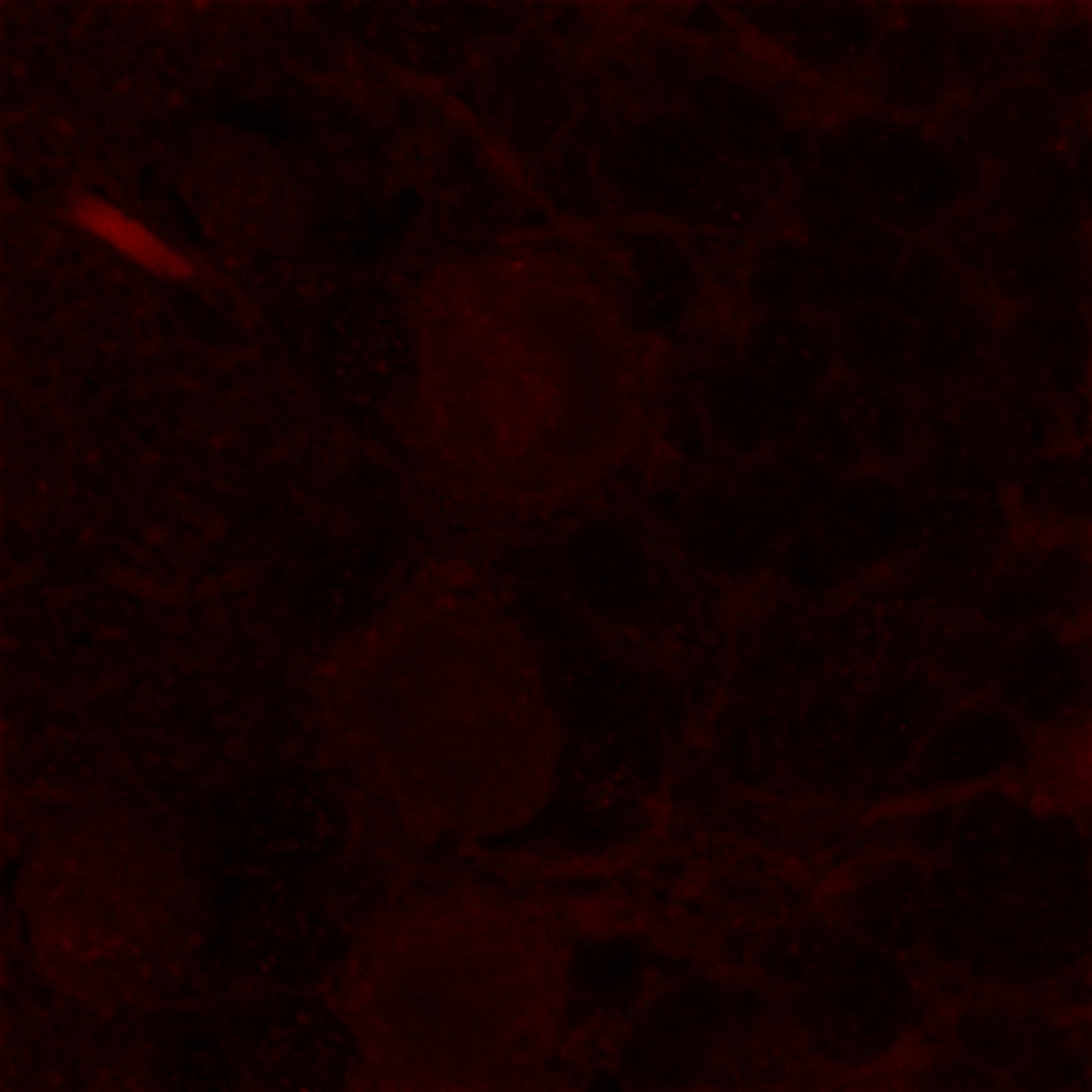

Supplement: Supplementary file 11 — Source data Fig. 10 [file 44318_2024_192_MOESM11_ESM.zip › Figure10/Figure10e/Atxn1-KI, 9weeks_Atxn1+PSME3/enlarge_PSME3.tif]

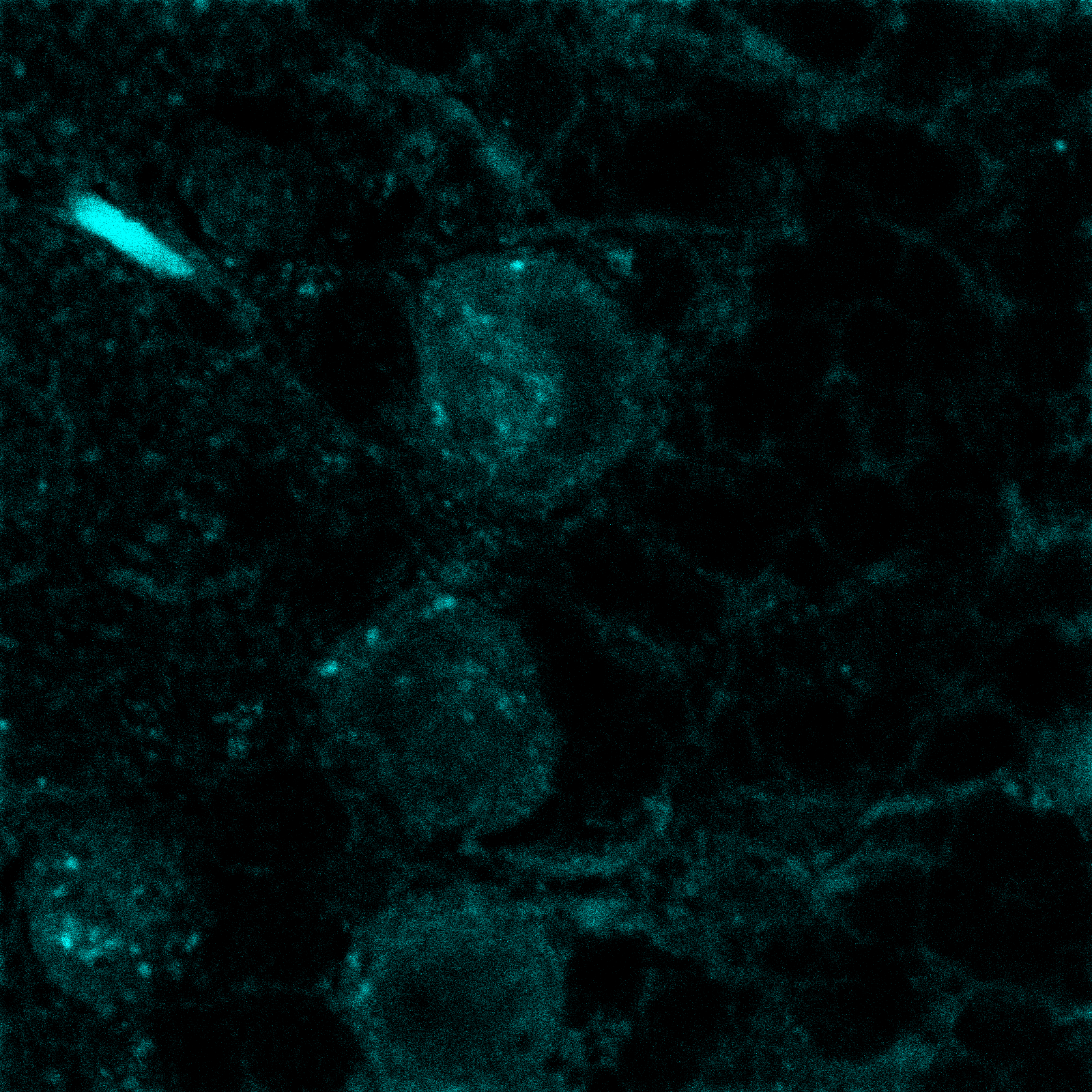

Supplement: Supplementary file 11 — Source data Fig. 10 [file 44318_2024_192_MOESM11_ESM.zip › Figure10/Figure10e/Atxn1-KI, 9weeks_Atxn1+PSME3/enlarge_ubiquitin.tif]

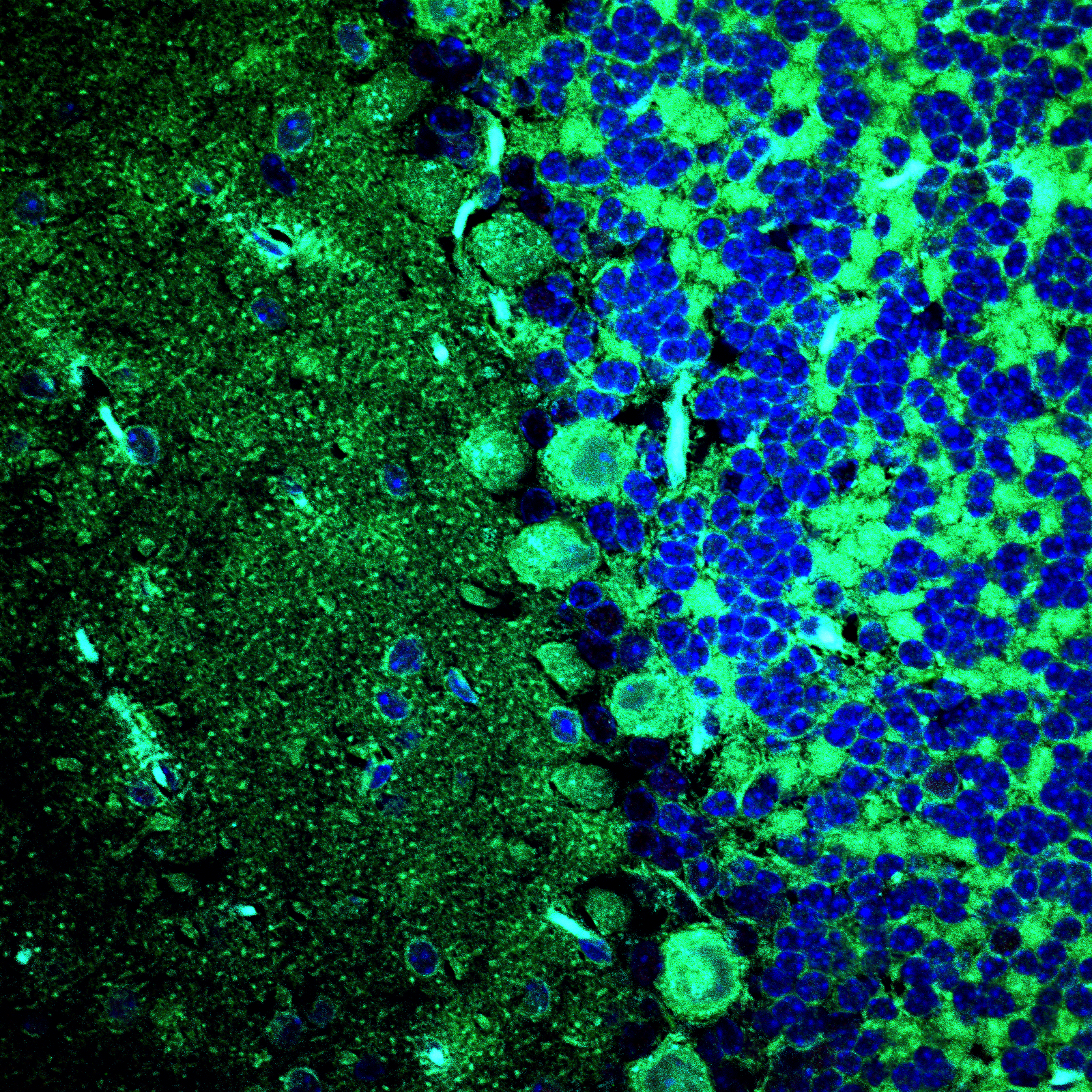

Supplement: Supplementary file 11 — Source data Fig. 10 [file 44318_2024_192_MOESM11_ESM.zip › Figure10/Figure10e/Atxn1-KI, 9weeks_Atxn1+PSME3/Merge.tif]

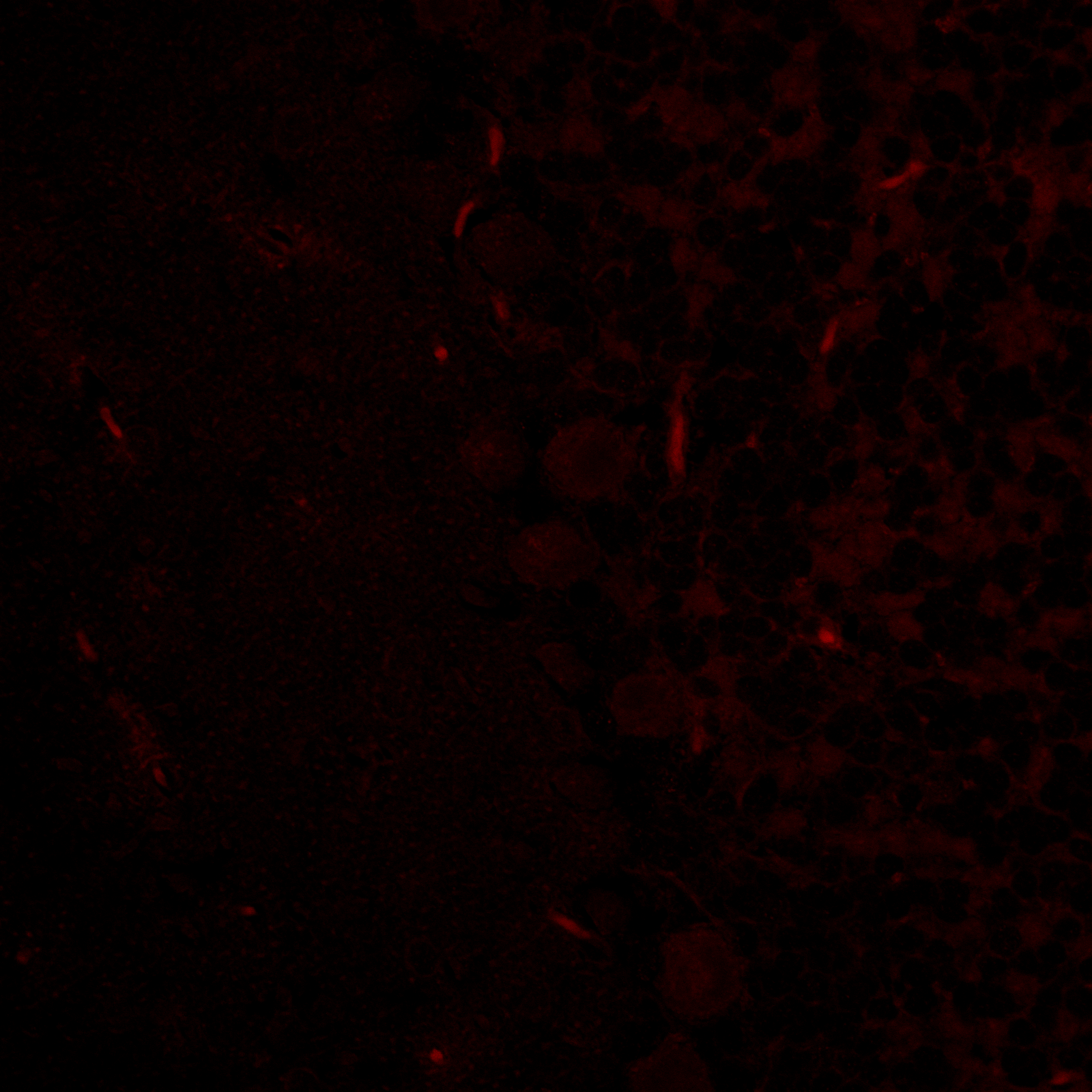

Supplement: Supplementary file 11 — Source data Fig. 10 [file 44318_2024_192_MOESM11_ESM.zip › Figure10/Figure10e/Atxn1-KI, 9weeks_Atxn1+PSME3/PSME3.tif]

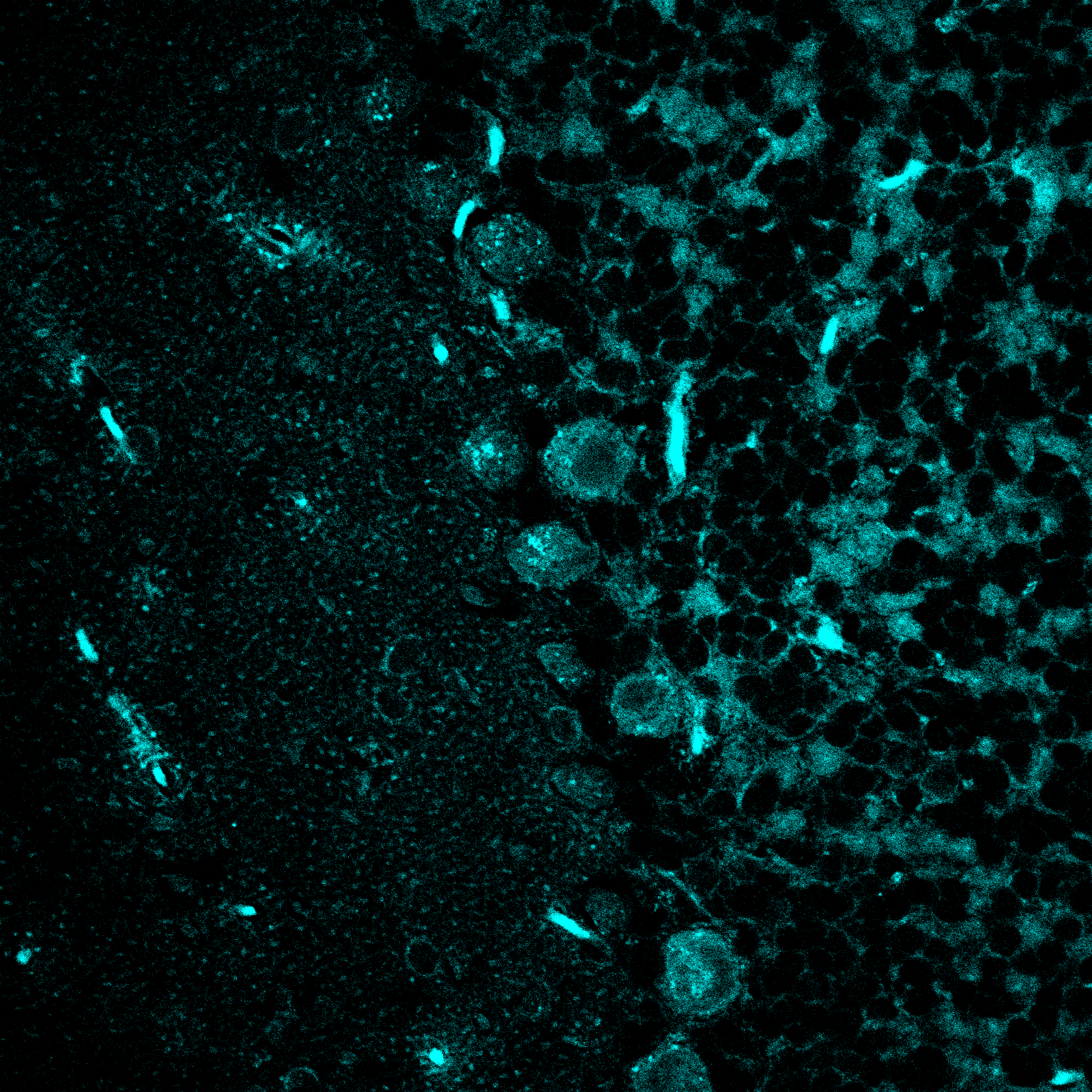

Supplement: Supplementary file 11 — Source data Fig. 10 [file 44318_2024_192_MOESM11_ESM.zip › Figure10/Figure10e/Atxn1-KI, 9weeks_Atxn1+PSME3/ubiquitin.tif]

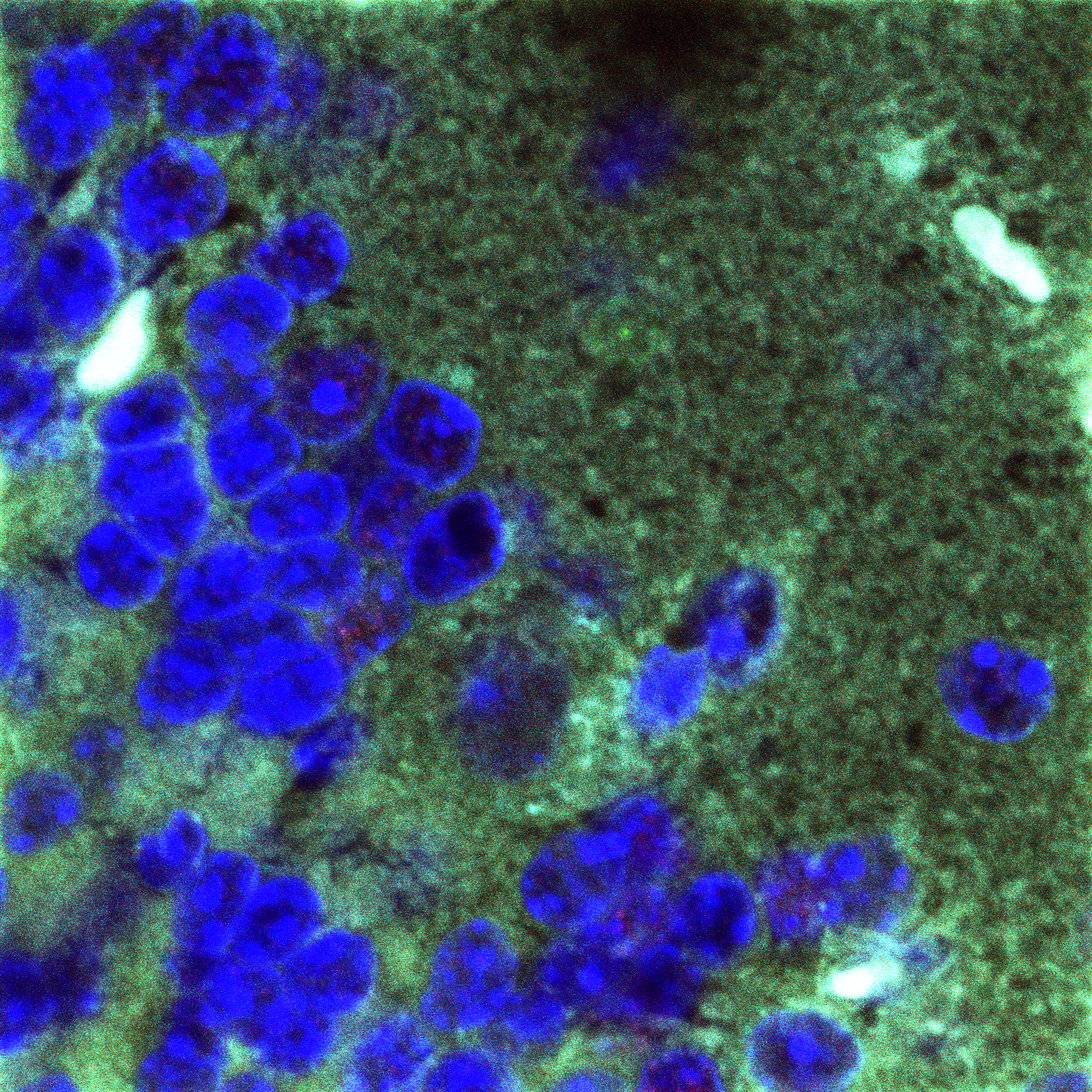

Supplement: Supplementary file 11 — Source data Fig. 10 [file 44318_2024_192_MOESM11_ESM.zip › Figure10/Figure10e/Atxn1-KI, 9weeks_PQBP3+PSME3/enlarge_Merge.tif]

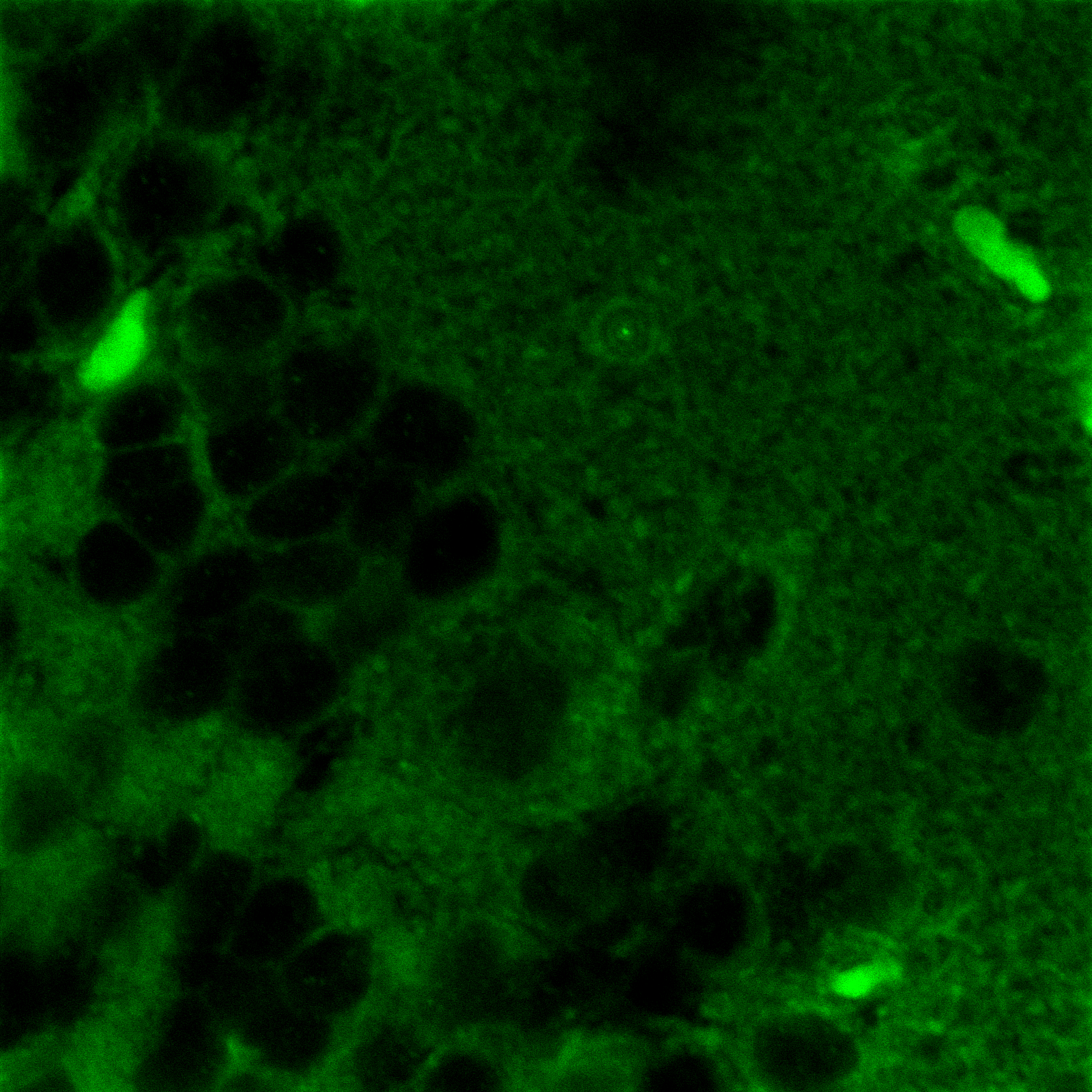

Supplement: Supplementary file 11 — Source data Fig. 10 [file 44318_2024_192_MOESM11_ESM.zip › Figure10/Figure10e/Atxn1-KI, 9weeks_PQBP3+PSME3/enlarge_PQBP3.tif]

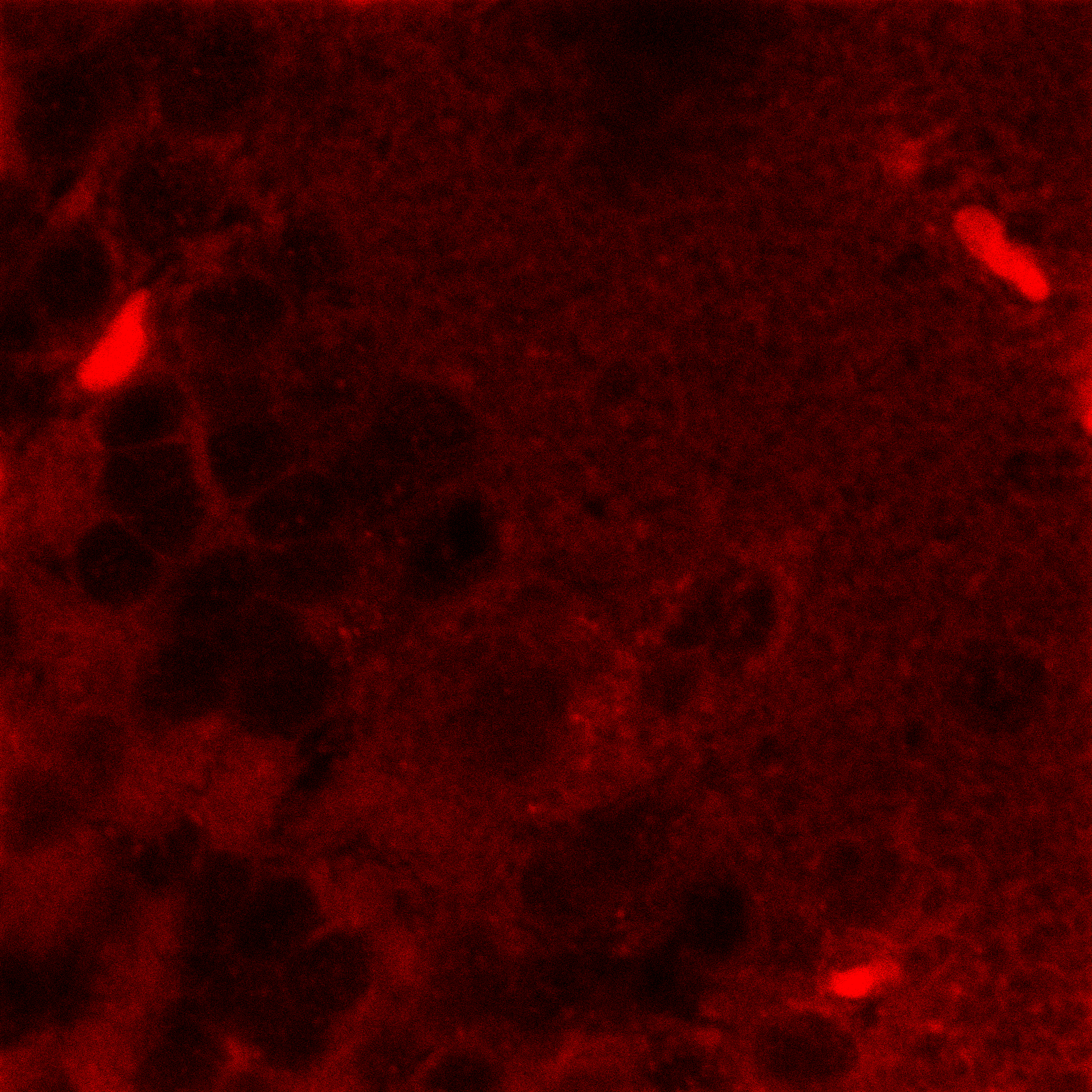

Supplement: Supplementary file 11 — Source data Fig. 10 [file 44318_2024_192_MOESM11_ESM.zip › Figure10/Figure10e/Atxn1-KI, 9weeks_PQBP3+PSME3/enlarge_PSME3.tif]

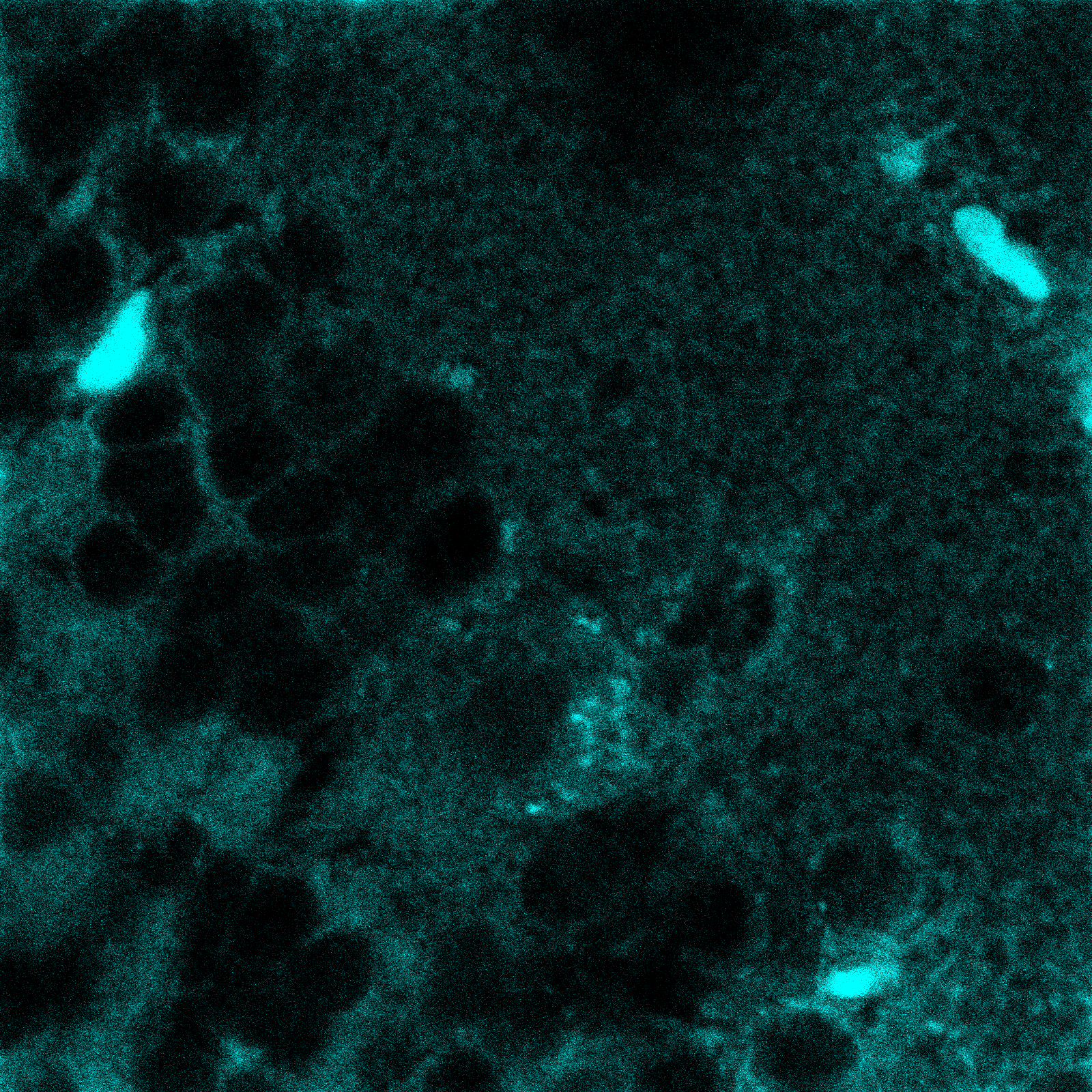

Supplement: Supplementary file 11 — Source data Fig. 10 [file 44318_2024_192_MOESM11_ESM.zip › Figure10/Figure10e/Atxn1-KI, 9weeks_PQBP3+PSME3/enlarge_ubiquitin.tif]

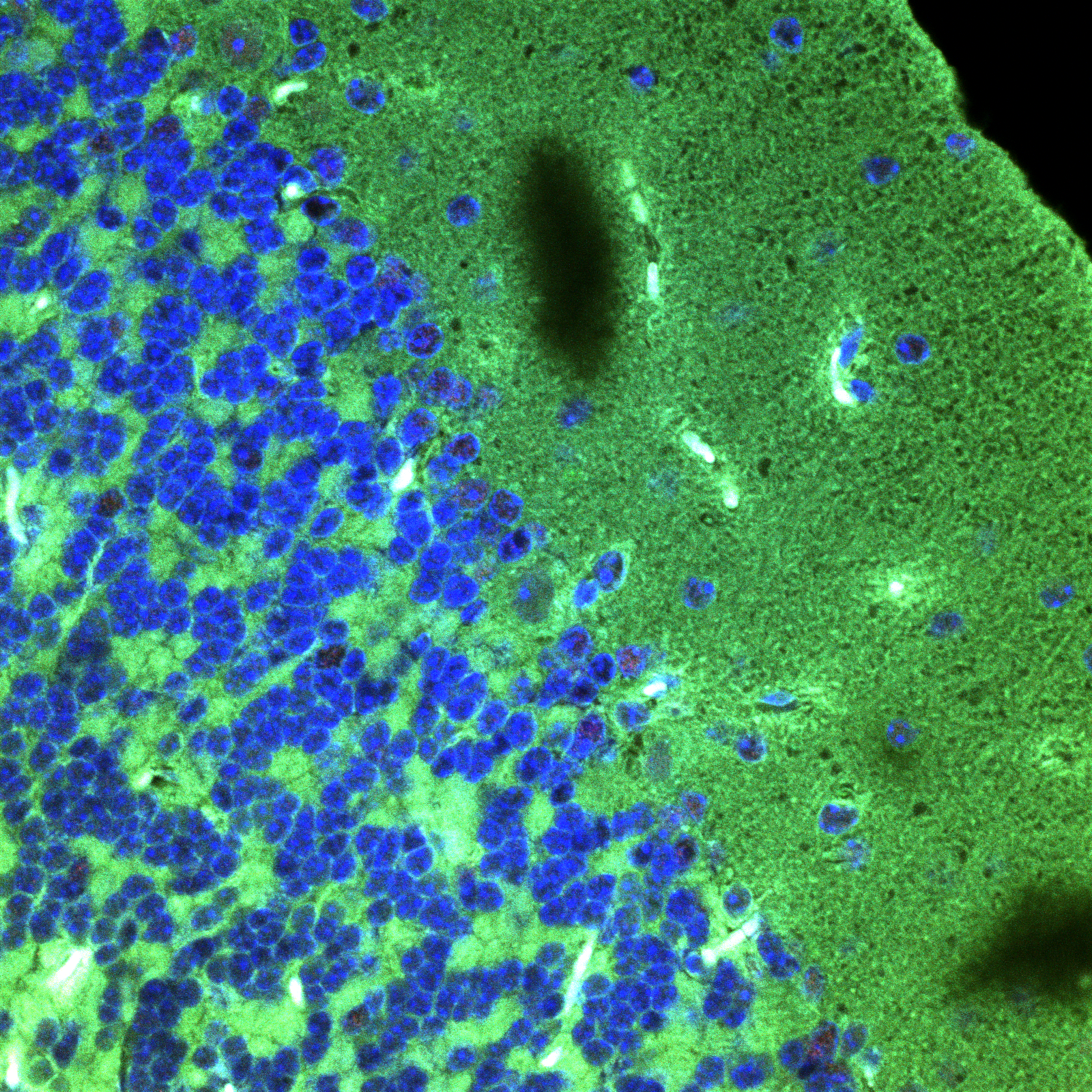

Supplement: Supplementary file 11 — Source data Fig. 10 [file 44318_2024_192_MOESM11_ESM.zip › Figure10/Figure10e/Atxn1-KI, 9weeks_PQBP3+PSME3/Merge.tif]

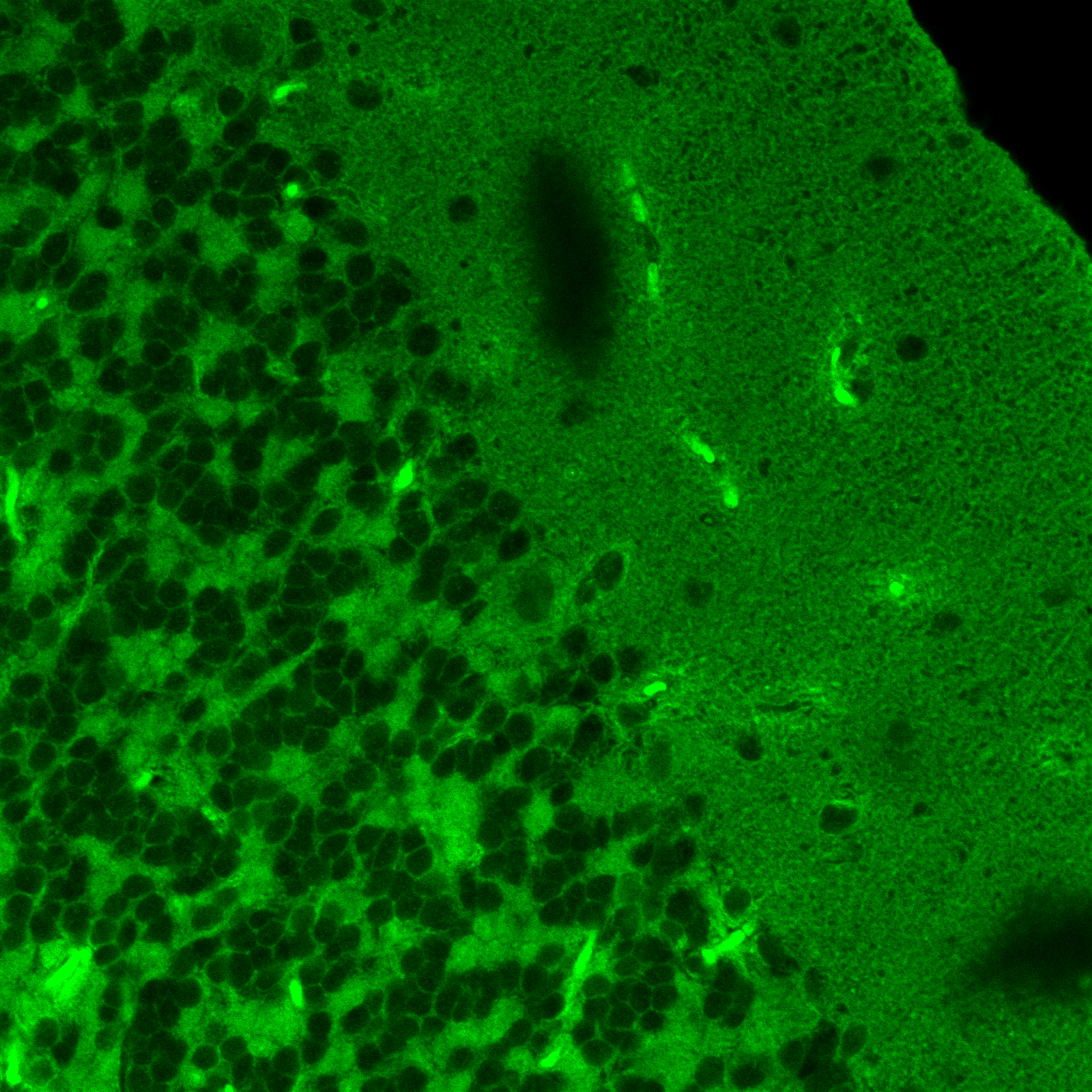

Supplement: Supplementary file 11 — Source data Fig. 10 [file 44318_2024_192_MOESM11_ESM.zip › Figure10/Figure10e/Atxn1-KI, 9weeks_PQBP3+PSME3/PQBP3.tif]

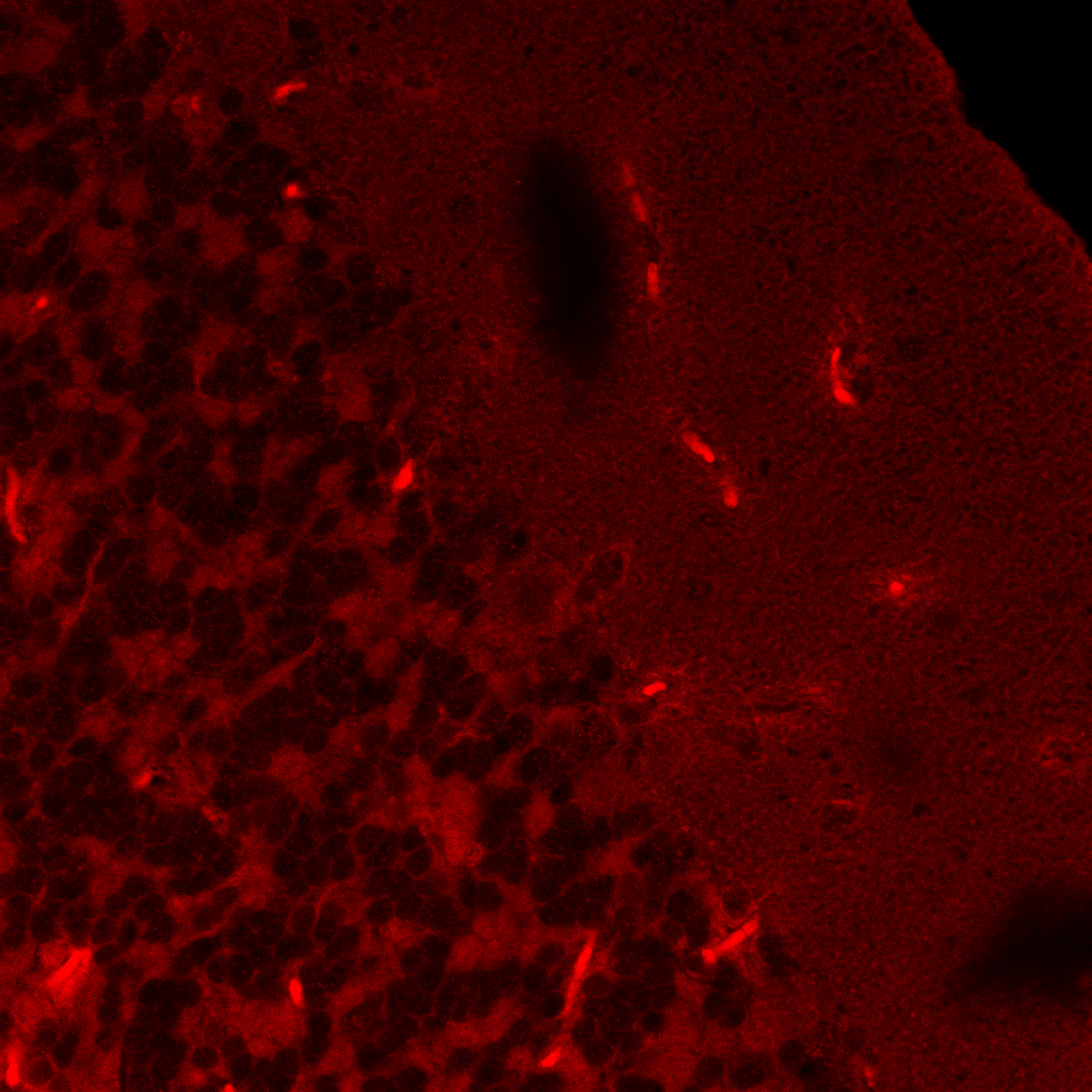

Supplement: Supplementary file 11 — Source data Fig. 10 [file 44318_2024_192_MOESM11_ESM.zip › Figure10/Figure10e/Atxn1-KI, 9weeks_PQBP3+PSME3/PSME3.tif]

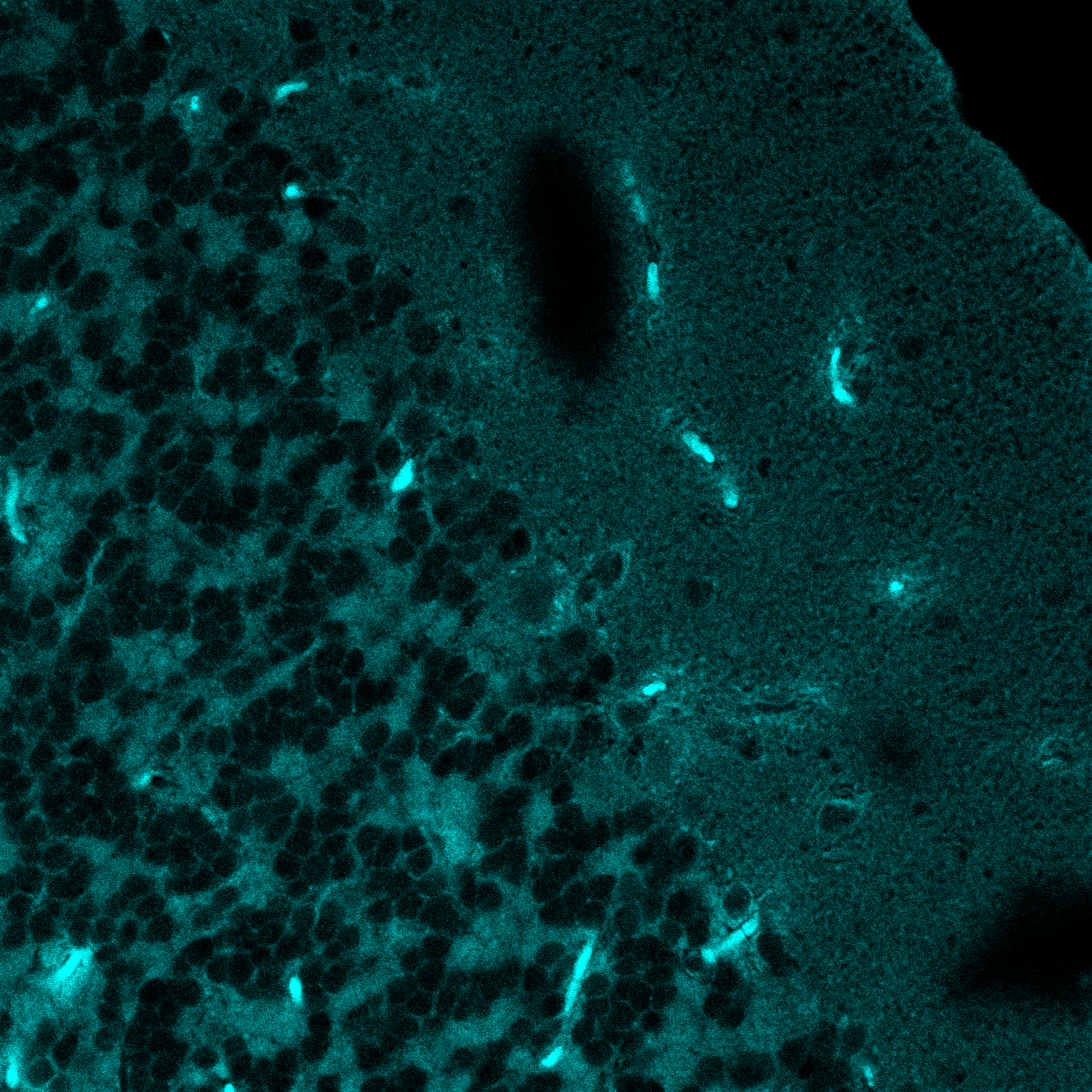

Supplement: Supplementary file 11 — Source data Fig. 10 [file 44318_2024_192_MOESM11_ESM.zip › Figure10/Figure10e/Atxn1-KI, 9weeks_PQBP3+PSME3/ubiquitin.tif]

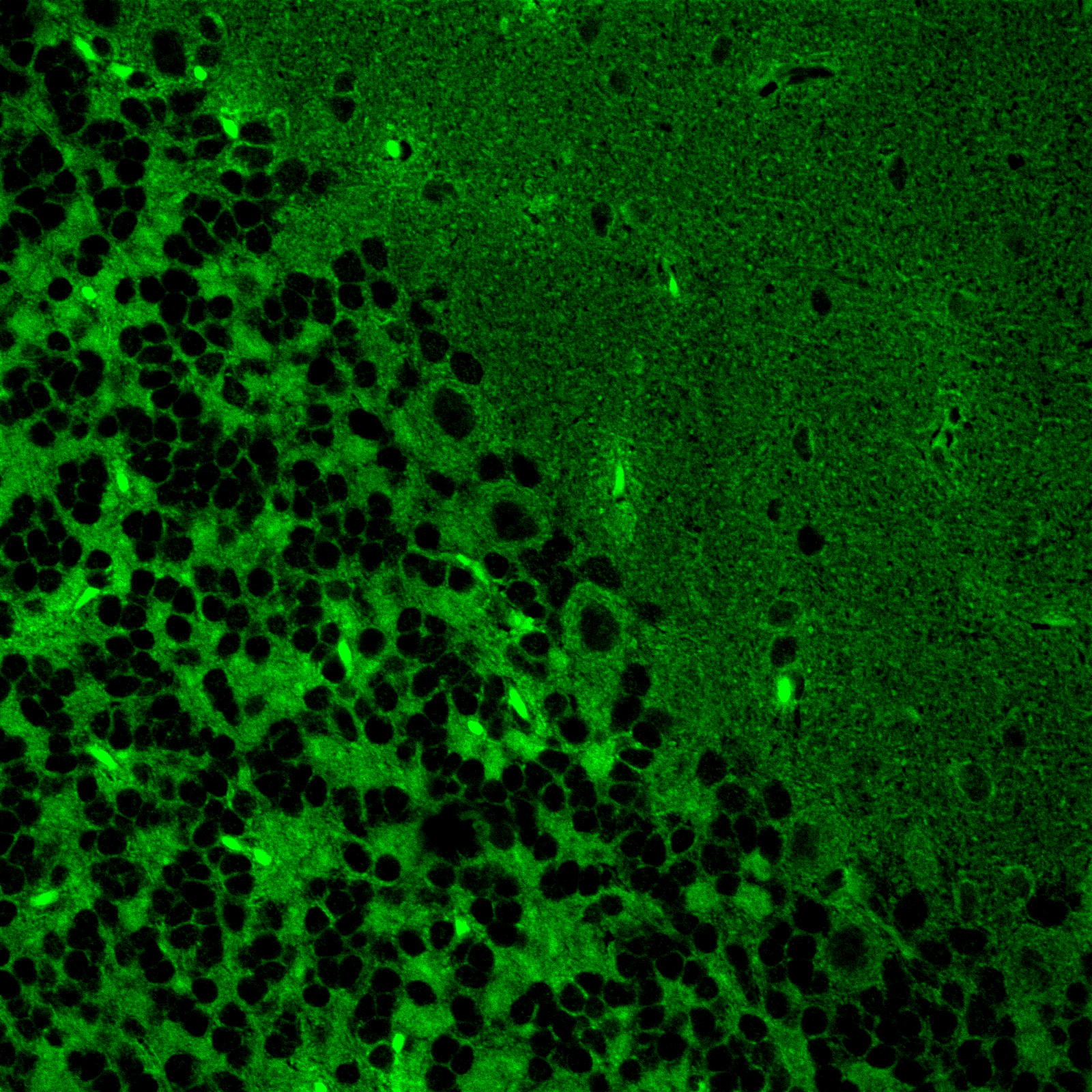

Supplement: Supplementary file 11 — Source data Fig. 10 [file 44318_2024_192_MOESM11_ESM.zip › Figure10/Figure10e/Sibling non-Tg (C57BL6), 9weeks_Atxn1+PSME3/Atxn1.tif]

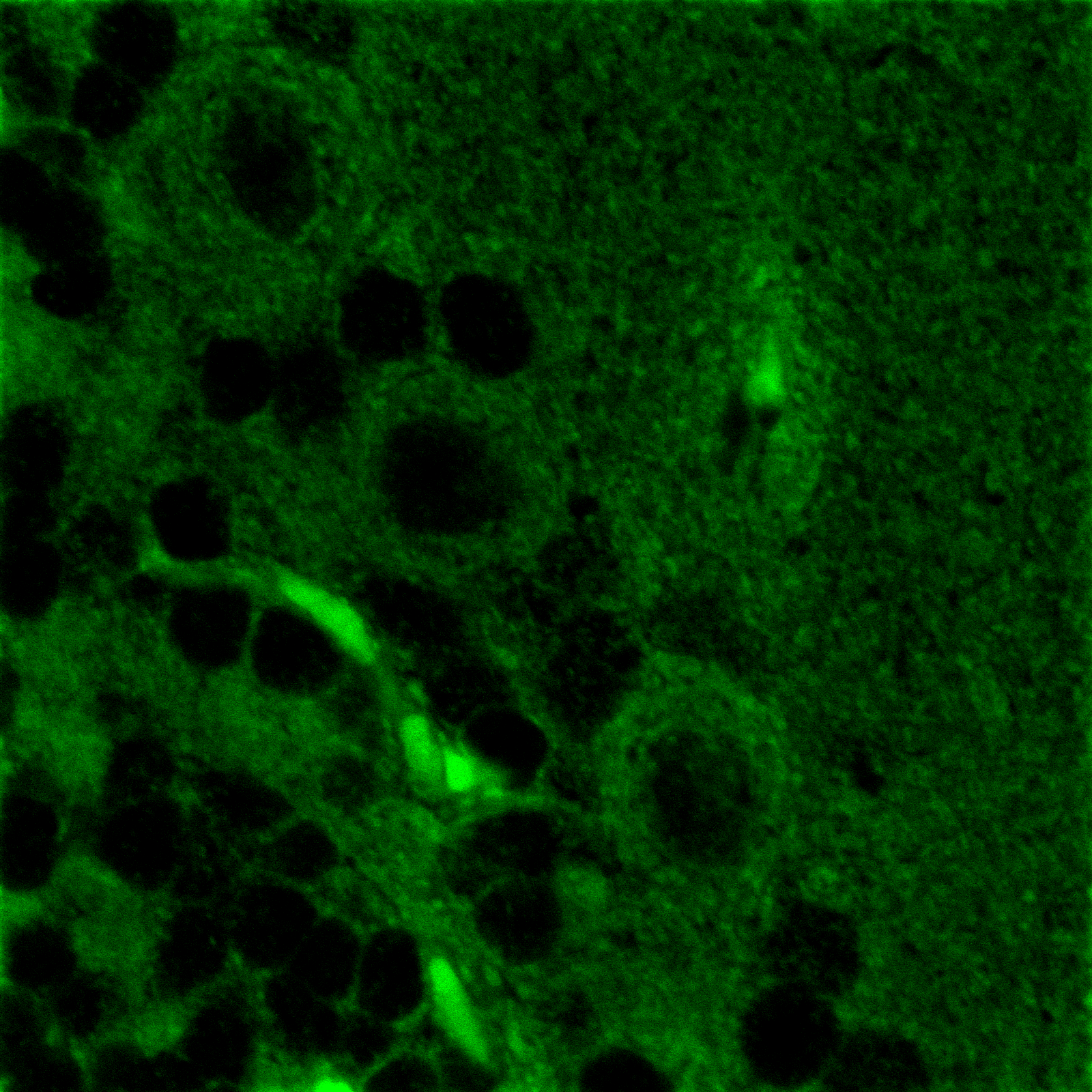

Supplement: Supplementary file 11 — Source data Fig. 10 [file 44318_2024_192_MOESM11_ESM.zip › Figure10/Figure10e/Sibling non-Tg (C57BL6), 9weeks_Atxn1+PSME3/enlarge_Atxn1.tif]

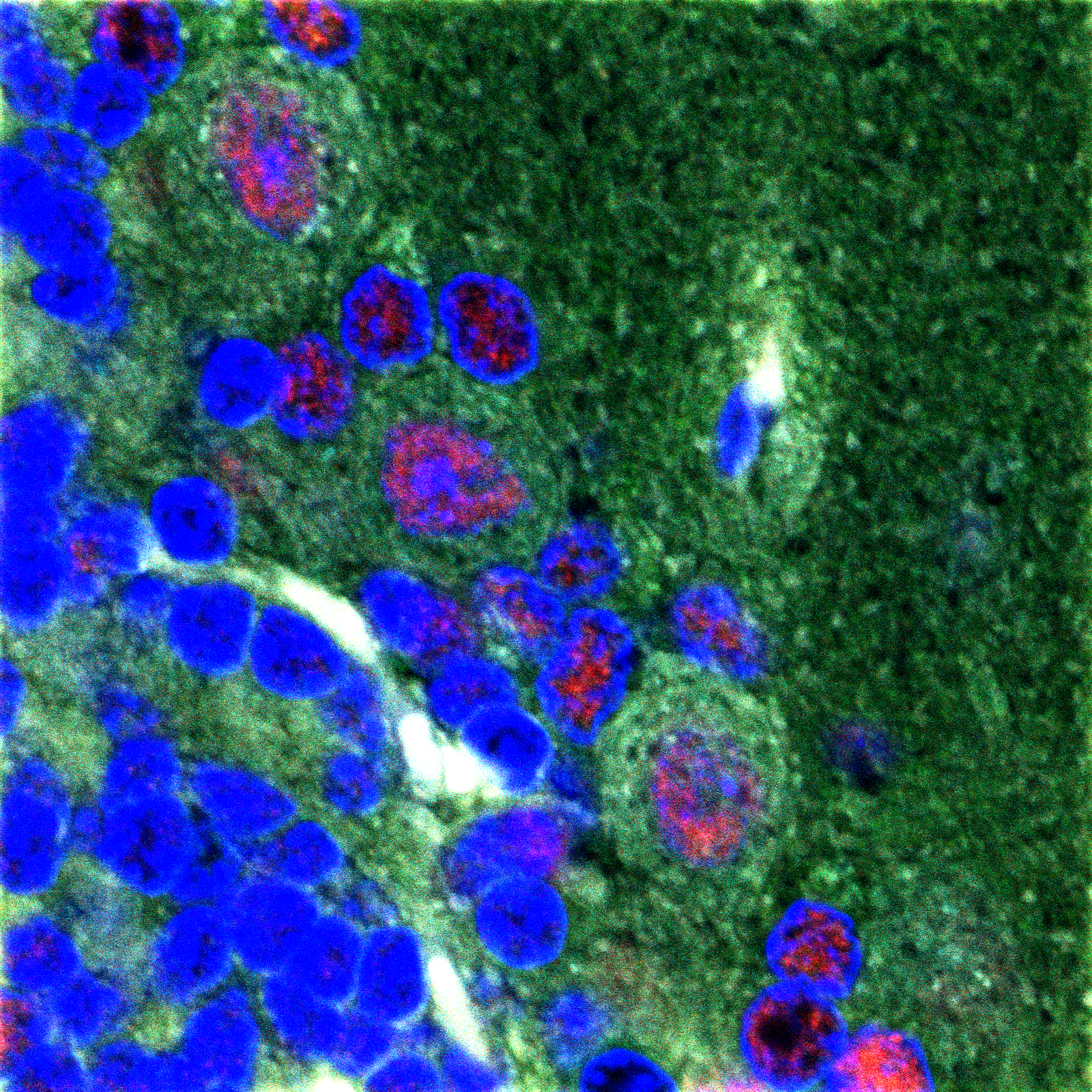

Supplement: Supplementary file 11 — Source data Fig. 10 [file 44318_2024_192_MOESM11_ESM.zip › Figure10/Figure10e/Sibling non-Tg (C57BL6), 9weeks_Atxn1+PSME3/enlarge_Merge.tif]

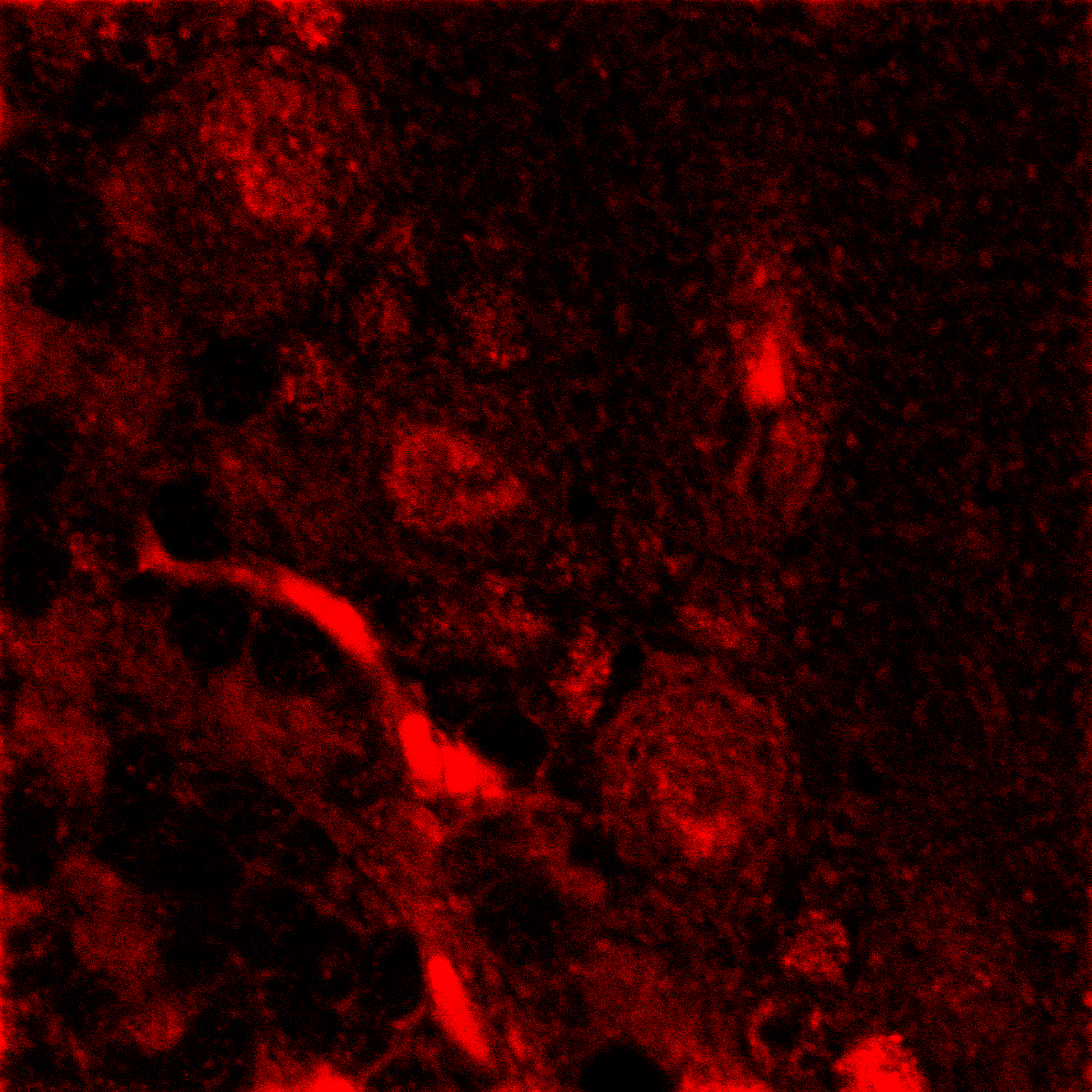

Supplement: Supplementary file 11 — Source data Fig. 10 [file 44318_2024_192_MOESM11_ESM.zip › Figure10/Figure10e/Sibling non-Tg (C57BL6), 9weeks_Atxn1+PSME3/enlarge_PSME3.tif]

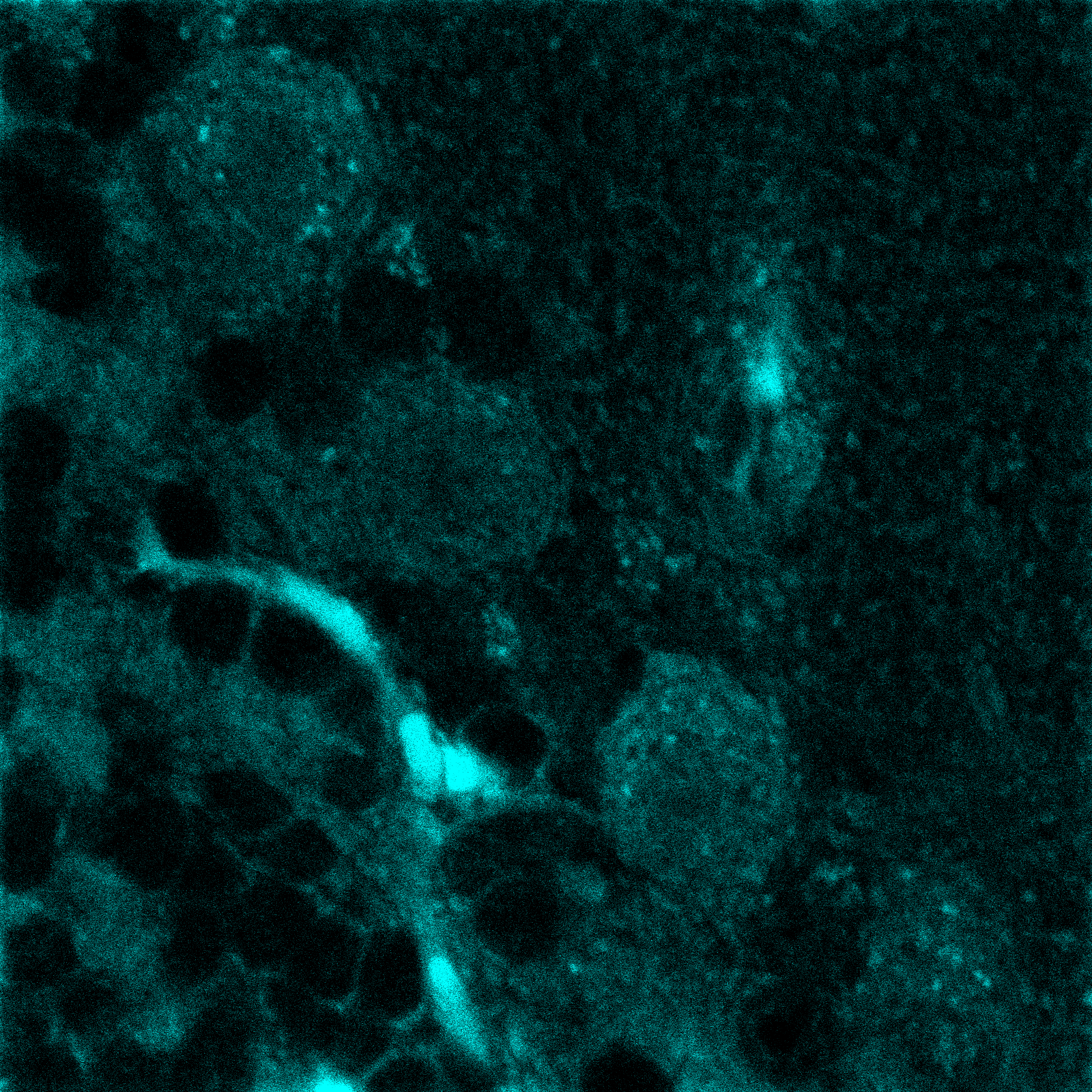

Supplement: Supplementary file 11 — Source data Fig. 10 [file 44318_2024_192_MOESM11_ESM.zip › Figure10/Figure10e/Sibling non-Tg (C57BL6), 9weeks_Atxn1+PSME3/enlarge_ubiquitin.tif]

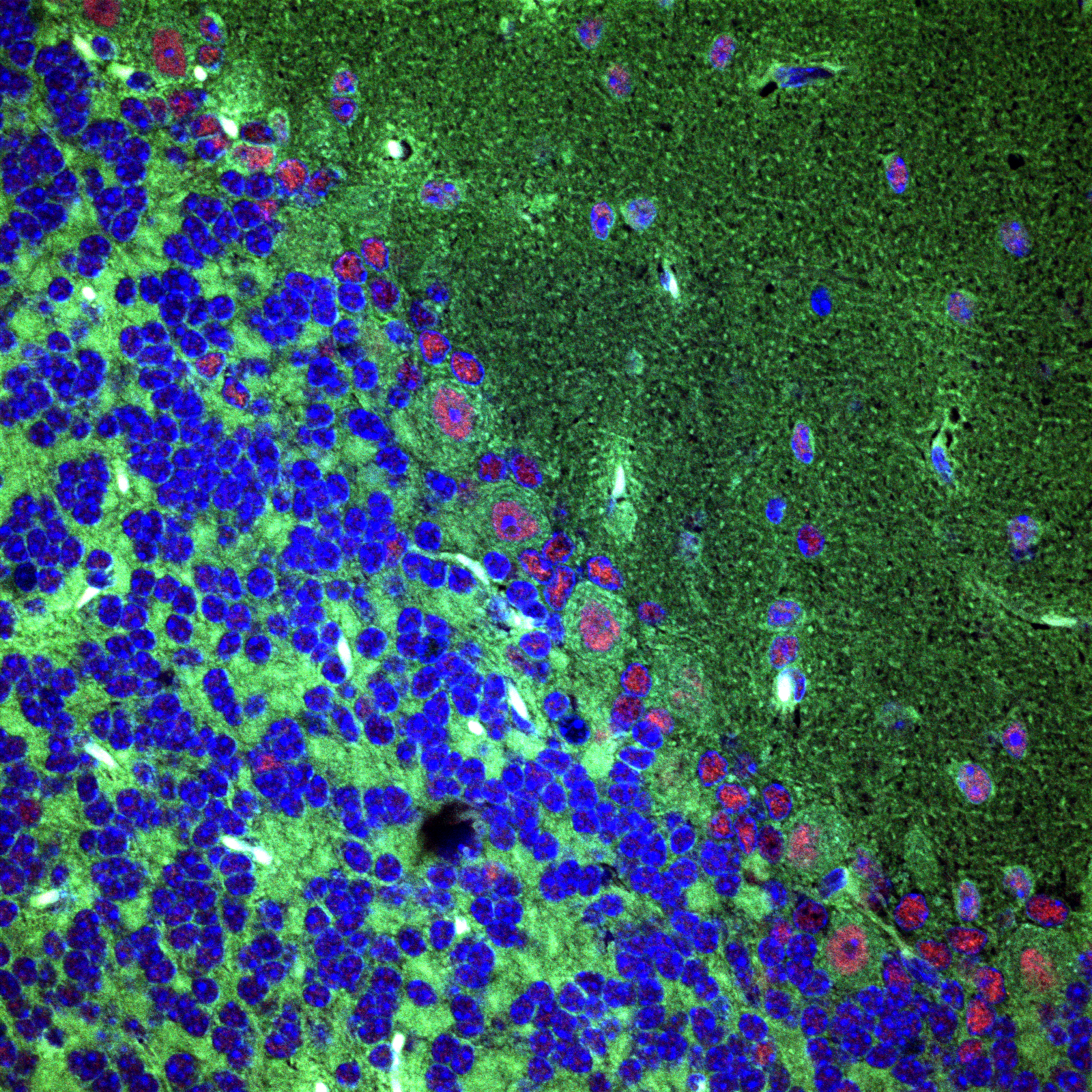

Supplement: Supplementary file 11 — Source data Fig. 10 [file 44318_2024_192_MOESM11_ESM.zip › Figure10/Figure10e/Sibling non-Tg (C57BL6), 9weeks_Atxn1+PSME3/Merge.tif]

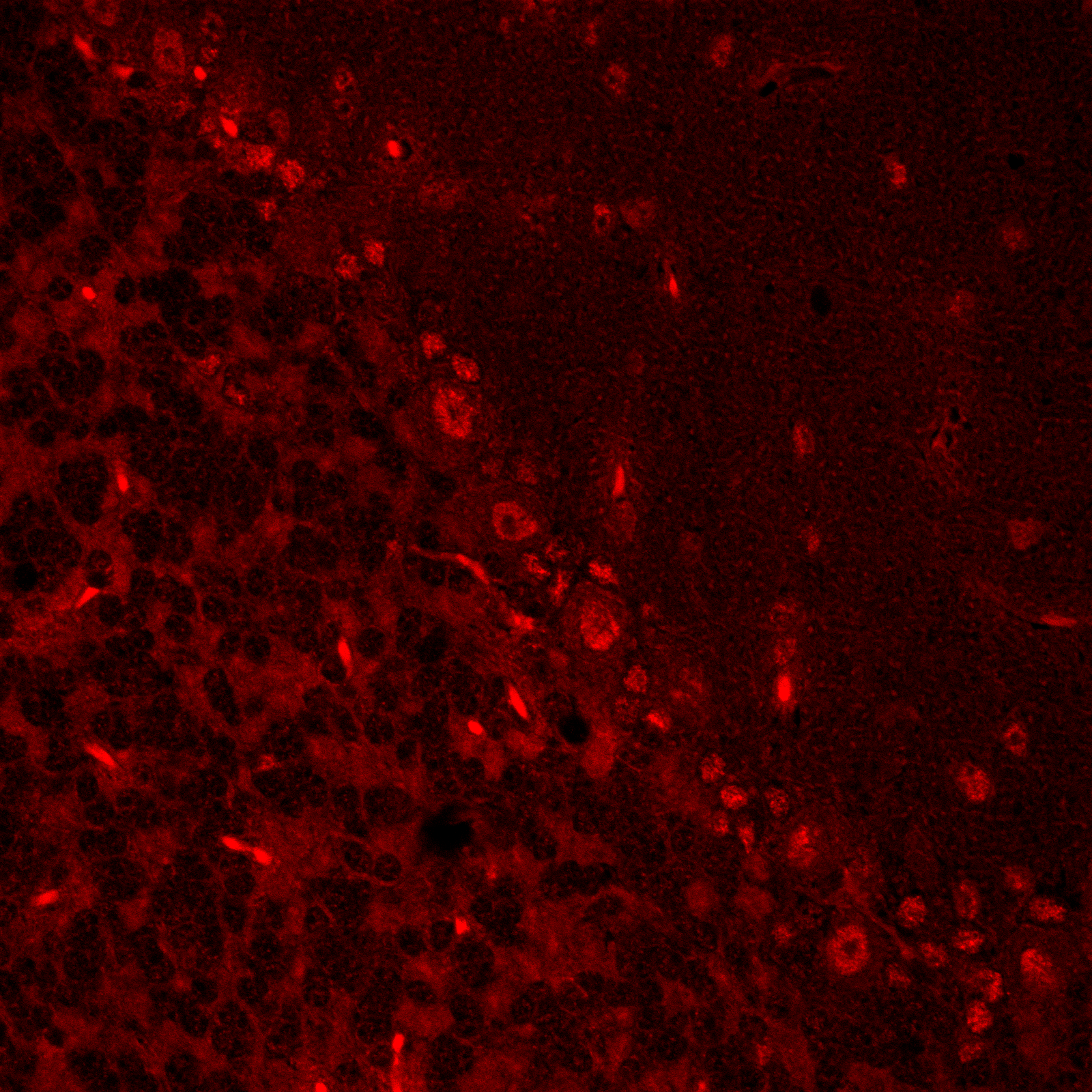

Supplement: Supplementary file 11 — Source data Fig. 10 [file 44318_2024_192_MOESM11_ESM.zip › Figure10/Figure10e/Sibling non-Tg (C57BL6), 9weeks_Atxn1+PSME3/PSME3.tif]

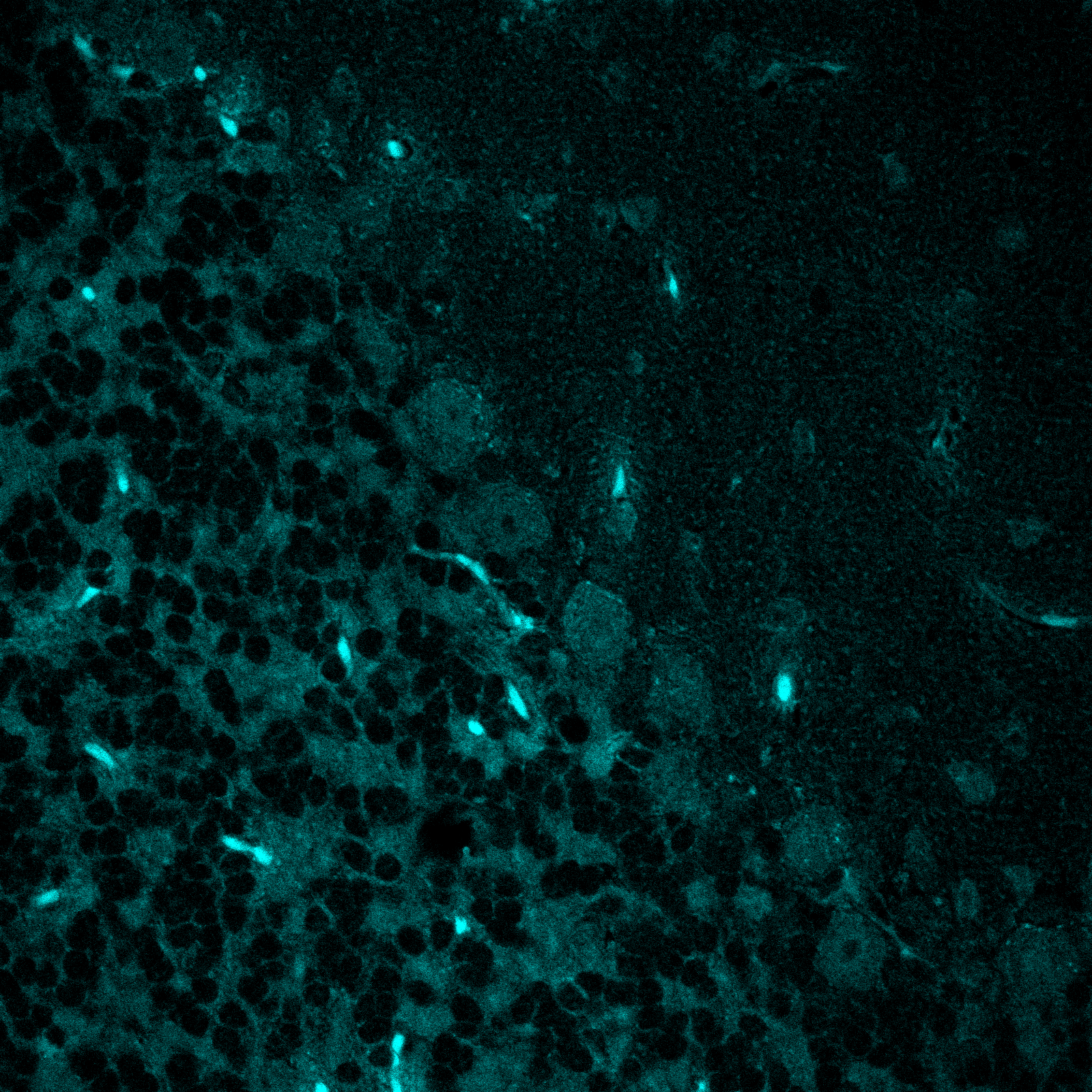

Supplement: Supplementary file 11 — Source data Fig. 10 [file 44318_2024_192_MOESM11_ESM.zip › Figure10/Figure10e/Sibling non-Tg (C57BL6), 9weeks_Atxn1+PSME3/ubiquitin.tif]

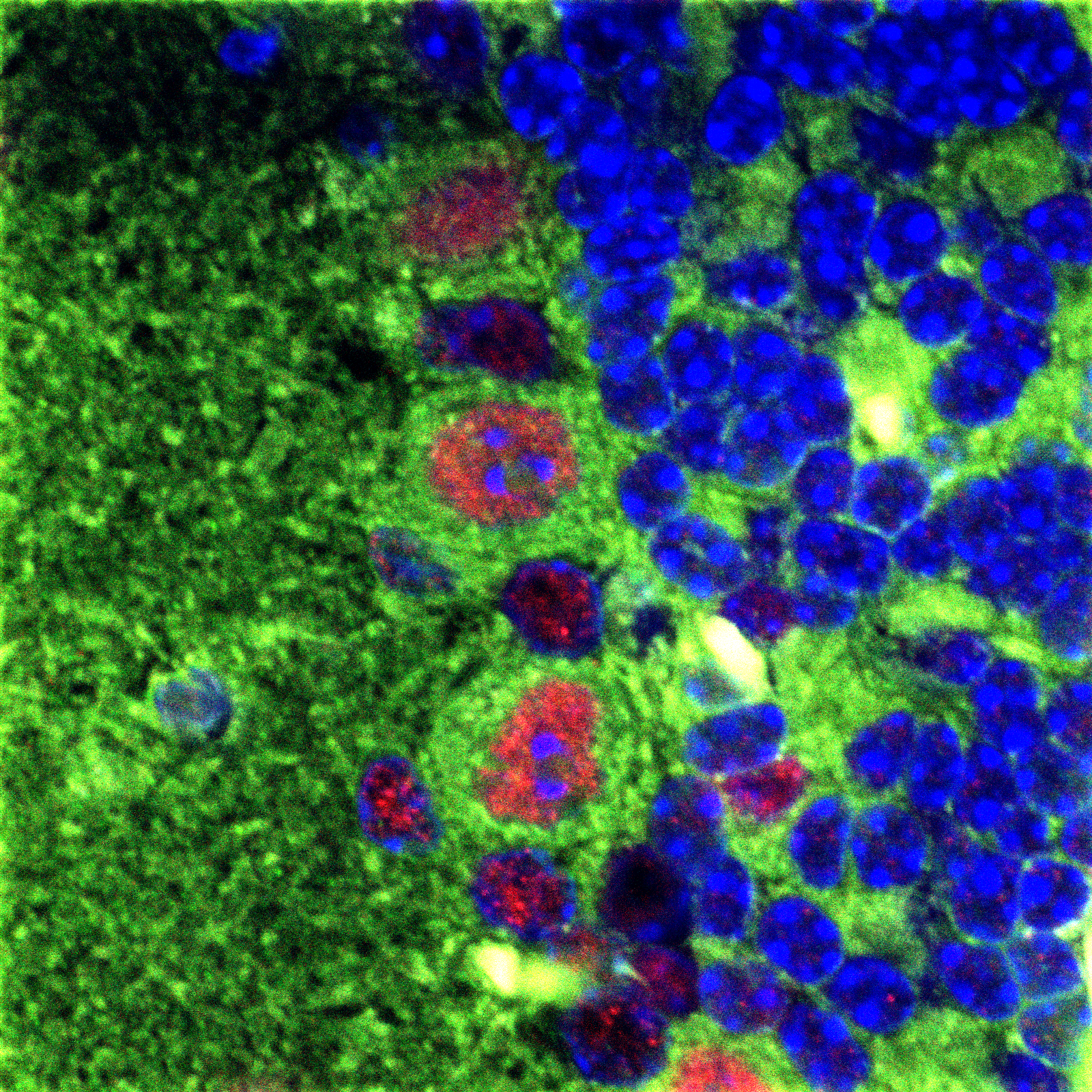

Supplement: Supplementary file 11 — Source data Fig. 10 [file 44318_2024_192_MOESM11_ESM.zip › Figure10/Figure10e/Sibling non-Tg (C57BL6), 9weeks_PQBP3+PSME3/enlarge_Merge.tif]

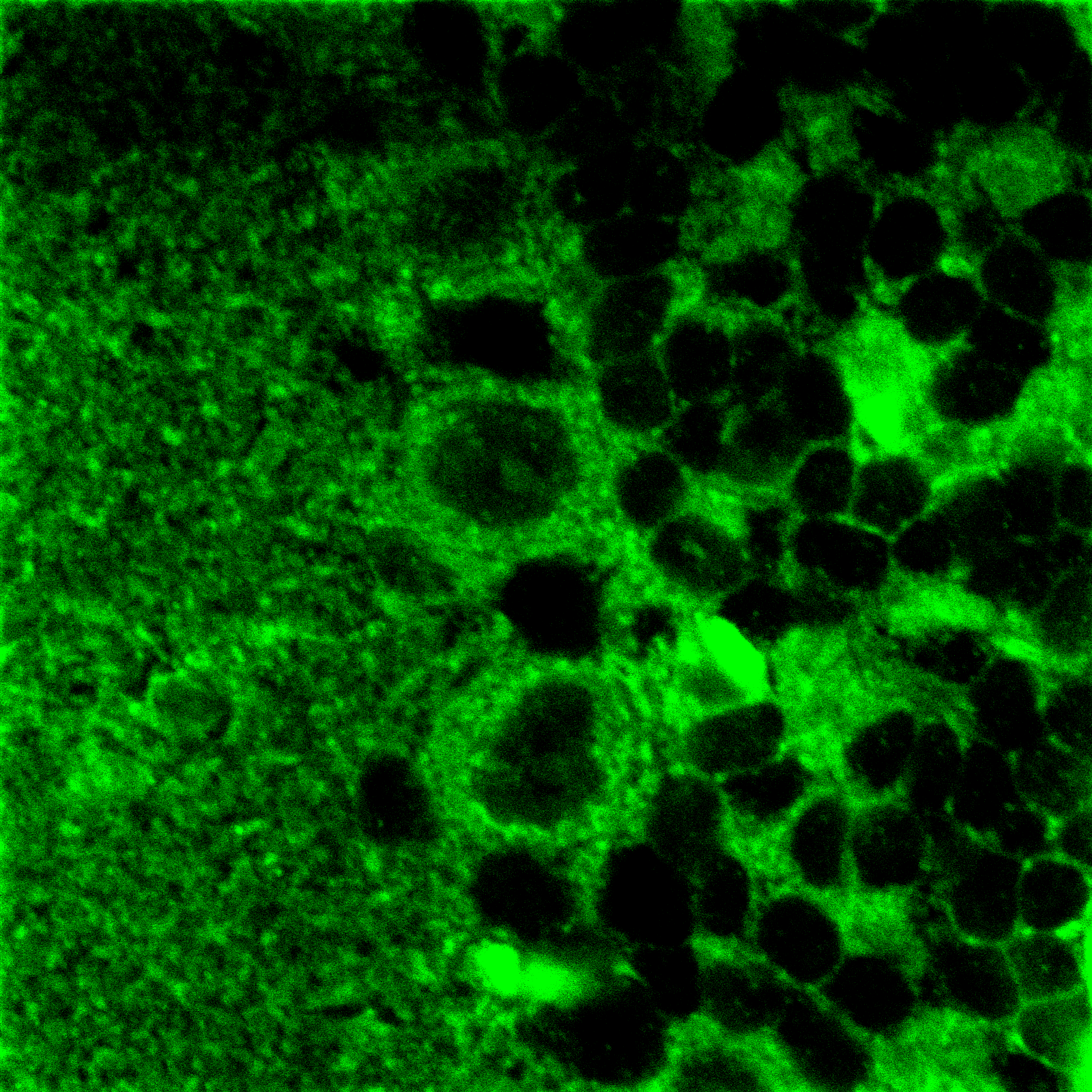

Supplement: Supplementary file 11 — Source data Fig. 10 [file 44318_2024_192_MOESM11_ESM.zip › Figure10/Figure10e/Sibling non-Tg (C57BL6), 9weeks_PQBP3+PSME3/enlarge_PQBP3.tif]

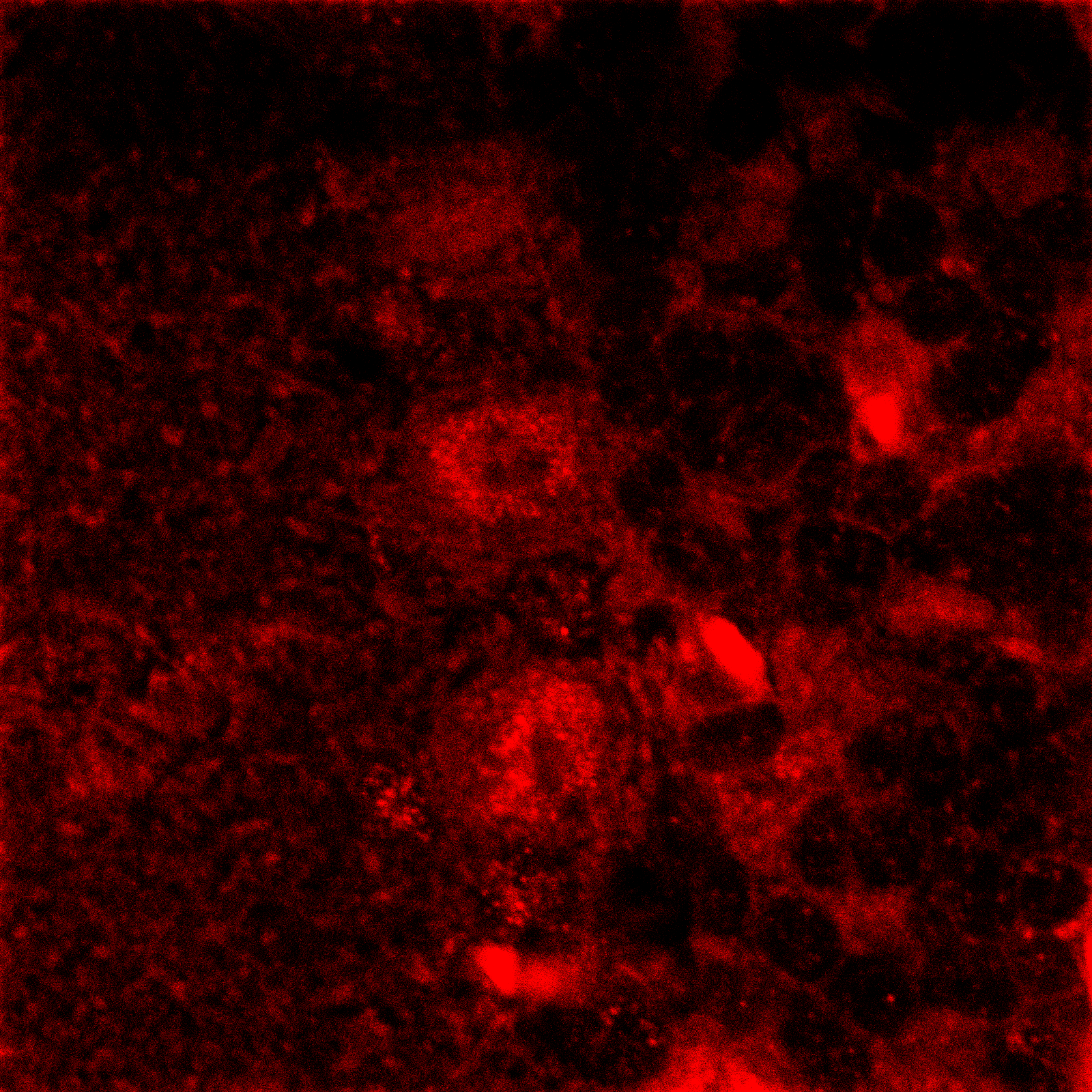

Supplement: Supplementary file 11 — Source data Fig. 10 [file 44318_2024_192_MOESM11_ESM.zip › Figure10/Figure10e/Sibling non-Tg (C57BL6), 9weeks_PQBP3+PSME3/enlarge_PSME3.tif]

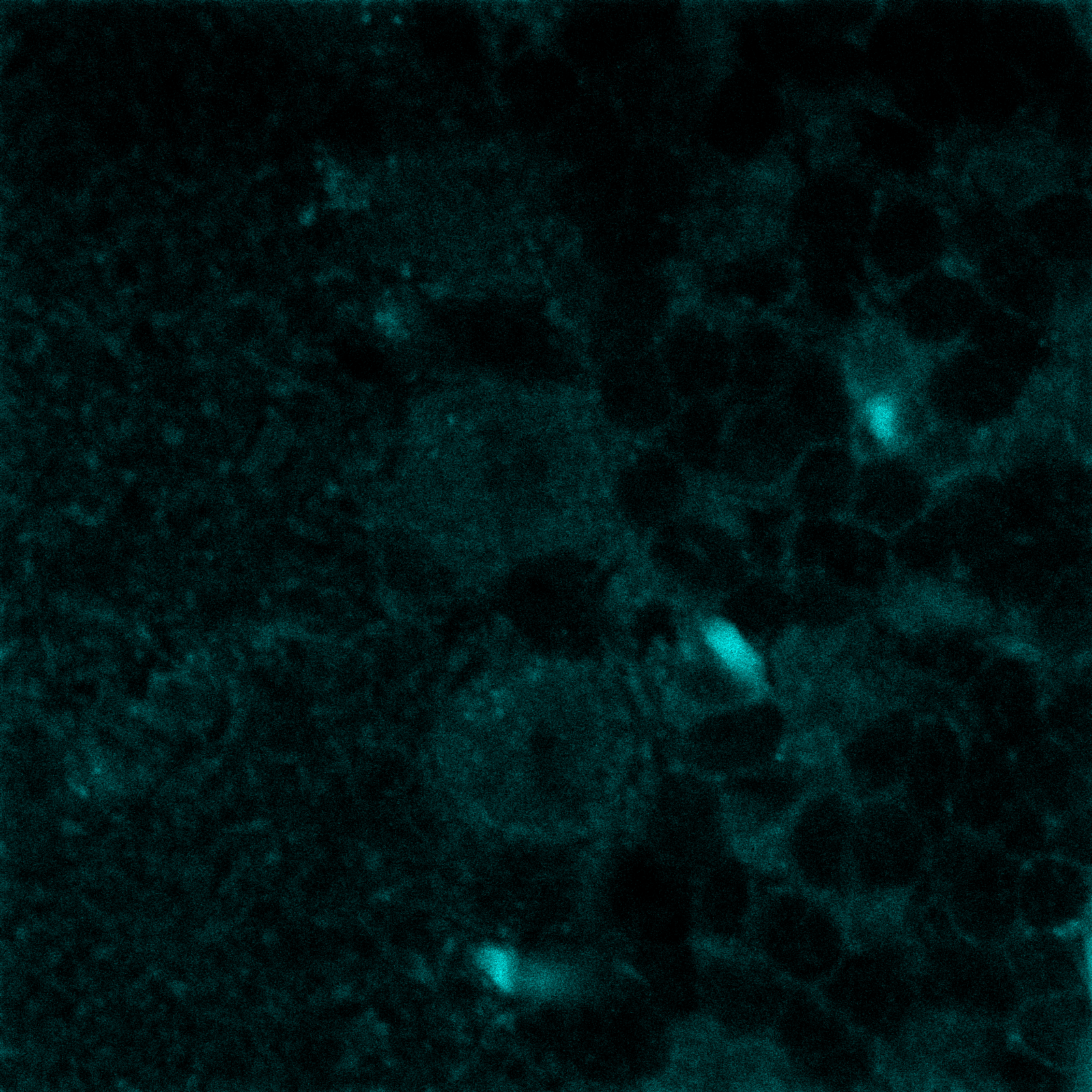

Supplement: Supplementary file 11 — Source data Fig. 10 [file 44318_2024_192_MOESM11_ESM.zip › Figure10/Figure10e/Sibling non-Tg (C57BL6), 9weeks_PQBP3+PSME3/enlarge_ubiquitin.tif]

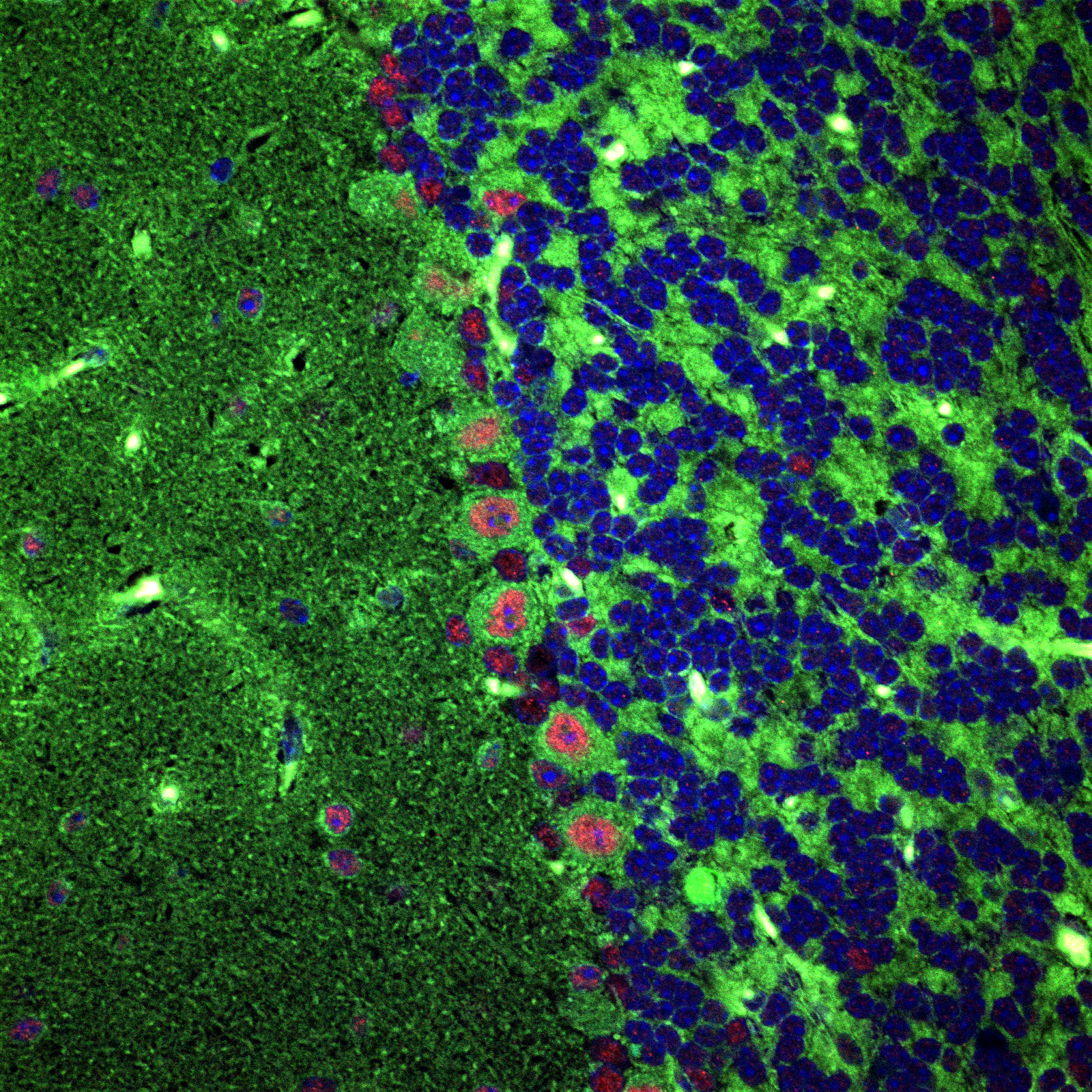

Supplement: Supplementary file 11 — Source data Fig. 10 [file 44318_2024_192_MOESM11_ESM.zip › Figure10/Figure10e/Sibling non-Tg (C57BL6), 9weeks_PQBP3+PSME3/Merge.tif]

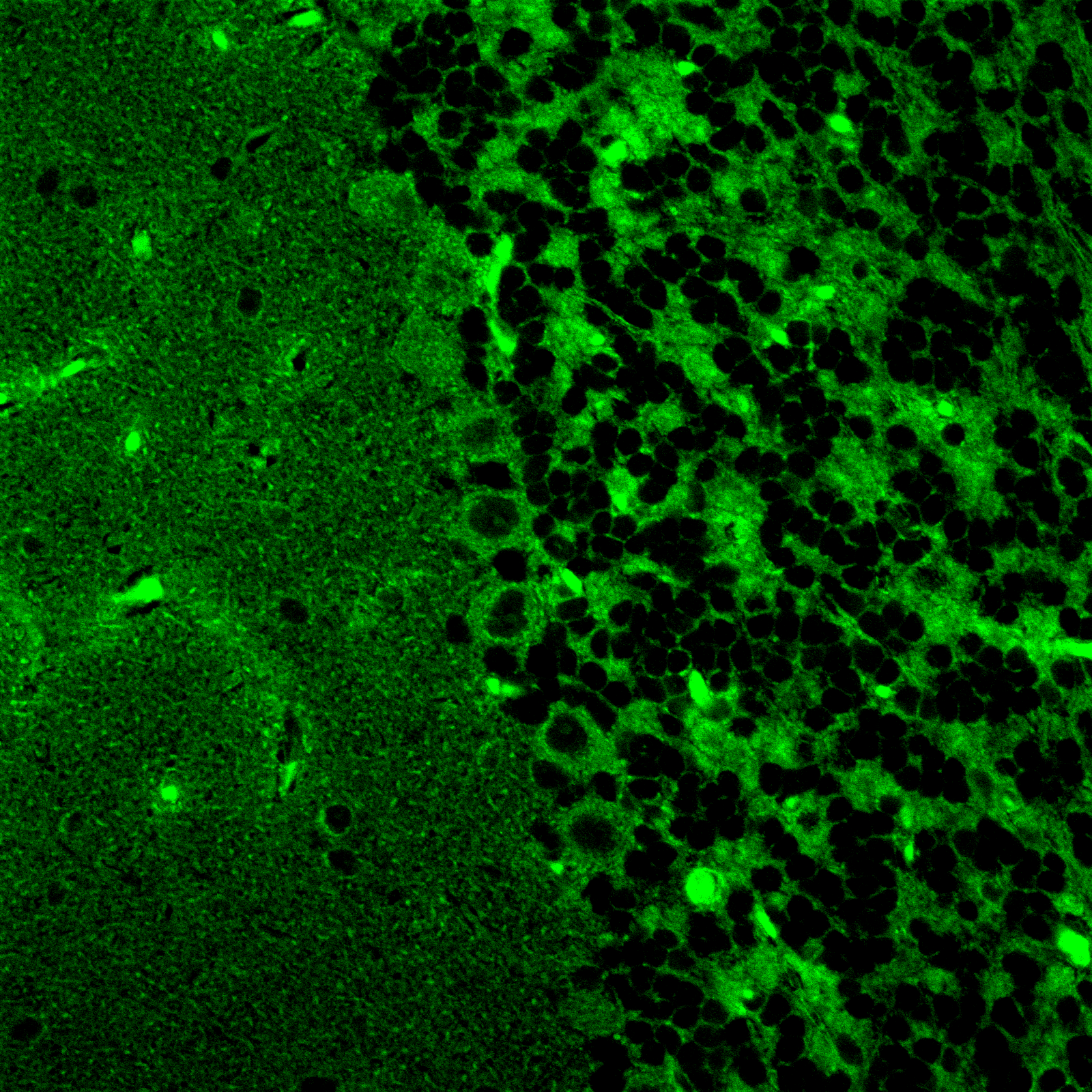

Supplement: Supplementary file 11 — Source data Fig. 10 [file 44318_2024_192_MOESM11_ESM.zip › Figure10/Figure10e/Sibling non-Tg (C57BL6), 9weeks_PQBP3+PSME3/PQBP3.tif]

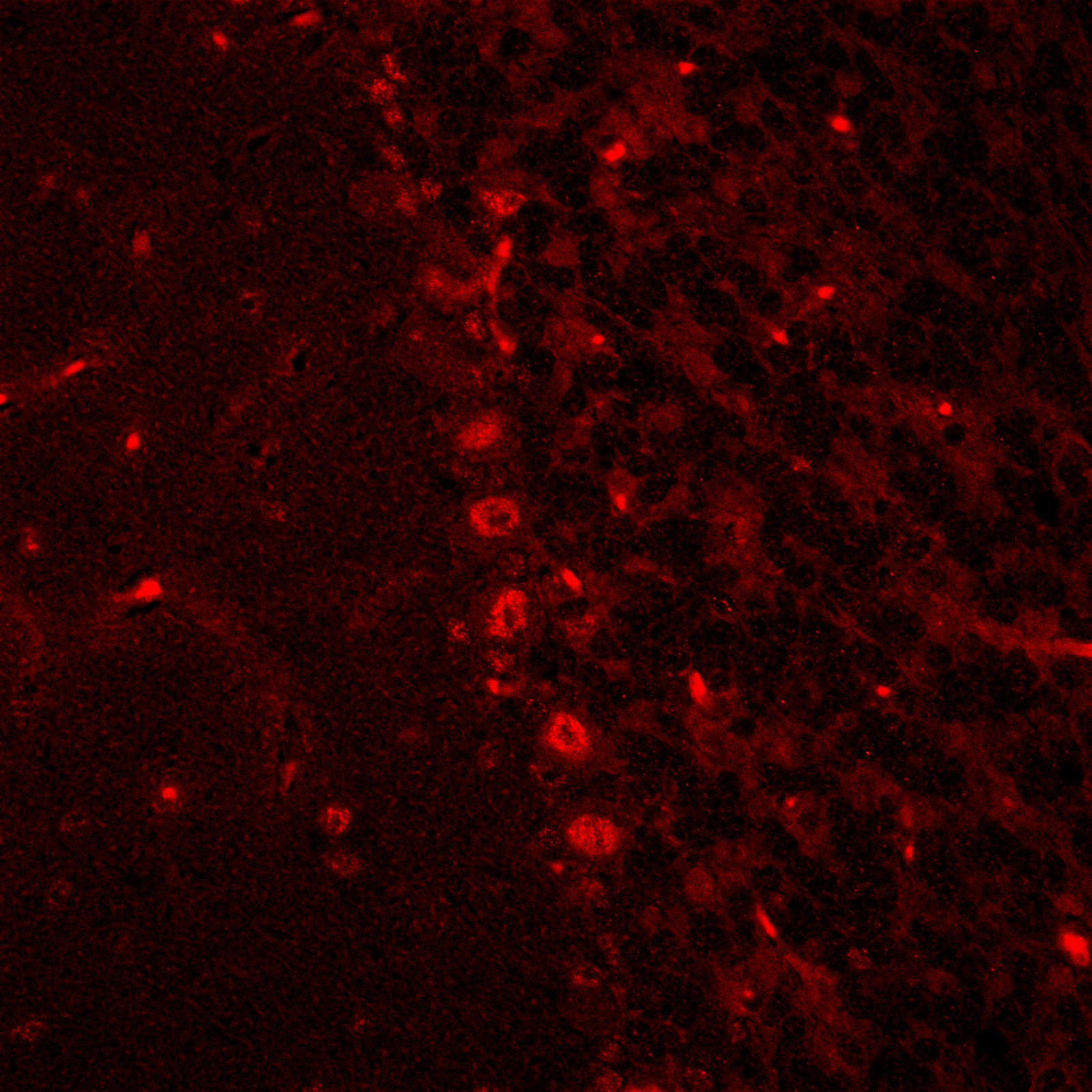

Supplement: Supplementary file 11 — Source data Fig. 10 [file 44318_2024_192_MOESM11_ESM.zip › Figure10/Figure10e/Sibling non-Tg (C57BL6), 9weeks_PQBP3+PSME3/PSME3.tif]

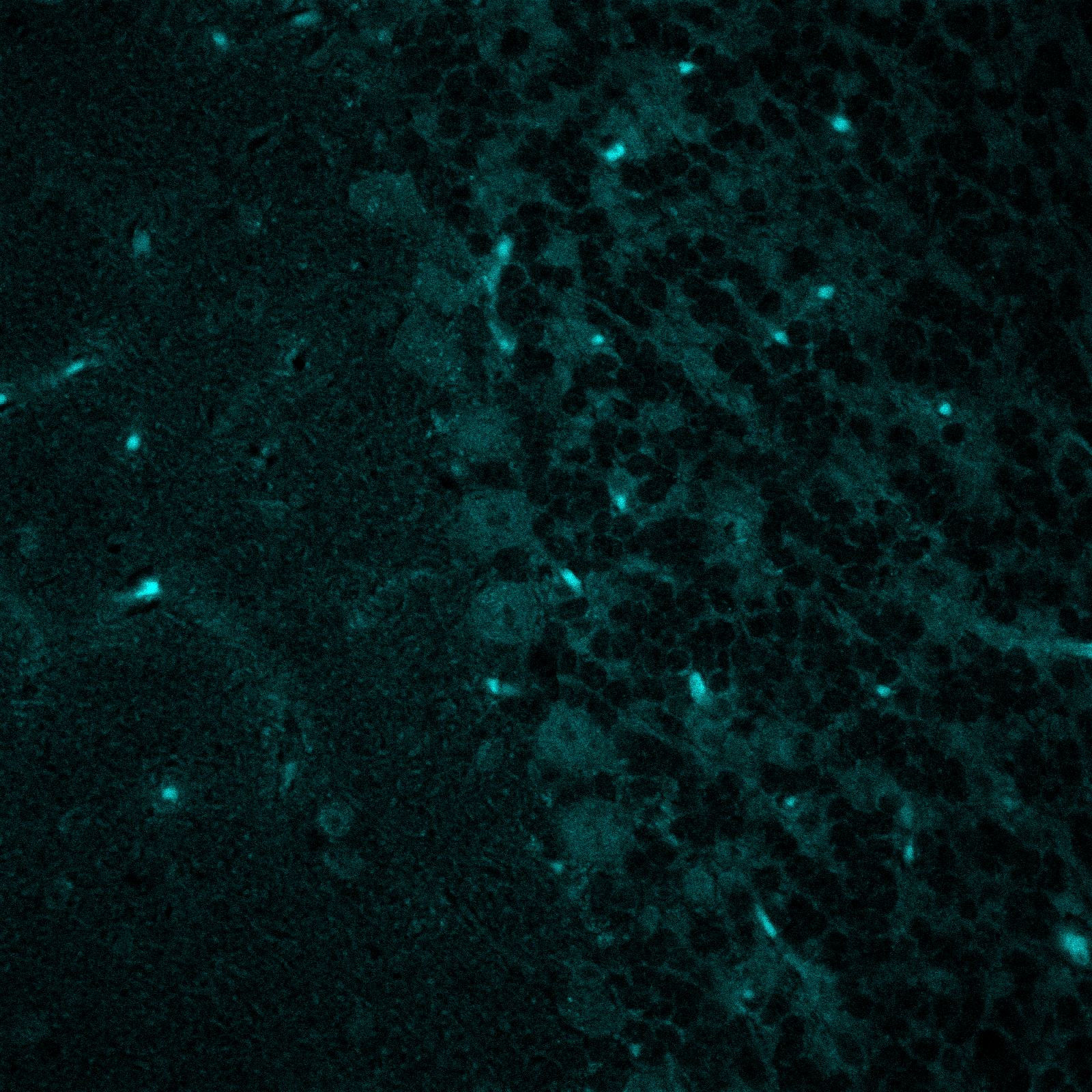

Supplement: Supplementary file 11 — Source data Fig. 10 [file 44318_2024_192_MOESM11_ESM.zip › Figure10/Figure10e/Sibling non-Tg (C57BL6), 9weeks_PQBP3+PSME3/ubiquitin.tif]

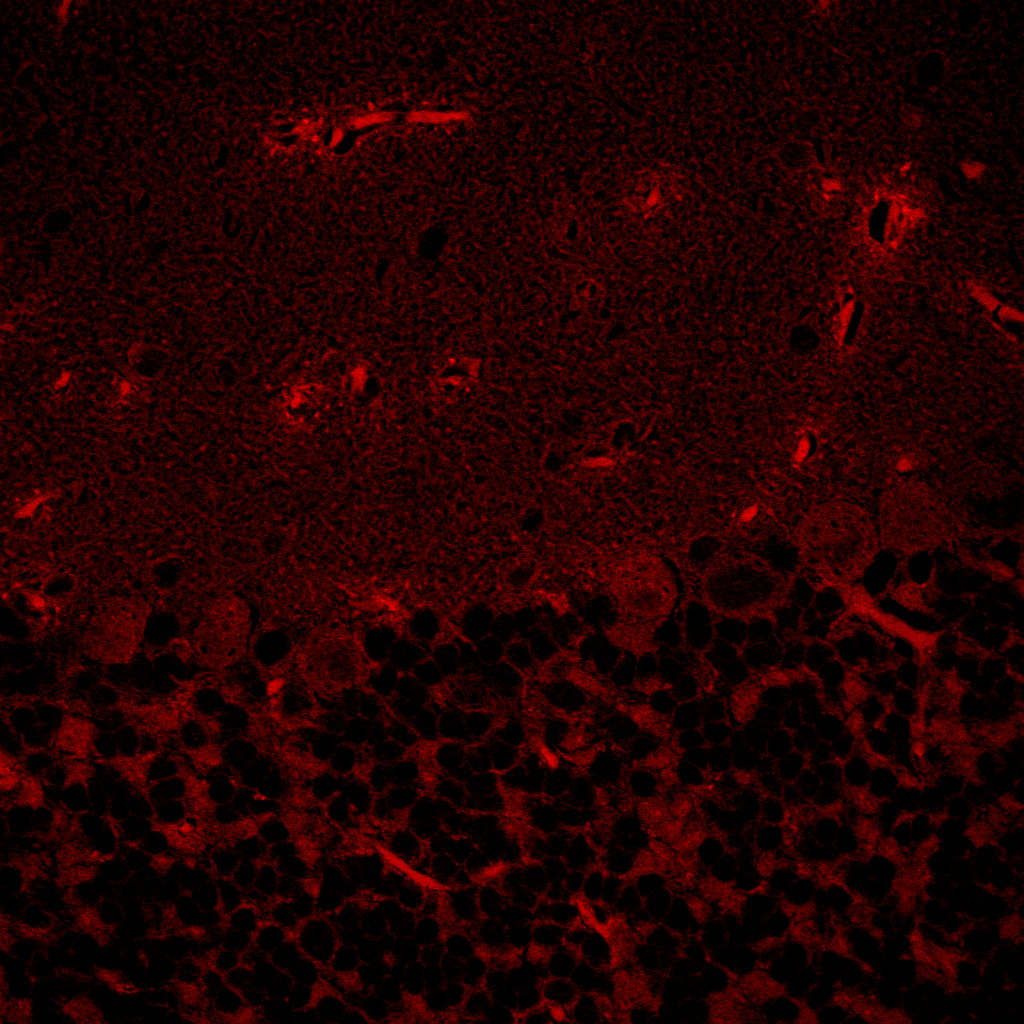

Supplement: Supplementary file 11 — Source data Fig. 10 [file 44318_2024_192_MOESM11_ESM.zip › Figure10/Figure10f/Atxn1-KI, 9weeks/Atxn1.tif]

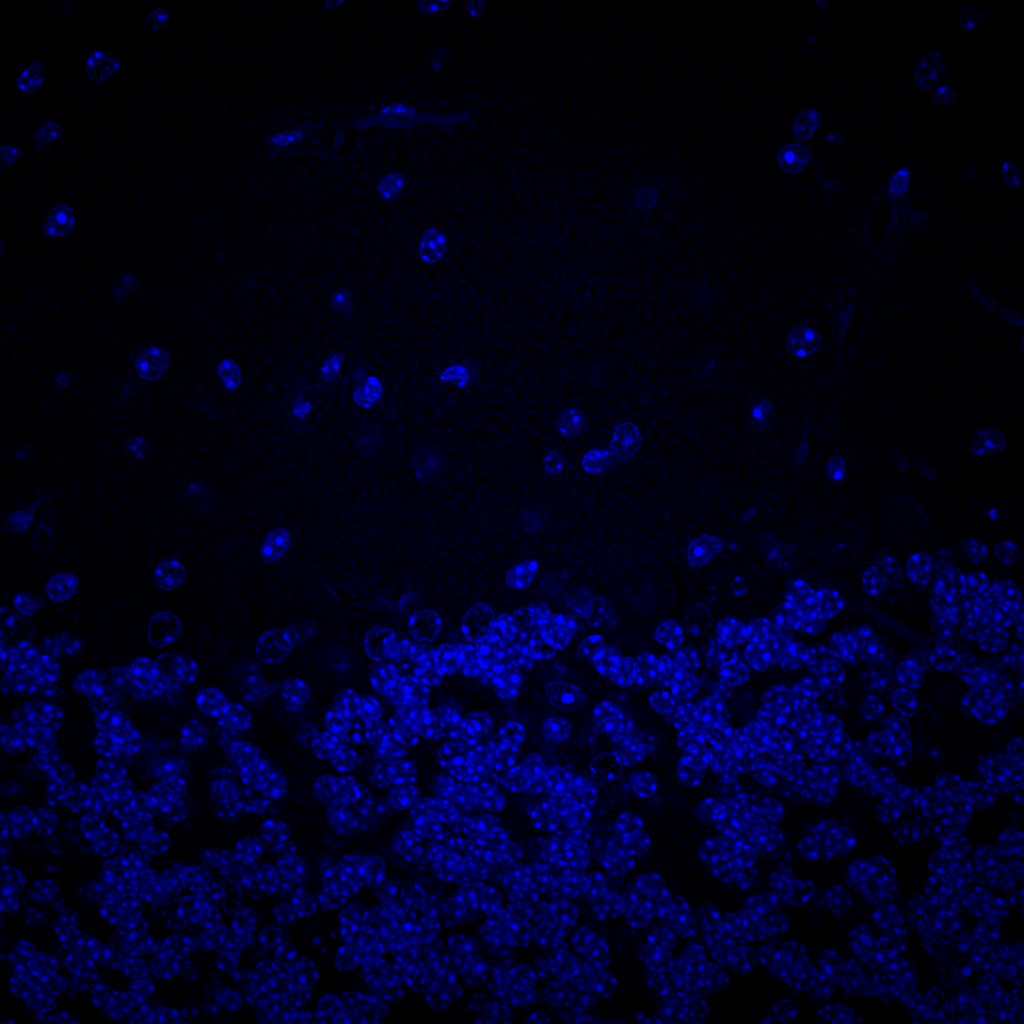

Supplement: Supplementary file 11 — Source data Fig. 10 [file 44318_2024_192_MOESM11_ESM.zip › Figure10/Figure10f/Atxn1-KI, 9weeks/hoechst.tif]

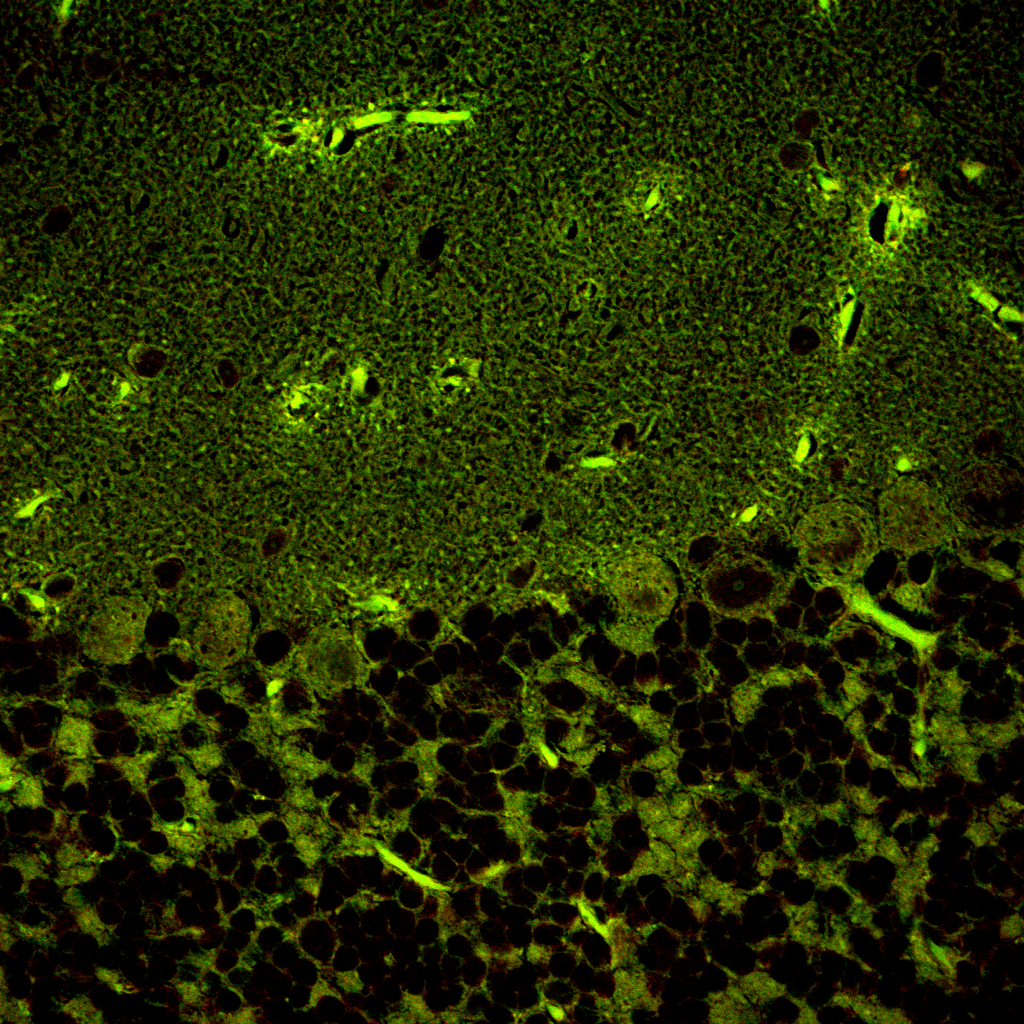

Supplement: Supplementary file 11 — Source data Fig. 10 [file 44318_2024_192_MOESM11_ESM.zip › Figure10/Figure10f/Atxn1-KI, 9weeks/Merge.tif]

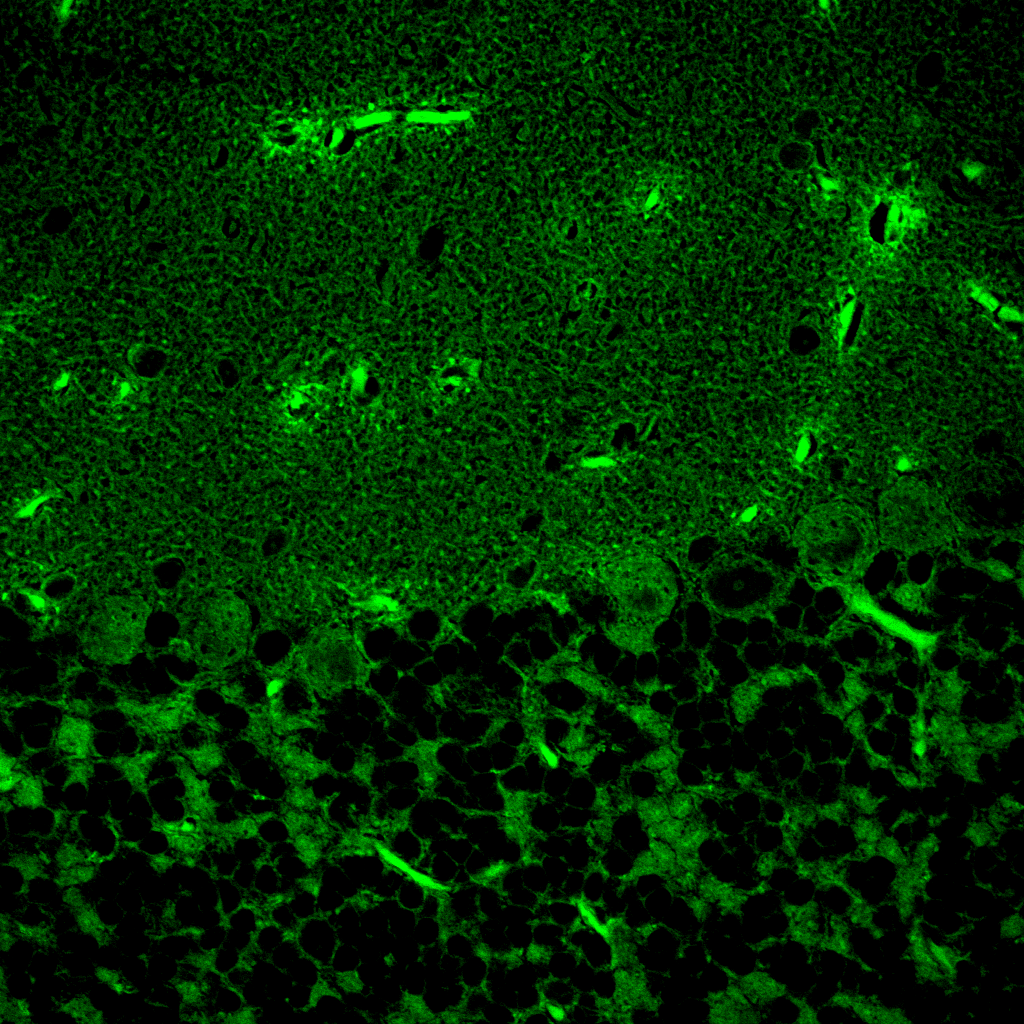

Supplement: Supplementary file 11 — Source data Fig. 10 [file 44318_2024_192_MOESM11_ESM.zip › Figure10/Figure10f/Atxn1-KI, 9weeks/PQBP3.tif]

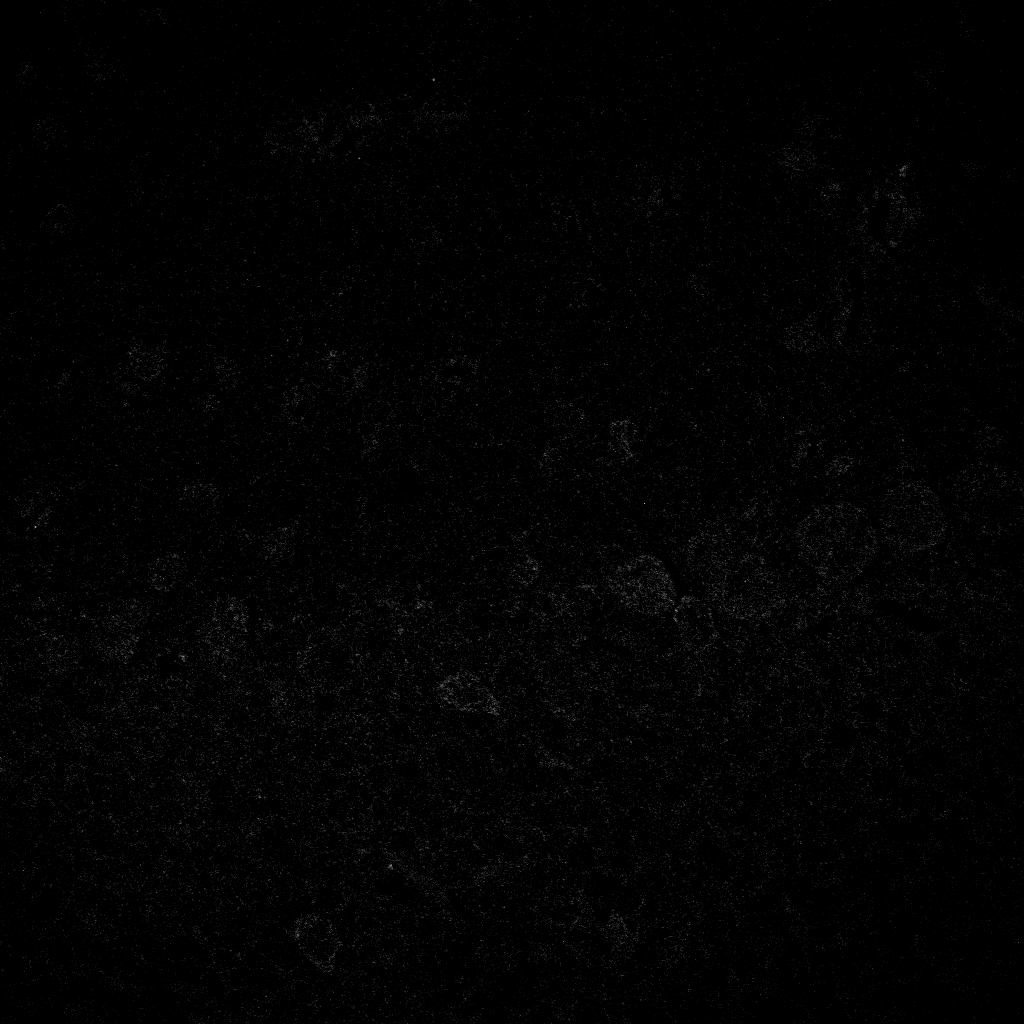

Supplement: Supplementary file 11 — Source data Fig. 10 [file 44318_2024_192_MOESM11_ESM.zip › Figure10/Figure10f/Atxn1-KI, 9weeks/ubiquitin.tif]

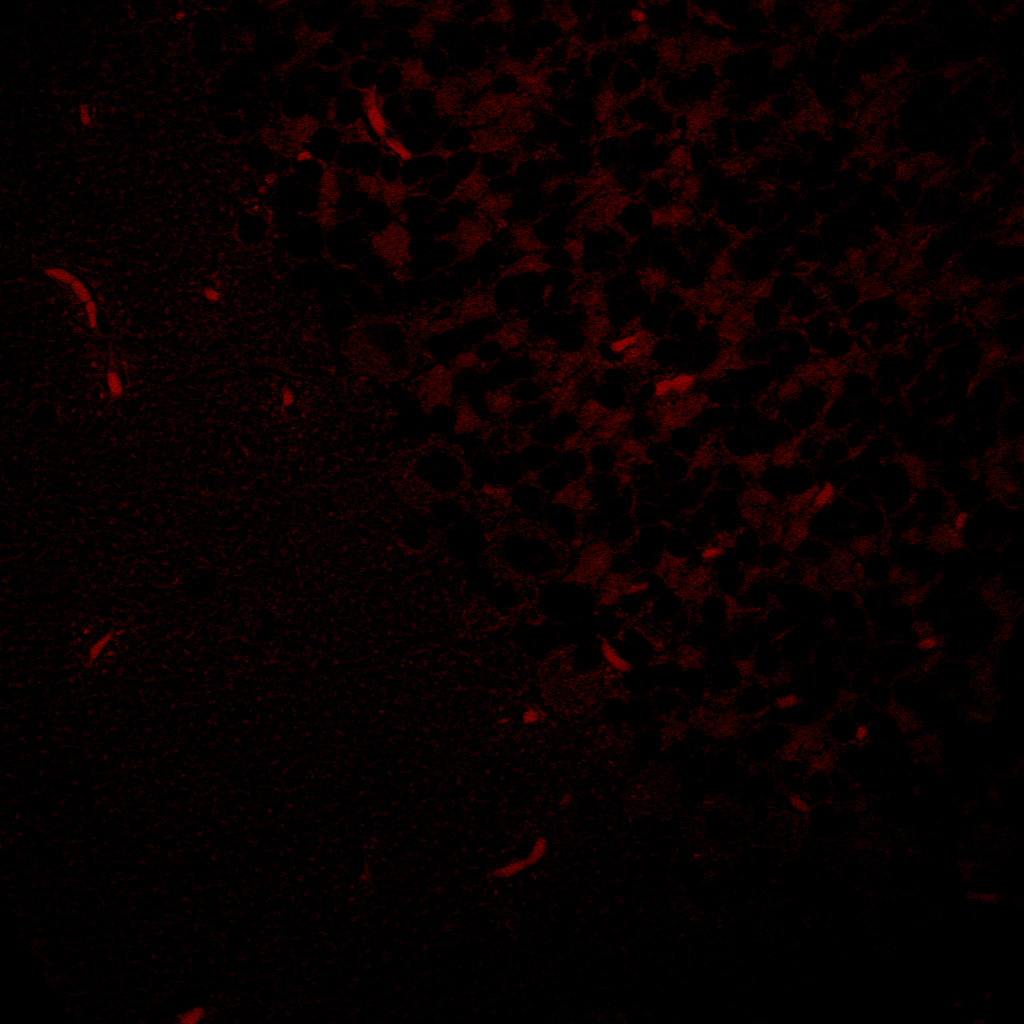

Supplement: Supplementary file 11 — Source data Fig. 10 [file 44318_2024_192_MOESM11_ESM.zip › Figure10/Figure10f/Sibling non-Tg (C57BL6), 9weeks/Atxn1.tif]

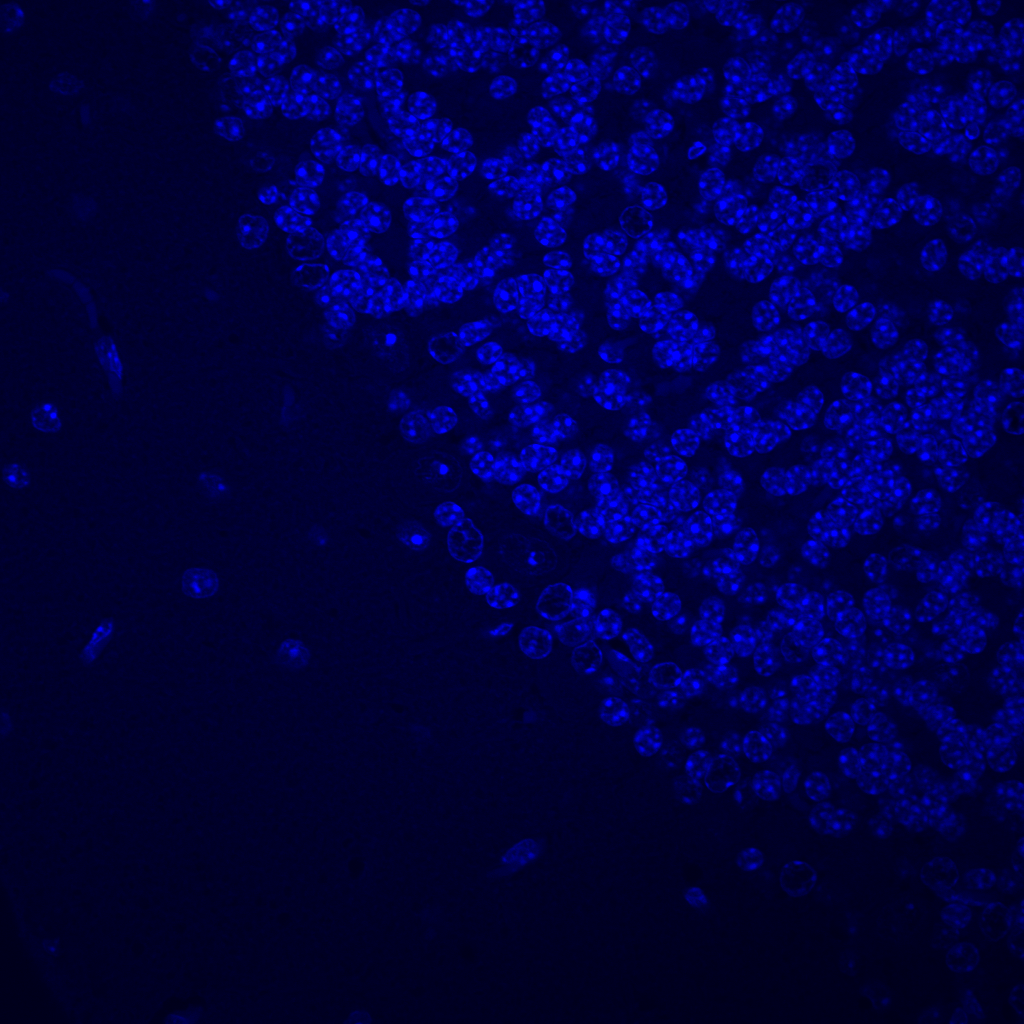

Supplement: Supplementary file 11 — Source data Fig. 10 [file 44318_2024_192_MOESM11_ESM.zip › Figure10/Figure10f/Sibling non-Tg (C57BL6), 9weeks/hoechst.tif]

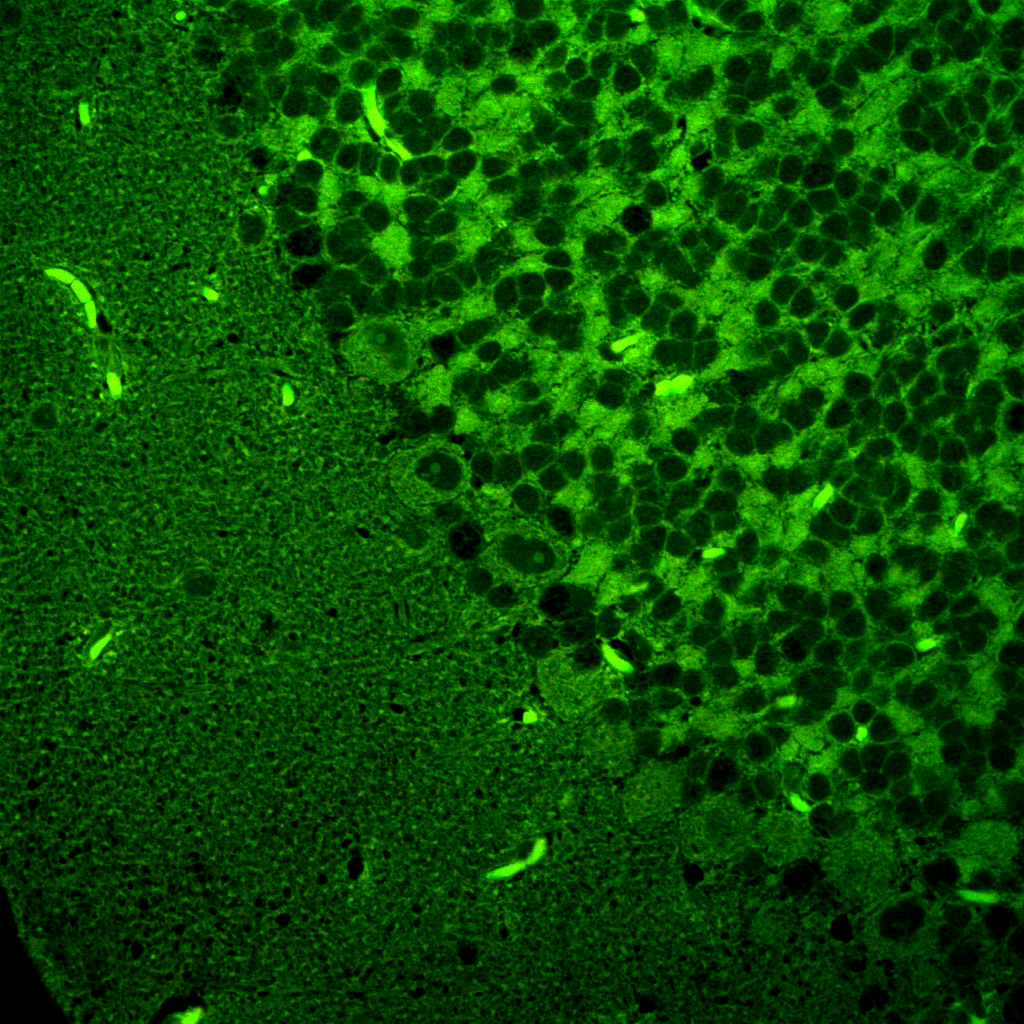

Supplement: Supplementary file 11 — Source data Fig. 10 [file 44318_2024_192_MOESM11_ESM.zip › Figure10/Figure10f/Sibling non-Tg (C57BL6), 9weeks/Merge.tif]

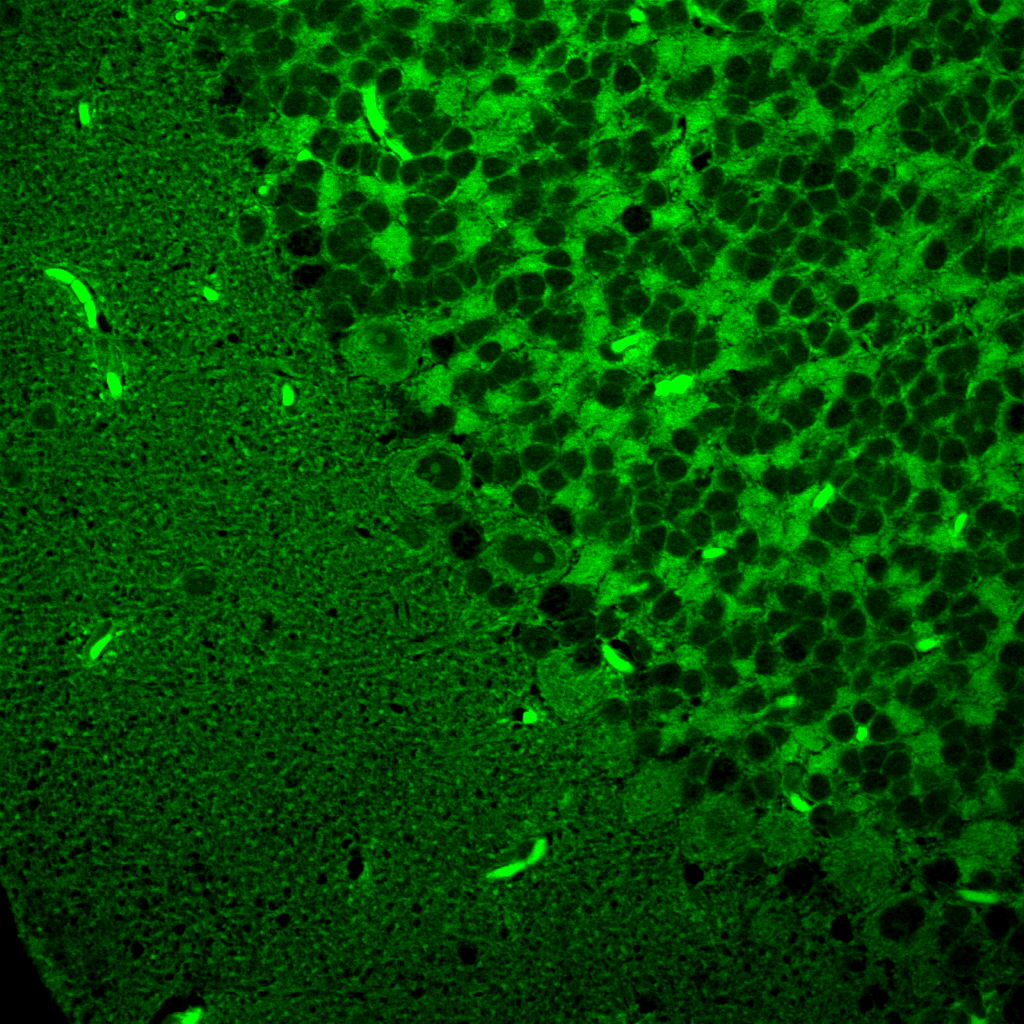

Supplement: Supplementary file 11 — Source data Fig. 10 [file 44318_2024_192_MOESM11_ESM.zip › Figure10/Figure10f/Sibling non-Tg (C57BL6), 9weeks/PQBP3.tif]

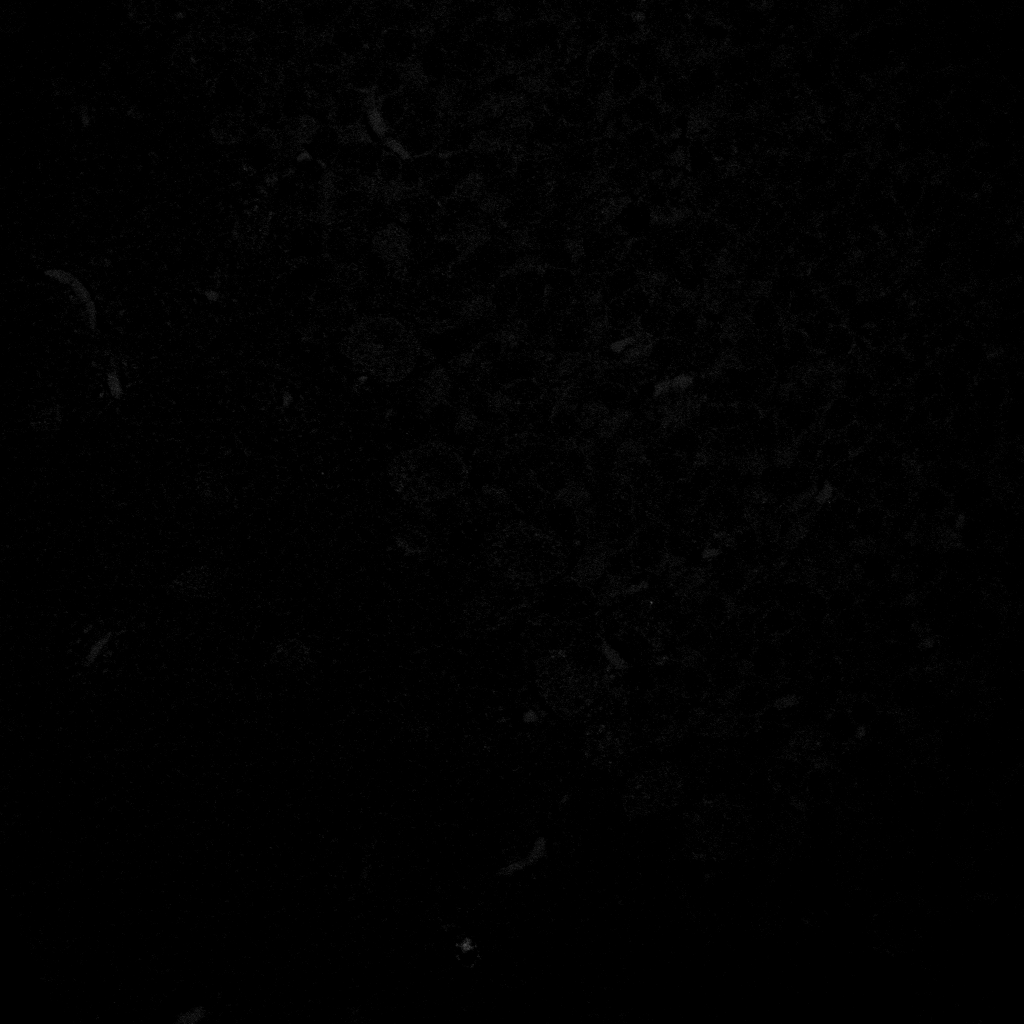

Supplement: Supplementary file 11 — Source data Fig. 10 [file 44318_2024_192_MOESM11_ESM.zip › Figure10/Figure10f/Sibling non-Tg (C57BL6), 9weeks/ubquitin.tif]
