# Supplementary material for: PQBP3 prevents senescence by suppressing PSME3-mediated proteasomal Lamin B1 degradation
Source: EMBO J. 2024 Aug 5;43(18):3968–99. doi: 10.1038/s44318-024-00192-4 (PMC11405525; doi:10.1038/s44318-024-00192-4)
Supplement: Supplementary file 13 — Expanded View Figures [file 44318_2024_192_MOESM13_ESM.pdf]

Expanded View Figures

Figure EV1. PQBP3/NOL7 puncti in the nucleoplasm and cytoplasm.

(**A**) Diameters of small speckles of PQBP3/NOL7 were quantified in images obtained by SRM. Box plots show the median and 25–75th percentile, and whiskers represent data outside the 25–75th percentile range. (**B**) PQBP3/NOL7 is decreased in the nucleus and overall at high cell densities. Representative images of PQBP3/NOL7 and Hoechst 33342 containing at four different cell densities. (**C**) Quantitative analyses of PQBP3/NOL7 signal intensities per cell (left) and per nucleus (right) are shown in graphs. Box plots show the median and 25–75th percentile, and whiskers represent 1.5x inter-quartile range. Source data are available online for this figure.

© The Author(s)

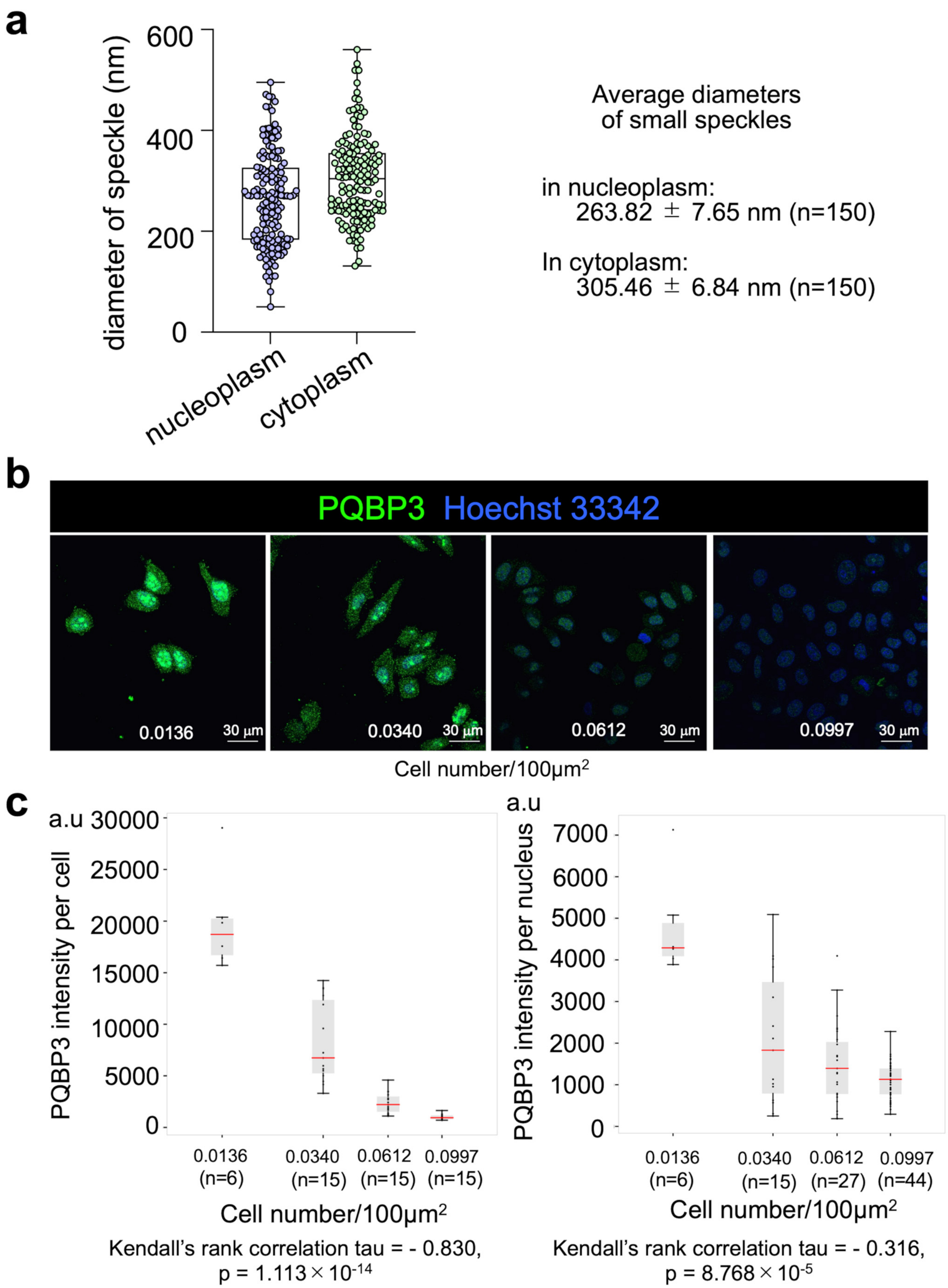
a
600
400
200
0
diameter of speckle (nm)
nucleoplasm
cytoplasm
Average diameters
of small speckles
in nucleoplasm:
263.82 ± 7.65 nm (n=150)
In cytoplasm:
305.46 ± 6.84 nm (n=150)
b
PQBP3 Hoechst 33342
0.0136 30 μm
0.0340 30 μm
0.0612 30 μm
0.0997 30 μm
Cell number/100μm2
c
a.u
PQBP3 intensity per cell
30000
25000
20000
15000
10000
5000
0
0.0136 (n=6)
0.0340 (n=15)
0.0612 (n=15)
0.0997 (n=15)
Cell number/100μm2
Kendall's rank correlation tau = - 0.830,
p = 1.113 × 10-14
a.u
PQBP3 intensity per nucleus
7000
6000
5000
4000
3000
2000
1000
0
0.0136 (n=6)
0.0340 (n=15)
0.0612 (n=27)
0.0997 (n=44)
Cell number/100μm2
Kendall's rank correlation tau = - 0.316,
p = 8.768 × 10-5

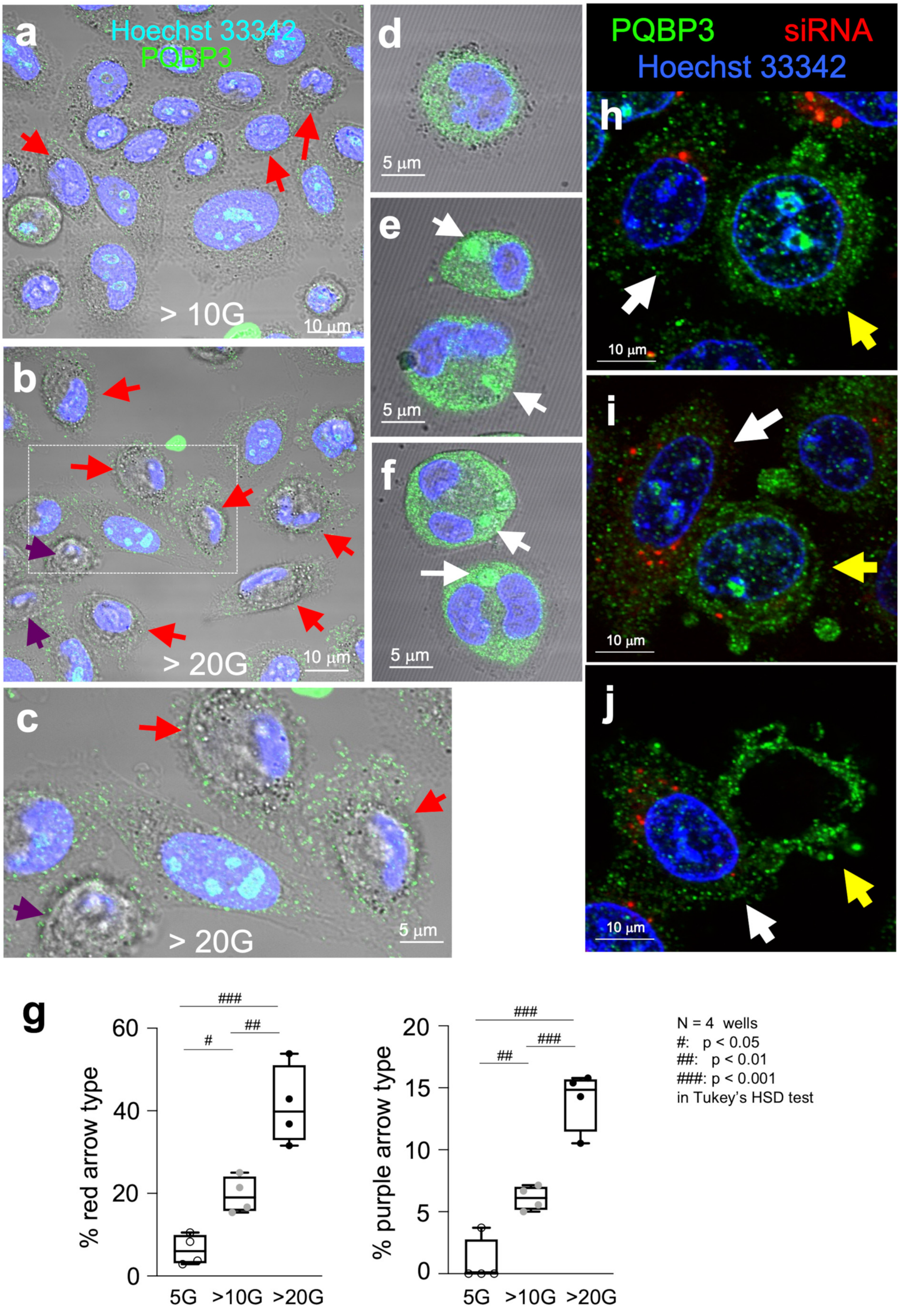
a
Hoechst 33342
PQBP3
> 10G
10 μm
b
> 20G
10 μm
c
> 20G
5 μm
d
5 μm
e
5 μm
f
5 μm
PQBP3
siRNA
Hoechst 33342
h
10 μm
i
10 μm
j
10 μm
g
% red arrow type
% purple arrow type
5G
>10G
>20G
#
##
###
N = 4 wells
#: p < 0.05
##: p < 0.01
###: p < 0.001
in Tukey's HSD test

Figure EV2. PQBP3/NOL7 in senescence.

(**A**) Confocal microscopy of HeLa cells after ten passages (10G), which were penetrated with Tween20, immunostained with anti-PQBP1, and costained with Hoechst 33342. Red arrows indicate cells with dispersed nucleolar PQBP3/NOL7 staining. (**B**) Confocal microscopy images of HeLa cells after 20 passages (20G) stained as described above. Red arrows indicate cells with dispersed nucleolar PQBP3/NOL7 staining, and chromatin distribution (Hoechst 33342-stained area) shifted and deviated in the nucleus. Purple arrows indicate cells in which chromatin was nearly absent. (**C**) Enlarged image of the area indicated by dotted lines in (**B**). (**D–F**) Specific distributions of PQBP3/NOL7 during cell division. Foci of PQBP3/NOL7 localized to the centrosome (white arrow) were observed in addition to the diffuse cytoplasmic distribution. (**G**) Quantitative analyses of percentage of red arrow type or purple arrow type of cells in three different passage groups. Box plots show the median and 25–75th percentile, and whiskers represent data outside the 25–75th percentile range. In red arrow type of cells, statistical significance was found in comparison of <5G and >10G (#: $p = 0.0415$), <5G and >20G (###: $p < 0.0001$), and >10G and >20G (##: $p = 0.0027$). In purple arrow type of cells, statistical significance was found in comparison of <5G and >10G (##: $p = 0.0082$), <5G and >10G (###: $p < 0.0001$), and >10G and >20G (###: $p = 0.0005$). (**H–J**) Yellow arrows indicate cells exhibiting morphological changes of cell death, in which siPQBP3 signals (red) were absent or low, and PQBP3 signals (green) were relatively high. Contrastingly, siPQBP3-transfected cells with high red signals and low green signals did not exhibit such changes or apoptotic features (white arrows). Source data are available online for this figure.

© The Author(s)

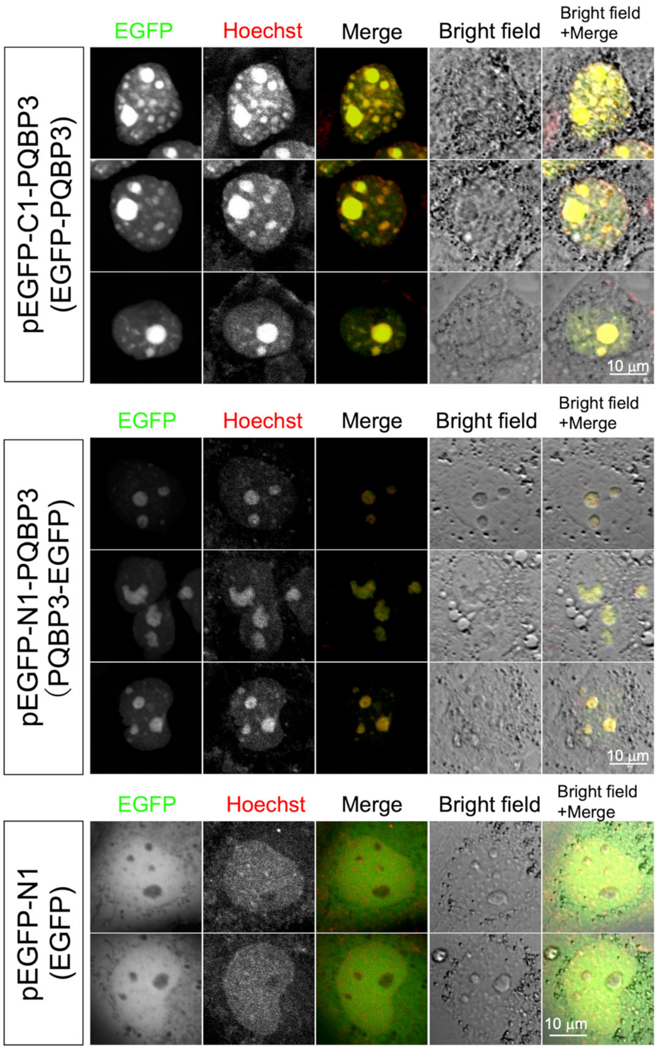

Figure EV3. Expression patterns of PQBP3/NOL7 fusion proteins.

HeLa cells were transfected with pEGFP-C1-PQBP3, pEGFP-N1-PQBP3, or pEGFP-N1 plasmids to express EGFP-PQBP3, PQBP3-EGFP, or EGFP proteins, and following Hoechst 33342 staining without fixation, EGFP signals in live cells were observed with confocal microscopy. Similar expression patterns were observed in EGFP-PQBP3 and PQBP3-EGFP fusion proteins. EGFP protein alone did not exhibit the nucleolar pattern of the PQBP3 fusion proteins. Some cell images are redisplayed from Fig. 7B. Source data are available online for this figure.

© The Author(s)

a

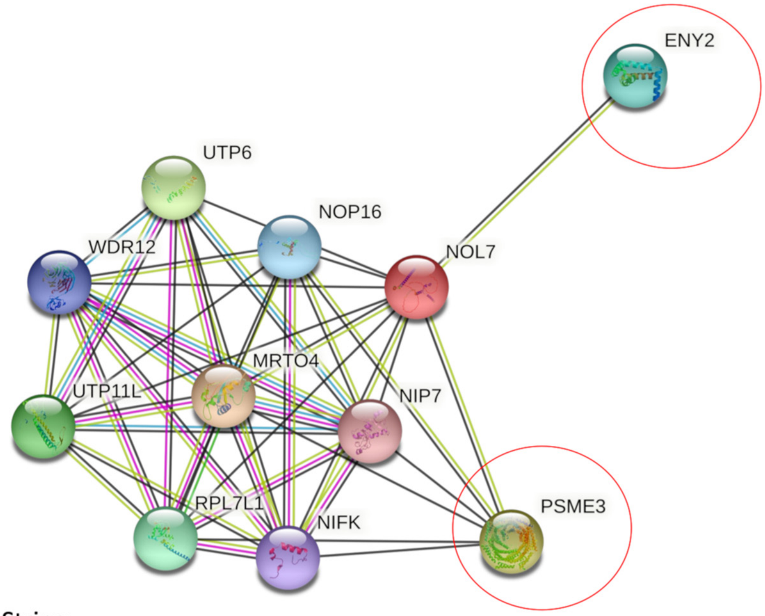

Information by String

Proteasome activator complex subunit 3; Subunit of the 11S REG-gamma (also called PA28-gamma) proteasome regulator, a doughnut-shaped homoheptamer which associates with the proteasome. 11S REG-gamma activates the trypsin-like catalytic subunit of the proteasome but inhibits the chymotrypsin-like and postglutamyl-preferring (PGPH) subunits. Facilitates the MDM2-p53/TP53 interaction which promotes ubiquitination- and MDM2-dependent proteasomal degradation of p53/TP53, limiting its accumulation and resulting in inhibited apoptosis after DNA damage. May also be involved in cell cycle regul [...]
Identifier: ENSP00000293362, **PSME3**
Organism: Homo sapiens

Transcription and mRNA export factor ENY2; Involved in mRNA export coupled transcription activation by association with both the TREX-2 and the SAGA complexes. The transcription regulatory histone acetylation (HAT) complex SAGA is a multiprotein complex that activates transcription by remodeling chromatin and mediating histone acetylation and deubiquitination. Within the SAGA complex, participates in a subcomplex that specifically deubiquitinates both histones H2A and H2B. The SAGA complex is recruited to specific gene promoters by activators such as MYC, where it is required for trans [...]
Identifier: ENSP00000429986, **ENY2**
Organism: Homo sapiens

b

PSME3

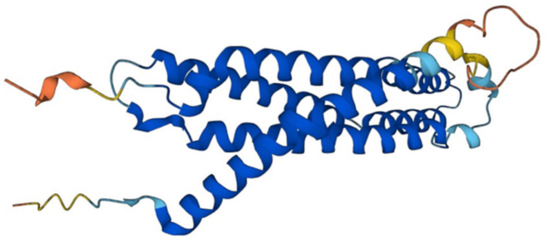

https://alphafold.ebi.ac.uk/entry/Q967U1

c

PSME3

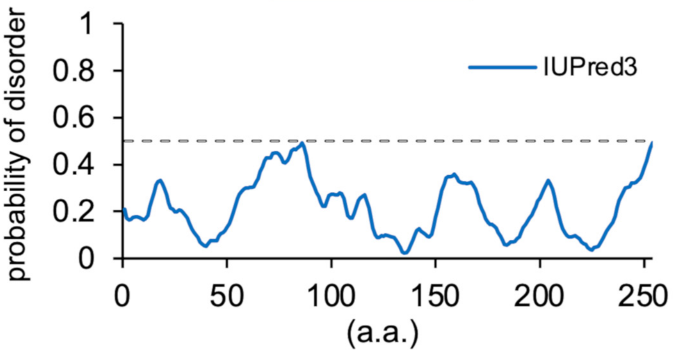

IUPred2A (https://iupred2a.elte.hu/)

© The Author(s)

Figure EV4. PSME3 as a candidate proteins interacting with PQBP3/NOL7.

(**A**) String (version 11.5) (https://string-db.org/) was used to predict interacting proteins with PQBP3/NOL7, and their descriptions in String are shown. (**B**) PSME3 protein structure predicted by alphafold. (**C**) PSME3 IDP prediction by IUPred2A.

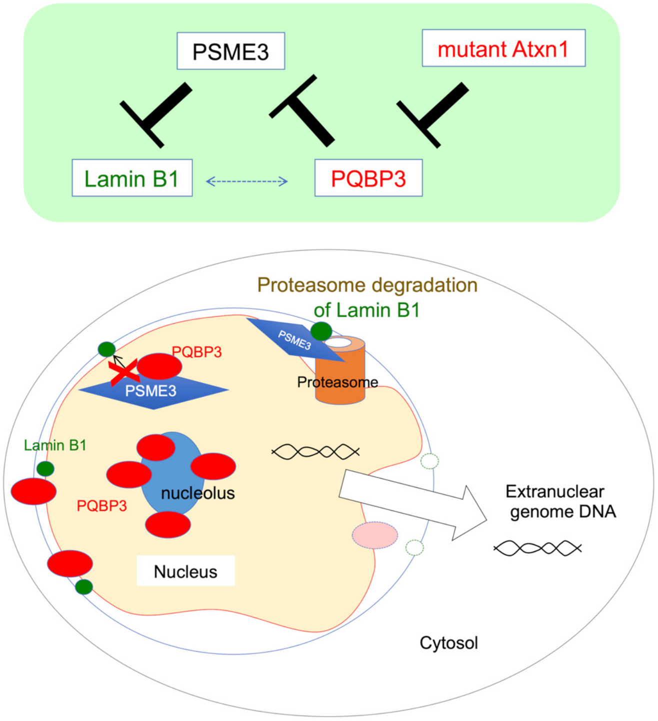

Figure EV5. Hypothesized mechanism for nuclear membrane instability mediated by PQBP3/NOL7 and PSME3.

Hypothesized mechanism of nuclear membrane instability under senescence, as suggested by the results of the present study. The upper panel illustrates the interaction and suppression relationships between PQBP3/NOL7, PSME3, Lamin B1, and mutant Atxn1. Under physiological conditions, PQBP3 complexes with PSME3 to suppress its protein degradation activity. In senescence, PQBP3 is decreased and not supplied sufficiently to the nuclear membrane for inhibition of PSME3-mediated proteasomal degradation of Lamin B1. In the case Lamin B1 is degraded, the nuclear membrane is instabilized, allowing release of genomic DNA from the nucleus to the cytosol.

 © The Author(s)
